# Transcriptome analyses reveal molecular mechanism underlying tapping panel dryness of rubber tree (*Hevea brasiliensis*)

**DOI:** 10.1038/srep23540

**Published:** 2016-03-23

**Authors:** Dejun Li, Xuncheng Wang, Zhi Deng, Hui Liu, Hong Yang, Guangming He

**Affiliations:** 1 Key Laboratory of Biology and Genetic Resources of Rubber Tree, Ministry of Agriculture, Rubber Research Institute, Chinese Academy of Tropical Agricultural Sciences, Baodao Xincun, Danzhou, Hainan 571737, China; 2 Tsinghua-Peking Center for Life Sciences, Center for Plant Biology, School of Life Sciences, Tsinghua University, Tsinghua Park No. 1, Haidian District, Beijing 100084, China; 3 State Key Laboratory of Protein and Plant Gene Research, Peking-Tsinghua Center for Life Sciences, School of Advanced Agriculture Sciences and School of Life Sciences, Peking University, No. 5 Yiheyuan Road, Haidian District, Beijing 100871, China

## Abstract

Tapping panel dryness (TPD) is a serious threat to natural rubber yields from rubber trees, but the molecular mechanisms underlying TPD remain poorly understood. To identify TPD-related genes and reveal these molecular mechanisms, we sequenced and compared the transcriptomes of bark between healthy and TPD trees. In total, 57,760 assembled genes were obtained and analyzed in details. In contrast to healthy rubber trees, 5652 and 2485 genes were up- or downregulated, respectively, in TPD trees. The TPD-related genes were significantly enriched in eight GO terms and five KEGG pathways and were closely associated with ROS metabolism, programmed cell death and rubber biosynthesis. Our results suggest that rubber tree TPD is a complex process involving many genes. The observed lower rubber yield from TPD trees might result from lower isopentenyl diphosphate (IPP) available for rubber biosynthesis and from downregulation of the genes in post-IPP steps of rubber biosynthesis pathway. Our results not only extend our understanding of the complex molecular events involved in TPD but also will be useful for developing effective measures to control TPD of rubber trees.

Natural rubber (*cis*-1,4-polyisoprene) is one of the most important raw materials in the world, and it cannot be replaced by synthetic alternatives for all applications owing to its unique properties including resilience, elasticity, impact and abrasion resistance, efficient heat dispersion and malleability at cold temperature^1^,^2^. Worldwide, more than 2000 plant species produce natural rubber, but the rubber tree (*Hevea brasiliensis*) is the only economically viable source. Although indigenous to the Amazonian rainforest, the rubber tree is widely cultivated in Southeast Asia. With the fast development of the world economy, the demand for natural rubber has increased continuously. Rubber tree plantations must be located in sub-tropical to tropical zones, and therefore increasing rubber yield per hectare is an effective means of meeting world demand. Rubber yield has increased substantially in recent years owing to the cultivation of high-yield clones and the utilization of ethephon (an ethylene generator), but yield can be negatively affected by tapping panel dryness (TPD). It is estimated that TPD-related losses account for 12–14% of natural rubber yield^3^.

The first symptom of TPD is the occurrence of partially dry zones along the tapping panel. In the advanced stage, the tapping panel is completely dry and lacks latex flow^4^. It has been suggested that TPD is a complex physiological disorder caused by overexploitation, i.e., excessive tapping and/or overstimulation with ethylene^5^,^6^,^7^. In contrast to healthy rubber tree, the content of some antioxidant substances including thiols, ascorbic acids, variable peroxidase and superoxide dismutase (SOD) are reduced in TPD rubber tree^5^,^8^. In laticifer cells, excessive oxidative stress can destabilize cellular membranes and trigger the lutoid burst with consequent latex coagulation *in situ*^6^. By investigating the histopathology and cytopathology of trunk phloem necrosis, de Faÿ concluded that trunk phloem necrosis is identical to or a variant of TPD and that it is a degenerative disease of the rubber tree trunk phloem somewhat resembling the plant stress response, programmed cell death (PCD), and plant tumourigenesis^9^.

Recent studies on TPD have focused mainly on identifying and characterizing TPD-related genes. Chen *et al.* reported that expression of the *H. brasiliensis* transcription factor gene *HbMyb1* is significantly decreased in the bark and latex of TPD trees^10^. Transgenic tobaccos overexpressing *HbMyb1* effectively suppressed cell death induced by various stresses^11^. Venkatachalam *et al.* suggested that upregulation of certain TPD-related genes might trigger PCD in rubber trees^12^. In 2009, they also reported that downregulation of *HbTOM20* (encoding a translocase of the mitochondrial outer membrane) in TPD trees might alter mitochondrial metabolism and result in impaired latex biosynthesis^13^. By identifying and analyzing TPD-related genes, Li *et al.* first proposed that the production and scavenging of reactive oxygen species (ROS), the ubiquitin proteasome pathway (UPP), PCD, and rubber biosynthesis (RB) pathways might play important roles in rubber tree TPD^14^. Gébelin *et al.* found that the target genes of TPD-related microRNAs are associated with RB, ROS-scavenging systems, and PCD^15^. The TPD-related genes also have been identified and analyzed with rubber tree oligo microarrays^16^,^17^. Li *et al.* characterized a novel translationally controlled tumor protein of rubber tree (*HbTCTP1*). The TCTP is a multi-functioning protein that carries out vita roles in various life processes, and we propose that HbTCTP1 may carry out antioxidant function during TPD^18^. Putranto *et al.* suggested that the candidate TPD-related genes in latex and phloem tissues are associated with ROS-scavenging, ethylene biosynthesis, and signaling^19^. Using the rubber tree clone PR 107 for RNA sequencing (RNA-seq), Liu *et al.* identified and analyzed TPD-related genes by comparing transcriptomes between healthy and TPD trees. They proposed that genes involved in RB and jasmonate synthesis are associated with TPD occurrence^20^.

Although some progress has been made with regard to understanding TPD, the underlying molecular mechanisms TPD remain largely unknown; in this respect, a prerequisite is to identify TPD-related genes and analyze their expression patterns and functions. It is well established that TPD has a long-term course comprising initial and advanced stages. The susceptibility to TPD depends on the nature of rubber tree variety. The rubber tree clone RY 7-33-97, cultivated by Rubber Research Institute, Chinese Academy of Tropical Agricultural Sciences, has been widely planted in China, and its susceptibility to TPD is higher than that of PR 107, which is planted on a small scale in China. The TPD-related genes identified to date are insufficient to completely elucidate the molecular mechanisms underlying TPD. With these considerations, we sequenced, assembled and compared the bark transcriptomes between healthy and TPD trees to elucidate the expression profiles of TPD-related genes. Our study provides new insights into the potential molecular mechanisms involved in rubber tree TPD.

## Results

### Rubber Tree Transcriptomes Sequencing, Assembly and Characterization

In this study, the TPD rubber trees were selected from the initial stage of TPD. We extracted total RNA from the bark of healthy and TPD trees, constructed RNA-seq libraries, and sequenced the transcriptomes using the Illumina paired-end sequencing approach. After removing the adaptors, low-quality, and contaminated reads, 108,167,188 high-quality clean reads were obtained and used to assemble bark transcriptome with SOAPdenovo2^21^. Ultimately, 57,760 unigenes comprising ~26.49 Mb were generated. The assembled unigenes and sequencing reads were submitted to the NCBI Transcriptome Shotgun Assembly (TSA) project, and the assembled unigenes not conforming to TSA standards are shown in Supplementary Dataset 1. The N50 and the average length of assembled unigenes were ~311 bp and 459 bp, respectively, and 15,659 unigenes (27.11%) were >500 bp (Table 1). Among the assembled unigenes, 43,098 (~74.62%) did not contain a gap region, whereas the remaining unigenes (~25.38%) were filled with Ns. Of the 57,760 unigenes, 46,485 contained coding sequences as determined by searching unigene sequences against protein databases using blastx (e-value <0.00001) or by predicting with ESTScan 3.0 (http://estscan.sourceforge.net/) (Table 1).

**Figure 1:**
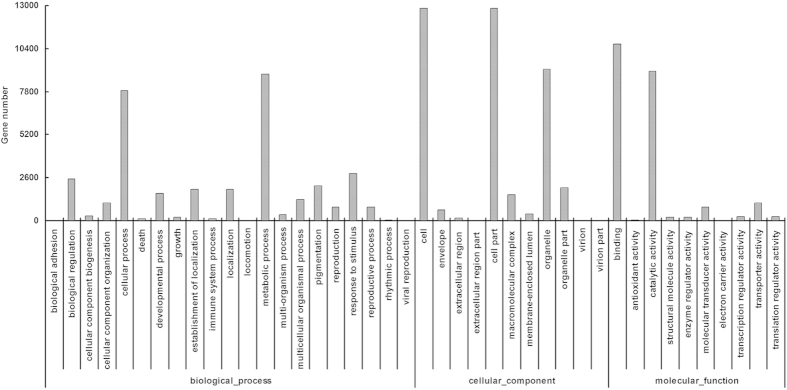
GO classifications of the assembled unigenes identified via transcriptome analysis of rubber tree bark.

To facilitate the global analysis of the bark transcriptome, all predicted unigenes were assigned to different functional categories with Blast2GO. The annotations were manually verified and integrated by gene ontology (GO) classification. Among the 57,760 assembled unigenes, 22,479 were categorized into 42 GO terms based on nucleotide sequence identity. Three main categories, namely biological process, molecular function and cellular component, contained 21, 10, and 11 GO terms, respectively (Fig. 1). Metabolic process (8872 genes) was the predominant term in the category of biological process; the terms binding (10,696 genes) and cell part (12,824 genes) were predominant in the categories of molecular function and cellular component, respectively. To identify the biological pathways enriched in rubber tree bark, we also mapped all the assembled unigenes to reference canonical pathways in the Kyoto Encyclopedia of Genes and Genomes (KEGG). Of the 57,760 assembled unigenes, 17,991 were assigned to 119 KEGG pathways (Supplementary Dataset 2). Among these, the metabolic pathway was the most represented (3015 genes, 16.76%), followed by biosynthesis of secondary metabolites (1462, 8.13%), plant-pathogen interaction (1408, 7.83%), spliceosome (947, 5.26%), ubiquitin mediated proteolysis (425, 2.36%), protein processing in endoplasmic reticulum (424, 2.36%), endocytosis (352, 1.96%), and starch and sucrose metabolism (341, 1.90%), etc. (Supplementary Dataset 2). These results from GO and KEGG analyses indicated that metabolic process genes are highly expressed in rubber tree bark.

**Figure 2:**
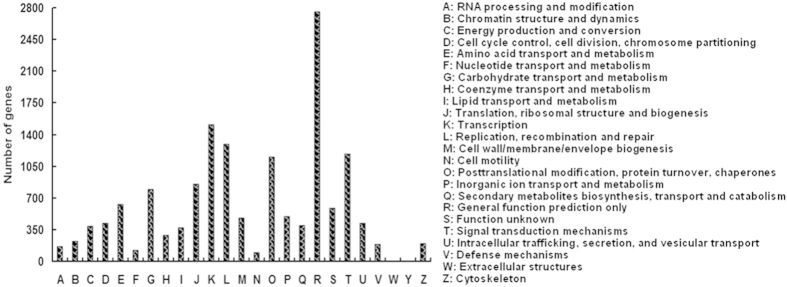
Histogram for COG classification of the assembled unigenes.

The Clusters of Orthologous Groups (COG) protein database facilitates the phylogenetic classification of all proteins encoded in a complete genome, and it reflects one-to-many and many-to-many orthologous relationships as well as simple one-to-one relationships. As Fig. 2 shows, 23,278 unigenes were assigned to 25 COG categories. The general function prediction only was the largest group (2756 genes, 18.28%), followed by transcription (1516, 10.05%), replication, recombination and repair (1299, 8.61%), signal transduction mechanisms (1186, 7.86%), posttranslational modification, protein turnover and chaperones (1155, 7.66%), translation, ribosomal structure and biogenesis (856, 5.68%), carbohydrate transport and metabolism (799, 5.30%), etc. Interestingly, we found that 398 unigenes (~2.64%) were classified into the group of secondary metabolite biosynthesis, transport and catabolism. Because RB is a secondary metabolic process, some of these genes might be involved in RB.

### Identification of Differential Expressed Genes (DEGs) between Healthy and TPD Trees

**Figure 3:**
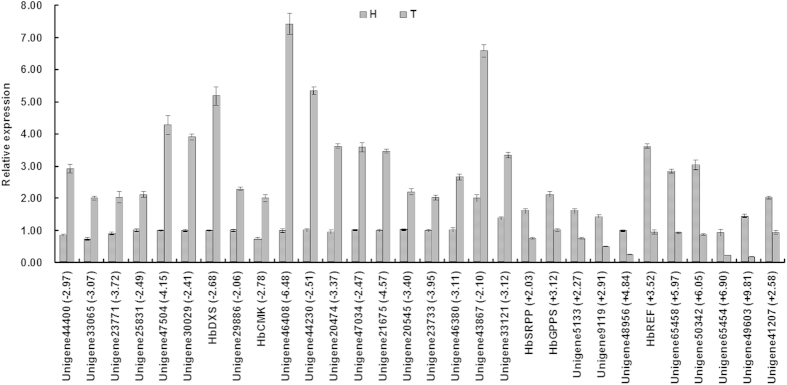
Real-time RT-PCR validation of the DEGs. Total RNA extracted from healthy and TPD rubber trees was used for real-time RT-PCR analyses, and *18s rRNA* was used as the internal reference. H and T represent healthy and TPD rubber trees, respectively. Data represent the mean ± S.D. The number in bracket refers to the log2(H_RPKM/T_RPKM) of the corresponding gene from RNA-seq analyses.

With 57,760 unigenes as the reference transcriptome, the high-quality clean reads from healthy and TPD tree bark were realigned. The healthy and TPD tree transcriptomes were sequenced with two biological replicates. The Pearson correlation R value between the two healthy rubber tree samples was ~0.79, and the value was ~0.88 between the two TPD tree samples. To maximize accuracy and reliability, the overlapping DEGs from each of the two biological replicates were used for subsequent analyses. With a q-value ≤0.001 and the absolute value of log_2_ ratio ≥2 as thresholds, a total of 8,137 TPD-related genes were identified as DEGs between healthy and TPD trees, among which 5652 and 2485 were up- or downregulated, respectively, in TPD tree bark compared with healthy tree bark (Supplementary Dataset 3). The expression of 30 TPD-related genes, including 25 randomly selected TPD-related genes and 5 TPD-related genes involved in rubber biosynthesis, was further analyzed with real-time reverse transcription-PCR (RT-PCR). As shown in Fig. 3, the expression profiles of all 30 genes were consistent with those from RNA-seq, suggesting that the TPD-related genes we identified through RNA-seq were highly accurate and reliable.

### Functional Classification and Characterization of TPD-related Genes

**Figure 4:**
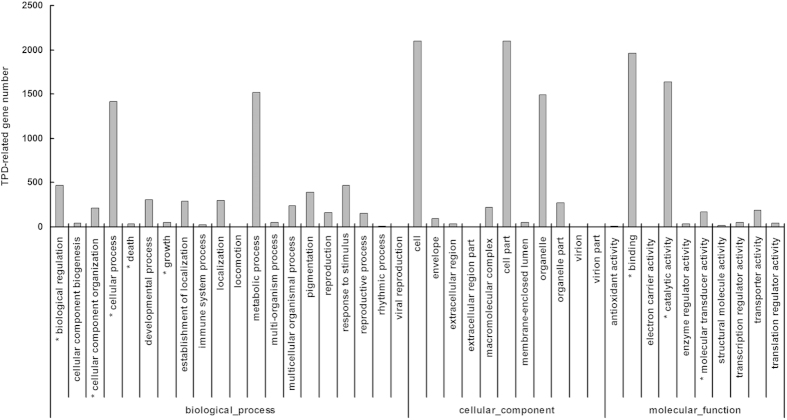
GO classifications of the TPD-related genes identified in this study. Asterisks indicate the enriched GO terms.

Based on sequence identity, the 8137 TPD-related genes identified above were classified into different GO terms. In the cellular component group, the TPD-related genes were associated with 11 GO terms such as macromolecular complex, antioxidant activity, and extracellular region, among which no GO terms were significantly enriched (Fig. 4). In the molecular function group, the TPD-related genes were associated with 9 GO terms such as binding, electron carrier activity, and transcription regulator activity, among which 3 terms were significantly enriched, i.e., binding, molecular transducer activity, and catalytic activity (Fig. 4). In the biological process group, the TPD-related genes were categorized in 21 GO terms such as growth, immune system process, and biological regulation, among which 5 terms were significantly enriched, i.e., biological regulation, cellular component organization, cellular process, death, and growth (Fig. 4).

To further understand the functions of TPD-related genes, we mapped all TPD-related genes to KEGG pathways and compared them with the whole-transcriptome background, with the goal of searching for genes involved in metabolic or signal transduction pathways that were significantly enriched. The TPD-related genes were classified into 115 KEGG pathways, such as ABC transporters, ubiquitin mediated proteolysis, endocytosis, starch and sucrose metabolism, and glutathione metabolism (Supplementary Table 1), among which the metabolic pathways term was the most dominant, followed by biosynthesis of secondary metabolites, spliceosome, endocytosis, ubiquitin mediated proteolysis, starch and sucrose metabolism, and RNA degradation, etc. (Supplementary Table 1). With a q-value ≤0.05 as the threshold, the TPD-related genes were significantly enriched in the 5 KEGG pathways plant-pathogen interaction, ABC transporters, monoterpenoid biosynthesis, base excision repair, and caffeine metabolism (Supplementary Table 1), suggesting their important roles in TPD occurrence.

**Figure 5:**
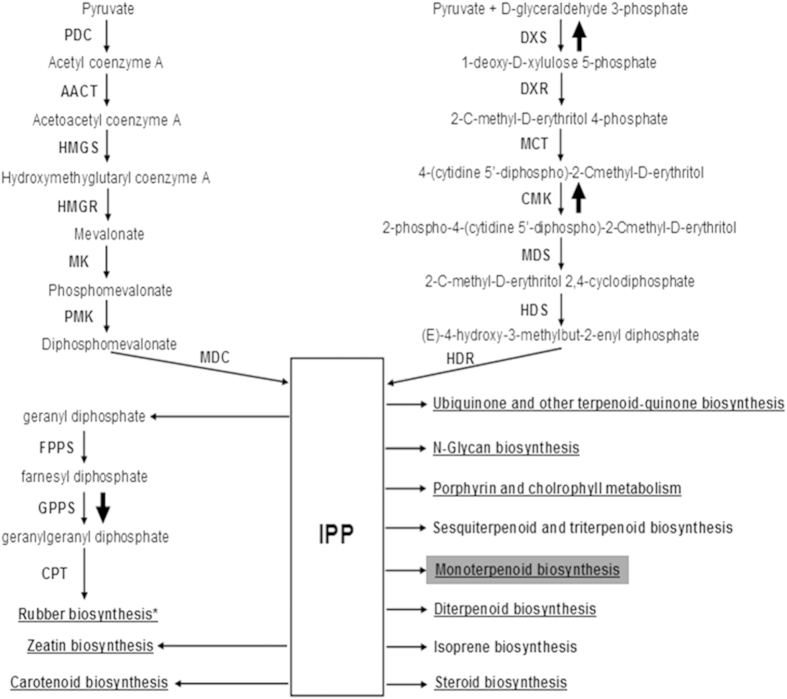
General pathway for isoprenoid biosynthesis and the expression profiles of TPD-related genes. IPP is a common intermediate for numerous isoprenoids and may be generated via the mevalonate (upper-left) or methylerythritol phosphate (upper-right) pathway in rubber trees. The IPP-requiring KEGG pathways that utilize IPP to synthesize different classes of isoprenoids are shown. The isoprenoid end-product KEGG pathways containing the assembled unigenes and DEGs identified in this study are underlined. The IPP-requiring KEGG pathway (monoterpenoid biosynthesis) in which the DEGs were enriched is highlighted in gray. PDC, pyruvate dehydrogenase complex; AACT, acetyl coenzyme A acetyltransferase; HMGS, hydroxymethyglutaryl coenzyme A synthase; HMGR, hydroxymethyglutaryl coenzyme A reductase; MK, mevalonate kinase; PMK, phosphomevalonate hosphomevalonate kinase; MDC, diphosphomevalonate decarboxylase; DXS, 1-deoxy-D-xylulose 5-phosphate synthase; DXR, 1-deoxy-D-xylulose 5-phosphate reductoisomerase; MCT, 2-C-methyl-D-erythritol 4-phosphate cytidylyltransferase; MDS, 4-(cytidine 5′-diphospho)-2-Cmethyl-D-erythritol kinase; CMK, 2-C-methyl-D-erythritol 2,4-cyclodiphosphate synthase; HDS, 4-hydroxy-3-methylbut-2-enyl diphosphate synthase; HDR, 4-hydroxy-3-methylbut-2-enyl diphosphate reductase; IPP, isopentenyl diphosphate; FPPS, farnesyl pyrophosphate synthase; GPPS, geranylgeranyl pyrophosphate synthase; CPT, *cis*-prenyltransferase. Compared with healthy trees, the upward and downward bold arrows indicate upregulated and downregulated of TPD-related genes, respectively, in TPD trees. *The locations of the two TPD-related genes *HbREF* and *HbSRPP* were not confirmed, and therefore they are not indicated in the RB pathway.

In 11 of the KEGG pathways, isopentenyl diphosphate (IPP) is utilized for the synthesis of different classes of isoprenoids including rubber. Interestingly, 62 TPD-related genes identified in this study were found to be involved in 9 IPP-requiring KEGG pathways and were enriched in monoterpenoid biosynthesis (Fig. 5). Of these 62 genes, 50 and 12 were up- or downregulated, respectively, in TPD trees compared to healthy trees. All the TPD-related genes involved in N-Glycan biosynthesis, monoterpenoid biosynthesis, and steroid biosynthesis were upregulated in TPD trees. In the KEGG pathways of zeatin biosynthesis, porphyrin and chlorophyll metabolism, carotenoid biosynthesis, and diterpenoid biosynthesis, the number of upregulated TPD-related genes in TPD trees was significantly greater than the number downregulated. The TPD-related gene in ubiquinone and other terpenoid-quinone biosynthesis was downregulated in TPD trees (Fig. 5). In all 8 KEGG pathways mentioned above, IPP was utilized to synthesize different isoprenoids, and therefore the expression change of TPD-related genes involved in these KEGG pathways may affect RB. Consistent with our speculation, we identified 5 TPD-related DEGs in the RB pathway. Compared with healthy rubber trees, 2 genes (*DXS*, *CMK*) in pre-IPP steps of the RB pathway were upregulated in TPD trees, whereas 3 genes (*GPPS*, *REF*, *SRPP*) involved in post-IPP steps of the RB pathway were downregulated in TPD trees (Fig. 5). As shown in Fig. 3, the expression patterns of all 5 TPD-related genes were validated by real-time RT-PCR, consistent with the RNA-seq results.

## Discussion

RNA-seq is a cost-effective approach for transcriptome analysis. Several studies have reported transcriptome profiling in different tissues of rubber tree^20^,^22^,^23^,^24^,^25^,^26^,^27^,^28^. In our present study, 57,760 unigenes with a total length of ~26.49 Mb were obtained and deposited in the NCBI TSA project, which further expands the rubber tree transcriptome database. These data will be helpful for developing molecular markers and cloning novel genes of rubber tree. Using bark as sequencing material, Liu *et al.* also sequenced, assembled and analyzed rubber tree transcriptomes. In their study, “general function prediction only” was the most highly represented COG group, followed by “translation, ribosomal structure and biogenesis”, and “posttranslational modification, protein turnover, chaperones”^20^. In our study, the “general function prediction only” was also the most dominant COG functional group, followed by “transcription”, “replication, recombination and repair”. Being consistent with Liu *et al.*^20^, our study revealed that “metabolism” was the most dominant KEGG pathway, followed by “biosynthesis of secondary metabolites”, and “plant-pathogen interaction”, suggesting that maintenance of rubber tree bark is highly dependent on metabolic activity.

RNA-seq analysis generated a comprehensive view of TPD-related genes that might participate in regulating TPD occurrence and development. In our study, 5652 and 2485 genes were up- or downregulated, respectively, in TPD tree bark. Liu *et al.* identified 22,577 DEGs associated with TPD using a similar method^20^. Among the 8137 TPD-related genes identified in our study, 2109 were reported to be differentially expressed between TPD and healthy rubber trees by Liu *et al.*^20^. The number of TPD-related genes with consistent or inconsistent expression between our study and that of Liu *et al.* were 1320 and 789, respectively. The inconsistencies may be attributable to the differences in rubber tree variety (RY 7–33–97 vs. PR 107) and/or the TPD stages analyzed (initial stage vs. advanced stage) in the two studies. Our results further underscore the fact that rubber tree TPD is a complex process involving many genes. Hence, future work should focus on the key genes in important TPD-related regulatory pathways (such as PCD, RB, and ROS metabolism, etc.) to elucidate the molecular mechanisms involved in rubber tree TPD. To understand the functional implications of TPD-related genes, 8137 TPD-related genes identified in this study were analyzed for GO terms and KEGG pathways. These TPD-related genes were classified into 41 GO terms including antioxidant activity, response to stimulus, metabolic process, transcription regulator activity, etc. Further analyses indicated that TPD-related genes were significantly enriched in 8 GO terms including binding, catalytic activity, growth, cellular process, cellular component organization, molecular transducer activity, death, and biological regulation. Whereas in Liu *et al.*’s study, the TPD-related genes were mainly associated with GO terms including cell, cell part, binding, catalytic activity, metabolic process, and cellular process^20^.

Of 115 KEGG pathways associated with the TPD-related genes, metabolic pathway was the most dominant, followed by plant-pathogen interaction, biosynthesis of secondary metabolites, spliceosome, endocytosis, ubiquitin mediated proteolysis, starch and sucrose metabolism, and RNA degradation. These predominant 8 KEGG pathways mentioned above were mainly associated with metabolism, PCD, protein degradation, and RNA processing. In addition, the TPD-related genes were significantly enriched in 5 KEGG pathways including plant-pathogen interaction, ABC transporters, monoterpenoid biosynthesis, base excision repair, and caffeine metabolism. Importantly, the TPD-related genes enriched in 5 KEGG pathways above are also associated with the GO terms such as metabolic process, immune system process, antioxidant activity, response to stimulus, death, etc.

As a stimulant of latex production in rubber tree, ethylene has been widely utilized in latex production. Chrestin suggested that rubber tree TPD is associated with ethylene overstimulation^6^. Zeng *et al.* reported that tapping and stimulation with ethylene raises the activity and content of ACC synthetase in rubber tree latex^29^. S-adenosyl-L-methionine not only is involved in caffeine biosynthesis but also participates in ethylene biosynthesis pathway, and therefore caffeine biosynthesis influences ethylene biosynthesis in rubber tree. Putranto *et al.* reported that the candidate genes related to TPD occurrence are associated with ROS-scavenging, ethylene biosynthesis and signaling^19^. In our study, the TPD-related genes were significantly enriched in caffeine metabolism. Based on these results, we speculate that the TPD-related genes in the caffeine metabolism pathway might contribute to TPD occurrence by regulating ethylene biosynthesis in rubber trees.

Some researchers have suggested that PCD might occur in TPD rubber trees^9^,^10^,^11^,^12^,^14^. The typical characteristics of PCD, such as DNA laddering, chromatin condensation, and nuclear membrane blebbing, etc., were have been detected in TPD rubber trees^11^. In contrast to healthy rubber trees, the nucleic acids content is decreased and RNase activities increased in the latex of TPD trees^30^. In this our study, the TPD-related genes were enriched in three KEGG pathways including plant-pathogen interaction, ABC transporters, and base excision repair (BER). Interestingly, all three enriched KEGG pathways were associated with PCD and related pathways. Plant-pathogen interactions contains involve both PAMP (i.e., pathogen-associated molecular pattern)-triggered immunity and effector-triggered immunity. During effector-triggered immunity process, the consequent increase in the cytosolic Ca^2+^ concentration regulates both ROS production and the localized PCD/hypersensitive response^31^. ABC transporters are associated with mRNA translation and DNA repair^32^,^33^. Base excision repair is the predominant DNA damage repair pathway for the processing of small lesions, i.e., those derived from oxidation- and/or alkylation-induced damages. This repair process is critical for removing damaged bases that could otherwise lead to mutations by mispairing or breaks in DNA during replication. The resulting single-strand break can then be processed by either short-patch or long-patch base excision repair^34^. Therefore, the expression changes of TPD-related genes involved in three enriched KEGG pathways might be associated with PCD, DNA laddering, and reduced nucleic acids content in TPD trees.

In rubber tree, IPP is generated by the methylerythritol phosphate and mevalonate pathways. Chow *et al.* suggested that subcellular compartmentalization of IPP for RB correlates with carotenoid synthesis in plastids^35^. When rubber trees require a higher carotenoid content, the mevalonate pathway supplies IPP for RB in the cytosol while the methylerythritol phosphate pathway supplies IPP for carotenoid biosynthesis in Frey-Wyssling particles. Once rubber trees no longer require a high carotenoid content, the IPP for RB is supplied by both methylerythritol phosphate and mevalonate pathways^35^. In our study, we found that two genes in the methylerythritol phosphate pathway (*DXS* and *CMK*) were upregulated in TPD trees compared with healthy trees. Lois *et al.* suggested that DXS might catalyze the first potential regulatory step in carotenoid biosynthesis^36^. Moreover, of 15 TPD-related genes identified in carotenoid biosynthesis pathway, 13 were upregulated in TPD trees, which might result in higher carotenoid content. ROS, as potentially toxic molecules, are capable of injuring cells. It was reported that uncompensated oxidative stress might be involved in the onset of TPD^7^. As important antioxidants, carotenoids can react with ROS, thereby eliminating destructive potential of ROS on essential biomolecules^37^. Therefore, we speculate that carotenoid production is upregultated in TPD trees to enhance antioxidant activity and thereby counter oxidative stress.

The first symptom of TPD is partial dry zones along the tapping panel. In the advanced stage, the tapping panel is completely dry and lacks latex flow^4^. ABC transporters are transmembrane proteins that use the energy of ATP hydrolysis to perform certain biological processes including translocation of various substrates^32^,^33^. Therefore the translocation efficiency of various substrates necessarily impacts metabolic processes including RB. In our study, the TPD-related genes were associated with 9 IPP-requiring KEGG pathways including RB; all these 9 pathways utilize IPP to synthesize isoprenoids, and therefore the IPP utilization of the other 8 (non-RB) pathways would necessarily affect RB. Besides pathways for the biosynthesis of ubiquinone and other terpenoid-quinones, all or most of the TPD-related genes in the other 7 IPP-requiring KEGG pathways were upregulated in TPD trees, suggesting the upregulation of isoprenoid biosynthesis. Consequently, the IPP available for RB probably decreased, which necessarily resulted in lesser RB in TPD trees. Consistent with this conclusion, three genes (*GPPS*, *REF*, *SRPP*) involved in post-IPP steps of RB were downregulated in TPD trees. Li *et al.* also found that *REF* and *SRPP* were downregulated in the latex of TPD trees^14^. Priya *et al.* reported that *REF* expression in latex of high-yield rubber clones was significantly higher than that in a low-yield clone^38^. Based on these results, we suggest that the observed reduction in rubber yield in TPD trees mainly results from reduced IPP available for RB and downregulation of the genes involved in the post-IPP steps of RB. Consistent with our results, Liu *et al.* reported that genes involved in RB (as well as those for jasmonate biosynthesis) were associated with TPD occurrence^20^.

Our results as well as those from previous studies related to rubber tree TPD suggest that TPD is a complex process involving many genes. The TPD-related genes we identified are mostly associated with ROS metabolism, jasmonate and ethylene biosyntheses, UPP, PCD, and RB. Based on all these results, we speculate that overexploitation (excessive tapping as well as overstimulation with ethylene) induces excessive ROS production, which alters the balance between ROS production and scavenging and results in ROS accumulation. As toxic molecules, the excessive ROS further results in PCD and UPP, which necessarily affect RB. To protect itself against further damage, the rubber tree initiates PCD, and latex yield decreases (or even stops), resulting in TPD.

## Methods

### Plant Materials and RNA Extraction

**Figure 6:**
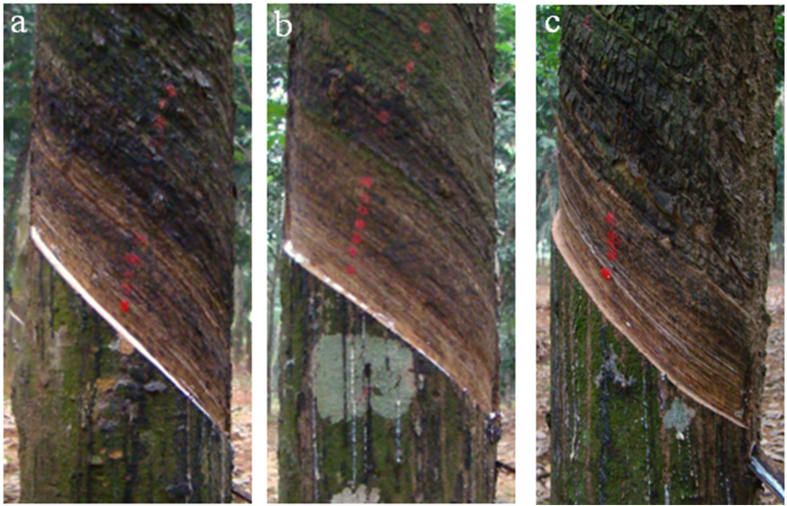
Images of healthy and TPD rubber trees. (**a**) A healthy rubber tree with normal latex flow. (**b**) A rubber tree partially affected by TPD in which latex flow is observed in patches. (**c**) A rubber tree completely affected by TPD in which no latex flow is observed.

The high-yield clone RY 7–33–97 was planted at the experimental farm of Chinese Academy of Tropical Agricultural Sciences in 1992. During the most recent 11 years, the plants were tapped for every third tapping, 1.5% ethephon was applied 2 days before tapping to stimulate latex yield. The rubber trees affected by TPD were also tapped along with healthy trees to maintain uniform conditions until sample collection. In this study, the trees with normal latex flow were considered as “healthy” trees (Fig. 6a), whereas trees with partial flow (Fig. 6b) or lacking flow (Fig. 6c) were referred to as TPD trees. Among the TPD trees, the trees showing partial or no latex flow were defined as the initial stage or advanced stages of TPD, respectively. The bark samples were separately collected and equivalently pooled from five TPD trees and 5 healthy trees. The bark was then washed with diethyl pyrocarbonate-treated water to remove the latex and frozen in liquid nitrogen for RNA extraction. The healthy and TPD barks were collected with three biological replicates. Two and three replicates were used for RNA-seq and real-time RT-PCR, respectively. For RNA-seq, total RNA was extracted from the bark tissues according to the method reported by Venkatachalam *et al.*^39^. RNA quality was assessed with a 2100 Bioanalyzer (Agilent Technologies). Oligo(dT)-conjugated beads (Qiagen GmbH, Hilden, Germany) were used to isolate poly(A) mRNA from total RNA.

### RNA-seq Library Construction, Sequencing and Transcriptome Assembly

RNA-seq library construction and Illumina sequencing were performed at the Beijing Genomics Institute-Shenzhen, China, according to the manufacturer’s instructions (Illumina, San Diego, CA). Paired-end sequencing was performed on an Illumina HiSeq 2000 platform, and RNA-seq was performed with two independent biological replicates. The *de novo* transcriptome assembly was performed according to Li *et al.*^23^.

### Gene Annotation and Analysis

All unigenes were searched against protein databases such as NCBI nr (http://www.ncbi.nlm.nih.gov/), Swiss-Prot (http://www.expasy.ch/sprot/), COG (http://www.ncbi.nlm.nih.gov/cog/), and KEGG pathway (http://www.genome.jp/kegg/) using the BLASTX program (2.2.26+) with an e-value < 1E-5, and the best aligning results were selected to annotate the unigenes. If the alignment results differed among the databases, the results from the NCBI nr database were preferentially selected, followed by Swiss-Prot, KEGG, and COG (NCBI nr, COG, and Swiss-Prot were downloaded in January 2014).

Blast2GO (version 2.3.5) was subsequently used to facilitate GO annotation according to the molecular function, biological process and cellular component ontologies^40^. The unigene sequences were also aligned to the COG database to predict and classify possible functions. Pathways were assigned according to the KEGG pathway database^41^.

### Identification of DEGs between TPD and healthy rubber trees

The expression level of each gene was calculated via the RPKM method (reads per kb per million reads) using RSEM software (v1.1.17)^42^,^43^. Using the assembled unigenes as a reference transcriptome, the clean raw reads from TPD and healthy rubber trees were separately realigned to the assembled transcriptome. DEGseq 1.24.0 was used to identify the DEGs using a random sampling model based on the read count for each gene from the bark of healthy and TPD trees^44^ and with a q-value ≤ 0.001 and the absolute value of log_2_ ratio ≥ 2 as thresholds. With a q-value ≤ 0.05 as a threshold, GO enrichment analysis was carried out using Blast2GO (version 2.3.5). The calculated p-values were subjected to Bonferroni correction, taking a corrected q-value ≤ 0.05 as a threshold. KEGG pathways of the DEGs were analyzed using Cytoscape software (version 2.6.2) (http://www.cytoscape.org/) with ClueGO plugin (http://www.ici.upmc.fr/cluego)^45^. For our study, the KEGG pathways with a q-value ≤ 0.05 were considered as being enriched.

### Real-time RT-PCR

In each real-time RT-PCR sample, gene-specific primers were designed and used, and the internal reference, the 18S rRNA gene (GenBank acc. No.: AB268099), was amplified along with the target genes. The primers for real-time RT-PCR are shown in Supplementary Table 2. Real-time RT-PCR was performed with the fluorescent dye SYBR-Green (Takara, China) and the LightCycler 2.0 system (Roche Diag-nostics, Germany). PCR was carried out as follows: 9 °C for 30 s for denaturation, followed by 45 cycles of 94 °C for 5 s, 58 °C for 20 s, and 72 °C for 20 s. Relative transcript abundance was calculated according to the instructions for LightCycler Relative Quantification Software 4.05. Standard curves were generated in triplicate, thereby allowing the calculation of primer efficiencies. The specificity of each primer pair was verified by generating a melting curve at the end of each run and by sequencing the PCR products; no-template controls were used for each sample and gene. All real-time RT-PCR experiments were reproduced with three biological replicates, and the values are presented as the mean ± S.D.

## Additional Information

**Accession codes:** The assembled sequences and sequencing reads were submitted to the NCBI TSA project, and the accession number was SAMN03704954.

**How to cite this article**: Li, D. *et al.* Transcriptome analyses reveal molecular mechanism underlying tapping panel dryness of rubber tree (*Hevea brasiliensis*). *Sci. Rep.* **6**, 23540; doi: 10.1038/srep23540 (2016).

## Supplementary Material

Supplementary Information

Supplementary Dataset 1

Supplementary Dataset 2

Supplementary Dataset 3

## Figures and Tables

**Table 1:** Statistics for the assembled transcriptome of rubber tree bark.

Items	Number
Number of high-quality clean reads	108,167,188
Length of high-quality clean read (bp)	90
Number of unigenes	57,760
N50 length (bp)	331
Average length (bp)	459
Total nucleotides length (kb)	26,485
Number of unigenes (≤500 bp)	42,101
Number of unigenes (>500 bp)	15,659
Number of unigenes (no gaps)	43,098
Number of unigenes (containing gaps)	7027
Number of unigenes (containing a coding region)	46,485
