# Supplementary material for: Transcriptome analyses reveal molecular mechanism underlying tapping panel dryness of rubber tree (Hevea brasiliensis)
Source: Sci Rep. 2016 Mar 23;6:23540. doi: 10.1038/srep23540 (PMC4804210; doi:10.1038/srep23540)
Supplement: Supplementary Information [file srep23540-s1.pdf]

Supplementary Information

Transcriptome analyses reveal molecular mechanism underlying tapping panel dryness of rubber tree (*Hevea brasiliensis*)

Dejun Li[1,+,*], Xuncheng Wang[2,3,+], Zhi Deng[1], Hui Liu[1], Hong Yang[1] and Guangming He[3,*]

[1]Key Laboratory of Biology and Genetic Resources of Rubber Tree, Ministry of Agriculture, Rubber Research Institute, Chinese Academy of Tropical Agricultural Sciences, Baodao Xincun, Danzhou, 571737 China

[2]Tsinghua-Peking Center for Life Sciences, Center for Plant Biology, School of Life Sciences, Tsinghua University, Tsinghua Park No. 1, Haidian District, Beijing 100084, China

[3]State Key Laboratory of Protein and Plant Gene Research, Peking-Tsinghua Center for Life Sciences, School of Advanced Agriculture Sciences and School of Life Sciences, Peking University, No. 5 Yiheyuan Road, Haidian District, Beijing 100871, China

[+]These authors contributed equally to this work.

*Corresponding authors; Email: heguangming@pku.edu.cn, djli.rricatas@gmail.com; Tel.: 86-898-23301174; Fax: 86-898-23300315;

Supplementary table 1 The KEGG pathways of the DEGs between healthy and TPD rubber tree bark

Pathway ID	Pathway	The DEGs with pathway annotation	All genes with pathway annotation	Pvalue	Qvalue
ko04626	Plant-pathogen interaction	218(0.204887218045113)	1408(0.157688430955314)	5.12E-06	0.000609568
ko00902	Monoterpenoid biosynthesis	6(0.0056390977443609)	13(0.00145593011535446)	2.94E-05	0.001747464
ko00232	Caffeine metabolism	7(0.00657894736842105)	16(0.00179191398812857)	0.00021104	0.008371289
ko02010	ABC transporters	23(0.0216165413533835)	106(0.0118714301713518)	0.0012931	0.038469844
ko03410	Base excision repair	24(0.0225563909774436)	114(0.0127673871654161)	0.00167242	0.039803644
ko00531	Glycosaminoglycan degradation	9(0.00845864661654135)	32(0.00358382797625714)	0.00302897	0.056944802
ko00906	Carotenoid biosynthesis	15(0.01409774436090225)	77(0.00862358606786874)	0.00346375	0.056944802
ko00562	Inositol phosphate metabolism	27(0.0253759398496241)	140(0.015679247396125)	0.00382822	0.056944802
ko03440	Homologous recombination	13(0.012218045112782)	57(0.00638369358270803)	0.00591981	0.068937922
ko03430	Mismatch repair	14(0.0131578947368421)	63(0.00705566132825624)	0.00608998	0.068937922
ko00604	Glycosphingolipid biosynthesis - ganglio series	6(0.0056390977443609)	20(0.00223989248516071)	0.00637241	0.068937922
ko03018	RNA degradation	44(0.0413533834586466)	268(0.0300145593011535)	0.01035533	0.102690356
ko04070	Phosphatidylinositol signaling system	29(0.0272556390977444)	166(0.0185911076268339)	0.01248402	0.114276798
ko04144	Endocytosis	53(0.049812030075188)	341(0.0381901668719901)	0.01671505	0.142077925
ko03030	DNA replication	17(0.0159774436090226)	92(0.0103035054317393)	0.02239763	0.17120964
ko00195	Photosynthesis	10(0.0093984962406015)	48(0.00537574196438571)	0.02310682	0.17120964
ko00511	Other glycan degradation	13(0.012218045112782)	67(0.00750363982528839)	0.02445852	0.17120964
ko00053	Ascorbate and aldarate metabolism	13(0.012218045112782)	68(0.00761563444954642)	0.0275435	0.182093139
ko00052	Galactose metabolism	19(0.0178571428571429)	110(0.0123194086683839)	0.03458403	0.216605241
ko00600	Sphingolipid metabolism	11(0.0103383458646617)	58(0.00649568820696607)	0.03819515	0.226338793
ko00270	Cysteine and methionine metabolism	37(0.0347744360902256)	240(0.0268787098219285)	0.03994214	0.226338793
ko04712	Circadian rhythm - plant	32(0.0300751879699248)	207(0.0231828872214134)	0.04864557	0.26312831
ko00410	beta-Alanine metabolism	9(0.00845864661654135)	48(0.00537574196438571)	0.05335619	0.276060287
ko00910	Nitrogen metabolism	16(0.0150375939849624)	96(0.0107514839287714)	0.06010755	0.298033269
ko03020	RNA polymerase	18(0.0169172932330827)	112(0.0125433979169)	0.07010266	0.32356933
ko00904	Diterpenoid biosynthesis	8(0.0075187969924812)	57(0.00638369358270803)	0.07069582	0.32356933
ko00240	Pyrimidine metabolism	36(0.0338345864661654)	246(0.0275506775674768)	0.07892643	0.347458538
ko00660	C5-Branched dibasic acid metabolism	2(0.0018796992481203)	9(0.00100795161832232)	0.08175495	0.347458538
ko00310	Lysine degradation	10(0.0093984962406015)	60(0.00671967745548214)	0.09499805	0.389819584
ko00750	Vitamin B6 metabolism	2(0.0018796992481203)	10(0.00111994624258036)	0.1068042	0.412914381

ko03420	Nucleotide excision repair	22(0.0206766917293233)	148(0.0165752043901893)	0.1092113	0.412914381
ko00785	Lipoic acid metabolism	1(0.00093984962406015)	5(0.000559973121290178)	0.1110358	0.412914381
ko00230	Purine metabolism	41(0.0385338345864662)	293(0.0328144249076044)	0.1152464	0.415585503
ko00950	Isoquinoline alkaloid biosynthesis	6(0.0056390977443609)	37(0.00414380109754732)	0.144065	0.5042275
ko03450	Non-homologous end-joining	4(0.0037593984962406)	24(0.00268787098219285)	0.1494045	0.5079753
ko00905	Brassinosteroid biosynthesis	2(0.0018796992481203)	12(0.00134393549109643)	0.1640966	0.531018524
ko00908	Zeatin biosynthesis	12(0.0112781954887218)	96(0.0107514839287714)	0.1651066	0.531018524
ko00563	Glycosylphosphatidylinositol-anchor biosynthesis	4(0.0037593984962406)	26(0.00291186023070893)	0.1907585	0.591222818
ko00640	Propanoate metabolism	10(0.0093984962406015)	69(0.00772762907380446)	0.1937621	0.591222818
ko00062	Fatty acid elongation in mitochondria	1(0.00093984962406015)	7(0.000783962369806249)	0.1989569	0.591896778
ko00072	Synthesis and degradation of ketone bodies	1(0.00093984962406015)	8(0.000895956994064285)	0.2453952	0.6952864
ko00740	Riboflavin metabolism	1(0.00093984962406015)	8(0.000895956994064285)	0.2453952	0.6952864
ko00945	Stilbenoid, diarylheptanoid and gingerol biosynthesis	27(0.0253759398496241)	207(0.0231828872214134)	0.2639202	0.703526678
ko00280	Valine, leucine and isoleucine degradation	8(0.0075187969924812)	59(0.0066076828312241)	0.2659185	0.703526678
ko00960	Tropane, piperidine and pyridine alkaloid biosynthesis	6(0.0056390977443609)	44(0.00492776346735357)	0.2660395	0.703526678
ko00790	Folate biosynthesis	4(0.0037593984962406)	30(0.00335983872774107)	0.2829834	0.732065752
ko00650	Butanoate metabolism	8(0.0075187969924812)	61(0.00683167207974017)	0.299524	0.758369277
ko04140	Regulation of autophagy	10(0.0093984962406015)	78(0.00873558069212678)	0.3231985	0.779962103
ko00500	Starch and sucrose metabolism	44(0.0413533834586466)	352(0.0394221077388285)	0.3282479	0.779962103
ko00380	Tryptophan metabolism	12(0.0112781954887218)	95(0.0106394893045134)	0.3411673	0.779962103
ko00591	Linoleic acid metabolism	9(0.00845864661654135)	72(0.00806361294657857)	0.3533204	0.779962103
ko00565	Ether lipid metabolism	10(0.0093984962406015)	80(0.00895956994064285)	0.3544433	0.779962103
ko00970	Aminoacyl-tRNA biosynthesis	12(0.0112781954887218)	97(0.0108634785530295)	0.3699014	0.779962103
ko00071	Fatty acid metabolism	11(0.0103383458646617)	89(0.00996752155896517)	0.3701916	0.779962103
ko00360	Phenylalanine metabolism	17(0.0159774436090226)	138(0.0154552581476089)	0.378506	0.779962103
ko04120	Ubiquitin mediated proteolysis	52(0.0488721804511278)	425(0.0475977153096651)	0.381761	0.779962103
ko00780	Biotin metabolism	1(0.00093984962406015)	11(0.00123194086683839)	0.3838404	0.779962103
ko00603	Glycosphingolipid biosynthesis - globo series	1(0.00093984962406015)	11(0.00123194086683839)	0.3838404	0.779962103
ko00350	Tyrosine metabolism	9(0.00845864661654135)	74(0.00828760219509463)	0.3867039	0.779962103
ko00860	Porphyrin and chlorophyll metabolism	7(0.00657894736842105)	67(0.00750363982528839)	0.4047899	0.802833302
ko00340	Histidine metabolism	5(0.00469924812030075)	44(0.00492776346735357)	0.4292308	0.837351889
ko00590	Arachidonic acid metabolism	2(0.0018796992481203)	21(0.00235188710941875)	0.4650482	0.879978597
ko00402	Benzoxazinoid biosynthesis	6(0.0056390977443609)	54(0.00604770970993392)	0.4676405	0.879978597
ko00630	Glyoxylate and dicarboxylate metabolism	5(0.00469924812030075)	46(0.00515175271586964)	0.4732658	0.879978597

ko00903	Limonene and pinene degradation	23(0.0216165413533835)	200(0.0223989248516071)	0.5185549	0.949354355
ko00300	Lysine biosynthesis	1(0.00093984962406015)	15(0.00167991936387053)	0.5485605	0.989071205
ko00592	alpha-Linolenic acid metabolism	12(0.0112781954887218)	114(0.0127673871654161)	0.6111046	0.9990973
ko00460	Cyanoamino acid metabolism	9(0.00845864661654135)	88(0.00985552693470713)	0.6133489	0.9990973
ko00196	Photosynthesis - antenna proteins	3(0.00281954887218045)	35(0.00391981184903125)	0.6134971	0.9990973
ko00730	Thiamine metabolism	2(0.0018796992481203)	26(0.00291186023070893)	0.6139203	0.9990973
ko00760	Nicotinate and nicotinamide metabolism	3(0.00281954887218045)	36(0.00403180647328928)	0.6362973	0.9990973
ko01100	Metabolic pathways	352(0.330827067669173)	3015(0.337663792137977)	0.6792704	0.9990973
ko00564	Glycerophospholipid metabolism	18(0.0169172932330827)	176(0.0197110538694143)	0.7127912	0.9990973
ko03022	Basal transcription factors	14(0.0131578947368421)	142(0.0159032366446411)	0.7298454	0.9990973
ko00670	One carbon pool by folate	2(0.0018796992481203)	31(0.0034718333519991)	0.7321857	0.9990973
ko04650	Natural killer cell mediated cytotoxicity	8(0.0075187969924812)	90(0.0100795161832232)	0.7604598	0.9990973
ko00480	Glutathione metabolism	9(0.00845864661654135)	100(0.0111994624258036)	0.7681707	0.9990973
ko00770	Pantothenate and CoA biosynthesis	3(0.00281954887218045)	43(0.00481576884309553)	0.7704707	0.9990973
ko04146	Peroxisome	21(0.0197368421052632)	211(0.0236308657184455)	0.78037	0.9990973
ko00051	Fructose and mannose metabolism	8(0.0075187969924812)	92(0.0103035054317393)	0.782882	0.9990973
ko00966	Glucosinolate biosynthesis	3(0.00281954887218045)	44(0.00492776346735357)	0.7860388	0.9990973
ko00620	Pyruvate metabolism	12(0.0112781954887218)	132(0.0147832904020607)	0.8069577	0.9990973
ko00130	Ubiquinone and other terpenoid-quinone biosynthesis	1(0.00093984962406015)	57(0.00638369358270803)	0.8254418	0.9990973
ko00260	Glycine, serine and threonine metabolism	6(0.0056390977443609)	77(0.00862358606786874)	0.8269698	0.9990973
ko01110	Biosynthesis of secondary metabolites	163(0.153195488721805)	1462(0.163736140665248)	0.8277083	0.9990973
ko00942	Anthocyanin biosynthesis	1(0.00093984962406015)	27(0.00302385485496696)	0.849095	0.9990973
ko00900	Terpenoid backbone biosynthesis	5(0.00469924812030075)	70(0.00783962369806249)	0.855763	0.9990973
ko00030	Pentose phosphate pathway	5(0.00469924812030075)	71(0.00795161832232053)	0.8648379	0.9990973
ko00040	Pentose and glucuronate interconversions	5(0.00469924812030075)	71(0.00795161832232053)	0.8648379	0.9990973
ko00250	Alanine, aspartate and glutamate metabolism	6(0.0056390977443609)	82(0.00918355918915892)	0.8720422	0.9990973
ko01040	Biosynthesis of unsaturated fatty acids	5(0.00469924812030075)	72(0.00806361294657857)	0.8734343	0.9990973
ko00940	Phenylpropanoid biosynthesis	27(0.0253759398496241)	283(0.0316944786650241)	0.8789119	0.9990973
ko00290	Valine, leucine and isoleucine biosynthesis	3(0.00281954887218045)	53(0.00593571508567589)	0.8909653	0.9990973
ko00450	Selenoamino acid metabolism	3(0.00281954887218045)	53(0.00593571508567589)	0.8909653	0.9990973
ko00920	Sulfur metabolism	2(0.0018796992481203)	42(0.0047037742188375)	0.8917584	0.9990973
ko00941	Flavonoid biosynthesis	16(0.0150375939849624)	183(0.0204950162392205)	0.8926232	0.9990973
ko00061	Fatty acid biosynthesis	4(0.0037593984962406)	64(0.00716765595251428)	0.8930343	0.9990973
ko04145	Phagosome	19(0.0178571428571429)	212(0.0237428603427036)	0.8948832	0.9990973

ko04130	SNARE interactions in vesicular transport	6(0.0056390977443609)	86(0.00963153768619106)	0.9007033	0.9990973
ko00400	Phenylalanine, tyrosine and tryptophan biosynthesis	5(0.00469924812030075)	77(0.00862358606786874)	0.9098257	0.9990973
ko00010	Glycolysis / Gluconeogenesis	12(0.0112781954887218)	152(0.0170231828872214)	0.9272946	0.9990973
ko00020	Citrate cycle (TCA cycle)	4(0.0037593984962406)	72(0.00806361294657857)	0.941467	0.9990973
ko00190	Oxidative phosphorylation	16(0.0150375939849624)	205(0.0229588979728973)	0.9635156	0.9990973
ko00944	Flavone and flavonol biosynthesis	3(0.00281954887218045)	67(0.00750363982528839)	0.9661002	0.9990973
ko00330	Arginine and proline metabolism	7(0.00657894736842105)	113(0.012655392541158)	0.9671481	0.9990973
ko00100	Steroid biosynthesis	1(0.00093984962406015)	42(0.0047037742188375)	0.9678801	0.9990973
ko03040	Spliceosome	93(0.087406015037594)	947(0.10605890917236)	0.981682	0.9990973
ko00710	Carbon fixation in photosynthetic organisms	6(0.0056390977443609)	110(0.0123194086683839)	0.9820616	0.9990973
ko00561	Glycerolipid metabolism	5(0.00469924812030075)	99(0.0110874678015455)	0.9829499	0.9990973
ko00520	Amino sugar and nucleotide sugar metabolism	11(0.0103383458646617)	170(0.0190390861238661)	0.9868055	0.9990973
ko04141	Protein processing in endoplasmic reticulum	36(0.0338345864661654)	424(0.0474857206854071)	0.987089	0.9990973
ko03010	Ribosome	20(0.018796992481203)	279(0.0312465001679919)	0.9941975	0.9990973
ko00510	N-Glycan biosynthesis	7(0.00657894736842105)	140(0.015679247396125)	0.9957573	0.9990973
ko03050	Proteasome	7(0.00657894736842105)	141(0.015791242020383)	0.9960871	0.9990973
ko03060	Protein export	3(0.00281954887218045)	105(0.0117594355470937)	0.9990973	0.9990973

Supplementary table 2 The primers for real-time RT-PCR used in this study

No	Gene ID*	Forward primer	Reverse primer
1	Unigene44400	AGCCAAAGGCACCTCAAGAG	GAGTTCAAGCACGCTTCTCG
2	Unigene33065	TGGCCTAGCAACACGTATCTG	TCCTGGTGCTGTTGTTGATG
3	Unigene23771	CCCTTCTTGCTCTTCTTCTCG	GCACAACCCTTCAAGCTAGT
4	Unigene25831	CTCGGTCTACCGGTTCTCCT	CCCTCGGATGTAGAACAAGG
5	Unigene47504	GGGTAGACTTGGATGCCATC	GGCTATGATGGTGGGACAAC
6	Unigene30029	CCTCCATATGGACAGCCTTC	AACCTTACACCTGGCTGTGC
7	Unigene32295	GAGCAATGCAGGCTATCTGG	GACTGGAGCTTGCTTAGGGT
8	Unigene29886	CAGCCTCGAACTCTGATTCC	GCCACCTGAATTTGTGGTCT
9	Unigene38841	CCATGTGCTTGAGATTGCAAC	CCTAAACGAAGGCAGATTGC
10	Unigene46408	GAAGTTTGGTGACCCCATGT	TCTTCCAAGTCCTTTGCACT
11	Unigene44230	CAACAACAACGGCAGCAGAC	ATCACCTGCGCAACTAGCAC
12	Unigene20474	ACCATAGTTCTGGGACGACA	ACTTCTAGCTCAGGCGATGA
13	Unigene47034	CAATATCCAGTGCAAGGAGA	CAAGTACTTCACCATGCGGT
14	Unigene21675	GGTACTTAGTGTTGCGCGGA	CCTCATTCAACGACTCCAAG
15	Unigene20545	AGCATCACCAAGCAGTTGAG	AGATGAGTTGGTGGCAGTGT
16	Unigene23733	GACGGTAGTTACAGTTGGCT	TGCCGACACAACAGAGTACT
17	Unigene46380	GTTCTGTGCCATCTTGAGGT	TGGGTACTATTTCGATCCCA
18	Unigene43867	GGGCACAGGATACAATCACA	CACTGAGACGTTGATCGTTC
19	Unigene33121	ATGGAAGGGTCATGTGTGGT	CATACAGGGCTGCAATGATG
20	Unigene62766	CTGCCACTAGTTCCTCAAGTG	GAGGACACTCTATATCCCTG
21	Unigene16838	CTCGAGGAGCTGCAATAAGAC	CAGGGAACGGGTATAGTTGA
22	Unigene5133	TGGCAAAGAAAGGGAAAATG	GATCCCAAACAGCTCATCGT
23	Unigene9119	CCACAGATTTCAAGCCAACA	AGGGACCTCACCATTGTCTG
24	Unigene48956	ATATTCGGAGGGGCAAGAGT	AGCTCTGGCATTCACAAAAC
25	Unigene4399	GCAATTGGTGCAGATTGTGG	CGAGCTGCTCTTGCACTGG
26	Unigene65458	CGGGATATGTGTTGCCTTCT	TCTTCTCTGCCACGGCTAGT
27	Unigene50342	ACAAAAGCTCAGACCCTGGA	GGAGGAGAGGCTGACATCTG
28	Unigene65454	TTCCTCAGTCACTACGCCCA	TAGACGACATGATCCCGACA
29	Unigene49603	GTTCGCACACAGGTGATGTC	TGCTAACATGCGAGAACCAG
30	Unigene41207	GCACCTAGCTTGCCCAGTTG	AAGAGAAGCACCACCACCTC
31	AB268099	GCTCGAAGACGATCAGATACC	TTCAGCCTTGCGACCATAC

* The gene IDs, unigene32295, unigene38841, unigene62766, unigene16838, unigene4399 and AB268099 are *HbDXS*, *HbCMK*, *HbSRPP*, *HbGPPS*, *HbREF* and *18S rRNA*, respectively.
