# Supplementary material for: Transcriptome analyses reveal molecular mechanism underlying tapping panel dryness of rubber tree (Hevea brasiliensis)
Source: Sci Rep. 2016 Mar 23;6:23540. doi: 10.1038/srep23540 (PMC4804210; doi:10.1038/srep23540)
Supplement: Supplementary Dataset 1 [file srep23540-s2.doc]

>Unigene8_All

GAGGATGTTGCTTCACCTGCGGAGCTTGCAAAAGCTTACATGGGTAGCAGGCCTTCAAAAATATCCCCATCAGTGCTGGGTTTGTGCAGTCGACCGACTGCGGAGCATTCAATTGCGCAGATTGATCGACTGTTCCCTTCAAAGTCCCCGATCAGTCAGTTGTGCCAAGGTCTTCACGTCATGTTGTATCTGTTGAAAATGGTTTTGTCACGCCAAGATCTCGTGGTCGTTCTGCTATTTACAGCATGGCTCCAACANNNNNNNNNNNNNNNNNNNNNNNNNNNNNTGCTGGGACAGAAACTAATGTTCTTGGTGCGCCATCATTTTCATCTCAAGGCATCTGGGGGAACAACAGATTTTCTGGATCTAAACAAGGGGCTTTAAAGCGAAGGAGTTCAGCTCTGGATAATGATATAGGATCTGTTGGTCCTATACGCCGAATTCGTCAGAAATCTAACCTCCTGCCTTCTTCTAGCACTCTTTCTATCCGTGGAACTGGCCTTGGTTCTGATGCTGCTCAACAACCATCCTCGTCACAGAAGCTAGTTGTAGCAAGCGAAGTATCTATAGAAAATGATGATAATAGTATTCATGGTTCTAGTTCTACCTCTGTCCCCTCTAAGTCCAGTGAGATGGCTTCAAAGATATTGCAGCAACTGGATGTGTTAGTCTCATCAAGGGAGAAGTCACCTACTAAGTTGTCACCATCCATGCTACGTGGACCAGCTCTTAGAAGCCTCGAGAATGTAGATTCTTCAAAATTTCTGGAGACTGTTCAAGATAATAATAAGACAGATGTTAAACGTGATGCCTCACTACCTGATGTACGAGATTCTCTGTCTCAAGAGCAAGATAAGGTTGAGGAAAATGGTCCAAAGAAGCTCAGTGTGTCTTATGAAAAGTTAGCTTCTGCAGTAAATGGTATAGATCCCACAAATTTGGTGAAGAATAATATGTCTGATTTCAAAAATATATCTTTTCCCATGGTGAATTCTGTTGCCCAGCCCCCTTTGCAGAAGAAATGGGCTTTCCAGATGAGTGCACATGAGGATTATCTGGAGCTTGATGATGATGACCATTTTGATAGGAGTGCATCTGCTGTGTTGGCTGAAGAGAAACAGAAGCTGGATACCACTTTAGTTGAAAGTAAGACCAGTTCTGCTGAGGCCATTACATCAGAGAAGCCTGCAGCTTTTTCTCAAGCCAAACCTGAAGCAAGTTCCATATTCAACCAAAAAACTCCAGTAGCTTCTGATGGGTCTGCATTTACTGAAAAGAACAGTGGATTTTCTGTTCCATCTGCTGCACCTTTGCCCAGTGTAACTGTCCAGCACGCTGTTGTTGTGGACAAGCAGGCAACTGTAACATCTGATAAACCTTCATCACCAAATGAATTAAGTGCTGCTCCTCCTATACTTAACTTTGGGGACAAAATTGTTTCTCCCAAGGAACCAAATGGTATCCCTTCTATATCTAATTTCAGCTCTAAAACTGTGGGTGTAGTACCACTGTTCACATTTGCTTCCACTTCATCAGTTGTGAGTGAGACTGCAGGCCTAAAGTTTGGTACTTCAAACCCTAGATCAGAAAGCTCAAGCAGCTTTGCTTTTAATGCTGTTGATGCAACAAAGTCTGGTACAAAAGAGCCTGAGTCAGACAAAACTGATAATATAAACAGTTTGAAGGCTGGTGTCTTCAGTTCAAATGAAACCTTGTCTTCTTCTGTATCAACTTCCTCACCTGCAACAGGCATATTTTCATTTGGCATGGGTACCAATACTTCAAGTTTAAACGGGTCTCTTGTAGATTCTATACATTCATTTTCTTCCCCTGATCCAACTCTAGTTTCTGATAAAATTGTTGGCCAAAATTCATCTAGCAGCTATAGCAACACAGTTGTTGACAGTGGTGCCATTACCTCTACCTCTGCTATGGCTACTACAGGTCCGAACACCAACAGCAGTAGCAACGTTTCTGTGTCGGCTTCAGCACCTTCCTATGAATCTAGACCTGCTTTCAAATTTGGTTCCTCATCCACTCCCAACTCATCAATAGCAGCAACTAGTGGTGTAGAATTGACAGAAATCAGGAAGGAAACAAGCTTTGGCAATCTAATGAGTGCTCCTTTTGCCAGTACATCCTCTGTGGTTACAAGCACTGGGGGCAGTCTTTTTGGTGGTACATCCTCTGCAATGATGAGCACAGGAAGTATTGTTTTAGATGGTACATCATCTGCAGTTGCCAGCACTGGAAGTAGCACTTTATTTGCTACATCCTCTTCTGTTACAAGCACAGGCAATGGTATTTTTAGTTTTAATGCTGGATCAAATACTTCAGCCGCAGCTGCAACTAATCAATCTCAGGGTTTTAATCCTTTTGGTGCCACTAGCGCTCAGGAATCTGGATCTGTATTTGCTGCTACAACTCAGAGCATGCCCATTCAATTTAGCTCATCTGCATCATCTCCTTTTGGGTTGACTGCGAATATGACCTTTTCTTCTGGAAGTTCCTTATTTAGTTCGTCAAATAATATGAATAAACTTTTCAGTTCTGGTGCTACTTTTGGACTGACTTCTACTTCTTCAGAGACTAACACTGTTGGCTCTGCTAGTAGCTCTATGTCAACAGGGTTTGGTTCTAGCTGGCAAACACCTAAATCTCCCATTTTTACTTCTGCATCTTCCTCTACTGGGTTTGCGTATGGAGCATCCTCAGCTTCTAATGCTACTAGCACCACATCCA

>Unigene27_All

GGACGTCAGAACTGCTATACAGTGTAGGTAAAGGTCTGTTGTCAGCCAAAGCCAAAGCTCTCTCCTTCTTTTCTTTCTCCAATATTTCTCGTATGGGACAATGAGCTGCAGTAACACACAGATTCTTTAGATCGCTTCCAGAGTAACCGTCTGTCATATTTGCAACTGCTTCCAAATCAACATCAGGTGTCAACTCCTCTTTGGCCAAGATAACCCTCAAAATTTTGTCTCTGTTAGGTGCATCTGGCAAGTTGATCATTAATCTCCGAGGAAGCCTCCTAATAACAGCCTCATCAAGGTCAAAAGGCCTGTTTGTGNNNNNNNNNNNNNNNNNNNNNNNNNNTTTGTACGTAGACCATCCCAGTTCACCATGAACTCGTTCTTCATTTTACGCATTGCCTCGTGTTCTCCAGGATTTTCACGTCTTCCTAGCATGCTATCCACCTCATCAACAAAAACAACACTAGGAGCAATTTTACTAGCTAGGGAGAACACTGCCTTGACATACTTCTCTCCTTCACCAAACCACTTTGATGTAATGCTTGACATAGAAATGTTTATAAAGTTTGCACCAGCCTCAGTTGCAACCGCCTTAGCAAGCATTGTTTTGCCAGTACCAGGAGGCCCAAAAAGTAATATTCCTTTACAAGGCTTAGTCAGCTGTCCCTTGCAAAATAATTCAGGCCTCTGAAGAGGAAGCATCACCAGTTCCTTCAAGGTATCCTTCACATTTTCTAATGCTCCAATGTCATCAAAGGTCACTCCAATGTCGCTAGGAGGAATAACATCTGCCAAAAGTTTCTTCTCAAATTCATTCTCAGTGACTACATCCTTCAGTGATTTCTTCAAGCTCTTGTTCTCACTTTGAATACCCTGCAGAATGCTCAACCCATACTTGATGCTCTCAGTGGATATCACGAGTTTACCTTCTTTGACAGAAGCTTCAGAACAATGCATGAAGTGGTGACTTAAAGCCCAACCAACTATTTTCTCTACACTTTCAGTTGCAAGGGCTTGATCTTTAATACACAGAGTCTCAAGGTCAGGGCAACACAGACGAACTCGGCTGAGAACCGAACGAATGCCAAAAATGTTGGCCTGTGCTTTCAGAGTTTCAATATCACGCTCCAACTGCTGCTTCCAGTCCGAAAGTAAAGCTTCATCCTGAGGTAGCTGTATTGTCACTTTGTTCGGGAGAAGCCGTCCAAGTTGCTTCATAGTTTTAGGAGTTTCCTTGCTCCTATCATGCAGTCGACCGAAGTTATCCGGGAATGCAAGATCAAGTAAAGC

>Unigene28_All

AGGGTTTCACCTAGCCAAACAATTCCTACCATGGCTGAAGTCATAAACATGCCCGTTGATTCTCTGGACCGCCGCAGCCGCGAACGCAAGGAGAAGTCCTCTGCCGATGACCCTCAATCGTCCTCTCCGCCACCACCTCCTCCTCCACCACAGAACGCTTCTCGACGACGCGACAGGGATTCAAGAGATAGGGAGCTCGACAGGCCACCAAATCGACGCGGCGTTGATTATTATGATAGGAATCGGTCTCCCCCGCCAAGAGAGAGGGAACGAGATTACAAGAGGAGGAGCAGTTTGAGTCCTCCTCCCGTACCATACAGGGATAGGAGGCNNNNNNNNNCCGCCGTATAAGAGGTCGAGGAGGGAAGATGGAGGGTATGAAGGGAGGAGAGGGAGCCCTAGAGTTGGATTTGGACCTGGCGATAGAAGGTTTGGATATGAATATGCTGGTGGATATGAACGTGAAATGGGCGGTAGGCCTGGATATTCTGAAGACCGGCCTCATGGCCGGTACATGGGTCGAGCAGGTGGATATCAAAGTGGTCCCTCTGAGTGGGACTCTGGACGTGGTGGTTATGTTGATGCTGCGAACACAGTGAGTACTCAAAGAGAAGGCTTGATGTCGTACAAGCAATTCATTCAGGAGCTTGAAGATGATATTCTACCAGCTGAAGCTGAACGCAGATATCAAGAATACAAGTCAGAGTACATTTCTACTCAGAAACGAGTATTCTTTGAGTCTCGTAAAGATGAGGAATGGTTGAGGGACAAATATCATCCAACCAATTTGGTTGNNNNNNNNNNNNNNNNNNNNNNNNNNNNNNNNNNNNNNNNNNNNGAAGGTTGCGAAGGACTTTTTACTTGACTTGCAAAGTGGAACCTTGGACTTAGGTCCTGGTATCAATGCCCTATCTGCAAATAAATCAGGACAGATGAGTGATCCAAATTCTGATGATGAAGTAGACACTGGTAGCAAAAGAAGACGGCATGGCCGTACGCTAGCTAAAGAAACTGATCTTCTCTCAGCTGCTCCTAAGGTTCACCCAGTCAGCTCAGAACCCAGAAGAATTCAAGTTGATGTTGAACAAACTCAGGCCCTTGTGCGCAAGCTTGACTCTGAAAAAGGAATTGAAGAAAATATTTTAGGCGGATCTGACAACGACAGAACCAATAGAGAGAAATCTCATTGCAGCTCCACTGGCCCTGTTATCATCATACGAGGCTTAACCTCTGTCAAAGGCCTGGAGGGCATTGAACTATTGGATACACTTATTACTTATCTTTGGCGGGTCCATGGTTTGGATTATTATGGAATGATCGAAACAACTGAAGCTAAGGGTCTTAGGCATGTGAGAGCAGAGGGGAAAAGTGCTGATGTGACTAATAATGGGAATGAGTGGGAAAAGAAACTGGATTCACACTGGCAAGAAAGGTTGAGGAGTCAAGATCCTTTGGAATTAATGACTGCAAAGGAAAAGATAGATGCTGCTGCTGTTGAATCCTTGGATCCATATGTCCGAAAGATTAGGGATGAAAAATATGGGTGGAAGTATGGTTGTGGAGCCAAGGGTTGTACAAAGCTCTTTCATGCTGCTGAGTTTGTGCA

>Unigene36_All

GACTCAAGAATTTTCTCTCTCGTTCTTTGTCTACTTTCCCAAGAAACAAACAGAGAAAGCCCCTTACAGTTTCTGACTTTGGGACCGTGAATCTTTGGACATTTTTGAGACTGACAAGAAGATACAAAGAAAATCAAAGACCTGTAATCTCGTTGAAACCTTTGCTTTTGCTGATTTGAGATGGAGAGATCTAGATCTAAGAGGAACTACTACTACGATCATCAGGACTATGATAGCGAAACCACCATGGGCAGGACCAAACCCAGGTACAACAATCACCAGTACAACCCCAATACCCAACGTCATCGTGGCGGCAGCACCACCATCAACAACAACCGCCCATCCAAGCAGCAACAGGACCCGTCCTTGATGGTAACTACCACCTACCGCATTCTCTGTCACGATATAAAAGCCGGTGGTGTAATCGGCAAGTCTGGCAGTATAATCAAGTCCATTAGACAACACACTGGTGCGTGGATCAACGTGCACGAGCTGATCCCCGGAGATGAGGAGCGAATCATCGAGATATCCGATACTCGGCGGCGGGACCCGGAGGGTCGGATACCGGCTTTCTCGCCGGCTCAGGAGGCGTTGTTCTTGATACATGATAGGATTCTTGAAAGTGATGCTCAGTTTGCTATGAACGGCGGCGGTTATGGGAATGAAGAGGATGATTATGGGGGTAGAGGAAACAGAGTGGCTACAAGGTTGGTAGTGTCGAGAATGCATGTCGGTTGTTTGTTGGGTAAAGGAGGGAAGATAATTGAGCAAATGAGGATTGAGACAAAGACCCAGATTAGGATTTTGCCTAGAGATCATACCTTGCCCCGCTGCGTTTCCATGTCTGAGGAGATTGTTCAGGTTGTAGGTGATGTGAATGCAGTGAAAAATGCTATAGCAATTATTTCATCTCGCTTGAGGGAGAGTCAGCATCGTGATCGAAGTCAGGGAAATTTCCATGGGAGACTACACTCACCAGATCGATTTTTCCCTGATGATGATTATATTCATCACATGAATAACACAGCACGTCGTTCATCTGTCGATGGGCCTTCATTTGGATCACGAGCATCTGCTACAAATTACAGAAGCAGTAACTATTCCTCCCGACAATCCGGTTATACAGTTGATGGTGGGGTTACCATGAATGACAGTGCACAGACCTTTTACGGTGAGGACCTTGTGTTTAGAATGCTTTGCCCCACTGACAAGATTAATAAAGTTGTTGGAGAGACAGATGGCATTGTAGATTTGCTTCAAAATGAAATCGGTGTAGATGTAAAGGTTTCTGATCCTGTATCTGGGTCAGATGAGCAAATTATTATCATTTCATCTGAGGAGGGTCCTGATGATGAGCTATTTCCAGCTCAAGAGGCGTTGTTGCATATTCAAACTCGTATTGTTGATCTTGTTCCAGATAAAGACAATATAATAAAAACTAAGTTACTTGTCTCTGCCAGTGAAATTGAATGTTTTGAGGGAAGAGATGGATCATTATCTGAGATGGAGAGATTAACTGGTGCAAAAATACAGATCCTGCCAAAGGAAAAACTTCCTGCTTGTGTATCAGGGGCTGATGAGCTGGTACAGATTGAAGGGGAAATAAAAGCAGCACGAGATGCTCTTGTTGAAGTTACGTCAAGGCTAAGGAGTTACTTGTACAAGGAGTTCTTTCAGAAGGATACGCCACCGTCTTCTGCACTGAGAATGGAGTCTGCTTCTCCAAAGAACTTAACCCCAGCTCGTGACGGTCATAGTGGAGGTGAACCCCCTGCTGCAACCTATCAGAATGTGCAGTCTGCTGTAGCAGTGGCACAACAATCAAAGGATGCTGGAGGATCTGGCACTGAAAAAGTGAAGCAGAATGACAGTGAGCGCCGTGAAGAGGTTCCCACAGCAATAAATAGAATCCCCAAACCANNNNNNNNNNNNNNNNNNNNNNNNNNNNNNNNNNNNNNNNNNNNNAGCTTGTAACAAAATCAAAAAACAAGCTTTCTCAAATAAGTGAGTTATCTGGAGCCAATGTAACCCTCCTAGAAGATAGACCAGAAGTGACGGAAAAGATCATTCAAATATCAGGTACTCCAGAGCAAGCAGAGAGAGCTCAGAGCTTGCTTCAGGGCTTTATATTGAGTACACAAGAAGATGGACCTTAAGCTAGTATGCGAAGCGGTTGAGCTGTCTAGAAATGGAAAGAGCCTCTCATATTTATTTGGAATCTAATTTCCTAAACGTATAACCCTGGAGAGTGAATTGTCTCTTAATTAGGCATGGAATGTTTTGCATGTCTTGTCTGTTGTTATAAATATAAATTTTCAAAACCAGTGTTCCAATGTAAATAAAAC

>Unigene50_All

CTCCAAAGTCCAAGCTATATGCTTAAAGGTGAGGCTTTGAATCGCTCTATCATTGAATCGAATAATCCTTCTCATATCCCATCCCCTACCTTCCAATACATCATCGTTTCGTGGACATCTAGCTCTCTCTTTTTGCTTCTTTTGCCCTGTGAAGCACACGTATCCAGCTCACCATACTAATTCTCTCTGCTTCTTTTCCTCTATTTATATCTATTTCTCTCTCCCTCTCTCTCTCTCTCTCTCTCTCTGAGATTTTTGATAATAGGGACTGGAGAAATGGTGTTGTTCAAGGTTTCTCGAGTGGAGACTACGCCCTTCGATGGCCAGAAGCCGGGAACTTCAGGCCTTCGCAAGAAGGTGAAAGTTTTCATNNNNNNNNNNNNNNNNNNNNNNNNNNNNNNNNNNNNNNNNNNNNNNNNNNACTGCAGAAAAAGTTAGAGGTGCTACACTTGTTGTTTCTGGTGATGGCCGCTATTACTCAAAGCCCGCTATTCAGATTATTATTAAGATGGCAGCTGCAAATGGGGTAAGGCGTGTTTGGGTTGGTCAGAATGGATTACTTTCAACTCCTGCTGTATCAGCTGTAATCCGTGAAAGAGTTGGAATTGATGGATCAAAAGCAACGGGAGCATTTATATTGACGGCTAGTCATAATCCAGGTGGTCCACACGAGGATTTTGGTATCAAATATAACATGGAAAATGGTGGACCCGCTCCTGAGGGGATCACAGATAAGATCTATGAGAACACAAAATCAATAAAAGAGTACCTAATAGCTGATCTTCCTGATGTGGATATTTCAGCAATTGGTGTAACCAATTTTGGTGGGCCTGAGGGACAATTTGATGTTGAGGTCTTTGATTCAGCAAGCGATTTTGTGAAATTGATGAAATCCATCTTTGATTTTGAGTCTATAAGGAAGCTTCTTTCATGTCCAAAATTCACTTTCTGTTATGATGCATTGCATGGAGTTGCTGGGGCTTATGCAAAACGTATTTTTTTGGAGGAGCTCGGTGCACAAGAGAGCTCATTGTTGAACTGTGTACCCAAGGAGGACTTCGGAGGGGGCCACCCAGATCCAAATCTTACTTATGCAAAAGAGTTGGTTGCTCGCATGGGATT

>Unigene56_All

CTCTTGCTTGTCTGTGACTCTGTGGAGAGTCAAGCGGGGAAGAAACCAAAGATTTTCCCATTTAGCTTAGAAATTGGGTAAATAAAAGAGAGCAACTTCGAATTCGCCAATTTTCCAACTTCTCTTCATTTCATGGCTATTTAACTGGAATTTATCCTTCGATTCTTGATGCTTCAGGGGGATCATATTCGAGTTATTGATGAAGGCATCAACCTTTTCAGAGAAACCTGAGGGCTATGGAATATAATTTCGTGCATCATGGAATATTTGATAGGCATTGCTGAAACATTAGTTCAGATGTTTTTGGCAGTGTATAGGACGCAGATTTGTGTATGCAGAATATGAGGATTTTGTTCTTAGGTCTGCTTTTTTCCCAGTGCCTTTTTGAGGTGTAATTGGTGATAGGCTTGAATTTGGTGGGCCTACCCTGCTGAGGTCATGAAAATTATTTGTAGTAGGATTTAGGGTTTAGAAGTCCCATATTTGGCTGAGGAAATAGACACAAATATACTGGATTCACTATAGCAATGGCTAGCGTAGCTCTTAGTCTTTTGCATGAAGGGCCTTTAGATATCCATGGAAGCTTTATCAGACAGAATGGTCCACGCTTAAATGACCCAATCTTGATTTTGCTATCTGTTGCGGGGTCTATGATTCCCATGCGTATTATGGAGTCGGATTCTATTGCTTCTGTGAAGTTGAGGATTCAGGCTTCCAAAGGTTTTTTTGAGAAGAAGCAAACATTGGTTTTTGAAGGTAGGGAACTGGCACGGAACAATTCTTGTGTGAAGGACTATGGCATTGCCAATGGGAATGTGTTGTATTTGGTGCTCAGGCTTTCAGATCTCCAAGCTATAACAGTTTGGACTGTTTGTAGGAAAGAGTTTGAGTTTCAAGTTGAAAGGGGCAGAAATGTAGGTTATGTAAAGCAACAGATTGCTAGGAAGGGGAAAGGGTTTGATCTCATTGACCAGGAATTGATATTTGATGGCGAGGAGCTAGAAGACCAAAGGCTTATTAATGATATCTGCAAGAATAATGATGCTGCAATTCACTTGCTCATTCGAAAATCTGCCAAAGTAAAGGCCAAACCTGTTGAGAAAGATTTGGAATTATCTATTGAAGCATTAGATTTGACTGAGAAGGGAGATGGTGTATTAGGAAAGCATCAAAGCAGGGCATTATCTGTGGCACATAGGGTTATAGAGGGGAAACAGTTACTTAGAGGTTTCATTTTGGAGCCTCTAATTATTAATTCCAAAATTGAACTGCCTCAAATAATCAAAGAGTTGATCAATTCTACATTCAATGGGTTAGAGGAGGGCAATGAACCAATTCGATCATTTGAGGGATCTGGAGGAGCATATTTCATGCAAGATTCATCTGGTCAGAAATATGTTTCTATTTTTAAGCCAATTGATGAGGAGCCAATGGCTGTGAATAACCCTCAGGGGCTACCCTTGTCAGTGAATGGTGAAGGTTTGAAGAAAGGCACACGAGTAGGAGGGGGAGCATTGCGGGAAGTTGCAGCTTACATATTGGATCATCCAAAGAGTGGGCCACGCTTATTTTTGGATGATCAGAAGGGTTTTGCTGGTGTTCCACCCACAGTAATGATCAAGTGCTTGCACAAAGGTTTCAACCATCCAGGAGGTTATGAATGTGCTACAAAGAATATAAAGATTGGTTCATTACAGATGTTTGTGGAAAATGATGGAAGTTGTGAGGATATGGGCCCTCGTGCTTTCCCTGTGGATGAGGTACACAAAATCTCAGTCTTGGATATAAGGTTGGCAAATGCTGATAGGCATGCTGGGAATATACTGGTAAGCAAAGATGGTGAGAAAGGGCGAAATGTGCTCATTCCAATTGATCATGGTTACTGCTTGCCTGATAATTTTGAAGACTGCACTTTTGACTGGCTTTACTGGCCTCAAGCTCAGGAACCTTATTCACCTGACACTGTTGACTATATCAAAGCACTTGATGCTGAGCACGATATAGCACTTGTGAAGTTTCATGGATGGGACATGCCGCTTGAGTGTGCTCGCACCCTTCGCATTTCTACTATGCTTCTGAAGAAAGGAGTAGAGAGAGGACTTACTCCCTTTGCCATTGGAAGCATAATGTGTAGGGNNNNNNNNNNNNNNNAGCAGATTGTTCAAGAAGCCCGGGATGCTGTGCTCACGGGAAGTAGCGAAGCTGCATTTCTTGAAGCTGTATCATCAATTATGGATCGCCATCTTGATGAGCTATCCCAGTAAGCCTCCAGTAAAATTCTGTAGATATATAATATGTGTGGAATTGTCGTCATGCTCGTGTATGGGTTATAGAAGATAACATATTGTATTCGTGTTTTGATGGTATTGCTATTGGGTTCTGTGCAATCTTGATTGGTGTTTGGAACTTGTAATCCCATCAACGGACTCATCTTGTTGATGCATCTGAACTTGTCTGCCATTTCCATGTAAAAGAGGAAAGGACATTTTAAGGAGTGAGTGAAGGAAGTTGTGATGTGGATGTTCTCATGATGTTAGTTGTGGTACTTGTGTTGACAATGATACGGACGTGTCTTTTTTCATCTAAAAAAAAAGATGTTGTTGGTTTCATGTTCATGTTAGACATGATTGTGTTTTGATGTACTTTTGGAGACTAATGTTTACTGGGTTCTGCCTCCAAAAGTACATCAAAAC

>Unigene65_All

CACGTTCCAATTCCAATTCCAGAAAATCTTTTGGCGAGTTCATGAAAGATAAGATAACATTTTCATTGGGCGAGAATGATACAAATAAATTGGCCAGGAATAAGTACACTGCAATATTATTCCCAAGTCAAAGAAATGCAACTGCCAAAAATACCTTCACTATAATTAAATTGCATCTAAAACTGGCAAATACATTCCTACAATTCCAAGTCCCATTCTTCAAGAGCTTAAAGCACAACGACAACCCCAGCTAGTATGCTCACTCGGTCAATGTTGGCTCAGCTTACGCTTCTTCGCCAACCTTCTCTGCCGCTCACGCCTTTCCTCTTCCTCTATCAGGCGAAGTTGCTCCTCATCCTCCTTCTTTGCAATTTTTGCACTTCTTCTCTCTTCCCTCATGATATCATCAAAGTTTGCCTCCATCATACTATCATCATCATCATCCTCATCATCATAACGATCAGCATACCTTTTTGTATTGAACATTTGCCTGATCATGTGAAGAGCCTTAATATCATCATCATCATCTTCAACATCAGAGAACGGTCTGGCAGGCTTTTTCTTGGGACGGTTATTTTGCAATGATGTACGGGATGACATTTGCTTGTCTGGCTTATTTATCTGAGGTTTTGAAGAAACCACTGGCTTATTTGGCATAATTACACTTTTCTTTGGTTCTGGTAATGCCCTTTTCTGTTCAACTTTTTGCCTTGGAACAGATGACTGCATTTTTGGAGGGAGAGGTTTGTGTGCACCAGGAAGGATTGTTTTTGCACCTGGTGCAAAGGACTTCTTCTCCACAGTGCTAAGAGGCATCTTTGAAGGTAAGCCTTTTGGCCCTGTAGGCCGGCCAGGACCAGACCCATTGTTGCTACCAAGCTGCCTTCTCGAGTCCATTGGTGTTGAAGTAGGTTTGCTGGTAGAAGTTGACTTGTGCAACCCTGATTTAGAATGCATCTGGCCATTCATAGGAATTGGTTTCCTTTCTTCACGACCACCACCACGAATATCTCTGACACTACTGCCTGATGGCTGTTTATACTTCTGTGGCACTTGTGCAGATCGTGCCTCAGATTTTGGAGCAGTGACTGTCTGAGGCACAGACTGTCTGGGAGGAGCTGGAAGTTCTGCATCATCAGACAACAGAAAGGAGTAATCTCTTGTATCCTTAAGTTTCTGCACCTTTGTTTTTAGCTCATTTCTGACTTTGGGTAGTGGTTGATGAACTCCATTCTTTGAACCTGTGCCAGTTGAGGAAGAGGTCCTTTTGTCATGATGGGAATTTGAAACTCTGAAAGCCAAATGTGGGTTTTCTAGTAATAACTTGCTTTCTTCTTTCTTGAATTACTCTTTGGGCAATAACTGGCTGTGAAGGACCAAAGAATGACCCATAATTATCGGAAGGAAGTTTTTTCTTTATCTCGTGGGATTTGCAAACAGAAGAACCATTTTCCCTTTGCATTTTCTTCCTAATTTTCTCTTTAATTCGTGCTCTTAATTCAAGATATTCAAGTTCCTCTGCACTTGGCTTTCGCTCTTTTTCCTCCTCATATTCCGCCTCCTCCTCGCCTTCTTCCTCTTCCTCATATCCTTCCTCCTCATATTCATCATAATCTTCAAAATCATCTCTTTCATATCCCCGCATTGCAGCTTATTATCTCCACTTCAATCAATCCAGAGAGCAGAGAGAATTGCACCTTACAACTACTCAAATTTTCTCCAGATTNNNNNNNNNNNNNNNNNNNNNNNNNNNNNNNNNNNNNNNNNNNNNNCGAAAATGGCCGAAACGAACCGAAACGCGAAGAGATAGCAAAGCGATCTCTTAGATCTATA

>Unigene76_All

GCAAGCATCAGCTTCTGAAATAATTCTGGTGCTGTTGTCATATATACAACGAATATGTTTTAACTGTTGATCATAGTGGGGTTCACTATGATCAATAGTCATAATACAATCATCAAAACATTAAACATGTAGTGTTTATTCTCAAAATGTGCATATGAATGTAATCCAACAACAAATAGAGCCTAAAATCTTGTTCTCTAACAAGTAATTAGAAACAACAAAGAAGCACCTGTGAGCCACAACCTATGAATGAAATGGATAATGCTCTTACTGCTATAAATATAGCTGAACATTAAATCTACGATCTTGGATCCGAAGTGGAAAGCGCAGCATACATGTCTGATATGGCTTTGGCAAAGTATTCTTTTGCTTGGGGCCTCTTAATCTTGAATGTTGGAGTGAGCAGGCCATTCTCCAGTGTGAATGGTTCAGGCACCAAGGTTACAGCTTTTGCAAATTCAAAACCTCTCAGCTGCGCTTCCTTTCCAACTTCATCCAGGTCAGTAAGAACTGCTGCCCTTGCTCTTGGATCATTGCACAATTGTCCTAAATCATCATACTTGATCCCCTCCGATGCAGCCCAATCTCTCAACACATCTGGGTCCACTGCAACTACAGCTACTAAACAGGAGTTGAAGCTGTCACCATGTATGAAACACTGGGAAACAAATCTGCATTTGGTATATACGTTCTCAATCTTCTCAGGTGCTATATATTCACCTTGTGCCAACTTGAAAATGTTTTTCTTCCTATCAATAATCTTGAGGCGTCCTCCAGGTAACCACAACCCAATGTCTCCAGTATGCAGCCAACCATCATCATCCAATGCTTCTCTCGTTTGGACAACATCTTTGTAGTAGCCATGGAAGATGGTGGGACCCCTAACACAGATTTCTCCACGTGGATATGGCTGATCCTCAGAGGTGTAGTTCATTTCAGGAACATCTATTAGCTTTATTTCACAAGCAGAATTGGGAGACCCAACATGACCAGATGAATTGTCACTCTCATCCATGATGCTTATCACACAAGAAGTCTCAGTCATTCCATATCCCTCAACTACTGGACAGCCAAAACACACCCTTAGAAAGTCCACTATCTCAGGTGATAAAGGTGAAGCACCTGATACCAAGAAACGAACTCGTCCCCCTAACTTTGCTCTTATTTTATTAAATACCAATTTGTCCCACATTGGTGATGGATTGCGGCCACTCATTATGGCATGCATCTTTGAGTTGTAGGCAGCTCTAAATAGCCTCTCCCTTAAAACCCCAGAAGTCTTCACAGCATTTGTAATTCCATCATATATACGGTAATACAACCGAGGAACACTACAAAATATTGTTGGCCTTAGAGCAGCCAAATCATCCATCAATTTCAGATTGTCCCCTTGATAGAAGCCAATAGCAACACCATAGTATGCTGATATAATTTGGTTTGATCGTTCATAGATATGTGCCAAAGGAAGATAGGATATGTAAATATCTGAGGGATTGAATTTGATTGCGAGACTTAAACCAGCAACACTTGCAATCAAGTTTCCATGAGTCAGGACTACTCCCTTTGGTGTCCCAGTAGTACCACTGGTGTAACATATGGTTGCAACATCCTCTGTTTTAGGAGGAATAAAAGGTTGCCGGTTGCTGTGGCCCTCACTAAGCAGTTTTAGATATGATATGAGCTTCACTCCAGATATTGAAGGAAGAGCTTGTAAGTGTCCATCANNNNNNNNNNNNNNNNNNNNNNNNNNNNNNNNNNNNNNNNNNNNNNNNNNACAAAGTGTTCAAAGTCTCTGGGACACAAAAGATTGCCTGCACACCAGCATGATTCACAAC

>Unigene77_All

TAATTGCTGGTTAGGATTGTTTGTACCAGGTCCAGTGGGGCTCCATCTGATGGTGTAGATCTCCTTAGAATGTTCCCTCAAATCATGAACATATTTGTCCTGCTTCATGCTCCATATCTTAGCAGTGATATCATCAGAGCATGAAGCCAGTAATGAGCCAGTTGGATCCCACTTGACACAATTAACCTCACCCTGATGCCCAGCAAAAGTTTTAACCGGGCGGTTATCTCCAACTTTGCAAACATATATCATATTGTCTGTGGAGCTTGTGGCAAATGAAAGATTATTGCGCCAGTCAACATCAAGTATAGGACCTGAATGAAATTCAAACTGTTGTCTCCATTCCTCAGTTTTCACATCCCACACTATAGCAGTTTTGTCACAGCTTCCAGTAACAAGAAAATCCCCTTTTTTATTCCACTTCAGAGAAAATATAGGACCTTTGTGTTTGCTCAATGTAGTCTTCAGTTCACCATTTGTGTTCCAAATTCTTGCTTGCCCGTCATATGAACCTGTTGCTAGTAAGGTTCCCTCACCATTCCAATCTAATGTTGTAACATCTTTGCTCTTCTCATTTGTTCTCGTCCTTCCCTTTACATGCTTCAATACCAGCACATTTAGAGGACTATTCTGCAGAACAGATTTAGATGTCCCCTCTGCTATTGTCCAAATACGTGCTGTGGAATCTCCAGACCCAGATGCAAGAAGTGATCCTGTCGGGCTCCACGCACAGGCACAAACCTCAGATGTATGTCCCTCCAAAATCATCACATCAGAACTTGGAATCTCGCATACCTGGGATGTTGATGTAGTAGAGACTTCCATTGGTTCTGGTACTCCAGAATTTCCATTTTCCTCATGTTTGACATTATTTTTATCCGCTTGATCAGCAACCATTTCTTTATCAGTATTATCCTCTTGCTGCTTTTCTCCCTCTTTTTCCTTTTCTACCCTCTCTCTATCCTTTTCATTTTCCTTCTCCCTTTCATGCCTATCCTTCTCCCTTACTCGTGCACGTTCTCTTTCATGCTCCCTTTCAAATTCTTTATCTTTTTCTTTGTCCTGGTCTTTGTCTTTATCTTTATCTCTGTCCTTGTGCAGATTTTTTTTCTTATCCTTTATCATTTGCCGTAATTCATATACATCTTTTGTTATAAGATCCAAAGGTTGCAAGAATGAGAAATCTTCATCCACATCTGCATCACCATTACTCAAATTTGCTTCCATTTCCAANNNNNNNNNNNNNNNNNNNNNNNNNNAGAGCACCAGGAGGAACCATATTGCCATCTATGGTACATTTGTTAATACCTGCCTCATATC

>Unigene91_All

AGACCACCAACAACTAACTCTTTAAGATAAAGTTCAGGTGCAATACGCATGTAGAGCTTCATGTTCAGTTCATTGTGATGAGTAACAAATGGGCGAGCAGCGGCTCCACCAGCAATCATATTCATCATGGGTGTTTCAACCTCTAGGAAATCAAGGTTGTCAAGAAAGCTTCTAAGATATGATATGACTTTAGATCGGGCCTTGAATATCTGTCGAACCTCCGCATTCAACATCAGATCTAGATAACGTTGACGATAGCGAGTTTCCTGATCTTTTAAAGCATAAGCTTCAGGATTCCTGGTTTCTCCTGGGCCCCAGATGTCAGTTCTCTTTATGTTGGCATTCTGACCAGGTTTCTCCCTTGGCATCATATGGAGACAATGAGATAAAACAGTAAAATATCTTGGAAAAATACTCAGCTCTCCCCTTTTAGTTTTTCCTGGAAACCCAATGACACCAACAATATCACCACGCTTCACACTGGAATGGAACCTAGAAAATTCAGCTTCATCCATGCCTGACTTGCTCNNNNNNNNNNNNNNNNNNNNNNNNTACTTTAACGTCACCACCATGCAAATCATAAAAGAACAGCTTCGAAGAGGAAGATCTTTTCTTCATGATTCGGCCAGCCAACGATTCAGCGATATCCTCAATATGCTCTCCATTGCTTAAGCTTCCGTACTTTTCTATGTACTCTACGATGGACAATGAAACAGAAAATTTGTGAGGATACGGATTTCCTCCTTCAGCCTTCTGGGAAGCAAGATATCTCAGCCTATTCTCATAGTATTGCGTTGGATCCATATCATCATCATCAGCTGCTGCAGATCTCTGAAACTGGGTGCTAGCCCTCTCTGCAGCCAGTCTGGCCTTCTCCTCCTCCTTGCGGCGTCTCTCTTCCTCCCTCTGCTTATTCTTCAATTCCTTCTTGCGCGCATTCTTGCTGATTGTTTCTCCTCCGGAAGCTGAAGACTCGGTGTCCATTGATAAACTAGAGACTTGTTTTGCTGTTTCGTCGACAGAGCTTTCCATCTTTGGCTTGTTTGTCTCTTATTTGTCTCTTTTTCATATGAAAAAATCCCGTGTGAGAAAGAGAGAAGGTTGATAGATTGGGTTTTATGTGCCATGGAAGTGGTACCAAGGCTAACTAGGGTTCATAATGCACGACTTTTCCTCACCGGCTACGAC

>Unigene153_All

GTCTCCAAATCCAAATATCCGCCCCTGGCTAGTGACTGCAACAGTGTGATACTGATCTGAGAAACACGATGTGCTCTTAAACTATCAAGGATTCGAGGTCTCATCACTTTATCATATACTCCCCTATCGGGAAAACCAAGGCTACCAAACCCCATCCAACCAAAGCCATAAACATGACCACTATCAACCAGAACAAATGTTTTCCTTTTTCTTGCACAAACCTGCACAGCAACGTAGCTCTTGAGGCTGTTCAAGGGTTCTGGAAGAGTCTTATCAATCTCATCTCCATGGCCCAATGCACCACAATAACCTTTTCCCCACGTATATACAAATCCACTGGAATCAAGTGCCACGGCATGTTCATCTCCAGCTGACACACGAACCACATGAATGCCCTTTCTTCTAAAAGTCTGGATTACACGAGGCAAGAACTCATTATGCTGCTCTCCATGACCAAGACAGAAATTAGAACCAGAACCAAAGGAGTAAACTGCTCCATTATCAGTTACAGCCAAGACATAACTGGGACCAGCTGCTATCTGAATAACAGAGCCGACTCCTTCAAAAGATTCAATGATTTTGGGTGTTGGTCTATCCACTGTGTCACCATGACCAAGCTGCCCATGTGTGTTGGTTCCACAACTATAGACATGGCCTTGTCTCGTAAGGAAAACAGTAAAGTTAAGTCCAGCAGCAACCTGCTTGCAAGGAACTCCCTTCAATGCCTCAACAAGCCTAGGCCTAAATATGGGGCGGCTTGTATCTTGATGACCACAGCAAAATGATGAATTATCTCCACATGTGAAAACCTTTCCAGACTCCAAAACAAATGCAGCATGATTGTGAGAGGCTGAGNNNNNNNNNNNNNNNNNNNNNNNNNNNNNNNNNGCTACGCATTGTGTTGTTTCAGGAGCATGCCCAAGAACACCACACAAACTGGAACCACAAG

>Unigene159_All

CCCATTTTTGCCAGCCACCGAGGAGATTCCGGAATGAAAAACAGGCCAGGTATCAAGACTGTACATGGTAATATTCCCAAAACTGCAAGTATTCTCCATTGAACAAAAAGTCCAAGCAGATAAGCCAACATGATTCCGAGTGTGACAGAGAGCTGATTTACTGAACCCAAAGCCCCTCTCAAGTTTTGGGGTGCTATCTCTGCTATATATATGGGCACCGTGTATGAGATAATTCCTACACCAAAACCTTCCAATAGCCTTCCCATGTAGAGAAAAGAAGCATCTCTGGCAAAAGAAATAGCAAGCCACCCAATTATATTAGGAATAGCAGCTATCATTAAAGACCCTTTTCTTCCTATGTACTCTGCAATCTGACCGCTGGCTATAGCCCCAACCATAGCACCCACATTGGACAGCGAACCAAATAAAGAGAANNNNNNNNNNNNNNNNNNNNNNNNNNNNNNTGATCGCAGTTTGGGTTGGAGACGAATAACCGCTGGTGAAACCGAATTGAATAGGACCCAAAGCGACTATCATGACACAGGCCACAACAGAGATGGAACTATCGCGAATGACCTGAGAGGAGCCCATCATGCTGGATTGCCTAGAACCCATT

>Unigene166_All

TCGTGATCGAGCCAGTGATTTGGGCCGGAGAAAGGAGCTTAGAGAATGTGTAGAGAAATTGCAGCTTCTGAAATCGCCTATGGAGCGCCAGCGCAGATTGGAAGAAATTCCAGAAATTCATGCTGACCCAAATATGGATCCAAGTTATGAGTCTGAGGAAGATGAAGGCCAAAAGGATGGAAAGAGACAAGACTATCTGAGGCCTGGAAGTAGTAGCTTTAACAGGAGAGGGAGAGAGCCAATTCCTCCTGGAAGAGGAAGTTTTGCTTCAAATGATTCGTGGGGAACTAGGAATTACTCGAGCACGAATCGGGAGTTGAGCAGAAATTTGTCCAATAAAGGTTTCTTGAACAAGGGGGATGATAATGCCGGAGCTGGTGAGATACTAAATGAAAGCTTATGGAGTCAAGGAAGGGACAGAGAAACACAACAATCACAAAGTTGGGAGAACCCAAAATCTGCAGCAAATTTGGAAACTAAGAGTTTCCATCCTGTGCTATCATCAGAATCAGTTCCTAGTGTTAAACAGGATATCGCAGTGGCACCTTCCTCTGCAGTGGTAACACAAGCTGCAATCAAAATTAATGAAACAGACAAGATATGGCATTACCAGGATCCATCTGGAAAAATACAAGGACCATTTTCCATGGTGCAGCTGCGGAAATGGAGTAATACTGGATATTTCCCTGCTGATCTGAGAATATGGAGAACTGATGAGAAGCAGGATGACTCTTTTCTTTTGACTGATGCATTGTCTGGAAATTTCCAGAGAGACCCTCAATTGNTCCATTGGTGGACAGCGGGTTCCTGAAAAGTCACAGTCCTCATCTATCATCCTCGTATTCCACAATTGCAGAAGAGGGTTGTAAACCTCAGCCTGAAATTAGTAATTCTACTGACCGAGNNNNNNNNNNNNNNNNNNNNNNNNNNNNNNNNNNNNNNNNNNNNNNNNNNNNNNNNNNNNNNNNTCTGCCTTCCCCGACACTGGCCCAAGCTGCCACAAGTGGAACAAAGGGGAAACCATTTGAAAGTAAATGGTCACCCACCCCAGCTCAACCTGCTGGTTCTCTCTTGGGAGCTAACTCACTCCCAAGTGGTAATGGGGAGCTGCAACGCCCTGCTATAGTTATCCCTGAAATCAGCCAATTGCCACATTTTTCAACTCCATCTTCAACAAAACTGTTGAATTCTGCAAATTCTCCTCAGATGCGTTCTCAATCAACTTTGTCTGGTGAATCACCAAGGGTAC

>Unigene170_All

CTATCAAGTCCGATATTAGCAATTCAAAACCTGTAGCAGAGAAGCCAAAGAAGGCGCCAAGAAGCAGGCCGAAACTCACCCCTGAGATTCTTCTCGGCGATGATGGCCTCGGCTATGTTCTTCGCCATTTCCCTCGCCATTTTATGTATCGTGGCCGCGGACACGAGGTAAGAGATTTAGGGAATCTAATTCGCTTATATAGTGAATGGCACTCTCGCTTGCTCCCTTATTACTCCTTTGATGAATTTGTTCATAAGGTGGAACAAGTTGCGGCCACAAGGCGTGTCAAGATATGCCTCAGAGATTTAAGAGAAAGAGTTGCCAGTGGAGGGGATCCAAAAAAGTTGCATGAATCAGCGAGAACATGTTGGACCAAGCCTTGAACAAGGTCATTCAGCTCCTGATGAAGCTGTGGGATTGCATCAACAGGGAGATCAACATTTGAGGAACTATGGTGCAGATGGAGTACAAGAAGACATGCTTCATGAGAGTGATGATAGAGCTACTGAAGAACCATCTCATACTGGCATNNNNNNNNNNNNNNNCCAGCTCAAAATAATATTGTTGGCTTGGCCAATGAAGTTTTGATTACAGATGAACAAAGAGCTCGCATGGAAGCTAACAGGTTGAAGGCATTAGAAAGAGCTGCAGCTCGGGCCCGNNNNNNNNNNNNNNNNNNNNNNNNNNNNNNNNNNNNNNNNNNNNCAAGTAGCTCAAAGAGGTTCCTAAATGATTAGTAAAACTCCTAAAGTTTAATGCAAACGAC

>Unigene174_All

CTCAAGGTAAATATTTGTTTGCAGTGCTCAGCTAGAAGGATCCAGAAAGAAAGAAAGAGAAGCCATGGCTGGTTTGCAAAGATCTGCAGTGTCTTTTAGGAGGCAAGGATCTTCAGGTCTTGTTTGGGATGACAAGCTTTTGTCAGGAGAGCTAAACCAAGTAGCAAACGACCCGAAGCAAGAACACGAACAAGAACGAGAACTAGAAGAAAAGCTAGATATTCAGCAAGAAAAAGACGTTAAGCCATCATCGAGAACAATCAGCACCATAGAAAGAAGCCGATCCAACGGTGGACAACGAGCCTACCGCACCGGCAAGGTCTCTCCGGCTATAGAACCTCCTTCGCCAAGAGTCTCTGCTTGTGGGTTTTGCGGTGCTTTTGGAAAACCAGCAAAGAATCATCGGAAGNNNNNNNNNNNNNNNNNNNNNNNNNNNNNNNGATAATTTTCCACGTTTTAGTGTAGCCGGGGATGGAATATTCCGGTGAGTTAGGGGAAGAGGGAATATGATTGTTTGCACTATTGGTACTATTTACATATGCTGTTGGGGTTGTAGGGTTAAGACCTTGGAAGTTCATCTCACTAAACTGAGATGCAGGGGCCTTTTTTTTTGAACAGAACCGAG

>Unigene181_All

CTCTAACAAGCGTTTTGGGCAAGCAACTGTATATCCTTCGTTGCTTTTATCCAACAACGACTACCTTCCCATTTCCCAACAATTCCGCACCTTCAGAATCTGTCGTTCTTTCTTTGATTTCATCACTAATATGAACAAAGTCCTTTTGTTCCATTCTTCGTAACAATCGCTTTCATTCTTCTGTTATTTGATTCTTTTAACCTAAGCAAAACCAATTATCTATTTTTTGGAGAGAGAGAGATTTGATGGCTCTGTGTAAGCGAGCGGNNNNNNNNNNNNNNNNNNNNNNNNTTTATTTAACCAATTTTCGACCGATCAATTCATCTTCCACTTATAGATTTCATGACAGACAGATATCGCAGCTCGTTAGCTCTAACGGAAAACGCGCCTTTCTCGTTGATACATTGGCTCTGGTGAGAGGTTTGGAAGCGCAAGGTGTGCCTTCGAAGCAAGCAGAGGCGATAACAGCTGCTATTACTGAAGTGTTAAACGATAGCTTGGAGAATGTGGCGCATTCTTTTGTTTCCAAGGCTGAAATGCAGAAGAGCGAAATGATTCAGGAATCAAATTTGTCAAAGTTCAAGTCTGCAGTACAGAGTTCTCAGGAGCATCATTTTTCTTTATTGCAACGTGAGACTGAAAAGCTACGGAGTGACATAGAAAAAATGCGCAGTGAACTGAGGTATGAAATTGACAAGGTCACAGCTGGGCAACGTTTGGATTTAAATCTTGAAAGAGGGCGCATACGTGATGAGCTTGCTAATCAGAGTGCAGAAACCAACAACCTTACGAACAAGCTTGACAGGGAAATTCATTCATTGAGAGCACAGTTGGAAGCTGCAAAGTATGACGTGATTAAATACTGCATAGGTACCCTTGTTTCAATATCTGCTGTTGGGCTTGCCGTTGTCCGTATCTTGTTGTAGTTTGTTCATTTACCTAGGTGATTAATGGTAAAATTCTGTAAGAATGCCGATTTTATTCTTTTTCTGTGGCAACTGTTGCCTGATTGTTTGATAGCTATTCCCTGTGAAAAGAGGTAATACCCACATGATTAGAGCAGTTGTCAATTTAACTAAA

>Unigene184_All

GTTTGGGGTTTCTCAAACAGGGAAACAAAATTACAATTCTATAACCTCAATTTGAAAACTAACCTCAATGATACAAGTATAGTAATCCAGGAAAATAGATTCCTAATAACATTCCTATCACAAGCTGCTGGTCCCAACACACATCAACACAATCGTGGTAGAATCAATAAGCCTATTTGGGTCCCCCAATTAGTGCACAAAGTAGAGCGAAGAATTATTCATTGTGACAATCAATTTAAGAACTTTGATACTATAAAACTATCATACACTTAAAACAGCAATACAGTACAACAACTTAGAAGCCAAANNNNNNNNNNNNNNNNNNNNNNNNNNNNNNNNNNNNNNNNNNNNNNCCCCCCCATCCGTTAACAACTCTAGTGTACCTGTCCTTAAGCCACCTAAAACTCTTCTTGAAAGAGCCTTTAACTTTCCCTTCTACAGTGTAAACCTTATAGCTAGCGACTCTCTTCTTTCGTTGCAGCTCCGCATCATTGAAACTCCAGCTCTTGGAAACAGACCCATTTGTAGACTTGCCCTTTTTGAACCGAGCATCGTTATTGCCCATCTGGGTTTGGGTCTGGGATTGGGGAGGGTAAACGGAGGATGCATAGGAGGCACTGTAACATCTGAGATCTTGCATGTTACTGACAGCTGATGAAGAAGTGGGACACACAGCACCCTGACCATGGTAGCTCTGTATTTGCATTCTTCCATCGCCATAAGACCTGGATCTGAAATCGTCCATTGACGAAGCGTGTGCGTGTACGCTCTCTAGAGCCCAGTGTCTGTAGAACGGAGCAATTTGGG

>Unigene193_All

AACACTATGGGATGATCTAATGGCATGAACGAATTGCAACTATATAGAAGTACTAGAGGGCAAAGAATTATATTGGCAGATATGTAAAACCTGCTCCATTCTGAAGGAGCAGTTTGTGGAATTGTGAGCTTAAGAAGAAATGAGATTGGGCGCTCCCATAAGTTTGAGATCTTTCCATAAGCCCTTTCAAAGGGCCCAGAACCCTGCTTCTCATCCTCCAAATTGCTCACCACCACTGCCCCGCCTTTGCCACAATCCATTTCTCCTCCTTCCCTTTTATGAACAAGGCCAATTCCCAAATCCATCCAAAATACTACCCCAACAAAGAACATATAAAATCCAACAAATCCAACAGCCTGCCAAAAAGTTATCTCCCCGCTCAAATACACATAAAATAGAGACAAAGCCCCCAACAAATAAAACCCAACATCCCTCACNNNNNNNNNTCCACATTAAATGGTGCTGCATATATCGCCACAAATCCAACCACAAACGCGGAAACAAAAGTACCAGCAGACAGTATAGCCCCAAACCCAGTTCTGTACTGCCCAGACCGAACTGCCGCCAGAGATGCAAAAACATCGGGTGCCCCATTACCAAGAGACAAAAGGGTCACTCCACCCATGCTTGGAGAGAGGTTTAAGTGATTGGTGAGCTTGGTGGTGACAATAGAGAAATGGGATTGGGCTGTTTTGATGAGGATATAAAAATAGAGAAGAATGAACAAAGAAAGGGAAGGAATCGNNNNNNNNNNNNNNNNNNNNNNNNNNNNNNNNNGGCAAAAGTGGAGAGAAAGGTAGTCGATGAGGCCATTTGTGGGGGGACGAGAGGAACAGGAGAGAGGGAGGGTCTGGTTCTGGGTTTGTGTAAGGAGAGATCTGTGAGGGATTTCAGAAGATGTAGAGGAGTGAAGTTGTGATTTGGAGAAAAATGAAGTGGAGAGGAGGGTCAAGGAGAGTGCTGCAATGGAGTTGAGGAGGGAAGCAGGAACAGGGATAGAGAAAGAGAAGGCCATGCCATTTCACAGAAGATTTGGC

>Unigene199_All

TATTTGCTTGGATGTCGAGCCAAGAGAAGCATGTGAAGAGCTACGTAGACCTCTATTCGTCGCTCGGCTGGGATTCACTTGTTTGTCACTCCCAATTCCTCAATATGTTCTTTCCTGAGAAGGCTGAAGCTTTAGCATTTGATATCCTCAACATACTTACTGAGGAGCTAAAAATTAAGTCATGCCCTCTAGTCTTTGCATCCTTTTCTGGAGGTCCAAAGGCCTGTATGTACAAGGTTCTTCAGATTATTGAGGGAAAGTGTAAATTACAACTGAATTTGGATGACTGCCGACTGGTTAGGGACTGTATTTCGGGCTATATCTATGATTCTAGTCCAGTGGATTTTACAAGTGATCTGGGGAGACGATTTCTTGTTCACCCATCTGTTCTTAAAATCTCCCATACACCAAGAGTTTTATCATGGATGGTAAATGTCATTAGTGATGGTTTGGATNNNNNNNNNNNNNNNNNCCTCAACAGATTTGAATCATTGCGAGCTGAGTATTGGCAGACTTTGTACTCTTCTGTTAGCATGGGGGCCCCATATCTTATTTTGTGTTCAGNNNNNNNNNNNNNNNNNNATGAGCTTGCTTCATATCAAGTTATTTGCAATTTCACTCAAAGACTTCAAGAACTTGGGGG

>Unigene202_All

GTTTAAGGCCACATATGCTGCATTGATGCGGAAGATCATCAAAGAGTGAACTTATAACAGGTGGATGTAATTCTCGAATTACATCTGACTTGAACTCCAATCCTATGAGACTTTTTGTCTCTGCTGATGTGGGTTCAGACAACACAACAGAGTTTTTAGCATCAGATTTGGGCAACGACACTTCATCTGTGGTGGATGAATGAGAAACAGCAGAAGAAACTGGAAGAGAAGAAGTTGATGTGTTGCTAGAGTTAGTGATGCTTGGGTTCTGTGTCTCTGATTGAGTAGGCAACTGAGGTGGTGCGGTAGACGATGTCCCCGACTTTGATGCAGATATCAAACCCTTGGCAACTAATGAGCTTAAAAGATTTGATATTGGATTAGCTACCTTATTCACTGCATTTGAAGCTTGTAAAGATGAAGGTGGAGGACCAGGAGGCAGTGGTGGTTGCTCTTCTTTCCTCTCAGAAACATTTGAGGCAACTAATGTGACATCATGTGACAAAGGAGCTGATGCTGATGCAACCCTTGTCCCAGAAGATGTTAATTGAGGAGGGGGAGGCCCACTTGGAAGAGGAGGCTGAATGCTCGACTCTGATGGATTTTTACCAACATCCTGAAAACTTTTATTAGTAAGGCCAACAGTGGTTATATTGGAGAGGATTCCACTATTCATAACGGCAGCCAACAAACTACTTGTGTTTGATTGTCCTGAAGTTTCTGCACTAAGTGGAGATGAATGATCTGGGGTAGAACTTCCAGAAGTTGATGGAGTACCAACTTTGGGAACTAGATTTAATAGTGTCCTTTGAATCTGATCAGAGGGTTCAGGCTGTTTGGTGTCTGCTTGTCTTGGCTGCAAAATAGGATTCTGGCGACTTCGTTGAAAAGGTGGTGAAGGAGATGAAGTTGGCAAGTCTTCAGGCTGCAGTTTTTGCAAGTTACCAAGCTGAACATTTGAAGAAACTAAATTCCCTGACAATTGATGTGCCTTATAATCAGGACGAGACAGGTCTTGCTCCACTGAATTTTGCAATTGCTTATGTGGATAACGTGCTGGAAATGAAGGTGAGGGTGGATGTTGGCGCAATGGTGACTGTCCAGATGGTGATGCTGCNNNNNNNNNNNNNNNNNNNNNNNNNNNNNNNNNNNNNNNNNNNNNNNNNACAGACCCTGAGGTTCCCATATGTGGTCGAACTGCCATTCTGGGCAAAGAGGAACTTGCTTTCACAGGCAACCCGCCAAGAGTGGNNNNNNNNNNNNNNNNNNNNNNNNNNNNNNNNCCTGTATTCATAGTAGAATTCCCAGAAATTATTGGCCCATCGGTTAGATGCAACTCCTTTAGTCGCCATGGGGAGGACAAATGGCGCCCAAAAGAAACTTGTTCTTTCTGTTC

>Unigene254_All

GACTCAACCACAAAGTTAAGGTCATCAAAGCCGGATGGAAAACTCACACACCATCCACTAATATAAATAGGAAAACTCAACGTCACCGACCAAATAGCTTCTGCACAATAGTTCTTGTTCTTTTTCAAATTTTTTAGTATATAGTTTTTGACGAAGCCCACATACATTTCTCTCAAGCTTCACTGAAAAACCAAAGCACAAGAAAACCTCACATTTAAACAAGAAACTACCACCTGTCAAGCCACAATTATGTTCTCAAATTGACTGCAGCATGAGCAGGTGTTTGTTACGTATAACTAAAATATCCAGGAACATAATGCGGCCCAAAGCACTCCAGTACAAAAGCTGGAGAACAAAAAGGGCCTGATCAAGGGCCTTTATCTGCTTATTCAAATATGGTCCTTCTTATCTAAGTACTTGGACTTGGCTGCTTAAAATCCATCTCTGAGTATCTGCGAGGCTTTTGTCTTTGCCCAAACTCATCATTCACAAGCTCAGGTCTCTGAGAAGGTNNNNNNNNNNNNNNNNNNNNNNNNNNNNNNNTGTCACTCTCTTTGCTCGAAGAACCCACAGAAGATGTCTGTCCGGATGGGCGAGCATGATAAGCCCCATCAACAGCAATATAAGGTGAACAATAAGTAGCCCCTAAAGCACCCGCAGCACCAGCCAAAACTGGTAGCCATGTCATTTCAGAATTTCCTAAACCAAGTTGAGGTTGAGCAACATATCCAGGAAACGCCATGCCAACAGCAGGAACTCCAATACCACCAGGATGCTGGCCCCCAAATTGCATAACTGGCAAGAAGGCTGGAGTTGCAGGGAAGTTCTGATCACCATGACCAACACCCATATTCCCTGAGGCTCCCATA

>Unigene280_All

CTTAAGCAAACAGGAAGAGAACCAAGGGAAAAGCGAACTAAACTAAACATTTACATATCCCATCTGATTGTGATAGCAACAAAATCAGAGAATTGGCGTGGCAAAATTTCCAGGCATCAACAGGCACATCCACCTGACTGAGCCCGAGACTGTGATGCGTTTCCACTGCTGGACTTCAGATTCACATCAACCATGTCTTCTTGCTTAGTTGAAGAAAGTGTTTCCATGCCTGGTAAGGCAGCGGCAATTTTCCGGAACAATGGCTTAATATTAAAACCAGCTTTTGCACTAGTTTCTATAAACATGACATTAAGATCACGAGCCTTTGCTTCTCCTTCTTCTATAGAGACTTGCCTTTTATCAACAAGGTCAGTTTTGTTTCCAACGAGAACAATGATCACATCACTGCCTGTGCGAACCTCTTCAATCCATTTTGATGTGTTCAGGAAAGTTTGCCGACTTGCAACGTCATACACAATGACAGCAACTGAGGAATCCCTAATGTAGCTTGGTNNNNNNNNNNNNNNNNNNTCCTGTCCAGCTGTATCCCACAACTGCAAGCGAACAGTTCGATCTTCAAGGTACATGGTTTTTGATAGAAAATCAATGCCAATTGTAGCCTGGTAAGTGTTATCGAATTTATCATACATGAAGCG

>Unigene328_All

TACAAGAGCTAATTCACACTCTGCAGACTTAAAGCTTAACTACCCCTAAAACCCGACATGACTTGATTGGCATCAGAGAATTGTGTACTGACCAACTCTAGAACTCGATCTTATGGAGGAGAAAAGGGAGTACTGAATCGAAATAACAAACTTGTTTGGCAGCCCTTCAGACCTCGTTTGTACAATGACAAGCTCCTTTAGCAACAAGTGCACGATTGATACCTATTGTTGACTTATGCTGCTTAGGCCTCAAGGCTGCTGCATGCTATCCTGCCTATTTTCCTTCGACACTGACACCTGTTTCAGATGCAGATTCCGTTGAATCTGTTTCAATAGAAGATAATTTGAGAAGCCGCCCTTTTTGGGCCCGAGGTGGTAGATGCTTTGCATGCTTTCTTGATCCTACAGTCCAGGGCAAGAAAACCCCAGTACCNNNNNNNNNNNNNNNNNNNTAACACCATAGGGGGCACTGGAGCTAGCATGACCACCGGGCTACGAAGGACTCCCCATTTGGTCATCGTATCCACTGCCTCGTACCCTCCAAGAGCTCCATTNNNNNNNNNNTGGCCCTGGCATGTTGGGTTGCCACAGGGTCATGGCACCAGCCACTGGGTTAGTTGGTGGGGACTGGCCTTGACTGGCATCAGGGCGCACTTTGAAGAATGTGATGCTGACCCGTTTGTTTGGAGATGGGCACATTACATGTCTTGCCATGTCAGAACTGTTTCCCCTCATTACTAAAAGGGACCCTTCTTTCAATGAAAGCATAAGTGGACCTCTATAATTCCCATCATTGTCACTCACAAGAGTACGTCCAAAAGC

>Unigene332_All

AATAGAGGACAGCAAATCAACTATGATGTAACATTACAATGCTACGTTCACAATTTTAACAACTTTTGTGTTAAATGGGTTAGCAGCAGAGCTTTTGCCAAGGCAGGTATTTGCTAGAAATCATCAGTCCACCTTTAAATATGCGGTGTTCGTCAGATAATCAAAGATATAGGCAAGCAACCATACACAAAGGGATTTTATGTGTTCTGACGAGTTCTCCATCGCCGAAATGGCTTAACCAAGTAAACAGATAATGGAGTTTCGGGAACTGGAACAAGTTTTCCGACTGCGGCAAGAGGCCAGCTGACAAAGGCAATAGCCATGGATGCAAGCCATAATTTCCAATTAAGTCTCACAGTTGACATAAATTTCCCAGCAAATTCAATGAGGATTATCTGTAGTACAAAAGTAAATCCAACTATTCCCATAAATAATCGGTTTTTGGTGATTCCACTAAAGACATTGATTTCATCTGGCTTTCGAGCATTGAACTCATTGAATATTTGACAGAGGACAAATGCATTGAATATCATAGTATTTTTTACGTCTGTAGCATGTTCCCTTTCTTCACCATTCAAATGGAGAATGCTCCTGCCCCAGAAGTTCAAAACAAGGAGAACACCTACTTGATACAAAGCCTGTATGAGTAAGTTCCTCCACATGATATTTGTTATAAGAGGTTCCCTTCGACCAACTGGGGATCTATGCATAAGGTGGTCTGTTGGTGGCTCTGTAGCCAATGCAAGTGCTCCAAGTGTATCCATGATAAGATTAACCCATAGAAGCTGCACAGCATTCAAAGGAACATCACCAGAAGAGACAGCTGCAACTACATTGATCACAAGAGCAGCAACATTAACAGTAAGCTGGAATTGAATAAATTTCTGAATATTGGCGTAGACAGAACGGCCCCATCGTACAACCTTTACAACTGAAGCAAAATTATCATCCAAGATAATGATATCCGAGCTTTCTTTTGCAACTTCAGTCCCTTGAATGCCCATAGAAAGGCCTATATCTGCCTCACGAAGTGCAGGAGCATCATTTGTACCATCACCAGTCACAGCTACAACTTCCCCTCCCTTGCGTAGGGCTTGAACAAGCAATAACTTGTCACTGGGAGATGACCTCCCCATCACCGTTATTTTCTTGGCAATTGACTCCCTTTCCTTTTCAGAATATTCACGGAACACCCTTCCTTCAATGATATTAAGCTCAGAAGCATCCTCATGTGAACCGAGAATCCCACACTCCAAAGCTATTGCTTTTGCTGTATGAATATTGTCTCCAGTGACCATTCGTACCTTAACACCAGCTTCTGTGCATATTCTCACAGCATCTTCTACACCAGGGCGACAAGGATCCTTAATGCCAACAATAGCAAGCAAAATAAGGTCATCTTCAGGTAAAANNNNNNNNNNNNNNNNNNNNNNNNNNACTTTGTCCAATTCATATGATCGGTATGCAATTGCAACACATCGTAAGCTACTTGCAGCCATGTCATCAATAGCAGCCTTGAAAAAATCCTTAGCTTCATCAATGGGTTGCAAGCAAAAGTTTGAATATATCCTGTACATGAGGCAAGAAGTATCTCAGCTGCTCCCTTCCAATGTATATGGACCTTAGAGTCAGTCCTTTGTACAGCAACACCACCTCGCTTTTTCTCCGAGTTGAAAGGGAAAATTTGGAGAACTTTTGATTGTGATCTACATACATTAAACTCCATCCCCAACTTGACTGCCCAGGAAAGGATAGCCTTTTCAGTAGGGGATCCAGAAACCTCAACATCACCACCATCCTTGGGCACAAAAACATTGCCGGTGCTGTTTTGTGCAACACCCTCACATAACAAAGAGTTAACTTCAAAGTGCAATTGTGAGGAGTCATTAGGTGGATTCATCTTGTTTCTCCCAACATAAGCTTCAACGACAGTCATCTGATTCAAGGTCAACGTTCCAGTCTTATCACTGCAAATTGTTGTTGCCGAGCCCATAGTTTCACATGCTGAAAGCCTACGAACCAATGCTTTATCTGCCATCATCTTTCGCATTGAGTATGCCAAGGTCAGCGTAACAGCCAAAGGAAGCCCCTCTGGCACAGCAACAACAACAATTGTAACTGCAATGGTAACAATTTTAATCACTCCATCTATTGCATCACTGAACTTGGTCTTGCCTTTCACAAACTGGACTACTCCATCTGAGTTTCTTGTATTTCCAGTGAAGTACCGGCCCAAAAGGACAGCAAGAACAGATACAGCTACCGAAAGACCAACAATGCCTATAAAAGTTGCAACTCCATTCAAACGTACCTGCAAAGGAGTCTCTTCACCAGTATCTTCTGAGATACTTGCCATCAACAATCCCCATTCAGTATTGATTCCAACACCAGTTACCAGCATAGTGCCAACACCATCTGCAACTTNNNNNNNNNNNNGCCTTATGATCCTTGTGAACAATTTTGCTTTCACCCGTCATGCTAGACTCATCTATGGCAAGAGAATGACCAGTGATTAAGATTCCATCAGCTGGGACCTGATCACCTATTCTAAGAGGTACAACATCACCAACAACAATATCAAATATTGAAATTTTCACTATTCTACCACCTCTCATGACCTCTAAC

>Unigene338_All

CAAAGAAACACCCAAACAAGTGGTCCCCATTATTACCTTTTTTTTATTTTTCTACACAAGTATTATTGACAAAATCAAGTCCCATGTGCTATCCATAGACCTTGTGGTATCAAATCAAATACAAATCAAGCTGTGATGTGTAATCAAATTAAAATAATATCACCTCTAAACACATGCTCAATGATACCATAATGAATCTCCCACCCTCCATTTTGCTGATGGAACCATTTTCATCATCAGCTACCGTCTGATCTTCCGAATCATCTGTCGGCGCATCAGCTTTTGGTCCATCCGCTGTCGCTGCCGCGCTGCTTTAGGCGCCGCTGCCGGAGCCGGCTTAAACAATTCCTTAGGCAACAATACCTTATCAATCTTATAAATTATTAACGGCTCCTCATCCTTTACTGTCCCCGTTATCTTCGCCGTTATAACTTTGGTTTCCAACGTAACGTCCTCTCCGTCACTTTGCACGGTGAAATCGTACTTATTGGCGCCATCAGTAGCCAGTGTGTTGGTGATTCCGTTATTGGATTTCAACATCTGCAAAGACTGGTACACTGGAATGCCGTGATATAACAGTAACGAGACTTTTTGAGCGTTGGTTAAGTTCTTGTATTTGGGGATGAAGCCGTTGATGACGGTGTCCGTGGGACAGAACACCGTTAACCCTCCGTCAAGATTTTCCTCGAATGTCGACTTAGCTCCCGACGCTATCAACAAGTCGGAAAAGGCTTTGCAGCCTTGCTTGGACATGATTGCCGTTAAATTCAACGTCGGCGCCGCAGTCGGTGCTTCAGCTTCAGCAGAATTCAGAGCCTGACTGATTTGCAGAATAGAAATGTTATATGGAATCTCTTCCAGAGACTTCACATAAACAGCATCAAGCTTCCCGTTGTTGTCTTCTGAGCCAAACGCGACCTTTCCAGCTTTAAGATCAGTAATATTGACGTAACCTGAAGCGCCAGCAGCTACACCAGTANNNNNNNNNNNNNNNNNNNNNNCAAGGTAGTACCATTAGTAATCTGGTGGAGTTTCTTGGCACCGAAGTAGTCAACAAGAACATGTAAAGACAAAACATTCCTGAGAGTGTAGACGGATAGGTTTTTGGCCAATAAAGATGACATGGCAGCATTGTCGAGAGCGAGGACAGTGATGGTCTGGCGGCGGTTGATTTCTGCGGCGAGGTGAGTGACTGTGAGATAGTGGCTGAAGGTGGAGAATGCAGGGTGCTTGGCTAGTATGCGAGTGATGTTGTGGGCATAACAGGTGGTGGAGGCAAGGAAGAAGAGAGAGAGAGTGAGAGCGAAGGTGGTGGTTAGCAGCGGCGTTTGAGGTGCCATTGTGGCGGTGAAGGGAGGAGGGAGTAGGGAGCAGAGAGTGTAGCGATG

>Unigene351_All

TTTTTTTTTTTTTTTCAAAAAAAAAGGATAAATCTTGCATTTCTTTAGGTAAATGTTCCATTATCATGCCCATGATACACGCATCCACATTCAGAACATCTAGAACAATGAGTATCTAATCAATCTATTTTTCTTATTTTCCTTTTCCTTTCATTCTATGCATTTTACAACAGAGGAACAGCAAATTTTATCACCCCAAAATTATCAGCCAGTAGAATTAATTAGTGTATAAATGCACGCTAGAACCATCCAGCCCCAGATGAAAACTTTTGAGATCACAATGGCTGAAGCTCTCGTTCAGGAACAACACCAACACGCTCATACTTACCAAGTGCTCGTTGTCTATGTCTCCATACCATCCATATGCCAATACCTGATAAACAAACAAGCAGGGCTGACAAAAGTCCAATTGCCACTACTACGAAGTGTTTCTGCCACCATGTCAGCTTTCTTTGAGGCTCAATCTTCCATTCCAACAATTGGTAGCTCCCAAATTTCTCAGGAAACTGCAGTCGATGATCCCTTAACTGCAGAATTATGTTCATTGCAGTGGTATTAGAAATGTAATCAGCAGATTCAGCAGCAAAGATGCCCCATCTAACACGGTAATTATTTCCTTCTGAGGAGAAATTCAACTTACGAACCTGCAAATTAGCAACTTCCAACTCAAGGGCAATGAAATCCGCTATTTCAGATAAGTTGGGCTTTATGTAAGAGTTGTTGACACTAATCAACATATCAAATGTTATAATTCCAATTCGAACTTCGCCTGGAACGAAATCTGAAGGCAATCTGCTTGGAGCTTGAGCAGGAGGCATTCCTTCACTTCTGTTACCACTAGGGGAAACTACATGTGCAGGCACAGGCACACCAGAAACTTTANAGTCTCTTCCATAGTTCAGAACAATTAGTTTTCCAAAAACCGATCTTATCACTCTCCCGATCATAAGTTACAAGAGTATTCCGTACTATAATTCCTCCTAACAGAGTAGTTTGATCATTCCCATTCTGGAAAATTCCCAAGCAATATGCACCGCTAACCTTTGTATGCCGGAAAAGGTAGTTCTCTGGGGACAGGGACAACCTTTGACCATTGCTGAATACCATATTAACCTCAGGAAAAGCTTTTGGCAGTTGAGAAACATCCCTTCCAGCACCAGCAAAACAAATATCATTGTAATTTGGATCAGGGCCATGGATCTGTTTAAGGAATTGGACTTCCTTCATAATAGCATGCTTGAATGCAAGAAANNNNNNNNNNNNNNNNNNNNNNNNNNNNNNNNNNNNNNNNNNNNNNNAAAACTGTTCCATGCTTTCCATCAAAGCCTCCTGGTTTTAGCTTCAATCGCTCCCCAGCCACAAGTAGTTCCTTCAGCTTAATATTGTAATATGGACTACGGTAAGGGTCTGAATGGGTAAAAACCATATCAGGGGGTGGAGAAATCCTACCTAGAACCATTGCACCACCACCAACATCCATCCCACCATAACATAAAGAGAATGAGTAGCTTATAATATTCTTCTCAACAAGCTGATCTACAACACTGAGCCGACCACGGCCCAACCCCATGATTCCATCAGCACGTTGCCTGAAAAGATCACCAGTTTCTGCATTTTCACAGCCAAAAATAGCTCGCTTAGGCGTGAGCTCGCTTTCATTTCCAAAAGAAATGACATCCTCAGCGAGAACACCACTGCTAGAACTCATCTCAGCATACCGTCTCTCATAAGTACATTGCTTCCCCTCGTCATCACAGTCACAACTGGGGTTGCACTTTATAGGTTTGTAGGTGCTAGA

>Unigene373_All

ATCAGCTTCAAAGAAAGAACCCCAACTCCTTATTTATGTTCTTCCCCATCCTACCTCCTTCCACCCAGCAGCCACCATACGTTTCATCATTTCCATCAAGAAGAAAACTTCTCAAAATTCAAACACTTTGATCCTCAAGAAAACGTACTTCTCAATACTCTGAAGAGTTGTGAGGTTTTTGTGTTCAGAACATTTTGAGTCTCATGGCAAACTTGGTTCACAAATCCGGTAAGGCAGCATCGCCTGAGAAATCAAACTTTGCTCAGACATGCAATCTTTTGAGCCAGTATTTGAAGGAAAGAGGAAGCTTTAGAGATCTTAGCCTCGGAATTAATGGAAAGCTTGAAGCTAAAGGTCCTGAAGCATCTAGGCCACCTGCAACAACACTGAATCTTTTGTCAAATATTGAAAATTCAGCGGAGACTTCAAGAAAAAACTCTGTGCCTGCTCCAAACATAAAACCCATGGATTTCTTCCCTCAATTTGCGGGTTTNNNNNNNNNNNNNNNNNNNNNNNNNNNNTCCACTGCTAAACCTGCAGGTTTGAGGAAATCTTCCAGAGCAGTAGATCCTGGAACTGCCCAAATGACCATATTTTACGCGGGCCAGGTGATAGTTTATGATGATTTTCCAGCTGATAAGGCTAAGGAGATCATGGCTTTAGCAAGCAAAGGAATCTCTAATGCTCAAACTGGCTTTACTGCTTCTACTTCTGCCATGTATAAAGTTAATCCGACCATTGCTATTGCTTCCAACAATGCTCCAGAAGGACTTCGGTTGCATCCTCAAGTTAATGGTTCAGATATGCCAATTGTTGGCAAAGCATCACTTCATCGGTTCTTTGAGAAGAGGAAAGAAAGGGTAGCATCAAAAGCACCATATCAACTAAACAACCCATCATCACTTGCTAGGCCTAGACCCAGTAAAGAAAGCAATCACTTGATTGTTGACCTAGAAGCTCAATCATCGAAACAGCTTGAGCTTAAGTTATAGTTTGATCACTTGTTCATGATCACCATAATCTCAAGCAACGTACGATAATGTAGTGTACTTTTTAAAATCCTAACTATTATTGTTATTATTATTTTTAATATATACTAATTTTACTCGTACAGAGTTTTGTAAAGTATATAACTTAAGCTCAAGCTGTTTCGATGA

>Unigene480_All

TAAGGTTTAAGGAGACTAACGTGGAACACTGGATGGCACTCCAAATGTGCCGGCAGTCTCACTCTATACGCCACCTTCCCAATTCGCTGCTCAACCATAAACGGTCCCTCATATTTTCGAAGCAACCCCTTGTGCCGTCTACCATGGGTACGGGAAAACATTCTCACAAGTACCATGTCTCCCTCGGTGAACTCCACATGCCGCCTCCTTGAATCAGCCCACTTTTTCATTTTCTTTGTAGCTCGGGCCAAGGAAACTTTAGCAAGTTCCACGGTTTCCTTCCACCCCTTGGCAAATTTAAAAGCACTCGGGTTAGGCCCCCGGTATGGTATCATCACAGTGTGGGGCGTGAGGGGCTGCTGCCCCGTGACTATCTCAAATGGTGTTGCCCCCGTCNNNNNNNNNNNNNNNNNNNGGTTGTAAGAAAACTGCGCCACATCTAACAATTCTGGCCAGTCCCTCTGATTAGCGGC

>Unigene519_All

AAATACTCAGCTTTCATCTCTGAAACCAGCTGGAAAAGCAAGTCATTGGAAACCCCATGGAACAAGTAAACCTTGTCCAGGAGAGAGTAAAAAAGATAATGTGAAATGCTTGAACGAATTGCTTTTGGAAGGGAATCAAGAATTTCTTGCTGCTGCAGTCCTTCGGAATCTGTCCTGAACTTCAAACACAGATGTGCAAGCATCTGATCTTGCAGGCGAAGAGGCAGTTGGTTCCTCTGAGCAAAACTTGAAGCTGCTTGTATGGCATCTCTAAATCTTCTAGTTCGACTAGTCCCATGAACAACCAAGTTGGTCATGTTTCCAATCAAATATGCTGTCAATCCAAGGTTAAAAAGCATATAGAAGATGTCAAAGATCATCTCCCTTGTATTCACANNNNNNNNNNNNNNNNNNNNNNNNNNNNNNNNNNNNNNNNNNNNNNNNNNTTGAAGTCACATATCGGATCCACAAACTCCTTTCAAGAAAATTGTCCCCTAAAGATACTCCAATCCATGTCTTATCAGGGTTATGATAACGTGCAGCAATAAGATAGTAGAAGCATCCAGCAAAGTGAACTGCAAAAAGGGTGACGCAAATAAGCTTGGCACATCGAACCCAGAAGTAGTTGTAGTTCCTATCCTTCTCCAATCTGGAAAATAGAGCACTAACTCTTCTAAGCCTCAAAAGGCGAAGCATGTTAAACAAGCCATATGAATATGATAGGAATAATTGCGGAGAGATTTTCCGAGCAAGTTCAGATGGGATTGTGGATATGAAATCGAAGGCAAACCAGGAACTCAAATACTTCCAAGCAATCTTTTTTGGATTGTCAACAAGTAGGTACGTGGTCTTGTCAAGGTAAGCTACAAAGAAGGTAAGAACTATGTCTACAGCAAAGAATCCATTGACAACATTATCAGCAATGGAGAGTGGTCCCTCTGGTTTCTTGAGAAATCCGAATTCAAAAGGCGACACCCAAGCAGTATAGATGACTAGAACAACAAGAAAAGTCTCCCATATTCTGTAGCGACGGTCATAAGGAGATATAATGAACCTCCTGAGCTTGACTCTGCGGTTACTTCTTGCACCAAGTGAAGGTAAAACCGCAGAAGAGAGGCTGTAATGGCTG

>Unigene521_All

AACTCCAAAGACAAGATTATAAATCTGAAGGATTCAAACAAAATGTATCTACATAACGATAATGATGATGATAATGTATAAAATCTCTTCCTCAATTGAAAAATCCCTATTAAAGATTATCAAAACTCCCAAAGAGGCAAAATGGCCACCATTAAATGCCTCTTCAATGTCGTGGGCATAATTTTATAGGTGTTAAAATTAACAGAAGGGAGGAAAGTTCTACAGAGACCTGATTCTTCAAATAACTAAAAGTAGCTCTAGACCAACAATCAATCGCAACGATAATAAATTAAGCCGTCTAGCTATAAGAGAGGCTCCATGCTATTCTTCTGCAATTTAAATTTGTGAAAAATGCCTCTCCCGTGTCTTTCTTCCACGTCTTTAACTCAGAAGGTAGCCAACCAACATTAATCTTATATGCTCATTCAGTTACATTCACAACTCGTCGTGAACCTCAGGTTCTGTGTTGACTTTTTCTTTGTCCAAGCCACTTGAATCCACCGTTGATTTGTCGCTTGAATCTGCTGTTGGTTTGTCACTTTCAGGATTCTCAGCTGTAGAATTTGAGCTGTTCAAATTCTCCTCATTAGTCTCAGTTTCATTCTTCTTAGGCTTCTCAACCTTCAGCTTTGGCTTGGGAATTTTGTTAACGGCAGCAACCTTATCTTGAAGATTGAACACCTTCATGTAAAGTTCTTCTGACGTGAACGCAGGTGTGCTAAATTCTGACGTCTTCTTCTGCTCAGCCTCCTTCTCATCCAGCCAACTTCTTAACTTGTCAGCATCACTTAGAACCTCATCTATTCCGCTCTTTGNNNNNNNNNNNNNNNNCCCAGCTCTGTACGATCTGTTGCAGCTCACCAAGGTACTTGAGAGCAATCTCAGTCGCTGCTGGCCTTGCAGTAAGCTCTTTATACCTGAAAAATATAGGGTCGCCAATAGCTTTTAATGAATCTAGACGATCCTGAAACTCAGTAGCAGTAGCATCTTCACCATCAGTATATAGCCATTCTTGGACCTCATCAAGCTTTTCAATGAAAGATTTACG

>Unigene522_All

ATTATGGATTAAGATGACATTGATCTCGTGATTACGACATTCTTCCCTGTATTATTCCTATCTATGTAATTTTAAACCTGGAACATTCACATTGTGTATTATAGCACCAGAGGCAAACCAAGAGCTGACAAGACGAACTTTCTCAAGTTACCAACTGCAGCAGACGTGCTGAAAACGAACAGGCTTGCACTTATCATGGTTGGATAAATTTGTCCAAGGCAGATGTATCCAGAGCAGCAAACTCCTTCACAGCAGAAGCAACAGAAAGTCCANNNNNNNNNNNNNNNNNNNNNNNNNNNATTGCTAGGTGCCTCAATGTTAAGGGGAAGAACAGGATCATGAAGCGAGAGTCTAAGAAGGAACCACCCACCATAACCAGAAGCTCGAACCCCTTCATAGTTGACAGGAGCTTTCTGAAGCTTTGGGTCTAAATCCACGGAGTTCTCCAAGTGTTTTAGCACAGCCTCTCCATATTCCCGGAAAGATCCTCCTTTAAGATCTGGATGATTCTGAATGATTTTAAGTCTCAATTCTACAGCAACTGCTGGTTCCTGTAGACCCTCTACCAGATTGGTCAACACTTTGCTGCCACCACCAACTCCTGATGCTCTGGCTGAAGCCAGTTTATTCAAAAGTTTGACCATGAGGTAAGCACCATCATCCAGCCAGTGATTTTCCTTGAGAGCTCCATGCCCGCTTGTTTCAATAGCTAGATGTGACTCCTCACCAATCGAGTTCAAACGAATAGCTTCGTCAATGACATTTTTATAGCCTCTCTTGAACCGATGGTGCTTTCCCCCAAGTTTCTTTTCAATAAATGTAGTCAGGCCATCTGAAGTCACACTGTCTGTAACAATAGTTGTTCCTGGATGTTCCTCCAGAACTATNNNNNNNNNNNNNNAAGACGATTCCGGTTGAACTCACGACCAGTAGAATCCACAGCAGCAGATCTGTCAACATCAGTATCAAATATGATCCCCAGATCAGCCTTGTTTTGAAGGACTGCTTGGGTGATAGCTTTCATTGCTACCTTGTCCTCCGGGTTGGGAATATGATTAGGAAACAAACCATCTGGTTCCAAGAACTGACTGCCATTAGTGATAGCCCCTAAAGGCTGAAGAACTTTTGCAGCAAAAAATCCTCCTGCTCCATTTCCTGCATCAACAATTATATGGAACCCCTCCAATGGCTTCTCTATATTTCCTGCAGCTTTACGGACTGCCTTCACAAGATCAGATGTGTACACTGTCATGTAATCAACTTTCTTTACTGATTCACAAGCCTGTCTTTTTGACTTCATTAAACTTTCATTTGTAAAGTTATTATATATATCTGCAGCACGTTCCAAAATGTCTTTAATGTCAGCCTTCCCAAGCCCTCCATCATTGGTGAAGAATTTGAAACCATTCCGATTGTAAGGAAGATGACTAGCTGTTATCATTATAGCCCCATCAACTGGACATAAAAATGCTTCATCTACAGTGAGTGTGCTATTAAACATTGCTGGGGTGGATGCCAATCCATACTGAATAACAACCAGGCCTGCACCAGCAATACCTTGAGAAACAGCATCCTGTAGCATTTGTGCAGATATCCGGGAATCATGACCAATGGAAAC

>Unigene523_All

GATGTATTGCCTCATACTTAACTTTTGCTAAACACATGTTTTCATTTCCTTTCCATGTGTTGAGCAAAAGTTAAGTATGAGGCAATACATCTCTAAGTACAAATGTTACAAAATATTTTTGGGAGCTCTACAAGTAAATATTGTACCACCCTTTCATAATGCAAAAAACCAAAAACACCATGCCCAACTGCTGCTTCTCCAAAAGGCAGGTCAAAGATTCCAGCTGTTTCCTCTCCCCATAATATGTACAAAAAGGTGACTCTGACACGAAGACAACTATTTGCAGCATCAATCTTAAACAACTTGTGAGGAAATATTTGCAGTCAAAAGTGTTCCACGAAACCCTTCCTCGGCATGCTGTGCAGTCATCTTTTGATCAGCAAGCTCATCAGCAATATCTCTATGCTCATGGATTTCAGAATCTTGCACCCAAAGCTTATCTCCAGGGAATATATTCAAGTCTGCAAGAGTAGCAGATTCCTGATCAAGTATCCTAGAACCTTTGTGTAGTATCTGGTNNNNNNNNNNNNNNNNNNNNNNNNNNNCATCATTTTTAGCTGGTATACTGAAGTAGAACCAGAAACCTTTAAATTAACTGAATTCCCGTAACTGGTCTTACGAGAGCGTTTGGAGGCTCGTCGATCAGGCT

>Unigene526_All

GCCACAGGCAGCGGAGCTGATCCTATCAGGGGGGTAGGAATCGAAACAAATCCCACAAGTGATAATTGCAGAATCAATTGAGAAAATTTCAGGAAGCAAAAGTGTTTTCTGAAGCAAGCCCACTTTTTCACGGACAGCGAACTCATCAGAAAACCAAGAATCATGGAGTTTAGTGAGGGACCAATTGAAGAAGCAGAGTAAGATACTTGCCTCAGGTTTTGGAAGAGAAAGCACAGAGGAGACTTGAGCAAGTTCATCCTCCATGCGNNNNNNNNNNNNNNNNNNNNNNTTGAGGATCCTGTAATTTTGATGCTTAGTAATAAGATGGTTGGAATGATTGGCATCGTGATCATGATGATGATCACAATTAGATTCCAAGAAGAGATCATCAGTGCTAAGGGCATCGTCGAAATAAGCATCATCGAAGCCATCGCTGAAGCAGTCAACGGAAGTCTCCTCCTCCTCTTCTGGAGTATTACTGTACATGGTTTGGCTTGGCTTATAGCGATCAAGTTGAAGGATGCTGAAATG

>Unigene541_All

TGAAGAAGAGATCGAATCAATCAATTTGTGAAGTGAAGAACAGTCCCAGAAGGTAGAGTGGCCTTGGCGGCCGGAGCAGGTTCAACGCTTGGAGAAGGAATTACTGGCTTTTCCAAAAGCTCGGAAGGCAAATCAGGTATCCCCTTCAAATAGANNNNNNNNNNNNNNCAAAGGAAATACTATTTGCTTCAGCTTAACCTGTCCGTACACTCCTTCAAAAATCCCAGACCCACCAGTCACTGCCAGATACGTATCCTCATACGTCAGATACGCTCCTTGCACTGATATGTGCCCATAGTCTCCGAAGTAGAAGCTGTATATAGCCTCGTATCTGTCACCTTTCTTTTCTGGTACGTGCTGGATAAGCACACAGAGACCAGCAGTTACACCTAACCTTTTCTTTAGGTCTCCAGTGTAAAGCTTGTTGGTGAAGGGCACGAGGTCACCCAGAGATGATTCTGCTCTTTTACTCAGTTTAAGAACTGCTGGGCTGTCCCTGTCTCGTTCGTTTATTTCATAGACGTTTAGTTCTTGAACTTTCACGGGCTCTCGAGATTGTGGTGATGACCGTGGTTTGTTGAAGAAGAAAGCTTTAGTGGCAATGCTCCAGGAGCTTCTTCTTGATGAAAAATCGTGATTCGATGTGGAGAGTGTCAACTTTTGGATTGAGAAGTCTTGTGAATGAGCGAAACCCCATTGTTTTTGTGGCTGAAAAGATGAAGAGTTGGATCTTAGGTTAGGGAATTTGGGAGAAGAAATTAAATTGAGAGAGCTTGTAGAGGCCATTGATGATGGATTAGGTAGCTTGAGAAATTAAGGATTGAATTAATGCAAGAATCCAGCTGCTGGATACGATTCAAACAAATTAAGGATTGGTAGACATATTTATAGGTACAAGATCAAGA

>Unigene560_All

ACCCTACCCATGAAACCTAATAGACCCAAAGGCGAGAGCCTCCACCTTGACGTGCAGCTACCACCGGAATCATCTGAAAGAGGTTGCCGGAGACCAAAAAGGGAGTTTCTTCTTCCAAATCCTTGCTAGCTTGAGCAGGGCTTCCCAAGGTCATAAAGAAATCGACAAAACCAAATAGATCCATCGCAACTGGCAAACTAGGCTCAAAACCAATCATAACAGGGGAGAGAGAATGGCATAGGCCATAGCAAAACCAACAAAATACTTCTTAACGCCAGCTATAAACCCTCGGAATTGCCTTCATTGGAACCTCTCCCGCTCTGAAATACCAGCTCATCCCACTGAATCTGCGACACCAAGAAGAAACTGACCCATGGAGACCAAGTTTCCTTTAACCAAACAAAGCCACTTGTAGGACAAAACAAGATCCCTCGCCATTAAACCCCATCCTACCAAGCCAAAACCAAGCACCAGAACTACGTACAGTTTCACCCAGAGATGAGGATAAAAAACTATACCTNTCTGGAAGGAGAGGAAAACCTTCCCAAACTATACCTTTGGAAGGAGAGGAAAAGCTTCGCCTATCTCGGAACGCCAGGGACGGCTTCGGCTAGCCAAGAGGACAAGGGCAAAAACCCTAAAAGAGATAAAACTTTGAGAGAGAGTCTCTCTCTAATCACGTTAAAGGGAGGAGTTAATATTAAATCTACACTTAGAAGAAGCCGATGTTTTCCCACACCTCAGAACCAGGAGGAATGGCCAAATGAAACTAATGGAGGAGCTAGCCATGGCTTCCNNNNNNNNNNNNNNNNNNNNNNNNNNNNNCAAGAAAATCGATATTGGACGCGTGTCAGAGGCTGGCAACTTAAGAAAAGTCAGGTTTCTC

>Unigene567_All

CGCCACAGAATTAAATTTGACAATTTCATTGAACATCAAGTATGAGTATTCATCCACTAGCATCCTAGATTATAAGCTCAAATTAAACTACGGTACAAAGGAGAAAAATTTTCAGTAAGAGATGAACGATCATCTAAATCACATGAAAATGCTTCATGTCAGTCCTTATGGCAGTTAGATTACCTCATCCCCATTCTTCCATTTCCGAGGATTGATTTGGTAATGGTTTGGTTAGTGACGTGCAACAACAACAACTAAATCTCAATCTCAATACCAAACTTGGTTAGTGACATAACGGAAAATTTCCGACCATAACGTTGTATCAGGAGGGGGTAAAAACCTTGATTTCATCTGCACAGCGAGAAGCACTGGCCAGAGCCAAAACTCTTGAAATGTTTATGTACAGAGCTCAAGAGTCATGAGGTTCTTGGACAATNNNNNNNNNNNNNNNNNNNNTTACCACTGTCTGATCGGAAGTTATCTCCTTTAAACCCAATCTCTACAGAACTGTCAGGTGGGAACCACCGTAACAGAATACCAAGATTTGCCACCGCAGGAATTAGCTTGAAAAGCAGGGGAGTCATTATCAGGCTAAGAGCTGTTGTCCCAAGAAGAAGTAGGTAAAGCTTCCCCTCAATGAGATGAAGATTAGAAGCACGGCTGAGAAGAACAAAAGCGAACTCCCCTATTTGAGCCAGCGACATTCCAACAAGCATTGCAGTCTTGTTGTTATAACCAAATCCTTTTACAACAGTAGTGATTATGGCTGTCTTTATGATTATGACTAATATAACAGATGCTAGCAATATATCAACATGGTTCCACAGAAAATGAACATGGATCAGCATCCCAAT

>Unigene575_All

GTTTGCATTGATCCAAACTTCAGCAAACAACCACCTTGCCAACACTTTTACCCGAATGAAGATACTCTACAGCATCTGCAACAGCATGAACACCAACAAATCTTTTGGGATCTATGGAAACCTTAAGCTTTCCTGTAGAGAAAAGATCAAATAGCCTGTCCAGATGCTGTTGCCATAAGTGACCATATTGAATTAGAAAAAACCCAGCCACTGTTTGGCTTTTCGCTAAAATCTTCTCACAAAGTCCAGTATAATTTGATGGCATCCATCCATGTTCTCCTTGATACTGTGAAATCATTCCGATCACAATAAGTCGTCCATGGACTGCTAAAGCATTCAGACACAGGCCAAACNNNNNNNNNNNNNNNNNNNNNNNNNNNAACACCTTTAGGAAATTCCTTCTTTAGAACAGTTTTGATATTTTCAACTTTGTAATCTATGACTCGGTCAACTCCCAATTCTTTCAAGAGCGTTGCCTTTTCCTTACCTCCACAGGTTGCAACTACTTTATTTCCTGCCAACTTTGCAAGCTGGACAGCAAATTGCCCAGTCCCTCCTGCTGCAGCAGTAACAAGAACTACTTTTCCAGATTCCATCTGTCCAGCCTTCTCTAGAGCAATTGACGCTGTTAATCCTGATGTAAGCATGGCAACAACTTCAGGATCTGGTCTTGGCACAGGAAGAATATGTTTTGCAGGAACCACTGTGAATTCAGCATAACTTCCAAAAGTCATAATTGCAGCAGGAGTGCCAACTTTCAAATCCCGAACAGATTCCCCCAAAGCTGCAATTATTCCCACTGCCTCAAAACCAGCATCAAATGGAAGACGAGAACCAAGGTCTTTGTTGTTGCCCGTAAAATACCGTCCAGAGCTGAAGTTTACATCACTAGCATTTACACCAGCATAAATGACTTTCACAAGAACTTGATGTGGTTCAATGGGTAATCGCAGTGGTGTGCTTACAATGCGGGTAGCATTGCGGAAATTGTGACTCAAAGTATGGACAACTATCTTCTCAAAGCTTTGAGGAAGTTGAAGATTTGTTGGGGCTTTGAAAGAAACCCTTTTCCTGGAAGCTGAAGAACGCACCAAGTATTTTGCTTCTTCTGCAGGAGCGGGCCAATATTCCATACCCCTGCGATGTGTTATCCACAGGCAAGAGCCAGCTTTACTCTCATCAGTAATGAGCTCAAATGCACCTTTGATTACCGTTTTCATCGGAATAAAGCCTCCCATCAAATCAACAAATTTAGCATCGATTTTTGATCCCATCTCAGTTTCAACAAACTCAGGACAAAGCACATTAATGCGAATACCTTGACGTTTGTATGGAACAAGTGATCTAGTAAACATAACAACACCACCTTTAGAGCCAGTGTAGATAGGGTCAGTATACAGAGGATAAAGACCTGAAGCAGACCCCAGATTTATAATGACTCCAGGCTTTTGTGCAGCTTTCATAGCTTTTATCGCAAGCCGAGTGCAATCAATTACTGCAACCAGGTTCACGTTAACAGCGTGCCTCCACGAATGAGTACCATCAGTTTGATCATTATAGAATGGTATAGAAGTACCTATACCAGCACTGTTGATACAGATATCCAGTCCTCCATATGTTGCTACATGATTCTCAAAAGCAGCAGCCAAATCTCTCGAGTTTGCCACATCGCATCTAATAAACAGAGCAGTTGGGAATTCCAACTTGGAATGGAATTTGGCATTCTCTTTCTCAATGAGGGATACAACTTCCTTCCCCTTTTCTTCCAAGAAATCAACAACCGTAACAAACACTCCCTTCGCTCCCAGAGCTAAACTAAGCGCCTTCCCAATCCCCGAGGCTCCACCAGTGACGAAAGCTGATAAACCTGGTTTGATCTCCATAGAAATGGCGTTTACTTCAATTCTCAGTAGTGATGTTAACGAGAGAAGATTA

>Unigene589_All

ATTCAAATGGATGGGCATCACGGTTTCAATCAGAAATGTCGGGTTTATTACAACCCTCTTCAGCACAGAACTGGCTGAGTTCTCAAGGCAGTTCATCTGGTCTTATTGTTAAGAAAGCTATCAGAGTTGATATTCCAGTCGGCAAATATCCTAATTACAACTTTGTTGGGCGCCTCCTTGGTCCTAGGGGAAACTCTCTTAAGCGAGTAGAAGCAAGTACTGAGTGCCGTGTTCTGATCAGAGGGCGTGGCAGCATTAAGGATCCAGCTAGGGAAGAAATGATGAGAGGGAAACCAGGGTACGAGCATCTAAATGAACCTCTTCATATCTTAGTTGAGGCAGAGTTACCAGTTGAAATAGTTGATACTCGCCTAATGCAAGCGCGTGAGATACTTGAAGACTTGTTGAAGCCTGTGGATGAATCTCAGGATTTCTATAAGAAGCAGCAGCTAAGAGAGCTAGCAATGTTGAATGGTACATTTCGCGAAGAAGGTTCTCCAATGTCTGGTTCAGTCTCCCCCTTCCACAGCAGTCTTGGTATGAAGAGGGCCAAGACAAGGGGGTAATGGATCCCCATTCTTATTTGGCTTTTGGAGCATGTGTGTTTGTCCTGCCAGTAATCAGCGGTTCCCATGCCACAGATGTTGCTGTGTCAGCCGTTGCCTCATGTTGGGTGCTAGTTCTAACTTGTCTCTATATGAAGTGTGTCTATCTTATGTTTGGGGGAGGTTTATCGACAGTATCACATGGNNNNNNNNNNNNNNNNNNNNNNNNNNNNCAAGACTGCCCTTGTATTAAATTAGGCTTTACTATATAAGTGTGTATTGACATTTTTGGTAGTGATGGGGGCTTATGTGAGAAAGATATGTTTTGTATACAAAAAGCAAGCTAAGAATTGATCTATATCCTAATGCATGTTGTTTGGGATGGAGTGATTTGTATTAAGAAGTGTAGAACGCCTTC

>Unigene606_All

TACTACAAATTCTCTCTTTGGTCCAAATATGACAAGTGTATTGACTAAAGGATACAGTGGGGACCGAACAGGCAAACAATACCGTATCTCTTACAATTGCTATAGAATAGACAAATGAACTGGCAGCAAGAATTTCTTGGACTCCACACACCAGCAATATCAGCGTTGGATACCATATCATGGTCCACGGATGGAAACCACAAGCCCACCAGTGAAATAGATTCATTAATCATTCACTGAATCGAGTAGCATTGTGCCGTTGTCAATAGTTTTTGTGGTCGCATAACAAGAACCATAAAAATTGGGAATAAAAGACATCCATATATTTCAACTACTTTTAAACAGGATGACCATTGTCAAGTACGAGTACTAAAGCACTAAACCCCATTTTTAATGGTCTATCATCTATCAACATCATCTCACGTCTGAGGTAACCAAACCCCCAAAAATCTCTCAACAAAGAACTACTAACCCAAAAAGATAGCATAGAATAACCATCAAAATGGTACTTAAGCAGCAAATAAGATGTCTCCATCATTATGCAACAACGGCCTCTTCCTCATGAAGAAGCATCTGGTCGAAGTGCCATACTCCATGCCCTTTGCCCACTCTTCTCAACTGAGCAACATATTCAGAGATTTTTTTGATGGCTTCCACCTGTTCTGCTAAGAACTCACGTTCAACAAACTCACCCAACTGCACATCATGGTTTCGCTCAGCCACACTGTGTAAGTTCAGGAGCTTTTCATTTGTTAATTTTTCCAGAGACAAAGTAAGCTCCATTGCATACAATGCATCTCCTTTTTCCACATGATCAAATTCCGAAAGGGGCATTACTATAGACTGCAGCTTCACTTTTCCGCCCCTTTTGTTCTGGTATTTCATCAATTTCTCAGCATGCTCTCTTTCTTCAACACTTGATTCCTTGAAAAACTTGGCAAGACCCTTGAGAGCAACATTGTCCCTGTCAAAATAAGCAAATATGGCATGGTACACGTAGGAGACATTGTACTCCACATTGATCTGTTCATTAACGGCAGCTTCACACTCATCAGAATACTTCTGTCTAGCAAGAGAAACTTGAGGGACAGTAGGGACCAAACTGAGCTCCTTCTTTACCTCTTCAAAGGGCTCAAAAATAACCCCAGTCAACGGCTTGTCATTTGAACCCTTTGATGCACAGAGAACAAACCCAGTCCCAGTTTTTCCACGAAAAATCGTAGGAGAAAGACNNNNNNNNNNNNNNNNNNNNNNNNNNNNNNNNNNNCAGATTGTCTCCATGAGTGCTCAGCAAAGAGAAAGCAGGAGCAGCCTTTAGCAACATATTTTTATGGATTTCTAAAGAAAGCTTTTTGATTATGAA

>Unigene624_All

AATAAATGATGGTGGCTAGAGCAACCAGGCTCTAGATATTTGTTAACTATAATTTGTCAGAATAATACAATGATTTCCAGTGAAGAACTTCTTAACGACTTATTTGAGGAAACAATAAATTAAATAACAAAGGGAGATACCGAAAGATGACAAGATAGTAGCACGAATAATAACTTTTCTTGGAATGTAACAGTTCCGAACTTGTTGTCAATCAAACAGCTATATCTTCTCATCTTAGTATTTTTAAGGAAGTTTTGCAGCGATAGCTCAAATCACAATGTTTCCACAGTATAAAGCGATGACATATACTTATTGCTATGCATTCACAATTGATATGCTTAAATCTAGTACATATTACAATACCTTAACAGAGAAAATCTCCATAAGAGCATACCAAGTTCCTGAAACTGCAAGAACAACACCTACAGTCAAGATCCCCACATCAATACACCATCCTTTTAAGTCCATTTCTTCCTTAAATACGAGCAGGTGAAATAGAGCTGGCAAAACAAATCCCAGTCCACAACATACACTACTTCCCACCAAGGACATGAAATCAGCAAAATTTGGCACCAATAAGGCCACCAAACTCACAGCTAAAACCAACACCCATCTAAGCCACAAACAATATGTCCCACCCCAAAACCTTCTCTCTGCTATCTCATAGACTGGGTTCATCATCAAGGGAAATGTGAAAAAAAGATTAATACAAAGACCAAGTTGAACCANNNNNNNNNNNNNNNNNNNNNNNNNNNTTAGCAGTAATAATATCTTTAGTTTCATCGCCAAAAGCAAAGTAACCCAGCACACCAAAAGATCCATAAAGCAATGAAATAAAGGCCATGCTTAACCCTACGGTTCTCCCAAATTTTTCCTTCTCTTTCATTTCTGACTCTATAGGCAAAACCATACCAATCCCTTCAAATGCGTAAACAGCCACCCCCATCCCGTAGAAAAACACAGACGTACCCCCAAAAGCCCTTACCTCCGGCCTGTTTCTCATCATTAGCAAAACATCCTCCACCATGACCACACCCATCGCACCAAGATCAACAATATCAGCAAATATACTCAAAGGTGCTAAATGGGTCAAAGTAGCAACAGAATTTAACACCAATTGGAACGGGAAACAACCCCATATGTAAAAACTCTTGGCAGACAATCCCATGATTTGGGAAGTTAAGCTCTGAGACGATGATGAATTGAATAAATTCACCAAAGTATTTGCAATAAAAATTAAATAACCAACACAAAATCCAGCCTGAGACAAGACAATCATGATATCCACAACTAACCTCCCAATAGAACCACAAACCGTGAAACCCAGATCGCCAAAAGAGTTGATATTGGAGAAATCAGAAGAAAAAGATTGGAGCTTGCGCCGTGTGTGGACTAAAAGCATCATACAGTAGTGGGTCAAGGCGGCAACAGAAAAGAGCATGATGAGGCTCATGATCCATCCGGTGCGTTTGAAAGCATAAGGAAGACCAAGAACGCCAGCGCCTACGATGGCAATGAAGACATTGGCAAAGGTCTTGGACTGGGACGATAGAGGCTTGTCCTTGCCTATGAGAGGCGTGTCTTCTGTTGGCCGGGCTTTCAGGGCGTTTGAGGATGATGATGCTTGTTTGTTTTCAAATCCCATGATTTATGTTACTGTTTATCTTCCAAAAAAGAAGAAGAAGAAGAAGCAAACGAGATTAAACTTTACAAAGAGAACATCACAAAAGATAGAACATGGAAATGGATGCACGAGGGAGAAGAAGAGAGAGGATTCTGTTTTTCTATCTTGGGGGACGTC

>Unigene625_All

CGTTTACCACAGCAAAATAATTAGATGGTTACTACACATGCGCCATGTATTGATCCCTATTTCAAAAATTCTAATAACATGGTCCTTCCATCTGGCGCAAAGTAAAAAAGCCAATAGCCAATAACCCTGATCACATTTTCCAGCTTTCAACACACTCAGTTGGGATACAGCTTGATTTATCAGCTTCCCATTGATGATGAGGAAGGACCATCGGACCAATGGCCATCACTTCACCAACCACCACTTCCACCTCCAGACCATCCATCATTACCAGACTCTGATTGTGAAGAACCTCTTTTGAAGCCAGTACCCCAACCAGAACTCCCATTGTCAGAGCCGCCGCCACCACCACTTCCCCAGCCACCAGGAAAAGCCTCCCTTCCAGGTGAATTCTGGACCTTAGAACCAGGGAAACTGCCCCAACCTCTATCACCATCCTTGGCATCTGAATCCCATCTGGAATTATTATAACCAGAATCCTGCCTGTCATTGCTATTGTTATTTCCTCTGCTGTTATAAGAGCCACGACCATGTCCTTGCCCACCATATGGCCTTGGTAACCCACTCTGATGACTGTCTCGACTTCTATAATCATTTCGGCCTGTTCTGGCGCCTGGCACAGAAGAACGATCTCTGTCAAAAGACTGGCCTCTCCAGCCACCATCATTTGCAGAACCACCCCAGCCACTACCCGTGGATGCCCCTGAAGAGCCCCCAGTTGCAGGGCTTCGCATTGGTACCATGGCAGCAACTGATCTAATAGACGGTGCAGATTCATGCATGGGATCATCAATGTGCCTCTGAAAATATGCCACAAGCCGATCAATGTCTTCAAACATCCTTTTCCGAAACTTGAATCCCTTGGGATATAGACCAACGTACTCGTGATGTGGATTTGTGCTCCTAATGTAAGTCAGAATAAAAGTGCCTGGATGTTCATGAGAAATCCCAAAAGAGTAAACTATCCTCATAGGATAATCCGATTTCTCAATCCTGAGTTGCTCATCAACTTCTGCTTTAGTGCCCCTTCTGAACTTGCGATAACTTAGCATTGCCTTTAGATGAGCCACCAAGGGATCAACATACCGGTCCATTACCTCATCCAAATCCTCAAAAGTGTCCTCCCCAATTTTCAGGGTCTTACCAATTCGAAGTAAACTTGTAATGTCCTTGTGTTCCTTTCCACCTTCAACTATGTCTTTGTGAGCATAAACTCCATCATAAACCTTAAGAGTTAGAGTTAAATACGATGGCCCACGAGAACTTGGACGAATGATACNNNNNNNNNNNNNNNNNNNNNNNNCCTTGTCAGATAAAAACTCCATTGCTTCGTCAGCTGTTATGTTCTGGAAGCGAGGGTGAACAATCATCCTAGGCTTAAAGTGTTTCTTTGCAAGCTCCTTCTCTTTGCGAGCC

>Unigene630_All

GATTGAAGATTGGAGGAGGAAAAGGCAGGATATTGAAATGAACAATCGTAAGGTAAAAGTGCATTCTGCTGATGGTGTTTTTCATCATACTAAATGGATGGATTTGAAAGTTGGGGACATAGTAAAGGTGGAAAAGGATGAGTTTTTCCCTGCTGATCTTATCTTACTTTCCTCAAGTTATGAGGAAGCAGTTTGCTATGTTGAGACCATGAACCTTGATGGAGAAACCAATTTGAAACTGAAACAAGCACTGGGTGCAACTTCAAATCTTCATGAGGATTCTAGCTTTCGAGATTTCAGGACTTTAATAAGATGTGAAGATCCAAATGCAAATTTGTATTCTTTCATAGGCAATATGGAGCTAGGAGAGCAACAGTACCCTCTTTCACCTCAGCAACTTCTGCTGAGGGACTCAAAGTTACGAAACACAGATTATATTTATGGGGTGGTAATTTTCACGGGTCATGACACTAAGGTTATGCAGAATTCAATGGCACCCCCTTCCAAGAGAAGCAAAATTGAGAGAAGAACAGACAAGATTATCTACTTCTTGTTCTTTATCTTGGTTTTAATGTCTTTTATTGGGTCAATTTTCTTTGGAATTGCAACAAGGGAAGATCTTGATAATGGAAGGATGAAAAGATGGTATCTCAGACCAGATAGCACTACTGTTTATTATGATCCAAAGAGAGCACCAGCTGCTGCTGTTTTGCATTTTTTGACTGCCCTTATGTTATACAGCTATTTGATTCCAATTTCTTTGTATGTATCAATAGAAATTGTCAAAGTTCTTCAGAGCATTTTCATCAACCAAGATCTGAATATGTACTATGAGGAAGCTGATAAGCCAGCACGAGCACGGACATCAAATTTGAATGAAGAACTTGGCCAAGTTGATACTATACTTTCTGATAAAACAGGAACATTGACTTGCAACTCAATGGAATTTATCAAGTGTTCTGTGGCTGGGGCCTCCTATGGTCGTGGAGTAACTGAAGTTGAGAGAGCTATGGCTAAGAGAAAGGGGTCACCTTTACCTCAAGAGGAGATAGAAGAAGAGGCCCATGTTGAGGAACAAACTGAACAAAAACCGTCCATAAAAGGGTTCAATTTTGTAGATGAAAGGATTACTAATGGTCATTGGGTTAATGAACCTCGTGCAGATGTAATCCATAAGTTTCTAAGATTACTTGCTATCTGCCACACTGCAATACCTGAAATTGATGAAGAAAGTGGAAGAGTAACATATGAAGCTGAATCACCAGATGAGGCAGCATTTGTAATTGCAGCAAGAGAACTTGGATTTGAATTTTATGAAAGGACACAAACTAGCATCTCGCTGCATGAGTTAGATCCAGTAGCTGGCAGGACAGTTGAAAGGAATTATCAACTTTTGCATATTATTGAGTTTAGTAGCTCTAGAAAGCGGATGTCAGTGATTGTAAGAAATGAGGAGGGAAAGCTGCTACTACTTTGTAAAGGTGCTGACAGTGTCATGTTTGAGAGACTTGCAAAAGATGGAAGAGAATTTGAAGAGCAAACCAAAGAGCACATTAATGAGTATGCTGATGCTGGTTTGAGGACTTTGGTACTTGCATATCGTGAACTTGATGAAGAAGAATACAGTGAATTCAATCGGGAATTTACGGAGGCCAAGAACTCAGTAAGTTCAGATCGTGAGGAAATGATTGAGGAAGTGGCAGCCAAGACTGAGAGGGATTTAATTCTTCTTGGTGCTACAGCAGTCGAAGACAAACTTCAAAATGGGGTTCCTGAATGTATTGACAAGCTTGCTCAAGCTGGAATTAAGATATGGGTTTTGACTGGAGATAAGATGGAAACAGCAATTAACATTGGCTTCGCCTGTAGTTTACTTAGACAAGGAATGAAACAAGTAACAATCAGCTCAGAGACCCCAGAAAATAAAGCCCTACAGAAAATGGAGGACAAGGATGCTGCTGCTGCGGCTTCCAAGGCAAGCATCCTCCGTCAGATTAGTGAGGGGAAGGAATTGCTCACTGCATCAAGTGAAAGTCCTGAGGCATTGGCTTTGATCATTGATGGGAATTCACTCACTTATGCTCTCCATGATGATGTCAAGGACAAGTTTCTAGAGCTTGCCATTGGATGTGCATCTGTTATTTGCTGCCGTTCATCTCCTAAACAGAAAGCTCTTGTTACTCGCCTAGTTAAAACAAAAACAGGTAATACGACATTAGCAATTGGTGATGGAGCAAATGATGTTGGAATGCTTCAGGAAGCAGATATTGGAGTTGGTATTAGTGGGGTTGAAGGAATGCAGGCAGTCATGTCAAGCGATTTTGCAATTGCACAATTCCGATATTTGGAGCGCTTGCTACTCGTTCATGGGCATTGGTGTTACAGAAGGATCTCCTCAATGATATGCTATTTCTTTTACAAGAATATTGCATTTGGCTTTACTCTCTTCTTCTACGAGGCATATGCATCATTCTCTGGCCAACCTGCATACAATGATTGGTTTTTGTCTCTTTATAACGTCTTCTTCACATCACTTCCTGTGATTGCCTTGGGAGTATTTGATCAGGATGTATCTGCACGCTTTTGCCTGAAGTTCCCGCTATTATACCAAGAAGGTGTCCAAAATGTCCTATTTAGCTGGCTTCGAATATTTGGATGGGCATTTAATGGGGTATTGAGTGCAACCTTAATCTTCTTCTTCTCCATCTCTGCAATGGAGCATCAAGCCTTTAACAAAGGTGGGAAAGTTGCTGGCTTAGAGATTCTTGGAGCCACCATGTACACATGTGTCGTGTGGGTGGTTAACTGCCAAATGGCACTATCCATCAGCTATTTCACTTACATACAGCATCTCTTCATCTGGGGTGGCATTGTATTCTGGTATATATTCCTCATGGCATATGGAGCAATCGACCCTAACATATCAACTACTGCCTACAAGGTTTTCATTGAAGCCTGTGCACCAGCTGCATCTTATTGGCTCATCACATTCTTTGTACTGATCTCCTCCCTTCTCCCATATTTCACATACTCAGCCATCCAGATGAGGTTTTTCCCCTTGTATCATCAGATGATACAGTGGATAAGAAGCGATGGACAAACTGAAGACCCCGAGTACTGTCATATGGTACGACAGAGGTCATTACGGCCTACAACAGTAGGNNNNNNNNNNNNNNGTGAGAGACGTTAGGGGTATTGATGAAACATTTTTGCATAGATTCCATTCAAAATATGCTTGTAGATTTTGTTGCAAAGAAAAGCTTCTCACTGTACATATGTGACACAGAGCAGTTCAGTTGTGGTGCTTGACAAGAAGGGTCTACAAGTGGGTATCGTTGGATGACCTGGTGTATCTATACCTTCAAAGTATTTAGTGTAGAAGATAGAATTTTTTTTTCCCCTGTTTTAAAAACCTCC

>Unigene632_All

CTTCAGAAGATACGACATCCAAATGTGGTCCAATTTCTGGGAGCTGTTACCCAAAGTAGTCCTATGATGATTGTGACAGAGTATCTACCCAAGGGAGATCTTTGTGCATTCTTGAAAAGAAAAAGAGCATTAAGAACAGCAACAGCTGTGAGGTTTGCACTTGATATTGCAAGGGGATTGAATTATTTGCATGAGAACAAACCACCAATAATTCATCGTGATCTTGAGCCTTCAAACGTATTACGGGATGATTCTGGACATCTCAAAGTTGCAGATTTTGGAGTTAGTAAGTTGCTTACAGTTAAAGAAGACAAGCCTCGACTTGTCAAGACACTTTCTTGTCGATATGTGGCCCCAGAGGTTTTCAAAAATGAAGAATATGATACCAAAGTGGATGTCTTTTCCTTTGCTTTAATTTTACAGGAGATGATTGAAGGCTGTCCCCCATTTTCTGCCAAGCAAGAACGTGAAGTTCCAAAATCATATGCTGCAAAAGAGCGTCCACCATTTAGAGCTCCAACAAAGCGCTATTCCCACGGACTTAAAGAGTTGATTCAGGAGTGTTGGAATGAGAATCCATCTAAGCGACCAACTTTCAGGCAAATAATAACAAAACTGGAGTCAATTTATAACAGTATTGGTCATAAGAAGCGCTGGAAGGTTAGACCATTAAAATGCTTTCAGAATCTGGAGGCCATGTTGAAGAAAGATCTTAGTTTGAGTGGCCACAGTCGTTCTTCACGATCGACAAACAATGCATGAGGCNNNNNNNNNNNNNNNNNNNNNNNNNNNNNNCTGTATGGTAGTAAAAAAGGATTCTTCTTTAGTTCAGATCATAGCTTTCTTCTTTGTAATTCCACCTGTTATCGAATGTGAATTCTTCCAAGTACGTTAACTTTCCCATTTAA

>Unigene636_All

GGATTTCTCAATGGTGGTAGCAAGAGAGTGCTTACTTCATATAGTGAAGCTATTCTGTTGAAGGACTGGCCTTTGGCTTGGAGTTGTTCTCCCATTCTTTCTAGACAAAATGTTGTTCCACCTGAATTCTCATGGGGAGCACTCCACCTAAGGAGTCCTCCATCTTTTTCTATAGTCCTAAAACATCTGCAGGTGATTGGAAGAAATGGTGGTGAAGACACACTTGCTCACTGGCCCACTGCATCAAGCATTATGACAGTTGATGAAGCATCTTGTTCAGTGTTGAGATATCTGGATAGGGTTTGGGGTTCCCTTTCTTCGTCAGATATAAAAGAATTGCAGAGAGTGGCATTTCTACCTGCTGCAAATGGAACACGCTTGGTGACAGCAAATTCTCTTTTTGTTCGCCTGACTATTAACCTGTCACCATTTGCATTTGAACTTCCTAGTTTGTATCTTCCTTTTGTGAAAATTCTGAAAGAGTTGGGACTTCAGGACGTGCTATCAGTTGATGCCGCAAAGGATCTTCTATTAAATCTCCAAAAAGCTTGTGGATATCAGCGTTTAAATCCAAATGAACTCCGTGCTGTGATGGGAATCTTGTATTTTCTTTGTGATACGATTATTGAGGCAAATGTGTCTGGTGGGATCAATTGGAAATCAGATGCAATAGTCCCAGATGATGGTTGCAGACTTGTCCATGCAAAATCATGTGTGTATATCGATTCTTATGGCTCTCAATATGTCAAATATATTGACACGTCAAGGTTAAGATTTGTTCACCAAGATCTTCCAGAGAGAATTTGTATAGCTCTGGGCATTAGGAAAGTGTCTGATGTTGTCGTAGAGGAACTTGATGAGGATGAAGATTTACAGACATTGGAATGTATTGGGTCTGTCCCGCTAGTACTCATCAGAGAGAAGCTGTCAAGCTTGTCATTTCAGAGCTCTGTATGGACTCTTGTAAATAGCATGGGTGGTGGTGTTCCTGCTACTGATAATTTATCTTTGGAAATCATACAAAATCTACTGGCATGTGTTGCTGAAAAGTTGCAGTTTGTTAAATTTCTCCATACTCGATTTGTGCTTCTTCCAAAGTGTCTTGATATCACCCTCACTGATAAGAATTCTGTTGTTCCAGAATGGGAGGGTGGATCCAAACACCGGAGTCTATATTTTGTAAACCGCTCAGAGACCTCTATTTTGGTTGCTGAACCACCTACTTGTATATCCATTCTTGATGTTCTTGCGATTGTTGTAAGCCAGGTTTTAGGCTTCCCTGCCCCACTACCTATAGGATCCCTGTTTTTATGTCCTGGAGGTTCTGAAACTGCAATTCTCAATATACTAAAAATCCGCCTCAACAAGAGAGAAATTGAATATACTAGTAATAAATTAGTAGGAAATGAAATACTGCCTCCAGATGCTCTTCAAGTGCAACTTCATCCTTTGAGACCTTTTTACAGTGGAGAAATAGTGGCATGGCGGTCTCAGAATGGAGAGAAGTTGAAATATGGCAGAGTTCCAGAGGATGTCAAACCATCAGCTGGCCAAGCTCTTTACAGATTCAAGGTGGAAACTGCACCAGGAGTGGTTGAGTATCTTCTTTCATCACAAGTCTTCTCATTTAAAAGCGTATCAATGGGAAATGAGGCTTCCTTAGCAACTCTGGCTGATGATAGTCGNNTTGATAAGCAGGCTATTGTCAAGATGCCAGAAAGTTCTGGAAGGACCAAAACAAAATCTCATCAGGTTGGTAAAGAGCTCCTGTATGGCCGAGTGTCAGCTGAAGAACTTGTGCAGGCTGTTCATGAGATGCTTTCTGNNNNNNNNNNNNNNNNNNNNNNNNNNNNNNNNNCTCTTCTGCAAAGAACGATAACTCTGCAGGAACGGTTGAAAGAGTCTCAGGCAGCACTTTTACTTGAACAGGAAAAGTCTGATGTGGCAGCAAAAGAAGCTGATACAGCGAAAGCAGC

>Unigene649_All

TGATTTTGGTGGGTTGGAAGAAAGTGCTACGGATATGAATGGTGGTGGCGTTGGAAGAGGGAGAGGCAGGGGTGATACTGCTGGGAAAGCATGGCAACAGGATGGAGACTGGTTGTGTCCAAATACAAGTTGTTCCAATGTCAATTTTGCTTTCCGAGGTGTGTGCAACCGATGTGGAAGTGCTCGACCTTCTGGTGCCTNTTGGTGGAAGCTCAGGGGCTGGTGGTCGTGGGAGGGGTCATAGTGCCCATAATTCTGGGGCCCCCAATCGGTCTGCCACTGGTTCTACAGGACTTTTTGGTCCCAATGACTGGCCTTGTCCAATGTGTGGCAATGTCAACTGGGCAAAACGTACAAAGTGCAATATTTGCAACACCAATNNNNNNNNNNNNNNNNNNTCCCATTACACAGAGGAGGGCGAGGTGGTGGTTACAAAGAACTTGATGAAGAAGAAATAGAGGAAACTAGGCGACGTCGGAAGGAAGCAGAAGAAGATGATGGTGAGTTATATGATGAATTTGGCAACCTGAAGAAAAAGTTCCGTGTTAAAACCCAACAAGCTGAATCTGGAAGATTGCCTCCTGGTGCTGGGCGTGCAGGATGGGAGGTTGAGGAGCTAGGTGTGGTTGACAGAGAACGAAGGGAGAGAAGCAGAGAGGGAGGGAGGGAGAGGGATGATAGGGAAAGCAGCAAGAACAGAGAACATAGTGATAGGGATAGGCACAGAAGTCGAAGTAGGGAGCGGGATAGAGGAAAAGATCGAGACCGGGATTACGATTATGATCGAGATAGAGAATATGGCCGTGACAGGGACAGAGATCGGGACAGGGACAGGGACAGGGACAGGGACAGGGACAGGTACAGGTACCGTCACTGAGGATAGTTGCAAAGGTTTTGGGATTTTGTTTGGAGTTCTTCTTCGAATAGAACTGGTTGCTTAAGTCTTTTTGTTTTTTTGGTGGGAAAGATGTGACTTGATTTAAAAGTTTTGGTGTACTTGGCATTTCTTTATCGTCTAAAATCCAAACATGTAGGAGTTGCGGTTTGGATTGTTAATGGCACAATGTTTAATTGACCATTAAGAAATCATGTTAACTTTGGGAGTTAGCTGAATTATGTTCATCTTATGTTTTTGAATGGACAGCCCTAGAG

>Unigene703_All

TATTAATGGGTGACAAAATAAGTTCCAATACTGCTGGAGCTGCTGACAATTCTCCTTTTACTCACTTGCAATTTCCGAACAGATCAGATGCAAAATCTATTAATAACCATAGAAGTCAGGCAAGGCAACGAAACCACTCAGAAGGAAAGAGAAAAGAACGAAACCTGGATAATGGTGGTTCTTTCCATCGTAATGGTAACTTAAAGCAATTTGCAGATACACAGTGTTCACATGGGGATCCACCTCATCAAGTTTTTTCTCCTCCAGGTGCCCAGCAACGTAAACAAAACATAGTTTCAGATTCCTTGAATCACTTGCAGACACATATTCCATATATGCATATGGACCATGGTCATTCTTCAAATCAAACTTCAGTTGGTCCAAATCAATCTGGAATCAAATCTGAAAGTGATGGCATCCCATCTCTTTCTCCAAAGGAGTCTACTTTTGAATCAAATCAAGTACAATCCATGGACAGTTCTCATNNNNNNNNNNNNNNNNNNNNNNNNNNNNNNNNNNNTAACAACCAGGAAGATGGAAAGGCTATATCATAACCAGGATTTACAAGTCCCATGCGCCAGGAATTTCAAATGTGCAAATACTGCGAGTCCAACAGGATTTTATGATTCAGTTCAGAATCAAGCTCATCACTCTGGATACGAGGTTGAAGGTCACAGTGAAATTGAAGGAGTTAGCATGGGGATTCCAGCAGAGCTAGATTCTTCAACTGCCCAAGAAAGCTCTTGTATGAGCTCTGTGTTAGATGAAATCTCACTAGAAGCAACTAGTTTTTGCCAGCTACAACAAGTCATGGAACAGTTGGATATTAGGACAAAACTGTGCATAAGAGATAGTCTATATCGCTTGGCTAGGAGTGCTGAACAAAGGCATAATTGTGTGAATGCAAATGGCGGTACGAGAGATGATAGAGACACAAACAATCCATTGATGGCTGAAGAAACAAACGAGAGCACTGGGTTCTTTGATATGGAAACGGATACAAATCCCATAGATCGGTCTATAGCACATTTATTGTTTCACAGGCCCTCAGATCCATCTTTGATGCCTGTCAATGATGGTTTGTCACTAAAGTCACATGCCATGGTTCATGGGTCTGTAACGAGTCCATCCATGATGGCTAAGGAGCAAGTTTGCCAGGATGAAACTGCCAGTGTTTCAGATAAAAGTTTGTTAGTGAGTGGCCATAAACAATAAATACTAACATGGCTCAATTATTGTAGAATGTCTATTTTTTGGATTACTGCACATCATGACGTACAGTAACTTTTTCATGTGTGTGGTTCCAGCATAGATAACATTTCAATTCTATGTTTGGATCTCATCGTTTCTCAGATGTTTTGTATATCCTTGTAATGGTTTAACCCGATAACACTCTTTTAACTATCATATTGCTAGGATTTTTGTTTGTTGCTCTGATCATGTCAGATTGTAAAAACCATTCATTGGTGTTCGAGTTTAAGGCAAAATGTATATAATTGTCATAAACTCGAACACCA

>Unigene704_All

GGAAAAGCAGTTGGCCCTGTTGCAAATGATTTCAGATCAGGAGAAATGCAAGAAGGCAACAATGATTTCGTTGGAGAGCTATCAGTTACTCTTGTAGATTCTCGGAAACTTTCCTATGTCTTCTATGGTAAAACAGATCCATATGTTGTTCTAAGCCTGGGAGATCAAACTATACATAGTAAAAAGAACAGTCAGACCACTGTCATTGGGCCTCCTGGTCAGCCAATTTGGAATCAGGATTTTCATATGCTTGTTACAGATCCTAGGAAACAAAAATTATACATCCAAGTGAAAGATTCCCTTGGATTTACAGATTTCACTATTGGTACTGGAGAGGTTGATCTAGGATCTCTCCAAGACACAGTACCAACAGACAGGATAGTGGTTTTACGAGGAGGTTGGGGGNNNNTTCAGAAAAGGCTCCTCTGGAGAAGTACTACTTCGACTAACTTATAAAGCATATGTTGAGGATGAAGATGATGACAAGACTGCAGTGGAGTCCATTGATACAGATGCTTCAGATGATGAGTTGTCTGATTCTGAAGAATCAAATGCTACTTCCATGTCAACTGAAAGGGAGTCATATAGTGAATTGGATAAAGAGTCATTTATGGACGTTCTAGCGGCTTTAATTGTCAGTGAGGAATTTCAAGGGATCGTGGCATCTGAGACAGGAAGCAACAAAGTTTTGGATGATGTCTCAGCTGCAGCATCACGTGGTCTTAATACTGAATCNNNNNNNNNNNNNNNNNNNNNNNNNNNNNNNNNNNNCAGTCATATTTTGGCTTGCTGTGTTTACAAGCATATCGGTTCTAATTGCTGTCAATATGGGTGGCTCAAGTTTCTTCAACCCTTGATATGACATGGAAAAGCTTGCAAAGAGTTGGTTTTAGTACTCCTGCTTAAGAAAGCAATTCCTAAATCTTCTTTCTCCTTATCTGGGCATTTGTTGTCAGTAGAGGAAACACCATAATTCAGCAAGCATGGAATGGTGGACCCATTTGGTTAACTTAAAAAGGGGACGAGAATTTAAATGGTTGAGACGGTGAAGCAAAGGGCAAACCCACCCTCACATGATGGGGATTCTTACCTCCTAGTTGGTTTTTAGTAGAGAAATATTCTTTTGGCGGCGGGAAATTGATGAATTAGGTGTCATTTTTATCTATTGGTTTTGGTTGGGTATTTACTGTGAATGTGAAGTGAGAGCATTCATTATTTTTGCCGATTTATTTTTAATTGTAATTTATATCTCAATTGCTATTATTTATAAAAATTGA

>Unigene715_All

GCCTTGCATGGATGGCTGCATCGTTGCTTGGACCCAGTAAAAGCACTGCAATTTATGAACCCGAAAGGAATGGAATCTCAGCTAGTGTTGTGGGTAGGGAGACGTTAAGGTTATGAACTGCTAAGTATGCAGCAATGCCAGCAGCATAACCAGCAAAAGCAAAACCACTCACCTTCCGGAAGTACCAGAAGAAGTCCACCTTTTCCATCCCCATATATGCAACTCCAGCAGCAGAGCCGATAACTAGCAAGGAACCGCCAGTACCAGCACAGGACGCAACTAACTGCCAAAACTCAGAANNNNNNNNNNNNNNNNNNNNNNNNGTGAGATCATACATTCCCATCGCTGCTGCAACCAGTGGAACATTGTCTATAATTGCTGATACCAACCCTATTGCAGTTGCAACAAGTTCAACATTTGGAATATGCGCATCAAGGTAATTTGCCAATTGGCGAAGAATCCCTGCTGCCTCCAGGCTGCTGACTGACAAAAGGATTCCAAGGAAAAAAAGGGCTCCTTGAGTGTCAATCCGAGATAAAGCCTGCGGTACTTTTAACTTCTGCCTTTCTGATTCACCATAATGAATAGCATCTGTAACAATCCAAAGTACTCCAAGCCCAAGAAGCATGCCCANNNNNNNNNNNNNNNNNNNNNNGGCCTTGAACACTGGAACAAAAATCAAAGCACCAAAACCCACAGAAAAAACAAGCTGTCCCCGAGGAGCCATACGTTCAGAAGCCAAAACATTAGGGGATTCCTGTTCCTTTCCATTAACTTCACTAGTGAGAGACATAAGAGCCAGCGGAATAGCTAGAGAAACAACTGAAGGTACTAGCAAACCCTTCATTGTTGGCAATGTGGATATCTGACCATGTATCCATAGCATTGTAGTTGTAACATCACCTATAGGAGTCCATGCACCACCTGAGTTCGCTGCAATCACAACAACAGCTCCTAGAAGCCTGCGGTATTCTGATGGTGGTACTAGTTTCCTCAACAAAGAAACCATTACAATGGTAGATGTCAAGTTGTCAAGGATTGAACTAAGGAAAAATGTCACAAACCCAACCACCCAAAGTAGAGTCTTGGGCTTTCGAGTGGTTATATTGTCAGTGACCAGCTTAAATCCTTGATGAGCATCAACAATCTCCACGATGGTCATGGCACCAAGCAAGAAAAACACGATCTCACTTACTTCTGCAGAAGCATGTGTCAACTCCGAAACAGCTATTTCAGTTGAAGGCGCCCCAATGCTTCGAACTATCCATAAGCTCACAGCCATCAGTAATCCTACTCCACTTTTGTTGAAAGCTAGAGACTCTTCAAAAATGATGCCTGCATATCCAATTCCGAAGAGTAATGCCATAGCAAGATCCTGATTGGCTGCAACCCAGGAATGGTTAATTGCTGCAGCCCCTGTTGCAGCTGCTGCGAAAACTGCAAGAGCTTTTAGCAAATCAGTCTTTGGCTGGTAAGTGGCTGCAAAATCTTGTGAACTAGTTTCATCTACTGAACATAAAGGATCACATGAACCAGC

>Unigene716_All

TGCCGACTGACATTTGATATTAAACTGTATAAATTACATAGAATACAGAACAAATATCACCGCAAGTTACTAAATCAGCCCAAGACAAGAAGATGGCCAAAATTGAAGAATAAAATTGATTGAAAGGAGATTTTTAAAAAAATGAGGCTAAAATTCCTAAATCTCTAAAATCAAATCAAGAACGGAATGGTCAAACCTCTCCGAGAGTACAACAATCCACCAAGAATCCTGCTCCCAAAACCCTAACTGCCCATTGGATCCTTAAAAGCGCGAAAAATCACGAGAATTTCACCCGTTAGAGCCAATTTCTAAACATCAAATTTCTTAGGCTGTCGCCACTGTGTCCAACTCTTTGCCAAAGAGCCAAGGAAAGAGGATCTGGGAGGAACTCCTTTCTTCCCATACAAGTGTTTGCCACCCAAGTCCGTGTAAATTGGGCCCTTACCCTTACTTCTTTCGATCTTGCCTATTTTGCCATTCTTCAAGTGGTACCGTATACCAGACCAATTTATAGACTGAGAGAAATGGGAACTGAATGCAGATATGGGGTATAAGAAGTTATCCACCAGCAATGCAATGAATACCAGAACCCAGTTATAAGTTGCAAGAGAGAGTCGAGGTGCCTCAGGGGATAGCATGTTGCACAGTTGAACTTCTATCCTAGTCAAATTCCACATTGAGAAAAGTTCTATAAAAGTGCACACAGCTAGGCCAGTGACCAGTAACAACCCATTAGAAACAAAGATTGTTTCCTCATGTGCATATCCTCGGATATAGATTCGCAGTGCTGCTGCAACATGAGTCATAGCCATAAGGTATGGCGCTACAAACCCCCATGACAAATAGCAGTGGGAAGAAAATAATGCACGGTTCATTANNNNNNNNNNNNNNNNNNNNTATATATGATTCCAATACAAATGTTTGCTTCCTCAAGTAATTCCAGTACCTTGAAAAACTGAGATCGCTAGCAAGGGGGTGAGGAAAAACAGCAACTGGAGGTGATGTAATAAGCCTCTTATGAGCACCAGCTAGAGCTGCAAGAGTCATATCATCAGAGTATCCACCATCTCGTAGTCCTGAGACCACACCATAGCGGTCATATCTAAAATCATCAGCATGCATCATCATGCACCCGCCCCATAGAAAGAATGTTCTTCCACCAGTGGCAAAGCCCATTGAACAAGG

>Unigene735_All

CAGTCATTTCCCTTTCACAGTAATAACTACAATAGTACGATTTCGAATTCTAATTCCAATTCTAGTGTTAATTACAATTCCACTTTTGTGTTCAATAACTTTGAGGGAGAAAGTTTGAAATTCAAGATTGAGGATTTGAGATCTGCCCTTCTCAATCAGATTTCGCTGAATAAACAAATTCAACAGGTTCTATTATCCCCTCACAAGTCCGGGAGTGCGAATGTATCTTCTTCAGATTTCGATAACGCTGCTGGGCCATTTCTGGGTTCTGGGTTTGGTTACGATAGGTGTAGGAAGGTGCAGTCGAGATTCTTGGAAAGGAAGACCATAGAGTGGAAACCCAGATCAGACAAGTTCTTGTTTGCCATATGCTTATCTGGGCAGATGAGTAACCATTTGATTTGCTTGGAGAAGCATATGTTTTTCGCGGCTTTGTTGAATAGGGTTCTGGTTATGCCAAGCTCTAAATTCGATTACCAGTATAGTAGGGTGCTAGATATTGACCATATAAATCAATGTTTGGGAAGAAAGGTGGTTGTCACATTTGAAGAGTTTGCACAAATGAGGAAGAATCATATCCATATTGATCGGTTTATATGTTACTTCTCTTCACCTACTCATTGTTACGTGGATGAAGAGCATATTAAGAAGTTGAAAGTGTTGGGGATTTCAATGGGGAAGCTTGGGTCGCCTTGGAAGGAAGATGTTAAGAAACCAAGTAAGAAGACAGTTGAAGATGTGCAGGCCAAGTTTACTTCCAATGATGATGTTATTGCAATTGGAGATGTGTTTTTTGCTGATCTAGAGAAGGAGCGGGTGTTGCAGCCTGGTGGACCTCTTGCTCACAAATGCAAGACATTGATAGAGCCAAGTAGGCGTATAATACTTACTGCCCAGCGATTTATTCAAACTTTCTTGGGGAAGGACTTCATCGCTCTCCATTTTCGACGGCATGGCTTTTTGAAGTTCTGCAACGTGAAAAAGCAAAGTTGCTTTTACCCCATTCCGCAAGCTGCAGATTGCATTGTGCGGGTGGTTGAAATGGCCAATGCACCAGTCATCTACCTTTCCACTGATGCAGCAGAAAGTGAAACTGATTTGTTGCAGTCACTAATTGTACTGAATGGGAAAATTGTACCGCTTGTCAGACGTCCATCTCACACTTCAGTTGAGAAGTGGGATGCTTTGTTGTCAAGACATGGCATTGAGGGCGATTCTCAGGTAGAAGCTATGCTTGATAAGACAATCTGTGCTATGTCTAGTGTTTTCATAGGAGCCTCAGGTTCCACTTTCACTGAGGATATTTTGCGGCTGCGTAAGGATTGGGAATCAGCTTCTTTATGTGATGAGTACCTATGCCAGGGTGAATCGCCGAACTACATAGCAGAAGATGAANNNNNNNNNNNNNNNNNNNNNTATTATTTTCTGCTATACAATGTCATTCCCTGTGAAGATGCTTTACCATGAATTACCTGCAAGAACGCAATTTATTCTTCCTGGGTTGATGCCTTTTACAGCTAATGAAGCTGAATCTATTGCCTTTGTATATGTTAGTGTATAATTGTAGCATTTCTATTTTGCACATAAGTTTGTGTTGATTTTTGCAAGACACGCGTTTAAAATACATCAGCTGAAGAAATTGTTCTCCC

>Unigene737_All

GGTTTAAGGAAGGTGAATTATGTCAGTGGTCACCAGCTCAAGCAACTTGGCTAAGGGAGTGATTTTCACAGAGCACTTCAGTTGGAGAGGCATGCAGTTTTCAAGAGTTTTCCTCTGAGCAGCTGTATTTATTTATTTTTCTCAATTTGGAATAATTCTGTCTCAGTGCATGAAGATTCAATGTGAAACATGTGAGAAAATTCAGATCAGTTTCTGCTCTTTGCNNNNNNNNNNNNNNNNNNNNNNNNNNNNNNNNNNNNNNACACTAGATTGAGGTATGGAACTGACCTTAGATTGTGTTTAGAAAATATACCTAGTAGCACCCAAGTTTCTGCTATTCTGAGGATTAATCTTGGCAATTCATTAGATATTAAAGAAAATGTCATAAGCTCAGTTAGGCAGCATTGTGCCATTGTCATTACCATATGTATTGAGGGGGCTTAAGGTTACTTGCTGGCCTAATTTGGCATTAA

>Unigene768_All

TCTCTCGATGACAAGTGTCAACATTCAAGCTTGTATCAGGGTTTCCCAGTGGGCTATAACCCACTGGAACACAAAAAAAAAAAACCGTGAGCTCTGAAATTTGCATATTTAGCACTAAATCCTGGTTGAATCTCACAAGTCAGATATTCATAGGTGTCATCATAATTCATGAGCAGGTTATGCAAAAGGGCTTTTCAACATCTAAAAGAGCTGAGCCTGAGCTCTAGAGGAATGGTCGCCGGAGTCAAGCCTTCTCAACCTAAACAGCCACCTGAATCTGCCAAGAATTGCGAGGCTAAATGGGTTTCAGTCAGGGGAGCAGAAGTAAAGGAAGTTGGGATTGACGAGAAAAGCAGGGTAAAGCAAGCTGAGAAGGCTGAGAAAATCATGCATGTCGTCTGCTGGGGACCTCATATGATCTAACTAGGACTCCAATACTTCTGGTAAATTACTAGCTTGTATTAAAATTCATGGCTTGGCAATGTTTTAGGAGAGCTGATGTACTGTATCCTATGGTTATACCCTTTGCAGGGAAATGCAGATATAATTGTTAAAGACCAAGTAAACATNNNNNNNNNNNNNNNNNNNNNNNNNNNTCAAAGCATCATATGTATTTGCAAGAGCAAAACCGAATTCTGCATAACAAAAGCAATGCAC

>Unigene772_All

ATTGCTTGTCGAAGTGATGATGATAAAATGGCTGATTACCTAGCTTCATTGAACCATGAGGACACTAGACTAGCAGTTGCATGTGAGAGGGCCTTCCTTGAAACCTTGGATGGATCATGCCGCACTCCAATTGCTGGGTATGCTTGTAAAGGTGAGGACGGTGATTGCATATTCAAAGNNNNNNNNNNNNNNCCCAAGTACTAGAAACTTCTAGAAAAGGTCCCTATACTTTTGACGATATGGTTATGATGGGAAAGGATGCTGGCAAGGAACTTCTTTCGCGGGTAGGCCCAGGATTCTTTGATAGTTGAAGAGCTTAATAGCAGCTTAAGAAGGCTGCATGGTTTAATTTTTAGGCGTAGCACTTTAGGCTTCCTGTTCCTTGATCCCTCTTGATCTATTATTCATCATATAATTAAGAGGAATAGCTGTTTGATTTGTTGTAAAGTAGAAGGGAATGTTTGGAGGCTGATAATTTG

>Unigene778_All

AGCTGCTGCAACAACAACAAAACATGAAAGCATTCCAAGATCTGAGTAAGCTTCCCCAGTTCAAAGACCTAAAAATGCCTCCTAATAACAACCAGAACCAGAAAGCTGTCAGGTTTGCCCCCCTTGAGGATGAGGACTTGAGTGATGACGACTTTGATGAGTTGGATGGCGAGGATTTTGATGATGAGGATGATTTTGATGACGAGCTGGATGATCCTCGACATCCGCTTAGTAAGATGAAGCCCAATATGCCCAGTGGCAACATGATGATGAATGGTTGGCCACCCCACCTCTTGAATGCTCAAAAGGGTGCTGCAAATGACGGTGGCAACGGTAAGAAGGGGGGAGGCGGCGGTAACGTAAATGGAGCTGTGCCTGTACAAGCAAATATGGGTGGTGGTAAAAAAGGCGGTGGAGGCAGCGGCGGCGGTAATAATAACGGAGGAAATCAGAATCAAGGTGGCAAAAATGGTGGTGGAAAACCACAAGATGGCAAAAACGGTAACAATGGAGGTGGTGGTAATAACAAGAACGGTAACAACGGCAGTACTGGCGGTGCTGGAAATGGTAATAACATGCAAATGAACGGCGGTAAAAAGGGAAATAACGGCGGCGGCGGCGGCGGTGCTGCTGCTATGAGTGATGCTTTTGGCGGTATGGGTGGGCCCCATGGTAATATGGGTCAAATGGGTAACCTGAACATACCCATGGGTCAAATGGGCAATATGCNNNNNNNNNNNNNNNNNNNNNNNNNNNNNNNNNNNNNNNNNNNNNNNNNNNNNNNNNCGGCGGAGGCGCAGGCCCTAATGGGTACTTCCAGGGAGCGGGGCCAGATCTTATGCCTGGTAATCCATACCACCAGCAGCAGCAGCAGCAGCAACAACAGCACCAGTACATGCAAGCATTAATGAACCAACAGAGGGCAATGGGTAATGAGAGGTTTCAACCTATGATGTATGCGAGACCCCCACCAGCAGTCAATTACATGC

>Unigene818_All

TCAAGAATGGGGTCTCATTTGAACTTCAAGGACTTTGGTAATGCCCCAGCGGGGGAGAGCAGCAATGCAAAGCCATCAGGTTTAGTCCGCCAGCCTTCAGTATACTCATTGACCTTTGACGAGTTCCAGAGCACATGGGGTGGAGGATTTGGAAAGGACTTTGGATCAATGAACATGGAGGAGCTATTGAAAAACATATGGACAGCTGAAGAGACTCAGGCAATGACAAGTTCTGTTGGTGTTGTAGAAGGAAGTGTCTCTGGTGGGAATTTGCAGAGACAAGGGTCGTTAACTTTGCCTAGGACACTAAGTCAGAAGACTGTTGATGAGGTTTGGAAAGACTTGATGAAAGAAATCAATAGTGGCGCTAAAGATGGGAGCAACATGGGATCAAATATGCCTCAGAGCCAACAGACTTTGGGAGAGATGACTTTNNNNNNNNNNNNNNNNNNNGCAGGGGTTGTGAGAGAGGATACTCAGCTAATTGGAAGGCCAAATAATAGCGGATTCTTTGATGAATTATCCCGTCTAAATGATAACGGCAATAATAACAGTAACAACAGTACTGGTTTAACTCTTGGGTTTCAGCAGCCAAATCGAAACAATGGACTTGTGGGAACCAGAATAGTGGAAAACAATAATAACATAGTTGCTAGTCAGGCTGCAAATTTAGCCTTGAATGTAGGCGGAATCAGATCCTCTCAGCCACTACCTCAGCAGCAGCTGCATCAGAACCAGCCCCCGCAGCAGCAACCACTCTTCCCCAAGCCCACAAATGTGGCTTTTGCATCCCCTATGCATTTAGTAAACGGTGCTCCGCTTGCCAACCCAGTAGTGAGGAATTCAGTTGTTGGGATTGCGGATCCGTCTATAAACAATAATTTAGTTCATGGTGGAGG

>Unigene820_All

GCCGCTTACGGTGGTTCCGATATCGTTTGGACTCCATATGGGCCAGAGTGGAGGATGCTGCGAAAGGTGTGTGTTCTCAAAATGCTTAGCAACACTACCTTGGACTCCGTTTACACCCTTCGCCGCCATGAGGTCCGACAAACCGTGAGTTACCTTTATAGTAAGGCTGGGTCACCCGTGAACTTTGGAGAGCAAGTCTTTTTATCTATACTAAACGTGATAACCAATATGTTGTGGGGAGGCACGGTACAAGGGAAGGAGAGGGCTAGCCTAGGAGCAGAGTTTAGGGAAGTGGTTGCGGACATGACTGAGTTATTGGGCAAGCCCAATGTTTCGGATTTTTTTCCGGGTTTGGGGCGGTTTGATTTGCAAGGCCTGCTGAAGAAGATGGATAGGTTGGCGAAGCAGTTTGATGGCATATTTGAGAAGATGATTGATAAAAGGGTGAAGATGGACGGAGATGGCAGGGATAGTGGCAAAGATTGTAAAGACTTCTTGCAGTTCTTGTTGCAGGTGAAGGACGAGGGAGATACCAAGACACCGCTTACCATGACCCAGNGTTGCTCATGGATATGATTGTGGGTGGTACAGACACATCCTCAAACTCAATTGAGTTTGCCATGGCAGAAATAATGAACAAGCCAGAAGTGATGAAAAAAGCTCAGCAAGAATTGGATGCAGTAGTTGGAAAGAACAACACAGTAGAAGAGTCTCACATAGACCAATTGCCTTATCTATGTGCCATCATGAAAGANNNNNNNNNNNNNNNNNGTTAGTCCCTCACTGTCCAAGTGAAACCTGCATTGTGGGAGGCTACACTATTCCTAAGGGTGCTCGAGTTTTCATTAACGTGTGGGCGATCCATAGAGATCCTTCAGCTTGGGAAAATCCATTGGAGTTTAGACCTGAGAGGTTTTTGGATAGTCAATGGGACTACAGTGGAAGTGACTTCAGTTATTTCCCATTTGGGTCTGGACGCAGAATTTGTGCCGGGATCGCAATGGCTGAGAGGATATTTTTGTATTCACTTGCTACCCTTCTCCACTCTTTTGATTGGACATTGCCAGAAGGAAAAATTATGGATCTTTCAGAGAAGTTTGGAATTGTTTTGAAGTTGAAGAATCCTCTGCTGGCCATTCCAACACCAAGGTTATCCGATCCTGCACTCTATGAATAGGTCCTAATTGCACAAATAGTTTTATGTT

>Unigene847_All

TTGTAAGCGCAAAATCTTAATTGTGGGTGGGGACTACCGCATGTCTCGGGGCTATACATCAGCAGGACCAATGGCTCCCTTCTACGTATTTCCATGCGGGCATGCCTTCCATGCACAATGCTTGATTGCCCATGTGACACGTTGTACCAATGAAACTCAGGCTNNNNNNNNNNNNNNNNNNNTGCAGAAGCAACTTACTTTTTTGGTTGAGGGAAGTAGGAAGGACTTGAATGGTGGTATAACAGAGGACTACATTTCTAGCACAACCCCGGCAGATAAGCTCCGCTCACAGTTGGATGATGCAATAGCAAGCGAATGCCCATTCTGCGGTGAGTTGATGATTAATGAGATCTCTTTGCCCTTTATTCTCCCCGAGGAAGCACAGGAAGTCAGTTCATGGGAGATAAAACCACATAACCTTGGAAGCCAGAGGACCCTCTCTTTGCAAGTGTGATCATATTCNNNNNNNNNNNNNNNNNNNNNNNNNNNNNNNNNNNNNNNNNNNNNNNCCCTGTGATGTGGCTCTGCCCGAGTCTCGGTTTTAATTGGGTCATGTGACTCCCTATAGAG

>Unigene850_All

GTAGAGCCAAATGGATCAGAGTCGTCAAATGATGAGAATTGACTATTATCAGTCTCTCTAGAGCCACGTACAGAATCAAATCTGGCAAAGGAATTGCCATAGGACTGGAAGAATCCACTGTCATTGGCATTAAATGAATCAAATCTTGAGAACCCATGATCCTGATCCGAGTCTCTAGTGTCTCTAAATGAATCAAAACTTGATGGGAACCCATGACTGTTATCAGTCTCTCGAAACGAATCAAACCTTGATGGGAGACTCTGACTGTTATCAGTGTCCCTAAACGAATCAAACCGTGAGAAACCATGACTCTGATCAGAATCTCTGAAAGAANNNNNNNNNNNNNNNNNNNNNNNNNNTGGATTCTCTAAATGAATCAAACCTTGATGGGAACCCATAACTTAGATCAGCGTCCCTAGTGCTGTTCATGGAATCAAATCTTCCAAGTGAATGGCGTGGGGATTGAAATAATCCACTGTCATGCATATTAAAAGAATCAAACCTAGAGATGCTGTCAAAGGACTGGTCCTCCGACCCTTCATTAAACTTTCGTGGAGAATTCCCAAAATTATAGGCTGGGGTGCCAGGAACAGAATCTGCAAATCCAAATGAGTTCTTCCCATGGTTATAGGCTGGGGTGCTGGGAACAGAATCTCCAAACCCAAAGGAGCTCTTCCCCTGGTTATAAGCTGGGGTGCTTGGCACAGAATCTGCAAACATAAATGTGCTCTTTCCC

>Unigene852_All

CCCACCCCAAAACTATAAACATCTGCTTTATTTGTTAAATAGCCCCGCATGGCATACTCTGGAGCCATGTATCCTGTTGTTCCAGCAATTCTAGTGATAACATGTGTCTTCTCTGCTTCGTAGAGTTTAGCAAGTCCGAAGTCCGATATTTTAGCGTTAAGATCTTTGTCAAGCAGCACATTTGTGGGCTTGATATCTCTATGGACAACCTTCAGCTTAGATTCTTCATGAAGAAAAGCCAGACCCTTGGCTATACCAAGGCAGATTTTGAACCTAATTGGCCAGTTGAGTTTCAACCGTATCTTCTCTGAACCAAATAACGCATGTGCAAGGGAATTATTTTCCATGTACTCATAGACCAGCAAGAGTTGGTTATCTTCTGTGCAACATCCCAATAGCTTTACAAGATTTGGATGTTGCAATGCAAAAATAGTTCCCACTTCATTTACAAACTCACGAGTGCCTTGCTTTGATTTTGATGACAGTTGTTTCACTGCTATTGCTGTCCCATTTGGAAGCACACCCTTATATACAGATCCAAAACCTCCTTCACCAATCTTGTTCACAGGATTGAAGTTCCGTGTGGCAGCTTTTATTTGCCGGAAGTTGAAAGAACCTCCTGGCTGTAGTTCAAGACCTTTTAGTTCTTTGTTATGCAATTCTTTTCCGCCAAGAATGCCTAGTTTACATATGAAAACTAAAAGCAATAGTGGGAGAAGGAATGCAGATCCTACGAGCGCAGCAATACTCATTGGTGATAATTTTGTGCTGGGTTTCCTAGGAACAGGAAAAACTGAAATAGCAGATATAAGAGGACCATAATATTCCATTGGGTTACGAATTGAGCCCTTGCCAGNNNNNNNNNNNNNNNGGATCTCCAGTTGATGGTTCCTCACAGTAGCAATGAAGTCCAAAGATACACTTTTATTCACACCCATAGCAGCTTCTCTAATATTGAAATTTTCAAGTACCTTCTTGCCCTGAATTGCCACATCAAACAAACGCTTCCCCTTGCTGCTGGGATCTTTATTTTCTGAATACATAATCTCTGCAAAGTGAAGTTTCACTATGTATTTGTC

>Unigene865_All

TTTTTTTGGCTTCACCTTCATTGTTTTTTCAATTTCCATTACTGAAGTGCTTTGCACAACCTTCCATGGCCAACCTTCTTCGAAACGCTTCTTCTCTGAGACGTGCTCTACTTTCTCCCGAGGTCTTAAGGAGAGGAATATTGGGGATGTCTTCTCAGAGTTGCAATTTTTCCACTAAAGGTAGAAGAAGATCAAAGTCGGATGGAAGTGAATCCAGTGAAGACAACTTGTCCAAGAAGGAGGTGGCTCTACAACAAGCCCTGGATCAGATTACTAGCACGTATGGAAAGGGGTCTATCATGTGGTTTGGCAGGTCAATTGCCGCAAAAGATGTTCCAGTGGTGTCCACGGGTTCTTTTGCTCTTGACATAGCACTTGGAACTGGGGGGCTTGCAAAGGGGCGTGTTGTGGAGATTTATGGTCCTGAGGCTTCTGGGAAAACCACTCTTGCTCTACATGTAATTGCTGAAGCGCAGAAGCAAGGAGGTTATTGTGTTTTTGTTGATGCTGAGCATGCTCTTGACTCATCATTGGCTCAGGCCATTGGAGTAAACACTGAAAACTTGCTTCTGTCGCAACCTGATTATCACAACCTGACTGTGGCGAACAGGCTCTCAGCCTGGTGGACATGCTTATAAGGAGTGGTTCTGTGGATGTTGTGGTTGTTGACAGTGTAGCTGCCCTTGTGCCTAAAAGTGAGCTTGATGGTGAGATGGGCGACGCTCACATGGCAATGCAAGCTAGATTGATGAGCCAGGCGCTACGCAAATTGAGCCATTCTTTATCGCTGTCACAGACAATTTTGATCTTTATAAATCAAGTGAGGTCAAAGATTAGTACCTTTGGAGGATTTAGTGGGCCAACTGAAGTAACTTGTGGCGGTAATGCTTTGAAGTTCTATGCTTCAGTGCGCTTAAACATAAGAAGGGTGGGGCTTGTAAAGAAGGGGGAAGAGACTGTAGGAAGTCAAGTTCAAGTGAAGATTGTAAAGAATAAACTTGCTCCCCCATTTAAAACTGTCCAATTTGAACTTGAATTTGGAAAGGGAATATGTCGAGAATCAGAGATCATAGATTTGGGGGTAAAACACAAATTCCTTGGCAGGGCTGGTGCGTTTTACAATTACAACGGTCAGAATTTCCGTGGAAAGGAAGCTCTTAGACGGTTTCTAGCTGAAAATGATGTTGAACGGGAAGAACTTGTGATGAAGCTCAGGCAGAAGCTACTTGATTTTGGCTCGAATAAGGAACAGGGAACAGAGGCTACTGATGGGGAACCTGTAGAAGGAATCATTTCACCTGATTCTACCGATGAAGAAGTAGTTACTGCAGTAGAGGCTTAGTGTGGATTTACAGGACATTGTAGATTGGGGTTATCCTGCTAGATTCCCTGGTATGAATCAGTTTTCAAAGGGATTCTGTAATCCCCATTTCNNNNNNNNNNNNNNNNNCGGCACAAGCTACCATTGCAATGCTTGTATATACACATGGCATTAGTTAAATGTAAGCTGGTTAATTTTTGTAGCTTTACGCAGGCCAAGCGCAAATTTCTTTAGATGTTGTCCAACCCCATTGTGTATGCAAACATTAAGTTTAGAAATGAAAGTCCA

>Unigene876_All

CTGCAAAATGACATGGGTTGGCTCTCATATCCTTTATTATGATTTTTTATCCACAATCAAAAATTTTTTTGTTCATTGCTTGTATCTACATCAAGTAAAGCAACTAGAAAACTACTACTACTACACTCACTAAAACTTCTTTTGTACTGGAGCTGGAGGAGGCCTGCTGCTATTTAGAAGACTGCCTCTTCTTGCCGGAAAAGATAAAGAAGACAAACCTCTTGAGACCTTCTTTGATATGATAGCTTTTGGAAGAGTGTATGGTTTGTGGGCATCCAATCCATTCCCATAGCTCTTAGATGCTTTGATCTTCCTCATATAAGGAGTTTCCTTCTTTGGAAGGTCTTCAATGCTTGTTACCCTTGATAGAGATGTAAAAGACTGAGACTTGCCTTGATAATATTTGGATAATCCTCTCTTGATGGGTAAATGGGCCATGAGTTCAGAAAATTCAAACAAAGGACCATTGTTAGAATTTGAAGAAGAGTGTAAGGTAGAGGTCGGTGAAGATGCATCATCTACCATATCTGACGAACAAGTTGATCCATTAGAAGTACTTGAATTATCAAGTGAAGATGCGTTGAAGATATTATCATCATCACCTTCCACGATCAGCCATTGATCTTCATGCTTTGCGTTATGCCTACCTTCCTGTTGTGGCGCAGAGGCTAGTACTGGAGCTTGAAGCATTCTTTCNNNNNNNNNNNNNNNNNNNNNNNNNNNATGGATGAGAGTTCTTGTTCTGAAGAAGAAGAAGAAGAAGAAAGTGCATAAATATTATGTCTCAC

>Unigene883_All

AAAAACCCCCATGATTCTGAAAAAGTTAGCTTTGCTTCTTCTCATGCTCCACATGTGAGACCCCACATCTAGCATGTATTCAACAACCTCCTTCCTTAAGGGTGGCTCTGCACGGCTCAGCCTCATGGACACAATCTGCGTTGCCTGGTGCCTCAAACTATCAAGTTGGCTAACTGTNNNNNNNNNNNNNNNNNNNNNNNNNNNNNNNNNNNNNNNNNNNNNNNNNNNNNNNNNNNNNNNNNNGGACGAGCAAGTGAAACGAACTGCCAAATGAATTTCTCCCATCTTCTTTAGACCATTAGGATGCAGCACAAGGAGAGGATAAGAGTGCGTGTAAACCCGATCAGTTTCAAGTGTGGAAAGCCGGATTCGTACCTTACCAATCCTGGAATCTTTGGCCGCTCCAGACTTGTCCCCTCCATGTAAATGGCAGTTATCAAAGACCCCAATAGTTATGACAGTACAGGGATCAAAAACTTCCCAAGTGTATTGCTCATTCCATTTGGGCATCAAACTGTCAATAATTGTTCGAGTTCGAACCCACTTCTGGCCATACTTCGCCACACAGTAAGCATCAGTTGTTCCCCGACTATCCTTTGGCTTCATGGGCA

>Unigene892_All

TTTTTTTTTTTTTTTTTTTTTTTACATAAGGTCAGGGAGACTAAGTGCCCTCTCCAGGCAATTTTAAGGACAAAAATAATCCTAGAGTACTCCCCAGCTGAATCCAGTTGGGTATAGATTAGGTAATTTTAAATGCTTATTTTTTACTTACACAATGTTGGGGCTTAAGAGTCCTTTATACTCATCTTCAAGTGCCTGTGGAACATCCCAGTGGAACAGGNNNNNNNNNNNNNNNNNNNAGGGGCTGTATACCATTAGAAAGCAGATCACTGATTAGAGAATTGTAGAACTTGACTCCTTCCCAGTTCACTCCAGTGCTAAGTTTTCCCTCAGGCAATATTCTTGGCCACGAAATCGAGAATCTATAGGAGTCTAAACCAATTTCTTTCAAAATAGAAATGTCTCCCT

>Unigene900_All

GTATCCATTAATGTGTTATTAATATTGGAATTCCAATCAATTCTTGGATTTAAAAATACATAAAGAGCAACACTACAGCTCTTACACCAGTGATTTTACCTTTCCCCAAGCTACATAAGAGCATCCCCGCAAAACTGTCTTAGATTTCCTCTTCATTGGCTGTACATTGCACAGAAGAACTTCAGATCTGTAGCAACGGCACAAGTCAGCCAACANNNNNNNNNNNNNNNNNNGAGCAACCAATCATGGGCATTCTATTTAAAAATTACCCTTGCTTCGTTGCTGTTAAATAATTTCCTGATTCATACAGGGTCTGCAGGAGGAAAAACAGAAATCTGGATGCTCAAACCCACAATTTCATCATGCAGGGGCCAAGTTCCATATAAAGGGGGAGCCAACTGGTTCATCATGGCAATCAGAACAGAACACGATCATCAACACTTCCAACAACAGTTATTGAGGCCTTTGAACCAGACCAGCCACACCCAATTACATCTTTAGCACAAAAAAATCTTCATGGGGATGTTTCAGCCCTCAAATGCTTGCATACAGATTTTCTCCAGCAGCAATGGCCAATCTTCACCAAGCTGCTGTGGATTTAACTCCATTGCAACTTTGAAATCAAGCAAAGATATTGAACACCGAGGCCTCTGTTCATGCATAACTTCCACCGGTCCACTATTGCTCTGCAAATGTAAGTGATTACTCGGCCACAGTCCATTGATCACTTTGCCTTGAACCTGCTGCTTATGGATGGTTTGCTTTCTTGACTTATGCGGAGACCTTGCTCCTACCAACTCAACAGAAGATTTGATCAACTTCTTCAGGTACACATCCAACATATTATTCAGCATATTTGCACATTCCGTTGAAACCCCTCCAACACCCTGCACAGCTGCGATATGCTCCATGCGTTTCCTTAGCATCTCTGTACTTGACAATACACCGCCGTCACTACAGTTGATAAAATCACCACAGCTTGCTGCTGGCATAGCTTTGCGAGCCCCACCCACACTAGCCGAACAAAATGGAATTCCAAGAGGCGCAAGTAATGGACTTCTAGAGAAACTCGAGTGGTTTGCTTGCTCCACCTCTTCCCCATCTTCCACAAAAGCAGTCTGATCTTTGCTATGTGCAGTTGGTTTTTCAGAGGACCATTGAACCAACCCTTCCCTTTCATTCGCAGGTTGCTCAGCAACTGCTTGAAGATGCCCTACTGGTCTTTGATAATCAAATGGAGTCAATTCCCCATTATCCATAATAATTTTACTGCCAACATCATCTGCACCAGTTGGTTGATGTGAGCCACATTCAACTTTCCCAGTTGGCCCAAGTGGGCTAGGTCTATCTCTGGGCTTACGATCACGCATCACTGACCTAATCTTCCGTGGGGACA

>Unigene901_All

GGGGTGATGTTGTGCCCAAGGATGTGAATGCAGCTGTTGCCACCATCAAGACTAAGCGCACAATCCAGTTTGTTGATTGGTGCCCTACTGGATTCAAGTGCGGTATCAACTATCAGCCACCAACTGTTGTTCCGGGAGGCGACCTTGCTAAGGTGCAGAGGGCTGTTTGCATGATTTCCAATTCCACGAGTGTTGCGGAAGTCTTCTCTCGCATTGACCACAAGTTTGATCTCATGTATGCCAAGCGTGCCTTTGTGCATTGGTATGTCGGCGAGGGCATGGAAGAAGGAGAGTTCTCAGAGGCTCGTGAGGATCTTGCTGCCCTGGAGAAGGANNNNNNNNNNNNNNNNNNNCGAAGGGGAGGATGGTGACGAAGGAGATGAGTATTGAAATATTGATTGAAGGATGCTCTTTTTTTTTAATTCTCAATTCCACCCTTTAATTTTGTTTTTTCAGTAGCAAATGGTAGTTTGAGCACAGACATTCATAGCATTCTAACTGACAAGCAAACCATAACAACAACGGCAGCCATATATTATCACACAAAAAGAGCATCCTTCAATCAATATTTCA

>Unigene908_All

CTCTCTCTTTCTCTCTCTCTCACCGTCTCCTCTTCTTCCTCCTTACAACGTCTCCAATGGAGAATAATTTTACATCATTTTTTCCCCTTTCATCTTCTGCAGCAGCAACAACCACGTCACTAAACATGGGCAGTTATCATGGTTTTACAGATTATTTTCAAGCTAAGGAGGAAAATGGATTCTCGGGTTTGATCCAGGAGACTGAAGATCATCATCATCATGCTGTGGTGGGTGGTTTAAATGGTAACAATACCAACAATAATAATAGCAATTTGTCTTCTTCTCAGAATAAAAGCTTTACTAATGGGTCTGAAAATGAAGAGAAATTATTATTAGGGACGAAGAAGAAGGGTTCTGAGAAGAAGATAAGAAAGCCAAGATATGCTTTTCAAACAAGGAGCCAAGTTGATATTTTCTCCATCTGTATCCATCATCAAGAATGTCAATGTCACTAGTGGTTTGAACCACAACTCTAGGTTCTCTCACTGTTCTGCTTCCAGGTGCTGAAATTTCTTCATTTTCACCTTCCGCTTTCCCCGCTTTCCATCTTTTGGCCTCTGGTTCATCTTCATCAAATTCATCGCTCCTGGATTTACTCTTCTGCGAATCGAAATCATCATCCCCTATGGATATTGAAGAATTCTCAGGGGTTGCAACAGAATCCATTTGTCCACTGCCATGGGTGACAAGTGATTGATCTTGCATCTCACTTGATGCAAGATTAGGAGCTATCATTGCTTGATTGGAAGCTGATGAAGAAGAAGAAGATGATCTCCTAGTAGATTGAGGCTTAGGATGGTTATGGCTAACCCTTTAAACTACCATCTCAGTTATTTGTCCATCCAAAGACCTTTCAACTTTCTTCTTTGTTGGGCAATTGGGGTAGGTGCACTTGTAATAACTTCTTGGGTTTTCACTTCCTTTAACTTGTTTTTGTCCATATTTTCTCCAATTGTATCCATCTTCTGATNNNNNNNNNNNNNNNNNNNNAATTGCTGCTGCTGATGACCTCCAGATTCTGACTGGGATCCATTGTTGGTTTGGGTGTTTGTTTGGATAGCAGCAATCTCTGGGGAGAAGCTACGCATACTCTTCCCCATGGAAAAATCATCTTGCTTGACAGATTCTTGGAAACTCCACCCTTGTTGCTGTGCAGTTTGAATTCTGGTGTTTAAAGATTGAAACATTGTACATGAGGTTGTAGGAGGCCTAATCGGTGGCTGGAAAGAGAAATCAGAATAATTTGGGTCTTCATGCTTGACATTTTGCTGGTTTCTACCAGAATTTGTCTCCCAATTTGTAACCTGAGCTGGGAATGTCCCGGTAGTTGGAGATGGCAAAATGTTGGAAGT

>Unigene917_All

ATAAAATACACATATATATACTTTTCACATAAAAGGCCTGGATTTTTCCCTCTTTTATGTCATACATTACAATATTTTTTTATATTTTTTTTCCTCTGTTGATTGGATTCATTACTTTTGATTTTCAACTTATTGTGTTATATATTCATGTGAAAATAAGTATATATATATATGGAATATAGATAGCTCAACTAGAGTGGATATCAGCAATACGTCTCATCACCTGTACTTGGGTATTAAGGCACTTAATGTAATGTGCAGCCTCGTCCAACAAACTGCATAAATCCATAGCTTCACCTCCCGGCACCAGCTTTCTTAGCTTGCTGGCTTGATCCGTACCAACTTCTTGATCATCACTCATCTTCTTCTGTAAACAATAACCCTTTTTCGTNNNNNNNNNNNNNNNNNNNNNNNNNNNNNNNNNNNNNNNNNNNNNNGCATCCCCGCCTCAGTGATCGGTTTCGAATCTTGCAAAGCATTGCCCTACTCCAAACCCTTCTTGACCCAGCAGCGCGAGCTAGGGATTTATCAGCAGCTGTCTTANNNTGACATCGCTGGAAGATCTCTCTAGGACAATATGGGAAAGGTCTTTGCTTGTTTATTTTAATCAAAGATCGAAGGAAGGTTTTAGTGAACCTAGATTTTAAAGAAGAACTAGGGTTTAAAGAAGAGTGGTGATCAGGATTCATGGAGCTTTTCTTTTACAGGGTGAAGTTAGGTTTTTTGTGTAGTTGGGTATTGATGATGATGCAGATATGCATATGATAGTACTTGCAAGTAAACAGTTGGAGATGAGAGATTTTGTTTCTGACACAAAGGGGCTTTGCTCAGAAATCAGATAGGCAAAAGGAAAATTATAATTCTGTCCTCTTTCTCTGACACAGAATCTATAAATCACCAACCAGAAAACAAGAGAGGGAGAGAGAGAGAGAGAGAGATG

>Unigene924_All

GTTGTGTTCGATTTTAGGTGATTTTCACATCAATTATTGTAAGCAAATTAACTAATAGGTTTGATTTGAGGTGGCTGTTTCGGTATTTTTCAGCATCAAATGTAGGAATAGTGGAATTCTGGATCTTAATTTTGCTTTTATGGGAGCTTACTAATTTTTAATTGGGCGAGGGACGGTGGAAAGTGGGTGTATGTGCTTTGANNNNNNNNNNNNNNNNNNNNNNNNNNNNNNNNNNNNNNNNNNNNNNNNNNNNNNNNTTGTCTTAGATGAAGTCTGCAATTTCAAGTTTATATTCTGATTAGTAATGCAGAGGAACCCCGTTTGGGAATGTTTGACAAATCCCTTCCTTGTTTTGGATGTGGAATTGAATGGGTTTCTCTCTTGCTTGGATTTGTGTGCCCTTTGATGTGGTACTTTGCAACAATTCTTTACTTTGGGAGGTACTACCATAAGGATCCAAGGGAACGATCAGAACTAGCTGCCTGTGNNNNNNNNNNNNNNNNNNNNNNNNTGCGCAATAGCTGCAGTGATTGCTCTACTTGTTATTTTGTTGTAGTCCTTGCCACCACAGTTTCATATAAGCAATGGCTACTCTGTGATTGAAAAGATGAAACCGATGCTATGTGGGTAAGACTTTCATTCTTTAGTTCCAATGAAAAGAAGAAAGAGCATGGGTGATACCAAGTTCTAAATATGTGAAGCCTGTAGCATTTACTCAATACCTCTTCAGTCTTGTGCATAAAACAGTGTAGAACTTTTAAGGGAGAAAAAAAATTT

>Unigene967_All

GATCACTTGCAATTTCCATAGTAACTGGGACACCGGAAAGTTCTTCCAGGATGGGTCTAGCAGCTAGAGCAGCATTATAACTTGTACCACAACCAACAAAAACAATTCGACGGCTTCGTCGTATTGTTTTAAGGTGATCCTTGAGTCCACCTAACAGAACAGTCTTAGCTTTGCAGGAACCTCCACGTATAAGCCTCCCCCTCATGGTGGTGGTTAGAGATTCTGGTTGTTCATGAATTTCTTTCTGCATATAGTGCTCATAATTTCCTTTATTTATTTGTTCTACTTCCATCTCAAGAACTGATAGTGCACGCTGGACTGATGAAGGTCTGGAATGGGCACCACTATGTCTTCCCTTATCATTATCAAACTTCAGAATAGACACACTTCCATCCTTAAGATGAACCACTTCACCATCCTCAATCACCAAGACTCTTTTTGTGTGTTCAACAATGGCATTAGCATCACTGGACAGAAAAAGTTCTTTAGAATGGCCATCCATAGAGAGAAATTTAGCATCATGAAATGCTGATCCACTGTTAATATCCTCATTCAGCTCTTTCACACCCAAGAGCAATGGACTTCCTCGTTTGCATGCAATTAACTCGTTTGGATAATGTCTGCTCTTAAAAATAAGAGCATAGGCTCCTTCAAGATGCCTCATGACTTCAAGCACAACTTGACTAAATGTGACAGGCTGAGCTCCTTCACCCTCGTTGGCTTTGTCAAAAACAAATTTGGCAAGCTTTGGAATTACTTCTGTGTCAGTTTCAGATTCAAAGGTGAACCCATGACGAACTAGTGTTTCCTTTAAGACCTCATAATTAGTGACAACGCCATTGTGAACAACCAAGAACTCATTTCCAGCACCTGATGTTTGGGGATGACTATTCCGTGGAGCTGGCTCTCCGTGAGTGGCCCACCGGGTGTGTGCTATCCCAGCATGAATAGAAAAGGATTCTTCCAANNNNNNNNNNNNNNNNNNNNNNNNNNNNNNGGTAGACTGATTTGACGAGGGATTCGATGTTTCCTTCCTGGCGGAAGACGAGAGGGGGAGGTGAAAGAGGGAGAGAATTGAGCAGTGGTTGTGATGATGATGACGATGACGAACATGAACAAGTGATTGGATCGGAAGTAGAATTGTCAATGGAAATACCAGCTGAATCGTATCCGCGGTATTCGAGACGGCGAAGGCCATTGAAAAGGACCTGAAGGATGTACCTTCGCTCTCTGTTCACGTTATAATTCAAATACGCAAATATCCCACACATTATCGCCTTCTTCCTCTTCCTCCTCTTCCTCTTCTTCTTCTTCTTATTCTCCTTCTCTTCTTCTTCTGGCTTCTGGTTATAGGCTGCTAGGAGGAAGAGAGAGGGAAATAACAGAGGAGGTGGGCAAATTTGACGTTTGACTTGTGGATTTGTG

>Unigene982_All

CGATGTCTAAATACCTTCAATTTCAACACTCTGTATTTTAACCTAGAAAAAATAGGTCTCATGTTTTCCTCACCGAAAAGCCAAAGACCCTCAGTTATGGACCATACCCATAAATTGCGTTCGATGAAAGAACATCGACAGAATCTTTCCTCTTGAGAATAAAGCATTCGAACCCCGCNNNNNNNNNNNNNNNNNNNNNNNNNNNNNNNNNNNNNNNNNNNNNNNNNNNNNNNNNAAGCTTACTGTATTTGCCAAACCAATCATCTCTTGTAACATCCACAAAGGGATCATACCCAAAGAATGTATGAGCTGTTCCACAATCTTCTCCCTCCCAATAATTCCTACCATAGCAACCACCAAAATAGCAGTATAGATCGTTGCAGTCACCATGATCATTTCTGAAATGGTATCTGCATTTAACTTGGAAACCAAGGTCGTTTGTGCTCTGGTTGTTAAAAGCAATAACAGCACACAGGGCAAAACCTAAGAACTCACTATTAGCCCAATGTGAAGGCAGCTGGATTGTCATGGACAATCCCCAACTTTGATGGCTGAACCACTTTGGAATACTACTTCCAGGGAAGCAAAAACCAGATTCCCCTGCTCGCACAGAAGGCATCTGATTGTGCAATCTTTTTGAATAAAGCTGAAATTTTGTTAGTGCATATGTCATGATGTTGTGCTTTGCAACAGCATGCAAGTTTCCGCAATTAGTGAAAAGGAATTCAAAAATGTTTCCCTCGACTATAGTCGAATCAATTATTGACACAGATCGGAGTGAGAAACAGTTATCTGCATCTAATTTTGTCAACTGGGGTGGAAGTGCTGTTATTGAACGTAGCCACTTGCAATCCCTCAAACCGAGGTATTGCAGCTCTGTGAGCATTCTGATGCTATCAGGTAGCTGACAAAAAGAG

>Unigene983_All

TTATATAAATTAAATCAAAGAACAAATACAATCTCGCAATCTTAATATTTTTCAACAAATCCAACGACTACTGGTTGTTGCGCCATAACACTCTACTTATACAACACATACAGATGATTACTATCCCTAAATTTCCTAATTTCTAGATCATCTGAGACTCTTAGCGCCATCAGATTTTTTTTTTTTTTCCAAATTTTATGTGTAAATACCCAAACAAACCGTTATCCCTCCCCTTCTGCTCTCTCACATAACCATGAGCCTTGAATATCCCATTGAATGGTGTGGGGATAAAGGTGATAACGGCGGCACACTAAATATCCCCATCACTTCCCCATCCCAAAACCCCTTCCAACTCGACGGAGTTAAGGGATCCGTCGATTGAGCTGCCTTGCCTTCCCTTTCCGGCGAAGGATTTTCCGTCTTTATGACCTTATTATTGCTGCTTTCCAAGCTATCTGTCTCCTTAATCTTCCTTTTCTTGGAAGAACTATGGTTATTATNNNNNNNNNNNNNNNNNNNNNNNNNNNNNNNNNNCGCTGGGTATTATTGGAATCGGAATCCCCAACTTCAAGAGGGAAATTCAGTATTGCCTTGCTTCCACGCAATCTAAAGGCAGCATTATCATAAGCTTTTGCAGCTTCTATGGCGGTATCAAATGTCCCGAGCCATACACGAGCTCCTTTCTTATTAGGATCTCGAATCTCCGCTGCATATTTCCCCCACGGCCTACGCCGGACTCCTCTGTAATGCTTTTCTCCACTAGAATGATCAGTTGCAGCTACTGATACCGCTACCGGCTTCTGCGGTGGGATAGCCTTCAAGGTTGCTGGAGGGGGTATTGATATGTTACTCATGGCTGGGTT

>Unigene995_All

GAATTATATTTTTAAATTAAATGCCATCTGCACTGCAGTTTCTTGGGTAAAAAAAAAAAGGAGCAAAGATATAAACGTGGTGAAATGAAGACACTAGTATATGCACGTGGTGAAGTGAAGACACTAGAACATGTCGTAACCACCATATGCAGAACAAGAGCTGATGAATAGTGCAGACAGTCTCATATTCTACCTCAACAAAAGACACAGAAAAATCAATATGTTGAAGATTCATAGGATTTGATCAGTGTGGTGGGAAATGCTACTGGCTGTATCACTAGCTCACATAGTGGGGTTCTCCCAGACATTTTTTGACAGCCTCATTAGCCTTCTGTGACTCTGCCCGTTCTTCTCGTTTCTTCTTCTCCCTGTCCACATACTCCTTGAGCAACTCTTTTGCTTGAACCAGTTTGGAAAGGAGGTTGCGATACACATCAAGGTCCCGATCCACACTTCTTTTATTTACATTTAAGGCTGCACAAAGCTTTTCCAATATCGTTGGCTCAGTAGCATTTGAAAGCTCAAGAAGACGAAATAGCCCAACGGCAAAGAATCGGCTGTAGCTGAAACTCCCATTTCCAGCCCTCTCTGCAATGTCCTTTAGTATCCCCTCAACTTCTCCTTCTCTGGATGAAAAGTCAACTAATGAAGTGGCAGTCTGAGATCGAGCCCACTCTTCTAGCCTCTTTGCATCAATTCTATATTGCTCGGGATCCTCCTTCAATGCTTTTATGTATGCTTGGAAAATGGCATCTCGATCCTCATCACTTGGGTATCCTTCCATGAGTTGATCATACACGGTAACAAAACCAAGGGCAAACACANNNNNNNNNNNNNNNNNNNNNNNNNNNNNCTCATCAAATGTTGCTGAACAATCAGCTCCTGCAGAACCGTATTGTAGATGCTTGGAATTGGTCTGTTGTACGCCTTAAGAAAATTGAACTTGGTCTCAGATACAGTGGGCACATCTGTAGCAGTGGACATGCAATGAATAACCATGCGAGAAGTAGAATTTGAAGCTCTAACGCCCATATACTGAGAAGAAAAACCAGAACGGAATCTGAAAGTGTCGAAATTGGAAGCGAAAGAACGCGTGGAGGAAGCAAAGGCCTTTCTGTCATTAGAGGATTGGGCAATTGCAGAGAAGGAGACTGAAGTGACTGCTGCCATTTATTCTATAGATCAGAAATGGGAAGAGAAGAGAGGGTGAAAATTTTGCGGGAAAGGAAAATTTTTCTAAATCATAGCGAAAAGGGAGAGAAAATCAAAGGAGAAATTGGCATTGGCATTGGGTTGTTGAAT

>Unigene997_All

CTTGCAATACATATACAGAACAGTATTTGTCGCGACATGGTAAAGGAGTAACAGCATTAGCAGTGTTGAAGTTACTACAATTTGCACAACAAATGCACAGCTCTTCCATCCATCGTTAGTGCCAATACCCAGTGTGATCCCTGAGAAAGAGCTAATGAATAACAAAATACCCACCAAAAACCCAAAAAACAACAATAAAGCGAAAGCCACCCCTCTCATTCCCTTTATGAGAAAACTACTCCGCTTCAATGGCTCAAGACCCCAACTCGATTCCACTGCCACAAGAACACCTACTAGATACCAATTAAGTTGCAGATACACTAAAATCAATATCAAAACAATCGAAACAACTACACAGAACCCGGTAAAATAAGGAGAAAAGAACTCGACTTGAAAACCCGCAAGCTGAATCCCCATAATAAGCAAAAACAAGAAAAACCCAGAAACAAGAANNNNNNNNNNNNNNNNNNNNNNNNNNNNNNNAAATGGTAGTTACTAAAAGGGGAAAGAAAGAAATCAAAGCCGATCTGATCGAGGCTACGAGCTTGACGGGTCTACCATAGAAGCCGTGGAGAACGCTGTAGGTGATAGAACCAACGGCAAGAAGAGCAAAAACAAAGATAAAGAGAGAGAAAAGGAGAGAAAGAACAAGGATTTTGACAGTGACAAGATTAGATGGGTCTTGATCAAGGAGGATGGAGGTAGAGAGGGAGATTTTGGAGTTGGGGGTGGAGGATGATGCGAGGAGGTTCTGGAGGGTGGGGTAAACAGTGAGAGAGAAGGAGAGAGGAAGAAGAAAGAAGACAGAGAGAGCGAGGAAATGGCGGGAGTG

>Unigene1002_All

TTTATAAGTGTGATTGGAGAGAAGAGGTAAATTTAAAGTTGGAAATGGAGAAGAGAGACCCAGAACTCAGATTGTTTGATAGCAGTGAAGAGCTGTCATCAGGACTTGCTGAATACGTTCATCAAATTTCTGAGTCTGCCATAAAAGAGAAAGGTTCATTCTCGCTTGTTCTTTCTGGAGGAGATGTTCCAAAACGCTTGGGGAAGCTAACAAGCTCAGCGTTCTTGAAAATGGTGGAATGGTCGAAATGGCATGTGTTTTGGGCTGAGGAGAACGTGGTTGCTAAGAGACACCCAGATAGCTTCTTCTGGCAAGCCAAAGAATATTTTCTGTCCAAGGTTCCTATACTTCCGGCTCACGTTTTCCCTGTGAGCCACGACGTACCGGGAGAGTAACTACGAGTTCTCCATCAGGCAACACGTTAGAAAACGGTCGGTCTCAGTTTCTCCATCAAGCGACTGCCCCAGATTTGACCTCATCCTTTTAAACTTTCCTCTNNNNNNNNNNNNNNNNNNNNCCCAATGGGTTTCACTTGTCTCAATCGATGGGTCTAAAGAGAAAGTGATGCTCACGCTCCCCGTCATTAATGCTGCAGCCCACGTGGCCATTGTGGCCTCAGGGGCTGAAGTGGCCCCTGAGTTTTTGGATGTGATGATGGGTCAGAAGTCCATTGGGTCCAATCCGGCCCGTATGGTGTGGCCCATAGATGGCAAGCTGGTTTGGTTTGTCGATACCAGCGCTGCTTCTTTGTTTCTCCTTGGCAAAGGATGTGCCGCCACTTCAGGTTCCTAAAATAGACTAGATTGTGAATTCGTTTGACTGATTCCAGAAGCTGGACATTGTGATTATTGTTTTTCACTGCTTGATTATGTTACTGTTACGTTATTATTAAACTTATCTACTAATTAATGAGAATTATTAGCACG

>Unigene1005_All

TTCTTATAATAGTTCACATCCTTCACTTGAGGAACTCCAACAGAAGGTCCTGATGAAGCTGGAACTGCTGTTGGAGGAACCTGTGCATTTGGAAGATACCCTGGTCCAACCCCATTTGGTTGGGCATAAAAGGGTCCACTTGAAGTAGTAGTAAACGATGAAGGAGTGTTGGTCTCTGCTCTATTCATGTGCAAGAAAGACGGTTCTGATAAACTTGCAGGAGGTGGAATGTCTGATGGAGGAACAAAAGATGTTGTTGGCTTGGGGATGTTCTGCACATTGGAGACAGCTCCATAGTCTTGAATCAACTTCTCAATTAATTTTGGGTTGTTGAGAATTTTAATGAGCAGATCNNNNNNNNTAACCTTCCTTGGTCACTGCTTTTATTAATGGCAGCAAATGCAGCCGATGCAGCAGCTGCTACGTCCGGGTTAANNNNNNNNNNNNNNNNNNNNNNNNNNNNNNNNNNNNNNNNNNNNNNNNNNNNNNNNNNNNNNNNNNNNNNNNNTTGAGGAGGTGGAATTCCAGAAGCTAATAGTTGGGACTGTGAGCTCATAGGAACATTAAATGGTCCCATTAAATCAGAAGGTGCATCAGTTGCATCTTCATCTTCAATGGGAGTAATTGGAATAAGAGGAACCTGATGATCACTGTTTCGAGAGTCTTCTACGTCTGCAGAGAAAGTAGGATTTGGAGGAATGGCAGATGGACGAGGATAAACAGCTTCAAGCACTCTCATCTCTCTCTGATTTTGAACCTC

>Unigene1030_All

GCAGTGGATCCTGATTCATACACGAAACCCAAGTAGAAGCCCCCTCCGACCGGAAAGCTTCGACGGAGACTGGCCCTCTGAACAGTCTCTTCCAAACCCACCACTAATCAGACGAAGAGAGGCAGAGAGAGCGGGAGGGGGAGATGGGGGAGGAGAAGAGACACCAGATGATGCAGAATCTGTTCGGCGATCAGTCTGAGGAAGAAGAAGAGATCGATTCTGAGCATGAATCGAATCCCCACTCTAATTACGCCTCTGATGAAGCTGAAGGAGGTCTGAGGAATGAGGGTGAAGATGAAGGTGAAGGTGAAGATGAAGTGGAAGGGCACGGTAATATAGAAGTGGAGAGTGAAGATGAAATGCATGAGGTAGAACCTGATCCAGGAGAAAGTGAGGGTGAAAGAGAACCAAGTTCTGAAGAAGTAGATGTTGTTGATGAGAGGGAAGTGAGTGAAGCTAAAGATGCGGATAGCGAAGAGAAAGAAGACTATGGTCACAGATTGGCAACAAGCAGGAGACGTGATATAATTGAAAGTGGATCAGAGAGATCTGAGGAACAACATTATGCTGACCACGAGGATGAGGAGGTTGACCAGGCCAGAAGCCCAAGTAAATCACCTGATGGGGAGAAGGATCACAATCCCATTTCACAGTCAGCTGCTGAGATTCGTGATGTGTTTGGTGATTCTGATGATGAGGAAGAGGCAGGATATGTGGTTAGGAATGAAATTGAGCACGATTCACATAGGTCTCCAATGGAAGAGGAAGGTAGCTATGAAAAGAATTTAAGACCAGAGGATATGCTGATGGACGAAGATGCTCGATATNNNNNNNNNNNNNNNNNNNNNNNNAGAAACCAGTTGGCCCCCCATTAGAGATAGAGATTCCATTTCGGCAGCCTCCAGCTGATCCGACCAAGATGAATATGATCAAGGTTTCCAATATAATGGGCATTGACCCAAATCCATTTGATCCTAAGACATATGTGGAAGAGAAAACATTTGTGACAGATGAATCTGGAGCCAAAACACGTATACGCTTGGAAAATAATATTGTCCGGTGGCGGACTGTTAGAAATCCTGATGGCAGTAAATCTGTTGAAAGCAATGCTCGGTTTGTGAGATGGTCAGATGGCAGTTTACAGTTATTAATTGGGAATGAAGTTCTTGATATATCTGTGCAAGATGATCAGCATGACCAAACACATCTTTTTCTTAGACATAACAAGTCACTTCTTCAATCACAAGGAAGAATACGGAGGAAGATGCGGTTTATGCCATCATCTTTGACATCAAATTCTCACAGGCTGTTGACGGCTCTTGTTGACTCACGGCATAGAAAGGTTTATAAAGTTAAGAACTGCATCACCGACATTGATCCTGAGAGAGAAAAAGAGGAAAAAGAAAGGGCTGAAAGTCAAACAATTAGAGCCAATGTACTTCTTAATCGGA

>Unigene1035_All

CGTTGCTTCTCTCTTTCTGATTCGAAAAATTCTTCTGCAGCTCGCAGCTTTATTTTTTTAGCGATTCTCCTTAGGAGCGATCAGATCAAAAATGGCTGAGCATTTGGCGTCGATTTTTGGGACGGAGAAGGACAGGGTGAATTGCCCCTTTTACTTCAAGATCGGTGCATGCAGGCATGGAGACCGATGCTCCAGACTCCATAACAAGCCAACCATTAGCCCTACGCTTTTGCTTTCGAATATGTATCAGCGGCCTGACATGATTACTCCTGGCGTTGATGCTCAGGGCAACCCCATCGATCCCCGCAAGATCCAGGAGCACTTCGAGGATTTCTATGATGATCTATTTGAGGAACTAAGCAAATACGGTGAAATTGAGAGCCTGAATGTCTGTGACAACCTTGCCGACCACATGGTGGGGAATGTGTATGTTCAGTTTAGAGAGGATGAGGATGCTGCAAAAGCACTTCAGAGCCTCAGCGGGAGATTTTATGCTGGTCGTCCCATCATTGTTGATTTCTCCCCGGTGACGGACTTCCGTGAAGCCACCTGCAGGCAATATGAAGAGAACTCGTGCAACCGTGGTGGCTATTGCAATTTCATGCATCTCAAAAGGATCAGCAGGGAGTTGAGGCGTCAATTATTTGGGATGTACCGACAAAGGTCTAGTCGCAGCTGAAGCAGAAGCCCTTATAGGTATCGCAGCCGAAGCAGAAGCCCTTATAGGCATCGTAGTCATGAACAGTACTATTATGGTGGTCGTGGTTCTGGTAGAAGGTATGATGACCGGGATTGTTATTATGAAAGTTGGAGTAGAAGGCACAGGAGTATAAGCCCTGACCACCATAGAGGACGAAGTAGAAGCCCTGGGAGAAGGCGTGATAGAAGCCCTGTTAGAAAAGGCAGTGAGGAGAGGCGTGCTAAAATTGCCCAATGGAACAGGGAAAGGGAACAACAACAGGAAAATGCAAATAAGGTCAAAACTGATGGTGGCTANNNNNNNNNNNNNNNNNNNNNNNNNNNNNNNNNNNNNNNNNNNNNNNNNNNNNTCTACCGCAGCAAGGAGTATATTGATATTGATCCAGTTTAGATGTCTGGTTGTTGGTTGGG

>Unigene1059_All

GGAGTCTAATCAGCGTGGGGAGTCTGTTTTACCTCAACTAGACAAGGATACTGCTGCAGTTAGGCCTCAGTCTCACCATGGTCAGTCGCTGTCTGAAAAGAACAATTTGGAAGAAGCTCCAGCTGTTGGGCATCAAGAGCCTAAGAGAGAAAGGAAGATAGCTGCACAGAGAGGACGTCCTGGTAGCCCTGTTGAATCATCATCCCCTAATATGGACATTCGGCACGACAAACGCATGTCTTCAGGGTTCCGCAAAAATGGAAATCAGAATAGCCGATTTAGTAGAGACCATGAATCTCGTGGAGATAGGAGTGGATCTGGGAAAGATAGCAAGCAACATAATGTACCTGCAGTCCGGGAGAGAGACAGACATAATTCACATTACGAGTACCATCCAGTTGGGCCCCACAACAGTGGCAAAGCAAACAACTTGGAAGTTCCCAAAGATGTTTCCCATAACTACTCTGGGACAGGGTATAGAGAGAGGGGCCAGGGTCACTCGAGGCATGGTGCGGGCAACTTTTATGGACGGCAAACTGGCAGTGTTCAAGTAGATGCTGGTCACGACTAAGGAGAAATTCAGAGAGGGGTTTTTTGGTAGTGCCTAAAACCAGCTTCCAAAGATATTTGTGGCTCCATCTCTAACACTTTCNNNNNNNNNNNNNNNNNNNNNNNNNNNNNNNNNNNNNNNNNNNNTCATCCCAACTGATATAATATGCATTGAGCAGGGCTTGGAGTATGCAGAGGGTGTCAANTGGGAGTTGCTTGATATGGTTGATTTCTCTTCATTTTTTCTAAATGTATCTGATCTGATAGGGTTTTAATTTGGTCACAGTATGCATGCTGTGGAACTCTCAATGTGTTTTGAGCAGTGTATTCAGTTATCTTTATTTCTTTGCTCTTAGTATGGAACGCTAGATAGAATTTAGTGGAGATGGGAGCTTTTCTGGTTCTCCTGTTTAATTTCAACCTTCTTTTTTTGTTTTCGTTTGGCTCTGTTAACATTGCAGGATATTCCTCTCTTCCCCTTGCATGCATTGAAAGTTTGATTTTCCGGTGAAAAGTGTGCACTAATGTAAATATCTTCG

>Unigene1082_All

GTATACTTACGAAATACATCAGGAATTCAAGAGCTCACAATTCTAGAGAGGATTACAATATTATTGAAATAAAGTTCATTAGTACTCATATAACATTAATGATGCTTCACTAGGCCTTTGTTATTTCAAATCAAACTTAAACATGGCAATTTCAGTTTGCGACAACATATCAGAATCATCGGTGAGGCCATGTCTCTCTCCAATAGCTCTTAATGCTCGATACACCTGAAGCATTGTTGGCCTTCGATCTGGAAAATCTTGAACACAGTCACATGCAATTCTCAGGACCTGAAAGATCTCCCCATCGTTTCCTTGCCCAACAATACATTTCTCAATAGCATCATAGAACCCAGAAGAACTTGTCGAGAGATCAGTGGTCAATTCTTTTGGAGTCTTATTAAATATGCTGAAAGATTTTATGATCCTGCAAGGATCTTTCCCTGTGATTAGCTCAAGAAGAATCATCCCAAAGCTGTGAACATCCTTCTTAACAAAGCCAATATCCCAGAATTCTCTATTCACAATGAAGTCCCTTGTTGATTGAGTTTCATTTGGATTGATGAACAGTGCTCCACCAAAATTCGATAATTTGGGCTCAAATTTCTGATCAAGTAAGATGCATTTAGAGTTTATGTTAAGATGGACTGCCTGAACACTGGGGTTGTAATGAATCCAAGCCAATCCTCTTGCAAGCCCAATTGCCACTTTAACCCTTAAAGTCCAATCAATAGCATTGGCATGGTGTAGCCAATCAAAGAGGGTTCCATTTGAAATGTATTTGTACACCAGAAGCATTTCCTTCTTGTCTTTGCAGAAACCTAGTAGGGGCACTAAGTTACTGTGTCTCAACCTACCCAGAGTCATGACCTCAGTGACAAATTCTTTCTTGAAATGTTGGGAGTTATGCAGCCTCTTAATAGCAAGAAACCAACCATTTGGAAGTGTTGCCTTGTACATTGTTCCCATATTTCCTAAACCGATGACATTCTCTTTGCTGAAGTTTGAAGTTACCTCACAAATTTCTTTGAAACTCATTCTGGTAACCAGCCTTTCCAGTGTAGACATCTTTATATCCATCCTTCTTCCATCAATTGGTTGTCTTCCCTTTTTGATTTTCTTCTCCATCGATTTCTTCTTGGACCTTTTGGACATAGAAAACCAAAAANNNNNNNNNNACCAAAGCTATAGCAACCATATAACCAACGACAAACCCTGTGATGAAGGAAAAAGAGAACTCATTTGGAGGAGAGCGAAGGCAAGGATCCAATGGTCGTCCACAGAGTCCTGAATTATNNNNNNNNNNNNNNNNNNNNNNNNTAAAGTTAGGTACCGGCCCAGACAAGAGATTGTTAGCGACACTAAAGCTCTTGATTCGATAAAGCAAGCTAATTTGTGCTGGGATTTGCCCTGTTAGCTGGTTGTTGTCGAGTCTGAGGACATTAAGATAAGAACAGTTTGCAAAACCAGAAGGGATTTCACCAGATATACTGTTGTTGGAGAGATCAAGTACAGATATATATGGGATTTTGTTGCATATATCAGATGGGATTGTGCCTGAGAGTTGATTGCCTGAAAGATTTAAACCTGTTATGCTGGTACAGTTCTCTATGCCCCTAGGAAAGTTGCCTCTGAGCCCCAAGTTCACCAAATGGAGATTTAAGACTCTATTCTCATCCTCGTGCCAGCAATCAACTCCTTTAAAATAACAGAGGAAATCTCCAGTATTGTTGTTCAAGTTCCATGAATACTTCCAGCTATTTCGAGGATCTTCAAAAGAATCTTTAATAGACTTCAGGCAAGCAATATCTGTCTCAG

>Unigene1084_All

CATTTTTCAAGTAACATGGAACATATTTATAACAGAAAATAACAAAATTCCATTAAACAATCAGGTTGCTTTTTTTCTTTCCCCCAACAAATTTGATTGTTCACACAGAATTCAAATTTGTCTCATGTAAATTATTGACCAAAGAAACTAGCATTTCAACAATCTAATCTAATCGAAAAGCTTTGAGGAGCTATCCTTTCACCTTAAAAGACCCGAGTAGCAGCAGCACAACAGCAGGTCCCTATGATGGTTAAGGAATACATAAATTAATTTAGAAGCAAATTGGGTAACCACCTTTCGTGTAATAGCCTTGAAATCAGAGAAAGTGTGTTCAAACCACATGGCCGAGTTAAGTAGAAAGCTAGCAGAGTCGTCTTAAAGCCGCTGCCGTTTTGTGACACTACCATGCTGCCGACAATGGTGATGGTTTTGCACAGTCGAGCAACATATATCAGGCACAAATTCCCCATCATCAGATTGATTATGAACGCGTTTTGGCCAATATAGCCCCGAGGCTGTAGGTGGAGAAGGGATCTGTGGCACTGCCCAGGAGCTGTCAGGCACCTGACCCAGGCAATCATCATCATCATCTACTTCTTCCTCGGCTGATCTTAAGATTGCAGAGCTGTCAGAGTCACTACGCTTGCCGCTATCAGCCTTAGATGATCCATTATTCATGTAGCTATAGTTTTGACTAAAACCAGTTAAGCCAAGAAATTCATCCATCTGNNNNNNNNNNNNNNNNNNNNNNNNNCACCAGAGAATGATACTTTAACAGGTTCAAACTCCTGTCCAACAACCTGTGCAGGCAAAACTTCATTGCTCGGACTAGCCAATGGCATTGGAGTTCCTCTTCTAGAAACATAATGGGATTTTGTTTCAGATGTTTTTTCACCCGAAGGTGACTTCCCCGAAGAAGAGGATGCACCAGGGTCAGTAGGTTCTAGACCAACTTTTACTCCGGTTAAAAGAAATCTCTGATGAGCAGACACATAGGCATTTGCAGTGTGAATAGCAACATCAC

>Unigene1088_All

TGTGCCTGTGAAGAACCGGAAGAACAGGAAGAAAAAGTTGAAGAACAAGAAGAAGATACGGGTGGTGAGTGATAACAAGTACATTTCAGGTCATGAATGGCTTGTTTCCTCGTGATGTCGAGTTTTGTACAGAAGACACAGTACCGTCCTCCAAATATATCTTCATCTTCATCACTATCGTCAACATCTGTGGTATCTTCTTCAAAAGTTTCCCCAGGTTCAATTCTCCAATCATCACTTGTGTAGTATATCAATCCCAACATTGGAAGAAGCACCATAAGAATTTTATCTATACGCTTGTCAAAGCATTCATTTGTCCTCTTCCAGAACATTAACGGCGCAACAACAGCTCCTGCCCCTACCCCAAAACCCAGTCCAGGGACTATGAACTGCCAATCAAATTCATTTCTTGGGTTTTGTTGCCTTGCAGTCGGTAATGCACTGGTATCTAGACATTTTTCTTCCAAAGGAGCTCCACATAATCCTTCATTATTTGCAAAGGAAGCTGCTGAAAATGATTGGAGTTGAGTACTTGTAGGAATCCTTCCGACTAGCATGTTGTTTGAGAGGTTCAGGACTGAAAGAAATGTTAGCTTTGCAAGCTGCTGAGGAATTTCTCCAGTAAGTTTGTTATCTGAAAGGTCTAAGGACTCCAGATGTAAGAGGTTGCCTAAAGCTGATGGGATTTGACCAACAAGTGCATTATGTGACAAGTTGAGAGCATAAAGTGCATTTAATTGCCCTAACCTTTCTGGTATTGGCCCTTCAAATTTGTTGCCAGAGACATCAATGGTTGTGAAGACTGTTAGGATCTTCACCAAATTCATTTCAAGGCCTTTGCTGGTAACTGTTATTGAATCTTGATAATACAGTTGACCAAGCCGTAGGACTGTGAATTTCAGGTGATCATGGGTTTCATTTCCATCTCCCATCATTGCCTTCCATTTAGATAAGACACTATTTGGCAGTCTACCACTAAAATTGTNNNNNNNNNNNNNNNNNNNNNNNNNTGAAGTTTTGTCCATTTGACATCGATCTGTGGACATAATAAGTTCCCATAAAAATTGTTATTTCGCAGAACAAGAACTCGCAAACTGGACATGTTCCTCAAAAGGCATGGGAAAGCATCATTGAATTTATTGCTTCCAAGGTCTAAAACCTCTAACGTGGTGCAAT

>Unigene1108_All

ACTTCTCTTCGAAGAGATTCAATTGGAGAATACTTGGCAGATGACGCTTGGGACTTCAAAGATCTGGAAGAGGCAGTAACAAAAAATTATTTCTAACTGGTTGTGGCAATAAGTTGCCCCTAAAAATTTTGGGGCTCAGTACATTTTTGTTGGTGGTTACATCATTCCCTCATTTCATTCATTACAAACTATAAAAATTTGATACAGTGATAGGTAGCTAAAGGCTTTTGGTACTCTCTCTCTCTGCAGATTAGTTCTATGCAAATTTTTGATAGCCAATTCCCAGAATTTTCAAGGGCTGTACTTTGCTAAAGTGAAACCGTAGATTCTATTCTAGAAATGAAATGGCGCATAGAAATTCTAATGTGTACGTTCGAGTAGCTGACTTACGAGCCATCATGGGGAAATAACCCTTCTGTTCTTGCATTACCAAGATCATGATTTTAAACTTCTGAACCTTTCTTGCCCGAGTCATCAGAATACATAAATTTGTCTAGTTAATTTGCTCAAGGCTACAGGTCAGCTCTTCCATTACACCTAAATAGTAAATGTAACTCCATAAGGTTGTTCACAATTACAGGTGATATGAACTATGGGGATTGATTCCCAGAACAAAGACACTTAGAAGGCCACAAAAAATTACGTTAATGAAGACAGAAAATGAATGAAGCATTGCAATTTTCAGTGAAAGAACTGGTAAATATGAAACAGGGATATATGCTTACTAAACCAGCCACGACACATTATCATTGTGGAAACTGCATAAACATGGATGTGAGTTCTGCAAGTAGATTAGATGTGGTTATTTTATAAAATATTCTTGTAGCCATCACTGCCATCCCTCTAACGAGTTGTTGAATTGGCAGGATCAATTCCCATTGCCTGAAAGTAGGCTTTCTCTGCATTAGTCATTCGATTTTGAAACATATCCCATTCTTTTCGACATCTCTCCCTCACCCCTTCCCATGGTGCCCTACCACTCTCTGCTTTCCCCTGGTTCCCAAGTGAAGGAACACCTTGATGAACAATCCATTGAGCATCTACGACTCCTATTTTCTCATGAGCAGGCTCAACACATTTCCTCAGAGCAAAATCTAAACCCCATCCATGAATCAAGTCATTCTGAATCATATGCCAAACACAACGCCATGCATCCCGAGAAAACACAGTCGCCATAATCTCAACAAATGCTGCACAAGGTGGCAAGTGCGGGTCAGTACACCAACCAGGCTTCTCTTCAGTATCCTTGTGAACTTCAGAGTCATCTCTCTTCTTTGTCATTGCCCATGTTGTCCCTCTATCAGGATCTAAACCAGGCTGTGAAATCTCCAGACCATGTTTCCTTACCAGTTTAATGTATTCCTCTGCATCAAAATGCTCTACCCCAAGATCCTCATCCCACATAAAAATATAGTCATAGGGTGCAACAATGTCAGGATGTAGAAAACGTTTGGCATACCACCACTTTGTTTGCTTCGAAGCACTCACGTGGATGGCTCGCTTTGACCACTCAAACTCATCCCATTCTGTTGTCCGACCATCATAATGAAATAACAGAATGGTGAAGTTATCTGAGAACTTTCTAATTGCTGCATCAATATTATTTTTCTGATCATAACCAACTGTAAAAGTAACCAGATACTTTGGTTTGGTGGCCAAGTCNNNNNNNNNNNNNNNNNACCCCACAATCGACGGAGGTAGAAATCTGATTCAGACTCTATTATACCAGGAGGTAATCTCTCAGCTCCTCGAGGATTTGTTGGAACCCATATCTTTGTATCATTATATATGTGAAGTGGAGAGTTGGCTTTGTTACCCTTTAGAGATGACCAAGCATTGAACAGTGCTTGGGTTGAAAGACCAGAGTACTTGTCCTCAATATACGTGAGATCAATGGAAGGAAATAGGCTGGATGGAAGATTGATCTTAGATAGTGAGAGTGTTGGAAATGATATTCCTAAAAAGAAGCCAAAAATAATTCCAACAAAAGTTGTCATGAAAAGCCTCATCATCTCGTTAGGTTTTCTTGTATATCCACTACGGATGGGAAGACGCATTATAGTCCAAATCTTCTGAGTCTACAACCAAAAATATACAAGATGAAACCCAGCAAGAAATAGCTGTTCCACTCACTCATCAAGTCTGCATTTTTCAGTAGCCTCCATATGAAATGAACTCCTTCATGGGAATATGACCAACTCTGAAATGCTTTTAGATAACAGTCCAGAGAACTGAGGCAATCTCG

>Unigene1121_All

CTTTCCCTATTGAGTTCCCTTCTTTTGCCCACAGCAACTGTTTCATTCGCACACATTGGAAAACTTTCCTCACACTCAGTTCTCCAAAATGAAGATATGGGGACAAAAGTGAGCTGGAGTTCACACCAACCTTTAGCCTATTCTTTGAGTAGTCAATTAGATGATGTTCAACAAATTCAGCCAGTGCCTTGTCAGCATTGCTCCAACCTGGTGACCATCCCCTTCCTAGTAAGGAATTGCTAGATTTTTCTGTTTCATCTTCAAGATTTAATTCCTCAACTGAGCATTTCTCAACTTTTCCTGCAGTTGCCAGTAATCGCCATGGAGGAAGATGTGAAACAGGTTCCATTTGCATGTGCAAGCACTTGTCCCAATACGAGTCAAATGTCGTAAAAGCTTGTCCACTTCCATTATATATTTCCCATGGTTCATACAACAAATCCCCATTATAGCTTTGCACAGAAATGCCAAGCTCCACCAGTTTTTCTTTGATGCTGTGATCACGAACAAGAGATACTGGATCATAGAGATGATTGAATACAACTCTTGTTGCTCCAATAGCATTGATGCAATCCAAAAGAGCAGCGAGAGTACTATGGGTTTTGATCAAGACAAGTTCAGCCCCAAGAGATTTCAGGGAGTGTCCCAAGTGGGCAAGAGACTGCTTCAGCCACCACCTCGATACTCTACCTGGGTAGAATTGTCCTTCTTCTTGAGGACACCATATGAAGATCGGAAATACACTACCATCCCTGGCTGAAGCAGCCAATGCAGGATTGTCCTCAATTCTAAGATCCCTCCTAAACCAAACAATGGTCTTATTGCTGCCCATATTGTAATAACTCAGATCTTTGAGGATCACACTGGAGTTCAAATCTTATGATGNNNNNNNNNNNNNNNNNNNNNNNNNNNNNNNNNNNNNNNNNNNNNNNNNNNNNNNNNNNNNNNNNNNNNNNNNNNNNNNNNNNNNNNACTTAATCAAATAAATAAATGCTGTCAGTTTCATGAAGCAACTCACTTCAAAAACAAGGGACCCATGGAGCAGAAAACCTGCGAATATCTAGAATGGCAACAGGAAGCTCCAGCCCAAGACAACAGGACCATAAATTAGGAATAACCAGTTCAGTTAATAATAGTGAAATTTGGCTGCTATCCCCA

>Unigene1125_All

TTTTTTTAAAATGGAGCAATCTTGCTGTTCCTTAACCATAACATATGGCACATTACAACGAAAACAAACGATACTTCGAGGGGAGAAAAAACAAAAGAACATGACAAAGATAACATCGTATCATTGCTGCAAGCCTAGTATATATGTACATATAGAAGGCGGAGACGGAGAAGCTGAAGGCTAAACCTATCTACATATGTCGTATGGACAGAAAGGAAGCGATTAAAAAGTTCTCTAAAGAGCAATAGCTTCTGATGAGTTGAGGCGTCCATTAGCACCAGCGAAACATCTGAAGAAACGCTTCTCGTTGTCTTCTTCTTCATCTTCACCCTCATTCTCATTCTCCTCTAATGATCTTCCCATGGTTTCGGGTGTGAATACATATGTCACCACCATTCCCAGAAGACAAACCGCTCCCAAAGCCACCAATGCAATTGTCATTGATCTATCGTAATCTTTTTTATTTGAAGCCCACAAAACCCCAAGAGACCCTATCAAAGCACCAATCTTCCCCATGGCCCCGGAAATCCCATGACATGTTGATCTAAATCTAGCTGGAAAAAGTTCGGCAGGGACTATAAAGGTGGTAGTGTTTGGTCCAAAATTGGCAAAGAAGAAAGTGAGTGCATATAGGAACAAGAAACCTCCGTTAGTGTTCTGACCCCAATAATGGTAAGGTATCCCAAGGGCAAAATAAACTATACCCATGAAGAGAAAGCCCATGATTTGGATTTTCCTTCTTCCAACACGATCGATGAAATAGACAGTGAAGTAATACCCAGGAATGGTGGAGCAGACTGCGACGATGGCTTGGAAGCGGGCGATTTTGAAAGCTGCTTTATAGACGTTGTCCTTATCATTTAGTTTAAGGAACTGGCTGTAAATCTGTGACTGGAAGAGGTTACTGCTGTAGAAGACTATGTCAAGGAGAAACCAATTGAGAGAACAAGAGAAGAGATCACGGCCGTGGCGGCGAAAGAATTTTCTGGAGAGAAGGGGATAGGTTGGTCGATCTTGTGGCCATGGCTCGTCTTCTGCTATTTGGCTCATTGAAACGTCTAATACCTTCTCCATGTCCTTTGCTGCTTGTAGGACATTATTCTCCACGAGGGCTGTGTATCTGGCAGTTTCAGGCATCATCATACGCCANTAACCCAACTAACAAGAAATTCTGAAACGAATAGATTAACATACCTGGCAGTTTCAGGCATCATCATACGCCAATAATAAGTGAGAGCCGCCGGAATTGCACCCAGCATCAGTATCAACCTCCATGAAATATCGGCGTCCTCAGGCGTATGATCCTGGTTCAAATTCCTTGATGCTATCCAGAATATCTTGCACACCACCATGGTCACCGCTGAGCTCGCCAAGATCCCAAATCCTTGCATCGAAAACACTCCCGCAATGAAAGACCCTCGAGTCTTCCTGTTCGCGAACTCCGACATGATTGTCGCCGATAACGGATAGTCCCCTCCGATCCCCAGACCCAGTAAGAATCTGAAAAATCCCAAACTCAACAGAACGCAGTTACGGGTCCTGCATATCGAAAACCCACATCCCGTAGAACTCAACACCATCAGCATCAATGCGACTCCATAAACGCTGCGCCTTCCCTTTCGGTCACCTAGTCTACCGAACACCACCTGACCTATTGCGGTGCCGAGAAGGGTGGTGCTGACCATTAAAGCGACTACACCGGTTGGGATTTCGTATTTGTTGGGTTCAGGCCTGTCCTCGTAGTACATCCTTCCTATAAGTTTCATAATTGGAGGGATGCAGAAGAAATCGTAGGCGTCAGTGAAGAGGCCCATCCCTGCTACNNNNNNNNNNNNNNNAGTTCTTGCAGAGTCAAGGGCAGAAAGGACTTTTAACGGCATTGTTGCTGCTGATGTTAAGAAGTTGGAAAGTGGAAAGGAAATGAAAATG

>Unigene1176_All

TTCTCAAGCCCACTAATGCCGGTATTCATGATATATCCAGATCGTAAATCAGCGTTTGGGCTTGGGCCATTTCCTTCACACCACACATTATTTGAACCCATTCTGTTTAAGAAATCTTTCAGTACCATCATGGATTCAGCATCACACAGCTTACCAACAACTCCAACAATTTCCTCTGGTTTAACTTGATGTAAAACCTCTGCAACTACTGCTAAGGCATCTCGCCAACTTACAGCCTTGAAGCGCCCATCAGCACCACGAATCATAGGATCATTTAGCCTCTGCCTCTTCAAACCATCATAAAAAAACCGAGTTTTATCTGATATCCATTCTTCATTTATGTCCTCATTTAATCGTGGAAGGATGCGCATGACCTCTGGACCTCTACTATCAATACGAATGTTGGATCCAACTGCATCAGTAACATCAATGCTCTCTGTCCCTTTCAACTCCCAGTTTCGGGCTTTAAATGCGAAAGGTTTTGATGTAAGAGCCCCAACAGGACAGATATCTATCACATTTCCAGAAAGTTCACTTGTCATAAGCTTTTCAACGTAAGTGCCAATTTCTTCTCCACTGCCACGACCTAGCATGCCGAGATCCTGGACCCCAGCAATTTCTGTAGCAAACCTGACGCACCTTGTGCACTGAATACACCGAGTCATCACTGTCTTCACCAAAGGACCAAGATTCTTATCAACCACAGATCGCTTCATTTCAGTGAACCGGCCACGATCAGATCCAAATGCCATAGACTGATCTTGAAGATCACACTCTCCACCTTGATCACAAATTGGACAATCCAATGGATGANNNNNNNNNNNNNNNNNNNCCTTCTCGTGCCTTCTTTGCTACAGGTGTGTCGGTCTTAATCTTCATCCCAGGAAGAGCAGGCATGGCGCAAGACGCCACAGGCTTGGGGGGCTTCTCGACCTCAACCA

>Unigene1207_All

CCGATTTGACGACCGCTATCAACGGCGGCGGCTCTGGAGATACTCAAAAAGAATCACGCCCGACCACCACCGCCCAAGCGGGCGGGGGTGGCGGCGGCCACTGAAGGAGATGAGCATGCTATCGCGAAGAGGATTTCTCGGAACGGCGGCGCTGGTCAGCGCCAGCGCCGTTAGCGGCCGCGTGCAGGCGGCCAGCATCCCGGAAGCACCGATCATGGACAAGGCGACGATGCAGCCACCGTTGCATCCCACCTCCGGTCCGGACTATCGGCCGGTGGTGACGCTGAACGGNNNNNNNNNNNNNNNNCGACTGGAAGGAATTTCATCTGGTCGCCGAACCCGTGGTGCGCGAATTTGCCGAAGGCATGAAGGCTCATCTCTGGGGCTACAACGGCCAATCTCCGGGTCCCACCATTGAGGCGGGAGAAGGCGATAANNNNNNNNNNNNNNCAACAAATTGCCGGAACATACCACCGTGCATTGGCATGGCGTGCTCTTGCCGAGCGGCATGGACGGGGTCGGCGGGCTCAACCAGCCCCACATCAAACCTGGCGAGACCTTCGTCTACGAGTTCGAGATGAAGCATAGCGGGACGTTCATGTACCACCCGCACTCCGACGAAATGGTGCAGATGGCGATGGGCATGATGGGCATGATTGTGGTGCATCCACGTGATCCGTCATTCCGCCCGGTCGACCGCGATTTCGTCTTCATCATGAGCACCTACCTGATCGACCCCGGCACCTACCTGCCGAAGGTCAACGAGATGACCGACTTCAACATGTGGACCTGGAACAGCCGGGTGTTTCCCGGGATCGATCCGCTGCCGGTGCGCCTCAACGATCGCGTGCGCGTGCGGATCGGAAATCTCACCATGACCAATCATCCCATCCATCTTCACGGACATAAGTTCGTCGTGAGCTGCACCGATGGCGGCTGGGTTCCGGAAAGCGCCCAATGGCCCGAGACGAC

>Unigene1230_All

TGTTGTTTAAAGGCCATGAATATGGTACTGCTTTGATGAAATACTCTTATGTGGTTGCCTGCCAGATATACGGGACTCAAAAAGCAAAGAAGGATCCCCATGCTGAAGAAATATTGTATTTGATGAAAACTAATGAGGCCCTTCGAGTTGCCTATGTAGATGAGGTGAACACAGGGAGAGATGAGAAGGAGTATTACTCTGTGCTTGTGAAATATGATCAGCAGTTGGACAAGGAAGTTGAAATTTACAGGATCAAGTTGCCAGGCCCCTTGAAACTTGGGGAAGGAAAACCAGAGAATCAAAATCATGCTCTCATCTTTACTCGTGGCGATGCAGTGCAGACTATTGATATGAACCAGGACAACTATTTCGAGGAGGCGCTCAAAATGAGAAATCTTTTGGAAGAATACAGGCGCTACTATGGAATTCGGAAGCCTACTATCTTGGGAGTCAGGGAACACATTTTTACTGGTTCTGTCTCATCGCTGGCATGGTTTATGTCAGCCCAGGAAATGAGTTTTGTCACCTTGGGACAGCGTGTTTTGGCAAACCCTTTGAAAGTTCGAATGCATTATGGCCATCCAGATGTCTTTGACAGGTTTTGGTTCTTGACTCGTGGTGGCATCAGTAAAGCTTCTAGGGTGATTAACATTAGCGAAGACATTTTTGCTGGCTTTAACTGCACTTTGCGAGGAGGCAATGTCACACACCAGGAATATATACAAGTTGGTAAGGGAAGGGATGTCGGGTTCAATCAAGTTTCCATGTTTGAAGCCAAGGTTGCCAGTGGAAATGGCGAGCAGGTTCTTAGCAGAGATGTCTATAGGTTGGGCCATAGATTGGATTTCTTCCGAATGCTATCCTTCTTTTATACTACTGTGGGATTTTATTTCAACACAATGATGGTCGTTCTGACAGTATATGCATTTTTATGGGGCCGGCTCTATTTGGCTCTTAGTGGTGTTGAGGCTTCTGCTATGGCCAGTAACAGCAGCAATAATAAAGCACTTGGTGCTATTTTGAATCAGCAATTCATCATCCAACTTGGCCTTTTCACTGCACTTCCGATGATAGTAGAGAACTCTCTTGAGCATGGATTCCTCCAAGCTATCTGGGATTTCTTGACAATGCAACTTCAGCTCTCATCTTTTTTCTACACTTTCTCCATGGGAACTAAAACTCACTTCTTTGGTCGAACTATCCTTCATGGAGGTGCAAAATATCGGGCTACTGGTCGTGGTTTTGTTGTGCAGCACAAGGGTTTTGCAGAGAATTATAGACTCTACGCTCGTAGTCATTTTGTGAAGGCGATTGAGCTTGGGCTGATACTCACAGTTTATGCTTCACACAGTGCTATAGCTAAGGACACTTTCGTTTACATAGCCATGACCATCTTTAGTTGGTTCCTGGTTGTNNNNNNNNNNNNNNNNNNNNNNNNNNNNNNNNNNNNNNNNNNNTGGTTGAAGACAGTTTATGATTTTGATGATTTTATGAACTGGATTTGGTACAAAGGTGGTGC

>Unigene1265_All

ATCGTCTTCATCGTCGAGCAAATGGAGGTATTGTAATCCTTCGTATTACCTGAAGAGACCGAAACGCTTGGCTTTGCTTTTCATAGCCTTTGTTTGTGCCACTTTGCTCGTGTGGGATCGCCAAACTCTCGTCAGAGAACACGAGGTGGAGATTTCCAAGTTGAATGAAGAAGTGAATCAATTAAAAACAACGCTAGAAGAATTAAAGAATATTCATGGAGATTCAATTAAATTGATTACTAGTAAATCTAGCAAGCCTTCCCAAAAGGCTATTCTTGATGATCCTATTGCCATTCAGCGAAGGGAGAAAGTAAAAGAAGCTATGATTCATGCATGGAGTTCATATGAAAAGTATGCATGGGGCCATGACGAACTTCAACCACAGACGAAGAATGGTGTTGATAGCTTTGGTGGTCTTGGGGCAACTCTAATTGACTCTCTTGACACATTATTTATAATGGGTTTGGATGAGCAGTTTCGAAGAGCTAGAGAGTGGGTTGCAAACTCATTGGACTTTAACAAGGATTATGATGCCAGCGTCTTTGAGACAACCATAAGAGTTGTAGGTGGACTGCTTAGTGCTTACGATCTTTCAGGAGACACAGTTTTCCTGGAAAAGGCCAAAGATATTGCAGATAGATTGTTGCCTGCATGGAATACGAATACTGGGATCCCTTACAACATCATTAACTTGGCACATGGAAATGCACATAACCCTGGATGGACTGGTGGTGACAGTATTCTTGCAGATTCTGGCACAGAGCAGCTTGAATTTATTGCTCTTTCTCAAAGGAGTGGAGATCCAAAATATCAGCTGAAGGCAGAGAAGGTTATAATGCAGCTTAATAAGACCTTTCCTGATGATGGTTTGCTTCCAATCTATATTAATCCTGATAGGGGAACTGGATACTCGACCATAACTTTTGGTGCAATGGGTGACAGCTTCTATGAATATTTGCTCAAAGTTTGGATTCAAGGGAACAAAACTTCAGCCGTCAGACATTATAGAGAAATGTGGGAGAAATCAATGANNNNNNNNNNNNNNNNNNNGAAAACAACGCCATCATCTTTCACATACATTTGTGAGAAGAGTGGAGATGCACTTGCGGACAAGATGGATGAATTGGCATGCTTTGCTCCAGGAATGATAGCTTTAGGATCATCTGGTTATGATCCCGCTGACTCTAAAAAATTTCTTTCGCTTGCAGAGGAGCTTGCGTGGACTTGTTACAACTTTTACCAATCAACACCAACAAAATTGGCTGGAGAAAACTATTTTTTCCATCCTGGCCAGGACATGAGTGTGGGTACATCATGGAATATACTGAGACCTGAAACAATTGAGTCGCTCTTTTACCTGTGGCGTTTAACTGGCAACAAGACATACCAAGAGTGGGGTTGGAATATATTCCAAGCATTTGAAAAGAACTCACGCATCGAGACTGGATATGTCGGGCTAAAGGATGTCAATACCGGTGTCAAAGACGATATGATGCAAAGCTTTTTCCTTGCAGAGACACTGAAATATCTGTATCTTCTCTTCTCACCATCTTCAGTCATCTCATTGGACGAGTGGGTTTTTAACACAGAAGCCCATCCTCTCAGAATCGTGACCCGTCAAGACGGAGACACTTCTGGGAATTCGGAGAATAAACAAACTATTAGGACACACAGCAGGAAAGAAGGTCAGTTTACTGAGCATTATGTTGACCCCACAAGCTCAAAGGAGCATAGATTTTGATGAT

>Unigene1286_All

ATCATTAAGGACAAGAACCTGAATTCTGCACCAAGGCGTGAAAAACCCCTAAAAAGCATTGGTATGCATAAACAGAAGACTAACCTGGAGCATCCCGAAAAAATTGCAAGTATCGAAACAGATAAAGAACTGGAAATGGAAATTGTTGATGTTGGACCCTCTGCTGATTGGGTGAAGATCAACGTTCGGGAATCTAAAGATTGCTTTGAGGTGTATGCTTTAGTCCCTGGGCTTTTGCGAGAGGAGGTGCGAGTTCAATCAGATCCTGCTGGACGCCTGGTTATAACAGGTCAACCTGAGCAGCTTGACAATCCCTGGGGCATAACACCCTTCAAGANNNNNNNNNNNNNNNNNNNNNNNNNNNNNNNCAGACGTCTGCTGTTGTTAGCCTTCATGGCCGACTCTATGTTCGTGTTCCTTTTGACCGAGTATCAGCTTGAGTTTCTCGTTTCTGTGAGCCTGATAAACATCTTCAAGGGTAAACATTTTAAATCGTTGGCAGAGACGGAAAGAAGGTCAAAATTGCAATGAGGGCATTGTGAGACATTTGTTTCTTTGTTAAATAGTTCAAACCACCAGACTTTGTAGAAGCC

>Unigene1336_All

ATACTCTCGCAGCCTCCTCTTCCTCTTCTTCGTCCTCCGCCTGCTCTACGAAAGACACGCGTGTCATAAGAATCAGCAAAGAAGAGGACGAGACCTGAAAATGGAGTCTACCATCGTTTCAAGTCCTCTTAGCCAAAATTACCTTTTATGCCCTTCTCGCAACTCTTCTTCTCTCCTTCCTCTTTCTTCCTCCTCTCACCGCTCACTTCATTTCTCTCCTTCTCCTTCCTCTCTCTCTCTTAAGCTGCAAAAGGGGAATCTTTTTTCTAATGCCCTTCGAGCTGTCTCGCCTTTCAGTCCTGACGCTATAGTTTCTGATACTTACAGAAGTGAAACAACTGAATTAGCTGACATAGACTGGGACAACCTTGGGTTTGCATATGTTCCCACTGATTATATGTATATCATGAAATGTGCTAGAGGTGGAAGCTTTTCTAAAGGTGAATTACAACGATTTGGNNNNNNNNNNNNNNNNNNNNNNNNGTCCCTCAGCAGGAGTCTTAAACTATGGGCAGGGATTATTTGAAGGCTTGAAAGCATACAGGAAAAAA

>Unigene1390_All

CAGGGGAAGGAAGATATCTCCTGATGATCATGAAGATTATAATGACACTAAATCTAGAGGTGTCAAAACACTTTATCTTGACATGGAAAAGAAATCTTCAAGCAGCAGTAGAGTTGAGTCTGATGCTGACAGGGGAAGGTCTCAATCACTTCAATCATCATAGTCAAGATNNNNNNNNNNNNNNAGCAATAGGAGGAGAGCTTCCCCCAACACCAGTTCTCATGGAGCTGCAGATGATTACAGGC

>Unigene1430_All

TGGACATCAGACATGCAGCAAATGTTCAGACCGCTTAGCAGATAAGCCATGTCCATTCTGCAGAAGGCCAATTCAAAGCAGAACACTGTTAGGCAGCATTATTTGAACACCAAAAAACGAGTCTCTTTGTTCTGGCTTTAGATGAATGATGCAAATATGAGGACATGAAACCATCTCTTACTGGAATTTTTTCCTTTTCAGTTAACACTTGCAGGGCACTGGTTCAAATGCTTGAAAAAAAAAAAAAAAAAAAAAACCTATACTTTGGGGTTTTCGGTTTTTTTTTCCTTTTAAATCCAACCCCCAATTCCGTTTTTTCCCANNNNNNNNNNNNNNNNNNNNCATCCCTGTAATTTGATGGTTGTGGGCATGTGAATTTATCGTTCTTGTTTGGGAGTCGGTGC

>Unigene1448_All

GGCATGTTATTTACTAGTTAGGTGGCTACCTCTTTGTTCCACTGTGTGAGCTATTTTCTCGCTGAACAGTACGAGGTACAAACATAAGACCACGAGTGTGGCGGCGACCAAAGCTGACAGAAGGAGGTGAATCTGAAGTGCTTAATCTAGAGACTGCTGATATGAGGCCATCAATGGTTTCACCTTCTACTTCCATTTTGGTTGAAGTTTCCTCCCTCTTTCGAATAGCTAGTCTACGCTGGTGCTTCTGTCTTGCCTTCTTTGATGGATGGGCCTTCTTGAAGAATTCAAATGCGCTGGGAAATTTATGCTTGTCCACCAAATGCTGTTGCCTACTTTTGTAGTTCTTGAATTTCAAAGCACAACCTTCCACCAGGCATTCATACATNNNNNNNNNNNNNNNNNNNNNNNNNNNNNNNNNNNNNNNNNNNNNNNNNNNNNNNNNNNNGGAGCCGTTGGGTAAACTCTAGAGCACACCGAACAGGATGCAGTGTGTCGTGTATTGTAGTGGTCTTCAAAGTTTTCCAAACATGTCAANNNNNNNNNNNNNNNNNNNNNNNNNNNNNNNNNNNNNNNNNNNNNNNNNTTCTGCAACTGATGCTTGTCATCTTCTGTTAAATCCAATGCAACCTGTTTGGCGAGAAGCT

>Unigene1451_All

TAAGTTTGATGATTTGAGAGCATCCAATGGATTGGTTAGCAAACTATCCATACCCGAGAACATCAAGAGATTTGTTTATGCAGTTCGTGATCCTGAATCTCAATCTGTGATTTACATACTCTCTGTTCAGAATTTGTCTGAGCGATCGGCTATAGATGCTGAGTGCCTTATTAGGGAGATTCGACCCGAAGCTGTTGTGGTTCAGGTGTCTTCTTCTGCATTATGTCAAATTCAATCAGAGGAGGGGGAATTGGGAAATAATATAGAAGAACCAGTGCCAACTTCGTTCTTTGGGGTGATTAAAAGATGTTTTGTTGATAAAATCAGTAAGGACAAGTATGAAAATGTGGCTGGAAAGTTGGTTTTGAAAGAAATTTTTGGGATTGGTTTTTATGGTCATATCATGGCAGCTAAGGGAGGGGCTAGGGGGGGTGGGTCCTTGTTTTTTCTTCCTGTAAAAACACCTGTTTATTCCNNNNNNNNNNNNNNNACTTCCAGTGAAGTTGATACAGGGAGTGTGGTTCGTGGTTTAGTTAGTAGTTTGATTCCACAGAAATTGGGTTCGGCTGTTTCATCAAGTTCAGGGAAATTTTGTCTTACAGATGAAATNNNNNNNNNNNNNNNNNNNNNNNNNNNNNNNNNNNTCTCCATATATGGAAGTATCTATACAGAAGTTACGTCCTTCAAGTTCTGTTTCAGAGGCAGGGTCAAAAGAAATTCACCCAGGAAGCAGTTACCTGTTGCCACCATTTGCCCAATCTGTTTATC

>Unigene1502_All

GTCATTCGTGATTTTCATGCCAAGAATTGATTTGTGGGCTGTGGAATCATGTCAGCAAGTTACTGAGGAGAGTGATGCATCTTCAACAGACCATCAACTTTCTGAGGAAACAGAATTTTGTTCCACACCAATTCAAGTTGTTGGAAAGGAAAATGAATCCATTGCACATCAATGCAGTGAATCTGAAATGCCACAGCCACAAGATGCTACCCTGTGCACCTCACATGCCTGGAGCTCATTTGTTGAGCAGGTGGAGTCTATACGCGTGTCAACATCCTTGATCATTCTGGCTACTTCAGAACTTCCATATCTGAAACTCCCACATAGAATAAGGGAATTCTTTAAGAGTGGTATCTCAAATANNNNNNNNNNNNNNNNNNNNNNNNCCGATTCTTGGTTCATACTGGTGGGAATTTCAATCGTGATGTGGTGGCCAGTCTTTCTGCGGCA

>Unigene1510_All

TTCCTTGCTTATGTTTATCACAGCCTACCAACTTCGACGTGCTATAGCCGAACTTCCCCAAAAGGAAAGCAGGGCAGGAAACAAAAAATTCTCTTTCTGAAAAAAAAAAAAAAAAACTCAAATCGATCTTTTTTCTCCTCCTTTTTGTCAAAATCACCTACCGTTTTCTGTTATTTGTGCTGAATTTTATGTAGTGCTTCTCTCCAGAGTTCAACTTTAGATTTGTTTATCTACTACTGTACTTACGATTCTAGCTCCTACAAGTATCCTCTGTTTTTGTTTTTTACTTTCAAATTACGGAAACTGGTAAAGGGATCAATCCAACTGCAACGATGAATCCATATTACGCGGTGAAGGAAGAGTACCCAGGATCAAGCTTGTCGCAGTCCGGTGATGAACCACCAATAACTGTCCTGCCGCAACCGATGGAAGGGCTGCATGACACGGGACCACCACCATTCCTGACCAAGACTTTNNNNNNNNNNNNNNNNNNNNNNNNNNNNNNNNGAGCACAGAAGGTACTAGCTTCGTTGTGTGGGATCCTCATTCTTTCTCCACAAACCTCCTGC

>Unigene1532_All

TATAGCCCTCCTCCTCCAGGGTGCGGACCCGGCGCAGACAGGGCGGCGGCGAAATGCCCACCCGCTTGGCCAGTTCGACATTGGTGATTCGGCCATCAGCCTGAATCTCATTGAGTATTTTCAGGTCGATTTCGTCGAGGCTCTTTGACACGGACACCTAATCCCCTGGCGACCGGATATTTCCGGCCTTTCGCAAAACTGTTCTAGCCAGCGGCACGGCTTNNNNNNNNNNNNNNNNNNNCTATACGCTATCTCATGGCTAGCTGGGGAAAATTTCATGATAGGGGTTGCATATCTTGCATAGCTCGCAGGATGGCCTACATTCGGAACTGTTACTTTTAACGCCCCAGCGA

>Unigene1576_All

CCATGAAAAATCTGATCATAACCCATCCCAGTTTGAGCCAGAACAGGTGAGATCTAAATATGATGTTCAGCCTGTGCATAAAGCAATCAATGCACATGAAGGGACATGGCAATCAGTGATCCTTTTTAATTCTTTGGAGCAAACTAGAGAAGAGGTTGTGATGGTTATAGTTAANNNNNNNNNNNNNNNNNTGTTGCTGTTTTGGACTCAAACTGGACTTGCGTTCAAAGCCAGGTTTCTCCCGAACTGGAGCATGATAGAAACAAGATTTTTACTGGGAGGCATCGTGTCCATTGGAAGGCTTCTGTTCCTGCCTTGGGGTTGCAAACATATTATATTGCCAATGGTTTTGCCGGATGTGAAAAAGCCATACCAGCTAAATTGAAGTACTTCTCAACGTCAAATTCATTTTCGTGCCCCACTCCATATGTTTGCTCTAAAGTAGAAGGTGACATGGCTGAAATCCAGACTCAACATCAAACTCTCACCTTTGATGTCAAGCTCGGTTTGTTACAGAAAATTAGCCATAACAATGGTTCCAAGATAGATTGTTGTGGGTGAGGAAATAGGTATGTACACTAGTCCAGGAAGTGGGGCCTACCTGTTTAAACCTGAGGGGGATGCTCAGCCTATTGTTGAGGCAGGGGGGAACATGGTGATCTCCGAAGGCCCACTAATGCAGGAAGTGTTCGTTTATCCTAAAACAGCTTGGGAACAGACGCCCATCTCCCACAGTACTAGAATTTATAACGGAGATGGTACTATTCAAGAGTTTCTCATTGAGAAAGAATATCATGTGGAGCTTCTAGGCAAGGAATTTAATGACAGGGAATTGATAGTTAGATACAAG

>Unigene1587_All

CACCTCATTGCTGTAACGCCCATTGTATCCCCCAAATGATACAAGAATATCCTCACCATTGTAGGAACTCACAGCCAAACTCAACCCCTCACTGGCAATGGGAACGCGCCCTTTAACAGANNNNNNNNNNNNNNNAACAGACCAAACAAGTGTTGACATGTTGAGGACAACAGTTTCAGAGACCCCACTCTTGTTGTTGCCACCACCAACAATAAACCAATTCTCCCC

>Unigene1600_All

GCTTTGTTTGGGATCGTAGAGGTGAAGAAGCTGCCTTTTTTTTGCTTCTGGTGGTGGATTTGGACCTTGGTGTGAAACCCACCGATGCTGATTAACTTGCTGGTTCACGAGATCTGGTGCATGGAGGTGAAGTGCAATGCAGCGCAGCTGGCATCGANNNNNNNNNNNNNNNNNNNNNNNNNNNNNNNNNNNNTAACATCAGATTTGAGGGTCCATCATCTGGAGTTGGTAATGTGATCTCTGGATCTGTCGATTTGGTGGTGTGTTTGAGGTGGTTGAGCAGCTCCGGGGAGGAAAAGGAGGCCGACACTACTGACTTGTCTTGTGT

>Unigene1606_All

TGAATTTCTGGGTCAAACTCAATACCTCCGGTGAAATCTTTCATAAAATCAGGAAGCTGAATTACTGGCTTCGGCTCGTCATCTGCCCATCGCGATTTTCGCTTGCGCGTACCGCTACCGGAATCATTATTATTAGTATCGGTTGACTGATTGCTGGTGGTGGAATCAGCCGGTGGATCCCATCGGCTGCGTCGACGTCGNNNNNNNNNNNNNNNNNNNNNNNCTGTCGCCACTATTGGTATTGGTGAGACCATTTTCGGATAGCAGGGGCCTAGAGATTTCGAGTCTCGGAGTGGTCTCGTAGGCGCTAAGAGGATTATGGCCGTCTTCAGTGAAATTTTGATTCAGGGTTTTGTCTGGATTTTGGGGATCGGAGAATTCAGGTTCTGGTGGCGGAGGAGGAGGATTTTCATAGTGAAAATCTAGGGTTTCAGGCGGCGGCTGAGAAGGATCTAAAGATTGGAATTGGGTTGGGTGGAGAGAGTCCATAGAGTCCATTGAATGGGGAGGAAGAAGGGGCAGGGTTTCAGATACAG

>Unigene1614_All

CCCGGTAGTTTTACAAAGACGCTTGAGAATGTTGTCAATTGTAACACAGCAGATGCAATTGAGCAATCGGTACAAATGAGAAATGTCTTGTTTGGTCTTATTCTCTGCAGAAACATTTCAAAAAGATAGAACCACGGACATGCTTAAGCAAACAGATTTATTCCCCAAAACTTGGCAGGCTGGTTGGTCCGGTAGCTCTTGTCCACACTTTTGATCAGGTCCATGTCCTCTTTGCTCAACTCGAAGTCAAATACTTCAAAATTCTCCTTCAGTCTCTCAATTTTTGATGACTTGGGGATGACCACTGTGTTTCGCTGGACGCCCCATCGCAGAACAATTTGAGCCACAGTTTTCTTGTATTTCTCGGCCAAACCTTTGAGAACAGGATCATCCAGACATGATACAGAACCAAACCATTCAGTATTAGCTGCAGCACCTCCAAGAGGCGTGTGAGCCGTAACGCAGAGTCCATGCTTCTGACAAAACTTGACAAGAGAATCACGCTGGAAGTATGGGTGTGTTTCTATTTGATTCACGGCAGGCTTCACTTTTGAGTAGGCTAAGCAATCTCTAGTAAGAAAGATGTCATAGTTGCTGATTCCAATGCTACGGACTAAACCCATGGAAACTAAATCTTCCATAGCATGCCAGGTAGTTTCCAACGACACAGTTGTGTTTATATCCAGCACTCCATCCTCGTCTAAGGCACTGTCAGTTGTACCCACTCCAGTATGCTTTGTGGCAATGGGGAAGTGAACAAGATAGAGATCCAGATAATCAAGCTGAAGCTTTTTTAAACTATCTTTGCAGGCCTCAATAACATGTCCATGGTCTGAATTCCACAGCTTGGTGGTAATGAAAAGGTCCTCCCTCTTGACAAGCCCTGTCCGAAAAGCTTCAGCAAGTGCTTCCCCTACTTCTGCTTCATTCTGGTAATCAGCAGCACAGTCAAAATGGCGATAACCAATCTTGATGGCATTAATGATGAGGTCTGTAATCTCTNNNNNNNNNNNNNNNNNNNNNNNNNNNNNNNNNNNNNNTTTTGAATCCGCTGTTGAGTGTAATAGCCATTTTTATCAACTCCTAAACTCTGCTCTCTACTCTGTAAC

>Unigene1639_All

TGGTGCTTTCTTTTTTAGGCATAATGAGTTCACTGTCAGCTAACCAATATGCTTGTTGGAGCAAAGGAGGGTCTGAACTGGGAAAATGAAGGGTTTGGTGGCCATAGGACCAGCTGGATAATGGGTTTCTGGTCTTGAAACTGGTGACTCTTTTTCTCTTGCTTCTTGCCTTGCCTGGGATTACAAATTTCTCCAAGGAAGATTGGGGGTTTTGTTGGGGCTTCTGTTGCAAGGGTTTTGGAGGGTTTGGTGTTGTATTTATGTNNNNNNNNNNNNNNNNNNNNNNNNNNNNNNNNNNNNNNNNNGCAGTCCTCTACAAATACTGACAGCCATTCTAAGCTAACATCTACTTCCTGCGCTTACAAC

>Unigene1643_All

TTTCACAAGCTCGTACTTATCCATTTTTTACTTCTTTTATTTCCCACAGCTAACCACAAATAAAAAACCTAAATTAAGCTCCAATTAATCTCCTAGTCAATCCCAATTCAGCTTCAATTCATATCTCCAACCTCAAAAACCGTATAAAGGCTACAACTTGACAATAATAATTCAATAAAAAACAGGAATTTTGAGTGAGAAGGGCAGGAATCTGAGGTAATTAACAGAGATTTAAGAATGAAAAGGGAGAAGGTAGTGATGGGTTNNNNNNNNNNNNNNNNNNNNNNNNNNNNNNNNNNNNNNNNAGGAAAGAATCAAACTGTGATTGTCATAAGAATAGAGAATCTACAAAACTGTAGCTTTTGGGTATTGAGAGAGAGAGAGTGTGTGTTAATGTATCCTTTTGCAC

>Unigene1646_All

GACGAATAATATATATTTGATCATTGCATTCACATACTACATATGTTGCATTTACGATGACAAAACTTTTCAAGGTTTTCCTGAAATACTTGTACTTGCCGGATCATAGCACAAAATTTGAAATCTTGGGGTCACTTGGGACTGGGTCAGCGTCCAACGTATAATGGATAAAATTTAAAACCCCTTCTGCTAAACAGTTTGTAACCATTTGCCATTATTGACAGCTACAAATGCATCTGTCAGAAAGACTGGATAACGTTTGCATGCATACATCATCTATGTATTAACGCCGGGTAATGTGAGAAGACTCAAAAAGAGCGAGCGATGAAATCACTTCCAGAGCTTGACATACNCAACAAAGCCAGTAACATCTGACACTGCCAAATCAGATACCAATTTGTCATGCGCACGTACATCCATCGTCTTGTTCTCAGCCATGTTCCAAAGCCCCAGATTCCCATAGCAGCCAATGATCAGCAGAGACGAGNNNNNNNNNNNNNNNNNNNNNNNNNNNNNNNNNNNNNNNNNNNNNNNNNNNNATTCATGAATGTATTCCCCTTTACTGCCGGAGCCCACCGTCCACACTCTAACCAAGTCATCACTCACAGATGCCACATACTCGCCAGAAGGATCCCAACACACAGAATGGATTTGGTTTTTATGGCCCTTTTAGTCTCAACATCTAGTATGGATACCACATTCTCTGCAGCAGCTGCAAGGATCCTTCCAAAATGAGGTTGAAACCTTGTCTGAGTAGC

>Unigene1652_All

GTCCGAAGTCGCTGGTGCTGATCATGGCAATGGCAGGGTCCACATTATACTACAGCCTCCACTGCAGTTGCCGTCCAAACACCAACTGCCAATTGGTGGGTCGTACTGGTACTGCCTGATATACTTGATGGCGAGTAATAAAATTACCCAAGCCAACAACCTGCAACTCTGAAGTCTGGTCCTCAACCAAATCATGAATACATGTGCAGTATACAATATACAACATTGGTGAGAAATGAAGACACATACCAAACTCCCGTTGTCTGCCTCAAAAAAAACCTAGTCAATTCCTCAGTTTACCCTCATTATTTACTAACCTATTACCATTTGTAACCCCAATCCTCTTGCTCCTTGAATCTCTTCAATACCCCATCTATGGCAATATCAAAGGCATCTTTTATACTCTCTAAGCCTTCGTAGCCTCNNNNNNNNNNNNNNNNTACACAAATCTGGGGATATAATCAATCACTTTTTCTCTCATTTTCCTTACCTCTTCTTTGCT

>Unigene1655_All

AGAATCTGAGCACCAATTAGCGCTGGATAAAGCCTGCTTCAGTCTTATCAAAGTTCGTAACTTCATATTATCATCAATGGCGGAAGCAAACAAGAGGTATGCAGTGGTTACAGGAGCAAACAAGGGGATTGGGTTTGAAATTTGCAGGCAGTTAGCTTCAAACGGGATTGTGGTTGTATTAACAGCCAGAGATGAGAAGAGAGGCCTTGAAGCTTTTCAAAAACTCAAAGACTCTGGTTTCTCTGATCTTCTTGTGTTTCATCAGCTTGATGTGGCTGATGCTGCCAGTATTGCTACTCTTGCAAATTTCATCAAAACCCAATTCGGAAAGCTTGATATCCTGGTGAACAATGCTGGTGTTNNNNTTGAAGTTGATGTTGATGCTTTGAAAGCTGAGTCTAGTAAGGGCAGTGAAATAAATTGGGGCAAAGTATCAACTCAAACATATGAGTCAGCTGAAGAATGCCTGACAATAAACTACTATGGTGCCAAAAGAATGACCGAGGCACTTATTCCACTTCTCCAGTTGTCTGATTCGCCAAGGATCGTTAATGTTTCATCCTCCATGGGGAAGTTGAAGAATGTAACAAATGAATGGGCTAAAGAGGTGTTTGTTGATGCTGACAACCTATCAGAAGAGAGAATTGATGAGGTGTTGGGCAAGTATCTAAAAGATTATAAAGAGGGTTCACTCGAAAGCAAAGGTTGGCCTGCTTTTATGTCTGCCTATATACTCTCAAAAGCAGCTATGAATGCCTACACAAGAATCCTAGCCAAGAAGTTGCCAACTTTTCGCATCAATTGTGTCTGCCCTGGCTTTGTGAAAACAGATATTAACTTGAATATGGGAATATTATCAGTTGAAGAAGGGGCNNNNNNNNNNNNNNNGAGGTTGGCACTGTTGCCTAATGATGGTCCTTCTGGTGGTTTTTTTTTTCGGAAAGAGGAG

>Unigene1664_All

CTCGACTTTGTCATCAATTAAAGCTCTGTAGTGCATATCTCGGGGTTCCAGGGAGCCATAGATCAAGAGACAATTGTGGAGAACCATTAGCAACATCATACCACTCCAAGTTTATGGGAACTGTTGATTATATTTGGCACACTAAGGAACTCATTCCTATCAGAGTTCTTGAAACCGTACCCATTGACATTTTGAGAAGATGTGCAGGGCTTCCTAATGAGAAATGGGGAAGTGATCATCTTGCCCTTGTGTGTGAACTAGCCTTTGCAGACGAAGACAACATGACCTGAGTCTTCAATTGCAGAAATGCTGACATGATAAAATTTTCAGCCTCTTTTACAAGTTGGGTATCCAAAGTTTAAATTAAGAATCCACCGACTCATTAATTATGTTTAAAATCTTAATATTTNNNNNNNNNNNNNNNNNNNNNNNNNNNNNNNNNNNNNNNNNNNNNNNNNGATTGTCAATATTTTTTAACGTGTTGCAAAGCACAAGCAATGTCAATATGTGATTTTTTTT

>Unigene1683_All

GGCCAAAAATCAGCAACTAGTAAATGTCGCAATAAATAACTCAACATTAATATCTCGCTTCGTTTGGATTATGCCGACAAAAATTTCAACCACGCACAATCCATAACCAACTTGGAGGAAAAGACTGTGGCCTATCCTAATCCTATGCCTACAGAATACATATCCTGCTTACCTGGCAAAGTTCCAGCTGTTAAAGCTGACAGGTGACAGGTAGGGCCTTGTCCAGAGAAAACATGATTATAAAGTCTGTGGGCATCAAGATCATGGATATACTTGATAGATTCTAGTAATGTCTTGAGGCTTTCATCATATTCAAAATCAGGTGAATTCAAAATANNNNNNNNNNNNNNNNNNNNNNNNNNNNNNNNNNNNNNNNNNNNNNNNNNGAGTAAATATGTCCATGGGATCTGTTGAACTTGCTAATATCCATGGCTTATAAAGTTTTGC

>Unigene1689_All

TTGACAGCGGCATACTCCTTGATGCTCATTTCCCATTCCTCTGCTCTAAAATAGGGTCCACAGACTATGTGATGAGCATCAAGGAGTATGCCGCTGTCAAAAGCCTGCCCATGGCAAAGTAATTACCAGGCACCCGGCGTGTATAGAATTTCAAAGCATGTCCGCAGGGTAACAGCAAGCATCTGTTTTGTGAGATAATATGATTCTTGCATTATTTCATTGCAATAAAATCCAAGTGCTTAAGCTGTTGCTGCAGATGGACACTTCCCCAAAGTATTCCACAGTATAAAATCCCCAGAAGTAACCTGAGTTGACAACTCCACAGGTGCTTTAACAGCTGGGTAGATGCTCACAGANNCTCACAGATTTAGCATCAATGGACATTCCAGATAATGAAGCCCCAGTTATCTTGAAAGATACTTTGGCATAGTTATATGCTTGCCAGCAAAATGGCTCCTCTAGATCAACTGGAGGAAGATCCTTACTCATTTTCTCCATTAAGTATTCCTCCACATTTACCATATTGTTTGTGNNNNNNNNNNNNNNNNNNNNATCACTATCTCTGTCACTCCTGTTATCAGAACCAGACTTTGAGGAAGGCGCTCTTTGAATTTGCCATGGCGCAAATCGAACTGTTCCAGGGAAAGTTGCTTCAATACTTT

>Unigene1734_All

CAGCCTAAGGGCTGCGATCTTCCACCGTTCGATCCCGTCTGGTTCTTGGAGACTTATAGAGACACCAAAATTGGATTCGTTGGCGATTCCTTGAATAGGAATATGTTTGTGTCTCTTTTTTGCGCACTGAAACGGGTTTCAAGCGAAGTGAAAAAGTGGCGTCCTGCTGGGGCTGATCGTGGGTTTACGTTTCTTCACTACAATCTTACTATTGCATACCATCGAACAAATCTTTTAGCGCGTTATGGTAGTTGGTCAGCTAATGCTAATGGTGGTGAGTTGGAATCCCTTGGATATAAAGAGGGCTATAGAGTTGACATGGACATTCCCGACGGCACATGGGCAAATGCTCCCAGCTTCCACGATATTCTAATCTTTAACACTGGACATTGGTGGTGGGCTCCTTCAAAATTTGACCCTGTAACGTCACCCATGCTTTTCTTTGAGAGGGGCCAGCCTGTGATCCCACCCTTACCCCCTGATGTTGGGCTGGACAAGGTTTTGAAATACATGATACTGTTTGTTGAGAAAAACATGCATCCAGGTGGGCTCAAGTTCTTTCGTACACAATCTCCTAGACATTTTGAAGGAGGTGACTGGGACCAGGGTGGTTCCTGTCTACGACTAAAACCTTTATTGCCTGAGCAAGTTGAAGAACTCTTCTCTATAAAGAACAATGGAACAAATGTGGAATCCCGTTTGGTGAATCAACACCTCTTCAAGGCCATTGAGGGNNNNNNNNNNNNNNNNNNNNNNNNNNNNNNNNNNNNNNNNNNNNTCATCCATCCACTGCTGGTGGGAAGAGGCATGATGACTGCATGCACTGGTGCTTACCGGGAATAACGGAC

>Unigene1760_All

ACCTTCAACGGCACGCCTGCGGCTGTCACGATCAGCTCCCGGCGGAACGAGCCCTGACCAGATTGAGATCAAAGGCCTCTTTCTCCGTGAGCCAGCGCATGTCCTTGCTCGCAGTCATCGCCTGCATGATGGAAGGCGAAACGCCCATCTCCGTCATGTAATCGAGAATGATGCCGGTGGTGCGCTGGGTACGCGACACGATATCTCCCGCGGACTCTTCGCCTTTAGCTCCTGTGCTGCTGAACTGATGCACGCCGAGCATCGACCGCGGCATGGCCTCCCGGATCTTGCCTCCCGCNNNNNNNNNNNNNNNNNNNNNNNNNNNNNNNNNNNNNNNNNNNNNNNNNNNNNNCTCCCCACCACCGTCGTCAGTCCGCGCGAGCGGACCACCGCACCCATGATCAGGCCCTGAT

>Unigene1776_All

AGTTAAGATTATAAAATTCTCCCATGCAAATGAATTCGCGTGAACACATTGAGAGAGGGAGGCGGCTACTATGCATCTAGTCCACCAATGTGTTCAATTTTTCATTTTTTCCATGGACAGGGGGACGAAAGGACTTTGGGGGAAGCTCCAATGTGTCAACAAATAATAAAAGCTTAATCAGTTTATGCATGAGAAAATTATCTCCATACATTACTCATTTGCCCTGGTTCAGGTACTGCAGATCAAGTAAAATTTTTTTTTTTTTTTTTTTTTTTTTTTGCAAAGGAAAAGATAATTCCATTTGATCTAGATTCAAAACTTACTTCAAGTTAAACTTGCATTACACATGCCAGCCACAACTACAGAGGATGTGGTGTGTCATAGTCAACATAGTTATAAAGGCACAAGGCGCACTAAGATGCAAAGAGTTTCCAGGCTATAGTTGCGAGCACATGCTTGAATTAGGTAAGATGAACAAAAATGAAATTTAATAAAAAATTAAAATCATACTCATAGATATTGCAGTATTTAACCAAACTTATCAAAATTACAAATCTATGAAGCATTAATAAATGNNNNNNNNNNNNNNNNNAAAGGCGCCAATGTGTCCTGAAACCTAGGCACAAGGCGTAGGCGCATGCCCGAGCGAGGCA

>Unigene1784_All

TCTACATGTACTTTACAGTATGATAAAAGCTTCTTCGTGTCGCATTGGCTTCTTTTGCCTTGGGCAAATTAACTATAGACCAATGCCAAGCTATGATTTCTTTGGCTTTGGCCAAATTCGCAGTTCTGGGATGCCGACCTTTAATGTGCATAAAACCAACAACTCCTTGGCAAAACACAGGGTTACCACCACAACATCATTCTATCATACACATAAATGTTGNNNNNNNNNNNNNNNNNNNNNNNNNNNNNNNNNNGTAGGTGCATAGGTAAAGACTTTTTTATATATTTTGTCATCTCATTCCAGGTAGAGTTTC

>Unigene1791_All

ATTTAGTTTGTGGTAGTTGGCGCATACTAAATTGATGCTTTAATACATAAAATGCATCTACTGGCAGGCAATTGAGTGGGCATATCAATGCAGAGAGTCCAGGAAGTCAACTCTATACATGTTTATAGAACCTTAAACAGGCTCATTTGTAGGTTCCCATACCCACACCGATCTCTCCACCAAAAGTCTGGAAGCTGTCTCTGGCCTTGGAGTAGAAAATATAGTAGAACTCAGAAACTATGGCAGTGAAGAAAGTAAAAGCTGCCCCTGCCCCAAAAACCCCCTTCCTCAATGTCTGACAATCAGGAGGGTTGTCACTGAAAACAGTCCTGTACTTGGTGTGATATGCATTCCTCACCGAACCCGCCAACAAGCATCAGCAATGAAGAAAAACCCCCAGCAGGTAATGAAGAGGATAACTGCCCAGGCCCTTGAACCTCCAGGATTCAAAGGTTTGCCACAGCAGAAGCATTGACTTGCCACCATTATAAGGGCTTGACTGGCCATGAGAAACAAAAATGCACCAACACCATAGCCAGTTGCAATATCCGAGTCATATACACAGTAGTTGTAATTTATTTCAGAATCCTGTTTGATTATAGCAGTGCTTCTTCTCTGCTCAGCTGCAACAGCCAAAGCGAAGGCAATGAGGTCGAAGATGAAGACCGTAATCAACAGAAGTTTTGAAGCCATGGCTATAAATCTTGAACACGTTCGCAGATCTTCCTCTTCNNNNNNNNNNNNNNTCTCTGGGAAGGAGAATCAAGACTCTAGCCGTCTCAATTCTTTCTCTTGTCTAAATATACC

>Unigene1817_All

CAGAGTACGGAGCCCGTGAAGGAGAGAGTCAAGTTTTCGGTTCCCCTTTCAAAGGAGGAGATTGAGCAAGATTTCATGGAGATTTCTAGGATTAGGCCGCCCAGAAGACCCAAGAAGAGGCCTCGAATTGTGCAGAAGTATTTGGACTCGATTTTCCCAGGATTGTGGCTAACAGAAGTTACACCAGATTCATACGAGGGTCCAGAAGCACCTGAGTCATGAAAGCATGACAGGGACAGAAATGAAAGTGTAATTGTTTTCTGGGGAATTTCTTTTATTCTGCTTGCTAACACTGGNNNNNNNNNNNNNNNNNNNNNNNNNNTAACATTGCACCATCTTTGTAAATTGCAAATTCAATAAAATAAAGTTATAAATTCCAGTTG

>Unigene1882_All

TAATGTAAAAATAACTTTCCATATTCCTGAGATGGTATCATTTGTTAATTTGTTAATCAGTCAACAGCAATTGAACAGATTGTCTAAGGATATTTCCAAACTCGAAAACTCACATCGAAATGGCAGCCAGATACCAGTTTCATGTTCATTAAATAGTACAAATAGCATTCAAGCCGTTCTACCTTATCCACACGATAAATTAGTATTATATACTCTTAGAGATTGTGATCACTTCACAACTTCAAACTTGGAACGAAGGTTGGAAATCATTACTGGGCACTCCCCCTTTTCATCATATTCAGCAAATTCACCTCCAGACAAATCAATATCTTCGTATTTAGNNNNNNNNNNNNNNNNNNNNNNNCGCTACCAAATACAAAACCCTCAGGTTCATAGCCTCTGCAGTCAAACATCATCAATGGGGCATATTTCCCTGTATCACTGATTTCCTGGGTAAGCAGNGCCGGGCACCATCAACACAGTTCCCTCCCTCCCACAAAACTTGCACTTCTGAACAAGATTAGCGGTTCCCTTGCTTGCTGGAACAGGAACAGTCTCATTCAAGGTCACACATGTTTCTTTCTGGCTCAACTCTCCACACCTTCCACATTTCACCTTGAAGAAGTAAGGGAAAGAAGGATCGTCGCAACCGCCCTGAGGCTGGAGGCTGGTGATGTTCTCGAGATCGGCGGTTATCATTAGCATGTAGTTCACCATTTCCACTGGTTTTAGCGTCAAAATCAAGC

>Unigene1906_All

AAGAAGATACATCAGATGTACGGCCAAAATTTCTTGAGAACTCTCCATGTTGGAATAAGTTAAATGGGGAATGATGACATATAGAAGCATTGAAGAGTTGCTCTCGTACTGGTACACCAGAAGTTGCAACTGCCCAACAAGGTCGCAGCACTTCCATTGATCCACTAACAACAGTCTGCTCAGCTCCATTTCCCCCAACAGCCTGGACATGGTCTAGGACGCCATCCCCATTAATATCAGCATGAAGGCCACCTTCTTGAAGATGAAGCTTACAAATAGAACGACCTGATGCCAAGTGAACAGNNNNNNNNNNNNNNNNNNCCACAACAACATTTGGAACCCACCAAAGCTGAGTGTAGTTTGTTATGGTTGGAATATAAGGTAATGGCT

>Unigene1946_All

CTTCTCTGACAAGTCTCAAGGGAAAGGAGTAGAGTTGGCAGTTTACATCTCTGATAGTGTTCCTGAAATGCTAATTGGTGATCCAGGGAGGTTCCGGCAAATCATCATAAATCTCATGGGAAATTCAATAAAATTCACACATCAAGGGCACATCTTTGTCACTGTACATCTTGTGGAGGATGTGATCGACTCCATAGATGTAGAGACAGAATCATCATCAAGGAACACCTTGAGTGGTTTGCCTGTTGCAGATAGATGCGGCAGCTGGGCAGGATTTAGNNNNNNNNNNNNNNNNNNNNNNNNNNNNNNNNNNNNNNNNNNNNNNNNNTCATTTCTGTTGAGGATACAGGAGAAGGGATCCCTCTTGAAGCCCAGTCTCGCATTTTCAC

>Unigene1955_All

ACAGTCATTGGTAAAACTAAATTTAAGTGGAAATCATCTTTTGAGTGTACCTTCAAGCATCAGCCAACTTTCTAACCTCGTAGATGTTGATTTTTCTAATTGCACAAGACTCAAGTCTTTGCCTGCTCTTCCATCTAATATTGAACATCTAGATATGAAAAATTGCACTTCATTGCAAATTCTTCCAGATCTGGTTCAACTTCATAGATTGCAAAAACTTCAGTGTGCTAATTGCAAGAGCCTTCAGTCATTGCCGGATCTTCCTACCAGTGTTAACTTTCTAGATATGGAAAATTGCACTGCACTTGCAACACTAACAAATCTATTTGAAACACGAAATGTGGGGAAGGGTTTTTATATAGGTTTCTCAAATTTTTCCAAATTGAATTACTTTCAAGGTAAAATTAGCGTGCCATTTACATGGCTCAGATATTACCTGCTTTGGTTACATGAAGTTCGGCAGCTAATGAAGTTAGAAGAAAATTGCAGTGTGGAAGATTTTGAAAAACCTTTCCCTCAGAGACGCATCCTTCCATTTTTGGAGCTCCCTGACTTCTATTTATGCTTACCAGGGAGTGGAATTTGCCATCAGAGACCTTGCATTTCATTTTTGGAGCTCCCTGAGTTCTNNNNNNNNNNNNNNNNNNNNNNNNNNNNNNNTCACGTATCAGGGAGAGGAGAGTCAGTTGAGAATAGAACTACCTGCAAATGATGAGTGGTGGAATATTGCAGGTTTTGCAGTTTGTGCTGTTGTTGGGGAGAGAAATGTTGGTGAAAATATTACTTGGTCCATTGCAGTCGGTAGAAAAAGTGGGCCAGCTGAGTGTTGGAGCAACCATGTTCCTGTCCCTGGAACTTCCCAAGTTACGTCGGATCATCGTAGCTTCTTCTTCCAAGTGAATCAACTGCTCGATCCAACTAGTGACTACTGTCCGACTGAGCTGTTACTTAATTTTGAGCCCAAAGAGAAAATCAAGAAGTGTGGA

>Unigene1960_All

CTTTAACGAGGATTTAAAGTGGACTCATTCCAATTACAGGGCCTCGAAAGAGTCCTGTATTGTTATTTTTCGTCACTACCTCCCCGGGTCGGGAGTGGGTAATTTGCGCGCCTGCTGCCTTCCTTGGATGTGGTAGCCGTTTCTCAGGCTCCCTCTCCGGAATCGAACCCTGATTCCCCGTCACCCGTGGTCACCATGGTAGGCACGGCGACTACCATCGAAAGTTGATAGGGCAGACGTTCGAATGGGTCGTCGCCGCCACGGGGGGCGTGCGATCGGCCCGAGGTTATCTAGAGTCACCAAAGCCGCCGGCGCCCGCCCCCCGGCCGGGGCCGGAGAGGGGCTGACCNNNNNNNNNNNNNNNNNGTCCCCCCCACGGGAAGGGGGGTCAGCGCCCGTCGGCATGTATTAGCTCTAGAATTACCACAGTTATCCAAGTAGGAGAGGAGCGAGCGACCAAAGGAACCATAACTGATTTAATGAGCCATTCGC

>Unigene1990_All

GTTCGATCAATGGCCAAGGTTATCAGAAGCTTTGGTGTTGCCTGATGTTAAGCCAGTTTCAGAAGATAATAAGGATAAACTAATAGTACTGGCTAAGCCAAATTCAAATCTTTGTGGGGCAAAGCGGAAGGCTGCAACACCACCTGAACAAGGTGCTGTTGGGCTCCCTGACGCTGGTTTGAAGAAGATACCCAAGGAAGAGTGGAACTGTACTCTTTGTAATGTTAGCGCTACAAGTGAAAGAGGTTTGAATGAACACCTTCAAGGCAAGAGGCACAAGGCTAAGGAAGCAAGATTGAGAGCAAATAGGGTGGCCAAAAACTTAAGTCCTATACCGTTTCCAAAGAAAACTGCAAAACCAGCTAATCTCACTACCAGTATTGCAGGCTCAGAACTAGAGGCAAAAGTTGAAGGGGAATTACTTGAAGTTGAAAAAGCTAATGATGATACAGACAAGAAAAGAGAGAACAAACAAGATTCAGGGAATGCAAATGATAAGCTGCAAAAATGTACATACAGAAAATTTGAAGAGAAAAGGGGCAGGGNNNNNNNNNNNNNNNNNNNNNNNNNNNNNNNNNNNNNNNNNNNNNNCAATTCAAGTTCTGGTGTGAAATGTGTCAGATTGGTGCCTACTCTGCAGTGGTAATGGAGC

>Unigene1991_All

CCGAGACGCCGCGTGGGTCGGCTGACAAGAGCTCGGTTGGCCCTGCTTGATGTGCCCATGGCCGAAGGCCCCGATCAGGCGTGCGCCAAGGGCGCATCGCCTCAACGGAAAATGGCACCCGCAGGAGCGTGAGCATAGCGACGCCGAAGGCGGCACGCGGGACCGAGTGCAGCGACGAGACCGGTTAAGAGGGCACCCGGGAACGGGGCAGTGATAGCGACGCCGGCATACTTCATGACGGCGGGCCCCCTTNNNNNNNNNNNNNNNNNNNNNNNNNNNNNNNNNNNNNNNNNNNNNNNNNNNNNNNNNNNNNNNNNNNNNNAGAGCCCGGTCCGGCACTTGCCGGACGCGCACAAACGCAAATGAAGCGCTAGTTGAGGGCT

>Unigene2013_All

TTGACAAAATGCTAGTTGTTACAATAGAATATGATGTAAATAATTCTCACAGCCAACAATAATAGGTCTCAAGCATGACTTGCCTGAGGGAACATAGTCTAACAGACTTCACTATTGTATAGCAGCAACCCTCCTACACTAATCTTAGGAGGTAAGCTAGTTAATGGCAAACAAAAAATTGAAATTCTTGGTAACCACATTCAATNNNNNNNNNNNNNNNNNNNNNNNNNNNNNNNNNNNGGTGACTATTGCAGCTGCATTAGCTGGAAATGCTCGCACTATTGTAGGAGCCAAACCTGCATAACACCCTTTAAGNNNNNNNNNNNNNNNNNNNNNNNNNNNNNGTACTAGAAATGGATTTCTGGTGGAGCTTTTATCTGGAGCAGTCTGGATGACAGTTTTCGCCACATCCAACGGCAAAACAGCAGACCAGAAAGCTACGCCACCAAGGCCACCACTCACAATTCCAACTCCCATGTCAATCAAGTTGTTGTGGTCAGAAAAAGAAGCCTTCAATTGTAAATGCATGTAGTAACGGACATACTCATAAACACTAAAGAAGACTGCATTTCCAAAAGATTCTCTTAACAGTGTTGCAAAGCCTCCTCGAAAAATGCCTGAAATCCCTTCATTTTTCACTGTTTGAAGGGCGCAATCAAATGGACTACTGTATCTACTGAACTTTGGAACCAAGGAGTCAGTACCTTGAACTTGCATCCTACACTTTACCAGCTCAGATGGACATAATACAAAACTTATAATTGCTCCACCATAAGCTGCAGAAGGAATTATTAC

>Unigene2031_All

CTCAGAATTCTGTTAATTCAATGGAGCAATCTAATCAAACCGATGAAAACGATCATGACCCATCAAGTCAAACCGATCAAAAGGACGGTTGCTTCTTCGACGTCTTCGAAGATTTTCCGTTTCATGATTCAGATCATCAATCCGCTTCCGACTCCACTCTCCTCGAAGAGGTTTCCCATCGGAGAAACATTCTCCGGCGACGGAGCCGGCCAACATCCGACCGTGGAATCTACGGCACCTTATCCAACGACTCNNNNNNNNNNNNNNNNNNNNNNNNNNNNNNNNNNNNNNNNNNNNNNNNNCCACAAAACTGAATACAAAGAGAAAGGACAGAGATATTATCGCGATTTAAAGGAAAATGAGAACAATTTGTACTTTACTGAGTCAAATCGGTGGAATCGGGACCGAGTC

>Unigene2046_All

CGACCATTACAAAATAAAATCTCACATCACAAAGCAAATGCTCAGAGGGGCGAGAAGTGGTGGGTAAAACCCAGATAAAACTACAACAAAGTTGGATAAAGCAAACTTTCCTAACCAGTCTGCTATTGAATTTGCATCTTTTGAACATAAATGCTCAAATCAGATGCATTTCAAACTACCACAGGAAACTTCAAACCAAAAGATGTGACCCTAACTACATCAAATGTACAAAGAGAACAACCTACTTAAAAAATTTATAGGTGACGTCNNNNNNNNNNNNNNNNNNNNNNCTTATATATGCTAATATAAATCCATTAATATATAGCAATCATGGCAGAGTACCAGAATTCCCATTCACACTTGCAGGTTGAGTGTTACTCTGGGTCTGAGGAACATAAAAAT

>Unigene2051_All

ACCAAGAAACATGAAGCAATCACAACAAATAGAATGGCAGATGTTGTGTTGATGTTAACCACACCACTAGATTTAACACCTTCTTTGAGCGTAAAATCACCTGAAGCATCCTTTAAAAGCTTGTCCTGGTCTATAGCCACTTCTCTGGCACTCCATACAAAAAAAAAGCCCCAAAGATGGTCCCACCAGCCATGAGCCACAAAAACACTTCTGCAACATCAACAAGTGGCCGCTGTGGAGAATATAGCTGCACAGAAACTGCAGAGCTATTCTTTATATAATTTTCCAAGCTTGCNNNNNNNNNNNNNNNNNNNNNNNNNNNNNNCCTATTCTTACATCAGTTTCATTTGCTTCACAAACCATTTTAAAAAGTTCTGTTCGATAGTTTATTATAAGAATAGCTGAAGCATTAGCGTCTTCAGCAATATTTGACTTGGTTGTGAAG

>Unigene2056_All

TTTGTTTGGAATCAACATTCAACTCAACCTGTGCTAAAATACTGTGAGCATACTGCGGCTGTAAAAGCAATTGCTTGGTCGCCCCATCTTCATGGACTTCTAGCATCTGGGGGCGGGACTGCTGATCGGTGTGTAAGGTTCTGGAATACGACGACTAACTCCCATCTCAGCTGCATGGACACTGGCAGTCAGGTGTGCAATCTTGTGTGGTCTAAGAATGTCAATGAACTTGTCAGCACTCATGNNNNNNNNNNNNNNNNNNGTATGGAGATACCCCACCATGTCAAAGTTGGCAACTCTTACTGGTCATACATACAGAGTACTTTATCTTGCCATCTCACCAGATGGACAGACCATTGTCACGGGAGCAGGGGATGAAACTCTACGGTTTTGGAATGTGTTCCCTTCCCCTAAATCTCAGAACACTGACAGTGAAATTGGAGCATCATCTCTAGGAAGAACTACAATCCGGTAATTAATGGAGACTTAAAAGAATCTGTGGCTGTTTCATCGGAGGAACAAAAGATCGTAGATATGTCTTCAGCTCGAAGATTATTCTACAAAATGGAGCCTCATCAGATTGATTTTCTTGAACCAACCCTTGACCAGCGCGGAAATTTGCAGGGACCTGAAAGGGCNNNNNNNNNNNNNNNNNNNNNNNNNNNNNNNNNNNCTGCAAATTATTAATTTTTTTCTGGTCCCTTTGTCCCTTGATTTATAAAGTGTATAGTGGG

>Unigene2124_All

TTTAACCAAAATTCTGAATCGTTTACCCCCACAGGCAGAGTAGAAATTCTAAACTCCAACGAGTCGCCAAAAAATCACAAAACCTCGTATAGTCCAGAAATGGGTCTACTCCAAATCAATTCAAACAAAAAACGCCAACAATTCACCAGTTGGATACCCTCTAGGACACACAAGTCAACTCAAATCCTAAACTCGGTCATCCCCAAACCAATCAAAAACAAATACTCAAAAGAATTGAAACTCCTTAATCGGTTAGCTTACTTGGCTGCAAAGCTGATCTGGGTCAATCTCTTTTTGGCTAAAAATCCGGGCTTTAGAAGAAGAAGAAGAAGGAGAAGAAAAGTGATTTGTATATCAATGGAGAATGNNNNNNNNNNNNNNNNNNNNNNNNAATAACTCCCCAGAAAGGAGGCTGTTTCGGAGTTTTCAAAGTCTTCCAAATCTATGGTCAATGCTTCCATTCTTGCTTCTCCTAGCAATCTCCTAAGACCATGGAAATAGAC

>Unigene2166_All

CTGTGGAACAAAGGGTAGGAGGAAAGGTTCATTGTCAGGATTAATTATCATGCGCCCCAGTAATTCTGCAGCAGCACAGTGCCAGGCTTTAACTGCAGAACCCAGCATCCCCATGATAGCTTGAACTGCACGCTTCTCTGTTATTTTGATGTAAGATGGCTTTGCTGAGCTGAAAATTCGAAGATCAAGCAGTGGAGCCAAATTTACTATTGTCTCAAGGGCATTTGTAACAAGTTCCTCATCCTCAGTAATATGATCTTCTATGCACTGGAATACAGTTTCCAAGCAATGCCGATGTTGGGCCATAATGACTTCATTCTCTGGCATGAAAGAGAAATTGCGTATGATATTTGAAGCAGCCACTGCACACTGCTGCTTTTCTGCTCGCCCCTCTTCATCTAAATTAAATAGTCCATCCTCATCAAACCACCACTCCGAAGGACGAACTTTGGCTGCATTCTTCTGCCCTGGAACTTCAGTTACAGAAGAACCTGACCCAAATCCGGGATGGGTGAGATTGTTGTTTGATCCTAATGCCTCGTGTCTGTTACCGAATCCTGTAACCAGAGAATTAGCACCTAGTGTTCTAACCCTAGGCTTACTGCTGAGCTCTTTAGGAAGAGCTATATCACGCCAGTCATCTATAACTTGGAGAAGAGCATCGAGCAAGCCAGGGATTTTAGCTAGAGAATCCTTTCGCATGTCTTCCTTTTCTTTGAAAGAGAGCATTGTCAGAGTGTTCAATGCCCATGTCAATTCGCTCTTTAATCCACTTTGGAGAGCCAAAACTATTCGCTTATTGTTTTGATCTAAAAAGGAGGAGTGAACTTGAAGCGATGGGCCAAGGAGATTGGACGGAGCCACGGTNNNNNNNNNNNNNNNNNNNNNNNNNNNNNNNNNNNNNNNNNNNNNNNNNTGGGCGCCCCCTCTTGGCCGCTGGCGCGGTAGCGCCACCAGCTGAGCCAGCGGATTTGTTC

>Unigene2227_All

CTTTCTACCGAAAAATGTTCAAATTTCTCCACTTCCTTCTTTTCTTGTTTCTCTTCATCTCCAGAAATTTCCTGATTTTTGCTCTCACTTCACCACAGGACATCTCTGCTCTCAAGGCCTTTAAAGCTTCCATCAAGCCACGTTCCATCCCATCATGGTCCTGCCTTGCCTCCTGGGACTTCACCACCGATCCATGTGCCTCCCCTCGCCGGACCCACTTCACCTGCGGCATCACCTGCTCCTCTGACTCAACCCGAGTCACCCAACTCACTCTAGACCCTGTTGGCTACTCAGGTCAGCTTGCCCCACTTGTTTCTCAACTCAGTCACCTCACTGTTCTTGACCTCTCTGATAACAACTTTTTTGGTCCTATACCATCCTCCATTTCCTTTCTTTTTAACCTCCAGACGTTGACTCTCCATTCCAACTCGTTCTCTGGCTCACTCCCTAACTTCATCACCAACCTTAAATCCCTTGAAGTTTTGGACCTTTCACGTAATTCCTTATCTGGGTATCTCCCAAAAGGCATGAATTCAATGTCTAGTTTAAGGAGACTCGATCTTAGTTACAATAAGCTAACTGGGTCTCTACCAAAACTACCTCCCAACTTGCTAGAACTAGCTTTAAAGAGCAATTCTTTATCTGGGTCATTATCAAAATGGNNNNNNNNNNNNNNNNNNNCAGTGGTGGAACTTAGCGAGAACTCATTAACCGGGGCAGTAGAACCCTGGTTCTTTGCCTTGCCAGCGCTTCAACAGGTAGATTTAGCCAATAACAGCCTGACACGCATCGAGATCTCCAAACCTGCAAATGGCTACAGCGACCTGG

>Unigene2229_All

AGTTGATTAAAACGTGTCCATGCCCATATCCCGTCACTTTCTCCCATTCTCTCAATGCATTCCAGACGAAACCCAAAGAGTGCATTTATGTCAGCAGCTTCAGCTTCATGGTTTCCCCGTATGAGATGAACATTCTCAGGATGTTCAATCTTGAGAGCAAGGAGCAAAGTTATTGTCTCTAAACTGTGCTGCCCTCTATCAACGTAGTCCCCCAAAAACAAATAGTCAATGTACGTAATGTCTCCTGCAGTGGAAGGAAATCCATATTCATCAAATAGACGCATCAAGTCTCCAAACTGCCCNNNNNNNNNNNNNNNNNNNNNGCTTTCAATTGAAGAACTGTGGGCTCATGCATAAAGATCTGTTCAGCTGCGTAGCAAAGCTCACCAACTTCATAAGAATCCAGGAAAAACCT

>Unigene2235_All

GGGCTAAAATTGCCTTGGAGATTGCAAGAGGGCTTGTGTATTTACACGAAGAATGTGAAGTTCCGATCATTCATTGCGATATAAAGCCTGAAAATATACTCCTAGATGAACATTTCACTGCAAGAATTTCTGATTTTGGGTTAGTGAAATTGTTACTGTGTGGTCAGAGTCGAACTTTGACCAATATCAGAGGAACAAGAGGATATGTAGCTCCTGAATGGTTCAGAAATGTAGCTGTTACAGCTAAAGTGGACGTTTACAGTTTTGGTGTTTTGTTGTTAGAGATTATCTGCTGCAGAAAGAATGTATCGAAACTCGAGCACGAAGAGATACTAACGGACTGGGTTTATGATTGTTTTGTGGAAGGGGGGATAGATGATGTGGTTGAATTTGATAAGNNNNNNNNNNNNNNNNNNNNNNNNNNNNNNNNNNNNNNNNNNNNNNNNNNNNNNNNNNNNNNNNNNNGCCCTCAATGAAGATGGCCCTGCAAATGTTAGAAGGATCGGTTGAAGTACCTTTGCCCCCG

>Unigene2256_All

CCGGAAAGTACAATGCACAGCACAAAGGTCCTGATCGTTCTTGTTCAAAACTTCTTACGGCATACAAGTGAAATACATAGGGGATATACAGGCAACTGAAAAATATTATAATTTTTTTTTAAAAAAAAAAAACAAAAATTCTAACCAGCATCACTATCATAGTTCAGGTGCTTCCGGCTCACGAGAACACCTTCATCCCATGTCTCCACAAACCTTGCTCCATCAACATCAATGCAAAGAAAGTGGCCATGTCGCCATCCATCTTGGAANNNNNNNNNNNNNNNNNNGACTTCACCAGACTTTGAATAGAAACGGCTTTCACCATTGGCTTTTCCCTTCCAAAAGTTTGCAAACCATCGGTCTCCAGTGTGGAAATAAAACCAACCCTTGCCATGCATCACATCATCCCTCCATGACCCCTGAAACACATCTCCGTTTCTAAAGTAGAATGCACCTAAACCTGATCTCTTGCCATAACGCCACATACCATCATATATGCTTCCACCTCCAAGGATAAGACGGCCCTTCCCTTCAGGAAGACCTCCTTGGCATCGCCCTCTATACACCCCCCCTCTGTACTGCATCTCCATAACAGGACTCCACTTTTTT

>Unigene2271_All

GTCATTTGAGGCAGATAAGGGTATAAAGCACAAGTTCCGCCATGGCGATGGAGGGAAGCCACCGTCTACTGGTGCAAACATGGGATAAGCACAGTGATTTACATTATCCAAAAGGCTTCATGCTTACTGCTTGATTTGAGTACCTCTCTTTTCCCTTTGAATTGCTTATTACTACTCTCAAACTTATCTACTAGCTTCCGATTCTATAGTTCTAATGTACACAAGGTGCTCTAATATTGCTAAAATCCAGGCATTTTCAATGGTGTTTAGGGTTTCTCGAAATCTCAGATGCTTTAAACTATTTCACACCGCAAAGGCCCAATCTTTGCCAATCGGGTTCCATTTAAAACCCCCATTAAACCCTAAATGCGTCTGCTTTACAGCACTTAAGCTAGAGTTTGATTACAAGTCACACAAAACTTCCACTACGTGTCAGTGTAAATACAGAGAAATGAGTAATATAGCTAAAGGTGGAAGTGTTTCAAATGGGTTTAGGCTTATAAGTGCCGTACCATGTTGTACAGTGCTGAGAGCTAGAAGTTTTTCTAGTTCATATTCCCAGGGAATTGGTAAAGGGAGTCCTGCATTGAAAGCTGGAAAGTCACTTCCTTGGTTAAGTTCTGACAAAGTTGAGGCAACGAAAGGGTTTGAGAAAAGGGCAAGTGCAAACATCAGTCGTTCTCATCGGGAGGAATCGACCAAAAGATTGGAGAAAGGCACTACTGGCTCTTCATGGCAGGAATCGGTTAAAAGGTTAGAGAAAGGAGCAGTTGCCAAAACTCACTCCTTGTCTCGGGAGGAGCCTGCAAAAGGGTTAGAGAAAGGGGCAGTTACAAGAAGTGCTCGATCTTCTTGGAAGGATTCAGTGGAAACTGCTTTGGAGAAGAAGAGCAATTATACGGTGAGGAATAAAGAAAATAGAAATAGAATGTTGGGTGGGGTGAAGGACAGGAAGTATGACACTGGTTACAAAGAAAGGAACGATAGTGTTGATGAAGAGGAAGATGAATTGGATGTTGTTGATGATCCCAGATGGGATAAGATAAAAAACACGTTCAAAGGAATGGCAGATGTGAAAGGTAGGACTGAGAAGCCTGCGCTTCGAAGGTGGAATAAGCAGGAAAATTGGGGTAGAAAGACATGGGGAGAAGCTACTGAATCAACTGTACCTAAGATGGTTGGCGAAGGNNNNNNTATGGGGTAGGTCCTGTTTTGGCTGCACTTTCAGCTGGTAGAAGAGAATTCTATGCATTGTATGTTCAAGAAGGATTGGATTTGAGTGGTAATAACAAGAAGAAGAAGGACAAGAAAGGGATTGAGAAGGTGTTGAGGATGGCTGAAAAAATTGGGTTAAGCATAAAGGAAGTTTCAAAACATGATCTGAATATGGTTGTTGATAATCGCCCCCACCAGGGCCTAGTGTTGGATGCTTCACCATTGGAGATGGTGAAAATAAAGGAGTTCGAGCCTGTTTTGCCTGAGGATGAGAAAGGTTCTCTTTGGGTAGCTTTGGATGAGGTTACAGATCCCCAGAACTTGGGGGCAATTATTAGGTCTGCTTACTTCTTTGGGGCTTCAGGGGTGGTGCTATGTGCAAAAAANNNNNNNNNNNNNNNNNNNNNNAGCAAAGCAAGTGCAGGATCACTTGAACTAATGGAGCTCAGGTATTGCAAGAATATGATGCAGTTCCTAGTATCTTCCGCTGAAAATGGGTGGCGAGTTCTTGGAGGTTCTGTTTCTCCAAAAGCTGTTTCCTTGAATGAAATTTTGCCTGGTGAACCCACAATTCTTGTTTTGGGTAGTGAGGGCGCTGGCTTGAGGCCATTAGTGGAGAGATCATGTACCCATTTAGTTCGGATCCCTGGAAATATTCCTGTGGATGTAACATTGGGAGGGAATGATGGTTTGGAACCTGAAGAAACAAATCTTGAGCGCTCAAGTGAAGAGTACCGGTCCCTTTTGGCCGTGGAGAGTTTGAATGTCAGTGTTGCGGCTGGTGTGCTTCTTCACCACTTAATTGGCA

>Unigene2295_All

GTTTCCTCATGTGGCTTGTTTCATATTGCTTATCCTAGTGCTTCTGAGACTTTTAAAACTGAACTACGGGCGATATACAGTCAGCTATGTCAAGATGACATGCCCATGGTTCGAAGATCTGCAGCAACAAATCTAGGAAAATTTGCTGCTACTGTTGAGCCTGCTTATTTAAAGACTGACATCATGACGATATTTGAGGATTTGACACAAGATGATCANNNNNNNNNNNNNNNNNNNNNNNNNNNNNNNNNNNNNNNNNNNNNNNNNNNNNAAGTTGTTGGAACCCCAAGATTGTGTGGCACATATTCTCCCTGTCATAGTTAATTTCTCACAGGATAAGTCTTGGCGTGTTCGTTACATGGTTGCAAACCAATTATATGAGCTATGTGAAGCTGTTGGCCCTGAACCTACCAGGGCGGATCTGGTTCCTGCATATGTTCGGCTACTTTGTGATAATGAAGCTGAAGTACGTATAGCTGCTGCAGGCAAAGTAACTAAATTTTGTCGGATTTTGAATCCAGAACTTGCAATCCAGAGGATTCTTCCTTGTGTAAAGGAATTGTCCACAGATTCATCCCAACATGTTCGTTCTGCTTTGGCTTCAGTTATAATGGGAATGGCACCAGTTTTGGGGAAGGATGCAACAATAGAGCAACTACTGCCCATTTTTCTCTCTCTTCTGAAAGATGAATTTCCTGATGTCCGCCTTAATATTATCAGCAAGCTTGATCAAGTCAATCAGGTAATTGGAATTGA

>Unigene2308_All

CCCGGAAAACACCTTGCTTGCTTCGCTAACAAGGCCCATCTTTGAATAAGCTGTCACCAGTGCACTACAGGAAACAGAATCCAATGCCAACCCAGAAAAAGTTACTCCTCCATGTACAAGTCTCAAACCATCCAGATCAAAGTTTTCATGGCACGCTCGTATAAGACACGCATAAGTAAAGTTATCGGGTCTGATTTGGGTTCCTAGCATCTTTGTATACAGCGATAGCGCTTCATCAAATTTGTGGGCTCGGGCAAAAGCTCGAATCATGGAATTCCNNNNNNNNNNNNNNNNNNNNNNNNNNNNNNNNNNNNNNNNNNNNNNNAGCAGAGCGAAGGTCATTGTTCAAGCCGTAGAATCTTAGAATTTTAGTAGCATAAAACGAG

>Unigene2366_All

GGCTTCTCTCCACACCATTTCTCCCCATAGTCTCCTTCCTCTTTCCAAATCCAAAAAAGCCACCGCGAAAACCCTTCCCAACTCGAATTTTCTCGGCTCAAAATATCGTGAGAGCCCAATTTTCTCTAGAAGCAAACGTAGAATTGAGAGTTGGTTCTTGAACTCCGTTGTGCAAGAAGAGCTTGATGTTATCCCAGTACAGAGTAGAGACAGCACGGACCAGCAAGAAGGGATGGCGGTGAGTCAGGTAGAGAGCGAAGGGACTGAGTTGGCGACTCAAGTTAGCGGGTTTGGATCGAATGAGGGGCAACTGTCTTTTGAGGGGTTTTCTTCTCCTTCTAGTTCTGGTATTGGAGATGATGGAGGGAGACAGAGTGAATCCGAAATGGATAGATTAATTGATAGGACTATAAACGCAATGATTGTTCTTGCTGCTGGTTCTTATGCCATAACCAAGTTGCTTACAATTGATCATGATTACTGGCACGGGTGGACCTTTTATGAGATACTGAGATATGCCCCTCAACACAATTGGAGTGCTTACGGGGAGGCTCTTAAGACAAATCCAGTTTTAGCAAAAATGATGATCAGTGGAGTGGTCTATTCTGTAGGGGATTGGATTGCACAGTGTTTTGAAGGTAAACCACTCTTTGAGTTTGATCGAGCACGAATGTTTAGATCAGGCCTTGTTGGCTTTACATTACATGGCTCTCTTTCACATTACTACTACCAGTNNNNNNNNNNNNNNNNNNNNNNNNNNNGGTGGTTCCTGCCAAAGTGGCCTTTGACCAAACTGCATGGGCAGCAGTTTGGAACAGCATCTATTATACAGCATTGGGATTTCTACGTCTGGAATCTCCCGCCAACATTTTTAGTGAATTAAAGGCTACATTCTGGCCAATGCTAACTGCAGGGTGGAAACTGTGGCCTTTTGCTCATCTTATTACATATGGTGTGATTCCAGTAGAACAAAGGCTTCTTTGGGTGGACTGTGTAGAACTCATTTGGGTGACAATACTCTCAACATATTCAAATGAGAAATCAGAAGCAAGAATATCCGAGACACCAGCAGAAGCAGGTTCCAGTTCTTTGTTGAAAGGCCCTCCTGAGGAGTAGATTAAATGCTAACAATCAGGAGAGTTTGAAAGATGAGTTGCTCAAGTTGGTTGTAAGAGTTGAGAGTTGAATCCACAATGCGATGGAAAAATTGCAATGCGGAAATTTTATGGACAGACGGGTTATAGAATATACCACGCACATCTTGCATACTTGCATACGTATGTATGAAGATATGAAGATTAGACATACTTTCTGAGATGATACACAATAGCCTTTCTAGTTCCTCCTAACTGTAAAAATCACGACATGGTACTGGAAATTTTCTATATTCCAT

>Unigene2367_All

ATACAATTCAACTATGCAGGGATGTTGCAAAGCACCTAAGATTCTTACTTCCCCAATGCAGTTGTACTCGAAATTCCTAATCTCATCCACTGAAGTCCCACGTATCTCTAAAGAACGCACCTTTGCTGCAGCTTCAACTGATCCAAGCTTGCATTCAATAATGGTACTAGAAACAGTTTTTTCTAATTCATCACACGTGGAGAAAGAAGTTATAGAACAACCAGGACCAGGCATGCTCTCAGTTGAACGAGAAACTTTGGTCCGACTGAGTGGAATGTACCTGCAAAAATACTCCGGATCAGTCTCTTCCCTTATATCATGAGGATGACATGCATCCACCAACATGCGAACCCATGTATCACCCCTCTTTGTGAGAATGATATTCCAGGCATGCGGCACAAAGTCCAGATAACCCCTGACAAGTTCACAGGGTATTGATGGCTCCATTCGGTCACATAGATACTTCATTAGCAAGGCTCTGTGCCTACAAATTCCAAACTGCAAATTTCCTAGAGGAACTATGATGGAATTTCTTCTTGCTTTAACAGAACGCAAAGATTTCTCGCAGAGATCAGAGAAAATTATATCTTCTGAAGTGCCCAAAATATTTTTTGACGCATTAATACCATCATCATTTCCAGTTGAACATGTGCAAACAAATGGCTTACTATAGTTTGAACCAGACACNNNNNNNNNNNNNNNNNNNNNNNNNNNNNNNNNNNNNNNNNNATCAGATACAAAAAGTGCAAGCAGTGATGCAATCTGCAAGTTATCAACAGCAACCTTATTCCTCTCCTTGACAAAACCATTTAATCTCTTCAAGCGGCAGACCAAAGCTTGGGCAGATAGAACTGTTGCATCTAACTTTTCATCCTTCTCCCTGTCCAAAAGAATGACCTCGCGTGAATCAAGATGCAAAATTTGTTCATAGCTCCTTAGTGGCATGAAAGGCCGGTCACGTCCTGCATCATAAAAGCCATCTGGAAGATGGTCCTCAATGCTACAAAATGATAGTTCACTATACTTGCGGGATAAACTCGACCTATCTCCTGTTGGCCTTCGACACTTGAATGGTTTTGGATTATCAAGATCCCTATCAGAATGCCTCTTTGACTTAAAATTGAAGTTGGGCTTTTCTGAAGATGAACCCTTGTCTTCCCCACCAGCTCCATTCCATGTCAAGGCTAAAGATTTTTCATGCACACAACACTGATCTTCCCCACCTTTACCTATAGTTTCAAGGTCACACGAGCAATTTTCTGTGTGAAACCCATTCTCAGAACTGATTCTATCACCCTCATCACTAGCATGTAAATTTGCAGCTTCAACTTCACCAGGATGT

>Unigene2387_All

TGGTGTCCTGGTAGACTCATGAGAAGAGTAGGCAGATAAATAGACAGAAATTGTACTATTTTGTTCAGAGTGCATTACATGATAGAGCAGGCATTTGGGTTGTCATCACTGAACATGGAGATGTAAGGGTCCTCATATCTGGTTACAGTGCCCGTAGTTGCAGTGCTTTCTACATTCCTGACATAGCCTGGAGGTGCCTTCTTGTCATAAGCCTTAGCAACGTAAGGCTGTGCCACTAAATTGTAAGGAACATAAGGCCAAATCTCAGCCTTTTTTCCTGTTGATTTGGCCTTCTTCAATACCTTGTTTGAATCGACATACCCAGTTACAGTAACCTTCTGTTGTTTCCTGTTTATCTCTACCTTTTTAACTCCACTTAATGAAGAAAGGGCATTCTTGACCTTGAGCTCACAGCCATCACAGTCCATTCTGACCTTGAGCTCTACAGTCTGTAATTGCTTCTTCTTCTTGTGTTTATGGCCACTACTTCCCACCAAATCAGATAAATACTCAAAAGTACCTCCAACCCCCATTTTTTNNNNNNNNNNNNNNNNNNNNNNNNNNNNNNNNAGACTTCTGTTGCAATTACAGAATCAAAAGTACTTCCAATGCAAGCAAGTTTTAAGAGCTTGACAGAAAGTAACAGATGGTAATTTCCAGGGAAAAACCAAGGAATGG

>Unigene2431_All

CGGGGAGTTTATATGAAAATGATCCCTTCTTTATGCTCATGGCTATTTCACCCATATAATATGCACAACTCAAAGTCAAGTTGCATTCTGGACTTGCATTACGCTCTGAAAGATTTGAACACGACAAGAGAGCAATGCTTCCCTTACGATCTGAAACCACCGCTGTATCAACATCCATAAGGACACAATCAGCAACTAATCTCTGTGATGGATCACAATAAACTTGCTCCAGTTTTCTAGTATCCTCATGATAAGAATAAAAAAGGATACCATCACGGCAATCACCAACAGCAATTCTAGTGAAATATGCAGTTAATGATATTATTGTAAATCGTGTCCTTGCTATAGCTAACTTTCTCACTCTTTGGGGATTATCATTTGGAAAACCACATACATAAAAAGTACTACCAGCAGATGCCAAGAAGTAATGATCAAGATATGGACAGAGTGCCAGTGCCATTCCAGGCCATTTGGTTGCATATGCCAATCGTAACTGCCATACTTCAGTTTCTTCAAGTTTGACTCCATCACAGCTACCGTCTGGGCTACTGCAAAGACTACTGCTCGATAGCTGTTCCGCAGTATGACCAACAACTTCACGAAATGGTGAAGTTCGTTGAGAAGACGACCCAGCCTTTGAACAGAATGTCATCGAACCACTATCTGAATTTTGCAAGTGCTCAAGGCAGAGGACTATTAGACGACCCTTCGTACTTTCAGCTTCACCACTTGGCATTATAGCTGGACNNNNNNNNNNNNNNNNNNNNNNNNNNNNNNNNNCAACCCTCACCAACTCCATGGATTTTCCTGTTTCTTCAGGTTCAAGTTTAAAAGATGATACTACTGAACCACTGAGTGGATCAACACAACAAATGTCAGATGAACTTGTGTCATTGCTAAGTTCAGTTCTCATCACAAGTAACAACCTACTTTCACTATGGTATAGGACCTTCCGTGGGGTACCTCCAAGATGAAACTTCTGAACATTGAGCCTCTTACTGTGCACCATTTCAACCAAATGTAGACTGTTATCTGCAACAAATAAAATTCCCTTTGGGCATTCAGCCGAACATACAGGCGTGGCATGAGTCGATGGCTGAAATGAGATTGATGTAAACGAGAGGCTGTGGCTTGCAGTTTGCACTAACCATGGTCTATCACTGAGTGCAATCATGTCTGCATCAAGAGAATCACTCAGAGGAACGAGGAAAACAGGAGTAATGCCAATACGACGGGTGGCAATCAATTGTAGATTGACAGGAAGGTCATCCATTGTCTTGCTCATTACGTCAACAGCACATGTCTGTGGGTCAAAAGTCGTTGCAGGCATATTTGATAAACCTCCACCAGTGTTTTCCATGCATAAATCAATTGGAAATCCATANNNNNNNNNNNNNNNNNNNNNNNNNNNNNNNGGCCACTCAAACCGAAGCAGCATTCCATTCCTCAAACCAGAAAGGACATAAGACCGATCAACCAGTACAAGCCTTACATCTTGAGGGATGCAACCACTAATAGCAGTCCCC

>Unigene2450_All

GCCCTTGCTCAAGAGTATAGCAACCATATCTCAATTGATGACACACTGGCATTTTTCTGTACATATTTTGAAATATTTTCTAAGCATAAAGATTACTTGCCAATACAAGAATCTATTACAAATCATAGTATGCAACTATTACAAACTACGTAAGGGTGTAAAAAGGGTATGACCAGTTACAAGAGAACATAAAGCAAAGGCTATAAATGCCAGGTAGGACAATACCACAGATGATCTTGCCATTGCCGGGAACTTGTCCTTACCCCAATTGGACTCCCAGTCCTCAACACGGAATGTAGCTGATGATGATGATGATAAGAGAAGATATGTCAACAACTGATCTAGAGAGAAATCCAGGTAGTAACGGAGCTGATTCTGGGAAACAAGCTTCCCGGTAGCCAAAGAGTAGGCAAGATCATATGCTTGCAGGCCTGAGTACACAAAACCCATCACGTTCACTGACATACAGTACCTGAACTCCTTGTAGCGATAGAAAGAGTCAAGAGCCCAACCCTCATTCTTATCAGCAGCCATGATCGAGAACGAAGCCAAACAAAAAACCAAACCAAAAAACCTAAAACCCAGCAAAGTCTTTCTCTTCACAATTTCCCTCTTCCTCCCTGAAATTGACAAATTCGCCCTTCTCCTGCTACTGCCACCGCCACCAGTAATACCATCTTCAGCTGATTTAACAACCTCATCATCTTGGGCGTCAGACCCGACCTTGGAAACATGCACTGCAGCTGCTGCCGCTGCCGCCGGGGGAGGAGGAGACGGAGGCTTTGCGGATTTGACATCTGGCGATGATGAGAATAGATAACTGTGAGAGATTTGATCTTCCGGGGGAGATTTGTACTTGGATTGTTTGATTTGTGGAGGAGATAATGCATAGCAATGAGACATAGAGGAATGATCTAAGGAACAAGTGGAATCGGGTGGAGAATCAAAGCGTGAGGAAGAGAGTGTAGGAGGGGGTTTAGGAGGTGGTGAAGACAAAGAGTAGTGCATGGTGAGTGGTGGTGGATTTGNNNNNNNNNNNNNNTCATCTACTTCTTGGAGCTGCGGCTCTTCTTCTTCTTCTCCTGGTAGTCGTTGATTATGCTCATTTTCTTGGACGTGGGGGTGCTGTTGCTGGG

>Unigene2461_All

GAAAATAAATTATTGCTGATCTGCTGCTATTATTTTATGTTCAGGTGTTCCTCAATTAATACAATCAATGTGTTTGAGCTATAAGCTATTTACAAGTTATACAAGAAATTACAAAGGCCTTCTGCCACCCCAATTGCACTATCATACTACACACACGAGATCGATTTCCATTCATCAGTGAAAGAATTATGTCACAAGAGATGACAGCAAACAATGCAACCAAGTAGATCTCGTAACATCAGAGCAAGTTGTTTATGATGCATGCTTACAGAGTCTACACTAGACAGCCTGACGAGGTGCGATAGATCTCATCAGCATAAGCAGTAGGAACATACACCGCATTTATCAGTGAACTGTGTGTCTGGCCATAAAATAAATAAACCAATTCTCCTGCTAGTAGCCATATTGAAACACGGATCCATGTGCTGGGCCCCAGATTAACTAACAAATAGGTGTTGACAAGAATGCATGCAACAGGTAAAAATGGAACAAAAGGGCAAACAAAACCTCCTGTGTGTCCAAAGCTGTGCCTTGCATTATCCTGTGCCATACAAGCTAGAACAATCAAACTGCACAACACAAAAGCTCCACCCACAGCACACAATGTGAAGCGCAGAAGGCTGGGAAGGAATTCGTATGAAGCTGCAGATGTAAGGACAAGAACCCCTATGCAAATAATTGTTATACTCCAAGCAGCAATTTTCCTCCTCTTTAGTTGATTTTGTTCATCTTGNNNNNNNNNNNNNNNNNNNNGGTGTGCAATTGATATCCCTGCTTTGTCAACCAAACATTGACTACTGTCACTGTAATTGAGAGAACCCTTAAAGTTTTGAGAGGAAACCCCCTGAATGTCACCACCACTGGATTGCAGTGACATCGAATCAACAGATTCATGAAGCGAAGAAGGGAGTGGCACCTCATCTGGTGGAACATATCTGAGTATTAAGACTGAAACTGCAACAGCAGTGAATGCAAGAAGAGTACCCACACTAACCATCCCTGCTAACTGTGAAACATCCATAAAAAAAGCCAGGGCGGCAGCAAGAAAGCCAATTGCAATTGTGCTCTTCACTGGAACTTGGGTTTGTTCATTAATGTCTGAGAAAAATGATGGCAACAATCCATCTCTAGCCATTGCCATAAACATTCGTGGCTGTGGAANNNNNNNNNNNNNNNNNNNNNNNNNNNNNNNNNNNNNNNNNNNNNNNNNNNNTATTATATTCACTGCCCATTGCATCCCATGAGAAGCAAATGCTGAAGAGATAGGAGTGTCC

>Unigene2469_All

CATCCTTGGTTTTGCATTCAAGAAAGATACTGGTGACACAAGGGAAACACCAGCAATTGATGTGTGCAAAGGCTTGCTAGGTGATAAGGCACGCTTGAGCATATACGATCCACAAGTGACTGAGGATCAGATCCAGCGGGATCTGTCATTGAACAAGTTTGATTGGGATCATCCAGTTCACCTTCAACCAATGAGCCCTAGTGCTGTGAAGAAAGTGAGTGTGGTATGGGATGCTTATGAGGCCACCAAGGATGCTCATGGGATCTGCATTCTTACCGAGTGGGACGAATTCAAAACCCTTGATTACCAGAGGATTTTTGACAATATGCAGAAGCCTGCATTTGTGTTTGATGGAAGAAATGTTGTGAATGTTGACAAGTTGAGGGAGATTGGTTTCATTGTTTACTCCATTGGGAAACCACTGGATCCATGGCTACAAGACATGCCTGCTGTGGCATGAGTTTCTTCTCATGGGATTTGAAGCTAGAACTGAGCAATCTGGGAGCTGCATTTTGATTCTTTTATTCTGTTCTTGTTTCTTTCATTTNNNNNNNNNNNNNNNNNNNNNNNNNNNNTCATAAACATTATAGATTTCCACTAAGATAATGACTATCTCTGTATATATTATTCAATGATTATGAT

>Unigene2503_All

ATATTGAATTCAACTATTTGAATATCAATCAAGTGAATGTTTTTGCACACATCCGTAAGCGTTTAAAATGACTGTTAGAACAATAGTTGGCCCCTTTTCCCCAAACAATTGGTTGTCACGAGGAAGCAATGGCAAGATCATAGAGGGCTTCAACACTTCAGATTTGGTGTCTGGCATCTTCAAAAGCCTGGATGGCGAACACTTCTCATGGTTTCATCAACAATTTTCTAACGTAATTGAGCACTTCATCCACATCCACTGTCCAGGCAACTTCTATAGGGGAATATCCTGTCCAAGGGTTGCTTGCATTCCATTGTTTAAGTCCTTGGTCCATTAGTGTGTGTCCCACACTAATGCCCTGCGTTTCGACTCTCACAACCCCCTTCTTGTAGGTGAAGAGATCAGGGCGAACTAGTGCCATAAAACTCACAGGATCATGAAGAAAAATCCCATATACACCGTCAGACTTCACATGCCAGTCTCTATAGAATTTGCACATATCACATAATATTTGAGCATATTTCCCCTTCGATTGCCTCAATTCAAGAAGGTCGTCATCTGTAAATTTAACTTGGGTTGTGATATTTATTCCGACGACAGCAATGTTTGCTCCAGATGTGAAGACCACGTCTGCTGCTTCTGGGTCTCCATAAATATTTGCTTCAGCAGCAGGATTTACATTTCCCAGTGCAAAGAAAGCACCTCCAAGTACAACTATTTTTTTCACCTTGCTTGCAAAGGAAGAGTCCCTTTTGATTGCCAGAGCCAAGTTAGTCAGTGGTCCTAGTGCAAGTATAGATACTTCACCAGGGTATTCAGAGACCTTGTCAACTAAAAATTCAGAAGCAGNNNNNNNNNNNNNNNNNNNNNCTGTTGGAGAAGGTAAAAAAATGTTTCCCAGTCCGTCAGAACCATGAACAAAGTCAGCAACACGTGGCTCTCCTCCCTTTAGAGGCTCAGGGCTGCCTTCTGCCACAGGAAGACCCGGACACCCTGCGATCTCACACAGAAGCAAAGCATTACGTGTTGCATTTTTTGTTGTGACATTACCAAATATTGTTGTCAAGCCTAAGATCTCTAACTCAGGAGATTGAAACGCCATTAAGATTGCCATGCTATCATCGATCCCAGGGTCAGTATCGATAATGAGCTTCTGGGGCATCGCTTTAGCAGGTGCATAGGCAGCCAAAAACCCGTCGTCTTGAGCATGAAATTTGTCCACGAAAGCATTCATTTTTGGCAGAACAAAAATGAACTAGGGTTGGTTCTTGCTCT

>Unigene2580_All

GGTTCAGAATTAGTGAGGTCGTCTTGTGCTTGATTTCGTTCTCGGTTATGACCGCTGACAAGACTCAGGGGTGGAGTGGCGATTCCTATGATCGATACGGAGAATACAGGTATTGCCTTTCTGTGAATGTTATTGCATTTGTATATTCGGGATTCCAAGCTTATGATCTAGTCCACTATCTGGTTGTGGGCAAACATGTGATCCGCCAACATGTCCGCCTTCATTTCAATTTCTTCATGGATCAGATATTAGCATATCTTCTGATATCAGCATCTTCATCAGCAGCCACCCGAGTTAATGCCTGGCAATCTAAATGGGGCAAAGATGAGTTCACAGAGATGGCTAGTGCTTCAGTCACGATGGCTTTCGTAGCATTTATTGCCNNNNNNNNNNNNNNNNNNNNNNNNNNNNNNNNNNNNNNNNNNNNCAACAATGGTTCAATTTGATCTGAGAGACGGTTTCAGATCGTATCTAATAATGTTGCGGGG

>Unigene2625_All

TGATAACCTGGTTAAGTTGTATGGTTGTTGTGCTGAAGAAAATCATAGAATTTTGGTCTATGGCTACCTTGAGAATAACAGCCTTGCGCAAACACTTCTAGGTGGAAGCCATAGCAGCATCCAGTTCAGTTGGCCAACAAGGCGTAAAATATGCATTGGTGTAGCTCAGGGGCTTGCATTTCTTCATGAAGAAGTCCAGCCACATATTGTTCATAGAGACATTAAAGCAAGCAATATACTCCTTGATAGAGACCTCATGCCCAAAATTTCAGATTTTGGTCTTGCTAAGCTTTTCCCAGCCCATATGACCCATATAAGTACACGTGTTGCTGGAACTGCAGGTTATTTGGCACCTGAATATGCTATTCGAGGCCAGTTGACAAGGAAAGCAGATATATACAGTTTTGGGGTTCTACTCCTGGAGATTGTTTGTGGGAGGGCCAACACGAACAGGCGGTTACCCCCAGAAGAGCAATATCTTCTTGAAAGGGTATGGCAAATGCATGTGAAAGGAGAACTGGTGAGTCTGGTAGACACATCATTGAATGAGGATTATGATGCAGAAGAAGCTTGCAGATATCTGAAGATTGGTCTTCTTTGCACACAAAACATGCCAAAACTCAGGCCAACTATGTCTACTGTGGTAAAGATGCTAAGGGGTGAGATCGATGTGAATGATGATAAGATATCAACTCCTGGATTGCTTTCTGAGTTCATGGGTGTCAGAAGTGACAAAGACCACAAAGATAAAGCTAAAGTAAAGAACTCATCTTACACAGACTCTGCTGGTTCAAGTAAGCTGGAAATTTCATCTTCATCATCAGCAATGGCCACCTCACATGCTACTATGACCTTCAATTCCATATATGATCGAAGCAATTGACTTGTGGCATTTTGAAGTTTCTTTTTTCCCCCATTTTTCCTTTCTTTTCTTTTTTTTTTTTTTTTTTTNNNNNNNNNNNNNNNNNNNNNNNNNNNNNNNNNNNNNNNNNNNNNNNNNNNNGTTTTTGTTTTTATAGAAAATAGCATGGTATATTTTGCTTTAAAGCAGGAATTTCCCCAGA

>Unigene2642_All

GATCTCGGAGATGGATAAATGATCGTTTGTTGATGGAACTTGTTCCTCGCTTGAATGCAGATGAAATTAGAGGCTTTTTTTCTCCCCCCCCCTTGGGGGGTGACGTACCACCCGCCATGACTAATGTGGGAGAGTGGGAGAAATTTAGGAATATAGACATGGACAAACAGGCTCATATTATTGATTCCCTAAGTGGCCCATCCACAAAGAGGAGGGGTCATGTTGATGCTAACAAGGTGGCTGTCTTAAAAGCATGGCATAGAATAGATTGCCGAACAAGAGAGGCACTTCGCCGTAGCTTTCTTACAGATCTCATTGAAGGCTATGAGGCATGCATACGAGCCTTCATTCAGGAGAGTGGAGATGAAGAAGTTCTTTCATTACAAGTTCAAGATCCCTTTCATAGATTGTTGCTGCATGGTGTCTGTGAGTTTTACAACTTGGTGTCAGTGACAGTGTCCGAGTCCAAGGACAAAGAGTCATTGAAGACAACTAGGATAAAGAAGAAGAAAATGGGTGTGGTTGATCTTCCAAATGTCACCCTGTCGCATTTTCTGAAAATGTCCAAGGAAGGTGTTTGGTGATCTAATTGGGCAGCAGGAGCTCCATGTTCTGTATATAAATAAATAAAACTCTTCATGATCCGTGCCGGATCCTGAAATGAGGTTGTAATATTTAACGTGGATGTAACTTTTCATTCACTTCGTACCATTTATNNNNNNNNNNNNNNACCTCTAGCTTCTGTGATATGCTGGAGCGTCTGAATGTACAAATAAGACCTGCAATCAACCTCTTTAGTTGATTGGAAACATATTTGTACATTCAGACGCTCCAG

>Unigene2648_All

GCCATTCTTAGCACTGCGGTTATCTTCGGAATGTTGATAGCATTACCAATTACCCTTGTCTACTACATTCTCCTTGGATTGTGATTTTTCTGAGTGGTATCCCAAAAATAGAGCAGAAATGAAGAGAATTTTGATTGCTGATGATTGTGTGTCACACTTATTGGCTTTGCATAGCTAGCTAGCTGCATTGACTGCATATGTTGGTGTTAGAGGAGGAATTCCCGGGGAAAGAAGAATTTAATACAAAGAAATTTGCCAAAGGTTCCAAGATTCTTGGGGCTAAAAAAATATTTTTAGCTTAGTTTTTAAGCCCTGATGTAAGGATCTTGCACCCATTAAGAGCCAAAATTAAAGTTTGTTCAATCTTCTAAGGTTTTAAAATGAGCAAATTGAAGGCCGTTCTTTGCATAAGGGAGACATGAAGGAAGAAAACACGGGCAAAAGAGTTCGAGCCATTAGAAAGCCATCATCATGGATAAGCTTATTTCTCTTCATTTCCTGTTAGTGCATGTAAAGNNNNNNNNNNNNNNNNNNNNNNNNNNNNNNNGCTACAAAACGACAATGCAAAAGATTAATGAATTGGATCAGAAGATACGAAGAGAAGGAGC

>Unigene2655_All

CCCACCCAGACCATCATATAATAGTTCTCATCTTTTCCCCCCTCAGTTTTCTCTCCCACCTAACCCACGATGGCGAAGTTGTGATGCTCATAGCAAAGATATGTGATGCAGATGCACCCTGCGAAGAAGATGCTCATGCCATGAAGATGTGATGCTGACGGCGACTTCTTCCCGACGCCAACCGACGTTGTCTCCCCTGTTCTCTTCCACTCATTCCCTTCTTGAGTGAGAATGGTGGATTTCCAATTCAAACTCACCAGCTGAGCTTTACCCACTCCAGATCTATAAGCTTTAGCCGCGATACTAGCTTTAATTGCTCAGAATGGAAAATTGTTGCCTACCTTTTCTTCAAAATTTCTAACCTACTTCTCCACTCAGATCTGTTTTTTCTCTCGCTCTCTCNNNNNNNNNNNNNNNNNNNNNNNNNNNNNNNNNNNNNNNNCTCTGATGGCTTCTTGCTCGGGGTATTGTTTCTGAAAGATGAGTACTGACGCAGATCACGGCAG

>Unigene2679_All

TGGAAGAGACGAAGAAGAAGAGGCATGGATTCTCAGGAAGCAAACCCTAATCCTCCAAGTTTCAGAGAGTCTTATCAACGGAGATCTCCAAACCCAAATTCAAGCTGCCAGAGATATTAGAAAGCTCGTCAGAAAATCTTCCGCTAAGACTCGCTCCAAATTCGCCGCTGCAGGTGTCATTCAGCCCCTCGTTTTCATGCTTCTTTCTTCTAACCTCGACGCCCGCCAGGCCTCTCTTCTTGCTCTCCTCAATCTCGCTGTCAGAAATGAACGAAACAAGGTCCAGATAGTAACAGCTGGTGCCATTCCCCCACTTGTGGAACTTCTTAAGTTCCAAAATGATAGTTTAAGGGAATTAGCCGCTGCTGCAATTTTAACACTCTCAGCTGCTGAANNNNNNNNNNNNNNNNNNNNNNNNNNNNNNNNNNNNNNNNNNNNNNNNNNNNNGAGTGTACAAGGAAAAGTTGATGCTGTGACAGCACTACACAACCTCTCATCCTGCCCCG

>Unigene2681_All

GTTTTGTTGAAATTTTCTATGATGATGAAGCTCCAAAGACAAAAATTAGATAATTTAATAGTTCTGCCTCTTAATTGAACACATGCAAGTCCAACAAGGTATTGTAGTGTCCCTTCTCAATTTCCTCCTCTTTCACCTGATGAAGAAGGAGGGTCTCAATTACAAAATCTTCCCAATCAATTCCTTTTGTGACCCTTGTTTTCCTTTTCCTCTTCCGAGTATGTGAATCTTCCAGAACACAAAGGTCACTACCATCACCTGAATTAGCATTCCATGGATGCATAATCCTTTCAATGCGCAACCTTGCAGCATTTATCCTTGCTCTCCTCCTCTTAACTGCCAAATGACCATTAACAAAAGATGGAGGCATAGGATTTAATCCCACATCTTTTTCCAATATTTGCTTCAACAAAAGCTTCTTGCTCAATTCTGATTTCCCATTCCACCATTCTGTTGGATGAAGCTCAAGCCCCATATTGCTTTTCCTCCTATGAACACCTCCATTTTCTATTGCATACTTACCACTGCCACCCTCCTTCAAGAACTTGTTATAAACCATGGACAAGCACTCATGTGAAAGCTGAAGCTTACCCACATCACCATCGCCCATTTTATTCATGCCACTGCCATTTTTGAGCTCAAAATACCGAGAATCATCATACTCCGCACGATCCTCTTCAACACCATATTCAATATCCTTTCTCAAACAGTGGCTAACCACCTCCCCCAAATCAAATCCAACACACTCCAAACTGGCCCCCTCCCTACCACACTTTGCCATTGACTTCATCTCCATATAACGAATCACAAAAGGGACATTTTTCACAATGTTCTTCATTGTCACATCTTTCCCCCATGGTAATGCCTCTGCCACTTTCACAAGCACCTCTAACAATTCCCTATACCTCAATCTACAAGTGGACACTGCTGCGTGCACCTCCCTTGCCACTTCCTCAATCTTCAAACCCTCAACCCCATTCAACTCTGCCACCAACACCAGAACTGCTGCCACAATTGGTGATGGCCTTCGCCCTGTGGATAAAAACCATTTTATTGCACACTGTATCAAAAACACACCTTGTTCTTGCATTCTCTCAACTTTATTGCTCTCAATCCTCCCTGCATTCGACAAATTTCTCACTACCTTCTCAAATGAGCTAACAGGCAACTTTATATTCAAATGATCAACAACCCGCATTATCATTCTACCCAATTCATGAACATCACAACCAATTACATTACCAATCTCTGCANNNNNNNNNNNNNNNNNCCTGACTACAACATAAGCACAGGCACCTATCAAAACTGGGAACCAATCGCCTTGCCCAAACTCACCTTCTGTGATTTTATCAATCATGGATTTGATTTCGCTAACTTTTTGGCCTAC

>Unigene2694_All

CCTTTCAGTCGAGTTACTTCACCCCTAAACTGGAAGAAAAATCGAATAACTGAACCTGTAAGCCATATAGACCACTTTATAGCCTGTTCAAAAGGGATTGAATGTGTTAATTTGATTGAAAATGTGTAAGTCATAAGAGAAGGGAGATTTAACAGAAAACCTTTATTTGAAATAGCAGAAAAAGTAGAGCAAGAAGCTTCCCATTCTGAGGCTGTGAAGGCCGCTTAAGCCGCATTATTTACGTAATTCTTTGATCGGAATCTTACCCCCTACCCCCACAAGGGAATAAGAGCTCCGAAAAAGGATATCTATAGCTGTGGAGAGGGAAGCGCTTAGAAGCTATGCTCTTCTTGAAGAAGTGGGGACATATCCCTATCGAGGGTTTTTTCTTCTTATTTTTTCTATTTTATTTATAAAAGTTTGATTTTTTCTTTCTTTGTGGAAGGGGAGCAGTTCGGTGCAAGCACCAACTGAGAATAGAGACTGACAATAGAGGATTTCCATTCATCTCCCTATCTGCTAAAGAAGTCAGATCTCTGCCACTGACGGCAAGCCTTGATAGGTATGGCAAGGAAGGTCTCTCTTTCTCTTACGAAATTTCTCAGTGTGGAGTGAGGTCTGAATTCTCAATTTAGCTATTGCGTACAATCTCAGATTCTAACCTAGCTATCTAGAAAATATTGGAATGAAAACTAAGAACTTAGCAAAGAGAGATGGGCTCAGGGTCCTGAAGTCAAAAGTGGACACTCTGACGGATATCCTCCTGAGTCTGGAACAGCGATTCGAAATGTTCGGAATGAAGCTGTCAATCATAAACCATAACACTACCAAGAAGAACCTCCTAAAGCAAAGGCCCTTCACGAGGATCGAAANNNNNNNNNNNNNNNNNNNNNNNNNNNNNNNNNNAGATTGGTGCGGGACTCGTTTGCTAGTCTTACGGGCCCACTTTGGCTCTTACAGCTTTGCCTGCATGCGTATTTTCCTGAGATTGCCCCTGAGGGTTCTTCTGTTCCTCCTGAGGTCGATTCGGTAATGGTTACCGTTACATCAACGATCAGCCTCCTCAGAAGAGTTTTGCTACCTGTTTGGACTTTTTCTTCAATCTGGGCACCCTACCAACCAGACGGATTCCAGCCTTTCAAGGACCTTGCTCGAGGCCCGGGTTATCTTAGAAGCTTCTCCCGGCAGACAAGATCGCTCAGAAAAAAGATTCTCAGGCCTGGGTTTCTTAGTTTGTAGAGATATACACTTCGGTCTGCAATACCTCGATGAAAGCCAATCAGGATGTGAACAAATGGCATGTCATGAAGAGGTTGAAAGGCTTGTACGCCCTGGTAGATGCTCGATCTAACAATAAAGGACGGCTAACTGCCTAGAAGTTGTTCGCGGTGCCTCAAACCGGAAGTCCAAAGCTCTTAAACCCTCTGTCAAGGCCAGTGAAGTTCAGTGGTTCTAGATTCCGTCCGGAGAGTCATTTTTGTCAATTTCTTTCTTTTTATGTATTTCTGGTATTTTTTTCTTATTATTTCTAAAGAGTTGATCAAAGTTAAGAGTAATTTTGCAATGTAATTGTTGATTTAGTTTTTATGCTTGGTCTTCAGTGAAAAGATAAGAAGAAGGAAAATGACAATAGCTAACCAACTGAGAAAAATTACATAACAACATGTTCTAACCTCAT

>Unigene2710_All

GCTGCTATGACTTGTAGAATAGCGGCCGGTGGTCCCGCAAGGTGACCGGCCCCCGATTGCAGCCGATTGAAGGATTTTCATGCTCTCGCAATTGTTCGGTGCCACGCCGCGCGCCGGAGTGAAAGGCCTGGAGGAGGCTGGCGACGGGACGCAATATATCTTCAAGCCATCACTGGTCGGAGGCGCGTCGCAATTCGACCTCACCGGCGAGGGACTGTCCTGGCAGGTGAGAGGAAAGCATGGTGTCTGGCCGCTGGAGAAGATAGCGGCGATTCGTCTGTCGTATCGTCCGGTGTCGATGCAGTCGCGGCGATTCCGTGCCGACATCGAAGACACGCGCGGCGAACGCGTGACGCTTTACTCAACGACGTGGCACACCGTCGCGCTGATGTCGCCGCAGGACAACGGCTACCGCGCNNNNNNNNNNNNNNNNNNNNNNNNNNNNNNNAGCAAAGCCGTGCTGGTGGTCGGAATTAACCCGGCGATCTACACGGCGGGGCTTGTCGTGC

>Unigene2713_All

GCTGTTTCATTACTAAACCTTTAAAACACAACACAAAATATTGATTTGGATAATAGATGCAAATGTATAACCCTGAATAACCAGTTAAACACTGGAAACATCACATCACTTGTTACAAAATTGCCTTGAAACACTGATATACATACATATCATTGACACCGTAGACCTAGTCACTAACTAAAATTATACAATTTGCCTCCCCATCTTACTTCCATTCCAACAACCCTAAGCATTGAACATAACCCACAAAAAATAACTTGCAAATAACCAAAGCACCTTTCTTAGATCAGCATCATTGTCTGAAGCATCAGCAGTTGCATCCACGAACAATAACACAACTGCATATTGCATTTTATCTGAGGCTTATCTGCACAGAGATGAATTGGCACCTAGACAGCTGCCAGGATAAAGAAGGCCCATGTACAAGCAGCAAGTTCTTCAAGTATAAAGCTATGATTTACTCTTGCTCATCAATGTAGGGGTGATAGAGACAACCCCAATGAGAAATAAAGTGGCAATGGAGTTGAAGTCGTAAAGATCCCCCAGTGATTCCAATTCTCCGAGAGCTATNNNNNNNNNNNNNNNNNNNGAATGAGTCCGATAAAGGTTGCCAGGAAAAAAATATGATACGGCACATCTACTATTGGTGAAGCAACATTTATGAATGTATTTGGCAGAGTTGGGGTTAGCCTCAAGAAAAGCATGTAGTTCAACAAGCTTTCTCTTCTTCTAGCTACCTGCTCTTGGAAGAAGCTGAGCTTGTCAGGCCATAGAGAGAATACAAGAGGTCGTCCAATCAGTTTTGAAAGGAAATAGCATGAAGAAGCACCAGCAGTAGCAGTGAACACCACCAAAGCCACGCCTTTGAATACTCCAAAAAGAGCTCCAGCAAGCAATGACATAAAAACAGTCCCAGGAATCATAAACGTTTGCATGAAAATGTAGACCACACAGTACCCTACCAGGACCTGTGCAGTGTAGTCACTTGTGTAGCTCTCAAGGTGATCTCTGAGGATTTGTAGATCTTCAAGAGTACGAGGCAGCTTGAGGAAGCTGTAATCGGA

>Unigene2719_All

CTCGAAGGCCTGATCCACGTTTCGGAAATGTCGTGGACCAAGAAGAACATGCACCCCGGCAAGATCGTTTCGACCTCGCAGGAAGTCGAAGTGCAGGTTCTCGAAGTGGATTCGGCCAAGCGCCGCATCTCGCTCGGTCTCAAGCAGACCATGCGCAATCCTTGGGAAGTGTTCGTCGAGAAGTTCCCGGTCGGCTCGACCGTGGAAGGCGAAGTCAAGAACAAGACCGAGTTCGGTCTGTTCCTCGGCCTCGACGGCGAAGTGGACGGCATGGTCCATCTGTCCGACCTCGACTGGAAACAGCCGGGCGAGCAGGTCATCGACAACTTCAAGAAGGGCGANNNNNNNNNNNNNNNNNNNNNNNNNNNNNNNNNNNNNNNNNNNNNNNNNCGTATCTCTCTCGGTGTGAAGCAGCTTGAGGGCGACCCGTTCGCAGAACCGGGCGACGTCA

>Unigene2766_All

TTTATGTTAAGTTGGATACAGGCTCCTGATATCAGGAATGGGTTCTTCTGATTGGTTTAAGACAATAATTAGTTTTAAAAAGGTGAAGGAAGAAAGTTCAAGACAAGCGAAGGGGTCTTCAGCTTCTGGAAAATCAAATGGCTTCAAATGGAAGAACCAATCACGCAAGGAGTCCGCTAGTTCTGCTGGTGGCAATCCAGGAGTTATTGAGGACTTGGCTGCCACTCGGATTCAAACTGCATTCAGGGCCTATTTGGCCAGAAAAACTTTACGCTGCTTGAAAGGAGCTACAAGATTACAGATCTTGACCCAGAATTATTCTGTTAAAAAGCAAGCTACAACTACATTGAACTGTCTTCATACATGGAGCAAAATACAGGCCCAAATTAGAGCTCGCCGACAAAGTATGGTAACAGAAGGCCGGTTAAGACAGAAGAAATTAGAAAATCAACTAAAACTTGAGGCAAAGCTTCATGATCTAGAGGTAGAATGGTGCGGTGGCTCCGTCACAATGGAAGAAATTCTTGCAAGAATACATCAGAGGGAAGAAGCAGCTGTTAAGCGAGAGCGAACAATGGCATATGCCTTTTCTCATCAGTGGAGGGCCAACTCTAGCCAAAACCTTGGGTTGGTTAATTATGAACTTGGCAAAGCGAATTGGGGTTGGAGCTGGATGGAACGCTGGATTGCTGCACGTCCATGGGAAAGCCGAATACCTGCTCAGTCCATCACTCCAAAGAAAGTGCAGAGTAAACAGACAAACAAAGCTGGTAAAAACACCAATTCACCAACTGCTAAAACCCCAGTTTCTTCTAAACCATCTTTGTCCAATGGGAAGGGAAATGATAAGGCCCGAAGATTGTCATATCCAGCTGCTGAAAAAGGTGCTACACTTGAAGGAAGCATTAAAACTGCAGAAGCCAAGGGAAATCATAAGGCCCGAAGATTGTCATATTCAGCTGCTGAAAAAGGTGCTACACTTGAAGAAAGCATTAAAACCGAAGAAGCAAAATCTAAAGGGGAACAGTTAGTATCTTAGTCCCTAGCCAGTACATAAAGTGGAAAGCTTAATCTTCAGCAAGTCATGGTAAGGGAAGATTCTTTTATCCATATGTTTGTTTCTGATTGTGTACAACAGGAGTGGCGTGATTGTGATATACTTCTGCATGCAATGCAGAACATTACACAAAGCCTGGATATGGNNNNNNNNNNNNNNNNNNNNNNNNNNNNNNNNNNNNNNNNNNNNNNNNNNNNNNNAAAAAATTTCTCTGATTTTGTTTTTTTTTGAAAAATAATTTGTTTCTATAATGTAAATAGGCAGAGGCACAGAGCATTGTTGGAGTAATTGCTGGAGTTTTGAGTCTGTAGATGTAAAATCCAAGTTATGAACAATGTCTGAACTTTTTGTGTTTGATTCAAAAAAAAAAAAAATCAGAGAAATTTTTTTGAGGTAAAA

>Unigene2768_All

TGCATATTGCTTGCTCACAAGAATTATCGACTACAAACTATTGCTTTGATATATACTAGAGAATATTTAATAATATTTACAGGTGGATTTGACTTGCTTTCATCTGCAATTGCAGAAAACAAAAATGCCTTTGTGGGGCTCCAAACACAAAAAACAATTACCACCCAAACCAAGTCACAACCCAGGCAGGCCCTACAAGTGCAGCCACTGATTCCTAATGTCTCTTTTACTGATCAAAATTCATGTCAACTCCTGAAAACTTTCTCCTAGATAAATTTGTATCACCTTAAGAACACTAAACTCCTGGAGCACTGCGTCCCATAAATAGATACTTGAGATCTTCAACTGTCAAGCGAGTAGCCGAGCCACCACTTGGATCTTCACCAAATGCAGATGCAACCATTTTTCTCTTCTCCTCCTGAAGGGCTAATATTCTATCTTCCACCGTGTCTTTAATAGTGAGTCGAGTTACAGTAACAGGACGAGTCTGACCAATTCTATGCGCTCGATCTACAGCTTGATCNNNNNNNNNNNNNNNNNNNNNNNATAACATGGCAAGCAGCAACCATATTCAAGCCAAGATTTCCTGCCTTTAGTGACATTAGCATTACAGTTACCTCAGGATCAGTGCTGAAATCTTTAGCAGCC

>Unigene2769_All

ATTTTATGGTTTTACTTTATTTTGCTTTTTTACTAAGATAGGCTTTTCCTTTCATTGAGAAGGGAATTTATTATTTATTTTTAAGGTTATTGTCTTTCACAATTTCTGGATTAGATTTGAGTGCTTTGTTCCTGACGTGGCATGCATAGATACGTCAAAAATGAATACCAAAACGATGCGTCTCCCTCCCCGTGGTGTTTTGACGTCAAATAAGCGCAAGGGTAGGGAGGGTTTTGATTCCCTGAAGCCGTCTCCACCACCAACACCAACATCAACAACAAAATTAGCCAAGCCGAACATATCTCAAGTCGGCGGGGGAAAATTACCTGACCCTGTTTCGTCTAATCAGCTCTTAGCCGGCTATTTGGCGCATGAATATCTCACTAAGGGAACATTATTTGGTCAACCGTGGGACCCGGCTCGAGCCGAAGAAGTAGCTTTGGAGCCGAAGAAGATTAAGCCGAGCCAGAGGTTGAAAGAAGAAGAAACCCAGCCGAATAAGGAAACTTATAAAAGGTACGTGGAGGTATCAAGTTTGTTGAAAGCGGGAGGGTCCCACTTACCTGGCGTTGTCAATCCAAGCCNNNNNNNNNNNNNNNNNNNNNNNNNNNNNNNNNNNNCGCTTGGAGAGATGATCTCATGTGCTAGGGCGTAGAGGTGAGGTTTCGACGCGTGGAACCAAACCATTGGGTGTCCACCTAGAACCATTTTGGGAGATTTTTATTAGGTA

>Unigene2832_All

GAAAACAAAGCCTCTCATGGAAAAGACTAAACCTAATTCCAATGAGCCTAAGCAAAAGGTGAAAATCATAGAGCCGCACAAAGATGACAAACCTAATGAAGAGACAGATGAGAGTAGTGATGATTCTTCTGATGATGATGACTCCAGTGATGATCAGGGCATGACAGCCAAAGGTGAGGATGAAGAGGATGACGAAAGTGATGATGAGGATGATGAAAGTGACGATGAGGACGATGAAAGTGATGATGAGGATGAGGAAGAACCAACAAGATTGAAGGCTGAACAAGGGAAGAAGAGGTCTGCAGAACCTGCTAAAAAAATCCCCATTCCTGATAAGAAGGCCAAATTGGTTACTCCACAAAAGACTGATGGGAAGAAAGTGGTTGGTCACGTGGCAACTCCTCACCCTTCAAAACAAATTGTAAAGACTCCTGCTAACAAGGACCAGAAAAAGGAGCAGACTCTGAAATCTTTTGCGTGTGCATCTTGCAATAGGTCATTTGGCTCTGAAGTTGCTTTACAATCTCATACGAAGGCTAAGCACAGTGCTGCACAGTGAGGGACGGAGGTGTATTGATTTGATGTTTTTGTGTTTGTCACATAAGAGACTGTTAGATCTAGTAAATTTCGAAGGAAAGATTAGTATAGTATATTTTTAGTTAAGCTTTGTTGGGTATCTTTTGTGGATCGCAANNNNNNNNNNNNNNNNNNNNNNNNNNNNNNNNNNNNNNNNNNNNNNNNNNTTGTGTTGCATTTGTTGGCTATAGCTGAAATCTGAAACAAGACAACAGGGCACTCGATTTC

>Unigene2847_All

GATGGGAATACAGCAAATGCCGATGTTTTTTTTTATTAATAACCAACTTGTTAAATAAATCCAAGTATATACAAAACTAAAACATCGGCATTTGCTGTATTCCCATCCTGCAATCTTCAAAATGCAATACATGGACAACTTAGGAAACCTTAACAAGAAATGTTGGCCGTGCTTCATCATCCCAATTGAATTAAAAGTTCAAGCTTCAAAAAGATGAATTCTCGTCTTGGACAGCTCAAGAAACCTAATTGAACAAACTGGGCCATGCTTCATTGTCCACAGCTCCCAGCTGAATTAAAAATTCAAAGTTTCCTAGAAGCCCTAAGATTATATTCATGCTTGATGAGATTTGCCCTCAGCAGCATTGCTCGTACATCTCGATCAAATTCACTTCCAGTTTGTAGAACTCGTTGAAAAGTGGCATTCCTTTGATCTGCATGTCGCGCATAAAGAATTTTGTTGTGCGAATCAATCCGTGCCTGTATTTGATTGTCAGTAATCAAGGCTTCAAGTTCTTTCTCTAGCCCTGCAACACTTGTTTTGAAAGCATTGGCCATCATGTTCAAATCAACAGAAACAAATGGGTGTGTATATTGAATGAGTGCTTTGTTACGGATTTGATCGTATAATGTCTCAACATGATCATGCAAATGAATGTCAAGCAATAGGTTTGCTTTGAGATTTCCCAGGTAATCCAGACACGAAGCATAATGGCTTGAGTAGAAATCATGAATAAGCTCTCTCACTTCAGGTACCAACTCCAAGAAATTGCGGAAGTTGAGATTGTCTATAACTTTGTTCTTCAGCTCTGTCCGCTCAAAACTTGCAAGTGCACAAAGTCCTCCATATGTTGCAACATCTTGAGGTGCAATAACTTCAGTGTAAGAATTTCCCAATTCAGGAGCCACTTCCAGGAACTTCCTAGCAGCAAGCTTGTACTTCTTGGCCTCCAAATGAGCTAATCCAGCAGCACAACGTAGTTTTGCTATTGTAATAGGGTCAAGAGCCTCAGGTGTTTGCTCTGCTTTGCTGACATAGCTTGTGACATGAGTAAATTGGCCCATCTCGATGCTAACAAGAATTGCACTCATGCACATATGAATAATGTGCTTTGATGTAGTACAATAATCACGGGTACGAACATAACTTTTAAAAGCATCCCCAAGAGCACCATGAGCATAATAGAAATCTCCAAAATCGTTGTATCCCATTCTTATGCTTTCCTTGATCAAATTTGTCCTGTAGGCATTAAGTTCGTTCTCGAGCCTCTCCTTCCTCTGCTCGGCTCTACGATCGACCGTCTCGCACCATGCGCTATCCATGGCGTAATTAGNNNNNCATCAATTTTCTGCACAACTTCACGAAACAGCTGCGTGTTCTCTCCCTTCTTGATCTCGTCATATGCCATTCGTAACGCCTCCAACTGCATCGGAGGATTGTCGCAGNNNNNNNNNNNNNNNNNNNNGAAGGCGCGTGATTTTGGTCCTCCCGGTGTACAAACCCGCATATGCTTCGATGTCGAGCTGCTCGCCACTAATAATCGGCCTATTACGGCTAAGACCGCCCCCCCCCTTCCCCTAAAACTCGGCTTCCATGTCAATGACTGAGAATTTGGGGATAGGGCTTTGTGTTGTAGTCTGCTTAGTGCTTAGTGATCTTTTGCTTTCCCTGCCATCTGACCGTACACGCTAGCTAAATTTG

>Unigene2935_All

ATTCAGTTAGGTTGTGGAAATCTGTTGTGGAGTTGTCTAATGAGGAGAATGCTAGAACATTACTTCACAGAGCAGTGGAATGTTGTCCTTTGCACGTCGAATTGTGGTTGGCGTTGGCAAGATTGGAGACATATGATAATGCGAAGAAGGTGTTGAACAGGGCCAGGGAAAAGTTGGCCAAGGAGCCTGCTATATGGATAACTGCTGCCAAGTTGGAGGAGGCAAACGGTAATACATCAATGGTGGGAAAGATTATAGAGAGAGGTATCAGAGCTTTACAGAGAGAAGGGTTAGTGATTGATAGGGAGGCATGGATGAAGGAGGCTGAGGCAGCGGAAAGGGCAGGCTCTGTGGTGACTTGTCAAGCAATTATTAAGAACACAATTGGAATTGGAGTGGAGGAAGAGGATAGGAAGAGAACATGGGTGGCAGATGCAGAGGAATGTAAGAAGAGGGGGGGGNNCTATTTATGCTCATGCACTAACTGTGTTTTTGACTAAGAAGAGTATTTGGCTTAAAGCTGCACAGCTTGAGAAGAGTCATGGGACTAGGGAGNNNNNNNNNNNNNNNNNNNNNNNNNNNNNNNNNNNTATAGGCCACAAGCTGAAGTTCTGTGGCTTATGGGTGCTAAAGGGAAGTGGCTTTCCGGAGATTTGCCTGCTGTGAGGGGGTGTCTTTAAGGCACTGATTCTG

>Unigene2938_All

GCACAAGAAAAACGAGCACTTCACGCTTACGCCTTCAGATCTGTTGACCCCAAAACCAAGCTCAACTTCCACTCTCTCACGCACGCTTCCAACAAATTTCAGAAATGGACGGGGGCGGACCGGCCCACCCGGCGGACACTGAAATGGAGGATCCGGCTGGGCGCGGCCCCCACCGCCGTCTGCCCCCCAACAACAGCAGCAGCCTGTCGCCCCTACCGGAGGCCTCGATAATATTCCGGCCACTCTTAGCCATGGTGGGAGATTCATACAGTATAATATATTCGGTAACATATTTGAGGTCACTTCTAAGTATAAGCCTCCTATCATGCCTATCGGCAAAGGAGCATACGGCATCGTTTGCTCGGCACTGAATTCCGAGACCGGCGAACATGTGGCGATAAAGAAGATAGGAAATGCTTTTGATAATAGAATCGACGCCAAGAGGACTCTCCGTGAGATCAAGTTGCTTCGTCATATGGATCATGAAAACGTTGTGGCAATCAGGGATATAATCCCCCCACCCCAAAGGGGATCATTTAATGATGTTTACATCGCTTATGAGCTAATGGACACTGATCTGCATCAGATAATTCGTTCCAATCAAGCTCTATCAGAGGAGCATTGTCAGTATTTCCTGTATCAAATTCTCCGAGGATTGAAATATATACATTCTGCAAATGTTCTGCACAGAGATTTAAAACCAAGCAATCTCCTCTTGAATGCAAATTGTGACTTGAAGATATGTGATTTTGGTCTGGCTCGTGTTACTTCAGAAACTGATTTCATGACAGAATATGTTGTTACAAGATGGTACCGTGCACCGGAACTGTTGTTGAACTCTTCAGATTACACTGCGGCTATAGATGTATGGTCAGTGGGTTGTATTTTCATGGAGTTGATGGATCGGAAGCCCCTATTTCCTGGTAGAGATCATGTGCATCAGCTTCGTTTGCTTTTGGAGCTAATTGGCACTCCATCAGAGGTTGAGTTGGGGTTTTTGAATGAAAATGCAAAGAAATACATTCGACAACTTCCTCCTTATCATCGTCAATCTTTCAATGAGAAGTTTCCAAATGTCCACCCGNNNNNNNNNNNNNNNNNNNNAAAAGATGTTAACATTTGATCCCAGACTGAGGATTACAGTTGAAGATGCGCTAGCTCATCCC

>Unigene2945_All

GGACAACTAAATAGATAAAAATACTTGCACAACTCACTAAAACAAGAGAGCAAATACAATCATTACCCTTAAAAAATTAATTTATATTTATTACCTTAGAAAGCAATAAATATTTTAAATATTCTTCTCATTTTATAAGCAGCAGCAGGAAATCCACAGCAATGGCATTGTCATCGACTCATCGTTATTCATTGTTATGTTAAATTAACTTGCATTGATTTCCTCCCCGCATCAGATTCTTGATTTTTTCACTTTTATCATCTTTCTCATCCTTACTGATCAAAAGCCACATAAGTCATTGTCCAGTCGTACAACTTCACTTGCCAATAAAACTTGAATTTTGTTTCTAAATTCCATCGGACGGTCAAGCTGCGCCAGGTTCTTTGGAATCTACGGATGCAATGGCAACCTGAAATACAACAGCTTCAGCTTTAATGGCAAAAATTGTCATCCACACTCGAATACAATATCAGCAAAAGATTAAAACCAATGTGCCTCAGAATTGCAGAATGACAGCAAGGCATTAAAGCCATTCAGTCCCGAAAGATGGTAATTTGGTATAATGATATCATCATTGGCAACATTGTATAACCAAACCACCAAAAAAAGCATTAGTAAAGCAAATACAAGTCTCATATCAGGTCCCATGAGCCCTACTGTTGCCTGTTTACTTTTGAATTTACAAACACATCAAACCATCTATTTGTTTGTCCATTTAGATAACAATGTCNNNNNNNNNNNNNNNNNNNNNNNNNNNNNGCTACACAAGGCGATGGAATATGAGTCCTGCTCTTCTCGCGGAGGAGCTCCACCTAAAATTGGTGGAGGACCTCAACGCATCAGAGAATTTCTATCTGACGTTTACTTATGGAATAT

>Unigene3129_All

CTCATTTTTCACTTGCACAAATAGTACTAACTCGAGCGTAACAAAATCAAGTGTTGGAGAGATCACACGATCAATGTGGTGTAAGCCATTGTTCATACTAACGTTTAATTGTTTGATTTGATATTAAACGAGAAAACGCAATATAATTACAGCCATTTGAAATTGAAATTCCACTTTTCTTCACCAGTGCAAGAACAATATAAAATCAGAAAATAGTTGGTTGAAATAGTTTATAACATTGAAGACATTAAAATATAGCACCAGAACTTCTTCTACTTTTTTTTTTTTTTTTTTTTTCTCCCCCCAGAATCCATCCTCCAGAATCCATCAGAACTCATACAGAGAAATTATACACATGGGAGCAAAACCATAATATAGATTCAATAAACATGGGATGGGTCTCAATACAAAGAAGCAATCCAGCACCTTGGTAAAAGCCCTCGCACTAATTAAATAGTTTGTATACTTACAATAGCTTTGCTAATCCGCAAAACAACCCTAAATTGGCAGGTGAGGTTTCCATGNTGGTGGGCCTTCGTTGTCACCAAAAACTCCAGACTACGCACAACCTTGATACCAGCCCAATTCTACAGAAAGCAGAAGTGCTTCTATACATGAAGCATTTTAAGAATGTGTACTTAAACCGCCGAAGAATAAAGGCAGCCTTGGAAACTGGAAAGTCGTCAAATTACAAAGTCTAGGCATCTACATAAATAGAAGACTGTTTCTCGGTGCAAACTTCTATTCACAAAGATTAAGACTAAGATGCTAATTGCCTTATCAAGTGATGATTTCTCTGACAAGCCAATCCAAACTAGGATAAGCGAAGTACAGTATTAGAAATGATGGGATAGCAAAAAGAACCGTAATAAGAATGTACAGGGCCAAAATGGCTGCACTAGCAGGTATTGCATATAAAATAATCAACAGGAATATAATGACCAAAGGAAACTTAGCTGTCAGATGAATGAAGAAAACCAGTGACTTCTGCAAGGGAAGAATTTATTCTTCCAAAATTGAGGATAGCATTGCCCTGATTATGGTCATGGTTTGTTCTTGATGGTTCTGGATGACGTCCACTTCTGATGTTATTGAGACTTGACTCATTTCTGGGAGCTAAATTGCTGTCTGATGACCATAGGGGTTGATGATCTTCACAGAAGGTATTATGGGACTTATGTCCAGCATCATTCTTTCTTTCAACCATCCAAAGTAGAAAGNNNNNNNNNNNNNNNNNNNNNNNNNNNAAGGCGGAAGGATAAGACATTGCACCACGGACAGGAAATAAAGAGCGGAAGTTGAACTGGT

>Unigene3162_All

CAAAATCTTATCTGAAAGCCTGTACAATCAAAATATCATTAAACTATATTAAATTTTTATCATTAACAATAAATCCATAGTTGAGCAGCCAAGCCCAAACGTTTGTGGCTATAGACACCTTGCATATATTCATTGCAATGCTCTATTGCACTCTTGGCTTGCAAAGATCATCTGCATGATGTTTATGCATGACAGGTATCTTATTGTTCAGTAAATTCCTGAGAAATGAATAGAATAACTATACTACAGGTTCGGTACTCCACTTGGGGTGAATCCGGTTCTCTACTGAAACTTGAAAGCCCTCTTACCAGCTCCCAGGCTGTACTGCAATCAATTTCTTGCTGATGAAAATTATTCATGTTATGCGTAAATGGGGAATACTTTGACATGACTTTGTCTGTACGGTCCAGTCTTCACATATTGTTTGAAGCAAATGGATCATAATTTGGATGAAGCACCTGAGAGAGCATGACTGACCAGCTCCCGCCTCAGGATCTTCCCTGCTGCAGTTTTTGGGATAGAATTGATAAAAGCTACTCGCCTTATCTTCTTGTACGGTGCAACCTGTTCTGCGATGAAATCCATAGTCTGAGCCTCAGTAATATTGCTTCCCGGTTTTCTAACTACATAGGCCATGGGAATTTGCCCTGCCTCTTCATCAGGATACGGAATCACAGCAGCATCAGCAATTTCAGGATTGGATTGCAGTAACTGTTCCAATTCAACTGGGGGCACCTGATATCCCTTGTATTTGATCAGTTCCTTCAACCTATCAACAATATAGAGGAAACCCTGAAAGTCAAAANNNNNNNNNNNNNNNNNNNNNNNNNNNNNNNNGTCTTTAACCAACCCTCTGAATCCAACGTTTCAGCAGTTGCCTTCTCATCTCCTACATAACCTTTCATGATAGTTGGCCCTCGCAACCACAGCTCCCCTCTCTGGCCAGCAGGCAAGGCCTCTCCAGTTACAGGATCAACAATCTTAGCTTCCATATTTTCGGCCAGGCGACCAACAGAGCCAAGCATCTTGCACTCTTCAGGATCTGTCATCCTTGTCGCCCCACCTCCAGTTTCAGTCAACCCATATCCCTGGACAATTTCCACCTGTGGGAATTTTTCCTTGAATCTATCGGCGACCTCCTTACCCAGAGGTGCACCGCCGCATCCCAGCAACCAACAAGGAGCTCAAATCATACTTTTTGGTCAATTCCGATTTCACAAACGCCACAATCAGCGGCGGCGAAACCGGCATGTAATTGACCTTGTACTTCTCCACGACTCTCAACATTCCTTCAAAATCAAACCTCTCCATCAAGACCACCGTCTCCCCCAGCGCAGCAGCAGTTAACAACATAATGAATCCAAAAACGTGAAACATTGGCAGCGTAAAAAGTGATACTAGGTGAGGTTCCGGTTCATTCGGGTCTACCCTGTTGTTGTGAAGGACCCCAGAGATTAAGGCGATCAGGTTCCGGTGAGTCAAAGATACGCCTTTGACCCTCCCGGTTGTTCCTGAGGAGTAGAGAATTGCTGCGGTGTCAGACTGGTTCACTTCAACGTGATTGAGTAGGTCATGGGTGGTGGCGTTGGATTGGGTCAACAGCGAGAGGAACTCAGGCGAGTCGATGAGGATGGTGCCGAGAGGGAGAGAAGGGAGTTTATGAGCTGTTTGTGACGTGGCGAATGCGATTTTGGGCTTACATAGTTGGATTTGGTGAGTTACCTCGGACTTGGAACCGAGTGGGTTGGCGGGAGAGATAATAACGCCAAGATAAAGAAGAGAGAAATAGAGAATTGGGACTTGGATAGAAGGAGGACAGAGGATAAAAGCGACGTCGTTGGTAGACAGAGAATAATGAGCATTGAGA

>Unigene3219_All

TTCACGTAGTAGGTATCGATGCCGGGGTTTTTGCCGTCGTCCGGATTCCAGCGATAGACCTTGAACTCGCGCAGCTCGGTTGCGCCGGCCGGCTTCGGCCATTCCTTGCCGCCGCTGATCGTTGAATTCTTCNGTGAACTGAACCATGGTCTCGAACCTTCGCTTGTTCGCTTAACTCTATACGGCGATCAGTAAACGCGCGCTTTCGGCGGAATGTACTGAACGTCGTTGGTCATCGTGTAATCGTGCACCGGGCGGTAATCGAGGGTCGTCTTGCCCTTGTCGCTGCTCCACGACAGCGTGTGCTTCATCCATTCCTTGTCGTCGCGGTTGGGGAAGTCCTCGCGCGCATGGGCGCCGCGGCTCTCGGTGCGGTTCGCAGCCGAGTCCATCGTCACCAGGGCCTGGGAAATCAGGTTGTCGTATTCGAGGGTCTCGATCAGGTCCGAATTCCACACCAGCGAACGGTCGGTGACGGCGATGTCGTCGGTGCCGCCGTAAACCTTGTGGATCAGCTCCTGGCCTTCCTTCAGCACGTCGCCGGTGCGGAACACCGCGCAGTTGTTCTGCATCACGCGCTGCATGCTGTCGCGCAGCTTCGCGGTCGGCGTGCCGCCCGAAGCGTNNNNNNNNNNNNNNNNNNNNNNNNNNNNNNNNNNNNNNNNNNCGGCAGTTCAGGCTGCCGTGCGTTCGGCGTCAGCTTTTCGGCGCAGCGCAGAGCAGCAGCGCGGCCGA

>Unigene3230_All

CCTGAATTCAATTTCCTAAATGGGGCCACAACAACCAATCCTGATGCTACATATGGCTGTGTAAAATCTATAATTTTGGTTCGATTTGTGACTATGGCAATATCACCAACAACAGCATCAAAGAAACCCGTCGTGATCAAATTCACAAGTTCTGTGTAGCTGGGGTTTTCTTTACCATTTCCGAAGGGGATAAACTGATATGGAACAGCATATGGTAATAAACTTACAGCAGCTGTAAATACATCAATGGCAAAACCCTTGAACATGTCAGTCCCTCGCACTTGCGATACAAATTCCTTGAAACTAACTCGAATGGGCACACCAATTCTCAGTTGCTTCCCATTATTGGGAAATACCCATCCNNNNNNNNNNNNNNNNNNNNNNNNNNNCCAAATAACACTGTATAATTGCTGGTTTGCACTAGAACGGTTTGGTGGCCTCATATATAGTGTCTCAGGAGGAAGAGTTGATAAACCAGAATAATTGGACCAGTAACCAATTCGTCTATACCCAGTTCCTATCACATTAACAACATCATATGCAGGAAGGGTCAGAGATCTGTCTGAATCAAACTTTAGTGGACCTGTTAAACCAACAAGATCACTCTTCAATATGTT

>Unigene3244_All

GTAAGATTCATGAATTTAAAAAAAAAAAAAATTCATAGAATACAACATCCAATTTATTGCAGTGAATTTCCTTTTTGATGCTTGATGCTATAGCGATTCCTCACTGTATGGCAATTTGCAAGCAGCAGCACTATCTTTTGACTTGTATTGGCCAAGCATTTCAGCAACAGTTCTGCATCGACACTGGTTTCCAGGGCGAATCACATTCCCGTAAACAGCCATTTCAATTATGTCAAGCTCCTTGTCAGTGAACTTTAGGTCACACGAGTAACCTTTCAATTGAGTTTTGTCCACCTCCTCTTTATCCGACTTATAATTATTGACCCTTGTTTGAAGTATTTGACAATTTTCAGCTGTAGATTCACCGCCTTTGGAGAAGGGTACGNNNNNNNNNNNNNNNNNNNNNNNNNNNNNNAGGCAGCCATGGCAATTGCACAAGCGCTTGCAAACGATGTTGCCAGCAGCGTCTTTACGCCACCTCTCAGGGTGTCGACCAGGTACTGTTTCGGCGTTAGCCCAGCATATCTTTTTG

>Unigene3256_All

TCTTTGTATTCAAGTAACCAGCATCAGCAACAAAGAAACTCAGCTCACATGTCAGCTACTGCACTTTTACAGAAAGCAGCCCAAATGGGATCTACAAGGAGCAACCCGGCATTCAGTACCGGCAATGGAATTTCACTGATGAATTCACCATCTCTCTCAAATGCGTCCAACTTCCATTCTTATGACCAAAGCAAAAGTCCTGAAACCCGCAAGTTTCTGAGCAGGCAACCAAATCAAGCTGCAGAGAACTTTAACGAACTAGCAAATTCACTTTCTCCTTCAACTCCAGCCCCGTTTGTGGGTGATTCGAATTCGAGGTCAATCTTGGGTACTAATAATGCCACGAAGAACTTGAATCATTTAATGATGCAATCAAATAGGAAGCAAAACCATGGGAGCTCCAGTGAAGTGGAGGGAAGTTTAACCAGAGACTTTCTTGGTGTAGGAGGTGAAACACGCAGACCATTCTTGCAACAAGAGCTAGCGAAATTTGCATCGATGAGTTCAGTGATGGATTTGAGCCAATATAGTTCTGGGCATCGATCGCTAAGTTAATCCAATTTCAGAAGGTAAGATTGCTGCTGTATTATACCCGAATTATGAAGCTACCTAGCTTAGNNNNNNNNNNNNNNNNNNNNNNNNNNNCTCCCCTATACAGCGTTGTAGAATATTAGGTGAAAAACAAACAAAACTGATGAAGGGACTGAGTACCGAGTTGCACTCCATGGAAGCAAGCGAGTGAACTCGGTCGAGTTTGAATTGAGAGACTTGTGAGTGGTCAGGGACGTGGGCACCACTGCATGTTGGAAGAGACTTAACAGAAAGAGACAGATATGGCAATCGATCAAGGGAGGCTGCTGGGACCCTCCATGTATGTAGTTTGTAGCTTCTCTCCTTTTTCTTTTTTCTCTNNNNNNNNNNNNNNNNNNNNNNNNNNNNNNNNTCTGCTACTCAGATGATCACAGATCACAGGATTCAGAGCACCCTCTACTGTGATGGCTCTGATTCTTTTTAACACTAGACTAAATAAAGGGTTAATTTTCGTAAAAAA

>Unigene3278_All

TTCGAAGCTTTCACGTTTAAGGCTATTCCTGAAACTGAGGCTGTGATTAGTCCTACCTCAATTCTTGACTCTACTAAATCTCTCTCTCCCTTCAAAAACCCGTTTTGGTATGACATGAACCAACCTAAATCCCCAAGAGTTTTCTCAGAGAATCAACGCTCATGGGACATATTAGACTCAAAAGGCATTGGTGTTGCGCTTATTGATGAAAAACCCAACGAAAAGATCAATAGCTCTTTTTCTGAACCCAGTAACAGAATGGTCTTGTTTGGAACCAGTCTGAGAGTCCAAATCCCACCACTTGCAAACTTTATGCTTTCTCCAACTGAGTCACCTAAATCCCCAGCTGATTTTGGAATTAAAACTAGGAATTGTCAATTAGCAGCATCTGGGTCTACAAATTCTGGAATCCAAACAAACTCTTCTCCTAGGGTGCTCACAGGATGTATATCTATGAGCGAGATAGTCCTCTCTGAGGACTATACGTGTGTGATATCTTATGGTCCTAATCCAAAAACCACTCATATATTTGACAACTGTGTTTTGGAGAATTATTGTTCTTTATCAGATGAGTCCAACGCTGCCCAAAAGAGTTTCCTCAGCTTCTGTCACACATGCAAGAAGAATCTGGAACAGAAAAATGACATTTTCATTTACAGAGGTGACCCGCAGTCAAGAATGCCGGTACCAAGAAATGCAGTTAGATGGGGTGGAGAATTGAGAATTTGATGATGGTTAAACTGTTCTTGATACTCTGCCACCGCTTTTAGAGTTCTCATCAAAGGCCAAATCTTGCTATAACATATTGCACTATGCAGTTGTTCCATTCTTTGACGATTAACAGNNNNNNNNNNNNNNNNNNNNNNNNNNNNNNNNNNNNNNNNNNNNNNNTTAAATTTTTTTTTTTTTTTCTTTTTTTTGTCTTTAACCCTCTCACCTGCAAATAGAGATAAGAGAGGATGAAATATTGTAGAGTGGAATTTGTTTAAGTGATGGCTAATTTTGGTAGTTTCTGCACTTTACCCAAAGTTTTTGGCCTTCTAGTGGAT

>Unigene3299_All

GGGTGAATTAATTAGCATAAGAAAGGCAATGTCTCACACTTTCATCTGTAATAATGTCTACAATTCATCAATGAATAAAAATTTTCCATCTGGCAACAATATAGTTCAGTAATAGAGCATACAACAATTTGCATATGGACAACTATGAAAGAAATGTGTGAAGTATCCTTAGTCATGCAGCTTGATGCATTCGTTTTCACAGCCAAGCAAGCCTTATAGAAACTATACAATTTTAAAAACTCAACCTGTCCAGTGGATTCATAATTAAATACCATTGCCTCAGCACTTGACGAGAAACTCTTCACAAGAAGAAAAATCTGAGCTAAAACCTTCAATCTCCTGAGAAGCAGTAAAATATTCCTACACATGTAGATCAACAGCCAAACCATGAGATGAAAATCATTGCTTATACATCCGGGAATGCACCATTTGGATTTGATCCCCTTCTCCATTCCAAATGTTAATCTTTTATAACCCATCTATGGCCCCTGATCTGATAGCCACCATAGCTTTGATCGCTACTACTTTCTCCCCCACTCTCTCCACTGGTATATCCAGATTTGCGACGATGTTTCTTTCCCCTTGCTACCCCACTGTCACTAAAGCTCCCATTTAAGAGGGGATCATCTATCCCACGAATCACCTTCCGTGTGGGTGAAGATATGCAACTGTCGTCTTTTGTTCTTCTCCTATCCTTGTCATGTCTCTTTATGTTTTGAGAAATCTTATGCAATTTCTCATTAAACTTTGTAATACCCTTTTCCACTGGCTCCACTGCTTGGTTAACAGTAAACTTCACATCACTCACAATATATCCACCACCACCAACAACAACATCCCGAACACTTTCTTTGATGGTCATTCCACTCTTCACATCAATGTCAGGCTGAGGGAGGCGTAACTTCGTGGGTCTTTCACTATCTCTAACCTCATCTGGATCTAGAGGGCAATCAACAGATGCATAATCTCCAAGAACTGAAACAGTTCCAGGAAAGCAGTCACCCATCAGCTCATAAGGCTTGGCAGGGAACACATACAGGTGAACAATAGAAGCAATGCCCATCTCTATACAAATGATGAAGTCCTGAACNNNNNNNNNNNNNNNNNNNNNNNNNNNNTATTGGACTTTTGAATAAACCAAGGGAGTAGAGAAGAGCAATTGCCACTCCTTGCCACCAAGTCAAAAACACAATAGATTTAAATGTCAGGAACTTGTATAATGGCTTTATGTGTGCCAGTTCATCCTTTGTGGC

>Unigene3366_All

GAGAAGTTTAAAAAGGTTATCAAAGACAACTACTACCAGAAGAACAAATTTGATTGTCGACTGACATCAGAACAGGTGAAGAATTTGTGCAAGCTTTTTCGAGCTGCTGGCAATACCTCCGAGTTGAAAGGGTTGCAAAGAAATCCTGGAGCAAAGACACGTACATTTACAGATCGAGACAAGAGTAGAAAACGACATCACGTAGCAGAAATCCATAAATTTGCAGATCAGGACAGGACTAGGAAGCGGCGCCTTGACACCTACGAAAGGGAAATTTATGCATCCCCTGTTGCACCTCTGCCATCGTGTGTACCTTTACCCCGAGCACCAACTCTTGTACCCCCTCCACAATCATATGCTTACGAACGGATCTTGGAAAGGAATGATTATAGAATGGATCCACCTGTTGAACATCAGGACAGACTATTTCGAGATTTGGAGCTGAGGCGTCGAGAGCAGATTAAGCATGCTAGGTACCCATTCCATGAGCATCATGATAGATGGTTTCTAGATTCAGAAATGAGGTATCGGGGGGAGATTGAACACCGTGATCCCGATGTTCTATACAGGGACCATGATCTCTCATATTCTGCTTCCCAGCCATCTGAATACCAACCTTACACAAGGACATTACATNNNNNNNNNNCTCCACATGGTATAGGTCTATATGATCGGTATATTGGTGCTAGATCCTGGTACTGAGAACTCAGAAGATTTCTGAAAGTAAGAAACTCGTGGCAGCCACCTTGAATTTGTGGANNNNNNNNNNNNNNNNNNCCAAAATATACCACTAGTTATGAAGCCTGTCTACTTAGTTTCAGTTTTTGACTGTATTTAGTGCAAAATTTGGACTATCTTTGAAACACGAAATACTCTAGCTTAAATGC

>Unigene3424_All

TTTTTTGCAGCCATGGATGATTTGTTTCCGTCTGTCTGAGAAAAGGCGAATCTGAGCCTGCTCTAGAAGATCTCTCTTTTTTTCCATGTTGCTGGATTTCTTTTCCAAGTGTTCTTATATATATAATTTCTTTGTCTATGAACCGAACACATTATAACCTACAGGAATCCAGCTAATGCTGAGTTTTTGATTTAATTATCCGTTGAAAACTGATATCAATAGCCCACTCTTAAATCATTCTCTTAACTAATTTACTTGCTACTGATTTCTCAAGAAATGGAATAAGATATTATGGCAACAACATGAACTCAACAAGGTTGAAGAGTTTTAAGTTTCAGGTCATTTTTTCCATGCTGCTTACTACTGCTGACTAATATAACATAAAGATTAAACCCAACAGACAAGTAGCCAGCAGAGAACGACCCAAACCAGCTATTGAAGGAGATTTTGGCTTAGTGCTTGGACNNNNNNNNNNNNNNNNNNNNNNNNNNNNNNNNNNNNNNNNNNNNATCTTCAACGTCGAGGGCTGGAGAAGGGGCAGCTACTGGTGGTTGAGTACCATCAGGAAGG

>Unigene3436_All

GGTTCTTACAGAAACCACAAAAAAGAAAAAGAAAATGGGAGTTGATTTAGCCAAAACTCTTGTGCTAGTGCAGCTTTCACTACTATGTTTCACTGTTTTTGGTGATAAAGAGATAGAGGCAGCGTCCCCTGTTCCACCACGCTATCATGTCTCCTCCCCGGCAGTTTCACCAAGCCACCCGCCTACTCACCCTCATCACCACCACCACCACCATGCCAGCTCACCAAATCTTGCACCGTCTCACCACCACCACTACCATGGTCACCCACCCACTGATGCCCCAGTTTACCCTCCCCTCCACTCTCCAGCCCATGCACCAGTCCACCCACCACNNNNNNNNNNNNNNACCCCCCCCTGTTCAGCCTTCCGTGCCCTCTCCGCCGAAAAAACAACCAAAACACCCACAACCACCTGTTAAACCACCGGCCCAACCACCAAAACACCCC

>Unigene3530_All

TTACCAATTCGAAGTAAACTTGTAATGTCCTTGTGTTCCTTTCCACCTTCAACTATGTCTTTGTGAGCATAAACTCCATCATAAACCTTAAGAGTTAGAGTTAAATACGATGGCCCACGAGAACTTGGACGAATGATACTTTCTCCAGGATCCTTGTCAGATAAAAACTCCATTGCTTCGTCAGCTGTTATGTTCTGGAAGCGAGGGTGAACAATCATCCTAGGCTTAAAGTGTTTCTTTGCAAGCTCCTTCTCTTTGCGAGCCTTTTCTTGCTCACTCTGAAGACTACTTCGATCTTCATGGTAATATGGATCAAGGTTTCGGACCTGCTGATATCGATTACTCCTCATCTCTCTCTCTCTACAAACTAAAAACACCTGATATCTATTCTTTTGGATTGATTTGATCTTGCAAGTGAGTATGTCACCTTCATGCAGCCTGTCAGACAACTCAGGCATATCCCTCCAATCATCTGCATAGTCTTCCTTTGTAAGCATACCAGTCAATCCAGATTCCAGCACACATATTGCCCTTCCACCTTGCACCCTGCGAACGGTAGCCTGTACTATTCTCCCTTCAGCAAGGGTATCCTCAGTTTCACCTGATATCATATAAAATTCCTCATCCTGACTTGGTTCTTTATACTGTNNNNNNNNNNNNNNNNNNNNNNNNNNNNTTCTAATTGTATATACTTGAAAGTCTCCTTCTTATTTTCACGCTTCTTATCATGGAGATACACATCAAGATCAAGGTTTTTCAATAAATTAGGCCGGTCTCTAACATGCTCTATTGCCATTTCCAGTGCCTCATCATCATCATTTGTGTCACCATTGTCCATTTCATAAACATCCTTAG

>Unigene3538_All

CCAGAAAATACAATGGAATTGGTATTGTCCCAGGATCCCATCCCAAAGCCAGCAGCAGAATAAGAATGAGAAGAACTCTGGTGGCCATTGTTGGAGTTGACGCCTTCAGCAACATTCTCACTAACTTCAGATATCTGAGAAAGAGAATCTTGTCCGGTAAAGCTCAACTGAGACTTCAACCTTGACACCGTGTGGTCACCAGTTGAGCCATTGTGAGAGTTATGCCCTCCACTTCCTGGTGTAATTGAAAAACCAGTTTCGGCAGAAAGATGGGACTGAAAACCAGCGGGCGAGCTTTTCTGCCTGACCAAAGAACTACTAATCTCATTGAACCCATACGATCTCTGCAACCCACCACTGGCGCTGCTCTTAGGCGGCGNNNNNNNNNNNNNNNNNNNNNNNNNNNNNNNNNNNNNNNNNNNNNNAGAGAAGTACTGATGACTCACTAACGAAGGCGAAGAAGAACCCAAGGCAGAGAATTCACGGTCAGACCCAATGACGGAATCGACAGCCCTGGTTAAAAACGAACCTGGGGCTGAACCATACCTGGTTATAGCAGTCGGGACCGTTGTGTTCTGCGGAGAGGAAGACGGTGGGTACATAGAGTTGAGTTTGTCGGGTTTGCGCCAATGGTGTTGAGCCATTGCTACAACAAAAAGAGAGAGAGA

>Unigene3540_All

GGATGATGAAGACAAAGTGTTACCTTGCATTTTGCTATCTTTTTCATTACGTTTCCTAACAAAACAACAAAAATCAATTATAATTGCAAGGTAACACTTTGTCTTCATCATCCCATCATTGAACGACTTTCTCATCGGCCAGCCACCATGGTCTAACAAGAAACAGAAATAAATAACTAAAGTAAACCCTAAAAGGGACTCCAGATTTACACATCCCAGAAAAAGATGATTTGAACCATCAAAACATGTTTCTTTCGGAGGGTTAGGCCTGTTTGGCACCACAACACCCACAATCTTTCATGATATCAAATGATATTCAAACCCTAAAACACCCAACATGATGGTGGTGATGAAGGGATTTTTCTTTATCAATCATTCCCTGAACATACCTAAACTCCTCCACATGGCAAGGTATGGTGATCGTACCCTTCTGATCAAACCCATATTCTTCTTCAGCTTCCTTTAGTAATTGCATGAACAGTGGGTGATTGAAGTATATCACAGGCACCACAAATCTCTGCTGTTCCTCTCCCTGGCCCACCTTGACTGCCAAACAACCTTTCGGAACGTCTTTCGCTTGCTTCTTGTTATTCCCATGATGATGATGATGATGAAGATGAGGCAAGTGTAGGTGAAAATTCCTTAGACTCTTCTCTCCACTACCCATTATAACGATCGATGACTGCATAAACAGGAACAAGAACAAGAAAGCCAAGAAATTTTTACAAGAAAACAAGGGAAAAAGGCCAAGGATCTGTAGTCATGGCTTGCTTGAAGAGGGTTAGAGAAAAATATCTAAGAGACCTAGGAACGATGGCCATGGTTGATATAAAACCNNNNNNNNNNNNNNNGGGAGGAGGAGAAGATTATATAGAAGTAGGGGAATTGAGAGAGCCCCAGAAAGTGGGTTTTACTTCCACAGCATAACTCACAGTTTAGCGTAAGTAAAGTTACTTGTTTTTGGCAAAAGTAGTTTCGAAGCCGTTTGATCAAAACGGGCAAAGATAACGAGATATGATATAATAGTTAGC

>Unigene3551_All

AATAATAATAACGTATATAAATTAAAATTTAAATACGTTATTATTATTCCAACCCTTTCATAATCATATTGGTCACTAAAAAGGGGCCCAATTATACATTGACCAAACATTTATTAGCTAAAATTATTGGGCCACTGTTACACTAAAATCCAATGTCTAGTAGACATATTTCCACGGTAGGCTAGTACGTTTTCAAACCTATAAACGACATGTAGTTGATTCAAAGACATTGATAGAGCTCCGAATCGAGGCGGGGTTTGATAATCACTTCAACTGGATTAACCTTCGGCAAAGCAATCCCCTGGCCTTCCTTCATATCCACAGGCAAGCCTGCAACCGTCGTCAAATCAAATCCCTGAAGAAGCCGAGCAAGGGTTAAGTGAACCACTTGCAGGCCAAAAGTAATAGCAGGGCACGATCTTCTCCCAGAGCTAAAAGGAATATACTCGAAATTTTGACCCCTAACATCAATATGAGCATGAGTTGTGAGAAATCTCTCAGGTTGAAACTGGTTTGGATTTTTCCAGACAAGAGGGTCTCGTTGTAGCTTCCATATGTTAACTACTAGTCGTGTGCCCTTGGAGACATGATACCCACCCAGATCACAGTCTTCCATGGCCTGCCGTATTCCTGTTAGAGGGCCAGGTGGGTATAAGCGTAGAGTTTCTTTGACAATTGCCTGCAGATATTTCAGTTTGGGAACATCTGATTCTTGCNNNNNNNNNNNNNNNNNNNNNNNNNNNNNNNNNNNNNCAGCACGCTTGGGTTGTTTAGTAGCAGAGACAATGCCCATGTCAATGTAACTGCTGTGCTTCCAGCTCCGGTGAGGGTTAGAATCATAGTAGTTGCCTTGATGACNNNNNNNNNNNNNNNNNNNNNNNNNNNNNNNNNNNNNNNNNNNNNNGCATGATGTCCATCAAGTCACTTTCTCCATCCATGTCTTCTTTCAACAATCGTTTTTTAAC

>Unigene3567_All

ACTTGAGAACTTCCAAAATACATTTGGAATGACCAGACCAAAAAGTTAGATACATAAGTATATCCATCATGTAAACTGAACTAAGAATTCAGTTCATTAAATCAGCCCTACTTCAGATAATACACTGCCAAAAAACAGATCCCATAAGCAACAGATGCACACTAAACAAACAGAGAGCTGACCTCTAAAGAACTATATAAAACAGATGCATGCTCTACGAAGAATTGCTTGCATGCGCAGGTGAGTGGTTAATGACCAATGGAGGACGTAGAGCTCGATGATTTGCGTCATTTTGTTTGCGGGCCTTGTACATCTCTTCTGTCTCAATCACAACTAACCTCTTCACCTGTTGGAGCATTCCCCTAACAGTGGGAGTATTCCATCGCATAACAGTACGGTTGCATTTGCGAANNNNNNNNNNNNNNNNNNNNGTCTGTGGAGTCTCCTGGTTCCTGGAATGCCCCTCACCAATGTTATGTATAAGTTAGGACTCCATGCAAT

>Unigene3589_All

TTCAATACTGTTTACTTGCATTCTTGCTTTATGTACAGACAACAGTTGATAACATCATACTTCCTTAAATACTTTTATATACAGAGGTAGATAATGCACACATATACCCCGTGTGATTCATATAGAATCCACTTCAGACCATTTCAACTACATTCGATCAAGTCAAATGATACATTCGTTAATAATCATACTCTTCCACATGCACACAAGATAAAGCCATCTTCAAATATTGACAAGAACGAAGAACTGAGACTGGTTAGGCGCATGAATTAGAGAATTCCTACTTTATTCTCCTAAAGGTTGAGCCTCAGCCAAAAGCCTAACACGCTGGGTGAGACGTCGCCTGCGATACATGCCAACCAATCGTAGGGCAAGAACAAGAGCAATAGCAGCATAATCAAACAATCTAACAATCCCCAGGCCTCCAGTTGGAATGAAGTTAAATGGAGTGGCAGCATATAAGACACTCAGTATCATAGCAAAAAGGCGCATTTCATGAAGGTAGGAACCAGGTCTATCGGGATCCATCATTTGTTGAAAAATTCTCTTAATGAATAATGGTGACTCTCGCACTTTCTGAACAAGGCCAAGCGCACCTCCAACAAAAAGGCGGTTGTACCTATGAACATTGTCCAAAACTTTGGTAACTTCTTGTTCGTGCTGCCTGTGTAATGATGCCTCTGGTGTCAACTTGGTAATGCCAGAAGTGCACATGGGACACTTGCAAGGCTTAGNNNNNNNNNNNNNNNNNNNNTTGCAGAATGCACCTGCCGCAAAACCAGTGGCCGCAATTGGCTTTGCAAGGAACAGTGAAGGCACCGAAACAGATGGGGCAACAATCATCGAGAGGAGGGCCGCCGATACGATCATCATCCCTAGGGTTTATCTGGATCTCCGCCTCTCTCTCTCCAGCACCACCTGCTTCATCGATTACACCACTCACATCTCCCTCAGAAACGAGGGCGTTAGATTTGCGCATTGTCACGGTTCTCATCGTTTCCCTCATTCCATGCGAGAAACGCGATCTCCATCGTTCCATTTTCAGTTCTCGAACGCCCGAGCAGTGAGAAATGGATTCAAGCCTAATGTTTC

>Unigene3674_All

TTTTTTTTAACTATGAATACTTTTTTCTTATCACACTATGAAAAATAAAGACAACTACAAAAGTCAGGACTCAATTGTCTTTAACATCTTCCCCCTTCTTCCCACTTTCACATATTTCACACTCAAAGTTTCCACCACAAGAATCAATCCAATGAATATATTAAAGTTTACATGAAAAAATAACTGATCTCAGCAAGCCTAAACTCAAGAACCAGCTACATAATTCTTTTTTTTTTTTTTAATGTTTTCCATTTTTGTCTTTTTTTATCTTGGGAGAAAAAAAAAATAAACAAAATAAAGGAAACCCCCCACCAGAAAGCTCACCAGCAATAACTGTTAGCCTATTCTTTGTAGCTGTGCTGATTTGATTGAGATGTATTGGAAGCTATACTCAGCCATGAAATTCTGCTGCAGGATGAATGTCAATACATACAGGAGTAGGAATAATCTCTCAGCGTCCTGTTTTTGCGCGTTTCATTCCACTTGTATTGAAAGGAGAGACACTACCGCTTGGACCAGGAGACTCTTCTCTATAACTTGAATTAAGCAACGCCAGTTCCCGCAACTGCTGCCTCTTGATGAAATCTTGTGATTCATCCACAGGTTTGAGCAGTTCTTCTATAATTTCTTGCGCCTGTCTCANNNNNNNNNNNNNNNNNNNNNNNNNNNNNNNNNNGATGTGAAGAGGATCATTCAGGTGCTCATATCCAGGTCTTCCCCTTAACTTCTCTTCCTTGTCTGGGTCCTTTATTGATCCTTTCCCTCTAATATATACACGGCAGCCCGTCGTAACTTCCACCCGTTTCAGTGAATTGCCTCTAGGGCCCAAAAGTCGCCCAACAAAATTGAAATTTGGATAAGTATCTACTGGAATTTCCAGGCGCAAAATTCTTTTCACAGTGTATGAACTAGGGCTTGCAGGTGCACCTTGCCAGTCCATTGTCATTCCAGGG

>Unigene3675_All

TCGGTAACATACAAAAATATAATTTAAAGTTTCAAGGACAGGTTGCTCAAGAATCTGAATCTGTGAACCCAATATAACTCTGGGAAGACAATTTGGTCAAATTACAAGCAAAATTTTGCTAGGCCAGGGACTTGCTCTTTCGAGTATGCAATATCCAGCACATGACACAGATAACGAAGAGATAACACTTAGTACACAAGTATCGAGTGACAAGAATATGCTAACATAGATTGGTTTTTATTTGTGTGAAAGTTGCTAAAAAAATGACATCTCTGAATAACAAAATGATTTTTCATAATTGAATTATTCCCCAAAAATACTTCACAAATTTGCAGCCATATCTGGTTGAGTGAAATATTTCAGTCTTCTTGAATCAGATGATATGCCACAGTAAATTCATAGCTTGGCAACAGGCATGTGATAGAAAGTAGCACGGGCAGTGTAGACCAGCTTCCTAGTTTTCTTGATTTTGAACTCCATGGCAACTACAGATAAATTTCTTCCGCTCCTCACTATAGACCCATCAACTAACAAAACTTCATTTTGTAGTGCGGCAGAGAGATAAGATATGCTCAGTTCACCAAGGAAGAGATGTTTATCTTCTTCCAAGACAGTTCTAGCACAAGCAATCGCCNNNNNNNNNNNNNNNNNNNNNNNNNNNNNNNNNNNNNNNNNNNNTTTAAAATAATTAGTAACGGCAGGTAGGACAGAAAAGATGCAGGTAACGCGGCCCCGTTGGACAAGGTCAGCCTTGAGGAGAGGGCGAATGAGATCAGAAAAGAAGTTTTTGCAATTGTAGTTTTCAGGGAGAAAGCTAGAGACCCCGCTGTGGTCGAAAAGCTCCATGATCTCCTCCGCAGAACCTGGAGGCAAGTCATTGGAGATTATTGTTTCTGTCGAAACAGATGATGTTTCTTCCGACAGAGACGAAACGGTGGCCGCGCCCGCGGCGGAGCTGGGGATTTTCGCCATCTT

>Unigene3721_All

GGAGTTGCACACAAGGTATATGCCTGGTGGGTTAGAGACCATCTATGATGTTGATCAAAGGGTCTCTAAGAGGACACAAGTGATCCCGCCGCTGCCTGAAGATAGATTTCTTTGTAGTTTTAGCCATATTTTTGCAGGTGGATATGCAGCTGGATACTACAGTTATAAGTGGGCGGAGGTACTCTCTGCAGATGCTTTCTCAGCTTTCGAGGATGCTGGATTGGATGACAGCAAGGCTGTCAAGGAAATAGGACGCAAGTTCCGGGAGACCATTCTTGCTCTTGGAGGTTGGCAAAGCACCATTAAAGGTTTTTGTAGAATTCCGCGGACGCGAACCATCACCAGAGGCCCTGCTTCGACATAGTGGTTTATCATCAGTTACAGCCTCAGCATAACTTAATACTATTCATCTGTATTCTCTCGGTGTATATCGATTCTAAATGATATGCGGGCATGTTCATTAGGCCTTCAAATGCTGTGCACTGATGATCATGGGCTAAATTCAAAGAGAATTGTTCANNNNNNNNNNNNNNNNNNNNNNNNNNNNNNNNNNNNNNNNNNNNNNNNNGCCAGATCTTGAAAATAACAACTAATTGCTTTCCCCTTTGATATCTGCTTTTATTGGTCGT

>Unigene3743_All

ACTTCTTTAGTTCCCTTCCTATTCTTTTTCTTCTTCTTGCCTCACCCTTCCATCGTCTTCCCTTCGGTTCTCTCTCTAGTGTACTTGATTTCAATTTTGTTCCTTAATAGCTTGCCTCCCTTCTTCTTTGGATAAGTAATGACCCTCATGCTCAATGTTTGAGCAGTGACCCACATATTAAGATCCTCGACGACGCCATGCATGCACTCATCCACTCACAGTCCCTCGCGCATCGACACAACTACCTCCTCCATGCCTTCCTTTCTTGAGTGATCATGGATTTTGAGTTTTGGCTTTTGGATTTAGCCATGAGTTTNNNNNNNNNNNNNNNNNNNNNNNNNNNNNNNNNNNNNNNNNNNNNNNNNNGTTCTCAATTAACAAGAGTGTGTCTTGATAAATTGATTTTTTTTTTTAATCAAACAGAAAAT

>Unigene3773_All

TTTGGGTGTTCAAGTTTATAGGAATTATCGTAAGGGTATTCATGGGAAGGGCATTTGTGTGAAGTTTGACGATAGAAGTGGGACTATTTCGGAGCAATCTACTCCAAAAAAGGCAATGCTTATGAGGGCAGAGACGAATATGATGTTAATGAGTCCATTAAAGGAAGGGAAGCCACATTCAACAGGGATTCAGCAGCTTGATATCGAGACAGGAAAGATTGTGACAGGGTGGAAGTTCGGAAAAGATGGGACTGAGATTACAATGAGGGATATAACAAACGATACTAAAGGGTCTCAGTTGGACCCATCAGAGAGTACNNNNNNNNNNNNNNNNNNNNNNNNNNNNNNNNNNNAGATAAGAAAGGGATTGTGCAGAGTATTGGTGGTGAATCGCCAGTTTTGCATTGGAGTCAGG

>Unigene3796_All

GTCAAGGCTCCACGCCAAATTTGACAAATCGGCGGTGCCAAACAGTACTCAAGAGCTTGGGAAATAGAAAGGTTTCCGATCATGCTGGGGAGAAGGTTGGTTTCCTTCTTCAGGAGCTCTTCAGCGTCACAGTTTTCAAGTGTTGCCAAGCCTGTGGATGAAGAAAAAAGCAGGTCTTTTGGTTCCAAGGCTGTTTCTTTTCTGTTGATTACTGTTACTGGCGGTGTTGCTTTGAGTGCTCTTGATGACCTTGCCATATATCAAGGCTGCAGCAGCAAGGCCATGGAGAAAGCCAGTAAAAATCAAGCAATCAAAGATGCTATTGGGGAACCAATTGCTAAAGGTCCATGGTACAATGCCTCACAAAAGGCATTCTGTATCTTGCACATTTCCTGTGTCTGGACCACGTGGCAATGGTGTGTTCCAATTGAAGGCAGTTCGTAATGGAGAGGACAATTGGTTTTCGTTTTTCCTTCCTCGTGATTGGGAAATCCTAANNNNNNNNNNNNNNNNNNNNNNNNNNNNNNNNNNNNNNNNNNNNNNNNNCGGATTAGTCTTTCTGAAAACTACCCATCTCCAGATTGCAAGGCATGCACTTCATGCCCACAGGGATCTCAAAAGCCAGAGAACAAATGAAAAGCTGCACACCATCAAGTATGTGGGAACATTTTTTCATTTTT

>Unigene3923_All

AAATTTACCATAGCCTTGAGAGAAATTGGGACATACAAGGAGGTTCTTCGATCTCAGGTTGAGCACATGCTAAATGACAGATTACTACAATTTGTCAATATTGATTTGCATGAGGTCAAGGAAGCAAGGAAACGTTTTGACAAGGCTAGCCTTATATATGATCAGGCTCGGGAGAAGTTTCTGTCACTGAGAAAGGGCACGAAGACTGATGTAGCTACTCTATTAGAGGAGGAGCTTCATACTGCAAGATCTGCATTTGAGCAAGCTCGCTTTAATCTAGTGACTGCACCTAATGTGGAAGCAAAAAAGAGGTTTGAATTTTTAGAAGCTGTCAGTGGGACAATGGATGCACATCTTCGTTACTTCAAGCAGGGATATGAGTTGTTGCATCAAATGGAGNNNNNNNNNNNNNNNNNNNNNNNNNNTTATGCTCAACAGTCGAGAGAGAGGTCCAACTATGAGCAGGCAGCTCTCAGTGAAAGGATGCAAGAGTACAAAAGGCAGATTGATCGTGAGAGTAGATGGTCTTCCAATGGTTCCAATGGATCTCCTAATGGAGATGGTATACAAGCAATTGGTAGAAGTTCACATAAAATGATAGAGGCGGTAATGCAATCTGCTGCAAAGGGAAAGGTCCAAACCATTAGGCAAGGTTATCTATCAAAACGTTCCTCAAACTTGCGAGGTGACTGGAAGAGAAGGTTTTTTGTACTTGATAGTCGAGGAATGTTGTATTACTATCGCAAACAATGCAGCAAATCATCTGGTTCTGGTAGTCAACTTTCTGGTCAAAGGAATAGCTCTGAGCTTGGATCTGGATTGTTGAGTCGTTGGCTGTCTTCCCATTATCATGGTGGAGTACATGATGAGAAATCTGTTGCTCACCATACTGTGAACCTTCTTACATCAACAATCAAGGTTGATGCTGACCAGTCTGACTTGCGATTCTGCTTCAGGATTATTTCACCAACAAAGAACTACACTTTGCAGGCAGAAAGCACGCTAGATCAAATGGATTGGATTGAAAAGATAACAGGGGTTATTGCTTCATTACTTAGTGCTCAGGCTCCAGAGAGGTGTCTGCCTGCTAGTCCCATGGGAAGTGGTCATCATAGGTCTGCCAGTGAGAGCAGTTCATTTGAAAGTGCTGACTTTGACCAATCTGCCGTTGATGAATTTACATCTGAGAGGAGCCTTGCTGGTGCTCATCATGAGCGACCATCGAGAACCTCACAGCAACGTTCCAGTGCAGAGAAGCCAATTGACGTGCTGCAAAGAGTATGTGGGAATGATAAATGTGCAGATTGTGGTGCCCCAGAACCAGATTGGGCATCCCTAAATCTTGGTGTTCTTGTTTGCATTGAGTGTTCTGGTGTTCACCGTAATCTTGGTGTGCATTTATCAAAGGTAAGGTCCCTCACACTGGATGTCAAAGTATGGGAGCCTTCTGTTATAAGTTTGTTTCAATCTCTGGGAAATGCCTTTGCAAACTCAGTTTGGGAGGAGTTGCTGCAATCAAGAAGTACCTTTCAGGTTGATCTCATCCCCACAGGCTCATACAAGTCTGATAAATCACAGCCACATTTTATCTGCAAACCCAGTCCAGCTGAGTCTATATCAGTAAAGGAGAAGTTTATCCATGCAAAGTACGCAGAAAAGTTGTTTGTTCGCAAGCCTAGAGACTGTCAATATCCTCATTCAGTGTCGCAGCAGCAGATTGTGGAAGCTGTTCGTGCTAATGACAAGAAGTCTGTGTACCGTCTTATTATCAATCATGAAGCAGATGTCAATGCCGNNNNNNNNNNNNNNNNNNNNNNNNNNNNNNNNCTTGCTAAAGTGATGCTACTACAAGAGCAGACAGGCCTTGACCACTGCTCTAGCTGCTCAACAGGGAATTCATTGGACAGGTCCTCTACTAGCTCTTTGAATTTGGCTGGTACTGGTGAAGGTCAGATCTTGGAGGATCTAGATGGGTGTTCCTTGCTTCACCTCGCTTGTGAAACTGCAGATATTGGCATGTTAGAACTCCTCTTACAGTACGGTGCAAATATAAATTCAACTGACTCGAGAGGTCAGACGCCACTGCACCGTTGTATTCTTAGAGGCAGAGCTGCATTTGCAAAATTGCTCCTTTCTAGGGGAGCAGATCCACGGGCTGTCAATGGGGAAGGTAAAACCCCTCTTGAGCTTGCGATAGAGTCAAATTTTGTTGACACTGATGTCCTCACCTTATTGTCAGACTCAAATGGGTGACATGCTCATTAAAGGATAATAATTAAAGATTAATTAAAATGGGGTTGGGGAGTTAAAAATTGATAATGGTA

>Unigene3933_All

CTGCTCACAAATTGTAAATTGGTATCTCAAACGAAAAAAAGTTGGAAAATTTTATAGTACAACACCGCAAGCCTGAAATAGTCATATACAGATAAAGAAAACACAGTGCAACAATCAAGGCCCTGAAAAAGTTCTCTGATAAATCATATGAACATACGAGATCTAATCACCTGATAAAATATACACGCACAAATTCAACTGGCAATGAAACTAAATCTCTTGCTCGCAATTCTATATATGTAGTGTTGTAGACACTGTAATGGTCCATATTCAGACTTACTTTAGGAGTGAAAGAATCATCAAAGAATTTCCAATACGCAATTCTCTCTAATGGGTTTCAAACTTTATATTCAAGCAAGAGCATCACTCATGGGCACGGTACTTGGAATATTTGATCCCAGGTTATTTGGTATTTGGGGTGGTACCTCTTTTGATTCTGTATTACCTGTAGACGTTGGTGTGTTGCTTTCCCCTGAAGTAACTAGATCTGGATTACTCCCTGAGGATGAGATAGGAGCTTTATCAGCAACAGAACCATGAGGTTCACCTGCTTCTGATTGCAGTGGATCATTTTCTACATCTGGTGTAGAACTAGGAGGTGCATTTTCTGGTCCTGCAGTAGTAGTTGTGGCAGGAGTTGATGCAGTTGCTGGTTCAGAATTAGCATTGGGAGGTGCTTCTAATTGTAATGGCTTACTAGGTTGCTGCTGTGAACTTTGTGTGCCAGGACCAGGTTTTTTCTGATTAGTAATTAAAGCAGAAAGAGATGGCATCCCTGGAGGAAGAATTTCAAGTGGTGGTATCTTCCCAGCATCTGCCAAACTTGTAAGCTTGGGTTCCTCAAGGGAAGCCAGAAATGCAGCTGCAGCATCTGTCTTTGTTGATGGGCCATGTTCAAGCTCTTTCTGTAGCATTTTGTTCCAAGCTTGAACCAAGTTTTTAAGTGTTGNNNNNNNNNNNNNNNNNNNNNNNNNNNNNNNNNCTCTGCAAGCATCCCTGTATCCTGCCAAGCCTTTTCCATGAGAACATTGTCTCCCAAAACAGCAGCTGAAAATGCTGCTTCACGTCCCAAGCCAATTGAAATCAAATTGTTTACCAAACTGCTCAGCCTTGTCAACTCCCCATGATTTGCTAGGCGTAGAGCAAGTCCTCTTAACTCATGACCCTGTAAAGCTCCTTTCACAGAACCTGCAGCGGCTAACCTCTTGAGAGCTTCACGAGCAATGTCAGCCTGTGCAGTGGCATCTGCAGCATCAATGAGGTCCAAAAACTCCTTTGCAAATTTCACAATTCCTTGAACAGCATCAACAATATTTTCCTTTTTTGCTGTTAAGTTAAGAATGTCATTCAAACCAAGACCAGTACCATCTTGCCCTACATCCCTGCTGTTGCTCATCGTAAGAAGGCACTGAAGTGCTCTTTTTAAATCATTGCTCTGCATGGCTAAGTCAAACTCCAACCTCTTAGATATTCCAGGCAAATGAAGAGCTTCAGTAGCATAGCCCATCCCCAGCATGAATTGTGCCAAATCATCATGATGTTCTCTACCAAGCCTACTTGCCCATTTAACAGCACTAACAGCATCTCCATAAGCAGCAAGACACCGGCAACGGATTCCAGGATGACTTAAGGATAAGGCATGCGCACACATATATCTATCAATAAGCCAAAGAACTCCATCTCTTACACCCACCACTATTAGAGGTCCAACAGGTCGTTTCTGTTCATTTGGAAAACGAGTAACTGCCACAGATACCCCTCCACCACCAACTGCTATCTCACTAACCCGTTTCTCTTCTATCTCCTTTGGCATTGCCATATCACCAGCATTCTGCTTTGGCAATGTCAAGAAAGGTGGCACGGAAGGAACATGCTGAAATGAAGCTAAACGAACCACCTGTAGCAAAGGTGGTCTTAATTTTAATCTCTCTTGTGTAGCAGTTTGGGCACCTTCTACTGTGATTAGGGCCAGCTCTCCATGTTCAGCAACTGCTCTTGCCTGTGCCTCTTTCATTTTCATCTCTTCTTTCATTTTTCTTGTTTCTAAATCAATAGCTGCAACTCCAGCATCCACAAAAACACATTCAATGGTAGTTGGCGTAGCCACAAAAAGCTGTCTNNNNNNNNGATGCCAAACAGCTCCTGTAGCATGGGAAATTGCAACATCCCCCAGATACCGATATTGAGGCCGCAAGGAAGATATGACAATATACTGCTGATAGGC

>Unigene3950_All

TGCAAAAATACGGCGTCCCTTTCTACTCTTCTGGTTGGGTTCCCTTCAAGGAACTCAGATCCAAGCTTCAATCTCACGACCGCCAACAATCCGACGACAAAGATGAAGATTCAGGCAATGAATCCACTCAGCAGGAGATCTCCGATCAATACTACGTCGTTCTGGCCGGAGGTGGTGGAGAAGGTTGCAGCGGCATCCCCAACGCTATTGTTCTCGCTCAATTTGATTTCGCCTCCAATTCTCTATCTGCTCAGCCTGTAGCTAAGCTTGGACTTGGTTCTGACGTGCCTTATAGAATGGTAGTTCACCCAGATGGAGATGGTCTGCTTTGTGCATTGCCAAAAAGCTGTCGATTTTTCGACTGGGATGAAGTGAAGGACAATGATGCCCACAGATTGAGTCTAAAAGAGTCAGAAAAAGTACTCACTCAACTCGAAGACATTGGACAGCAGTTAGCATTGGCCTTTAACAATGACGGTTCTGTATTTGCTGTTGGTGGCGAGGATGGCTATTTAAGGGTTTTCAAGTGGCCCAGCATGGAAATCATTCTCAATGAGGCTGAAGCTCATGCTTCTTTAAAGGATTTATCTTTCAGCCCTGATGGGAAATTTCTTGTTTCCTTGGGAAGTCGTGGCCCTGGAAGGGTTTGGGATGTGACTTCTTCAACAGTAGTTGCTTCTTTATCGAAGGACAATGATGAGGTTTTTGCCATGTGCCGATTTTCTCAAAGCAGTGATAAGACTCAGGTTCTTTACATTGCTGCAATAACAGTAGGCAAAGGTGGAAGTATTCAGACATGGGACTCAAGCTCATGGAAGAGGATTGGCTCAAAGCATATAAGTCGCGATTCAGTTTCTTCTTTCAGTGTGTCACCTGATGCGAAGTTCCTTGCAATGGGTACAGTTGAAGGAGACGTGCTGATAATAAATCCAATCAATATGCGAGTTCAAACAGTGGTTAGGAAAGCACACCTTGGCATGGTAACAGCACTAGCATTCTCGCATGATTCAAGGGCTTTGGTGTCTGCTTCCATGGACTCGAGTGCAAGGGTGACACTAGTTGAGGAGAAGAAAAGTGGAGGATTGAGCTTGTGGATCATTTTATTCTTAATTTTACTTGCAATTGCTGCATATTTCATGAAGAACGAAGGACTTCTTCCTTCTCTTGGGTAGAATTTACAGACTTCACCAATTAAACGTGAAAATTCTACCCAAGAGAAGGAAGAAGACATAAAAGCAGATGTTTAATATTGTAACTGACAAATTGGAACTTGTCAAGGTCCAATCACGATACCATGTTTTGAATTTAGTCTTGCGGATAATCAACGGTTGCTGGGTGAGCTGGGCTGGNNNNNNNNNNNNNNNNNNNATCAAGCTACCCCAGTTTTATTTCATGTGGGTTGATACTTTTCTGTACATGCTCGTAATCCTTTGTTGCAAATGGATGACTTCTTTTCAGGCAAAAAAAAAAAA

>Unigene3955_All

ATCATGTCATTCCACGCGCTGCTCCGTTCTCAGGACAAAACACCGAAAAATCCTCAAGAGCCAAGTTCCCAACATTTAACCTTCTCAATTCCGCGTTTTCTCTTTCGATATCTCTCCTCTGACTATCTTTGACCAAGCCAAATGGGTAACTGCAGCAGCCACCTCCCCTCCACGTTCAGCGCTAACGCCGCTGACTCTTCCGCCGCTTCCCGCCTCCCTCCTCCTCCAAATGGCATCACGGTCCACCCACCATACGCCTCTCCCCCGCCTCGCCCTCCACCACCGCATTCACTGCAACCCTCCAATCGCCCCTCCTCCACCACTCCCGCTGTGGGCCGAGTCCTCGGTCGTCCCATGGAGGACGTCCGCAACACATACACGTTCGGCCGCGAGCTGGGCCGCGGCCAGTTTGGAGTAACCTATCTCGTCACCCACAAGGAAACCAAGCAGCAGTTCGCCTGCAAATCCATCGCCACCCGCAAACTCATCAACCGCGATGACATTGAGGACGTCCGCCGCGAGGTCCAGATCATGTATCATCTCACTGGTCACAGGAATATAGTGGAGCTAAAGGGAGCATATGAGGATAGGCACTCGGTGAACTTGGTGATGGAGCTATGCGCGGGAGGGGAGTTGTTTGATCGGATCATAGCTAAGGGTCATTATTCAGNNNNNNCAGCAGCCAATCTATGTAGGCAGATCGTGACTGTTGTGCATGATTGTCATTCCATGGGAGTTATACATAGGGATTTGAAGCCTGAGAATTTCCTTTTCTTAAGCACCGATGAGAATTCCCCTTTGAAGGCTACTGATTTTGGTCTCTCTGTGTTCTTTAAGCCAGGTGATGTATTTAGAGACCTTGTTGGAAGTGCATATTATGTAGCTCCTGAAGTATTACATCGAAAATATGGAGCTGAGGTTGATATTTGGAGTGCTGGGGTTATCCTGTATATTCTTCTTAGTGGGGTCCCACCCTTTTGGGGAGAGACCGAACAGGCTATATTTGATTCTGTTCTTCGGGGACATATTGATTTCTCCTCTGACCCATGGCCTTCCGTATCCAGCAGCGCTAAAGATCTTGTGAAGAAGATGCTAAGAACTGATCCTAAGGACCGACTTTCAGCAGTAGAAGTACTAAATCATCCATGGATGCGAGAAGATGGTGATGCATCTGATAAGCCTCTTGATATTGCTGTGTTAACCAGAATGAAACAATTCAGAGCAATGAACAAACTTAAAAAAGTAGCTCTAAAGGTAATTGCCGAAAACCTTTCTGAGGAAGAAATTATGGGCTTGAAGGAAATGTTCAAATCCATGGACACAGATAACAGTGGAACAATCACTTATGAAGAGTTGAAAGCTGGTCTTCCTAAATTGGGTACCAAGCTTTCTGAGTCCGAAGTGAGGCAATTAATGGAAGCGGCCGATGTGGATGGAAATGGAACAATTGATTACATTGAGTTTATAACAGCTACTATGCACATGAATAGAATGGAAAGAGAGGACCACTTATACAAGGCTTTTGAATATTTTGACAAGGACAAGAGCGGGTACATCACAATGGAAGAGTTAGAACATGCCCTTAAGAAATATAATATGGGCGACGCAAAAGCAATAAAAGAGATCATTGCAGAAGTCGACACTGACAATGATGGAAGAATTAACTACGAGGAGTTTGTAGCCATGATGAGAAAAGGCAATCCAGAGTTGGTCACAAACAGAAGACGCAAATAAGCCTTAAGTTTTGCTTTTCTGATGGCTTTAATATTTTGTGAATTTTTTGTTTTTCATTCTGTACTGTAATTGAGATAACATATGGAACATCAAATTTAGCTGGCATTGTATTGCACAGCAAGCCAATCACCGTCTTGTGAGATCCTTCGCGGTGTGCCATGCATGCCCTTTGCACAGAGAAATAGGCTTCAATGGCATTGCATTTTGGGAGATTTTTCACCATTTTTGGTGACACATATATTTCTCAAATGCCAAAGAGGCATGAGAAAACAACAAAGGAATGGCTTGAAGCCAATCACGTGGAAGGTTNNNNNNNNNNNNNNNNNNNNNNNNNNNNNNNNNNNNNNNNNNNNNNNNNTTCACCAATGAAAGGGGCCCTGGGTAAACGATGACAATGCTAGGAAGTTGTCTGAAGAGAAGCTTGACATTCCCATCTATAATGCAAATTTGGAATTGGAGTCTCAAGGCAATCAACGAGGAAGGTAGAATCTGATTCCTGAATAACAGATATAGCTCCCATGCTGCTTGCCAACTGAATAGCTGTTACCAATGCTGCTGCTTCACCAACAAAGACCGAAGAATAGGCCACCTTACGGGTTACCCCATGAGCCAGCTGTCCTCTATAGTTTCGGACGATGACTGCTGCATATGGTTATATAGTTGATGCTCCCCGTCTATTCCTGTTTAATTTGTCAATGACAGAAGCAATGAAATTTTTAGGTGACGAGCCAACG

>Unigene3969_All

AGCCATTCAACTTCATCTCCAGTTTTGTATTCTGGAAGTTCATCAACCACTTCACAAAGCAGCTAGGAGTTCCAACAAGCTCTTTCCTCAATGGGTCTTCTCCTAAAGATCTGAGAATTGACACCACTGCAGTAACCATTCCTGGGTTGGGTGGTCCAATTTTAGCACTCAAAGAAGATTGTAATGGACACCAACATTGCTCCATAGAGTCCATCTGAGTTTTATCCACATTTATACCTCTGAACTTCAGAAGACTCAAAAAATCAGACCAAATATCAGCATTTTTATTTTCAAAAACACCAGAACCTGATTGGACCAGTACCTTTACCCATCCTTGGTGATTGGAGTCAAGCAAGAGTGACTCTAAATTTGGGAAATGGATGTGTAAACATTGGAGTATGATGGCAACACCTGCCGGCTTGATNNNNNNNNNNNNNNNNNNNNNNNCTGCCATACGCTGTGGATCCTGGAGCCGTCTTGCAAAGACATCAGCAACTCTAGATAGCTTGCTTAATCCTACAACCCTCTGACCAGATGGGACATAACCTACATGACATTTAACCTGAAAGGGAAGCAAGCAAGACTCACAATATGAGAAAAGGTCAAGATCTCGAACAATCACAATCCCACCAGCTCCTCCGGCATGACCTACTGCATTATCCAAACCAGCTTCAGGGAATAATGCACTGTGAACAATGTCCTTTGCTTTCTGATTATAACCTCTGGTTCCATGGAGAAGGGCCTTGGCAACACGAAGTGGGGTCTTTTTGAGACCTTCTCTATTAATATCCTCGCCGAGACCTTGCAATAGAACTGTCACAGCATTCTCAATAGCTAAAGTCTCTGGTTTTTCTTCAAATCCCAGCTCAAGGCAATCGAATTTCACTCCATTCTCAAGCTCCGCATTGAAATGCCCCTCATCCAAGGCACCCATCGACAGACCACAAATCCCACCAACAATAGCAGTACAGATCCCAAAGAAGAGAATAAAACAGAAGTTACGGAATCAAATAGCCT

>Unigene3972_All

ATGAGAAGTATCATATTGAAGGATCCTAAGCTTCCCTTCCTTAGTCCCATATACAAGCCCACCTCCAATTGAGGGATGAAAGCATGCAACATTGACCTCATCTTCTGCACTAGGAAGAACTCTTACAAGTTTCATATCAGAAACTCGGTAGACCTCAAGGATAGTGTAAATCGGTACTGTTGTCTCCCTGTCAATGACAACGCTTTTAAGTAGTGAACTGTGCCGACGACCATAGGCAAGCAGTAAGTGCTCTGATGTGGGAGAAAACTGAATTGAAGTCAAACAGTGAGCAGCTCTTATAGCCCGGGATGCAAGCACCAAGCCAAACGTTGCCTCCTCAAGGGAATATATCCGAAGCTCATACATAACTTGATGGGCTGAAATTGGATGTCGTGTTGGGGATGTCGCAGCCCCGGCATTATCATGAAGCACCTGGCCTTGTAAGCCAGGCTCAGCTTCCGGATGAGGAAGCAAACATGCAACACAAGCTGCTAAAAATCTCCCACAAGGTGAAAAATGAGCACCCATTTCACTGCAAAGTACAGCATGTGGTATGGTTAGGCGACATCGCTTGGCATCAAGAGGGGCAAAGGGATCTTTTACATCATATGGCCAAATTCTTAACTTGACAGTGCAAGGTAATTCTTCGGCAGCAGCTGCAGCCAAAGAGGTAGCAAATTCAGAGTGGATTCTACTAGCAACAGGCAAAGCATCATTTTCACTACGTCCCATGCTACCAACACCAGACCCAGTTGCTGATGTTTGGGATCGTGAAGATCGATAATGCAGACCAGATCTCTCACTGCCCCTAGATTGGCCAATATTGATTGGAATTACAGGGGATGAAGCATGATNNNNNNNNNNNNNNNNNNNNNNNNNNNNNNNNNNNNNNNNNNNNNNNNNNTGTGTGGCGCGCCGTTTGGCTCTGGCCAACTAACCACCCATGCAAAAAAGGAACTTCCCAGTACAGTCTATCACCAAAGGTGTACTGGGAAGTTGGAATTATTTCTCTCTACAGTCTGCACTTCCGCTGTCTCAACAGTATCCATTGCGAATTGAGGAGCTTCGTTATCAATGTCACCCATTAAAGAATCAGTCCGAGTTTCTTCTGGTACAGGAGATGAGCTATTAGGTTCAATCGGAGACAGCAGAAGTTCATACTGGCCTGATGGAGTTGAATATGTCAGAAGCCGCACGGAAGTGAAAGGATCAAGTCTTTGTTGCATACGACTTGAACCAACATCTCCATCATTTTGCTGCAGAGGTATTCTACCAACATCTCTAGAAAATGAAGGCTGTATAAGGAAGGGCAAGGACATTAAAGGCAGTTCATCCGCTAAGCCAAGGCGGCCATCAGAATGAGCATCTGCCAAATATACAGTAGGAGGAGGATAGCGCAGATACCCTGGAGAAGTTGCGAGCGTCATTGAGGAATCAGATGAATCGAGGTCATTTACCTCTGCTGTTAAGAGAAATGGAGCACCATGTGGGTGAAAATGCACAGCACGAAGTGAACGACGTGTCTTCAGCACAATAGCTGGTGAGGATGTCTCCCCTCTATTGTTATATTGCCATATGTATAGCTTGTGACCAGAAGCTACAGCAAGAAGCTCCCCATGAGCATGAAAAGCAATAGAAGCAATTGGACGGTAGAAGTCACGAGATCCTATACACTCTGCAGCATTTGCATTCCACAGACGAACTTCATGATCCAAACTTCCGCTTGCTAGTATTTCTGGGCATAAAGGATGAAATCTAACCACCCAGGGTGTCCTCCGATGACCACTCAAGACCTTTAAGCATTTCCCAGTTCGGCAATCAATAATCTTTACAGTATGATCTCCATGCGTAGAAGCAAGGGCCTTCCCATCAGAACTGAAGGCTGCAGCTATGGTTGATCTTGGAGGAGGCAACAGCGGACAATACTTGGCTGATAAATGCAGCAAAGATTCTGCCTCCGCCCATGAAACGAGAGCGCGCCTGGCATCTCGGGCTGATTCAGAAAAAAGCTCAAGGGAATCCAGACGCTGATCGGAGGATTCACCCCACCAATTTTTCGAGGCATGCTTTGCTCGAGGGCAAACCTCTCTTAGTGCCAGCAGTCGAAACACATTATTACCACTGTGACGGCGCGAAGAGGGAGGGTGAGGATGAATGCGAGGATGCGAGCTGGAGGTAGATGGCGGATTAGATTCGTCCACCCAAAAGGATCCCCTCATTTCCACAACATGAAAACGATCATGGTTCGGAATTTTCCGTCATTTTCCCGAGAAAATTTTGGAACAGGGGAAACAGACAGTTTTTCCGCTATAATTCTATACCAGGCACCTCTGAGAAG

>Unigene3977_All

GACAATCCAAAAAATTGATAACAATGCCCAACCAGCACCCAAGATGTTACCATACAGGCAAAATGTATCAAAAGAACCAACTGCTAAATTAAAAGGGGAAAGACCTTGACAATAGATTTATATCTTACAAATTGATAAATAAAAAATGTAGTAAATGTTTGAGCTACTCCAACCTGGATAATCAAAATCTCACCTCTGGGGAACTACCTCTCCTTTCCAGTAACTTCAGCATCATGAATCAGCACTTCCATTGCAATGTTCCTCATTGTTTGTCAGTAAGCACACAAAGGCTTTGATAATTTGTATGCTGGCAATTGGTAAAGATACCATTCCACCCCAGAAAGCTTGGACTTTCCCATAAAAATGTTCACAGGTCACAGCTGCCACATCCCATGGAAAGAATATCCTTTGAGTCTACTGTTCCCTTAAATCTCTGTAGAAGAATTTGGAGCGCTCATGCAAGAACCTAATGAACCTCAAAATGGAGCCTCAAGTGATTCATCCCAGACTTCTGCACTTCCATTAGATGCATCTTCAACGTCTTCCTCCATTGACAGCCTAATATACTCCCTATGTGCAAAGCGATCCCACTCTGTCAATGTTGGATTCTCACGTTCCTGACGAATCAGAAATGGATCACGGGTTTCGAAGGCCATAAACTTGTTAAGGTTGAAAAGGATATTGAACACACTTCCTGAAAGTTTACAACCTTTTAAATCACTTAATGTGATATAGCTCTCGTTCTCTGGTCCAATCATGTCAATTATCTGGCATAATATATCTTCAAACAGCACAGGCTCTTGGGCCATGCATTCCATTCGATGCAACTGCTCTTCATAAAAGAATTGCATTTCATTTCGTGTCAGAACTCCATTTCCATCCAAATCTATGCACTTGAACCAGTACTCAAGACTAGGTTCAGATGACTTATCCTCCTCTGACAGTATGAAGTAGACAAAATCTTCATATCCCNNNNNNNNNNNNNNNNNNAGTAAACTTTCTTGGAACCTGGGAAAATATCCTATCAACAATCCGGTATGTAAGTGCATGGTTACCATATCTGATCAGGTTCTCTTTGTCGATCAGAAAATCATGATCCATGTCCAGTTCCCAAAACTTGCAGTATATCACATAAAAATGTTCATAAGAAAAGTACCTCAAAACCTTATTTATGTCCTCTTCTTCATCTGCATGCTGCATTGCATCAATCAAGTTTCCACGTTTCAGCTCCCTGAGGGTAAGATGACCATTTCCAGATCTATTTATGTAGTAAAATATTCTGTATATTACAGTTTCAGCATATCTTTCCTGAAA

>Unigene3981_All

ATGGCAGCCTCCAAGAATCCATTTATTTTAGGTAGCCAGGCAGCAAAACGCCTTCAGCAATATGTCTTCAACGTCTACAAGCGTTTGGTTCAAGCTAATGTTTTGAGTCATGCCTGGTACATATAGTTCATGTCAAAGACATTACAGGACCTGAGAGGACTGCTCTTACCCTCATTAATCCTTAACAAGATGGTCCATTGTTAACCTTGTTCTCTCTACTGCACGCTGAGGCCTTATCAAGAGGCCATGCTGCTCCTGCCATCACCGCTTCAATCTCCCAAACCTCTCGGGGAACATTAGTCATGTTATTACTACCATTGGCAGTGACATGCACATCACTTTCAATTCGAACTCCACCAAAACCTTTAAATCTGCCAATTGCTTCGCGATTGAAAAACTTGGAAGTATTCGAATTCTCCATGGCTGGATCCAATAAAGCATCTATGAAATAGCAACCAGGCTCCACGGTTATCACCATTCCCTCCTCAAGTTCTCGGGCAGTGCGTAAAGACCTCAATCCTGGTTCCTTAGACCTTTTCGGTCCCTTTAAGTAGCCACCAGGATCATGGGTGTCAATTCCCAATAAATGCCCCAGGCCATGAGGCATGAAAACAGCACCCAACCGCTCAGTCATCATATCATCTACATTGCCAACAACAATGCACCCTTTCTTCAATGACTCCAGAATTATTTTCTCTGCTAATTTGTGCATGTCTAACCAGCTAACTCCAGGTCTCATTGCAGATATGACAGCATTGTGAGCATCAAGCACAGCATTGTATATGAGAGACTGATCACTTGTAAACTTACCATTCACAGGGAACGAACAAGTTATGTCAGACCCATAAAAATTATATTCAGCTCCCATATCAAACAATGCCATATCTCCATCTTCCAAAGTCCTATCATTTGGAGCTGCTGCATGCCCATAATGGAGTACAGCGCTATTTTCACCAGTAGCACATATACATGTGTATGAGCAGTGCCTACAGCCACCATACATGTATGTGTGGTGAAGAAATATGCTTTCCAACTGATACTCTTTCATGCCCACCTGAATTTTTCTCATAACCTCCACATGTGCTTCAGAGCTGATGTCATTAGCAAATTTAACAACAGCAAGCTCCAGATCGGACTTCAAAACACNNNNNNNNNNNNNNNNNNNNNNNAGTCAAATCTGATTCAAACTTTTCTATCCCCTCAAATCCTGCTGCATTAGAGAAGTTACTGCTGTCAGTATTGAGTCCATGCAAGAGAAACAGTAGAGGTTTGTCAACTTCCTTGCTGATTTTATGCAGGGCTCCTACAATTTCATCTGTATAATAGACCATGCTAACCTTGTACTTTTCCTGAAAATAAGATAATGGCTTTATTTCTCCCAACCAAACAGCGTAATCAGCAGGTAGCCTAGGAACAAACAGAATAGAATTCCCTGTTGCAACATCAATTGCTCCACAGAAGCCAGGCTCCGTCACTCCAAAAAGATAAGCAAAATAACTCTCCTGCCTGAAGAGTTCGATGTGATCAGTACAGTAGCGAAATTTTTCCTCGCCTCCCTGAAGAAAGACGAAGCCGTGGAGAGGACGGGAGGTTTCGATGAGGTATTGACGGAGGGATTTAAGGAGCTTTTCGCGGTTGGTGACGTAAAGCTCCGTTGGGACTTTAGGGGGAGA

>Unigene3992_All

GAATGTGGCACAGACTGTTGATTGGCTGGGTGAGCAGATGAATTTATTGGCAATGACCCTGGAAAAGCACCAGCAGGAACTGAATTTGGTATAAATTGTTGGTTTCCTAGGGATCGTGGGAGGGGCAATTGTTTATCAGGATGCTCTTGTTCTGCTACACTTTGCCTATCAAGCTGCCATTGTGGAACATTANNNNNNNNNTGTAAATCCTGCAGCAGCCAAGGAATCTGCTGAAGCAGTAGTTAATTCTTTTGGAGGCCCAGGTAAAGAATTTCCTGGTTGTCCACCAACCATCACACCTTCAAGCGCATAAATGGTATCTTGATCATAAACTTCCTTGGAAGCCCAGAACTGCAGAATTTTCTGTAACCGTGGCTGATTTCCATCCTTATTTTGAGGATTGTGATACACTCGTGCAAGCATGGGACCAAGAACTGGCTTGAATGCAAGGGCCTCATTATCAAGTTCATGGGGGTTGATCCTCCTCTGTAAACTGTCAAAAAGAATATCATTAGCGAGATAAATAATGTGCAGTTGCCTCTCAGAATCATCTAGAGCAAAAACCCTGTCCCTTAGCGCCTCAGCAAGAGCAGGTGCAAATGGAGACCTCTGCATGAACCAAGTTTTGGCACCTTTAATAGACTCCTTGGTTCCATTGAGACTGTTAAGCACATTGCTCAGCTCCAGTGCAACATCAGGAGGAAGTGGACCAGAAAGACCTTTGAAAGACTTAGAAGACTGTTCAAATTCTAGATGACCATGAGCCCCANNNNNNNNNNNNNNNNNNNNNNNNNNNNNNNNAGGAGGAAAAGGAGGTTGGTGCATTGGAGCACCGACCATTGAGGCCGAAGGCCCAATAGCAGGGCTCATCATTGTGTTCGGAGGGGGATGCATCATCGGCATAGAAGAAGGAGGGAAAGGATGGTTGAAGGGCCCACCTGGGGAATGTGTAGATAACCAAAGCTTGTAACGATAGTAGTTATGGCCCTCACCCCCAAAGAGGAAACTATAAGCAGGATTATCCTGCTGCTTTTCACGGATCATGGCTTCAAATTCAGGGCCATTCTTGGTGGCATACTCAACAAGTTTGTCGATACGTTTTTGGAGCTCTGGATCAGATGGAGGAGGAGCTGGAGGTGGAGCAGAGTCATAATGGCCATGGAAAGGAGAAGGATAGAGGTGAGGGGGTAAATGGGGAGGGAAAGGAGGAGGAGGATGTTGTTGATGAAGAAGCTGAGGATGGGCAGAAGGTAGAGAATGGGGCTGATGTTGTTGCTGCTGCAAGTGGTGTGGGTAAGGGAATTGTTGCATGGAAGGATGGGGAGACATAAATGAAGGAGGAGGAAACTGCTGATGTTGAGGATGAAATCCAAACTGTTGCTGTTGTGGTTGAATATTGGCAGCTTGTCGCTGATGCTGCTGCTGCTGGGCAAATGCCATAGCAGAAGCTGCTGCTGCATAATCATGAGCTTGTCGATCCATCAACCTAGAATGCAATTAGAAGCAATGATTCGATTGCTAAGCTCTTCTGCTTCAATTTTGTTGAATAGCCGACCGATTCGAGCGGTAGAGAAAATGACTGGACTGGTTAAAGAAGAACCAATAAATCGAGTGCCGCGTCCATTGAAACAAGGCGACGGAACTCGCTCTCGCTGGGAAAATAGGAAAAACGTCCCTCGTACTCCGGACCCCTTTTATCCAAATATGCTTTTACACTATGGGTGAGCATATATTTGTCTTCCCCATGATGGTTCTTTGGCCGTGGCGTCGTTCTTGCTCCCTCCACAAAGCACTAGGATT

>Unigene4007_All

CTTCACTCATTTATCTGATCTTATTTTTAGATTATAGCAAAATTCAGTTCTGCCGCTCATGGAGGACGAAATTCCTAGTTCTAGCCCATCTCGCCTTCCCACCGATCGCATCAAGCTCAATGTTGGAGGCAAGCTCTTCGAGACAACCATATCTACGCTGCAATCCGGCGGTCCTGACTCACTCTTAGCGGCCCTCTCCAGCCGCCACACTCATGACCCAGTTTTCATTGACCGTGACCCGGAGATTTTCTCCGTTTTCCTTTCTCTGCTGCGTTCCGGCCGCATACCCTCTACCGCTCAACGCTTCTCTAAGCAAGAGCTCGCCGATGAGGCTGTCTACTATGGCATCGAATCGCAGCTCCGATTTGCAATGTCTCCGCCTCCTCTCTCCGGCATCGACGCGTCCCTTCTCACCATCATCCAACCCGCCTCAGACGGCCTACCCTCCACCTTCACTGCTGCAGCTGGAGACGGCTCCGTCTGGATTGCACACGGAGGACAAATTTCAGCTTATGATTGGAACCTCAGCCACACTGGCACCATCCGTACGCATCTTGATGATGTCACCTCAATCCGACGAATCTTGCCGGAGGTTGCAGCCGTCGGATCAAACTCCGCTGCTGGACTCCATTTTTATGATTTCTGTAGCGCTAGTCACCTGGGGTCAACTCACTGGACTGACCCGTCTGATCCTAGAATATATAAAGCTCGGATAACTTCAATCGCTGACTCATCGAATCAAGTTTTTGCTTCCTTCGATTGTCAACACAGAGAGAATTGCATACTAGTAATAGATAAATCAACACTCCAGATTGTCTCCGAGATTGCGAGGCAATCTGGGAGCTCAGCGAAGAATATGGTCCCCGGGAAGCTGACGTGGCTGCCCGATACCGGGGTGATAATCGGAACCNNNTCGGAACCGCAGTCACGAGCGGAGCGTTTGGGTACTCGGGATATATCAGAATATGGGACCCAAGGGCTAGTGGTGCGGTGGTTTGGGAGACCAGCGAGCCTGGTTCTGGCAGGAGCAGCAGATTTGGGGACTCATTCGCGGACGTCGATGTCGACGTTGAGGGCTTGACTTTGTTCAAGATCTGTTCCAAGTCTGGTGATTTAGCAATGGCAGATTTACGCAATTTAGGGGATGATCCTTGGGTTTATTTGAAAGATAAAAACCCAATGATGAGATTCACTGGTGGCCCAGTGGAGAGTGTGATACATTGCTATAAGAGTCAGGTTTTTGTGGGAAGAGATGGGGCTCTGGAGGTGTGGTCAAGGGTGGTTGAGAAGGAAAATGGATTAGACACAGAGAATGTGTTGGGTGAGGAGTCGTATAGAAGGAATTTTGTGGATAAATTGGAGGATTCAGAGAGAGGGACAATTAGGAAAATTGAAGGGGGTGGGAATAGGTTGTTTGTCAGCAGAGAAGATGTTGAGGGTATTGAGGTGTGGGAAAGCTCTTATTTATCTGGTGCAATTTCAGTTTCGTGACCCAGAATTTAGCTTATTTATTGTAGGAATATGGCCTTCGAGAGGCTTTTGCATTCTCAATGCATTTGTTGTAAAGTGAACAAAAGCATATGAAACCAACCTTGTTTTGACTGGCTTTCATCAGTTCAATTCCAATTAAATCTTCAGAGTTCTTGGGTCACGAAACTGAAANNNNNNNNNNNNNNNNNNNNNTGTTAAACTGTTATAAGTTTTCCCAGTGTCTTTTTTTTTTTGGTTTGAAATGCATTGAGAATGCAA

>Unigene4018_All

GTTGAGGTTAAAAATGGTAAAAAAAAAAAATTTTCCTTCTAGAGAGAAGGGGATAGTTTACAACCCAATTAATAAACTCCACGCGAATGAAAGGGCCCTTTCTACTCTTTTTCCTATTTTCTATTAAATATAAACAAAGCCTAAAATGATTAGGACTCCCTTACCTTTCACTTCTCTTCATTTCCCTCCTGGCCATATATAGTGACAACTCAAAGTACATTCAAATCCAATAGCCTATAAATGTTCCAGAACGTTAGCAAGGTTAAAAAAGGCCAGAGCATGAGTGGGAATGCAAACACAAAGAAGAATATTGAAATACAAATCTTAGTAATAACAGTGGAAGCACTTTAATACATGTCCTATCAATCTCACACTCCCGGTGAACATGGCCTACAGAAAACCTGAACCACTTCTTGAGATATTCCAGGTGTTCCTTGGCCACCATCACCATCTACCTCAGATTCCCTTGCTATCCTTAATTTTTGCCTCCATATAAGAATCTAAGAAGTTCTTTGTTCACAATCACACTTCATACCTCTGGCAAGTATTTCTTTACCTTCCTCTGTAAGACAGATTTGTTATACCAGTTCTGACTGGAGAATACACACTGACGCTATAAACTCTACCAAAGAGTAGCTCACTAACTGTCTAACTGAAGGAGTAGCTACCCAACCATCTACCCCATAAATCACTCATTGCATTCGAAATATAAGTGCAATCAATGGAAGAGAACACAAGTAGAAAACAAAAAATATTGGAGAATGGACTGTTGATGCAGATCTTGAAAACTGAACAGATCCTGAAAAGCTTCCTGAAGTAGTTGTTACAGGACTTGTACTAGGCACGGCAACATTTTTGCAAGATCCAAAAGGTGCTCCAGCCTGACAGGCAGTCACAGTGCTACAACCACACAATGTCTGGCCATTCATTCTATCCAAAACATCAAAAACACCGCCACCATCGCTTCTCATGTAAAAGTTGACTGCCAAAAAATTGGGCATCATATTCCCTGCTGCTTTATAACAGGTGCCAACCATCTGAGCAAGTGGAGTTGAATGCTCTTTGCAGGCTTCACTCTCAACTGGATATGTTGGGAAGTAATTCTCTAGGAAAAGAGATGCACTTCTGGAATTCAATGGCTTTGACTCTTTTCTNNNNNNNNNNNNNNNNNNTTACCCCAGGATCTCCAGACTCATTCTCCAACATATACTTCCATTGATAAGCAATGCCTTCCTCTGCTTCCTTTGAAGCAAGATAGAAGTGAAAACCAGAAGCCGGTGATTGTCTTGCACCATCTGAGTCACAGTGGGCCAATCTTCACCCTTTTTGGGCATATTGGAGACAGGAAACCAATACTTATCTAAACCAGCATTAGTGAACAGCTTTGTTAACCCTTTT

>Unigene4031_All

GCATTTCATTGAATAAGACTAATGTAGTATACATAAGAATTGCCACATGATTATATTCATTTGAACATCACAAGGAAAAAGAATAGAAATACCTAGAATCAAAGACTTACAGTGAAAATCATCGAGGTAATGCCTGAAGGACCAAGGTATTAAAGACGACCGCTTTCTATAATAATGCATCGACAGTGGCATGGCATGAAATGGAACTGCTCTAATATTTGCCTCATAAGACTATCAATCTGTTGTACTCTGTTGCTAATAAAGGCCGATATGAACCACAAATAGTAAGAAGCAGCAAGACAAAACTGTGAGGTGCACAATGACACGTGGAAGTAGCATCGTATTGCACTTTTAGCGTGTTGCATGAGGTAGTCCCACACTTGCAAAAGAAAGTGACCTTAGATTGCGGGCTTTGGCTATGATTCTTGAAGAACTAGATTCAATTCCAGAGCAGATTTTGCCACTAATCCGCCTTTCCTTATAACTGCTGGAAGAATTTGCTGGATTTTTTGCAGTTGGACAAAGCACTGCTTGCAGCATTCTAGCCTTTGCTCTGCATGAAGATCAACACCAACAGCTGGAGGAGCAGAAATGGTTGAGCGTACTACCGGGCCAAAGACTGCTACAAGCTTCAGTAGCATCTCCAATGAGACACTTGTGTGCCTTTCCATCTTGCTATCTAGTAAGCCCACAAGTACAGGAAGTAAGCATGAAAATAGGTCTAAGGTAAGAATCTCCATTTTCTCCATGAGGATACTGATTACATCTGCTTGCACAAAATGATCTGGAAGTTTTCTTAGAGCATTGATAGCACCCTTAATGTCATTCCGTTCCCAAAAATGCCGAACTACCTGTAATTTTGTCANNNNNNNNNNNNNNNNTAAAAATACATCATGAGTTTGCATCAAATCTTCACTTATATCCCCAAATTTAGAAGAATTCGTGTCCCTGCCAGAAATCTGAGGCTCCTCCAGCATTGGTGGTGTTCTATCCATTTCACATACAGTGCGAAGAGTCATGTCAGTTGCCATGGAAGGTGATCTATCTGTTTCAGGTATAACTCGAGGATTCACATCAGTTGTGGTGGGTGTTCTATCCATTTCACGTATAACACGAGGGGTCACAGCAGTTGCTCTAATGGGTGCCCTACCCATTTCAGCCATACTACCAGTAATCACACTAGTTGCTTTGGCGGTCATGTTTTTGGTTTCAGGTATAACACGAGGGGGCATACTAATTGCTGTGGGAGGTTTTCTATCAGTTTCAGGTATAACACGAGGGTTCATATTGGTTGCTGTGGGAGGCTTTCTATAGGTTTCAGGTATAACAAGAGGGGTCATATTAGTTGCCGTGCGAGGTTTCCAATCAGTTTCTGGTATAACACGAGGGTTCACATTAGTTGCTGGAGGAGGTTTTCTGTCAATTTCGGGTATAACAAGAGGGGTCATATCCCTTGATGTGGGAGATTTTCGATCACTTTCATGTGTAGCACCAGGGTTGATATTATTTGATATGGTGGCCATCCTATTTGTTTCATGCACAATTTGAGAGCCATATTTATTGATTGATCTTCATTGTTGAATTTCTCTCGTCTTTCAAACCTCTCAACCAGAGAGCGTGTCCTGCCTGCTACCACAGCAACTCC

>Unigene4045_All

GAACAATTGCTGCTGCATCAGTCATATTTCTGTCCCATTTTGCAGCATCTGTCATGGCTTTTTCATGTTGGTGTTGTAGGGAATCTCGAATTTTATCCTTTGCAGGAGATGATCCCAAAGTTCATCCTGATCCACATGGGGGTCCTGATGCAGACCAACTTAGGAGGTTTTCACTGGAGGAATTGCAACTGGCAACAGATTATTTCAGCAATGAAAACTTTCTGGCTAGAGGTGGATTTGGCAAGGTTTACAGGGGGCGTTTGGAAGATGGTTCACTTGTTGCAGTAAAAAGACTGGAAAATGAACCTACACCTGGTGGGGCACTTCAGTTTCAAACAACAACAGAGATCAACATGGCTGTGCATCGAAATATCCTTAGGCTACGTGGGTTTTGTATGACAGCTTCTGAAAGGCTACTTGTTTACCCCTGCATGACTAATGGGAGTGTTGCCTCACACTTAAGAGAGCGACCTCTATCACAGCCTGCACTCAATTGGCCTACAAGAAAGCGAATTGCATTAGGCTCTGCGAGGGGCCTTTCTTATTTGCATGATCACTGTAATCCAAAAATTATTCACCGCGATGTGAAAGCTGCAAATATTTTTTTGGATGAGGAGTTTGAAGCTGTTATTGGGGACTTTGGGCTGGCAATATTGATGGACTACAATGCTAGTCACATTACCACTGATGTATGTGGCACAGTAGGGCATATAGCTCCAGAATACCTCAATACTGGGATCTGTTCAGAGAAAACGGATGTTTATGGGTATGGAATTATGCTTCTGGAGCTAATTAGTGGACAAAGGGCTTTTGACCTTGCTTGGGTTGCAGCTGAAAATGATCTTTTTTTGCTTGACTGGGTAAAAGTTCTCCTGAGAGAGAACAGGTTAAAGGAGATGGCTGATCCCGATCTCCATGGCAATTACATAGAAGCTGAGATGAAGCAGCTAATCCAGATTGCATTGCTTTGCACACGGTGGTCACCATTGTACCGGCCGAAGATGTCAGAAGTGATTAGATTGCTTGGAGGTTATGGGTTGGAAGAGAGGTGGGATGAATGGCAGGGGATGGAGACACCTGACCAGGAGTTGAGACTGGCCCTTCAACCTTATTACTTTAATTTTGATTCAACAGAGCATCTGCCTCCAATTGAGTTATCTGGTCCAAGATGATTATTCCTTTTCAATACCCTTTCTACCGATCAAGAACTGACCCNNNNNNNNNNNNNNNNNNAACTAGCTACACTATGCTCTTCTTAAATAATGGCATGCATCAGGAAAGCAAAGCATATATTTAAATATACAACTTTTCAGGTCCTTTTGCCCCCTCTGTTTCTTATGAATAATATCATCAACTCGGAAAATGGCAATTTGTGGAATCAAGTAAATCGATGTTCCTTGATTTGCATTATGAAGTTGGAAGCAATTAGGGAG

>Unigene4070_All

GGTGGAGGAATGGGAATGGTTGGTTTAGGAGCTGGGGCTGTTAGAGTTCCAACCGGATCTCCTGCAAGTCAGATATCACCAGATATGATCACAAAAGGTAATGCAGATACTCCTTCAATGTCACCAGTTCCTAACATGTTTGGCCGGGGAAGAAAAGCCAGTGCTGCTTTGGAGAAGGTAATTGAGAGAAGACACCGGAGAATGATTAAAAATAGAGAGTCTGCTGCAAGGTCACGCGCTCGTAAGCAGGCTTATACATTGGAGCTAGAAGCAGAAGTTGCAAAACTTAAAGAACTGAACCAAGAGTTGCGGAGCAAACAGGAAGAAATTATGGAAATGCAGAAAAATCAGTTCCTGGAGACAATAAATCGGCAATGGGGATCTAAAAGGAAATGCTTGCGGAGGACACTAACAGGCCCTTGGTAGAGCTCATATATTTGATTGTGGATAGTGGCAGGGCANNNNNNNNNNNNNNNNNNNNNNNNNNNNNNNNNTGCAGCTTTGGCCTGCATCTTATACTTCGAAGGTTAGGTAGCTGTACGTATAGGAACAGAGGG

>Unigene4076_All

ACTCCAGTGCAGTAGTAAACTAGGCCATGATTACCGCGCAGAGAAGCTCTTCGGGGTCTTCCCATAGTCCTAGATCTCCCTCATCGCAGCCTTACTTGTCGATTTCGGTGACTGATCCTGTGAAATTGGGCAATGGCGTCCAGGCTTACATCTCTTATCGCGTCATCACCAAGACAAATTTTCCTGAATACCAAGGGCCTGAAAAGATTGTTATTCGGCGTTACAGTGATTTTGTTTGGTTACGTGACCGTCTTTTTGAAAAGTACAAAGGTGTTTTCATTCCTCCACTTCCAGAGAAGAGTGCTGTAGAAAAGTTTCGTTTCAGTGCTGAATTTATTGAGTTAAGGCGTCAAGCACTAGATATATTTGTCAATAGGATAGCCTTGCATCATGAGCTTCAGCAGAGTGAGGATTTAAGAACATTTTTGCAAGCAGATGAAGAGACAATGGAGAGATTAAGGTCTCAGGAGACTGGTATTTTTAAGAAGAAACCAGCTGATTTGATGCAAATCTTCAAGGAGGTGCAAACTAAAGTGAGTGACGTCGTTCTTGGGAAGGAAAAACCGGTTGAGGAATCAAATCCTGAATATGAAAAGTTGAAAAACTACATCTTTGAGCTTGAAAACCATTTGTCTGAAGCTCAGAAACATGCATATCGTCTTGTAAAGAGACACAGAGAATTGGGGCAATCTCTATCAGATTTTGGAAAGGCAGTGAAACTTCTTGGAGCTTGTGAAGGAGATGCCCTCNNNNNNNNNNNNNNNNNNNNNNNNNNNNNNNNAATCAGAGACGTTATCTGTTAGGCTTCAGAAGGAGGCCCATCAACTTTTAATGAATTTTGAAGAACCTTTGAAAGATTATGTTCGTGCCGTGCAATCTATTAAAGCCACCATAGCTGAAAGGGCCAATGCCTTCAGGCATCAATGTGAACTGGCTGAAACAATTAAGTTGAAGGAGATAAATCT

>Unigene4087_All

TAATAAGATAAGAGGCACACAATTATTACACTATTGATGACACAATAGGAGGCAAAAGCTGAAAAACACATGTATTGCAACAAATTCAGCTAAATTTGAATCCTCCCGTGGGAAGGCTAGGCTGGTTCGTCCCAAAGCTCGTTTCTCCATCAGCGCCATCTACAAAATTCTGGTCTTCCTCCTCTTCGATCCAATACCTTTCCAAAATCTTCACAGCCTTTTCATAAATCTCATGGTTGTCGTGGCTCTGCAGATTCTCAATTTTATCTAATCCATCACAGTCATCTATTATCTGTGCGTAAATATTGATTGTGGTGTTGCCTGATTCCTTCTCAATCTCCCCAATCTTCAGAATATTCTCTAGACCCTCAAGGCACACTGTCACAATTCTTGGATCAGGACAAACCAGAAGATCACAAAGCGGCTTAACACAACCTTGGCTCACCAGGTGTTGAATCTGTTCTCGTGATCCCCCAGAAGTGGCATTTGAAATTGCCCATGATGCCTCCTTCTTGACTTCAAATTCAGCATGTTGTAGCNNNNNNNNNNNNNNNNNNNNNNNNNNNNNNNNNNNCGCCTGTATTTGATTTCTATTGCCAGCAGTAATATTTGAAATTGTCCAGCAGGCTTCTTTCTTAATGCTCTTTTTGTAGTTTTGTGTAAGAAGTTGGTAGAGACAAGGGAGCCCTTGATTGTCAATTATACACTGAGTCTGAGCATCATCACCTGTGACAATGTTTCCTATAGCTCTTAAAGCGGGTACAAGAACTGCTGTTGATTCATGAAGTAGAAGCTCCACAAGTCGTGGGGAAACGCCTGCTTCAATCACAGCCTGAATTTTGTCATTCGTGCCATCGGAAAGATAAGAAAGCGCCCAGCAGGCATCAGTTAACACTTCATTATCAGTTGAGTAGATAAGGTGCTGAAGGGCTGGCAATGCTGGCTTAACCTGCTCAAATGGAGCTGGTGGCTTTCCACGGCAAAAGTTCGATAAAGTCCAAGTAGCATT

>Unigene4090_All

GGCTGTCCATATGCTTGTTGAGGTGGGTATCCAACGGAGGGAGGAATGGGCTGATCAATGCGTGACATTTGCTGAACAGGGGGCACTGCCATCACTGGTTGTGGTCCAAATTTGCCATCTCGTTTGTCCATTTCGATCTTGTGTTGCGTCTGCATGCATGCACAAACCGTGCAATACACCATGTCAGCCAAACATGAAAGTAACTGAGAAGCCTCTTGAATTTCCTCACTCCCAACAATCATTGCGACGATGGAGAATATGCACGCAATTTGTTGGAGACAGAACATAAAACCAATAATACAATTGTCACATTGCGTTGTCTGAATGTTGAATTCATCTTGCAACAGAAAGCGGGTAGAGGCCACTGAATTTCCNNNNNNNNNNNNNNNNNNNNNNNNNNNNNNNNNNNNNNNNNNNNNNNNNNNNNNNNNNNNNNNTATAACCAGCACAACAGACATACCTTGACATGTCATTGTAAAGAGCTCGTTTGCGGAGCAAATATGATACACATGGCCCACACCAAAACGCAAAGCAGCAATAAGGGGGGTCAGCTTGAGTGGTGTGCATGAGGTCAGTGTGCCACAGATTCCGGTAGTTCTGACGGAGCTGCATCTTGTCTAGATGCTCCTGAGAAGCCATGGTGAGCTTTACGATCGTTTGCACAAGTAGCTCACAGGCAAAAAACTTGAGTGTTGAGATTTGAGAATTGAAATCAGATAAAGTGAATATGTATAGATATAGAAGCGCAAGAAAGAGTCTTTTTGACTCGTTCGATGAAGTTTCTTTGTAATCGAGACTTCCTTAATACTTCAACATTCTTAAAAT

>Unigene4107_All

GATTTTGATTGATCAAATCATCCAACGGTGTAATTATATGTGTAGTGACACTCGAAAGGTCTGAATTTCGCGGATTTAGCATTCTAGAGAGAGATTTTCTCTTAGATCTTTCTTCCTTTTTTCGTTTCTCTGGAGTTTTCGCCGAGCTTGTCCTCCGGTGGGCTTCCTTGCTCCTCCAGGCAGGGAAGGCATTTTATCTGGCTTAATTGGTTGTAGTCCCTCTCCCTACCTCTTCCCGTTTGTTAATAGTTTTCGTGATGGGTTTTGTTGGAGGACCTTGGTTCTTCACGATGAGGGTGATTTGTAAATGGTCTGTGTTCAAAGAAGNNNNNNNNNNNNNNNNNNNNNNNNNNNNNNNNNNNNNNNNNNNGCCTAAGAGCTTAGCTTGGTTTGATGGGAAGGGATTTCATGGCTATGGCATTAGAAACTCCGGGGCTTTCATAGCTTTCTCTCTTCTACAGGAAGCCGCCTTAAAGGTTATATGAGATTGAGATGACGGCGAGATCTTTTGCGTCACCCTCCCGGAAAGGCAAGCTGTGTTGCAGCATTTGATGATTATTGGAGGTCCGGTTTGTTTATGGGGTTTGGTTCAAGGGGTCTGTTGGGCCTCCTGTGGTACTCCTTCTGAACTATAAGTGTTGGAGTTCCTTCTAAATTGTTTTTTAAGTGTTGGAGTGCTTCTCATCCCTAGTTGGAGATCAATGCAGATGGTTTGGATGGCGGTGCTGAGATGGTTGGGCAGCAGCTTCATCTCTTTGGGGCCGTATATGTACTAGGCTTGTTTCTTTAGTCCTCTTAGTTTTACTCTCATGTTTCTTGCTGTTATGCCCTTGTTCTATCTCTTGCCTCTGAGGCAATATCAATATTCCACATTTACATTTAAAAAAAAAAAAAAAAGAATTTAGGGGATCTTAATTAAATGCAGAAAAATTCGCCTATGATTTGAAT

>Unigene4123_All

CTGATAATAACCCTTTCTCTTTTGTTACCTCTCTCTCTTTCTCAGAGAGGAAAAGGAAAGATCGCGTTTTCTTGGAATGAAATCACCAAGAACGAGAAACAAGTAAGCCGTGTTGATCTTTCCCCTTGTTCTAAATCTTTGTATTTCCATTTCAATTACACATTTTCTCATTCTTTCTCCTAATCTCTTCGCTGTCCCTTTCACTTCCAATGCCAATCACTGCTGTATAACAACCATTAATTCTCCATCTATCAATTTCGCCCATCAATCCTAACACTTCCTTCTTTAATCCACATTCAGTAATCACAATGGGTCTTTTCCGGCTACTCCCTGCCGTTTTCTTCCTTTTGGTTGCTGTTCTTTCTGCTTCCTTCTGCCTCGGGATTCGTTCCATACCGGGGAGAGCAGTGAATGTCGGTGGTGGTCTTGAACTGGTTGGGCTCCGATTCGCTGAGGCGCCGGACTACCGTAATGGAATAGAATGCTCAGTGTCGGGCAAGCAATAGACAATTCGTATCTTCTTGTGACCCTTCTTTGGTTCACGTAGCGATGACTCTGGACTTGGAGTATCTACGAGGTTCAATCGCCGCTGTTCACTCCGTTCTTAAACACGCTGCCTGTCCAGAAAACGTCTTCTTCCATTTTATTGCGGCGGAGTTCGATCCGGCGAGTCCGCGAGTTCTGGCCCAACTTGTTCGATCCACTTTCCCTTCACTCAGTTTCAAGGTTTATATATTCAGAGAAGACACCGTTATCAATCTAATCTCTTCATCAATCCGTCAAGCCCTAGAAAACCCACTAAATTACGCAAGAAACTATCTCGGGGACATCCTGGATCCCTGCGTTGACCGAGTCATTTACCTGGACTCCGATGTGGTTGTTGTGGACGACATACACAAGCTCNNNNNNNNNNNNNNNNNNNNNNNNNNNNNNNNNNNNNNNNNNTTGCCACGCCAATTTCACCAAGTATTTCAGTGATGGGTTTTGGTCTGACCCTGTTCTTTCTCGGGTTTTCTCAGCTAGGAAGCCTTGTTACTTCAATACTGGGGTAATGGTGATGGATTTGGTAAGGTGGAGAGAGGGGAATTACAGGAGGAGAATCGAGAACTGGATGGAGGTACAGAGGAAGAGGAGGATTTACGAGTTGGGTTCACTGCCACCGTTTTTATTGGTGTTCGCCGGTAACGTTGAGGCAATTGATCACCGCTGGAACCAACACGGCCTCGGCGGGGATAATGTGAGAGGAAGCTGCAGGTCACTGCATCCTGGTCCAGTTAGCTTGCTGCATTGGAGTGGGAAGGGGAAGCCTTGGGTTAGGCTTGACGGCAAGAAGCCTTGTCCTCTTGATCATCTGTGGGAGCCTTATGATCTATACAAAGGTTATGAGAACTCTAGAACGAAGGGTCAGTCTCTGGGAATTTTAAGTTCTTCTCAATCTACAACAACAATGGGTTACTCGAGTTATTTGTGATTCTTTTATTTTTTTTTTTTTTTCAATTCTTTTCCAATTTTTAGTTTATGCATTTCAAGAAATTCTTGCCTAGATGTGTACAGATTATTATCCAGATGGATTTGGCACTTGGTGGGTGGGGAGGGGAAGATTTGTGGGTTTTATTTTTTTTAGCTAATATTCTTGACAAATTCATGTGCTTACAGTGCTAGATTTCAGTTCCTTATTTTAGGGTTCCATTGCTATGGAACAATTTTGGTGGGA

>Unigene4125_All

ATCCATTCAGTTTTTCACTTTTTTTTTTTTTTACGACTTTTCAGTCAAATTTTGATTGCTTTAAAGTACCCACTAGCATGGGTTTTCTAGTACTAATGGCTCCTGTTACAGCAGTTTCTCCCCTCTACTAACTAAAATTCTCAGTTTGTTCTTGCCAGTTTTAGTACTATCTGTTTTGTCTTTAACTATACACTCGCCACTTTCCAATACATCTCCCTCTGAAAATGGCTCCATCTGCTGCAACAAGAGCAAGAGCAAGACCAAAATCAACAAGAAAACCAAGAAGAACCCATTACAAGAAGCGGCTTGTCAAGTCCAAGAACATCCAAGAAGCATCAGTGGTTGAGGATTTACCTATTAATAATTCTTCCTCCTCCTCCTCCACCACCACCACCACCACAAGCTTCGATACTTGTTCCAAGGTTGATGGCTTAGATTTCGAGGGTGTTGATATCTCTACAAGTTCATGCTCTACTCCAAAAGCTCAGAGATTTCGGATACCAGAGATCGTTACATGCCCACCAGCACCAAAGAAGCAAAGAGTGATATCAAATTGCTCATTGCAAAGGAGACCAATCGCTTTCTTTGCCCCTCCAGATTTAGAGCTCTTCTTCTTCTTTGCTCTCCGCGATATCTCAGTCTAGAACTTGTTCATATATGATATGAAGTCATTTCAATAGTCTAAGTTGTGATCATTANNNNNNNNNNNNNNNNNNNNNNNNNNNNNNNNNNNTGATCACTAATATATAGTGTAATAGCTACTTTGAGATTTCAGAATTCAGAGTGAATGATGGCAGTAAGAAGTTGTTAGCTTTGCTCTTTCTGGTTTGCTATTTGATTTAGTTGGGTTTTCAATTTGTAGGGTATACTAATCACTAAATTTAGCTGGGGTTGTCCAACTAAATTTAGGCTTATGCCTATCAGCATTTTTAGATGTTTTGCTGACTATGGGGTTTGAAGAGTGAGATGTAATGGCTTGATATAAGGTTTGTGAGTGTGAATTTAGTGTGAAATTACCAGCCTTT

>Unigene4148_All

AATTATTGGCATCTTTAAACTAAGAATAATACAGAAAATGACTGCATGAATCGCTTTCATCAATGATTAGGACAATATGGTGATACAAAAATTATACTTCTCTTCAGAACTTTCAACTCAATTTTTTTGCTGTATAACACTACAGAGCACTGAACCTCAAGTGAGGAAATGACAATTTCCTTCTTTTATAAGAAAAGAAAAAAAAAATGGTCTCCAAATGCATTCACTATTCTGCCTGAATGAATTGGAGAAACGTAATGGTGAGGATCATTTCCCTGGTTTGAATATAATGGTAAAATTGTCGCAAAATTGAACTAGAATCTATTCTCAATAGCTGACTTCTTGGTTCGTTCTACCTTTGTTCGAACTGTATCCAAAATTATCTCCGTCTCAAGGTCAAACATTGTATTGACAGCATGCATAATTTCAAGCATCTCTTCTGGCTTACGGCAGTTATTTGCATCAAGTCTAAGGTGCTGCATTGGACCATGAAGGAACTTCTTCAACATACCCATGCTAAGATCATAGACAGCCTTCTTTTGTTTCTCTGAAATGTCGTCCATCTTTGACAAACACTTGTCCAGTTCAGAAGCTCTAATCCTTTCTAGATAAGCACGCAACTTCTTGATGGTAGGAACAGTCTCAAGAGAATCCTTCCAAGCTTCAAACTTGTGCAATTCCTCAGAAATAACTGCCTGTGCTTCCATAGCTTTCCTGAGACGATCTTCCTTATTAGCAGCCACAACTTCCTTGAGATCATCTACATTGTAGACTTCTGCTGTCTCCAGATCTGAGACAGATGGTTCTACATTCCTAGGAACAGAAATATCAATGAAAAACCGCCTTCCAACTTTTGGGTCCACAGGGGGAAGTCTCTGCACATGCTCTTTCAAGAACAAAGGAGCCTCTGATGCAGTGCACGTGAAAATCACATCAGCTTCACATGCACATGCCATCATTTCTGAAAGGGGTTTATATATGATTTCAGTATCCTTAAGCTCTTCACGTATGGCATTAATTCTTTCCTCAGTTCTATTCACAACCACCATTTTGGTGCACCCTTTTGCAGCTAAGTGCTTAATCACTAGTTTCCCCATCTTGCCAGCTCCAATTACCAAAACTCTAGCAGCATTATATGAAGACTCTGGAAGCTTCATTTGACTAAGTTCTACAGCAGCCGAACTGACTGAAACTGACCCTGATGCAATGTTAGTTTCAGTTCTAACCCGCTTTCCAGCAATGATTGCATGTTCAAACAGCCCACTGATTTTCCTGCCAAAGCCAGATACCCCTTGTCCAAGCTTTTTAACTTGTTTCACCTGGGCAAGGATTTGACCCTCCCCTAGCACAANNNNNNNNNNNNNNNNNNNNNNNNNNNNNNNNNNNNNNNNNNNNCAATATAAACTGGTGCTGGCAAAGCTCAGAAACAGGAATCCCACTAATCTTAGACATCCAC

>Unigene4154_All

CTCCCTCAATTGAATTTAGGCAATAATTCAATTCCACCTGCTTGTTATCACAATTCAAACTCGTATGCTGAAGACCATTCCACTAATAAAATTGTATGTCATTCCATTACGTTAGTTTTGGAAGCATACATTAATATATAAACCTTATGGCTTATCCAGTTGTCTATTTCTAATGGCGAAACGCCAAAAAAAAACATAAAACAAGTCCAAACTCTAGTTTTTCTCCCATGGATCTGCGACTCTTTGAAGCTCTCTTTCTTGAGCAAAGGCCCAACAAGCGAAAACCCATTTTGAACAAACTAACAAGTTCAAAAAAGAGCTCACANNNNNNNNNNNNNNNNNNNNNNNTTGTCACTTGGTGCTGTGCATTTTTCCATGCTATTGAACCAAATTCATAGCTAGCAGCCTGGTTTACACCTTTCTTTGCCACGAAAGTCACTGTGTATCTAACTTTGTCTCCTACATTTCTGAACACAAGTTTGGTGGGATTCACTTTCACCACCACCGCAGGTGGCGCAGTCACAGCCACTTCATATACAGACCCTGCCTCCCCCACATTGGTCAATTCTCGGGTATACCTTACAACCCTTTTGTTCGCAAACACCACCGAGAATGATGGGTAATTGAGCTCACCTGGGTCGTTGAATTTCCTCGAACAAGTAACGTTAGGACGCTTAACAATGGCTTGAACACGATCAATGGAATAAACAAGCGAGCACAAGAATGTTAAGTACTCCTCAGTTGAGATATCATAGACGAGGCCCGGAGAGAGAGCTCTGTGTGGGTCGACATGGCCAGAACCATGAGAAAAAGGGTTGGAAAGTGCACCAGTAACGCTCCCAGCATCCCGCAAGGGGGAATTGGTGTTGTCAACGACGTAGGCGGTGGTCATGAGGGCCGACTTGATTGCGCTTGGGCTCCACCTTGGGTAGGCAGCCTTGAGCAATGCAGCTACTCCACTAATATGTGGGCAAGACATGGATGTTCCTGAAATAATGTTGAATTGAGTCTTCCTGGTATCCGTCTCCAAGCCAGTGGGTCCTACAGCCTCTGACCATGCAGCAAGGATGTTGACTCCAGGGCCAATCAGATCAGGCTTCAAGATTTGCGGTGTCACTAGATTAGGACCTCTAGAGCTGAACGCCGCAACCACCGGCGACGGCCGCACGTTCAAAACGGTCCCACCAAAACTCAACAAAGCCGTTGGATTTTGATGGGTCTTTACATACTCCCTAATCATATTACCCGCCTTCCTCCCCACCGCCACTGCCGGTAATAAATGGCTGTCCGCCACCAATTCCTCACCGCTCGCCTCTGTGTTTGCCAGTATCATCCCAATCCCACCAGCGTCACGGACAACCGCACCCTTTTCTACTCGGGCGTTTATTCCTCTATCGCAAACCACCAATTTCCCACGAACGAAAGCCGGTTCGAGAGAACCGGGCAAACACAAATTGCTTGAACTGTTTCCCTTGTTGTAAACCAAGCCCAACTGTTTATTCCCCATTCCCGTTCCACTATAGAGAGAAACGCCAGTAAACCGGTTATTGTTACCCAACAGAGCGTAAGCCGGGAAATCCCTGTCCAGAGTTCCAGCTCCAACGGTCATGATCCAAGGAGCCACATTAGCCAAAGTGGCTTTATTGGGTCCGCTGTTACCGGCGGAGCAAGAGACGAAAATTCCCCTCTCCATTGCAGCAAAAGCTCCAATGGCAATGGTATCGCGGTAATACGGAACGGATCCG

>Unigene4163_All

TCTCTCTCTCTCTCTCTCTGCCATAAAGGTGGGTGTCTGTTTGTGCTTACCGTTACAGGGAGTTGACGGTGAAGATGCCGAGGCCGGGGCCGAGACCTTATGAGTGCGTGAGGAGAGCTTGGCACAGCGATAGACACCAACCCATGAGAGGTTCCATCATTCAACAAATTTTCAGGGTCGCTAAAGAGAGTCACAGTGCCGCAACTAAAAAGAACAGGGAGTGGCAAGAGAAGCTTCCTACCGTGGTCTTGAAAGCTGAGGAAATCATGTACTCCAAAGCCAACTCTGAGGCTGAGTATATGAACCTAGAAACGTTATGGGATCGTGTGAATGATGCCATTAACACAATTATTCGAAGAGATGAGAGCACTGAAACCGGAGAGCTTTTGCCTCCTTGTATTGAAGCTGCCCTCAACCTCGGCTGCATTCCAGTTAGAGCTTCGAGAAGCCAACGACACAACAATCCCAGGAGTTACCTCAGCCCAAAAGTGCAAGAACCTGTATCTGCATCGCTTACAATTTTGGAAAAAACTAATGATAAACAATGCCCTCAGTCAGCATCACTTCAGTCCAGCTGTCAGTTGAACTTTGCAAGAGCCACCAAGGCTGTAAATTCAACATTTCCAGTATCAGAATGTAACCGTGATTTGAGTAAGGCTAGTAAGCTTGTTGCACCTCAACTTTATGAGAATATTTCTGCTGGCTGTAGTCAATTGATGTCAACGGACACTGACAAGCAACTGAACTTGGGTTCAGTTTATCCTTTGTATTATGGAAATAACTATCACAACAAAGATCCCCATTTGGCTTCCCAAGTCGCAGAGAAGAATCCCAACATTATATATGTTGGTAATCCCATTAGCACATCAATGGGTGCTTTGCAGAACCTATTCTTGTGTCCTAGTGCTGAAATTGCTGCCAAGAGGATCTCACAAGCAAATTTGGAGAATACTCAAGGGAAGTCACCTGGGGTGCAATGTGATTTGTCTTTAAGGTTGGGTCTATACACTGACCCCTGTATCAGCATGGAAAGAGGTTCAGCTCAAGAAACTGAGGTTGTTGTTTCAAGCAGTTCTCAAGATAGGGGCAAGCTTAGTGATTTTTCTCTGCAAAAGAACAAGGATTTGTGCCTTTCTCGAATTACAAATACTAATGATCCTTTTATGTCCTGTCCAATTAAGTGTTTTTCAGTGGGTGAAGATCAGAGTTTGGACACAACTATGAGAAAGCGTAAGGCACTCTTCAATGACAATATGGAGGATGGACAATTTTGCTGGCAACCTGAATTTCCTTCTAACCAGTTCATTGGTCGGATAGAAAGGCCANNNNNNNNNNNNNNNCAATGTTTTCCTGTTGTAGTGTATATGCATCAAATTTTGAGATGCCTCAATTATACTGGTCAATCCATTTTGCAAGCTCGTTTTCGCATGTAGTTCTGATCACAAAGATTTACACGCTAATGGTGGGATTGCAAGTGGGGATTTAGCAAAAAGGTGAATTGTAAA

>Unigene4174_All

CAAATGGATGAAGAGCTGGATTGGACTGAGGAGATGACAAGCTATGACCAGGTACCTAAGTGGCTTCGAGCCAGTACGAGAGACGTGAGTGCTGCAGTTGCTAATTTATCAAAGAAACCATCAAAAAATATCTTGTTTGCTAGTGGTTTGGGTATGGAAACCAATGAAATGGAAACTGAAAGAAAAAGGGGGCGACCCAAGGGGAAAAAGTCTCCTAACTACAAAGAAATTGATGATAATGGGGAATACTCTGAGGCTAGTTCTGATGAGAGAAATGGATATTCTGCACATGAAGAGGAGGGAGAAATTAGAGAATTTGAAGATGATGAATCTAGTGGTGCTGTTGGGTCCCATCAGTAAGGACCAATCAGAAGATGATGTTCCAGTATGTGATGGTGGGTATGAAAGCACTAGAAATATCCAGATGGTAGAAGAAGGTGGTTCCTCTGGATCTTCCTCTGATAATCGAAGAATGACAAGAATGGTGTCTCCTGTTTCTTCACAGAAGTTTGGATCCTTGTCTGCATTAGATGCTAGACCAGGTTCTGTTACTAAAAAGCTGCCAGACGAATTGGAGGAAGGGGAAATTGCAGTGTCTGGAGATTCTCACGTGGATCACCAGCAATCTGGAAGTTGGATACATGATCGTGATGAAGGTGAAGATGAACAGGTTCTGCAACCTAAGATAAAACGAAAACGTAGTATTCGGGTTCGGCCGCGCCAGAACTTGGAAAGACCAGAGGAGAAGTCTGGCATTGAGGCACAACGTGGAGATTCATGTTTGATGCCATTCCAAGTGGACCATAAATATCTAGCACAGCTGAGGACTGATACTGAGATGAAAACATTTGGAGAGCCCGAAGCTTCCAGGCATGATCATAGTGAATCCTCAAAAAGCAGGAGAAACTTACCTTCAAGGAGAATAGCTAGCACATCAAAGTTGCATGCTTCGCCAAAGTCTAGCAGATTGAATATACAGTCTGCTCCAGCAGAAGATGCTGCTGAACTCTCCAGAGAGAATTGGGATGGCAAAGTTACAAATACAAGTGGGAACTCATTGTTGGGCTCTAAGATGTCAGATGTCATCCAGCATCCAGAGAAGGTGTAAAAATGTAATTAGCAAGTTTCAAAGGAGAATAGACAAGGAAGGCCAACAAATTGTGCCTCTTCTAACTGATTTGTGGAAGAAGATAGAGAATTCTGGTTACATGAGTGGTGCTGGAGCGGAAGATTGAATTGCGTGTTGACAGATTAGAATACAATGGAGTCATGGAGCTTGTATTCGATGTGCAATTTATGTTGAAGGGCGCAATGCAATTCTATGGGTTCTCGCATGAGGTGAGATCTGAAGCAAGGAAAGTACATGATCTCTTTTTTGACATCTTGAAGATTGCATTTCCAGACACTGACTTCCGAGAGGCCAGAAGTGCACTTTCTTTTACTAGCCCCNNNNNNNNNNNNNNNNNNNNNNNNNAGACAGGCAGCTATAGGCCAAAGCAAGAGACACAGGTTAATAAATGAGGTAGAACCGGACAATAGTGC

>Unigene4194_All

TTTGCAGCCATCCTTAGGGAAGAATCATGAAGCTCTGCAAATCTGGTGGCTATCTGAGTGTACACTGGTAAAAGTTGCTTTTCACGAGATTTTATCTGTTGTTGCACAGATTCAACCATTCCAGAATTTCTGCTATTCCTGGCTTCCTGAAGTTTTGCCTTCATAGCGATCAGCTGTTGGTCAAGCCGACCCATGCACTCAAGCAGCTCCTTTGTTCTGAACTTGATCTCAATTATTCCTTCAGGCTCAAGGACATTACCTTTTGCTGTTCGATCAGCATACATTTCAATGTGGTCTGAATTGATCTGACTGTCCACAACAACCCATGCCCCACCTCGAAGCTCACCCATCATGGGGATGTACACAAAAACAGGTTGCTTGTATGTCCTAAGGTTCTCAACTATGGTCGAACCTGCCTGAAGGATACCTTCAAAAAGATCCCTCTGTCCACCCGAAAAGCCTCTCCAGTTTGCAAGAATGAAAAGTGGAAGCTCTTCTCTGTTGAAATCCAATATTGCTTGAGCTGTTTTGGTTGCAGAATCTGGAAACCATACTTGTCCAGCCTGGGGGACAACCCTCTNNNNNNNNNNNNNNNNNNNNNNNNNNNCAGGAATCACTTGCATTACAGTTTGTGTTTCAACAGCAATTATTCCAACAGGGATTCCTCCAAGCTTTGCCCTTCCTGTCACAACTGTCCTTGCCCAACCTTCCAAAGTCTCAATAAAACTATCCCTGTCAAAAATACCCCCAAGCCACTTCCCACTGCTGTCCGAAGCACCACAAATAGCAGCACGAGGATCACATGAGTTTTCTGGGAAGTACTCCACAGGCCTTTCAGTAGGATCTGAAGGGCTTAGAATTGGAAGTGT

>Unigene4213_All

GGGGATTTTAATTAACAAAATTGCTCGAATTTACATATTTGTCACCAACACGACAGCACTTGAACTGCATAACCCTGTCCTGACTTCGAAGCTTGTGCGGCTTAATCTTGCAAATTTAGCGGATCAGAATTATTCCCTCCTCTCCAAGAATCTCCCATTTTCCTTTATTTCTCTCTCTCCCTCTCCCAAAAAGCACTTTTCCAGAAAATCAGACTTTTTTCAGGTGACGGAAATGAATTCCTATTCCTTCTCTGATTTCCCCGAGGACGTCCAGCTCTGTATCCTTTCGTTCCTAACGCCAACCGAGGTTGCCATTTTCGCTTGCACATCGAGGCGATTCTCTCCACTCTGCCAAGACGACAGCAAGCTCTGGTACGCAATTTGTGATAGGCGGTGGGGATCCAAGACCCAAATCAAGAAATGGGGTAATGGGAAAATCTCCTACAAGCTCCTCTATAGAACTCTCAACAAATGGGAGAATCTGATTGGTTTCTGGCGCCGGTGCGGCCACGGTCAACATAGGCAATCCGCCGGTGTGAAACCTCCGGCGTTAGTTTTCTTCGAGTGGGGTCCGTCGTTTCTTTCTGGTACTAGAGTCTCTCCGTCTCAAAATGGTACATATCATGTAACCAAAACGCCGTTCCTTTGGATGGGCATATTACCGGAGGGTCAAATTATAAATTACCTTGATCCCGATGGGAATAACAGTGGGCTATCTGGTGAATTGGGATTCTTGGAGATGGATTTGATTCCGGTTAACGTTAATTTTATCGGGGATATGCATCTTACTGTGGAAGAGAATGTGGGTTTTGCTTATTCAAGGAGTGCAGGTGCGACGAATTTGAAAGGGGAGTATGGTGAGGATGTGAGTAATGGGCTAGAAAGTGGGTCTCCGGGGAGCTTGTCGGAAACTTCGGAGTTGTATCAATATTATGCGAATAGAATGAGTCCTGGAGCAGATAGGTCCTGGAGGAGGCAGAGGAGGAGAGAGAAAGAGAAGCATGGGAGGAAGAAGTGGGCGACAGAACATTTCTTGAGGATTGTTGATTCCTCGCCTACGCCAGCTCGGCCATTGCAGGGATTATGGAAGGGAATTTGTGATGATATGAAGTTGGAATTCTATCTTGTAACATATGATGGGGTTGGCATCTCTTGCCGAAGGGTTGGGGACTTGTCTGAANNNNNNNNNNNNNNNNNNNNNNNNNNNNNNNCATCAAGTCCTGTATTTATTGTGTCTCCATTTTCACCTGAGGAAGAACATCTGTACAGGAGCCGCATACATCAGCATCTGTCCATGACATCAGAGGACATGCATTATGAGGTGGTCTCTCGCATTATGTACATCAACTCAAGTTATGATTTAGTCATCCCAGGTTTGGCAGGAGGTTCTGCAAATCCTTGGCATGTTGAGGGAAGGATTTGGCAATACGAGAATGGGACATTTGGTTTTGGGTTTCTT

>Unigene4247_All

GGAATGGAGAGAACTGAATTATTGCCTTAGAGTAAGAGCTTGTAAGGCAAAGTTGTTATCACATGTGGACTCCTCTGTGGAAGATGCCTTGAGCCAAATGCAGGATCTGATGAGAGAAAACTCTATAAACAAGGATTGGGTGTCTATTGGAGGCCTTACAGATATTATCATATCTATCCTTGGGAGCTCTCACAACAAAGATGTAAAGAGGAAGATTTTAGTTACTTTAAAAGATATAGCCGAAGGGCATGCAAAGAATAAGGAAAAATTGATCAACCATGAGGGGTGGGATCACATTATTCCCTGCTTAGTGCGTGACTCGAGCATCTCCAAGGCTGCAGTGGAATTGCTATTTGAGTTGCTGCAAGAGAGGTCTGGTTGGAATGTTTCTGTTTGCAGGAAACTTTCACAACAGAGCAGTGCAATTCTTTTCCTTGTCACCCTCTTGACTAGTCCTGTCAGGGAATCTTCAGTTTATGCAGAAAAAATCTTGAATAAACTTTTTGAAGTTGATGAGGAGAATATACCCCGTGCCGCAAAGTCAGGCTGGTATAAGCCACTTGTTGATCGGATTGTACAAGGGCCGGAGTCTTCAAGGATATCGATGGTCAGAGCTGTAGTTAATATGGAATTGGTTGATTCAAATTTAAAGCTACTTGGTGAGGAAGGGATAATACCTCCTTTGCTTGAAATGGTGGGATCTGGCAATATCGAATCAAAGGAGTTGTCCTTGTCTGCCTTGATTAAGCTGTCTGATTGCAATTTCAATAAAGAGCTTATTGCTGCTGCTGGTGGGCTTTCTCTTGTTTTAAACCTCATGTTCTCTTCCCATATACGAACAATTATTATTACTAAATGTGCTGAAATCCTAGAGAGAATTTCCTCTTCTGATGATGGAATTAAGTACTTTGTTGATGAAAATGGAACCCAGCTCAACTTAGAACCAATTATCAAGAATTTGTTAGGTTTGCAACAGGTCCCAAGCTTATCCCATAGCGTTCGAAGGCCTGCCTTGCGTGCACTTATCGGGATATGCAAATTTGATGCAGCATTGGTTAAGACTGCAGTTCTTACTGCCAATGGTGTATCGTTAATACTTCCTTTTCTTGATGACACAGACTCACAAATTCGTGAAACTGCAATTACTATTCTTTTCCTCTTCTCCCATCATGAATCACAGGGAGTGGTGGAACACCTGCTTAAACCAAAAAGGCTTGAGGCTTTGGTGGGGTTTCTTGAGAATGATGATAAGGGTGATGTGCAGANNNNNNNNNNNNNNNNNNNNNNNTATTAGCCAATCTTCCCAAATCTGAAGTAACACTGACTATGAGGCTAATTGAGCTGGATGGGGTCAATGCGCTCATAAATATTATCAGAACAGGGGACATGGAAGCCAAGGAAAATGCTCTAAGTGCACTGTTCAGGTTTACGGATCCCACAAATCTTGAGTCACAACATATTGTGGTTGAACAAGGAGCTTACCCTTTGCTTGTAAACTTACTGAGAACTGGTTCTGTAACTGCAAAGGCCAGAGCAGCAGCACNNNNNNNNNNNNNNNNNNNNNNNNNNNNNNAGTCCGAAGCTTGTTATTGTGTCCAATCCAACTGGCTGCTGGTGTTTTCGGCCAACACGCCCTAAGTTATGCCCAGCACATGCAGGCATCTGTAGTGTAAAAACTACATTTTGTCTCCTGGAGGCAAATGCTTTGCCTTTCTTGGTAGAACTCTTGCAGGGAGAGGTTCCTGCAACTGCTCATGAAGCAATCCAGACACTCTCCACACTGGTTCTGGAAGGGTCTCCTAACAGAGGAGCCAATGTGTTGCATGAGGCTGATGCCATAAAGCCTGTGATAGAGATTTTAGGCCGGGGAACAGATTCTCTAAAAGAAGAGGCTTTGAAACTCTTGGAGAAGGTCTTTTTGTCAAGGGATATGGTGGAACACTATAAATCAACAACTAGGTTTCATCTTGTTAGCCTGACAGGGAGGAATGTTCACGAGGATAGCCGAATTGGGAGGAAGGCTGCAAGAGTTTTATCACTCCTTGAACGCTATTCAAAATCATCATCTCTTCTGCCAGGAATATTTGGTTGAATAGATCTGTAGTCAGGCCTATCTCTGTTCTTCAGGAGTCAGGAATTCTTTGCACATGAATACTTACAATATTAATTTACTTGATGTTTTGGTCATCTTACTCGAGTCAGGATCTCATGCTGCAATAGTGTCATGGTATTTTGTGTGTGACTATTTTTTCCACCCTATTTTTCAT

>Unigene4285_All

TGCATGGAAAGACCTCGGAAAGTGGGGGCTAAGTGGACTAATAAACACATTGCTCCAGATGAAAAAGTAGAGTCCTTCGAGCTTGATTATGATGGTAAGCGTGACCGATGGAATGGATATGATACATCAAGTTATGCCCGTGTTGTTGAGAGATATGAAGCACGAGATGCAGCTCGAAGTAAGCATCTTAAGGAACAGCAGCTTAAGAAGTTGGAAGAGAAAAATAGCAACGAAAGTGATGAGGCTCTGGCTAGTGATGAGGACAACTATGAAGATGATTTGAGGGTGGATGAAGCCAAAGTTGATGAGAGCAAACAAATGGATTTTGCGAAGGTAGAGAAGCGTGTACGCACAACTGGTGGTGGGAGCACAGGGACTGTCAGGAATTTGCGTATACGGGAGGATACAGCAAAATATCTTTTAAATCTTGATGTCAACTCTGCCCATTATGATCCCAAAACCCGGTCAATGCGTGAGGATCCTCTTCCAGATGCTGATNNNNNNNNNNNNNNNNNNNNNNNNNNNNNNNNNNNNNNCGGTCAAGCTTTGGAGTTCAAGCAGCTGAATATTCATGCTTGGGACGCATTCGATAAGGGACAAGATATTCACATGCAAGCAGCTCCATCCCAAGCAGAGTTGCTATATAAGAATTATAAGGTCATAAAGGAGAAGTTGAAGACCCAAACAAAGGACACGATTATGGAGAAGTATGGTAATGCTGCCAGTGAAGAAGAACTCCCAAGAGAGCTTTTACTAGGACAAAGTGAAAGACAAGTTGAATATGACCGTGCTGGACGGATCATAAAGGGGCAGGAGACTGTGCTTCCCAAAAGCAAGTATGAAGAAGATGTTTACATTAACAACCACACAAGTGTGTGGGGTTCATGGTGGAA

>Unigene4292_All

TTTTTTTTTTTTTTTTGGGGGAAAACGAGGGCCGTCAGTAAAATTTGTCCAAGACTCGTGCAAGTATAACCTCTAAGAAATTCAAGGAAAAACTGGAGAAAATACAAGGTCACCAAACAGACGACATACTAACATAAAAAAAACCAAATAAAACATAACCAGACCACCAGATATAGTGCATCTCTTCCTAATTTTTGAATGTCCAGTTATGGGCACTCCGTGGCCCACTTGAAATGCCTTCATAGTATCGAGAGTCAAGCTTTTGATTGTTCCAGTCCTTGACAAAGTCTGTAAACAAATCGCGTGCAGTCTCTGATGAAAGTTCAGAGAAAAACATCTTCTCCTCTTCCCTCAGCCAGGTTGCAAATTCGTTGTTCTTAGAGAAGTAATCATCATTGGAAAGCTCTTGGAATTTCTTGCCATGATCACCTTTGCGATGTTTGCCACTATGCTTATCCTTTGATTTCTTATCTTTATCAGAGCGGTGCTTGTGAGACTTCTTGTACCTTCGCTTCTCTTTCTTATCACTCTTGCTGCTTCTCCTTNNNNNNNNNNNNNNNNNNNNNNNNNNNNNNNNNNNNTTTCTGTGTTTTCCTTCATCTTCGGAGGCAGAAGAAGAGGGGCTTCTCTTCCTGGTTTTCTTCTTCTCCTGGTCCTTGGATTTGCGGTCGCTTCCCATTCTCCGATTGATATTTTTTAATGACAATTGAATTGTG

>Unigene4299_All

AAAAGGAGCATGGTCATTCTTTAGATAAAATTCATACAATTATAAATTCAAAATTTCTAACTTATATTTCTGCCAAATATAGAACCTAAGCAGAAGCATTTACTCTATACAATGTGTTGGAATAAAATCTCAATTCTCTTGCAAAATTTGAAGCAATACCATTCGGGCGAATCAAGAGATTAGGGAAAAGAATTCCTCTAGCCGACTTTCACTTATCACCACACCATGTTTTTGGTTGAAAACTAAATTGATAGATTCAGCCCCCACATTTGTCAACTCATTAGATGCATGGATCTCCATGTATTCATTCAAGTTGTTCACAGTCCCATCTTCAGCTACTATTGGTTCATAAGCAGGAGGCTGAGCAATCCAGTTCCCAGCAGATTCTTTCATGTGTCTTCTATCAGATGCAAAGTTGCGGAGGAATAATGGAGCATGCACAATCCTGAAGAACCTCTGGTAGTCAGAAAAGAGCTGAAATGATGGCCGGATAGTCTCAGAAATGTACACAGCCAATGGAGAGGGAAAGGGAAGACTCGAGTCTAAATCCCACACTACATAAGGAGAGTCACCATCCCCTTTTCTCTGAATGCAAATGACATGATAATCCCAAAGGACAATCCCATCTGCTCTCTTACTGGCCTTTTGATGCCACAGAGGAACCTGTTTATTTTCATTGGAAATGAAAGCAACAAAGAGATCAGATCCCTCAGCATCTGCTATTCCTTTCTGGCACAGTTTCTTGCAAAGGAAGTATACATTCTCCTCACAGTAAAATGGAGTATGATCGAATTGAGAAACGTTCACTTCGCTTACTCTTACTTCCAAGTTTGCACTCGTCATCTTCCTTCTCTTTCCCGTCAAGCTTTTTTCTCCTACCAACTTACCATCATTTCCTTATATACCTCCAAAACTNNNNNNNNNNNNNNNNNNNNNNNNNNNNNNNNNNNNATGTAAACGGTAAACCATAAGCCATTTATTTGCGAAAAGGTCGCATGAACGACGCCCCGTTTAATGTTTCTCTAACCATTTTTACAATTAATACAAGCAAATATCACCGCCACAGCCGCCAAAAATGGATTTGGAACCACAACCAGAGACGTCTGCGCCGTCGCATCGCCGTCGTTCAAGCTGCAGTAACTGCAATCGGCCACTCCCAGTCTGCCTCTGCCACGTAATCCCCACCTCACCAATTCCAACTGCGTCACAAATCATAATAATTCAACACCCACACGAATCCCGCCACAAGCTCAATACTACACCTGCACTCACCAAATGCCTACTCAACGCCACCTCTCTCATATCCCGCCGCCTAACCCCTAACCTCCACCCTCTCCTCACCAACAACCACCCCTCTCCGGCTACATTTTACCTCTTCCCTCCTACCCATTCTTCGCCGG

>Unigene4310_All

TACCAAATATTGAAATTTATCCTTAATTTCAAACAACAATCACAAAACAGTAATAACAATTACATAAACATACAAATTCAAATCTAAATTCAATCACATAGATCTTAAAATTCCTCTCTACCTAAGCAATGGTTGAATTATCGGTGGGAGAAGTCCACTCTATGTCTTTGAGAGCAGGTCTCAGCTCTTTGCTGCAGAATTGGTTATACTCTAGAGTGTCACCGTTATCTTTTTTCACCTCCACCACCAAAAACGACGGCGTAACTGCGAATATTTCTGCTGCGACAGCTAATTTCCCTTTCCTTCCACTCTCTTGACCCTGCATCCTCACTTTGGTTTCGCTCTTCTTCACGCTGAATCTCCCAGCCTTGGCCACCTCTTCTAGTCTTGAAATCACGCTACTTGCTGGTCTCGTTGTCACAAATCTCAACTCCTCCTTCTCCTCCCTCTTCTTCTCTTCGAATAGAGGTGATAAATCAAATCCTTCTGATAATGATATTATGTGAAAAGCATTTAGTGTTTCAGGTTGCTTTGATTTGCCTCCGTTGTTTTGATCTTCATCACCATTGAAGGCATCAAATTCCATTTCCTCCTTCGTTCTTATAGTTTTAGGCACTGATTTCTTGAACCATGAGGAATCCATAATCTTGGCTATGGTGATTCGAGTGCTAGGATTTGGATCGAGAAGTTTTGTGATTAATCTACGAGCTTCAGGGGAGAACCATGGTGGGCATTTGAAGTCACCTCTGTAGATCTTTCTATACATGGCCACTATATTATCATCTTGAAATGGTAAGAAACCAGCGAGAAGAACATAGAGAATAACACCACAAGACCAGAGATCTGCTTTAGCACCATCATACCCTTTCTTCCCAATAACCTCCGGTGCAACATAGGCTGGTGTGCCACACGTAGTATGTAACAAACCATCTTGCTTCAAGTGCTCAGAGAAGGCNNNNNNNNNNNNNNNNNNNAAGCCCAAAATCTGTAACTTTTAAGTTGCCATCTTCATCGAGAAGAAGATTTTCTGGCTTCAAATCACGATGATAAACTCCGCGACTGTGACAAAAATCGATGGCAGATATCAACTGCTGAAAATAAACTCTAGCAACGTCCTCCCTCAAGCGACCCTTAGCAATCTTAGAAAACAACTCACCTCCTCGAACAAGTTCCATAACAAAATATATCGTAGATTTACTCGCCATGACCTCGTACAGCTTGACGATATTGGGGTGCTTCACCATCTTCATAACAGCAATTTCCCTCTTGATTTGCTCCATCATGCCGACCTTGATTACTTTCTCCTTGCCGACCACCTTCATCGCCACGCTCTTTCCCGTCTGCAAATTACGAGCATGGTAGACCTTAGCGAAGGTGCCATGTCCTAGCATTCGGCCGAGCTCGTATCTCCCATGAAGCAAGGTTGAGTGAGAATCTCTGCGCTTTTCTGCCATGAATGTACGCAAAGAGAAATCCGCTACAGAGACAATATTTTATTCAAAGAGTATATACAATACAAAGAGAATATGAGATGAAAATTCTGAGAAAAAGAATTAGAAAAGAGGAGCTTGTGCAGTAGTTTGGATTGGGAAAACGATCACCCGTCTTCAACGTGTTTCCGGTGGCAATTTCTGCCGCCGATACACCGCTTAGCACGGCAACAAAGTTCACTCTTGGTCAGCAAAGCAGCTCCCATCTCTCCGCATTCCCCACGGGAGATTCGTAATTCCTACCCAGAACAGAGGACGCAGAGAAGAGTTATTAAT

>Unigene4317_All

TTTTTTTTTTTTTTTTTTTTTTTGCAGGGCAGAATTGCACACCTAAATCTCTAGATAAATATTATAGTTGCAGGCATAAGTGTCATGTATCCCACTCATGTATTTGATGCATGGACAAGCAAAGAGAGTTTGCCTCCTTTCAACTCGAAAAATACATGATCAGAAGATTCATGAGACTGCAAAAATAAAACCAAATCTGCTAGCTTTGATGGTAGAATAGAATGGAAAAATCTCATTACTGCTCTTTCCAAATCCGCCCAGTCACCCTAAGCTTCAATTCCTTTCGGTCTCCACCAGAGAAGAAAAGAGCCATGTTGCGCTGAATTATGAGACCATCAAAACTGTCACGGACCTTCCGATGGCTATCCCTTATGCTCCTAGGCACTCCTTGCCATATCATCTTCCTTCCATTCCCACCAACCTCGAGGCTGTAGCTATAGCTCTTTGCCTCATTGTCATCGCCCATAAACCGCAAGAATGCTATGTAGACAGGGGCCATCCCAAGCTGGAAAGCTTCGAAATGTAGACAAAAGTACTGACCAAAGCAGCTAAANNNNNNNNNNNNNNNNNNNNNNNNNNNNNNNNNNNNNNGTGGATTTGATTTGACATATCTATGGTTGAAAGTACTGCCATTATGCATGTCAACTTTGTGATCATCCTTCAAATGGGCCACTAGATAAGGAATATCACCAATGACTGTGCATTCTGATCCAGCATAGGGACAGTTATATGGTCTATAAACACACTGTGATTCATGTTTTAGCTTGCTGTAGTACGGGTATATGCCAATACACCCAAAACTCTGATATTTACAAGGAAGTTCAAGAGATGCAGCAACTTTCTCTAATGCAAGACATCTGATGTTACCAAGCTCATGCCTGCAGGTGGGGCACCTATTATGAACTCTGGGTTTGCAGCCAGAACATAATGTATGACCATTTGAGCACTGATGAATAGGAGGATACATAGCATTTAAGCACACAGGACACTCTACTAGCTCTCGAACACTGCTAGTCACAGTAACATTAGGTTTCAAAGCTGTCTGAGCAGGATCATTCACACTTTCACCAACATCCATCATGTCTTCATTTTTAGGAGGATCAATAACCTCTGGCTTACTTCTCATGTCATCAAAAAAAGGATTTCCAGATGCCATGCTAGTATGACCACTCACCGAATTTTG

>Unigene4337_All

GTGTAAAATAAAGAATTAAAGTGTTGCCCAATGTTACATAGGGTGAAAGATCACAATAATACATGCTAATCCAGTTATATTGAACCCAACGCCCCCTAATATACAGAATTCACTACCATCAATGAGTTTTAATTTTAATGATACTACATTCATCTTTAATACTGAGGTTTTGAGTTCTGAGTCCTTGTCAAAGCTAAATGAGCCCCAAAAAAATAGCTATATTACATATAAAGCCAATATCTACCTCCTAGACCGCTTTGTTGGCCGCTTCGTTGCCTCCTTTCCACTGCCTTCCCTCCCCCGCTTTCCTGAAACCTTGCTTGTCTCCGCTCCCTTCTTTGATGGCCTCCCAACGTTTCTTTTGGATGGACTACTCTCCTCTTGTTTCTCATCGTTGTTTCTCCTCAAGACCCTTTCTTTCCCTTTTTCTACCTTCCCAGTACCATCAGTTTTTTTATCTCCACTACCATTGACACCTCCCCTAGTATTCTTTGATGTTTCTACTGGAGAATTTTTCTTTATTCTCTTCAAGAAATCCACCGCACTTCGTTTCTTATCCAATGCCAATGCTTCAGTTTTCTTCTTATCAGTTTTCGGAGTTTCAGGCTTGTCAGCATCAGCTTTTGTTGTAGGACTTGCATTTTGCTTCTTGCTTGGAGCACTAGTAACTTTGGTGAGATTTTTGTTGGTTCTTCTCGAGCTTCTTGTGCCAGTTGCAGGCTTCTCCTTCGACTCTATCTTCATAGTACTCTGTTCAACTATTGTTGGCTTTGGATCACTAGGCTGTTCATTGTCATTGCTTTGCTGCGGCTGTTGTTGCTGCTCAATCTTTTGTCCATAGCCAGAAGAGGAAGGCTTCGCAGACAATGAACTCCGTTTGCGACACACTACAATGGGAGCTGAACCCTTGAGCAATGAGTTTGATATTTCAAGCTCACTTTTGGGCTGAGATGCAGTATTTTGAGATGCTACCGTAAACTCAGATTTCTGGGTCTCTTTTTTCATTTCATTTGAGAGAAGCAATCGGAGTTCATAAGCTGCAAGTGATTCGGTTGAGGATTTGGGGAAGAAAACAATAGCATTGTTGACGAGAAGCAAGAGATCTCGATAGAATGAGAGATTTGAAGAAGAATAGGATCCNNNNNNNNNNNNNNNNNNNNNNNNNNNNNNNNNNNNNNNNNNTGACCATGTTTTTATACGCATCCGTTTCCTGAGTTTCGAGCCGGCCCTCAAACAACGAACCGTGTCTATGGGCCCGAATCATTTCGATCAACCCAATCAACGGCTCAGATTTCACGTTGATCCCATCGCCTCCAATTTCTTCCCCCCTCTTCCTCTCTTTCCTCTTCCTTTTTCTCCCTAAACTCGCTGAACTCTGCACCTCACTACTCAACTGAGTCACTGAGTCACCCAACTCGTGCGATTCCTCTCCTCCTCCACCCACCGGTTTCGAATTAGAAACGCTCAAAACCGGCGCTGCCTCACAAGGACCTTCCTGAACCGGCTCAGCTTCCATCTTAACCCCATCCTTCTCTGCCGTCTTCTGTTCCGATCCGGTGGAGTTCGACTCGTTCACCGACCGGTTTTCCCGGTCAGATTCTTCGCCGCCGGAAACGGAATCCTTCTTCTCTGGTTCGTCTTTTTTGTC

>Unigene4394_All

ACACTCACACTCTTCACCCCAATCCCCTTGTATTTATTCACCAGTTGCTCCACTGTAACCTTTAACTCTCCCAAATTATAGAACACTTGCAATCCTGTACCAACTTCAGCCTGATTCAATCCCCCCATTCCCCTCTCCAGAACTTTCATTGCCTCGTCTCGCAGTCTTTCGCCAATTTCTTTAATCCAATTGAGCTCTTCATCAATGCAATCGATTCCCATCAAGTCATATTCATTGCACATATTCAAGATCTCACAATGGAGCTGGGCTGCCTTGGCCAGATCCAGCTTCTCAGGCTCTGTCTCCGATGCCGAGATCAGATCGCGCAGCTTCTTGGACAATCGGAGAGCACGGATCGTGTGTTGCAAAAGCTCTGTAGAGCAATGGAGATTGGAGAGCTGTAGTGTTTTGGACTGAATGGATTTGTGGGGTTCGGAGAGCTCCGATCGTACGCGGCGTACAGAGGACTGGAGAGAGGAGACGGCGGATCGGACGGTGGAGAGAGCATGTTCAGCATGCTTGAGAGATGATAACTGGTTGAGGAGTTCGGTGTGGCGAGATAAGACTTCAGTGCGGAGTTGGGATTCGAGGAGGCGGATGGCATGGTGGAGGTGTTCGGCAGTGGAGGCGGGGGAGCCGGAAGAGANNNNNNNNNNNNNNNNNNNNNNNNNNNNNNNNNATGAGGAGAGAAAAGGGGAGAGGATAGGGTCTTTGGCAAAGGAATCTAAAGGGGAAGCAGCTGCGGAGGATGGGGCGGTGGTGGAAGGAAGAGAAGAAGAGGAGGAGGAATTTTTTAGGGTAGAGAGGCGTTGCAGAGGGGATGCAGAAGGCGAGAGTGCAGATCTCTGCAGTGCAGGTGAAGCCATCTTCTNNNNNNNGGTTCTTGTTATTTTTGGCCAATCAACTTGAACCACAATTTTTAGTTTTGATGCATAGTTG

>Unigene4397_All

TTGGAAATCAGAGATGTAGAAGTCAACTGAGCGCCTTTCTAATGACCTGAGTCCCCAAGTGAAACGGCGATGCGTAGGGTTGGCTGCTTTTGAATCCCATCCTCGATACTCATACAAACTTGTGGACGTATATACACATCTAGGAACTTTTTGGAAGGATGACTCAAGAGGCACATTACCACAGGTGACCACCCCAGATATCTTCACAAATTGTCCATTCTTGGCATTTCGCAGCTCAGCATCTGGGTAACAAGCAATAAAACCCATGATAGCCCTCCTCCCAAAGAAGGTATTCCATATGAATAATCCAGCAACCGCACCAAAAAGAACCACCACAACAATTAGGAGAATGGCATTATGCACTGCTCCAAGAATAAAACCACCAGCAATGAAACCCATGACAAAAAGTAGAATCAGTGACCACAATATTGGCTTTGGGAAGTTCTTTCTGAAAGAAAAGTCATCTTCTTGGCTAAGTACAGTAACCGCTTGATTGTGAACAATGGCAGATCCTGGAATTTTCATTGAGCCCATAGATTCTAATGGANNNNNNNNNNNNNNNNNNNNNNNNNNAATTCTGGACCCTTAAATAGGCATGGAGACCAGTTGCAGGAAGAACAGGAGGAAGGGGACCAGAGTTTTGGCGCCCAGAAGGTGTTACTCCACCAGACTGAGGGCCAGATGACTTTTTTACAGGATCTCCATGTCTATTTAAGGGTCCAGAATTAGATTTCTTTAATGAAGCTGAACCAGACATGCCTCCAGAAGATGCAGCACCTGATGTGGTGTAGGCAGCCCGGGCTGCTGCATTGGGCATGATTGGTCCTGAATGAGAA

>Unigene4410_All

AATGGTACAATCAAGAGGAGAAGTCATTCAAAGCATCACAAGTGTCACAAGCGTCACCGCATGGCAGAGTCAAGTGATTCAGATTTCTCAATTGATGAGGACAGAAGCCATGCAAAGCATCATAAACGTCATAGGCGATCCCATAGCATGGACTCAGGGTCAATGGATTCTGATGATTGCAGGGATGACCACAGTTGTGACCGAAGAAGCCAATCTTTAGGAAGGTCATCAGATGACAATAATGAAGAAAGTAGAAAATCAAGCCATAGGAGGCCTGATCATCATTGTCAGAAGCACCGGTGCCATCATTCCAATGAGGGGAAAAATTATTCGCCCCCAGGTAAGGAGGGAAATCATCCACGTCATGGTTTACATGAAGGCAATGGAAATCATGCTGACAAAAAGGTGAATGAGAAAACTAACACCGAAATGGATGGACAGAATGAATAGGGTTTCGTCGATTTATCACTTTGGGGGTAGCTCAGAACTCTTAAGATGGATGGAAATTGCTTAAAATTTATGGGAGGCTTGTACTTGTCTTTTTATGTTGTTAAGACTGATGTGGGNNNNNNNNNNNNNNNNNNGCAACCTAAATTGCCTTGAATTATATTTTTGTTAAAATATAAATTTGCCTTTTAACTGGTGG

>Unigene4420_All

TTTCTCCATTGATATCCATCTTTTACAATTAGGCTCTTGTCTTTGGAGTCGGTTCTGACAAAAATTTGTGAAGGCTTGACCATGGGAATCTCAATCCTTGGTTTCTTGATTGAGCCATAGACCTCATAGTATGAGACGCTACTACTCAGAGGTGAGTTGCTACCAATTTCTTGAACACTTGCTTGAAGGGCACTGACCTTACAGCCCATAACTTCAAGCATAACCTTAAGAGTTTCATTCTCTTTTCGGGTACGTTTCAGTTCATCTTGGAGAGCTTCCGCCTGAATAAATAAGCATATGAACAAAATCATATTAGAAAAGAACGAAATGGACATGCCATTAATGGTAAAATCTAGCCCAGTTAGCCAGTTTGGCTATGCAGGTTGCCTTCACCCTCCAGTGCACTCGAGAAAGCTGATTAGAAACAAAGCTTTCTTGAGAAGTGCCTTGTGCAAATCATTCAACCTCGACTNNNNNNNNNNNNNNNNNNNNNNCATCCATGGTAACGGGTTTAAGAAGGTTTATGAATGTCGGTAGGAGATGGGGTAATATTGATGACCAGAG

>Unigene4431_All

CCCCTTTTTCTGTTCATGAAAAGAGAATGGAGAGATGGAAATAATAATAATATACTCGCGATATTGGTAATGAGAAATCTTGGAAACTCCAGACATTTGACTGTCCAATTTGCCTAACATGCAACTCTCTCATCTATGAACTGCGCCCTAAAATTTTTCCCGAGTGTTATGCGTCTGGAAACAAGGGAATGGAGAAACAAAAATACGCTTGTTGTTGCCATCTTCACTTGCGACTACCGACTTCAAGTGGCTCAACCACCTCAGCTTCTAATGTTTCTGGGATATTCACTTGTGACTACCAGCTTCAACTGGCTCAACCACCTCAGCTTCTAATGTTTCTGGGATGTGAGGTTGAGGAGATGCATGAGTAGACCATTTCCCTGATATGGCAATGCCCATCATCTCATATATCTTCCCACCAAGCTGTGCTGGATTCTCAGGAGTAAACCCACTGGAGACTAAAGCTGCATCATAAAGAAGATCAATAGCTTTCAGGGCATCTTCATCATCTGGGTTAATCCTGCATGCCTCATTTAAGTTCTTAATTATTGCATGTTCAGGATTGATCTCAAACACTCTTCTGCCTCTCATGAATTCCAGGCTAGACATGTCACCAACTGTTTGTGACTTCATTAGCCTTTCCATGTTGGCAGACCAACCAAATTTCCCAGATACAAGAACGCAGGGTGAGGAGCTCAGGCGGTTTGAGATTTGTACACTGGCAACTTTGTCACCCAAACGCTTCTTAATCCAATCACAAGTTTGGCCAAACTCCTGCTTCATCTCCTTCTCTTTTTCCTCATTCTTATCACCTAAATCCAAGTCTTCCTTGCTGATATCAACAAAATTCTTCTCCTTGTATGATTTCAGGTTCTGGACAGCAACCTCATCAATGGGATCAACTAAGAACAGCACTTCAAGATCCTTCTCAATAAGTTTCTCTAGGAAAGGTGTGTTCTTGGCACTGGTCACACTATCAGAAGCAATATAATAGATATCCTTCTGATCAGGTTTCATGTTCTCTACATATTCATCCAAGCTTATCATCTCCTCCTCACTCTGGGAAGAGAAGAATCTAAGCAGTGGCGCAATACGCTTGTGGTTTTCACGATCCTCAATGCAACCCAATTTCAAATATTTGCCAAAGTTCTCCCAGAACTTCTCATAGTCTTCTCTATTCTCACTCATAGATATTCCCAAAATCATGTCAAAGGCCTTTCGAACCAATCGTTTCCGCATGATCCGTACAATACGACTTTCTTGAAGAATTTCACGTGAAACATTGAGAGGAAGATCATTCGAGTCCACGACACCTTTCACAAAGCTTAGATACCGAGGGAACAGCTCTCCATCAAAATCATCTGAAATGAATACTCTTTTCACATAGAGCCTGATATTTTTTGTCTTGGGATTAACTATGTCATCCTTTCCTGTAGGAGAAACAGCTGGCACATATAGTACAGATCTGAACTCCACCTCACCCTCTGTTGTGAAATGTGAGGATGCTAATGGTTCCAAGTATTCATTAAATGTTTTCTTGTAAAAACTCATTATATTCCTCTGTAGACACTTCCTTTGGGTTGCGAAGCCATAGTGGTTGGGTCTCATTAGTCAGTTCCCAGTCCCAGTACCGCTCAACAACGGTTTTTGTTTTCTTCTTTTTATTAGTTTTATCATCCTGTTCATCTTTTTGGGCTTCAGTTGGCTCCTCATCAACCTCAACCTCTTTAGTGTATCCTTTTTCCTGCCAGGTGTATATTGGAAATGAAACAAACTGTGAGTAGTTTTTCACAAGCTTCTGAATCCGCTCTGGATTTGCAAAGCCCTTGTCATCATGCTTAAGATACAATGTAAGACGGGTTCCTCTTGGAATGAGCTTCTCAGGGTCTGTCTCCTCTCGAATGGTATAGGAGCTAGCATTGGCCTCACCTTCCCATACATATTGTGTATCAGATTTTGGGCTTTTTGTCGAGACAACCACCCTATCTGAAACCAGGAAAGATGAATAAAAGCCAACACCAAATTGACCAATTAAATTGTTATCAGCACCAGCATCTTTGCTATCCTTCAATGCCTTCAAAAATTTTGCAGTTCCACTTTGTGCAATAGTTCCTAGACAGTCAATTAATTNNNNNNNNNNNNNNNNNNNNNNNNNNNNNNNNNNNNCACAATTCCATTGTCCTTATCAGTTTGAATACGGATATCAAGATCAACAGCATCCTTCAAAAGCTCAGGCTCTGTGACACCAAGAAATCGCAGCTTATCCAAG

>Unigene4434_All

AAGAAGAAATCCATTAGAACATGTCTTAAAATAATCACACAATAGTAAGCTCTCGTGATATAGTTCATGCGAAAAATCAACCATGAAGAATCTGAGTGCCAAGGCTAAAATTAGCATCCCCAAGAAAATGTTACAATGAGCCTATCATCTAATAACTACAGAAATAAGCATCTCTTTGTTTGTTCACTTAACTAATCTCGCATTGTATTGCAGCACCTTATCTCTTCAGTACTTTGTGGATCTAAAGTGGTAATAATTAGTGGACACAGCTTTGAGCAATCTAAATTGCAAGAGGTGGATAAATATATATTGTCAAACAATTGAAGATTTTCAAAGGCAGGATAGATGAGTACCAGGAAACTGGTATGATAGGTGTCCTTCACAAAAGTTTGTTCATGGTTTACCTCTTTCCACGACTTCCATGGCCAGATCTTGGACCAGGGTACCGAGAAAACTGCAGCCCTAAGTAGTTAGATTCATGACCATGTTCATCCATCTTATAACCTTGCAAGGCGCTCATTGCAGTTGCTGCACAGGCTGGATTTTCAAAATCAACAAAACAAAGGATAATAGGATCTCCACCACGATGCTTGGATTCTTTACTCACAAGTCTTACTTCCTTATATCNNNNNNNNNNNNNNNNNNNNNNNNNNNNNTGCTGTCAGAAGGGAGTCCCTCAACATATAGAGTGCTGGAAGCATCTGGAGGTAGAGGTATAGTTTCACGAACAGGCCTAGCTTAGCTGTTGTATCCACTGAAGGCTGACTACGGAAAACAATGTCCTGACCATTTGGTGCCAGATCTGGACCTCTAGCACGAGGATGGCCTGTTATACCAGAATCAAGTATTGGAAGACCAGTCATCGCACTACTGGCAGCCCTTCCCAACCCAGCACTTAATCCACTAGCTTCTCCGGAAGAAAAAGGAGTAATTTGCGCATTTTGCAGATATCGGTCGTACGCTGATCCAATTGTTTTTGTGTCCTTCACAGCCTGATACCTATTTTGATCCCCATCTTTTGGATAATAGTTATGA

>Unigene4445_All

CCCTCCCTTTTTCATACGTACACAACCAAGAGACACTAGAACCTGAGCTCAATCTATTTAAAACATAGATCCACCCAACGCCAGCCCAGGCTAACCGTAGCTAGCAAGGACTACTGTACAGAAGAACCTTCCAATAAGTAATGGAATATCAGGTCAGACATAATATATAAAAGAAATACTGAGCCTCCTATTGCTATCTGAAGCACTCCCTCCCCCACCACCCCTTCCACTATCGGCTAAAACTTCGCCTTTACATTTCAAAATTTATGTTTGCAAATTGGAATCCTTGAAACTAAACAATGGATTTAGCTTCTATTCTCAAGTCCCCTACACATGTGCTCCATAAGAACAAAAAGTTCCCATATACATAATTTCAGAAGAAGATAGCTCTCCCCAACACTCTAGCTCCAACCAAAAACCACAAGATGGCACATAATGCTCCAAGAATAGATAAGAAAAGAATACCCCTACTAGTTCCAACTTTAGTTGAATTTGCAGTTTGATTACTACCAACAAGATTATCAGTATTGATATNNNNNNNNNNNNNNNNNNNNNNNNNNNNNNNNNNNNNNNNNNNNNNNNNNNNNNNNNNNNNTTATTCACCAAAGGAGTCTCGGCAGCTGATGCAGGAGTAGTAGGTATTGACTCCAAAAATTCCCACAAAGCAGAGGAGAACCAACCAGTAGCACCACCATCCTCTCCACCACTTTGATTCTCATTAGCT

>Unigene4478_All

CAAGAAAGAAGATGTCCACACAGAGACACGGTAATTCTCCTTCCACATCTCCTGAAGAAAACGCTATGTTCCTTGATATACTTCATGAGGCTCCTTTATGTGGTCACCGGAAACGCAGGAGTATTTTTGGAGCTGTTGTCTACTGTGTTCTACTGGCAGGTTATGCTGTTTTGGGTGCGATAGCTCCCTTTGTATTTCATCACGGATTGGTGCCATCGCTGCTTTGTAGTTGCGATGTTGCTCTTCTAATAGTCACAGGCATCTTCCAACAATATTTTGTTTCTCAAGTCCAAAAAATTCGTTTACAGGGCTATTATAGTTTCNNNNNNNNNNNNNNNNCTTACCATTTGCCATTACTGCCTATGGAACTGCTGCAATGTTGCTTGTCATGGTATGGAAACCACAAATCAGCTTTCTTTCAATCCCAGCATTACTAAGGATTATTGTGTTGACTGAAGCAATATGTGCTGTGTCCTTTTTGAGCATCTATGTTGGTTATCTACAGAAGTACAATTCATTAGATTCTCAGCCTGATATTTTGAAGTCATTATATTCTCCACTCCAGCAATCAAGTTCTTTAGAAGGCTTGAGGTATCATGATGGTGGTCGACTTTCTGATCAGCAAATGGCTTTACTTCAATATCAGCGTGAGAACCTTCATTTTTTGAGTGAGGAGATTCTTCATTTGCAAGAGTGCCTAAGCAAATATGAGCGGTCTAATGATGGGAACACACCTCCGGTTGATCTTCCTCACATGTTAGCAGCTCGTGAACAGGAATTACGGATGCTTTCTGCTGAGATGAATCAACTGCAAACTGAGCTAAGGCTTGCTCGGTCTTTGATAGCTGAGAGGGACTCTGAGATACAGCAAGTCCGCACTACAAACAATCAGTATGTGGAAGAGAATGAAAGACTAAGAGCTATTTTAGGGGAATGGAGTGCACGAGCAGCAAAGCTTGAGCGAGCATTGGAGGTAGAGCGGTTGTCAAATCTTGACCTTCAAAGGAGGCTTCCAACAAGAAATCAATCAAATGCATCACCTGAACAAAGTGGGCAGCGTACAGCTTAGTTCCCTTTTGGAACTTTTTTGCCATTTCTCCGGAAATTTTGGTTACATTGGGGGAGTTGATGGTAATGTATATTACAGTACCTCGAGGAAACCAGTTGAGGAAGCATTTGATTTAACGATTGCATATCTTGTTTGATGCTTTCTCTTATTTTTCTCTTTAAAATTATTAGAAAAAAAAATAGGGGTG

>Unigene4483_All

ATTATTTGCCAACCTTCTTCCATTTGGTTGATCCTGGTGGCATTGCAAATTGGTCAGTAACACATGTAGATTGGTCTGAGAGAAAGTGGCATCCAAAATCTTATAGGGCAAAAGATATTACTTATGAACTTCTGAAGAATATTACGTCGATTGACCTGAGTATACATGTGACAAGTGATGAAAAGAGGGAAGTGCAGGTACAACCATGCTTATGGAATGGTATCCAACGGCCATGCTATTTATTTGCTAGGAAATTCTACCCAGAAGCTATTGATAACCTCTTGCAGCTTTTCTCCAACTACAAAACAATGTGAGCGAGATAATTTTGACTCTAAATTCTTTGGTCGGATCAACAGTTCCTTTCGACACAGTTCCTTTTGACCCCCCCCCTACTGCATCTTGGTTTCTTGGTATATCTTAAAAATTTGTAAATGCACGTCACCGAGGCATGAATGTTCCCCGGCACGGTTCGTGTGCATGGGCAACTAGGAGAGCAGTTAGGGTGACATGGTAGGGTTGGAGNNNNNNNNNNNNNNNNNNNNNNNNNNNNNNNNNNNNNNNNGTCCATTGTTTTCCCTCCATTTTGGGCTATTGATGTTCCACTTTGGGTATAGAAAATTTTGGGGGAGATTTGATTTAAGTGCATTTAATGGTTGGATCTTTTGTAAAAGTTGAAAAATGTGGACTGGACTGAAAAATGCAACACTTGGTGCCACTGTTTTGAAAATTATCCAAAGTTGTGTAAATTGGGGGAAAAAATTTATGTGATTGTATATTAACTT

>Unigene4516_All

TTAAAATAAAAAATAAAAACAATTATTTTCTCAAATAAACAGCTATCTTCGGTCAAATCATGAAGTCTCATTGATATTTCACTTTCACCACTGCATGAATTTAAGGGCAATTACACCCTTGTGGGAAAGCACTATCAATTGTTAATGATTTTCTTTCTTTCTCCGCTTCTTGTAACTTTGTTCCTTTCCCCCACTTTTTCCCTAAACTAAACCTATCATCTATCTAATACAACAATGGATGTTACAAAAATTAAGGGAAGGACAAATTCAAAGGATGTCTGATGAGTTGCTATCAGATGCAGTTGACGTTCTTAGCCCATCAGAAGTGATGATAGTATCTGTCCCATTGTCTTCTCAGATGTGTCGCCCTCGTTATCCACCTCCACCCCCGTCTCTAGAACAGCAGCATCCAAAACAAGCTTTCGTTGTGCAATCTCATAAACATTCTCATCAACTGTACCCTTGGTCACTAACCTGTATATGGTGACAGGCTTTGTTTGGCCAATGCGATGGCATCGATCTTCTGCCTGCCGATCAATCTGAGGGTTGAAATCCATATCATGGATAACAACAGTATCAGCTCCAGTCAAATTCAAACCCTGCCCTCCAGCTCTAGTCGATAGCAAGCATGCAAATATAGAAGTGTTATTGTTGAAAGCATCAACTATGGTCTGTCTCTCTGTCACCTGGGTACTCCCATCAAGTCTTTTGTATGTCAACCCAATTACATCCAAAGTCCATTCTAAGATGTCTAACACTGAAGTCCACTGGCTAAAAATTAAAACTCTATGCCCATCTCTCCTGAGTGCAGGAAGAAGTTCAGCTAGCGCCCGACATTTTGCTGAAAGCATGACATGCTTATCTGAAAGGATTCCTTTAGTATCTTTAACACCATAATTATATAAAAGCTGGTGAATTGAAAAGTCACTATAGGTCTTGAGTTCTTCAATAACTCTTTCCAAGGTACATTCAAAGCCAAAAGCACCAATTGGATGCAACTTCTTAGCAAAACGAACAACATCCTCATCGAAGTAAATTCGCCTCACTAATAATGGATGATTTGCTATCTTGCGAAACTGAACAAAGTAATTTGAGATCTGCCGCCGAGGAAGAATTCCAAAAATAGCATTCACGTCAACATCCGAAAGCTTTGCAATACGAGCACGTGAAGCTGCACGATATTCCTCTATGGCTTCTTTGTAAGCATCTTCCTGATGCTTTTCCATGAAAACATATTTAACCCGCTGTATCTTTGGAACAAGTTGTTGCATTACATCAGACTTTAAACGCCTCAATATGAATNNNNNNNNNNNNNNTTCATTCGACCAATTAAATCCCTATCTTCTGCGTTTAGTAGCTTTTTCAAATCTACATCCTCAGTAGCAAAAAGTTCAGGCATCATAAACTCCAACAAGGACCACAATTCATGCAAATCATTCTGTAGGGGTGTCCCTGTTAGCATCAGACGCTGGTTTGCATTTCTTGCAACTGACATCAGGTTTTTCCACCTATAGCTGTTCTTATCCTTCAAAGCATG

>Unigene4532_All

GATGATCCCACCCATGTTCCCATTCTCCAAAACTCTCTTCTTCCTTCTCTTCTACACTCTCTCCATTATGCCCCCTCACCTATGCCAACACCCACTAGACCCACTAACCCCAGAAGAGATCACGCTGGTTCAAACCATTGTCCATAGTTCTTACCCCAACTCAACACACATCCTCACCTTTCAGTACGTTGGCCTCGAAGAACCACCCAAACNCCTTTCATGGATAAGAAACCCTTCCAGAAAAAACCTACATCGCCAATCCTTCGTAATCGCCCGTATTGACCAAACCACTCACGAAATCATCGTAGACTTGTCACTCCGNNNNNNNNNNNNNNNNNNNNCAATGGTTATGGCTACCCTTTGTTCACTTTCGAAGAGCAAATCGCAGCCAACCAATTGCCGTTTCAGTACGCACCGTTTTTGGAGTCGATTAACAAGAGAGGGTTCAGTTCAAGATTGAAGAGGTCGTTTGTGGGAGTTTTTCCGTCGGGTGGTTTGGGGAGAAAAAAAGGTCCAAGAGGATCGTGAAGGTGATGTGTTACTATTTAGATGGGACTATTAATTTGTACATGAGGCCAATTGAGGCTATTTCACTGACCGTTGACCTTGAAGACATGAAGATCATAGGCTTTAAGGATAGATTAACGGTTCCAGTCCCAAAAGCGGATGGAACTGATTTCAGAGGATCCATGCAAACGCCGCCGTTTGGACCGCTGTTGAAGGGGATTACGGTGGCGCAACCTCATGGGCCAAGTTTCAGTATTGACGGACACAAGATTAGGTGGGCCAATTGGGATTTTCATCTAAGTTTCGATGCTCGAGCAGGTCCAGTTGTATCTCTGGCATCAATATTTGATGTTGATAAGCAAAAGTTTCGTCAAGTTTTATATAAAGGGTTTGTATCAGAGCTGTTCGTTCCATATATGGACCTAACTGAAGAATGGTATTATAGGACTTTTTTTGATGCGGGTGAATATGGGTATGGTCTGTCTGCAGTGCCACTTGAGCCACTTAGGGACTGCCCAGAAAATGCATATTTCATGGACGCTTATTTTGCTAGCCAAAATGGAATGCCTGTGCAGATGCCTAACATCTTTTGCATCTTTGAGAGGTTTTCTGGGGATATCATGTGGCGTCACACTGAGACTCTGATCCCAGGCAATGTGGTTCGGGAGGTTAGGCCAGAAGTGAGCCTGGTGGTGAGGATGGTTTCAACTGTGGGCAACTATGACTACATAAATGATTGGGAATTTAAGCAGAGTGGCTCCATCAAAGTCACGGTTGGACTGACTGGTGTGCTAGAAGTAAGAGGATCAATATACACCCACAAAGACCAAATAGAGGAGGAAGTGTTTGGTACATTATTAGCAGAAAATACCATGGGTGCNNNNNNNNNNNNNNNNNNNNNNNNNNNNNNNNNNNNNNNTGTTGATGTGGATGGAGATGCAAACTCCTTTGTCAAATCCAGGATGCAAATGACACCAGTGAATGATGACAAATTGCCTAGGAAGAGTTATTGGAGGGTTGTAAGTGAAACTGCTAAAACAGAGTCTGATGCAAGGATTAAGCTTGGCTTGGAGCAAGCTGATCTATTGGTAGTTAATCCAAACAAGAGGACCCATATTGGGAATTTCATTGGTTACCGTTTGATCCCTGGATCAACTACAAGTCCCCTCTTATCACATAATGATTACCCTCAGATTAGGGGAGCCTTCACTAACTATAATGTGTGGGTTACACCGTATAACAAGTCTGAAAAATGGGCAGGGGGACTGTATGCAGATCAGAGCAGAGGGGATAATACCCTAGCTACATGGGGCCTCAGGAATAGGATAATAGAAAATAGGGACATTGTGCTTTGGTACACGGTGGGTTTTCATCATGTACCATGCCAAGAAGATTTTCCAGTAATGCCAGCATTAAGCAGTGGATTTGAGCTCCGGCCAACTAATTTTTTTGAGAGAAATCCGGTGCTCAAATTCAGACCACCTCAATCTGTTCAGTGGCCAAACTGCTCTGCAAATAGATGATGGTTTTAAAATAGTGGGATTTTGCTTGAAGTGCACTCAACATATCTTTTATGTCTTTTCTTCCT

>Unigene4545_All

TAAAGAATCTCATTCCAGGTATCAGGCACACCGGGAAGACACCAATGGCTGCAGTCTTGATATAGGAGTGGTGATCTCCTTTCTTCCGGTGATAGATTCTGCTTCCTGTAGATAGAAGGATGACCATCCTTTCGATAATCTGTCATTTGCGTGACATTTAAGTAAGTGACATGGGTCTTCATCCCTCTTAGCACCTTCTCCAGCACCAACATCTTGGGTGGATATTCCCTTAGATATGTGGCATTCTTGATCGGCTCAACCTCGCTGTCACATGCACCACCAGAATTCCATTGCCCACCACTGAAATGTGAAGCAGAATATCCTCTGAAGAAGACCAAAGACTTCATTGGATTTACATTGGCATCAACCCATCTGGCCCAGGTTGTTAAAGCTTTTCGAAATGCCTCGAGCACATTCAATTCATCATATACATGGCTCCCTTCTTGGTAATAATCTTTCCCTTTAGAAGTTTTCTCATGAGTCCACCAGTGCCCAGTGTTGAAGATGATAATATCTGCAGTTTTATATTGGTCTGACGACCTCCCTACTAAATCAAGCCGAAGAGTCTCCTTCTTTATTCCNNNNNNNNNNNNNNNNNNNNNNNNNNNNNNNNNNNNNNNNNNNNNNNNNCTCTACAGTGCAGTTATAATCTTTGAATATGAAGGAGTATGAAGCTTCGCCTCTAAAATGT

>Unigene4564_All

ATTAGTCATGCCGTGATGAAATCTTTTTGTGTTCCAGAGATGAGTCTTTTGAGCTCGTCTTTGATGGTAACACAACCCTCTAAGAAGTTCTGAGCTGACGCTTAACTTTTCTTCAGTTTTACTGTATGTTTTTCTTTTATTTATTAATTTATGGGTGATTTTCTGAGGATCAAAAAGCGAGGTGACGATAGAAATTCATTTGTCTGTCAATCCACTGACTATTTGTGTGATGTTATCATGAATCCCGACAGGTTCTGATCGCTTCCTAATCCTCAAGCTCACTGCTTTTATTTATTTTTTATTTTTCTGCTTTGGAGGGATTTCTATTTCGTTTTCGGCAATTTGATTTGTGATGGGTCTTTTTTTTTNNNNNNNNNNNNNNNNNNNNNNTTATAATTGTGGCTACTAATCTTTGTTTATCGAGTTAAAAGAAAGGGTTTTGTGTAATTGGGCAGGTGATGAATCTCATATTTTTAATGGGTAATTTGCGTCTGAAACATTTCATTGATGCTTTATAACCTTGGAGACCTGATGCCCTTGCAACTCATTTATGTGCTTTAATAAC

>Unigene4569_All

AAAGTGCTGGAAGAGTTTGTGTGGGCTGTATATTGTAATGGAAGAAAGATTGGCTACTCTATTAGAAGGAAGCAAATGTCCGATGATGAGCTTCATGTTATGCAGCTTTTGAGGGGCGTTTCGATGGGTGCAGGTGTTCTTCCAAGCCCAGATGAGAAGGAAAGTACAGCAGATGGTGAATTGACGTACATAAGGGCAAGGTTTGAGAGAGTGGTGGGATCCAAGGATTCTGAAGCTTTGTATATGATTAATCCTGATGGTGCACCAGGGCCAGAATTGAGTATTTTCTTTGTCAGGGCTCATTAGAAATTCTAGGCTCTATGTCTAAACTTTAATTTCTTGTTTATTCGTAACAGGAAACGAGCTGGNNNNNNNNNNNNNNNNNNNNNNNNNNNNNNNNATCCCATGGATTTTTCAGGGTTGATCAGTGTTTGCAATACCTTAAATTAACACAATTCCAT

>Unigene4578_All

AAATGCTACCGCAATTGGAACACAAGGTCGGAATCCTGCCCTTTTTGTCGTGGGAGCTTAAAGAGAGTTAACTCAGAAGACTTATGGGTGCTCACTTGTAACAACGATGTGGTTGACACAAAAACAATATCCAAGGAGGATTTGTTGCGCTTCTACCTCTATATCAACAGCTTGCCAAAAGATTACCCAGATGCTCTTTTCTTAGTATATTATGAATATCTAATGTGAATTAGAGAAGATGAGAGGAGTGATGTACAGAGAGCAAAAGCCGACCCTCTAATGTAAATAGAGTCTGGTAAGCACACGATTGTTCCTCTGATTCTTAGACCAAAAAAAATAAATCTCAATTGANNNNNNNNNNNNNNNNNNNNNNNNNNNNNNNNNNNNNNTAATGTCAAAGCTCTTGTTGGTGGAAAGGTACATTTCCTCGTGCGTAATATGGAAGAGGCTCTTAAAGGTTCAATTTAAGCTCACAGGCTTGTTGAAAGTGCACGTAAAAGCACTTCTGTACTTGGTTTTGCCATTGAAAAGCATAAACAATGGCAAAACCAACTTGTGTTTCTCGCTTTGAACGATCATAATGGCATTAATTGGACAAAGCCTTGCCGAAGTTAT

>Unigene4631_All

CCCAGGTCCAACTGGAAATTATCAAGTGACTCTGATACAGGCCCAAGCAATGATATTGGATCAGGAACATAACATGGATCTTCAAAAAGCTCAGGTTCCTTAGGCATAAAGTCATGGGGCCCTTCAACAACACGAGGAAAAATGGGTTCAATGGGAGATGAGAAGCCTAAACTAGTGCTACTGGATACATTTGGATATGTTGAGAGAGTAAATGGCGATGGCAGCCCAAAATTGATTGGATTATCAAAAGGTTGTAATGATGATGATTGCCCATGTAACTGTGGGCTTTGAATTTCTGCTTGGGGCTTCAAATTAGGCCTACTAATGACATTTGAGTTAGAGGATGAAGTCATCGATNNNNNNNNNNNNNNAAATAATTGTTGCCATGATTTTTTAGGTGTTGTCCTTGCCGGTGTATCCGAGAGCACAGGACGGTTTACATTCTTATCATCTCCATTCATACTTAAATTCCCAGCTACACGCTCAGGTGGACGGATATCCCTCCTATGGGCAGAAGTATGGCTATGATCTACAGAACTGTTTAACTTATTTTCTTTTGTAACATTCGCAGGAGTATTAGCAGTCTTTCCAAAGAAACCACTTCCTGTAAAAGCTCTAGAAGAAGAC

>Unigene4634_All

TCGCTGGGTGTCCTTGAAAGGAGGAGTGATCATGAATTAGATCCAAATCACTGTATTTCAGCCTTCAATGAATCCTTGGATTGGTCTCTGGGGGAAATTTCTGCTGCTGCCAAATCAAACCCCATCAGTTGGCCTTGTCCTGAGATTTCTTTGCTACAAGATTCCTGTGATGAGCATATAGNNNNNNNNNNNNNNNNNNNNNNNNNNNNNNGGAGCTCAGCAGCAAGAATTGAACCACTTATGTCTGCGCTAAGGGAATGTAAACTCCCTGCTTTTCCCGACGATATATCCTGGTCTGGTGTAGGTGCTAAATCAGTTGAAGAGATTGAGAACCTGAGATCACAACTTGAGAATTGCTTGATCCGATACATGACTCAATCAAGTGGAATGATGACATTTAACTTAGCAATAAAAGAGGCACAGGTAATGCTGCAAAAAAGTGCTCGACTTGAGCTCCATGACTCGTGCTACTATATTATTCCAAAGTGGATCTCAGTTTTCAGACGGATCTTTAATTGGCGGTTGACAAGTTTATCCAATGGTGCAGTCTCTTCCGCCTATATTTTAAGGCATCATCATGTTGATCCGACTCCACTTATTATTGATGAGTTTGTGCCAGAAGGAAATGTAGCTATGCCTTACATAAATCAACCCTCTTTAGATGAAATAATTGTTGGCTGTACTCCCTTTCTACCAATAAGGGGTCAGCCACAGCTGGAAGCTTTTCAACCTCTGCAAGGGACAGTCTCAAACGGTGGGGTTTGGGGGGCTGTTAGAACAAATGACTTGACGGAGAATGAAAGGACTTCTGCACAGTTTGTCACAGATAATATTGATCATGTAACCAGGGGACTAAACACTGCCGGTGTTGAAGCGACGTGTGCTGGCAAAACTGCTAAAGAAGCTGACAAATTAAGTAAGTTGTTGGAACAGTGTAACATATTGCAAAATTCCATAGAGGAGAAACTTTACATTTATTTCTGAGAATGTAGGAACGAGTTTGTTCTTTGTATATATCCTCAATTCGAGAGGAGAGGTGGCCAACTCTACTATTGTAGTGATGTACTCTCATACAAGGAAATAGTAAGAGCTCCATTTTGTTTTA

>Unigene4676_All

GAAGACATAGACGTTATACTCCACCACTGCATCCCCAGTCTTCAGGTCCAACTCATGAGTCCTGGCCTGAGCGTACCCACGAGCAAACCGGAAAAGCCCGCTTCCGCCCACTATCGGCATCTCTCTAACGCCGGAGAATACACTGTTCCGTCCCAAAACACTAAGGTTGCTACCGTTGTATTTCCCTTCCGTGAAGGCGAAGTTTAAGACCATTAAAAAACAGAGTTCGGTTTGTGATGCTGATGCGTAAATCCCTTGTGCTCTTCCTATGAGCTTGGAGCTGGGGTGGGGCTCGAGAGTCAAGGGGTCGTCCATCATCGTCATGAGTCCAAATGCCGATGAGGAGGTGTTGGTCATGGCTGCTTGTGCTACTGGAACGGCGGTGGCGTTTCGCCCACTAACTATGTCGTGGAAGTAGAAGCGAAGGTGGCTTAGTTTCTCTTTCTTGAGGCCTAGTGTTGCCGGAGATAAGATTCTTGAAAAATGGTTAGATTTTGGCGTAGCAAGAGTCGGAAAAGAGCACAGAAAAATGGAAGGAACGAGGAGAAGAAAAAGAAAGNNNNNNNNNNNNNNNNGTTTTAGCCATAGCGATTCGGACCTGGCAGCGATTCTGGGGAAGAAGATGAACGGAACACCAGCTTTAATTGGGCTTACTGGCTTACCTGTTAGCCTTTGAAGTTTGACCCTTCCG

>Unigene4694_All

GCAATTTCCTGATCAATAAAATATTGTCCAGGAACCTCTACATCAACAACATGGAAATCACGTAACACCCTGCTTTCTTCTTCAAGCTTCAAGACGGCAGGAAACCTGTCCTCAACATTGCTCTGAAGAACATTTTTCCAATGTTTCAATCGCTCGGTAAGTTCAGAAAGGGTGGCAGGGAAGGTAACTGTACTTTCTGGATCAAGATCGCGCTCAAAATCCTGCTTGTATTCTCTCACAAAATCAACATGCTTGTTCACAGCATCAGCAGAGAAGCAAGCCCTGCAAACTCCAGAAAGTTCCTTCTTGAGAGACTGAGGAACTTCTGCTGTAGTAGCAGTTGGATACTTGTAACAACGGTGGAGCAGTGCATTAACCACCGCAAGAAGTCTCTCCTCTGGCAAAGTAACAAAACGTGAACCAATTTCTGTGAGTAGGACCTCAAGTTCACTAGCCAAATTAGTGTGTTTGCTTCTAAGGGCCTCCATTATGTCCTTTGCAGCATCAAAAGCACTGGCAGCAGATGCAACAAATCCCAGAGCACCACGCCTTAATGCATTCTGACCGCTATCATTGATAGTTGAAGAATTTTGTTGCAAAGGTTGATCATTCCCCGCATGGACACTGCTCTCAACTGTTGTGGCTGCAGACCTTTCAGGTTCCTGCCCATGAGAGTTTCCACCATCATGAGACCCAATTCCACTACCAGGTTGAGGGCCTTGGTGAACTTGATTGTCTGTGGTCTGTAACCTTGCATTTCCATCAGATATTCCAAGGGAACCAGCCCCAGCCCCAGCCCCAGCCCCAGGCACATTTTGCTGCATTCTTTGCTGCGCCATTGCCAATCTGCCTAGTTCGGATTTATTTGCAACATCACGACGCNNNNNNNNNNNNNNNNNNNNNNNNNNNNNATACAAAGCCTGTGGATACACAGTGGCAATCTTCAACAAAACAAGTTTGCAGTGAGGTGCCTCTGCCCTCTGCAAGGAAAGTAAAAGTTGTGGAATCCACAAGAAAGCCATACCCAGTTGGGTATTTGGTCCAAGTATTTATCAAATGCCCTCCCCACAGGCTCATTGGGAGTATCAAAGCTCAGAAGATATAGAACCCGAGCTAGATGGCTTCTGGAATTGGAAACACCATATTTGATACCTTGAAGAAAGCAGCTAACTGCATATTCAAGCCAAATCTCATCATGGGTTTCTTTGAAAGCCATATCACAATAGTTTCCCCAGCTTATCCATCCTTTTGGCAAACCTTTGAAAAGACTGATGGCATGTGAATATGCAAGATTAGCATTTTCAGAGTCGCTTAGTTTTAATAGAAAATCCCCCTTGAGACGAAAAATCTCTGCTTTGTGTTTCACAGCAAAATATTCCAAGTTAGTGCTGTTTATCAAATTGAGACCACTTGTAAGCTCTCCCTTCATCTCCAGATAAGCCTTTGCTTGTTCTCTAATCTTGACAAAGGCTTCCTGCACTTCCATGGTTGAATGGCCATACATCTTCTCAAGAATTATCACACAAACATCATAAAGGCCTTGCTTACGAGCAATATGAGCAAGCTTATTCACATTCCACGCTTTATCACGATAGCCAAGGTGATGAAGTTGTGAATTTGTATTGCCAAAATCCTTGAATGCATCAATGACAGCATTATACATTTCATTCCTCCACTGCAGCAAATCATACCAAACAGACATATTATCCCATTCATTTGGTGTTCTTAGTCTCCAAGTCTCAAGAATGTCCTTGAGATCTGCATACAGATTTCCATGCACCCCAACAACAGAACTGCCAGAAACTTTGTTCCCATTGGCAATATCCACCAGAATTCGAGCAGACTCTTGAACTTCAACCAGCTGCTGGAATTGTTGCAAAAGAGGAATCCTAGCATGAACAGACATTTCAGGCAACTGCCACCAATGTTCTAATGCAAGATCAACACCCTTCCCCACTATGTTTTCAGCATCCCCGACACCATTTGCATTTCTATCATGAAGGGCAAAAAAGGCCTGAATCAGACGAAGTTTTGGAGTTTCTTCAACCTGAGCCTTGGGAATAACATGATCCTTCATATATGTCCAATCAGGTAATTTCCAAAGGGTATCAAGAAGAATTTCATAATTCTCAACACTTTTACCAAAGTCAATTAAGGCATCCCACTGACTGAGCTGGTTCGCACAATAAAGCCATTGTTCTTCCCAAAGACACATCTCAGCCTTTGGTACTGTGTTGTTGTATGTTCCTTGAGTTGCCTTTACCATGGCCTGGTAAAAGAGGCTTTGAGCACGCTGCCAGTAACCATGCTGGACTAGTGAAAGTCCAGCTCTGGTTTCCGCTGTGATTGACCTTTTCTTCCACAATCCACACCGCATATCTTCTTCATTGAGCAAACGATAAAGCTCAGCCAGTGACTCGGAGCACTTCGTGTCATTCATAAACAACATGACATGACTTTCCAGTAAAGCCAAGGCAATGTGCCATGCATTATAAGTCTTTCCAATATATTTAATGAGTTCACTTGGCATCCGAGGTTGAGGGTGGCTCAACTGAAGACCTTCTAGAAGTGCTTGCACAACATTTGGTCTGCTGGCTTGTTGCTTCTTATGATAATCTTTTGAAAGAAGAGTAATCATCGGTTTAGCTAATGTTACCTGTTCTTCCTTATGCAAGGTCACCCAGACAATGGGGAATACCAGCACCCACAAATGATATGCAACATTAGCATCTGTATGCGCCAGCTCTCTCAATGGAATAACAAGATCAGCTACCTGAAGTTTGCTCATCTCATTCAAAAACTGAGCGTGCTTGAGGACAAGGCTATCAAGTGTTAAAGGTGCTTCCTCAAGACCCCCCGGAACATCAGTAACCTGTTGTTGCATCCCAGAACCATCTGGAAGAGAATTTGAGACCAAGAGTGGCAACACTCTGGCAGAATTTGGAGCAAGCGTGATGGGTTTATCCTCAACTAAGATAGCCAGAAGCAGATCAAGACCTTGTTTGAGCCAGAAAACATCACTTAGAGCTTCCCAGTCCTGGACTTGGATGATATACTGCAGTCTTGTAAACAATGTTTTCCCTAGAGATTCATGATAAAGTGAGAAGAACTTCATCCTAATGTCAGGATCCTTTGCACGTAAACCAAGCATGAATTGTCTTTCCACCTTCTGAAACACCTCTTGACGTAAGGCCAGTGGATATTTGTTCGAATCAGCACAAATTCCATAAAGGAGCTGAAGATATTTTCTGTTCCACTCTTCCAAAGCATCAGGTTGGAAGTTCTGCTTATCAACTTGCGAAAGCTTTTGCAGAAAAGAGACTATTTCCTTAGGATTAAGAAAAGCACTTGAAGGGACTGCTCCCTGTTTGCTAAAGTCATCTTCAATCCACCCTTTTATCACATCTAGGATGCAAAGAAGCACACTAGCATCAGTTCCTTTCTCTGAGAGCAAAGAATTCAAGATTTGAGTTACAGATCGTTTGCAATCTGTGACAACCATGACTTTTTCACTGATAAGCTTCAATACAGATTTCAGATTAGAAATGACAGCACCAAGATCAGAACCCTGATGGGAAGAAGAGACTGCTGAATCTGGATCTGCTCTCTGACCTTGTCTCAAATGAGAGCCTGCTGATGATCCC

>Unigene4695_All

CAAAGCTTTCCCACATAAAGTCTACATATAAACCATCCCTGGGAGAGAATTCAAGGGGTTATTCTCATCACTTCTCTCAAAATATGGGTTTATGGAACTACCATCTAGAGGACAACAATCTTTGCTTTCAAGCTCCCACTTTCATTGAATGGCTCAAGCCATATTCCTCTCCTTCCTCTTCCTCCTCCTCCTCTTCCTCGCTTACCTCATCTTCTTCCTCCTCTTCTTCTTCTGTGACTCAACAAGTTCAGCTCACAAACCCCATGAGCATACTAAAACTTCCTCTCTTGTACCCACAACAACAGCAGCAGCATCAGCAGCCAGAATTTGTGAAAGAAACCATCCAATGCTTACCTCTTCTAAGCAGACTCACAGAGAATAAAGCATTGAAAGAAGAGAATATGGGGGTGCAAGAAACTAGTTTTGGACTTAAAGAAGAGAAAATTGACAAGGTTACAGTGGCTTTGCACNNNNNNNNNNNNNNNNNNNNNNNNNNNNNNNNNNNNNNNNNNNNNNNNTTGATCTTAAAGAACAACAACAACAACAACAGCCACCACCCATCAAGAAAAGCTTCCAAGG

>Unigene4724_All

CGATATTTCTTCACTAGGAATCACTCTACAATAGTTGCCTTCGCCATTGGTAAAAAATACGTTGCTGGAAATGGATTTTATGTGATCGGCGCTCACACTGATAGTCCTTGCATCAAGTTGAAGCCTAATTCCAAGGTAACGAAAGGTGGGTTTTTGGAGATTGGAGTTCAGCCATATGGAGGTGGTTTATGGCATACATGGTTTGATCGTGATTTGACAGTGGCAGGACGGTTGATAGTAAGAGATGAAAAGGATGGCTCTGTTTCCTTTTCACATCGTCTGGTTAGAATCGAGGAGCCCATTATGCGTGTCCCCACACTGGCAATTCACTTAGACAGAAACGTCAATAGCGATGGGTTTAAGGTGAACACGCNNNNNNNNNNNNNNNNNNNNCGTCCGTTAAGGCAGAGCTCAATAAAGTGATTGCGAAAATTGGTCCTGATGGAAATGGGGAAACTGATGGGAAGAAATCGACCATTTACTCAAAGCATCACTCAATTCTACTAGNNNNNNNNNNNNNNNNNNACCAGATGATATATGTGATTTTGAATTACAAGCATGTGATACTCAACCAAGTATAATAGGTGGTGCTGCTAAAGAATTTGTTTTCTCTGGAAGGCTCGATAATCTTTGCATGTCATTTNNNNNNNNNNNNNNNATTGATAGATGCTACATCTTCAGAAAGCGATCTTGAGGATGAGAGTGGTGTTAGAATGGTGGCATTGTTTGATCATGAGGAGGTTGGATCTGATTCAGCACAAGGAGCTGGGTCTCCTGTAATGTTAGATGCTCTATCACGGATCACAAGCTCCTTCAAATCAGATTCTAAGCTGCTTCAAAAAGCAATTCAAAGGAGTTTCCTTGTATCTGCTGACATGGCACATGCACTTCATCCCAATTATATGGAAAGACATGAGGATAATCATCAGCCCAAGATGCATGGGGGGCTCGTGATCAAACACAATGCAAATCAACGCTATGCAACCAATTCGATCACTTCCTTTCTATTTAGGGAGATAGCATCAAAGCACAACCTTCCTGTTCAGGATTTTGTGGTCCG

>Unigene4749_All

TTTCTTCCCTTCATGAATTCTTTGACCAAACTTCTAGATATCATTAATTTCAAAATTGGTTGCTTAACTCTCCCTCTTCCCAACAGTTCCAAAAAAAACTCTCTTATGGCAAGGCTCCCCTTTTCTGGTCTTTCAACCATTGCTGTTCTCATCATTTACCTCCAAGCTTGCAATGCGCAGGTTAGCCATCTCTGTGCTCCTTCTTCTTGTGGCAATCTCCATAACATAAGCTATCCTTTTCGATTAAAAGATGATCCACAAAACTGCGGCTACCACGAGTATGAATTGTCTTGTGAGAAAAATCAAACGGTTTTGTACTTGTATGCCGGAAAATACTATGTGCAAGCAATCAATTACGATAAGTCCACTCTTAGAGTCGTTGATGCTGCTGTTCACAAGGATAATTGCTCCTCCCTCCCAAGATATTCTTTGACTGCAGCCAACTTCAGCTATATAGATCCTTATGACTTGAATTCACCCCGGTGGAATTTCAGTAGCAGACCACCGAAGCTTATTCTGTTCATGAAGTGTGCAAATCCAGTGAATTCTCCACTCTATGTGGACTCTTCTCCTTGCATTGATGCAAAATATTCTTATGTCATGGATAACTTTACCAGCATAACAGATATTGAGAACTCCTGCAGTGTAGAGTTGATGGTTTTGTCATTGTTACCTGCAAGGGTTGGCAAGAACTTTTCCTTCATGGATATCCATAATGATCTGGTATATGGATTTGAGCTTTCATGGTCTCAAATTTTCTGTCAAATTTGCAGTGGTCGAGAATTTTGCTTTCGCGAAAATGGCATAGATAGGCCCACCGGCTGTTCAGAAGATTTCTTCAGGGACTTGAGAGACAGGATCAGAGCCAGCCGGTATGCGATACTGATGATTATTGGATTACTCATAGTTGCAAAAACTATTTGTTGCACTCCATGTGTGATGGCGTTCTTGATTTATACATGGTCAAGGAGGCATTTATCAATGTATGACAGCATTGAAGAATTTCTTCAAAGTCAAAACAACTTTGCCCCAGTAAGGTACTCTTACTCTGATATTAGGAAGATGACAAATGGCTTTAAGAATAAGTTGGGTGAAGGAGGTTATGGCTCTGTATACATAGGGAAGCTTCGCAGCGGCCGCCTTGCAGCTATAAAGATGTTAGGGAAGTCTAAAGCAAATGGTCAAGATTTTATCAATGAAGTAGCATCAATTGGACAGGTTCACCATGTTAATGTAGTACAACTAATTGGTTTTTGTGCTGAGAGATCAAAACGTGCTCTTGTATATGACTTCATGCCTAATGGGTCTCTTGATTTTTTCTCGAGAAGGGCATGCATACTTAAGTTGGAAAAAAATGCATGAGATATCTCTCGGAGTGGCCCGCGGCATTGATTATCTTCACAGGGGTTGTGAGATGCAAATTTTACANNNNNNNNNNNNNNNNNNNNNNNNNNNTCTTCTTGATGAGAATTTTGTTCCAAAAGTTTCTGACTTTGGGCTTGCAAAACTTCACAAAACAAGCGACAATACAGTGTCTCTTACTGCAGC

>Unigene4767_All

AACCCACTTCCGATTTCCCACCAGTAAATCAAAACTCCTTTTCCTTTTTCTTTCCTATAATTAAGCACTTTCTTCCATCTTCTTCTACGTTCTCAAGAAACAAACACCCTTTCAAACATGTCTGCTGAAGTTCTTGTTGTCCCTGCTTTTGGACAGGGCCATCTCTTTCCTTGCATCGAGCTATGTAAGCTCATAGCTACTCACAACTACAAAACTACCCTTGTCATTTTCTCTTCTCTCTCCTCTTCCGTCCCCTCCACTTTCCGGGAAAACCCACTTCTCCACATCCTTGAAATTCCATCTCCGTCTGGATCCGTTCCGATTCCCATGCATGCTGATAGCCGGAAACAGATGCATTTATGCCTTGAAAACCTCCTCTCTAATCGCTCCCAGAATCCAAAACTACCCATTTGTGCCATTGTTGATGTGCTCGTTGTGATGAGTTGGTCGGCTGATATTTTCAAGAAATTTCATGTTCCAACGATTGGGTTCTTCACCTCCGGCGCCTGTTCCGCCGCAATGGAGTATGCTGCATGGAAGGGACACCTGCAGATACAGCATATAAAACCTGGAGAGATCTGTTTTCTTCCAGGGTTGCCCGAAAGAATGGGTCTAACCGTCTCGGATCTCAAACGCCGGCCTCATGGCCATGGGCCTCCTCCACCTGGACTTGTAGGTCCACTAGGTGGGCCATCCCAATCGAAGCCGAAGATAATGGGCCCACCGGCGCCTGGAGAGCAACCACCGTGGGTTGATGACGCAGAAGATTCCATCGCTTGGATGATAAACACGTGTGATGATCTGGAGCGTCCATTTATTGATTACCTGGCCGATGAAGTTAAAAAGCCTGTTTGGGGCGTGGGCCCGCTTTTCGCAGAGGAATATTGGAAATCAGCTGGCGCACTTGTTCATGATAGCAAAATGAGAACCATTCGACTGGCTAATATTACAGAGGAGAGCGTGATCCGATGGCTAGATTCAAAGGCACGTGGCTCAGTATTATACGTCTCATTTGGCAGTTCAGTGGACATCACTAAAGAAGAGTATCCACAGTTGGCTGACGCATTAGAAGCCTCGACCCATCCATTTATATGGGTGCTCCGACCAGATGCGGGTCGGGCGGGTCCTCCACGTGGACAGTCCGATCCAAATAATCCAGAAGAAGGATATTACCCACATGGGTTGAGCGAGAAAGTGGGTGAAAGGGGATTGATAATATGTGGATGGGCACCACAATTGTTGATACTGAGTCATCCATCAACAGGTGGATTTTTATCACACTGCGGATGGAATTCAACGATGGAGGGGCTAGGAAGAGGGGTCCCATTTTTAACATGGCCCTTAAGGGGTGACCAATACTATGATGCCAAGTTGATAGTGAGTCATCTTAAGGTGGGATACAGTGTATGTGACGATTGCTTGGAGAAGGTAAAGAAGGATGATATAATGAAGGGAATAGATAAGGTAATGGGAGACAGTGAAATGAAGAAAAGAGCAAAGGAAATTGGAGCTAAATTTGATAAAGGANNNNNNNNNNNNNNNNNNNNNNNNNNNNNGCATTTAGTGATTTTATTAAGCAAACTGTTGCTTAAAGCACAGTCGTTTTATTTTATATGCTCATTTCCTTTTATTTTCTTTTTTTTTTCTATAATAAAAAGTTATTTATGCC

>Unigene4770_All

CAATTCATCGATGAAGCTGAATCGAGGAAGAAGAGAACGAGGAGGCACAGGGATGAGCAGAGCTTGTACATGGAGCTGAGTGTGGCTATGGACTGCTTGGAGCACATATGCACCGAAGGGTGCACGAGCGTTGGGCCGTGTGACGTTCAGCCCAACAAGAAGAGAGGCCCATGCAGCAAGTTCTCCACATGTCAGGGCCTGCAGCTTCTGATCAAGCACTTCGCGACATGTAGGAATCGGGTAAATGGAGGGTGCTCCCGTTGCAAGAGGATGTGGCAGCTACTAAGACTTCATTCTTCCCTNNNNNNNNNNNNNNNNNNNNNNNNNNNNNNNNNNNNNNNNNNNNNNNGAAAATGCAACAAGAGAAAAAGGGCGATGATGCACTATGGAGGCTGCTGGTAAGGAAGGTAGTGTCGGCCAGAGTGATGTCTTCTCTCAACCTGCCCAAGAGGCAAAGAGGAGCAACTAAAAGAC

>Unigene4776_All

TAATAGTAATCCGCCCAAGAGCCTTGGGCAATCTTCATCACCCTTTGGCAATACGGGGATGCTCAATCCATCGATGCCCATCAACTCAGCATTCTCACAATCACAATCTCAGGGGCAAATTAGCACTGGCTTTCAGGGTCAGTTCCAGCTGTCCCANNNNNNNNNNNNNNNNNNNNNNNNNNNNNNNNNNNNNAGCAAGCTCATGTGCAAGCACAGGCCCAAGCAGCTCATGCTGCTCAAATTCAAGCAGCGCGTGCACAGTTCCAAGCTCAGTTGCAGGCACAGGGAATGTCTCTTAGCCAGGCCCAAAATGCTGCCTTGGCCAATTTAGGTTCATCCTCACCTTCGTTTTCGACTCCCGGTAGCCTGAACGCCAAGCGGCTACCTCTAAAACCCCCAGTTCGGCCTCCTGCTGTTCCCATGCCAACCATGGTTTCCCCATTGAAAACTATGGATCTCATCTCTGCAGCTCGCAGGAAGAAGCAGAAGCTT

>Unigene4824_All

CAAAAAACTCCATCACTGCTAAACTCTCCCACTCTTCACTTCTCACTTGCTTTACTTTCCTCCTCTAAACATCTAACTTCATAATGGGGACCAGAATTCCTTCTCACCAGCTCAAAAATGGCCTTCTAGTTTCAGGTCCACCCGAGCACCTCAAGGAGAAGCAACCCACAATGGCATCACGCGCCGTTCCATACACTGGTGGTGACGTCAAGAAGTCTGGCGAGCTTGGCAAAATGTTTGATATCCCTGTCCTCGATCAACACTCGCCTTCTGGCCCTCCTCCCTCTATACCCAAGCAACCCTCCCGCCCTTCCTCCTCTTCTCAACACAATAGCGGATCCGTCCGATCCGGGCCCAACTCCGGCTCAGTTAAGAAATCTTCCGGTCCCCTTCCCCTTCAGCCCACCGGTCTCATCACCTCCGGACCCCTCGGCTCCGGTCCATTGGGCTCTGGCCCACTCCCTTTGGCCCACCGGAGATCCGGCCAGCTCGACCATACTGGCTCCGGGGTTGGATCCGGCTCTAGCAAGGCTCTCTACGGGTCAGCGGTCACCAGCTTGACGGAGGATGTCAAGGTAGGTTTTAGGGTTTCGAGGCCCGTGGTGTGGGTGGTATTAGTGGTTGTGGCGATGGGATTGCTTGTGGGTGCATTCCTGATGGTGGCAGTTAAAAAAGCGGTTATTTTGGTGGCAGTGGGGGGGGTATTAGTTCCNNNNNNNNNNNNNNNNNNNNNNNGGGAAGGAGAGGGTTGTTAGGGTTTGTTATGAGGTACCCTGATGCTGAGCTTAGAGGTGCTGTTGATGGACAATACGTCAAGGTCACTGGGGTTGTCACCTGTGGCAGTATACCTCTGGAATCATCTTATCAGAGGGTACCCAGGTGTGTATGTGTTTCCACGGAGTTGTATGAATATAGGGGGTGGGGTGGAAAATCTGCACAGCCCAAGCACCGCTTCTTCTCATGGGGATCTAGGTATTCAGAGAAATATGTTGCGGATTTTTA

>Unigene4842_All

TTATTCTCATCTTCGTGTACCAACAAAGCCAGTAAATACAATTACAACTAACATGGTACAATGGATCCATCAATTAATAAAATTAAAGAAAGCAGACATAAATGCAATATATCAAGGAAATATAATAAATATGCGTTTTTAAAATCATTAGCACTACCAAATCATTGCTGACTTCGATTTAATTACCTGGTTGGATTGCAAGCCGTCATGAATTTGTGTACACATGGGGAGCAAGCCTAAATATTCACCCAAAACTTTTATTTTTTTTTTCTATTACATAGAATAACACGCACCAATGGGCCATAAAAACGTCAATCGATGAAGAGGCTAGTACACAAAGTCGATACAGAAGCAGCACTTAACATGGAGGAGAGGTCTTCCTTCCAGCCATGACACTGTCTTACCATGTCCCCAGCGAGTTTCGCCGAGAATTCTCATCATATCCCAGTATTGGGGTTATTTCCAGCAAGAATAAGAGAAATTTCTAATCCACGGCTGAAGGCTTGGTTGAACATGAGCCTGGTTTGGAAAGTTGATACAAATGGGAACCATGAAAATAAGGCAATAGGAATAAAGATGAGCATTCCCATCCCGGCATCATACAGACGGGCAATTGAGCGGATGGATTTCCATAATCCTAGCTTCTTCATCAAAGGCTTCCATGCAGCAGCAATAGATAGAATTCCCCATCCAGTAGGTACAAATGCCAAGATGCAAGCAAAAATGTCTGGAACTGACAAATCTGTGAATACAACTGCAACAGCTAAACCAGCTAATGCCAACAAGAAGGAGACACCTTGGACGAAGCGCAACAAAAGCTGAAAATTGACAGATATCTTCTGACTGAAGGTGAATACCTTGAAAAGAACCATAAGCACTGCTAGAACAATCCATGAAAAGCCATATATCGCTAATGATGTATTTGATTTTTGTATATCTAGCTTGTATACAATGCCATACTGGAAAATAAAGAATCGTAGACTCAAAATGGTCTCCAGTATTCTCCCTTTCAAAGTACGGATATGTGCCAGTTCTTCATCCCACCATGCTTCCCAGCTTTCTTCTCCCTTAACCCCAATTCCACCTCTGTAAAGAAGCCAATTTGTCCAATCCCTGAAGTCCTCTACTGTCTTTTGCCACTCAAACGCAGATGGATTAAACAAATAGGGAGCAAAGAGCCAAGAAAGAGCCATATACCAACTGCTAACTGTAAGAAGAATGTAAGACAGTGCACCACCTTCATTATATCCATATGCAAGGTATACTACCAGCAAAAGCACAACTTCTAGCCCTTTAACAAAATGACTGCGGGAATAGAGCCTATAGTTTTCAGAAAATTTAATATGGCGGACCACAAATCCCCTACCAGTGGCATGGTACCTTGCACCACCATGAAGAATGGTTCGACCAAAATAATGTGTTCTAGTGCCCAATGAAAATGTGAAGAAGACAGAACAAAGCTGAAGCTGCATTGTGACAAAACTGACAATGGCCCTCAGAAAACCCTGCTCCAAGATAAAGCCCAAAATCATTGGCACAGCAGTAAAAATACCAATTTGAAAGAGGAACTGTGCATTTAGAGCAGCACTGAGGGCAGTGTTCTGTAGTATATCAGATCTTACTTGAATTTCTTCGCCAACTCCAGATAGTGCCAAATATGCCTTCCCATAGAGGAAGATGTATACAGTCAGCACCGTTAACATTGTACAAAAATAGTATCCCACAGTAGTAAAGTAAAATGACATCATTCGAAAGAAATCAAAGAGCTGCCCGAGCCTGTACACATCCCGACTAAGAACTTGCTCACCGTTGCCACCAGCAACCTTCCCTTCAAACAAAGCAATCTGGTTGAGACCAACATCTCGTCCTTTGCCAACCTGAATATATTCATGATGAGTAATATTCCCTTGGCGCAAAGTTGAATTGAACCCTGCATAAATGTCTTCACTAATGTTGATAACTCGAGAAGCCTTGCTGATACCGCCTCGAGTTATATGGAAGACTCTATCAAAGACATCTGGATGGCCATAATGCATACGGACTTTCAATGGATTAGCCAAAACACGCTGACCAAGAGTCACAAAGCTAGTTTCCTGATTTGACATAAACGAAGCTAAAGAAGAGACACTTCCAGTAAAAACATGTTCTCTAACACCAAGAATTGTTGGAGGATGAATACCATGATCACGATGGAATTCTTCTAGAAGATTTCTCATCTTTAAGGCTTCCTCAAAATAATTATCCTGATTCATATCAATTGTCTGAACCGCATTTCCACGAGTAAATACAATTGCATGGTTTTGATTCTCAGGTTTTCCTTCTCCAAGTTTTGGATTTCCAGGCAACTTTATGGAGTAAATTTCCTTGTCTTTTCCATTGATATCAGCCTTCACAAGCTTTGAGTAGAATTCTCTCTGTACTTTACCATCCTTCAGAGTTTCAACTTCATCAATAAAAGCAACACGAAGAGCTTCATTTCTTTGCATGAGTAATGCAATATCAGCTGCCTCAGGTTTTTGGTCCTCTTTCTGTTTTCCATATATTTGACATGTAACCACATATGTAAACTTCAGATCAGCCTGAGCTCGTGCCTCAGGAGACAAGTCAAANNNNNNNNNNNNNNNNNNNNNNNNNNNNNNNNNNNNNTGCAGATTCCATATCCCCAGCGGTTGCTCTTTCCAAGTAGCTTTGAAGCATAAGAGCTTTTCTATAGTACATCATTCCACGAACTGTTCTGGCTAGCGTTTGTCCNNNNNNNNNNNNNNNNNNNNNNNNNNNAAAACCGAAGTTCAAGGATATCACTAGGACTATCAAAGAGTTCTGTGTCCATAGCATTTTCATCACGGCCAATTCGAGCAAGAAAGTTCTTCCACTCATCTGGAAATATTTTCTGGAGGTAAAACAAAATTGATATTCCATCCTCATTTTTCTTTTGAAGTTCAGCCATGCTATAAAGAACAATCTCCGAATAGTAAGGAGTAAACACACTGAAAGACAGCATTTCACGGACTGGCTTTGCCTCAGGCATACCCATAAAAAGGGAATTTGTAAAGAATTCTAGCCTACGTCTCGCTTCAATATTTTTGGGAATATTTGAAGCAGATTCTTTGATGGTTAAAAGTGCATGCAATCTTCTGATCTGTGTTTTCAATTCATTGTTTCTAGGCCACTTCAAGTCTGTAAACAAACGGCCTTGAGACCATGCTTCGGACAGTACATTCCACGTCTCACAGTGCTCCCTCATGATGACAGAAAGGAAATCAGATCGCACAACATCATAAAGATCTTGAACAGCTTTAATTGCACCTTTTTGCAGTTCTGGTGTCTCAGATCCTTTC

>Unigene4860_All

GTTCTCCATGTGGTAAGCCAGGAGTCCACACAAAAAGCATGGAATTTGTGTTGACATGGCAGAATCCTGAGCTTCTCTCCAACACTATAGTCTTCAAGGCATATGGCACATGTTCTTGAAGTACAGTTATCCTCTAGAACAGCTGTGAATATTAGGCTTGGCATTGCTTTCACCAAGCGGCGGCTCATTCCATGGAACTCCCGGACACGAGAAGAACGAGGCTGCTCTCTTCTTATACGATGCCTACGAATAAAGAAACAAGTAGCCAGCACTGCAGACATGGCCAGCAGAGAAATAAAAGATATTGCCATGATAGACCATGCAGAATTTTCAAAGCTTGGGATTAACCAAAGCTCCATGTCTCTCAGCCCAGCATACTTCTTGAGTGTTTCACCAGAAGATTTAGAAACAAATACAGCATGTATTTTTATACCAGTTGAAGTCCCTGCCATTGAAANNNNNNNNNNNNNNNNNNNNNNNNNNNNNNNNNNNNNNNNNNNNNNNNNCAGCCTTCTGTGCTCTTCTAACTTTATCCTCAAAGCTACACCCTCCTCTTATAATTAAAACATATGGGGAAGTAATATTTGAAGCTTTTTCAACTTCATTTGTCAAATCTGAACACGCATCAAGGGGCTCTGCCAAATACAATACCCCACATTCACCGGAGCTCTTAACTGCTGGAGCGAAAATAGCGTCAACGTCATCGAAAGACAAAGTAACATCCCTGCCAATCAAAACCACACTCGCCGAAGTCATCAAACAGCATGCGGATAAAGAGAAGAAGAAAATCACGACAAAAACAACACCACCTTTCATTTTCGATTCCCCAAAGAATCTCACAAAACCCCAAAAAGAATTATAGAGAAAAAGAGATTCAGATATGCAATTTTAGGGCTTTGATTTGGAAGAAGATGGATACATCGGCCTCCATTAGAGAGAGAGAATAATTCTC

>Unigene4905_All

GCTCCACTGGCACTGCCTAAATTTTTTTCTGCTTCCCCAACCTTTAGAATGTTCTCAAGTCCTTCCAAGCAAACTGTGACAATTCTGGGGTCAGGACAGATCAGGAGATCACACAATGGCTTGATACACCCCTGGCTAACCAAGAACTTGATTTGTTCATGGGTGCCACCAGAAGTAGCATTTGAAATTGCCCAAGCAGCCTCTTTCTTGATCTCAAATTCAGCATTCTGAAGCAGTTGGACAAGGGGAGCAATGATACCGGCTTCAATTATGGTCTGTATTTGATTTACATTTCCAGCTGTGATGTTTGAGATTGTCCAGCATGCTTCTTTCTTGATACTCTTCTTATAATTATTGGTCAGGAGGTTCAAAAGGCATGGAAGAGCTTGATGGTTTATCATACACTGAGTCTGCATGTCATCACCAGTGACAATATTTCCAACTGTACGTAATGCAGGAATGAGCACTGTTGGAGATGAATGAAGTAGAAGTTCAACAAGCCTGGGGCAGACTCCTGCCTCAATAACAGCTTGGATTTTGTCATTGGTTCCATCTGAGAGATAAGACAGTGCCCAGCATGCATCCGTCAGGACTTCCTCATCATTTGAATGTATAAGACGCTCAAGCGCTGGGAGAGCGGGCTTTGTCTGCTCAAACAAAGGTTGTGGCTTGCCCCTGCAGAAGTTAGACAATGTCCAAGTTGCATTTCTCAACATAGAAAGCTTTGCATGTTCATTAAACTGTGCAAGTAATGGCATCAAAGTCCCATGGCTAAGAACAAGATCACGACACTTAGGTGAGTCTCCAGCAATGTTTCCTAGTGCCCAAACAGCCTGTTCCCTGACATCGTCAGTGGGTGAACTTAAAAGCTTGACAAATATTGGCACAGCCCCATGATCAATTACAACCTTGGTGTTTTCTGATGTCCCAGAAGCAATATTTGTGAGAGCCCATGCTGCCTCAAACTGAAGCTGTGGAAAATCATCCCTTGCAAGGAACTCAATGAAACGAGGAACAACACCAGATTGTACAACTTCATTGATTGGAGGATTGCGTTCTATTGAGAGCAGCTTTCGAAAAAGAGTGGTTGCCTCAAGCTGTGAATTCTTGTCTTCTGACCAAACACCTGCAACCATTGCTGGAANNNNNNNNNNNNNNNNNNNNNNNNNNNNNNNNNNNNNNNNNNNTGCTGTTGCTGATGCTGCGCCTGCACTCCCTCGCGTCGCTTCTTCTGCAAGTTCTCTTCTCTCTTGTTCTTCCGAATCTCCACCATGTTGTCCTCTCTCCTCCTTCTTCCTTCCTCCGCATCCACTGCCACCTTGTACTTGTTCCTCCGCACCTCCGTCCTGGCGCTCGGCCTCAGCGACATGGTCGGCAATCGAAAAAAGTCGAATCAAAGATGAAAGCAGAGAGGGCGATTGTGAGAGGAAATGAGCGGTGGCGTTTGATAGGTAGCAGAGAAGGGAGGGAGTGTGGGAGAGGAGGGAAGGGTTTTTG

>Unigene4921_All

TTCTTACAGGAAACAATTGTTAGTATGGCTCTGGTTGGTGCAATAATTGGAGCTGCATCAGGTGGTTGGATTAACGATGCGTATGGACGGAAGAAGGCTACGCTTATTGCTGATGTCGTCTTTGCAGTTGGATCAGCTGTCATGGCAGCTGCACCGGATCCATATGTTCTTATTCTGGGTCGACTCTTAGTCGGCCTTGGAGTGGGTATAGCATCTGTCACGGCTCCTGTGTATATTGCAGAAGCATCTCCATCTGAAGTAAGGGGTGCACTAGTGGGCACAAATGTTCTCACNNNNNNNNNNNNNNNNNNNNNNNTCTTTCCTATCTCGTTAATCTTGCTTTTACACAGGTCCCTGGAACATGGCGGTGGATGCTTGGAGTTGCTGGTGTGCCAGCTGTGGTTCAGTTCTGTCTTATGCTCTGTATGCCAGAATCTCCACGTTGGCTTTTTATGAAGGATGATAAAGA

>Unigene4927_All

CGAGTACATCCGACGTAACCCAACCTAAAAGCCATCAAAGGAATAGACTTTGGAATTTCCTTTGGGAACCATGAAGCTCTAGTTTCCAAATCCCATTGCACCACCGTAACTTAGGAGACACTTTTGAGCTCGCTGCATATAATTCACTCACACGCTAAGCCAAACCAAGTCTCGAAGTAAATTCATCATGGACCCTTCCTCCGCCACTCCTCTCCCCCGCCAAAATCCGTGGTGGCGCCGCCATGTGCTTCTCAAAACCAGCCTCTCCTCCGAACTCTCTGGAGCAGTGGGTGACCTTGGCACCTTCATCCCTATAGTTCTCACACTCACTCTGGTTTCCCACCTTGACCTTTCCACCACCCTAATCTTCACCTCCCTTTACAACATTTCCACTGGCCTCCTCTTCGGTGTTCCAATGCCCGTCCAGCCCATGAAGTCCATCGCCGCCGTTGCCGTCTCTGAGCTCCCCCATCTCACCACAGCCCAGATACCACCGCCGCCACCCTCCTTATACTCGGTGCCACCGGCCTCATGTCCTTCTTCTACAGCTTCATCCCTCTCCCTGTTGTCCGCGGCGTTCAGCTCTCCCAGGGCCTTTCTTTCGCCTTTTCTGCCATCAAATACATTCGATACAATCAAGATTTTATCGCTTCCAAGCCAAGACCGAGGAGTGGNNNNNNNNNNNNNNNNNNNNNNNNNNNNNNNNNNNTCTGCTTTCCTGTTTCTAGTTCTCACCAACGGCTTCGGTGGTGATAATCACACAATCGACGACAATGATAACAATTCGTTGACCCGCCCTCAGCGACGGATGAGTAAANNNNNNNNNCCGCAATTCCTTCTGCTCTTATAGTTTTCTTATTTGGGTTGGTATTATGTTTCATTCGGCATCCTTCCATCATCAATGATCTTAAATTTGGTCCATCAAGAATCCAGCTGCTGAAAATTACATGGGAAGATTGGAAAACCGGGTTCTTNNNNNNNNNNNNNNNNNNNNNNNNNNNNNNNNNNNNNNNNNNNNNNNNNNNNNNNNNNNCTATCCACTGATTTGTTCCCAGATCACGAATTGTCCGCGACAAAAGTTTCTGTTAGTGTTGGGGTGATGAATTTGGTTGGTTGCTGGTTTGGGGCCATGCCTGTGTGCCATGGAGCAGGTGGGCTGGCAGGGCAGTACAGGTTTGGTGCCAGGAGTGGTGCGTCAGTGGTTTTTCTGGGGATAGGAAAGCTAGCGATTGGATTGGTGTTTGGAAACTCTTTTGTGAAGATTTTGAGTCAATTCCCAATTGGAATTCTTGGAGTGCTCTTGTTGTTTTCAGGNNNNATGGCATCTAAAGATATGAACACAAAAGAACAATCTTTTGTGATGTTGGTTTGTGCTGCTGTTTCGTTAACTGGGTCTAGCGCTGCATTGGGTTTTGGGTGTGGGATTTTGCTTCATCTGCTGCTAAAATTGAGAAGCATGGAGTGTTCTTGTTTTGGGTTTTCCAGGTGCAGCTCTAAAACTTCTGCTGATGAGGAATCTGCTCTCATTCCTTCAGATTAGAGAATTTTCTAAAGCATTCACCATGAAATGTCCTATTTTAGATGATTTAGAATCTTGCAAAATATTTGCTGAAATTGACAAAGCAATCTAATGATAAAGGTATGAGATGGCTGTGGACAAATAGGGGATCGATTTATGAGAGGAAGAAAAGTTATTTTCTATTGCAAAACACATTTTATTGAGAAAAATTGTTGTAAAATATGCATATTGAAAGTAATTAGAAAGAGAAATTCTTTTTTTGTTGATGGGACTCGTCCTTGGAGGAGTCTGTTTCTGCTGTCTGGGTTCACTCCATTCCAATTCAGTA

>Unigene4934_All

GCTTGCACAGAATCAACTTGCCTATCAGCAAATCCACCATCAACAGCAGCAGCTTCAGTCTTTTTGGGCAAATCAGTATCAAGAAGTTGACAAAGTTGCTGATTTCAAGAATCACAGCCTTCCCCTAGCAAGGATCAAGAAGATCATGAAGGCTGATGAGGATGTGAGGATGATATCAGCTGAGGCCCCAGTTGTATTTGCCAGAGCATGTGAAATGTTTATTTTAGAACTGACCTTGCGGTCTTGGAATCATACAGAGGAGAACAAAAGGAGGACATTACAAAAGAATGACATCGCAGCAGCCATCACTAGGACTGATATTTTTGATTTCTNNNNNNNNNNNNNNNNNNNNNNNNNNNNNNNNNNNNNNNTATCTAGGGGATCAATGCCTGTTGGGGGGCCTGCTGATGCACTCTCATATGGTTATATGCCACATCAGCACGCAGCTCAAGTTGGAACTCCTGGGATGATAATGGGTAAACCTGTGATGGACCCAGCTATCTATACCCAGCAGTCTCATGCCTTTATGACGCAACAGATGTGGCCACAGCAGGGATCAGAACAACAGCAAGCTCCCTCAGATCATTAATGACCAGATATGAAAGTTGAGGTTGTGTAAGTTCTGTATTTAACTTACCTATCACTACAGCATGAAATTTGTGTCCGACAGCCCAAAACTGTTTAAGATCATATGAGCATGTTGTAGAGAACTACTGTGATGTAATTGATAGCAGCAATACTTGT

>Unigene4949_All

GGCTTAGGTGAACGAAATACTTTGGCCTGTATAAAAATCATCATCTTGCCTCGTGAACGACTGTAACTTTACAGATACATATTCTCGCATTCACATCATCATTCTCACATCCAGGGCGGCAAATATGCTTCTTGCTACAATTTAGCTTTCTTTCGACGCCTCCAATACAGAGCAAACAGCTCTCTGTCGCCAGCTTCTCTTGCTCTATTGGTAATCCACCTACCCCCTTGGGCCTTAGCTTCCATATCCAATGGCTCATCCTCCGCAACCTGACCCCTTTCCTGACTCCCGCCACGAAGTCGATGCCGGTGCAGCCTTCGTCCTCGAATCAAAAGGCGAGTGGTGGCACGCGGGGTTCCATTTGACGACGGCGATAGTGGGGCCTACAATACTGACTTTGCCATACGCGTTCAGAGGTCTAGGGTGGGGCCTGGGCTTCTTATGCTTAACAGTGATGGGAGTCGTTACCTTCTACTCTTACTATCTCATGTCCAAGGTGCTCGAACACTGTGAGAAAGCCGGTCGGCGCCACATACGCTTCCGTGAGTTAGCCGCCGACGTTTTAGGGTCTGGTTGGATGTTTTATTTTGTCATATTTATTCAAACAGCCATCAACACTGGAGTTGGTATAGGAGCAATTTTGCTTGCAGGGGAATGCATTCAGATCGTGTACTCGNNNNNNNNNNNNNNNNCAATGGATCCCTAAAATTGTACGAGTTCATAGCAATGGTGACAGCAGTAATGATTGTTCTCTCTCAGCTTCCAACCTTCCACTCCCTCAGACACCTCAACATGGCTTCACTGCTTCTCAGTTTGGGCTACACTTTCCTTGTAGTTGGTGCTTGTATTCATGCAGGTCTCGCAAAAAATGCACCTCAAAGGGATTATTCGCTAGAATCTTCTGAGTCTGCAAGGGTCTTCAGTGCCTTCACTTCCATCTCCATAATAGCTGCTATTTTCGGGAATGGAATACTGCCTGAAATCCAAGCAACTTTGGCTCCCCCTGCTACTGGGAAGATGGTGAAGGGCCTTTTGATGTGTTATAGTGTTATTCTGGTTACG

>Unigene4960_All

AATAGCCAAATGCATAAAATATTACACCAACAACCTTCAGTCTCTTTTTAGCACAGATTAAACGGTTCCCTTTTTTACTCCTTTGCTGATTAATTCAAAATAAAAATAGATCGATTTAAAATAAAAATATCATCTGAAATGTAGTCGCTAGCCAGTCACCATTGAGCAAACGAACAAGGGCAGCTCTAATGCAGATGCGAAAAGATGGATTACCTCGTGGCCATGTAGAATCTAAAGTAAATCCTGTTTATTTACATGCAACAAAGGCAGACAAACAATTGCAATTTGATATAGTTTCCTCTAGGCACCAGGGCCTGTTACACCTGCTCGAGCTCTCAACATTTCTGTTCTCTTGCATCGTTCCATTACCTCCACAAGCCATTCTACACTTTCTTTAATACCCTTCCCGTCATATGCCGAAACTGCTTCAAACTTGTAAACCCTCTCATCCAACTTCTTCAGATCCAAATATCGAGCAATTTCTTCAGCTGATACAGCTTCAGAAAGATCCTGCTTGTTTGCTAATATCAAAAGAGGGGCTCCTTGCAAATCCTCATGCCTAAGAACTTTTTCTAATGCAGACTTTGAATCTTCAAAACGTGAAGGACAAGTAGCATCAATTACATATACAACAGCATGAGCCTCTTCATAATATTTCTCCCAAATTGAGCGAAGACCGTGCTGACCACCCAGGTCCCAAAATACAAGTTTTGTATTTGAAGCTTCAATGCGACCAATGTTAAGCCCAACAGTGGGAACAATTCGATCAGGAGGAAGGCCTTCTAAGTTTGAGTACAACGATTTCAATTTCTCCAATAAAGTTGTTTTCCCCGCCTTGTCAATTCCAAGAATGAGCACATGAAACTCTGTCTTACTGAATAAATACTTCNNNNNNNNNNNNNNNNNNNNNNNNNNNNNTTCTGATATTTAGAAATTTAATTATTACTTGCTCCTTCAAACCTTCAGGCGTAGGAGAGATGGAACTTCATAATTCGAACTTGATCTCCATGATGCGCGCTTCTTGAAAATCCTGAAATTTTGATGCTTGAGATGAGAGAAACGAAGACCGAGATCNNNNNNNNNNNNNNNNNNNNNNNNNNNNNNNNNNNNNNNCTCTTAATCACTAACAGAACACACATACACAGTCTCCGTCTCTACCGGCGCTCTTATTCACATAACCTGTTCGGTTTATGCAAGGCTTTCATCGTCCTTGTTTG

>Unigene4966_All

TAAATGTAGAAAACTGTGATGCTTTCACACGAGTCTATTTAGATCTAATCTACTTGTTTACATCATCTGAGATCTCTTTCAGGAAAATACTAGTCCAAGTATCACATGACTAGATTATAACATTTGCAGAAGCCGTTGGCAAGAGCAGAAAGGCGATATCTTTAACGACTAACCATACTTATAAAAATTTAGATNNNNNNNNNNNNNNNNNNNNNNNNNNNNNNNNNNNNNNNNNNNNCTATGTTTGGCGGTTGCCACAGCTGCCGCTTTCATTTCAAAAGCAATCTACTTCATTTTCTTCTTTGGCCTTCTACTGTTTTGATTGGTAACCTTCTTGTTGGATTCCTTTTTTGCCTTAGCTTGTTTATCATTGGTCACTCTGCTTGGTTTCTTCTTTCGATCTTTTTTATCATCATTGTCATTGTCATCATCAGCACCACCATTATCAATTTCATCATCATCAGCAGCAGCAGCACCTTTGTCATTTTCTTCATCATCATTGTCATCACTTGCCTTGGATGATTGTTTTGTTGGAGGCTTCTTGACATGACCTCTAGGCTTCAACTCTCTCTTTGATTTAGCCTTTCTAGCAGCTTCCTCTTCCTCGCTTCCTGGTTTGTTGGCATCATCTTTAACTTCATCATCATCATCATCATCATCATGGTCACTCTTTTTTCTCTTTGGAGTTTGTCTTTTCGATTTTACTGCTGCTTTCACAGCTTGCCCTCCGCTTAGCTTTTGCAACTCTGGTACATAGGCTGTTGTCCTACCACCAGCAGTGATAAAATCAATTTTCTTCCCAT

>Unigene4970_All

GTTGTAACATTCGGCTGAGATTTTGATTACGCTGAACTAGTAAATGCAAACAGTTTCCTACAATAAAAATGAAAGTGTCTTTGATTATGTAGTGAATTTACAGCAGGATAGAAAATAACTACACTGTCGGAAAAAAAAAAGGAAGAGCAACCCTAGAAGAGCAAAAATCTGACATTGCAGGCATTACATAATCAGACTGGTGCACTAGATCAATATTCCCATTTGTTCATTACCATACCCTCTGGAGCACTACCTTCTACCTTCTTTGAACCCACTTCTTTCCAGTCAGTTGAAAGTACCGTCCCGCTTGACTCCACAAAAGATTTGGTCATAGCCCTTCTCATGTTGTCATCTGCATTTTGGTAAATGTCCCTGAATAACTTGTTCAAAGCTGCATCACCATCTAGCTTCTCATCTCTTTCCTCGCTCTTCACTTGGGCTTCCAACTTATCCCAATCTTTTGCTCTTGATTTTGAAGATGGATATGAAGGCCTTCGAGATCCAACTGATGACACATTTATTTTCTGCGGGACTATAATTCCCTTGCAGTATTCAAGAGATGTCCAGTTAATAACTTCAGCTTTTGCAAGATGGATTTCAATCTTGGTTGACAATACTTGATATTTGCTCCTATCAGGTAATATTTTTCCAAACAATTGAGGTTGAAAATGATATGTATCTTCACCAGGGACATTGATGGTAACACTGAGAATTTGTTCACCAAAATCAACTGTGACATTTTCTGCTGTAATGCCCTTTGCAAAAATTGTTAAAACCACTTCCTCTGGCTTCTGGTAGTATTCATGTCTGTATTTTGGTTTTTCTGAAGAGATTGTTTCATTCTTACAGGAACCAACAGATGCAGGNNNNNNNNNNNNNNNNNNNNNNNTGGTGCCAAGGACTTCCTTTGATCATAAAACTCCTCTGCTATGTGCAGATCACATTCTTTGATCAACTTGGTAAATCTGGAATCATCTGGAGCCAAA

>Unigene4977_All

GGGAGAGAGGGAGAGAGAGAGAGAGTACAGAAAACAGAACCAGAAGAAAGAGTCCAACCTGAAGAACAAAAATGTCGAGCTTCCATATTTCTATGATCCTAGCTTTCATCTTCCTTGCCTCATCGGTTTTCGCAGACGACGTCGTTGTCCTCACTGAGGATAACTTCGAGAAGGAGGTCGGTCAAGATCGTGGTGCCCTTGTCGAGTTCTACGCTCCCTGGTGTGGGCATTGTAAAAAGCTTGCTCCGGAATATGAGAGGCTTGGCGCAAGTTTCAAGAAGGCAAAATCTGTTTTAATTGGAAAGGTGGATTGTGATACACATAAAAGCCTCTGCAGCAAATATGGTGTTTCTGGATACCCAACAATTAAATGGTTTCCTAAAGGGTCATTGGAACCCAAAAAGTATGAAGGTGCACGTGCTGCTGAAGCCCTTGCTGAGTTTGTGAATTCTGAAGGAGGAACCAGTGTGAAGATAGCTGCTGTTCCATCCAGTGTTGTGGTATTAACACCAGATAATTTTGATCAGATCGTCTTGGATGAAACAAAAGATGTCTTGGTTGAGTTCTATGCCCCGTGGTGTGGCCATTGCAAACAACTTGCTCCAACCTATGAGAAGGTTGCAACAGCATTTAAATTGGAGGAAGATGTAATTATTGCTAACCTTGATGCTGACAAATACAAAGATTTGGCGGAAAAGTATGGAGTAAGTGGCTATCCTACTCTTAAATTCTTTCCAAAGAGCAACAAAGGCGGTGAAGATTATGGTGGTGGGAGGGATTTAGATGACTTTGTAACACTCATCAACGATAAATGTGGGACTAGTCGTGATGGGAAAGGGAGACTTACTTCAAAAGCTGGTATAGTTGCTACTTTGGATAACTTGGTTAAAGAGTTTATAAGTGCTGGCGATGATGAGATGAAAGCAATTGTTGCCCGCATGGAAGAAGAAGTTGATAAGCTTAAGGGAACCACTGCAAGATATGGCAAGATCTACTTGAAAGCTGCCAATAACTGTGTGGAGAAAGGTGCAGATTACCCCAAGAAAGAAATTCAGCGACTTGAGNNNNNNNNNNNNNNNNNNNNNNNNNNNNNNNNNNNNNNNNNNNNNNNNNCAAGAAGAACATCTTGTCAACCTTTGCTTGATGAATGGTAGAAGCCACAAGTGAAGTCACATCCTCAT

>Unigene4978_All

TAAGCACCTATCAATTGAAACTTTTAACAATTATCATTTCAACGTCTTGATCAACTGAACTCTGGCATGGATTAGTTCCTTCCATATTTACAGTCAAGTGCCATAAACAAACACAATATAGACCTAAGAGACAATTCTCGAAGCTCAATAACAAATTAAAAATAAGTCACTATAAATCAAATGGTAAGCTTTGCCTCTCAATTAAATATTAAATTAAACACTATTTGAATTTACCTCAGGGCAGCGGGGAGTGAGGAGATATATCCTGTTGGAAATATTGATAGACGAGAATGATTAGTTCCCTTTGCACCGTACTCGTCCCTCATGTTGAAGACACCACCCGTTGCACTTGTTAATGCTTACATACCCACTCACTGGAGGCAGCAGATGAACTCCTCTCTCTTTCTCTCATCATCATCATCATAGATTCAATAAATATTCCAACCCTAAAAAGGGTATTATTGTCATAACTTTGAGTGTTGTCTCCACGGGCAACCACCTTCAAATGCCTGCTCCAACACATCTTCCATTCGCTTGGCTAGTAAAATCTCCAGATTGCTAAGCACAGCTGCTGGTACTTCCACTAAATCCTTCAAATTTCTTTCTGGTAATATTACTCTCCGGATACCATAACGGTGAGCTGCCAATATCTTATCCTTAATACCACCAACAGGAAGCACAAGGCCCCTCAAAGTCATCTCTCCAGTCATTGCTGTATCTGCTCTTACTTTTCTCTGACTGAAAAGTGAAACAAGTGCAGTTACTAGTGTCACACCAGCTGAGGGCCCATCCTTGGGTACAGCCCCAGCAGGGAAATGTATATGAATATCCCGGCCCTCAAGGAGATTAATTTCATGAGCAGCTGCTAACTTAAGATCTGTTGCCCTGGCTCGTACCCATGTCAGTGCTATTTGTGCTGATTCTTTAATAACATCACCAAGTTGCCCAGTGAGATGTAATTCACCCTTTCCCACCATAGCTGTTGCTTCCACAAACTGAACTTCTCCACCAAAGGTAGTCCAAACAAGACCAACTGATATACCAGGATTTGAAACACGCTCTGCAGCTTCTTTATCATCAAACCTTGGGGGCCCTAGTACTTTCTCCAGCATTGCCTCATCTACAACCAAAGGTGAAACCCTGAATGAATTTGATAGCTCATGATTGTTATCATTCATTGGAATGACTTCCATTTCCACTTCAGCTCCATCAGCAAGTCTATTTTCCAACAGTGGAGATGCCAGACGGTGCATATCTTTGCTCAATGGAACAGCTTGTTCTTGCTCTGCAACCCTTACAGCAGCAGCACGAGCCAAGGCAGCTAAGTTCCTCTCCAGATTCCGGACACCAGCCTCCCTAGTGTACCTCTGAATGACAAGTTTCACCATAGCCTCTGGAATTTGAAGGAATTCAGAACTCAATCCATGTTGATCCAAAACTCGTGGAATCAAATGTCGCATGGCTATTCTAAGCTTTTCTTCAGGCGTATATCCAGGGAGCTCAATGACTTCCATCCTATCTAAGAGTGGAGGAGGAATAGGCTGCGCCCTATTTGCAGTAGCCACAAAAATCNNNNNNNNNNNNNNNNNNGAACATTCAAATAGTGATCATTGAATGTCTTGTTCTGTTCAGGGTCTAGAACCTCCAGCAGAGCTGAAGCTGGATCCCCACGCACATCGGAACCTGTCTTGTCAATCTCATCCAGCAGCATGACTGGATTGCAAACACCTACTCTCTTTAAACCATCAATAAGACGCCCAGGCATGCTTCCAATGTATGTTCTCCTATGCCCTCTAATATCTGCCTCATCCTTAACACCACCAAGTGATATACGAACAAATTTTCTGCCAAGTGCAGCAGCAATAGATGATGCCAGAGATGTTTTCCCAACTCCTGGAGGACCAACAAAGCACAAGACTGGGCCCCTTGCATCTGGTTTAAGCTTGCGAACAGCTAGATATTCGATAATTCGTTGCTTGACC

>Unigene4999_All

GCAGCCCAATCTCCTCCAGGCCATGGCTGTGTTCTTCATATTCATATTCATGACCATAATCTTAAATATTCGTAATCCGACCCGTGTGTTTGCCCAAGCTGATGAGCAAAGCAGAAACTGCAGCTCCTCATTTCAGTGCGGAAACCTTTCGGGTGTTGCCTATCCTTTCTGGGGATCCAATCAGCCAAGTTACTGTGGCCACTTGGGGTTCCAGCNNNNNNNNNNNNNNNNNGCCAAGCGGCAGAGATCACAATCATGAATTTAACTTACCAAGTCCTTAAGATCAATAGCCAGTCACAGACTCTCACAGTTGCCAGAGCAGATTACATAGCAAATCTCTGTCCCAGTGTCCTTTCCAACACCACTTTGGACCCCAATCTTTTCACTTATGCTGCTGATATTCAGAATATAACTCTGTTCTATGGCTGCCCCAACACCAACATGTCATTTCCCACAACTCCAGGGTTTTCCTCTCCGTTTTCTTGCAACTTAAGCAGCGTCAATAGCCAAAATTTTTATTTGACAAGGCACGCAGATAGCTTTCCCACCGA

>Unigene5031_All

GCTGGTTAGAGGGGAAAAGAAACACTAATAAATAAACTAACTGTAAAAACACAATCTAAAGTCACACATGAAAGTTATCATTTCAAATAAATGTAGAAACACCGCAGCAAAAGTCAAACCAAAGATAGCACCAAAAATGCTCAGTTGCTCTGATATCTGTGTGTCCTTTAACCACTGTCTGAGAGTGCAGTTAACACTGAAACACACAAGGGCTGCAGATGCCCCCAGCCCTTAGTTCATGAAAAAACTTCCAGAATGTCAGTCATTTCCTTCCTCACTCAATATAGAACTTATTTTTGAGTGTTGGAAGTGATCGGCCAGTAGACTGTCTTATCTCATAGCCAATCTCAACCTCCATCAATCTGTGATTTTCCTTTGAACGGTTCATCATAAAAGACACATTCAAACAATCCCCTTCACTGATGCGATCAGGAGGGTGCAAAAGGAAAACCTGCTGGCCCCAATGTGTGCCATCATCTACGCTAGGTGCAGTTGTCAATTCAACCTCTTGCTGGGCCAGATCATCCTTTCTTCCTCGAAAATGAACATCAAACCATCCACCAAATCCGCAGAGTCTAGTGTTTTCCATAGTGATTGATGATGAAAATTTTGATCTGACCTCAAGGACATCATTAACAGTAGCCGTTAAACAATCAATTTCCTTGATGATGGCAGCTATCCCTATAACTTGATGTGGATGAAGGTTTTGCCACATTGCTGTCTGCAGATAATATTTTCTTTGCTCTTCATTGAAAGGCTTAGTTAACGCATTCATATCAACACCATAGTAAGTTTTGGTGTCTTCCACAAAATTATACCAATCATCCATTGCTCCATCATAATCACCCTTTTTTTTGATCTACCAATCCAGACCTAATAGGTGCCATCCACATGCGGGCATGGCTTGGATACATGACTCCACTTGGTTTGAGCCAGCGGTCACGAGCACNNNNNNNNNNNNNNNNNNNNGAAGTAACCCATCCATTCTGAGATGATTACATCAACTTTCTCTGGCAAAGTAACATCCTCC

>Unigene5038_All

AGAAGTGCCATGAGAAAGGATTTGGTGTCACAAAATGGGAAATGTGCAATCCAGCCCCATGAAGGACCTGCTTTGCATTTGAACCCGAAAATTCACTGTTTTGAAAAGAAGGCTGGAACACTTGCAGATTTGATCAATTAAAGTGCTTCACCCAACTCATACAGTACAATTCCAATTCATGTATTCACTCCAAGTACAAACCCATTGCTATAAGAAGGAAGATTGCTAGAAGTGCCATGAGTCTTCACAGGAGAGCGAAAAGGATTTGGTGTCACAGGATGCGTAATGTGTAATCCAGCTCCTCGAAGTACTTGCTTTGCATTTCCACATGAAATTTCTGACTGTTCTGTAAAGAATGCTGGAACACTTGCAGATTTTATTAATTCAAGTGCACCACCCCAACTCATATAGCTATCTAATCCACAGTTCAATGCAACTATAATATGGGGAGATGGCAGGTAGGTGGCTTCCTCTTGATAAATGCCTCTCACGAGATTCACCCTCACCCTGCTACTTATTCCTGAAGTTGTGCCCGATAAATTGGTTGGAACTTCAGTCCCCACCATTACAATATGTATATTGCCTGATCCATTGAGCAAATGGCTAATCTCTGCAAATGCTGGCAACCAATCCAACTCCCCTTCAGGTCCCAGGTAATGAAGAATCACCTCTTTCCCCTTGAGTAACAGATTCTTTGAACTGATGTTCAGGGTAGTCAAAATGTAATAAACTGTCAGTGGGTGAGAAAGAATGTCAGCAACAGGGCTTGACAATGGCAGTGACCGAAGATTATAGTACTCTGGCCAACCAGAAAGAAGGATTGGGCTCGATATCCCNNNNNNNNNNNNNNNNNNNNNNNNNNNNNNNNNNNNNNNNGATACTCATTGTCGTCCAGCCCACCCCATGAATCCCACAATCCACCTTTAACAGGAAGGAGTCC

>Unigene5053_All

TTCAGAACTCGAAAATACAAATCCCTAACCCATTTCTTCCTCCAATTTGTTGCTGCCAGAGACTCTCCTTCCCGTCGATCATTGACTGTCAAAGCTTTTACTGTGTTTCACTTTTGACTTCGAATCGGCCAGTGTTCACGGTCGAAGCCAAGAGTCAGAGTGTTCACTGTGTATTGTCGACTGCCAGAAGCTCCATTGTCGAAGCCAAGAACCAGATTTCACAGAAGCCAAAGCAGAAGGTCTATTGGTAGTCTGCTGGCATATGCAAGATGTACTCCAGAATAGCACAAATTCGCCTTGTGAGTTCACATCATGAGGTTTATGAGCCATGTGATGATTCATTTGCACTAGTTGATGCACTTCTAGCTGATCAAACTAACCTTTTGGAGCATAATCCTAGATTATGTGTGGAAGTGGGTTGTGGTAGTGGTTATGTTATTACCTCTCTTGCTCTTATGCTCAGGCAGCGTATTCCTGGGGTCTACTATATAGCCACTGACCTCAACCCTCATGCAATTAGTGTAACGCATGAGACACTGGAAGCACATGGTGTTCATGCAGAGTTGATACACACTGACATTGTGTCTGGACTGGAAAAGCGACTGGCGGGGATGGTAGATGTGATGGTTGTGAACCCACCTTATGTCCCAACACCTGATGATGAAGTGGGTTGTGACGGAATTACTTCTGCTTGGGCTGGAGGGGAGAATGGTCGGAGAGTTATCGACAGGATATTGCCCATTGCGGATGTTCTTTTGTCTGACAGAGGCTGGTTATACATGGTCACTCTTGCAGCAAATAATCCTTCACAGATATGTCGTCGAATGAGAAAGAAAGGTTATGCTTCTAGAATTGTCATCCAGAGATCAACAGAAGAAGAGAGTCTTCTTGTCATCAAGTTCTGGCGGGATCCTGACACTCAATTGGATGCAAAGAAAACCATGACAAACAAAACAATTCCTGAAAAGGTCGCGGAGTCACTGTTTTCGCAGTTTCGTCAATTGCCATTCTGGAGAAATGGTGGTAGCAACAGCTGATCAATGAGAAGTCTTCATAAAAGCATGTAGGCAAAGTTAAGTTAGAGCACAGCAGAAAGCACCAGCAGGTCATAGTTGTTTATGATCTGGAATCAGATTGGTTTCAGATGAATCGACATTCATATATTCATCGAAGCCCTATGCATGCTTCAACTAGACGTGGAGTTTCAGTGTAAAGGATGATCAACAATCTTGTGTAAGATGAGGCAATTTTTATGTNNNNNNNNNNNNNNNNNNNNNNNNTAATGATTGTGTACGTAATGCGAGTTCTCCATGAGAGAGGGTTTTTTCTCTCTCTCTCTCCCTCTCTCCCTCTCTCTCTCTCTC

>Unigene5077_All

AATTAGGGTTAAACCAGGAGCCCTTTGAAAATTAGCCTTTATTAAATATAGTTCTTACATCATTTAAGCACTTAAAGCTTCAAAGGGCTCCTGGTTTAACCCTAATTGGTGGTAATTCATGTAGTCAATACCATTTAACCTTACACAACACAAGACCTGCTCTGGTAACTCTTCAGGTTTGTTCCCCTGTATGGTGAGGCCAACATCCAGGACACAAATCTTGAGTCTGTCTAAGAACGCTTCAAAACCTTTCCTAGAGATGTAACTGAATCTATGCATGTCTATGTCAATCTCAAAGTAGTTTTCTCCTAAGTAAAATTCATGCTGAGGTCGTGAAAGAACAGGTTTTTCATTGTAAGCCTGCATAAGCTTCCTCTCCGCAGCACTTAAGTGAAGATCTTCTACATTTACAACTCGACCCAATATCTTCAACCTTTCACGAAAGGGTACTATTGTATCTACAGGGAACCCTTTGACTTTTTCAACTTCATCATCAATTAACCGTCGGATACTTTCTTGAAAGTGGATTGGAAGTTCCTTTGAATAACTGTCAGAAAGCTTAAAATACAAAACAAAATTCATTCCTTCTCCATCTGTTTCACTATGAAAAAGTGTGGCAGTATACAAGGGAATCTGAACATTTACAACAAGTATAGTTGGGAGTTTCCCAGAAGAGTTAATAACAGGAAGCTCCACAAACTGGGCAATATGATCAATTTTTCGTGGAGATAGGAATACATCAACCCCAAATGGATAATATGCAGCATACTTAGAAGCAAAGTCCTTCTTTCTGTCCCTGAAATAATTCTGTCCCCGAACCTTGAAACTGCACGGCTCTATATGTGACCAACAATTAAGCATTTTCTTTTCTATTGGACAAAACGGTACTTGGGAACCAGCTATTGGTCTCTGCAGAACTGGCTTTGAAGAAAATAGAGTGTTATTAAGGTGTCCTTCCTTCCATTTGAATGAGAGTTTTAAGGTAGCCTTTTTCCTTGCACTTGGTGGACTAGAGCTCAGTGATCTTCTCTTTTCAACTGGTGGAACAGTTGAAGCAAGACAAGGCAAGCAGTTGCCTGGAAGAATTCCACAGTTATCTAATAACCCTTCCCCTTTACCAGCATTTTCATCCACAGAGGACGCAATTTCATCAAGGAACACAGGTTGTGAGGGTCCTTCAGATTTTGATAGGGAATCTGCATAATCTGAGCTGACTAGTTGACCATTTGAATGTTTATCAGCCTCTCTCACAGACTTCCAANNNNNNNNNNNNNNNNNNNNNNNNNNNNNNNNNNNNNNNNNCACTTGAAGAGGTATGTTGTACATTTAAAGTACAGTCTACATGATTTATGTCTCTGCTAACTTCCAATCCATTTAAGGATAACAAATCATCTGAAACACTTTCAAAATCTTCATCACAATCAGACTCAAACATTGCAAATGAGTCAAACCATGCCTCCTCAATACTTCCTGCATGAAAAGTGGGGTTAGAGAAGGACGAGCGCTGATCAGGTGGAGCAGCAGCAGCAGATGCGGGCCTATCAAACTCATCCAGTGATCCATCAGATAATCTAGACGACACTCTTTTCCTGATCCCTTTCCTCTTCTTGCGTGTCTTCTTCTTAGAAGACCTCAATCTACCACCTACACATCCTTCCGGGGTTGATACACAAGCCCCCATACCTCTCCCTTCCCCAACCCCACCTCTCTCTTTCTGTTTTCGTCTCAGAAGATCAGAAAGTCACCATCTTCAACAAAAATAAAAGCAGAGAGATTAACAACTCTAGTAGTCTTTACGAA

>Unigene5083_All

CATTACTCCATAATTTGCCCTATAAACAAATTGCCTATCAATGCTCGTCTCGAGCAGAGCTTCAAAGCTGAGCCCGAGGCCATTGTTTTAGAAGGGAGATCCAAATGGTGCAGTTCATAACAAATGAAAAGAAAATGGGAAATTAGTGAGAAGATTGAAAACAAGGCCAAAGCAAAGGAAGTTTTCCTAAATCTTGTCATGCAGCGTGACAACCAAGTCATGTGCATCGTCAAGAAGCTTCCATACATCCAATTGTCCAGCCATATTGTTGCAGTCTTTCATGATATCATCAACACTGCTATACTTCTCGATGATCAGTTTCCTCCTCTGCAATACACAGGCCGCTATTGCATAAAGCAGCAGATCATCAGTAGGAGGGGCTCTCAACCTCATCCTTCCCCAGGCAGACTTGGCAATCCCTGCACATATTGCTGCTTGGTCAGCCCACATCACCTCCCATAGGCACAGTGTCTGCTCAAGGTTTTAACTCCCTCCTGAAAAGAACCACCACCATTCTGTACACAAAAAAGCAGTCCTCTGCTTGGAGATTCTCTAGGTGCTTGTAAAGATGGATATCCTTGCACTTAATTATCTTGGCAACAATACCAAGTTGCCTCCGGATTCCCACTTCATCAAGCCGGAAATTATGCCTGGCCTTCTTCATGAAGCCCACAAAGCACCAAAAGGCTTCAAAATCCTCCTCCATCACTGCAATAATTGGAGAAAGCAAATCACTCATTCCNNNNNNNNNNNNNNNNNNNNNNNNNNNNNNNNNNTCATATAGTGCATAGGCCTCAAGGATAGCCACCAGGCGAGTAGCATGAAAGATCCTGCAAGGCTC

>Unigene5096_All

TAAAAATAATCAGCATTTACAGACTCGAACAGAAACTCAACAACAAAAGATGCTTCAAAGAAAGATTTCATACGGTCCTAAAGCCTCAGGAAAGATGGATCACACACTAAAATTACAAAAGAATATTAAAGGAACAAGAGATCAATAAACCAAATCATATGAGCATATTCACATCCTAATTTACAAAGGGGAATGGACCTTCCTTAGGAACTTCCATGAGATCTGACAGATGAAATGGATTGAGCAGAGACAGAGGCAGTTGGGCTGTTAAGTTCCCGATCTCCGTCAATAGCTTTTGGTTTTCCACTTAATCTTTCCACTGCTTCTTGACAAATGTCATTAAACCAGTCATCTTCTGGCAGCAAACTGTCTGGATCCATTCCTGTAGCAGAAAATGCTGCCACAGCCTTAGAAATGATCTCATTGACAACTCGATGCAAATTCCTTGACAAGTATCGTCCTTGGGAAGCAAAGCACATCACAAACTTCATATCCAAATAAAACTGTTGAAGACCAAGAGGACCCAAAGGCCTTGGGCCTTCTTCAATATCATCCCAAAAGCTTTGATCTTCTGAAAGCCATAATATAACTGTTTCTGTAAGCCTCATCAATAGCAATGTAGCAAATCTTTCCCTTCCTACAAACATATCTGCTGCTATGCTAGCCATTCGGTTCAGTTTTAGAAAAAGTTCCTGGAATATTGGAGATGGAAACCATTCTACCTCATCTGCACTTCCATCCATGTTTATGTACATTTCTGCACTAAGGTGACTATCACCATCTTCTGAAAAAATGAGATCAAGTGCATGTTGGCGACAAAAAGTATCTTTTAATCTATCAACTGAACTCACAAGCCGTTTCCTCCATTCTCGCTGCTCAGGATGGCGATTTTGCCTATCCGAAGTCCTCCTACGGGGATCATCCTTGTAATTCGCTTGGTGCAGAGGAGAGAGCTTCATGGCAGCCCGTGGGAGCAGTTCATCAGCCAATAATGATGCATTGGCTAGCAATGCAATCTGCTGGGCTTCAGTTTCAGCCATCCGTACAATTTTATTACCTGAACCCTCAAAGTTTGCTTCTTCTTCTATTGAACCTGGTAATGCTTTTATCAGCATGTTCACATATGAATTAAATACTTGAAACAAGCCTTCCAGTGATTGACTTCCCAGCTGCATGCTTAAAAGTGGTCCAACATCCTCAAAGAAGTCCTGGACCATCAAATTGAACCGATGGGCACTGCTTGTAAGTTTATGCTGAAATGCAGCTGTGTTACCAAGAGATGCAACAGAAGACCTACCAGACTGTTTTGGAGGGTAAGTAAGTATCCAATCATCAGCAGCAGCCAAAGCAGCAGTGCTCTCTTCAATTCGTTTAAGATTTGCATCTAATGCTTGCTCAACACTAGGTCTAAATAGTTTTAAGAGCACAGGACAAAGTGCCAAGCCACGAGCTTCCAACAAAGAACAATGACCTAAGGCTATTTGAACACACTCTGCAGCAGCTCTTAAACCTCCAGCAGCGGCTGAGGATGTTAATGCATGTCTTTTAACTAGAACAGCAAAGGCCTCTGTTTGCTTTGTAGCCCACATTACAAGCTCAGAAGTATAAGCTGGTTCCTTGCCAAAAATAGACAAAGAATCANNNNNNAGCAGAGAACACTAACTGTGAAAGTGCAGCAGTATATGCCCCTCCATACGAGGTGCTCGATGGGCGAAGGCTTTGCATGTTGCACTGATATCTCTGGAAATGAGCATTGAGTAGTAAATTATGAGCACGAGGACCATCCCCCAGCTTTTTAAGAGCTGATATAGCTGCACGAAGTTCACTTCCACGGGTAGAAGGTTGACAAGCAGCTTCAGCAAGCTGATCAGCTAACTTTTGCCGGCGCTCCATAAGAGCCATCTGCAATGACCCAAGTATATCAGGACTCAATGATTTTGTTTCTTTTGCTTCAGATGCTACATGTTCTCCTTCATCAAGGGCTGCTATGGCTTCATCCACTCTCCTTTCGGCTAACAAAACATCAAGGAGATCAGGGAATTCAACTGACCACTTCTCCAAGTCAGAAGGTTCTCGATGTTCAGTATTTAACAAGCCATTTATTGCAGAGGCTTCCGAAGCTTTGGTAGATATTGAATCAATATGAACACCCTCCGCTAAACTGTGAATTAAAGTAGCCTGAGTAGATAGCAGATTTCTGATGGATGAAAGCTCCCCCTCTAAATCTGATATCTCTTTTGAAGTGCGAATGAAAGCTGCATAATTTGCATAGACACTCTTACGCATTTCTTCAGCTGAAGCTTTCTTCAAATCCAAAAGATATGAGCATAATTGTCTTATTTCCTTGTCATTGAGAGAGCATTTAGACTGAACATAAGCATTGGCATCAAACTTATCGGACTTGAATACATTCAGACCTTCTTCAAGCTTCGTCCCATTCTCCTTCACCGGCGTTCCTCTGGAGCGACCAGGTTTCACTGTAGCCATAGAATTTTTCTTCTAACAACAAAATCAGATAAACTCCTTTGAATTAGTGATTTCAACTTTAACAGAGATCTAATCTCAAGCAGGTCAAGGAGAGTTGGCGAACAGAACAATGATTTCTTTCGGAAAGAAGAGAGAGGGGTGCTTTTTCCCTTTCTNNNNNNNNNNNNNNNNNNNNNNNNNNNNNNNNNNNNNNNNNNNNNNNNNNACTCGTCACTCGTGGGACAGCAACTCAAGTCTTGCAAATGAACTTCACTGGAAGTGGCTGATTCTCCGAGGGAGATGAAGGGTATCGATGGGAACAAAGAACAAAAGAGCGAGTTGAAAGATCGTCGTGAGTTGAGAGAAGACAAACTTGAAAACAGCGCCAGTGCGGTTAGATCGGCGGTCGCCTTCTTGATCTTTAATGAGAGTCCGTTCGAGAGGGTGGGGGAACT

>Unigene5098_All

TCATGCGCTGGAGTGATCTTGACAGCACCAGTCCCAAAATTGGGATCGACGAGAACAGCATCACAAATTATAGGAAGTTTTCTTCCATTAAATGGATGAATTGCAAATTTGCCATGTAGATGACTGTACCTTTGGTCATCAGGATGTATGGCAATAGCAGTATCACCAAGCATTGTTTCCAATCTAGTTGTGGCAACAACAATTTCACCCAAATCTCCCTCCAAAGGATAAGCAAATAAGGTTAAAAGACCAAATTCCACTGGCTTATCATAACCAGGAACCTTCAACAGTGTCTTCTCTTTGATGTCAGTATAATCTACCTCAATATCAGATATAGCTGTTCGTAAGGTGCAATCCCAATTCACTAATCGGATATCCCTATAGATGAGACCTTCCTTGTATAGCCTAACAAATTCCTCGATCACAGCCTTTGATCTTTTATCATCCATTGTAAAACACTCACGAGACCAATCAAGAGAGGCACCCAAACGACGCAGCTGTCTCAGTATGGCACCCCCATAATCCTCCTTCCACTTCCAAACTTCAGAAACAAATTTCTCACGACCAATATCATGTCTGGTTAAGTGGCGTTCACGCATTAATTTCTTCTCTACAACCACCTGTGTTGCTATTCCAGCATGGTCCACACCAGGTACCCACAAGGTATTATATCCAGNNNNNNNNNNNNNNNNNNNNATCCTCTATAGCAGCTGCAAGGGCATGGCCTATATGTAGGGCCCCAGTCACATTTGGTGGTGGAAGAACTATGGTAAATGATGGTTTGGAGCTTTTTGCATCAGCTGTAAAATAACCCGACTTTTCCCACCACGTATACCATGATTTCTCCACAGCAGTTGGATTATATTGTTTTGCCATTTGTGCAGAAAGCCTTTTCTTATCACCAGAAGGAGTCTCTGGGTCAACATAATCTTCAGGATTCTCTTCACCTCCAGCATCTCGCCTAGCATTCTTTTTAGCAGAACTCTTTTTTGAAGCATTATTGGATTCTTGTTGTGCCTTAAATTTAGCTGCTTTTTCAGCTGCCTTAAGTTTCTTCAATTCCTTTTCCCTAGCCTTTTCTTCTTTCTTCTTCTTGCG

>Unigene5101_All

TTTTGCACAAGCTTCTTACTTTGTGGAAAGCAACTTTATTTTTCACACATAAGGTGTCTCTAACACCAGAAGTCAAATTAATTGGTGTTTCCACAAAGTAAGAAGCTTGTGCAAAATAATATACTATAAGCATTTTGTATGGCATCATAAGTTTCTCAGTATAATAATACTTCAGTAGATTAGATGCCTTTACATTCAACAATACTGAGATCCCTATATAATGCACAATTACTTAGAGAAACAATTCAAAGAATTTTTCACTTTCAAGGAATGTAATACATAGGATTTGGAAGCTTGAATGAGCGATTTGACGTAGGAGAACCCAGTAAATCGCTCCATTGGTCACCAGAGTTTCCTAGAATTCGATATCCGTCTTTCACCATTTCACTTCTTTTCTCTGACTTGTATATTGTCGCTAGTTTCCCATGATCATCAGAAGCCCTCAAAATAAGCTTGTCCCAATCCTGAAAACCTGCATTGATCAAGTTCTCCTCAGTGACACTCCTTTGCTTCTCACTGCGTCCTGTTAGCAAGAAAACCTTGAAGCCTAAACCCAGAACCTCCTTATAGAGTTTTAAACTGGGCTCTATAGCCGGTGCCACAGCCTTCCCAACCCACTTATCAAACTCCACTGGATCAAAAATCTCCAAGCCATAACCATGGTCAGCATAGTAAGGGAGATTGGAGAGCAAAGTTTCGTCAACATCGAACACCCAAGCATCCTGGCCATCCCCACTCATTTGGACACTCTTGGCATAAACTCCAGCCTCATTCGACACCCTCTCGAGATCGAGTCGATAACCCCTTCCCATCACGTAATCTCTGACATACTCTGCACACTCCTGCGGAATCGTTTTCCACGGATTCAAATTGTTAGCCTCCACCGCAAATCTCCAGCTGGTACACTGCAATTGGAGCTCATTGTCCAATTGCTTGAATTGGGTAGTTTCGATTATGTTCTCCGGGTATTCAAGGATCAAAGGCCGAGGTAGAAGGTGAGAATTTAACGGTTCATTAGAGAAAACGAGGGAAAACAAGGAAAAGAAAAGGAACAACCCTGGCAAAAGCATTATGAGGATTTTGATTTGGGAATTGGGAGAATGTATCAAATGATGGGAATTGAGGAAATGGGATTTTGACTCCGATTATTATAGAAGGGAATCTGTAAAAAATTGAATCTTAAGAATCCTCTCTGGTTTCTTTTTTTTTTTTTTCTGGGGAAGGAGAAAAGAGCAAAAGGTGAGTGGATGAATAGATTAGATAAAAAGAAAATCCTTTTCTTGGTGAAGGATAAGAAAAGAGGAANNNNNNNNNNNNNNNNNNNNNNNNNNNNNNNNNNNNNNNNNNACCTTCCTTTCCTGTTTTCTGCAGCTACAGAGAAATGGAGAGGAAGGAGCGAAGTAGCGTGTTG

>Unigene5136_All

GACGACGAGAAGAAGGAAAATGAATAATAATAAATAAAGACAACGGCAGCAGCAGCAAGAGCAAGAACAGCAGGCATCCCGTCGCCGTAAAAATACAGATTCCGCCGCCGTTACAGCTCCACCGACACCTCCACTCTTTACCCTTTTTACTGATTTTCTTTTGGATTGTTCTCTCGATCAATCAATCTGAAGGACTCCGCTTCTCTCCGCTTCCTTGCGCCCTCCTCGGGGGATGCCGATGCTGAGGCCTCTCAACGTCCGCTCCCTCCAGCCCCTTCACAACTGCCGCCAATATCAGCCGAACCACCACTATCGCCTCAGTCGCAATCATTATCACCACTCTCGGACATTATGATATCGAATAGGGATCCTGATGCTTTTTTCAGCGGCGGGGGGGNNNNNNNNNNNNNNNNNCTTTCTGAGAGCCAAACTGCTAAATTCAGCTATGGATATTCAAGTTTTAAGGGTAAAAGGGCTTCAATGGAGGATTTCTATGAGACCAGGATATCTGAAGTCAATGGTCAAATGGTTGCCTTCTTTGGTGTTTTTGATGGTCATGGAGGTGCCAGAACTGCTGAATATCTTAAAAAAAACCTTNNNNNNNNNNNNNNNNNNNNNNNNNNNNNNNNNNNNNNNNNNNGACAGCTATTGTTGAAGCATTTAGACAGACAGATGCTGATTACCTCAATGAAGAAAAAGGCCAGCAAAAAGATGCTGGGTCAACTGCATCAACTGCTATGCTCCTAGGGGATCGGCTTCTTGTTGCAAATGTTGGTGATTCTAGAGTGGTAGCCAGTAGAGCTGGTTCAGCTATTCCATTGTCTATTGATCACAAACCGGATAGATCTGATGAGCGCCAAAGGATTGAAGATGCTGGAGGTTTCGTCATATGGGCAGGAACATGGCGGGTTGGTGGTGTTCTTGCTGTTTCTCGTGCATTTGGTGACAAATTACTCAAGCCATATGTTGTTGCTGAGCCAGAAATTCAGGAAGAAGAAATTGATGGTGTGGATTTTATAATTATTGCAAGTGATGGGCTTTGGAATGTCTTATCAAATAAGGATGCAGTTGACCTAGTGCAGGACATAACAGATGCAGAAGCAGCATCAAGAAAACTAATACAAGAAGCTTATGCAAGGGGTAGCTCTGATAACATTACCTGCATTGTGGTTCGATTTGACAATTCATGAGCGGACAGATCAAGGTGATAGTCATCCTAGCTCAAAATGGAAGAAGCATCAAGTAGTGCTTTATTTAGCCCTACCTTTGGATGGACTGTGAGTAGGTGATGGGTGTTACAATGCAGACTAACAATTGAATTTCCAAGTTGTTGATGTAGATCCATGTGTTTATTATGGATAGCTTCATGGTACCTTGATATAAAAGATTGTGTTTTGTTGCCACCATTCAGATCTTGACTGTTCCTTTAGAAACAAAAACAAGGTAAATTACAGATGACACAATTTTTATGAATTGCAGTTTAATTTTAGCCCAAAGAATTTTTCACCTTGTT

>Unigene5163_All

CAAGCGGCACAATAAGTTAAACGGTTTGGAATTTTATGAAACCTTGATGTTACCTGATGCACAAGTGAATGCATGACACATGTAGTCCTATCCATGCTAACGTTAGTAAAATCAATCTCCCTTATAAAGAAGAGGAGGATACAATCCCGTCCAGTTTTCTAAGCTCTCCTACAAAAGAGGAAGTTTGCCTCTTCTGGACAGGAGGTGTACTTAAGTCCCTCATAATGTTATCATTTTTTTTTTTAATCAAATGACCAAAGCGACTGTACATCTGGGCTTGGGAATAAACCCCCAATATTGGGCCTAGTGACAGGTTGCTTCCCAACACCTTCTCCTGAACTTTGCACAGTCCAATCCTTCTTGGACCAACAATTAGCTGGAGAAAGCTTAAAAGAATGGTTGGTTGCAGTGTCTGTAGGACTCCAGTAAATTATTTCATTCCGAGCATTCTCCTCCAGAGGTTTCAGAGAGAAATGACTATCCCCCTCTAATGGAGGGAAAAAAGCATTCTGNNNNNNNNNNNNNNNNNNNNGCTTGAACAAGTGACTGGACTAAATGCCCCACCATTCTGGCCATTGCGAAGAAAAACCTTCTGTGGAGAATGAGTACCAGAAAGGACTTGGTCATCTTTTGAAAAAAGAGAAGCCNNNNNNNNNNNCTGAGATGAAGACGGCAAGACATCTTCCTTGTTTGATCTGCATCGATCTGGAGGTAACAGCCAGCTTCCTGGACCACCTACCAAACCTAAACCATC

>Unigene5191_All

AAGGAGCAAAGCATAGCTTCACATGGTTAACAAATAGCCAAAACACAGAGTCTATACACAAGAAAACCACAGAGGATATGACAATGCCACCACAAGATAGATCAAAGCCACTGCACAACTTCTCATTGCCTTGCTTCAAATGGGGTCACCAAAGAGTCCTTAGATGNNNNNNNNNNNNNNNNNNNNNNATCGATCTTCTTCTTCGATTCCCAATGGATTTCAATCCAAACCCACCTGCCTTGACACCCCCGAGAATCACAAACCAATCCCAATTCAAGAATATCCCATTTCTCCTGATCTTAGATTCAATGAAGGTGCTAAGAG

>Unigene5230_All

TCCGTGAAGTTAGTCTCTTTTATTCAAAAATGACCATCTCCCATTGTTTGCATTTGCGCATGGTTGACAAAGATTTCATACAAACACTGACATTGCTAACTTATATACACTATCCGATACTCTGATGGAGATAATTTTTACCACAGAAACACGAGGAGACTTTCATATTAGGTCCATCTTCAGCACCCACCACTCGCAGGTTCTATTGAACTCAGAAGCCCATTACCCCTCAGCTGCATGCAGTGCTCTTCAGCATCAGNNNNNNNNNNNNNNNNNNNNNNNNNNNNNNNNNNNNNNNNNNNNNNNNNNTGCATTATGTTAACTGCATTATCAAGAGTCATTCCAGGGATAACCTTCATCAGTACTTGGACAACATATTCCTTTTTATTATAGTTGTCATTGTGAAGTATCACACGATAAGGTGGAGCTGTTTTCCTTGATTTCCTCAAATCAAACTCAGATTCACGGCCAGGAGTGGTTTTCTCTATAGTTGGTCTCTCCAATAACCCACCTCCTTTACCTGATCTTGCAGCTGATAAAGCCCTGAGAATCCCTCGGTTGATCCATGGTCC

>Unigene5268_All

AAAAAAAAGCACTTGTCTTTAATGTGGCTTTACATGCATCACAGGGGGGTATTGTTATATATATACACTTTCANGAAGTACAACGATCAACCACGTCCAGATTATGGAATCGAGGTTATGTACAGGTTTGCTGGATTTGACCCATTTGAGAGATCAACTTATTTTGGACCCTTCTTTGATTTGGGGCAGTTCGAAAGATTTAGGCGGATTTTTCACCATTCAACCTATCGAGTTTTGCTTGGGCACAAAGAAAGAAAAATCCTAAGCAGTTTGTTGGTGAAAGAGAATAGATTCAAGCAGCGAGTTTGGATACGAGGAAATCGCCCAGAGGAGGAAGAAATATTTCAATTTACCATGGTCCAGAAAGTTGGTGGTTCATGGGATGGTTATTGGCTGACAGAGTCTCTTTTAAATGATGGAGATGCTTTTGCTGGTGGTCTAGCCTACTAACTATGCACACAGTTGGGNNNNNNNNNNNNNNNNNNNNNNNNNNNNNNNNNNNNNNNGTTTGCGTGCTCAGCCTCAGAATTGTGTACATATTGTAATAAACTGATATCATTATGAGCCCTC

>Unigene5307_All

CAGGAAGACACCAATGGCTGCAATCTTGACCACTTGAAGCATAGTGATTTAAAGGGTTTTTCCCGTATTTGGATGGATGACCATCTATCCGAAATTCTGATAAACTAGTAATGTTCAAAAAAGCAACAGGAGTTTTCATTTGCTTGATGATATCTTCTGTGATTGTTTTCTTCTCAGTAACGTGTACACCAAGAATTTTATTTAAGGGTTTTGTAGCTTCCTCGCAATGCCCACCTGAATTCCATTGGCCTCCACTGAAGTGCGAAGGTGATGAACTTCGAAAGAAAACTTGGGTCTTTCTGGAATTGANNNNNNNNNNNNNNNNNNNNNNNNNNNNNNCTTCTGAAAGCTGAGGAAACATCAAGCTTGGGATAAACTTGGTTCCCCTCCTGGTAGTAGTTGATCCCAGCTTTTGTTTTGAAATGAGACCACCAATGGGCAGTGTTGAAGATTAGAATATCAGCTCCTCTCCATCTTGATGATCCATGATCAATGGCGTCAATCCGCAAGGTCTGCACCCGCTTCTTCCCTACTCTTGCTTTGCTCTCATGAACCAAGAAATGTGATACATAGTATTCAACGGTACATTTGTAATCCACAAATTTGAAGCAATAATTCCCTTTCTCCTTGGTGATTCTCCGTCCATGGGTCTCATACACCATCTTGGGATCTCTAACAGCTCTCATTAACATACACAGCATGGACTCCCATTGATTCCTATTAATTGAATCTCCAACAAAAACCAGCCTTTTCCCTCTGATTAGATCCAGCATTCTGGTTGCATTGAACCTACTTGGGATGTCACAATCTTGAGGTTGCCATCGCCACTTCATGTAATCTTTATCCAGTCTCCCATTTCCCACACAATCGAACCCTTCATCTATGAAAGGACATGAACTACTCGTGTAAAGAGGATAGCTTTCATCATAGACCCACCGCCCTTTTGTAACGTCACATCCTCTTGTGCTTCTATCTATGATCCTGCCTTTACTTCTAGCTTCAATATTATCAATTTTAGTTAATCTAACTTTCTTCTCTTCANNNNNNNNNNNNNNNNNNNNNNNNNNNNNNNNNNNNNGTTGCTCTCTCTTTCTTCTCTTCAATTCCCTCTGCCCTCGGGGCTTCAATTTTTTCAATTGAATCTACTTTAGTCTCCTCTGCAGCCTTCTCACTCCTGGAAACTTC

>Unigene5320_All

AATGAGGTCAAGAAATATGACAAGACTAATAATTCATGGAGTGTTGTCAAGCAATTGCCAGTGAGGGCTGACTCTTCTCATGGTTGGGGATTAGCATTTAAAGCATGTGGTAGCAGCTTATTAGTGATTGGAGGGCATAGAGGTCCTGAAGGTGAAGTTATTGTGCTGCATTCTTGGGATCCACAGGATAGAAGTATGGATGGGCCAGAATGGAATGTTCTTGCTGTCAAGGAGCGAGCAGGTGCTTTTGTATACAATTGTGCGGTAATGGGATGCTAATGCTTTGGCACATACTTGTCTTTGGTGCAAACTTTGACTTGTGTGCGTAATCAAATGCTTGATTGGGGAAGCTAGTGGGGAAAGGGAAAGGGGAAGGGCATGGCATGGCATGACACAAATCCAAGATATATTTGCNNNNNNNNNNNNNNNNNNNNNNAGATTACAAGGCTGGTAGTTTTCTCTTTTCTTTTTTAATTGTCTAATTAGCCACTATGTTGTAGAGTTCTGAAGACCCATGAGAGTTTTAACATAATATTGTCTAAAGAATT

>Unigene5349_All

TACAAATTGGAAGAAACATTTACTGCCCAAAAGCAGTTGTCAAAATCAACAGAGTATGAGTTTGGAAATACAAAGTGGTTTATGTTCCAGTATTCAATGTCTTTGGTGAATATTGAACCAATTGATTTTCTAGACCTTGCAGGCATTTGTTAATAGATATCATTAACATGTATCTGCCATCTAGAGAATTTTCGAAATGTCTACCCAATATATTGCAAGAGAAGCATCTCCTAAAGCTCCAATTCCAAAGCAGTTAACTTCGACCTCGGCCAACTTTCTGATAAAGCAAAAGGCTCAAAAGTTCCTCAAGTCGCAGGAATTGAAACGCAAGGAGACCTTCAAAGACGCCCAAACACGCCCCCGCCAAAACGTCGATAAGGAAATGCCTACCAAGCAAAACCCTAGACAAGGCCGTCATTGCCGCCCACACACGAACGGCCACCACCACAATCCCTACCAGTTTGGTGTCGTCCCCNNNNNNNNNNNNNNNNNNNCCGCCACTCGCCCTCAACTCCGCCAGAGCCGCTTCAATTGTGTTCGCAGATAGCGAAACCAAGGCGGCGACGAGGAAAACGCGAGAAGCGTGCCCGCTGGGGAAGGAGTAATTGTCAGCGGAAACGGCGGCGGACATGTGAGGATTGTAGGGAGGGCGAGGACGGCGGAAAATGATCTTAATTAGGCCGACGAAGGCGAGGTCGAGGAGGAGACCGAAGAGGAGAGGGGAAAGGAAAGGAAGGAGAGAGGGGGAGAGAGGGGAAAGGCAAAGGGCGAGTGTAATGGGAAAGGAAAAGCGGAAGTCGGCCGATAATTCAAGGACGAGGTGAAGCGAAGCGGGCAGGTATGAGTTGAATAGAGAATGAAGAAAGTGAGAGAACTTGGCGTCAAAGGTCAAGAGGTGGCGGAGAGGTGAAGACCGCGTAGCCATCACCCGTTGTGGTGGTGGCGGTTAACAGTACGGATGTGATGGTTAAGCGGGTGTTGCGGTGGTTAAT

>Unigene5362_All

CGCAGAAGCGTTTTGGGACTAACCGCCGCGCAGATCGTTTTGTGAGCGCGGAGGGCCGGTCTCTCGATCAATAGCCAGGACGGGAGTGCGGGGGCGGTGGCCAAGCCGAGCGAGAACAGTGTGAGTAACAAACGTTGACGCCGGGTTGTGCTAACTTTGGTAATGGCTGGCCGATCAGCCGCTTAATCCGCAACATATCCTGGCAAGGGCTTCCGTCATCAATACCAATCTGTCCGGCGCAAAGCACTACGTTTCCATTCCGAACATGATTGACCCGGACGGAGGAACGCAAAGCAGACTCAGCTCATTCGCACGGTGGCGAGCAAAAATGGTCCGCTCGAGTGCTTTTATATTGGATTGCAGATCTTCGATCTGCATCATGATCAGCTTGCGCACGACNNNNNNNNNNNNNNNNNNNNNNNNNNNNNNNNNNNNNNNNNNNNNCGAGTTGGTGAAGTTCCGGAATCCGCCAGACGCCTTTTGGTGCGATCAGCCCGAATTCG

>Unigene5372_All

CTTAACAAGATCACTTCATCAGTCCAACAGCTTCTAAATAAGGTGGACTCAAATCCAAAACCCAATTATGATACTAATGGAGTTGCCCTCGCTGGCAGAAAAAGTAGCCAAAAAGACAAATTTCCCGCATGGTTTAAGCGTGAGGACCGTAAATTGTTAGTGATAAATGGAGTGACTGCTGATGCAATAGTAGCAGCAGATGGAACTGGGAATTTTACTAAGATAATGGATGCGGTATCAGCAGCACCTGATTATAGCATGCGTCGCTACGTCATATTTATTAAGAAGGGTTTTTATAATGAATATGTGGAGATCAAGAAGAAGAAATGGAATCTAATGATGGTTGGTGATGGCATGGGTGTCACAGTAATTTCTGGCAACAGGAGTGTCATTGATGGTTGGACCACCTTTCGCTCAGCAACCTTTGCTGTTAGTGGGAGAGGATTTATAGCTCGAGACATAACTTTTGAGAACACTGCAGGTGCTNNNNCAAGCAGTGGCTCTCCGATCCGATTCTGATCTATCAGTCTTCTTTCGATGTGAAATTAAGGGATATCAAGATACACTCTACACTCATACAATGAGACAATTCTACAGGGAATGTCGTATTAGTGGGACTGTAGACTTCATTTTTGGTGATGCAACTGCTGTCTTTCAAAACTGCCAGATTCTAGCCAAGAAAGGCCTATCAAACCAGAAGAATACTATAACAGCTCAAGGACGCAAGGACCCAAATGAACCAACTGGATTCTCCATCCAATTTTGCAATATCTCAGCAGATATTGATCTTTTGCCTTACACCAACTCCACATACACATATCTTGGTAGACCCTGGAAAAATTTTTCAAGAACTGTTGTTATGCAATCCTACATGAGTGATGTTATAAGGCCACAAGGATGGCTGGAGTGGAGTGGTGATCAGTATTTGGACACTTTGCATTACGGTGAGTACTTGAATTATGGGCCAGGAGCTGGGCTAGCTAACAGGGTCAAATGGCCTGGGTATCATATTTTCAATACTTCCAATCAGGCCAATAATTTCACAGTTGCCCAATTCATTGAAGGAAACTTGTGGTTGCCATCAANNNNNNNNNNNNNNNNNNNNNNNNNNNNNNNNNNNNNNNATGAATGTTGGATTCTTGGTTTCAACTATAGTTGTTCAGTTTTTTCCTTTTTTCTTCCGTCCCT

>Unigene5376_All

CTCTTACGAGCTGCTGCAGCTTCATCTTCTTCTTCTCTATTTTTACTGCTAAGAACTGAAGAGTAGAATTACACTACTTCCTATCTCTATACATGGAGTTGGAGTCTACTTGTAATCTTGTATAATTCGATTCCTCAGCATCGCCCTCCACCACTAAAATCGAATTCAAGATGACCCTCCACTCCATTGTAATCCAGAAACTACTCAGCACTAATGCTCATTTGGGCCGCCGCGTGGTCGCCGATCACTTCAAGATCTATACGTACGGAATCCGGAACGAGAAGGCCATAATCGATTCCGATAAAACCCTAATCTGTCTTCGGAATGCTGCCAACTTCATTGCCCATATGGCGCGTGACAAGAAAGCCCGTTTTCTATTCGTCAATACCAATCCTCTCTTCGATGAGATCGTAGAACAAATGACCAAGAAGATTGGGCTGTATAGCCCNNNNNNNNNNNNNNNNNNNNNNNNNNNNNNNNNNNNNNNNNNNAGTTTTAGCCCTAAAAAATTTAGGTCGAGAAATAAAAAGGTTTGTTTTGGACCAATACAACCGCCAGACTGTGTAGTGGTGTTGGATTCAGAGAGGAAGAGTTCAGTGATTTTGGAGGCTGATAGGCTTCAGGTTCCAATTGTGGCTCTGGTAGATTCCAATATGCCATGGGAGTACTATAAGAAGATTGCGTATCCTATTCCTGCCAATGATTCAGTTCAATTTGTGTACTTGTTTTGCAATCTGATTACCAAGACTATCTTGCTCGAGCAGAAGAAGTTGAAAGCATTGAAGGGAGATGCTGCTAAAGAAGAGCCAGTTGTTTCTAAGAAAGAATCCAGGGAGAACACGAAACAGATTGAGCAAAGTGAGAGCGAGAGCAAAGTCAGCCCTTCAATTGATGAACTGCTAGTTGTTCCTTATCAGAATTTGGCTCCTCC

>Unigene5407_All

CGAATAAATTTCCAAACAGAAAAGAAACTGAAAAGGTGGAGTTCCCTGGTACTGTGGAAGAGAAAGAGTCTGGGAAGCATATTCACAAAGTGACAGAGGAAGCTGAGAAGATCGAGGAGAAACCAAGTAACTCACATGTGAATAGCAAATCTGTTGCCATGGAGGAGGAAGAAGTCATTCTTTTCAAGCCTCTCACAAGATACAATTCTGCCCCACTTTATAGTGCTATATCAGCTAATGATCAAACAACTTCGGAAGATAGAGCGGACCTGATAGTGCCTGCTGATGAGTGCTTGCGACGTGCAACTTCACTGTTAATAGCTCAAAATCAGGCCCATGGTGATCCTTCAGCTTTCCATTCTGATCTCACAAATTTCAGGCGCAAGCCATTGCAACAGCAGGAGCCTCTTGTAAAGGATGCTGTAGCACTCCCATTTTCAGAAGCCTCTATCTCTTCCTCTGGGCCTCCCTCTTTCAGCACCTCCATTGCTGCTGGACCACCCTCACTCAATGCTTGGGTCCTTAATAGAGGAACTTTGAAAAATGATAGAGCAGAAGGGAAGAGAGAGATGAATAAAATGCCGCCTTTTGAGGAAATTGCATCTGCATCCCTCAATGATCTCTCCATCAGTGATTCTGAGAATTCTGTCACTAGTTCAGGGCATGAAGCTGAGACCATGCGTAACTCTTCTCCTGCTTATTCAGCTCTATTGCCTTCTGCTCCATTCTTGCCTGATGATGCTAGTTGGTTTAATGGAATTCAATCTACCTTCTATGACTACGATGGCTCAGGAAACATCAACAGAACAAACAATTTCTTTGATGCATCACAAGTGACCAACTATTCAAATTGGCCAGGTTCTCATCAGCCGCTTGATTATGGTCTTGGTATTCCTGGTCTAATGGATGGATATCCACCAGTGCGCCGAATGACTTCTTCTGAATGGCTTCGTCAATACAGGGAAAATCACAATCTTGAGCGAACCACGAATCATGCACGGNNNNNNNNNNNNNNNAACACTGGAAACTTTTATGGTCATGATACGTCTAGGTCTGGTCTTTTTGATCAATTTGGAGCCCCTTTGGCTGCTATTAATCCATTGATTTATGAGGAGAGGCCACCATTACACTCAGGTTTTCCTTCTGTTTATGGTACTGTTGACTACAGAAGGGAGAAGCTCTATCCTGGTTATCAAAGACCAAGGCCTTATGGGTGTGGTGCGGTGAATGAGCCTGAGCCTCTTCTGCAGTATCTAAAGGAGAAAGAATGGTTGCTCCAGCAAGATCCCACATTGAGAGGCCCTACGTATATGGGAAGTTGAAAATTTGATCTAACCAAATATGAGCATTTTACATAGCTGCATATCTTTCATTGGATCCGATCTTCATCCGCCG

>Unigene5493_All

CCAGTAAAATGAAAAAGGCCTTTGGTTTGAAATCGCCAGGCTCCGGTTCCAAGAAGAGCCCCGGTTCGGGTCCGGGCTCGGGCCAGGGGAAGTCCCGGCGGTCTTTGACTGTTGGAGAGTTGATGAGGAGCCAAATGCGGGTGTTGGAGACTACAGATTCGAGGATTAGGAAAGCTCTTGTGAGGATTGCTGCTGGTCAGGTTGGAAGGCGAATAGAGTCGGTTGTTCTCCCGCTAGAGCTATTACAGCAGCTTAAGCTTTCAGATTTTACTGATCAACTAGAATATGAAACATGGCAGAAGAGAACTGTGAAAGTTCTTGAGGCTGGACTTCTTATGCATCCACGCGTGCCACTTGAAAAATCAAATCCTACTTTACAGCGGCTACGGCAAATTATTAATGGAGCTTTGGATAGGCCTATAGAAACTGGAAAGNNNNNNNNNNNNNNNNNNNNNNNNNNNNNNNNNNNNNNNNNNNNNNNNNGATCTGATGGCTCTTTTTCTGAGATATGCCACTGGGCTGATGGTATTCCATTGAACCTCCG

>Unigene5525_All

AAATGCAGGGAAAAAATTATGGAAAGGACATCCATTTCCATCGACATTCAACTAGCAGGAAGCCGTATATACAAATAAAGAACATTGAGCATAAACCCTCTAATAATGGGAATACTATCCAATTGCAGTCACACAAGAGAAAAACCTCTGGAAACAATAAAATACAACAGCTGATTTCCAAAAATTATCATCACCAATTGTCCTTCTCATTATTTCCTGCTCCTCATGATTGTGATAACAGCCGAAGCCCAGCTTCCAAATCTTCCAAGGTGATGGTACGTAGTTCATCAANNNNNNNTCAAAATCCAGCCTGAAGTCCAAATTCTCTCTAGCATTAACCAGCATTGTATCTACTAAACCTCCATTCATCTCCCTACGTTGCTTTTCTGTTGTTTCTTTCTCTATCACTGTTGCAATGGCATGAATATTGCATTTAGAGTGTAAATTAAATCCATATAACAAACCTTCCTCTTGTTGATTATTCATCTTAACATGGGCGATCTTGGCTAAATCTTCTGAAGTGAAGTCATCAAAATGGAAAAACTTTGTAACCCTTCTACAAAAGCCCTCATTAGAAGCTATCACACGCTTCATTGGTTCACTGTACCCAGCAAATATGACAACAACTTTTCCACTGTCCATAACAGACATGATCTCTTCCAAGGCTTCTAATCCATAGTCTTTATCATCTGCTTTCTGCATGGGTATTAGCCGATATGCTTCGTCCACAAAAAGAATTCCTCCCTCTGCTTCTTTAATCTTCCTCCTAGTCTTTGGTCCAGTATGACCAACAAATTCACCAACCAAATCCGTACGTTGAACTTCTGTTACCCTATCAGTGGGTAGGATTCCCACAGTAATCTTCCAAGTATTCGAGCTACCATAGTCTTACCTGTTCCAGGATTTCCCAAGAAAGCCATATGAGGAGGTCTTCTCACACCCACTTTTAGCCCAAGGGCCCTGCGCCTCTCATCCAAAAGCATCCCCTTTGCCCATTTTCTTAATTGAACCTTAAGCTTATTTAACCCCACTACGTTAGATAATTCATTTTCAAGTTCCTCCATCTTAGCTTTTGTTTCACCACATGCGTCAAGTGCTCTTTTCTTTCTCTGCTCTTCCATATGCCATTGTAATAGTTCACGCAACTTTGCACTTCCTGGACCTCGCGAGACATGATTAATAGGAGCCATGCCCTCATTGTCCTTAGCACTGCAATCAGCATTATACTCAAGCAATGTCTTGACAGTTGAGCAGTCTNNNNNNNNNNNNNNNNNNNNNNNNNNNNNNNNNNNNNNNNNNNNTTAGCTTTGGCTTCAACAAAAGCACCATGGGCAAGAAGTAATCGTGCAGCATCATTGCAACCATTCTTTGCTGCCATGTGTAACGGAGTTTCTCCATACATGTTCTTGGCTTCCAATTCAACTTTCTCCACCCCTCCCCAGTCAAGTAGGAATTTCACTATCTCGGCCCTGTTGTTACCAGCAGAAACATGCAGTGGTGTCTGTGCCATAACAGGATTTCTTTCATTGAGAAGAGCAGGATTGGCCCGCAGCAGCTTCTGAAAGCCAAGAAGATCTCCAGACTGGGCGCAGGCATGAATGGTGAGGGGTCTGGAAGATCTAAAGCGTTGATCC

>Unigene5534_All

TCAAAATTGACTCAACATATGATGGTAGATTTGATAGTGAATATTTCCAACAAACTGAAGAGTCAGACCGGTTTGGGCAGGAATATGAAATGCGTAGAATGAAACAGGATCAAGGTTTAGAAGTCATAGAAGAAGGATTGGACACTCTGAAAAACATGGCCCATGACATGAATGAGGAGTTGGACAGGCAAGCGCCACTGATGGATGAAATAGATGAAAAGATGGACAGGGCAACCTCAGACCTGAAAAGTACTAATGTGAGACTTAAGGATACTGTCAACCAGCTNNNNNNNNNNNTTGCATTGACATCATCCTCCTCTGTATAATTTTGGGCATCGCTGCTTATCTATACAATGTGTTGAAGTGAAGTATTCCGGCTNNNNNNNNNNNNNNNNNNNNNNNNNNNNNNGGTATGGACAGACTTCTGCGGCTATAGAATTTTATTTGCAAATTACAACAGTTTCTGCGCTTGGTGTAAAATTACGAGTCTTTATATACATTTTGTAATCTAATTGTTCTAAGCATCTGCAAATCCCGTAGGAACCATTCTCATGTTAATAGGGTNNNNNNNNCCACTTCGTCAATTATGGCTCCATCCTGTTTTCTTGAAGTTGGGATAAATAATTCTCGATTCTCATGCTCAACAAGAACTCAATTCCAG

>Unigene5541_All

AGTATAGTTAGTCCAATCTCTTTTCTAACTAGTTCAATTGTTTCACTCTTCACTGGATGTCCTGATGCATCAGTCACATAGAAAACATTCACAGCCTGGGAACCCCTGGTTGTAACCTCTGCTCGAGTCACTGACAGACCATTTTCCCTAAATATGCGGGTCACCTCAGACAAAAGGCCAACCCTGTCTTCACCACAAAGTTCTAGTCTTATACCCTCAGGAGTTCTCCTTCTAATAGCTGCCTCCAAGCAGTGGATAACCCTTTGCCTCTCTGCTTCAGAACTAATAGGGGACCCATCCATATGCCTGATATAATATTCCTGATAAGCCTCTGGCCCTTCAGCTATGACAGTTGCATGATATACAACATATTGCATATCTGTTAATGTACACACAGTATCAAAAAGTAGCTTAGGGCGGTCAGGGCACCTCAAGTTCACAGCCGTGTATCCCTTATCTGCACAATTTTCTACTGTTACAAGGGGTTTGCTCCGTTCACTTGTTGATCCACAATCCGCATCATCCATATCATAGTCACGATCAGCATACATCATCTGGTGCAACCTCCTTTCCTTATGAGTGGAACCCACAGACACAGCTGTATTTGCACTTCGTTTATCCCTATCTCCCTTCAGAACATACAGGAGGAGCTGTTTGATCTTAGTGAGTCTATCATGAACATCAATAGGTAATCCAGTAGCTTCATCAGTTATGTAAACAACTGATGCCATTCGTGAATTGTGAGTCCAGACTTCTGCAGCCACCACGTTACATTTGAGGTCAGTAAGAACAGCAAAAACTTCTGAAAGCAAGCCTGGACGGTCTCTTCCAGTCAATTCANNNNNNNNNNNNNNNNNNNNNNNNNNNNNNNNNNNNNNNGACCGGAAGCTGCGTGCTCTTGGTCCCAACGACTGTTGGATTCGTTCAGCCACATCATCCTTAGAGAGCTTATTCCCATGTTGATCTGTGACATGAAACACATCCATGAACC

>Unigene5581_All

CCCAGCTTTGTTACACAGTTCGCAGAGAAACTGGCAACTATTGTCTAGCTTACTTCCTAGAATCAAGCAATGGAGGTAGAGGATTGGGAAACTTCTTCAGCTGAGTTGAGCGATACCGAGGCCAATTTGAAAGATACAAATGATGAAGACTTGTACTATACTCCTGGTTCTTTACCCAAGTTGCAATTTAGGAGCGATATTTCAAAGGCTCGATGGGATGATGAGATGGGTATGGCTGGNNNNNNNNNNNNNNNNNNNNNNNNNNNNNNNNNNNNNNNNNNTTGTTCGCAATGGCAAGATCTACTGCTCAATTGAGGAGATTTTGTTTTTGGCTGAATTGGGGGCATTGCTTCTCTTGGATGATAAAGATATACATTTTTCTCTAAAAGATATATATGGGAAAACTGTAGATGAAAAGAATGGGTGCTGCTGGGAGCTTTTTGAGGTCTATAGGCACCTCAAGTCTCTTGGTTACATT

>Unigene5607_All

TTTTTAATTGAACATATTCAGTTTTGACATTCTAAAAAAAAAAATATTCAGTTTTGGTACAAGACAATTAATTTTATACGGTGGAACGACTACATGTACAAATGATATACTACAAGACTAAATTCACTATATCAAAAAGAAACACAAAAAAAAAATGAAGGCAAAAAATGGACAGCTCAAGTGCTTAGATTCGCATCATCAATCTCAATGGCCGCAAACTACAACTCAATTGAATAAAAAACCGTAACTACCAGTGGAAACAAGACACTTATGAACAGAATGTTGTTCCACTGGTCAAATACATATGTTACACCCACACGCTGTGAACTGTAAGTTCAGCAATTGATTTGGTACCTGCACTGGAGTAATCCATTCCGTCGTGTACATTCTATTGATGTCCTGGTAGTATTGNNNNNNNNNNNNNNNNNTTCAACTAATCAAACCGCAGCGGCCTAAACCTATGACTTTATAAGGGGCCTCAGCCCTTCCCCTTCCCAACCAAGTTTGAGCATTTGATCTACCACTTTCAAGCTCAAAGCTGGATCAGTGAAGATTATCAACTGCTTATCTGCAGTTGACACCAACAGATTTGTGTTATCATTGTTTAAAGCCAGAGCTGTTATATCTTGGCCTTCTTCAAGC

>Unigene5609_All

TTTCAAAGTCCGAGATTCGCTACGAGGGCATCCTCTATCATCTTAGCGTTCAAGATTCCATTATTGGCCTCAAGAATGTAAAGTCTTATGGAACAGAAGGTCGAAAGAAAGATGGCCCCCAAATTTCCTCAAGCGATAAGGTTTATGAATTCATTCTTTTCCGTGGAAGTGATATTAAGGATTTGCAAGTTAAGACTGCACCACCTGCACAAACTGAAGAACCAATTCCTGATGACCCTGCTATCATTCAGTCACACTATGCAGGGGTTCCTTTAAGTTCTTCAGCTTCAGTTGGTGGTAAAACTTTGGCAGAATCTGCTCTGGTGCAAGATATGCCTGCTTTGACAAGCAGAGGCTATCCGACTGCACTGACTTCGTTGCAGTCTGTAAACCATGTTGGTTCAAAAAATCTTTCCCAGGCTGCTGAAGATTCTGGTCAATCATTTTCTATGTCAATGAATTGGCAAGGGTATAATGGAACATCAATCAATAGGTCTAATACTTCCCAGCACTCTATTCCTTTTCAACCCCCATCCATGGTTTCATCTTCCTTGTCAGCACAGAATCAGATGCCTGCTGCAGCATTGACTGATTCTCCAATAATGGGATTAACAAATGCATCAGAATTTGCTACTCCTGTACCATCTGTTGCCTCTAATAATTCAACTTTTAGTCCTGTACAATATTCTCTTTCTCATGATATGCCATCCTTCTCATCTGTAAAAACGTCCCTACCATCGCTTACTTCATATACTAATATTAATAGGCAAACGGTATCTTCAGTTCCATACTCTAGTGAAGATACAGGTCTCACTAATGTTCAAAATGTTAGTAAAGCTGTTTCTGACCCTAAAACAGTGTACACTGCCCAATCTATGCCTTACCCTATATCTTCTTATGAGGCTTCTACTTCAAGCCCCTTGTTAACACCAGCTCCATGTCTCTTAAGTCCTGATCAATTGGCACAGTCTAGATCACATGTGCTTTCTTCCATAGTGTACCCTGATCAGAAGAATACGGATCCTCTGACTCCTTTATCATCTACGTCACTGTCCTTTGTTCCTTCTCCAGCTTCTCAAGAACCATTATTGCCATTGCCNNNNNNNNNNNNNNNNTCACTGAAGAATTTGATTTTGAAGCCATGAATGAGAAGTTTAAAAAGGATGAAGTTTGGGGTTATCTTGGAAAGGCAAATCGGGGAGATAACACAGTGGGCACAGAGGACAACCAAGGCTTCGAGGACAAAGAGGTTCCTCGCCTGGTGCCTCATTTTGATGCTAAGCCTGCATACAAGAAAGATGAATTCTTTGACACAATTTCTTGTAATACACTCAGCCAGGGAAGGAATGGTCAGAACCGATTCTCTAACCGAATGACGCTAGATAATGAGACTTTTGGTTGGCGCAATCAGAGACCTAATCCTGGCTATGGTGGATGGGGTGCCGGACGTGGCTATTACTATCATGGCAGACATAATGGAGGAAGGGGATATGGTTATGGTGGGAGGGGGAATGGGAACTTGCCTTTCTGATGCAGCCCTTTCAAACCACCACCACACTCCCGCCCTCCCTTCTCAAGAGATCCATTTGTTTTTGCATATCATGG

>Unigene5631_All

GTGATGTTAAAACAGCTGCCAGAGGAGGAGTTTGGCCCTGGTATCGGTATCAGAGAGTACTCTTTTCTGGACAATCCTTCATTGCCCAAACAGGTGAAAGATTCATGGCTTGATGTCGAGCTTTGTCAAGAAGGAACCCAAGATTGTTCTTCATCAAACAATACAAGTCCATCAGGGGTGCTCAGATTTCCAAATCACAGCAATGAATATATGTTCAAGNNNNNNNNNNNNNNTTCAAAGATATTAAAGTCATTCAGTTCTCTTCAATGCAAGACGCATTTCTAGGTTTCACTGACAAGAAAAGAGAAGAAAAATTTAGGAACCGTGTGAAAAGGTATGTTGGTATATGGTGTTGCGTGGAGAATCACACGCCTGGACACATATATTATGACCTGTACTGGGATGAGAAACCTGATTGGAAAGCAATGCCACCTGAGACTCCAGAGCAGGACCACCCACCTTGGTAAGGACTAACCTCGAGCATATATTTATTGAAATAGAAGAGGTGCNNNNNNNNNNNGAAGAATGCATGAAGCTAGTTTAGAAAGAAGAGGTAGATGGAAGTTAAAAGAGAATCTGCGAAGATGATGATTTAGTTCCTAACATAGATTAAACCAAG

>Unigene5634_All

GCCGGCCGAGGCAGGAGGAGCAGGTGATTGTTGTTTCTCGCCGTCCGATGGCTTGAGGTAAAATGGATGGGTGGGAGAGAGATCTTGGAAAGATTTTCTGAAAGCGGAAAAGATTTGAGGGTCGAATTCTGAACACTTGGAATTTGCTTCTTCATCGGCTAATTGAAGCCACAGAGGTTTCCGATTAGTCTGCGGCTTATTTGATGAAAATGTGACACCATTAATTCTTGGGCTATCAAGAACTGGACTTGGATTGCGAGAAACATCGAAAGAGAAGCTGCGAAACCGATGCGAACCGGAGGAGGTAGATGTACAGACCCATCATCTTCTTCAACCCCTGCCATAAGCTCCCAAGCATTGATCGTTTCAGGCTCTCCCGGCGGCGTTCTTACCGGTGTTTTANNNNNNNNNNNNNNNNNNNNNNNNNNNNNNNNNNNNNNNNNNNNNNNNNCTTCAATTAAACCCACCGAAAATTCTTTGGTCTTCAATTCCTCATCATCTGAAATCCCATTTTGCCCATCACACACACTCAAATTCTCCTGATCATTGCCTCTCGCTGCCCAAATATCAATAGCATCG

>Unigene5635_All

TTGACCGGTGTTATGCACTCGCTGATGCTAGTCCCATATGACGTCGTTTGGATCGTGGTTGAGGCTGGTGGAGTCAGTAATGAAACCGCTTCGATTATCGCCAAATCGGGAGTTAAGACTATTCATGTCGGATTCAACCAGAGAATGCCGAATTCTTGGGAGCGACGGCATAAATTGGAGGCTAAAATGCGGCTTCGTGCTTTGAGATTTGTGAGAGAGCAGAAGCTAGATGGGATAGTGATGTTTGCTGATGATAGTAATATGCATAGTATGGACCTATTTGATGAGATCCAGAATGTTAAGTGGTTTGGTNNNNNNNNNNNNNNNNNNNNNNNNNNNNNNNGCCGATGAGTCATCATTGGTTACAGTTAAGGAGGAGGTTGAGAAGAGGTCGTCAATGCCCG

>Unigene5643_All

TGGAGAGATTGGCAAGTAACTTGATAAGCGAAGAGATTTTGGGGAGGTTGGATTTGGAGACGCTTTGCACGGCGGCTTGCGTGAGCGGAGCCCTGCGCTTCTCCGTCGACTACCAAGTCCTTCCCTTTCTTTCTTCCCTCGATTTCCCACCATCTCTCAATCCGGATTCAAACGCTCTTTTTAATATCCTCAGTCGCTGCACCAGAGGAGGAACCCTTAAACTTAGCACGCTGACCCTCAATTGTCGGCGCCTCCAAGATTCTTGCCTCACCCTTTTCTTAGGTGAACAACTCCAGCAACTCAGTTTANNNNNNNNNNNNNNNNNNNNNNNNNNNNNNNNNNNNNNNNNCCATAGGAGAAAAATGCCCCTTTCTCAGGGTGCTAATGCTAGAATTTGCAGATCAAGGCTCAC

>Unigene5666_All

CAAAACGAGTAACAGGAAGTAAAGCCTGCTGTAAACCAGAGAAATAGGCCTGCTGTAAACCAGAGAAATAGGCATAGACATCAAAGAATTGGAACGGAATTATGAACTCAACCCTCTTGAAATAAGGGTATACAACTTCAGTTTAAGTTGCAGTAATTAGAGATTGTGAGGACTCGGACAGTAATGGCATGGGTTTTTGACACCTCTAAATTTTTTAATTTTTACATCCAAAACCACCAANNNNNNNNNNNNNNNNNNNNNNNNNNNNNNNNNNNNNNNNNNGAACCTTAGGAACAGAAAAAAGATGGTCCAGACAGAACCATAATGCACTTCTAATGCCCAACCTACTACCTTCAAAACAATGGGCCTGATGCTCATGGCTTGAGAGCAAGAGCCAATCCAAACTTGGCACTCTTCTCAATAGCCTTGCTATCCACCTCTCCAGA

>Unigene5670_All

ATTAGCTGTGCAGAACAAGAAAACTTAAAAAGAATATCAGTGGTGAATCATAAGGAGGAGGATGAAGAAAGTGTGTTCAGTTTTAGTTCTCTTCCTATCATTGATGATAAGTGGGTGTTCATGTTTGAACCGTAGCCAATTCCCTTCTTCTTTTTTCTTTGGGACTGCTACTTCTTCTTATCAGATTGAAGGAGCATACTTGGAAGCCAACAAAGGCCTCAGCAATTGGGATGTATTCACCCACTTATCTCCTGGAGAAATTAAGGATGGAAGCAATGGGAATACAGCTGATGATCACTATCATCAATTTTTGGATGACATAGAGTTGATGCATTCTCTTGGAGTAAATTCGTACAGATTCTCGATTTCATGGGTCAGAATCCTTCCAAAGGGTAGATTTGGAGAGATCAATTCGGAGGGTATTGCATTTTACAATAAGCTTATTAATGCTCTCTTGCTTAAAGGAATAGAACCATTTGTTACATTGCACCATTTTGATGTTCCTCAAGAACTAGAAGATCGATATGGCGCTTGGCTAAGTCCTCAAATGCAGGATGATTTCGGGTACTTCGCAGATATCTGCTTCGAGGCATTTGGAGACAGAGTGAAACATTGGATTACACTCAATGAAGCTAATATGGTGGCCCAATATGGGTACTACAGTGGAATATGGCCACCAAACCGATGCTCTTACCCTGTAGGCAAGTGCAAAGCCGGAGACTCTGAATTAGAACCTTACATCGCTGCTCATAATATGATACTAGCTCATGCCACAGCTACTGAAATTTACAGAAAAAAGTATCAGGAAAAACAAGGAGGGAAGATTGGCATTGTGTTACATATCTACTGGTATGAGCCACTAAGAGACATTCCAGTTGATCGTGTGGCTGCTCAACGAGCTCTAGGTTTCATTGCTGCTTGGTTTATGGATCCCATTATGTATGGAGAGTACCCACCAGAGATGCGACAAATTGTAGGTCTAAGGCTACCAGCATTTTCGATGGAGGACAAGAGGAAACTGGCAAACAAATTGGATTTCATTGGAATCAACCATTATAGCACTCTCTATGCAAAGGATTGTCTGCTTTCACCATGCAATTATCATGATGATTTACTCAAAGACACCTTTACTTATGGAACTGGAACAAAAGATGGAGTTCTTATAGGAGAGCCGACAGCAATGCCTACGTTCTATGTTGTTCCAAATAGCATGGAGAAGACAATCACGTATTTCAAAGATAGATACAACAACACGCCCATGTTCATCACAGAAAACGGATATGCACAACCTAGCGGTAGAAACATTGAAGATATGCTCAATGACACGAACAGGGAGGAATACATGGAGGGTTACCTCACTTCTCTAGTTTCTGCAATGAGGAATGGAGCAGATGTGAGGGGCTACTTCCATTGGTCTCTGATAGATAACTTTGAATGGACATATGGATATACCATAAGCTTTGGGTTGGTTCATGTAGACCGCACAACAATGCAGAGAACACCAAAAAGATCAGCCAAATGGTTCCAGCATTTCCTGAAAAATGAAGCTCTTCATCATCCCCAAGAATGGCAACAGAACATTGTGCATTTGTAATCTAGAACAGTGTTCACATGTATAAATATTTATATTTATTTGAAGTTGTTTACACTGTAGCATTTGCAACTGCAAAATGTAATATTATTATTCTTTATTCAAAAATAGTTTTCGGATTGCAAATGCTACAGTGTAAACAACTTAAAATAAATATAAATATTTCTACATGTGAACACTGTTCTAGATTACAATGGGCTGTTGCCATTCTTGGGGATGATGAAGAGCTTCATTTTTCAGGAAATGCTGGAACCATTTGGCTGATCTTTTTGGTGTTCTCTGCATTGTTGTGCGGTCTACATGAACCAACCCAAAGCTTATGGTATATCCATATGTCCATTCAAAGTTATCTATCAGAGACCAATGGAAGTAGCCCCTCACATCTGCTCCATTCCTCATTGCAGAAACTAGAGAAGTGAGGTAACCCTCCATGTATTCCACCCTGTTCGTGTCATTGAGCATATCTTCAATGTTTCTACCGCTAGGTTGTGCATATCCGTTTTCTGTGATGAACATGGGCGTGTTGTTGTATCTATCTTTGAAATACGTGATTGTCTTCTCCATGCTATTTGGAACAACATAGAACGTAGGCATTGCTGTCGGCTCTCCTATAAGAACTCCATCTTTTGTTCCAGTTCCATAAGTAAAGGTGTCTTTGAGTAAATCATCATGATAATTGCATGGTGAAAGCAGACAATCCTTTGCATAGAGAGTGCTATAATGGTTGATTCCAATGAAATCCAATTTGTTTGCCAGTTTCCTCTTGTCCTCCATCGAAAATGCTGGTAGCCTTAGACCTACAATTTGTCGCATCTCTGGTGGGTACTCTCCATACATAATGGGATCCATAAACCAAGNNNNNNNNNNNNNNNNNNNNNNNNNNNNNNNNNNNNNNNNNNNNNNTCTTAGTGGCTCATACCAGTAGATATGTAACACAATGCCAATCTTCCCTCCTTGTTTTTCCTGATACTTTTTTCTGTAAATTTCAGTAGCTGTGGCATGAGCTAGTATCATATTATGAGCAGCGATGTAAGGTTCTAATTCAGAGTCTCCGGCTTTGCACTTGCCTACAGGGTAAGAGCATCGGTTTGGAGGCCATATTCCACTGTAGTACCCATATTGGGCCACCATATTAGCTTCATTGAGTGTAATCCAATGTTTCACTCTGTCTCCAAATGCCTCGAAGCAGATATCTGCGAAGTACCCGAAATCATCCTGCATTTGAGGAC

>Unigene5672_All

AAACCATCAAACACATCATTTCTCTTTTCTTCTCCTCTTTATTTCTTGCTTACTATTTTGAGGTCGATAGCTACGATTGCTTTGGAGAGAATTAAGTCAACGCTTCAGTAGAATGGAGAGCACAATGAAGGATTTAAGGGGCGGTGCCACCGTCCTCGATATGGATCCCAAGTCCACTGTCGGTGGTGGCGTTGAGGATGTTTACGGCGAGGATTGCGCCACCGAGGATCAGCTCGTCACTCCTTGGACTGTCTCAGTTGCCAGTGGATACTCGTTGTTAAGGGATCCGCGTCATAACAAAGGGCTTGCCTTTACTGAGAAAGAGAGGGATGCCCACTACCTNNNNNNNNNNNNNNNNNNNNNNNNNNNNNNNNNACTTCAGGAGAAGAAGTTGATGCACTCTATAAGAAAATATCAACTTCCTATTCAAAAATATATG

>Unigene5682_All

TCAAATGCAAAGTAGTTATTCACCCAATCAGAGGATGTATCAAGCACATGGTCCTTATGATAGTCCTGCTCCCTTCAGGAGTCCAAGAACCAGTCCTTTTCCCATGCATCAAGAAAATGTAGCTGGCTATTACAGCAACCCCTCCGATACTTATACAAACTTATACAAGAAGTCCATACCCCAACCATGGAGGAAACCCAAGCTTTCAACCAGTAGGGAGCCCTGGTTTTTATTATGGACAACGTGGGCCTCCTCAGCATGGCAACAGTCCAATCATTGGCTCAGGGCGTGGAGGTGGTTCTCCTTATTCAGGAAGAACCCAGGGACAGTGGCATGGTGGCAGAAGAAACCAAGTATCTAGCTGGAGTGGTAGGAGAGGGCGGGGGTTTCATTTTCGTGGAACTGCTCCAGATGAGAAATTGGGCCCAGAGCCTTTTTATGACAAGTCAATGGTTGAAGACCCATGGCAAAATTTAGAACCTGTTGTGTGGAGGGGTCTGGACAGTGCAGTGAATAATNNNNNNNNNNNNNNNNNNNNNNNNNNNNNNNNNNNNNNNNNNNNNNNNNNNNNNNNNNNNNNNNNNNNCAGTTCTCAACAAAGCCTTGCAGAATACCTTGCTGCCTCATTTAATGAAGCTGTCAAAGATGCAGCAAGTGTATAATTGCTGCTCAGATCAAATGGAGAACCCTTCTGCCACTGCATGAGTGAAATTGATGCTGACGGAGAGATCACAGAGATCATTGCATTGTTCAACAAGAGAAGTAAGAAGGGTCTTTACCATGCCCAAGCCAAACAGGCATCTGCTGGATCAAATGATGCCCCTTCACCAGTTTTCTTCTTGGGAAATCTAGATGCCATAAGGCACCAAAAGTTTACACACCACTTTGTGAAAGGAAATCTACAATTGCATATAATTATAAAATTTAATGTGGCTTGTTACGTGGATTCCAGCATATGAAATTCTCC

>Unigene5689_All

CACAACTCTTAAGTTTTCTTCAGTTTTTCTCCTCCCTTCCAGCCAATTTGAACTGATATTCTTTCGTGTAGGATCTACAACTGCGTATTATGTTCAAGGCAATGGGGCACCGCTTACAGGAGCTAATGATCCTTTTCTTCTTAGCAATCTTCTCCAAGGCGGCCGAGGGCCTCAAGCCTTGCAGGGCAATCGTTGCACCGGATGCAGCTAATAATAATTATTGCCTCAGCTGGAGGTTGGCAGTNNNNNNNNNNNNNNNNNNNNNNNNNNNNNNNNNNNNNNNNNCACTATGTTGAAGCTTACATGGTGGGTGGGCAATATGATAGGGACATTGACTTAATTACAG

>Unigene5747_All

ATTTATTAATTTTTTTCCCACTATTCTAAATAGAGTTTTTCAAAAGAAAACAGAAGACCATATACAATACTGGAGCAACTTCCCACAGTACAAAATGATGTAACAAGGACAGCCAGCAACAGCATAAGATGTAGAAGCTCTCCTTGCAATTTAATTGGCTTTCCCGGAACAGATATCAAAGGCTGGATTTGCAACTTTGCAGCCTTATGAATAGTCTCACAAATAAGCTGAAAATATATTACATGCCTTCCTTCTCTTTTACCCGAGCAGAAATTTTATCTTTCTGTTTCTTTCTCTTTGGAGCACCAACTGAAGCTGGGTCCACCAATGGGCCAGACCATGTCTGGATAGGTGCTTTTGAATATGAGAAATTCGTGGAACTGAATGGGACATCAGGGGGATCAAAAACAGGATCAATCTGATGTGAAGAGCCCAAAGGATAACCAAGTGTTCCATCTTGGNNNNNNNNNNNNNNNNNNNNNNNNNNNNNTGCTCTTAGCATTTGCATGTGTTATTAAACGCCGTCTATCAAGATTGGCTTGAAGCTCGGC

>Unigene5760_All

TGAGTATCAAAACTATTGGGAACCTAAAATGTTTTTCCCAAATGAAGAGCTTGATTTTGATAGCTGGACCATGGATGAAACGTTTTCCGGCTATTACGATTCCAGTTCACCGGACGGTGGTGCTTCGGCGGCAGCTTCTAAGAATATTGTTTCGGAGAGGAATAGGAGGAAGAAGCTTAATGATAGGCTCTTTGCTCTAAGAGCTGTGGTCCCCAACATTAGCAAGATGGACAAAGCTTCCATCATCAAGGATGCTATTGATTACATCCAAGAACTGCATGAACAAGAGAGAAGAATCCAAGCTGAGATAATGGAATTGGAATCTGGGAAATTGAAGAAGAGTCCTGGTTATGAGTATGAGCAAGAGTTGCCTGTTTTGTTGAGATCCAAGAAGAAGAAAATTGACCAATTCTATGATTCTGGTGGCTCTAGAACTTGCCCAATTGAAGATCTTGAGATGAGCGTTGAATACATAGGAGAAAAGACTCTGCTAGTGAGCTTGACATGTAGTAAAAGAACAGATACAATGGTGAAACTGTGTGAGGCATTTGAATCTTTGAAGCTTAAGATCATTACAGCCAATATCACNNNNNNNNNNNNNNNNNNNNNNNNNNNNNNNTATTGAGGTCAGTACTCTTTCTCTTTCATTCCTACGTGTCAAAATTACTGTACATCCAGCA

>Unigene5798_All

ATGAGTTTCCTGCTTTTTTCACAGAAACAAGTGGCTGTAAGGCAACTTGTCGTGTTGACAGCCCAGAGGAATGTGCTCGGCTGATAGATGCTAATATGAAACTTAAGCTTGGGACTGGAATTTTGATTGCTGTTCCGATCCCAAAAGAGCACTCAGCTTCTGGAAATTTAATTGAGTCTGCCATACAAAGTGCCCTTAGAGAAGCCAGGGATAAGAATATAACAGGCAGTGCCGAAACCCCCTTCTTGCTTGCAAGAGTGAATGAAATAACTGGAGGTGCCTCACACTGGCATCAAATATTGCACTTGTAAAAAATAATTCTCTTGTTGGCGCAAGGATTGCAGTAGCCCTTGCTCAGCTTAGAGAGCATTCTAATAAAGGGAAGGCGTTTTGAACATTAACAATTCCATATAATGGTGAGATCACGTGGTTGCCACAGGATGATAGCTTCTTCGGCAAACAAATGATGCCCTTGCTTGCTAGTGTTACCTTTCACCCTGTGTTTTGATCTCCAAGTGTCTGGTANNNNNNNNNNNNNNNNNNNNNNNNNNNNNNNNNNNNNNNNNNNCGATTTGGTCCTCTATCCAGCTATATTAACTGATGACATTCTCCCAGTGGCTGCATCCCCC

>Unigene5815_All

AGCATCAATGACATGTATTATTTTTTACTTGTATGCCAATGGGGATGAAGGGTTAAATCTATGTCAAGATAACAATCAAGAACATTAATTGATCAGTGTAGAAACTGAATTAATCATTAAGTTTACAGTCAACATTATTTCTACCAAGTAGCCTACTCTAGAAAATCTGCTACCAGCTCATAATGGTTACATCAAACTCCTACCGATGAGGATCTCTCTTTATGATATGCTTACTATCTCATAATAATCCATTATCTTGAGTAGTCTAGGCTCCGGCTCTGGCTCCTACTTGGAGTCCTGCTATGGCTCCTTGGAGCATAAGGTGACCTTGAGTAAGATTGCCCCCTGTACCTTCTGTCCCTAGGAGAAATTGACCTTGAATAATGCCTTCGCCTAGGAGAAGGGGAATAATACACTGGGCTACGAGAATAGCTTCGTCTGTAATGTGGTGATCGCGAATAACGGAGTGGAGACCGCCTGCGGTCATATGCCCGATCCCTTAAACGTTCCCTAGCCCTCATTTCTGCTGGTTTCTTCCTATTTTCCTCAGCAAAGACAACAGTCAGCTCCCGACCAAGAAGAATTTCACCATCCATATGATGCTTAGCGTCTGCAGCATCAGCTGGATCTACATATTGGACAAATCCAAAGCCGCGTGGCTCCCCAGTATGGTAATCCCGAGGCAAGTATATATCCTTGAGAGGACCAAATTGCCCAAAGGGTCCACGTAGATCTTCTGGCCTGCAATCATGCCGAAGGTTGCGAACCAGAAGACTAGTAGGAAGGTCCCNNNNNNNNNNNNNNNNNNNNNNNNNCCTAGGACTCAGACTACGGCGTCTCCTTCCATAACCCCTAGGGGGTGAAGGACTATAACTG

>Unigene5816_All

CTGATACATATGACTATTTAATTATACACTTGTCTTAGATAGACTCAAAAAGCTCAAATCAAACTATTTTTTGATATTCTTGGTAGAAATAAACAACAATATCCACCAAATGGTTTTCCTCATCCTTATTTTTTCCTTGGACACTGCATGCTTTTCTCAAAGAATGTAACAGCCTATGTTTCTCGTACTGTATGTGAATCACCAAATGAGACCCTAATATCAAACTGAAAATTTTTCCCTTTTCTCTTAGTCAGAATCAGAAATATAAAATTTAAGAAAAAAACTACTATCCTAATGGTCGATGGTATCACTAGGAACATCCAACTCAGGGAGAGAATTGGGAGTTGACAACTACAACCAGTTTCTTCCAATCCAAGGACTCCATATTCGGGGAAAGGAAGACNNNNNNNNNNNNNNNNNNNNNNNNNNNNAACTTTTTGCGCAACAATGGCCATGGGATAACTTTCCCAATATCAACTTGCAGTACTTG

>Unigene5823_All

GTAAAAATATGGGATGGGTAAATTACATCAATGCTTACTAAATTAGTTACTGAATTATATTTTGTCACAACAGATCAATGTAAAATAATAAATCACTCCCCATAAGCAGCCACTCAATTTAATGTCACTGACCCACATAACCTTTTTGCTTGGCAGCAATAGCAACAAATAGTCTCATTTCCATTCCCTTGCGTTGTATATTTCCTCCATTGAATTTTCATACAATCAAACAATAGAAGTCATGAAACAGGAATATGGAAAAGTGCAATACCCTTTCTTTGTACACTCATTCCAATATGATCTCATCTTTTCAGGACGCCTTGTACAAATTCATTTCTTTTGCCATGTAAAAGTGCTATGCCCATTAAAAACATGAAGAGCACTTCAGCGATTCCAAGAAACGAGAAAATACATGATGGCAAGTGTTATGAGCATTCCCACATATTTGATCCATCTATCAACACGATTCCGCCTCTCAATGAGTCTCAATACAGAGTTGGAGAGCCCCACCGTGTTAAGAACATCCAGAGCTTTGCGTTGTACATTCTTCAAGCGTTCCCTTTGCTCTGAATATTTGGAAAGGATGGCTGATCCTGTTGATAAAGATTCTTGCAACATCCTTTTTGAATTTTGAACCGACTGCATTGCTTGTGCTTCCTCATCAAATATTCTTAAAACATGAGCTGATTCACCATTAGCTCTTCCAAGCAATTCTGCCCTCTCCTGAGCTTCCCTCATCCGCTTTTGATTCCTTGAGAAGTATCTATCCAGACTTTGTTTCAATGATTCAGCTTCTTCTGCTACTAGTTCTACCTTTCTTTTCCAGAGATCACGTTGGGGTTTAGCAGCGATTGAGCGCCAGAGACGATCCATTTCAGCGCAAAGGGACTGGATCTGAGAGATGTCCTTNNNNNNNNNNNNNNNNNNNNNNNNNNNNNNNNNNNNNNNNNNNNNNNNNNNNNNNNNNNNNNNNNNNNGTCTCGAGCTATGAGCAGGACCTTCTTAGCATTCTGATAAATCTCGGATAGAGTCCCTCCTCCTTC

>Unigene5835_All

TTGACAAAATAGCTGTACACATGTAAGGGTTTTCCATGGCTATAGATATCATTAGTTTATCAGTAATTTATGCAAATTATGCATTTAATCACCAGTTAATGCCCTCGGTATGTGCGCAATGGTAGACAAGGACATCCCAGTCTGAGAAACTGCTCTCACAAAGGCCATGTTTGCTGTTAAAGTTGGAGCATGCCAATGATCACCAGTCCCAAAAACCACCTTGCTCTTCAATGCCAGCTTTCTAGTTGTAGGAAGCCACATCATGCGCTCTGAAGGACTATCAGGCACAACCCTGTGCAATCACAAACTCAAAGTTACCCTTAAACCGCTGCCTCAATTCAATCTCTGGTGTAGCCAAAAGTTTCTCACTACAACCATTGCTGATGCCCGATACAAGAGCAATAAGAGCTGNNNNNNNNNNNNNNNNNNNNNNNNNNTTTGGGAGACCATAACTTGAGTCTTGAAATAAGAGAACAAAGCCCATCATCCTCATCCAAGTTTACTTTGGTGTGCTCTTCTTGAAGCTCTAGCCCAACAGAATCAACAACTGATTCTTGAACAGACCCACATNNNNNNNNNNNNNNNNNNNNNNNNNNNNNNNNNNNNNNNAAGCGAATGTTTATATGGTGTTCGAAGCACATTTATCCAATCCCCTTCCAATTCCTCGCAGAAATCAATCTCAAATGCCCCAAATTCGTCACACAGCTTCTGGAAGAAAAAATCACCAAGTCCACGAGAAAAGAAAAGAAGAATTGAAGAAGGTTTGAATATCTGGGCAGAGCGGGCACCATCCAGGAGTCGTTTAATTCTCGATTTTAAGTACAAATTCCAAGAAAGATACTTGGGATTTCTATCAGAAGCAATGATCCATACTGGGTTTTNNNNATTGACAGTGCAAACTATATCAACATGGACAGTGAGTGCTTTCGAAGAAGAAGAAGAAGAAGAAGAAGACGACACAGGAATTGGCTTGCAAACACGCGAAACCGCCGTCACAAATGGCTGCTCGAGAAGGGGAACGACGGCCTCCAGGTGGCCGACGTTGACACTGAGGGATTGAGTAGGAGAAGGAGATCGAGAGAGGAAATGGAGCTCAGACANATTTGAGGAGACTGCGGGAGCACGAAATGGTGATGCTGGTGCGAGCAGGTAACCTGTGTATTCTATCTATGATTGTTTGGCATCTATTTCTTGCGACTTGAATTTCCTTTTCCGCC

>Unigene5893_All

GGGGTGTACTCTTCATCACAACATATTTTTTAAGTAAACAAATATCTATTAGGCATGTGTAATATTTATTTATTTATTCCCATTAAACTTGTACATTCTTCCAGAAATGAGATGAAGTCACTATTGAGTTTGGTATATTCTTTTAGAGACAAGAACTTTCTCCCAAGGAAATGCAGATACAGAAACTTCCAGACCATGGGATTAGAAAATATGTTGTGATGAAGAGTACACCCCTTCTAGAGAAGAAGAGAGAAGTCAAATCTGGAAGGTGAACAAGATAGCAAATAGGAGAAACATATAAATAGATNNNNNNNNNNNNNNNNNNNNNNNNNNNNNNNNNNNNNNNNNNNNNNNNNATTAACAGCCACATTAGCCAAAGCCTTTTTATGGCGTCGACCCTTCTTTAGACCCCAGCCCTCCAATTTAGCGATAGGACATCCACACCTCGCTCGGAAAAGCAAAACAGCCCTCCCATTAGGTGGAGCCATCTTCCGCAATTCAGCTCGCAGACACTCATAATCAGGATTCCCTTCACTCCCACCTTTCCATGGCAACTTATCGATCTCCTTGGCCCCACTGGATTTCACACAATCCTCCGTCTGCAACTGCAAATCGAACTCCATCGCCTTCTTCAACCCCTTGCATCCAGACTTCTCGCACTTGCGAGATCTGGGCCCGCAGCAGAACTCAACAAAAAGGATAGAAAGGAGGAACAGCGCCAAAAAGCCGCAAATGTAGGGTACGGGAGGGATATGGACGGAAAGATGGGGGGAAGAGTGAGAGAGAATAATGGAAAGGGAGCGAAAGATATAGGAAAAGTAAGAAGTTAAGAGGAAAATCCCAGAGAAAAGAACCAGAATCAGAATTAGAATGTCGAGGGTGGCAGATGGAGAGTGTTTACACAGAAGGGAAGAAGAAGAAGAAGAAGAAGA

>Unigene5938_All

GCATAAATAAACACAACAAACTCCAAAAATTGGAGAGTAGACAGAGATTTACCTAAGGGAACTCCAGACTTTGGTGGTTATTTAAAAGAAAGGCAGAAGGATATGTGGAGTTTTGCTGAACATTTTGAGGGTTGGCCATAGCTTATTAAGACAAAAGGGATGGCCGTAAGATTATATGAACACAATTATCAGACCTAACGTGTTCTAGACCTGAATCATGGTACCATTGTGGCTGAGAAATGAAGCCTCCTATTTGAGGGATTGAAAAAGGAAGGGCAAAAAAAATATATATGTTTTCATGAATTTGATCAAAAAGTTGTTCGATATTTTGCCTATATTGAGGTATGGGCCTCTGTGCTGCTGCTGCTCCCAGTTGAAGTGCCTGAGCTCTGGCTAGACTGTTGCACACCAGAAAGAGGAGCAGGCCCACTCTTTGAAAAGGTTGAACCCACCAATCCGCTAGATGTGCTTGATGATGAAGACATCCTGGTTGAACTGGAGTCCTTGCTTGACCCTGAAATAAGTTCAGGTCGCAGTTCCGAAGATGACGATGATGGGGCTAGAGTACCGATTGAAGAGCCCACAGCGTAAGGTGCAACAGGCATATCAGCGAGAGAAGATGCAGATGGACTGTAACTCAATGTTCCCATTGGATGGTCAAATTTGCATGCAGGTCCAAACTTGCATTGTCCTCGTTGTGTATAGTGGGTACAAGTTGGTGCACCCGGACGCAAAGGAAGACCAATGGGGCTAAGAAGAACAGTNNNNNNNNNNNNNNNNNNNNNNNNNNNNNNNNNNNNNNNNNNNNNNNNNNNNNNNNNNNNNNNCAATCACCAGTTTTCATGTAATACTGGCATTCTGGTTGGCCAGGTCTTTCTGGAAACGAGC

>Unigene5965_All

ACTTGTAAATGAGCAATATACAAAAGAAAATACAGAACTCGACTGGTTAATCATCGATTCTTTGAATTTCACCCATCATGATCCGATTGCTTGTTACTTTAAAACCAAGGAGTGCAGGATCAATTTGCCTCCCTTTTTTACCTTTCTTTTTGCCACTTCGGCCCCCTTGTGTACCATCNNNNNNNNNNNGGACCCCGCTCCTGTCATGTGGACCTCTGGAGCAGTGGTCTTCTTGGCATTACTTTTAAGCATGTCTATAAATGATGGCTCTGACACATCAGCATCCCCACAAGATGAGGTTCTTCGGAAAGGCACATCTTTCTTGCCGGAAGCCGTGCTGTCAATACCATGGCCCACTGGATTTCCCCCCTCAACACCACTTGAACTTTTCCCCCTGACAACTGTATCAGAGACAAGATCAGATAACCTTTCTTGAGATGATGAAGCAATAGANNNNNNNNNNNNNNNNNNNNNNNNNNTTGCCCTTTGGACAGTACAGGCACCCGATTTAAGTTAATATCTTCAGCAAACGAATTTTGTTGCCCAACCTTGTCATCATAAAATCCAGAAACAACAAGAGAAGAATGCCTGCTGAGAGCATTAATAGGAACCTCTCCACGATCCATAGTAACCAATCGTGCTTGATCAGCTATGCCATCGTTAATCTCAGTAACTGAACTCTTTGCCATACCAAGTGCTTTGGACCCATACTTCCTCCCTTCCACCTCTAGGTACTCTTTGTTGATAGAAGACTTATCAATGAAGCTAGAATCCGCAAGAACAGCCTGAGAAGTCTCAC

>Unigene5987_All

TTTTATCCGTTGAGCGATGGCCCTTCCATTCAGAACCACCGGATCACTATGACCTGCTTTCGCACCTGCTCGAGCCGTCACTCTCGCAGTCAAGCTAGCTTATGCCATTGCACTAACCTCCTGATGTCCGACCAGGATTAGCCAACCTTCGTGCTCCTCCGTTACTCTTTAGGAGGAGACCGCCCCAGTCAAACTACCCACCAGACACTGTCCGCAACCCGGATCACGGNNNNNNNNNNNNNNNNNNNNNNNNNNNNNNNNNNNNAGGGTGGTATTTCAAGGTTGGCTCCACGCAGACTGGCGTCCACGCTTCAAAGCCTCCCACCTATCC

>Unigene5996_All

TGACTTTCATAACGGAACCAGCTTACCGTGAGATTCTAAGAGCGTTTAAACGCCAGATTAAGTTACCAAGTGCAGCTGAGCTGACTCCGGGTTTCGATCTTTGTGTGAATGTGTCGGGCGTTTCAAGACCCACCATGCCTAGAATGAGTTTCGAACTTGATGGTGACTCGGTGTTTTCGCCTCCATCGATGAACTACTTCGTCGAAACTGCTGACCGAGTTAAGTGCTTAGCGATTCAACCCGTTAACGCAGATGGCGATTTCTCTGTGATAGGGAATCTGATNACAAGGATATTTGTTGGAGTTCGATAGAGACAAATCACGGCTCGGTTTTTCACGTCGCGGCTGTGCTATCCCCTGACAATAAAACGGAGNNNNNNNNNNNNNNNNNNNNNNNNNNNNNNNNNNNNNNNNNNNNNNNNNNNNNNNNNNATTGGATTTTTAATTGTCAAATTGTTGCTAAATATAAATTAATATTTGCGGAGATTGTGGGAATTTTTGGACCATTGCGCAGAAATCTTTCACCCTCCATCATGGCG

>Unigene6029_All

ATCAGATATATATTTAAAAAAAAAAAAAAGGTATAACCTCTTAGAACACAAGCACGCTACATTATTCTACAGAAAATTGATATATGGCACATGGTGATTGGTGAAGAATAAGGATTTGTTACTTGTGAGATGTCAAGATTTATTGACATATAGGACAGGAACTAGCCCTGCCATTTTACCATCCCTTCCAGGACGCTTCTTCTTCACATGGTACCATCCATCAACTTCGTATTCGATTTCGACCTCTTCTCCAGCTGTTAAATTTAACTCATCGTCTCCACCAGCAGTGAAGTCATAGAGTGCCTTTCCTGATTGTGGATTTCCAGAAGAAAGTCGTTCCNNNNNNNNNNNNNNNNNNNNNNNNNNNNNNGAAGCTAAAGGATTCTCAAATGACTCGTACCTCTGCATAACAGATGATGCATATGATGGAGGCTCTTCTCTAATCGGAGACCCTGTCTCATAAATTGATGAGGAGCCACCATACCTTCTTCCCCCTGACCTCTCCGAGCTCTGTGATGCTCCATAATTGGTTGGCTTTGAACTGAACAGTGATGGGTAGCTCATTCCACCAAAGTGGGATGATATGGAAGTTTCAACAGATCCTGTTGAGTCTGGGGAAGATGTCCCAGATGACCTCCCATCATCTTCCTCCAATTCTGAAGGCTCCAATAGTGTCTTTGCCCACATATCATCATAGCTAACTGAAGGTCTAGAGATTATATTCTCTTCTTCAACATCGGGAGCATCAGTTCCAATACCAGCAAGAAATTCATTGACCCTATTCATTGCAGGTGCACTGTTTCCCAATAACCCATCATCACCAAGATTTGCTGCCCAGGCATTCACCAAGTCATCATCAAGTGCATCAGACTCAACAGGAGGTGGCTTAGATTCATAAATTAGGTCAGAAATTCCAGTGGCAACAGCTGGGTCACTTAAACCAGTTGATGCACTTATGTTGTGCTTTGTACGGTAGACATCAATAAGTTTTGCACTTATTGGCCCCAAAGGTAGATACTTTGCTCGGGGAACATAACAAAATAATGAAACGAGATCTAGCAACCGTTCATGGGTTTCATAAAGTTTCTTCAACTCTTCATCCGTCCATTCTTTATTTGCGTATATCCTTGATCAGTTCATCTTGTGCTCTGTACATTTCATCAAGAACCTTTATCATAGGACTAATTAAGAATCCAAGGCCTGTACCACTGGCTCCTTGATCTTCC

>Unigene6044_All

GTAACGAAAAGCAAGTTGCAGAACCTCAATACCTTGATAGATAGAAATATATACATTACACCACGACTGGTGCTTCCCTCTAAACACAATGCTTTAGAAAGAGACAGCCCTTTTTTTTCCCAATCCTTCTTTGACTTTTAATTACCATTCACACATTGAGCAGCATCACAAAAAGATGAGCCACCAGACAATAAACACAATTAAGTCCTCCAGAATCTTGGAGAAGCATGTGTACCAAGTAGCTCCCACCAACCAAAGGATTTACAAGTTCTAACCGTTGTGGCAGCATACCTGGCACATTGCTGGCTAACTTGGACCTGGAATAGTTATATTAATCATCCATAATTCTGATGGAATCACCAGATTTAGTGCTTCCTTNNNNNNNNNNNNNNNNNNNNNNNNNNNNAACTGTATTTTTGGAGCTAGTTGTTTTGCTACAAGCACCATGAGAATCCACATCTGCTAGACCATTCTCCAATGCTCTAACCANNNNNNNNNNNNNNNNNNNNNNNNNNNNNNNNNNNNNNNNNNNNTTGTTCGAAACTCATCAAATTCCTTTGAGGGACCTTCCGCATTGAGGAACTGTTGTAGTTCCTTCTCTGCGCATTGATGAAGCCTTTCCA

>Unigene6074_All

AATAAAGAGAAATACATTACAGATACTGTATATACATGAGCAGATATTCACAATACATAATGTTCTTTATTACCACAAGCATTTAAACAATACAGCTATTGATAGGATAGACTGACACAGACCTTACTTGAAGCATATTTTTATACTTGACAAGAATTAGCCTTCACTTGTTTGCAGCCATCCAAATGGAGAAAGTACCTTCGGTTGGCAAGGTCAAGCAGCACGGAAAGGCTCATCATTCTTTAGCCNNNNNNNNNNNNNNNNNNNNNNNNNNNNNNNNNNNNNTCAGGCAGCACTTCTGGATTCCTTTCCTCTGCAATATTTGGATGATGGGATGGAGATGTACTTAACGC

>Unigene6192_All

CTGAACCAGGACCACGTGGATCAAATTCATCAGCCACCTCGAATGCCTGGCCAACAGGAGTTACAGGTGCACTCAAAACAGTAGTTGTTTGACTTTGATTATTGCTTGCAGATGGAGAAGCAGCTCTGGGTGCAGGTGGAGAGGAAGCTCTAGGTGCTGCTGGAGAAGAAACTCCAGGAGCAGATGCAGTTGAAGTTTCTCCATTCCTNNNNNNNNNNNNNNNNCCGGGATTCACTTACAGCTTCTTCATAACTAGGAGGAGCAATATTCTGTTCAGAAAATTTTCGTTCAAGCCGCCTTCCATCCTGAGATTGATCATCAGCTCTAGCACCACTACCACGAGATGAGGATTGAACATCATCGTCAAAACCACGGTCTCTATCAGAACTCCTGCTTCTTGAGCCATAATCATCAATCCTTTGACTTCTTCCGTGGTAATCATCTTCCCTGTATCCATCTCTGCTGTAGCGTTCCTCATAATCTCTGCCATAACGATCTCCGTCACGACCATACGAGTCCCCATATCTACCATATCTATCATCATCCCTATAGTTGTATTCTCGTTCTCTCCCATTGCCATATCCATTACGATCTTCATCCCTGCTTCCATAGCGGCCTTCATAACGGTCATCGTCATATTTGTCACCATATCCTCCGGTACTTGAATATGAACTGGGTCTATACATTCCACCAGCTGACATGTTGCGGAACTTGTCCCGATTAGCAGCAGCCTTTTGTCTAACTTC

>Unigene6268_All

CTCTGTGCCACCAAAGGAATCCAAGAATTGGCGTAACCCAGCCCGATTCTTATTGTGGAGGGACCATGTTACGTAGCGTGTGGTGCTCACTAAATCCTCCATACATTTTTCATGGCATGCTCTTTCTGTAGATTCTGCAAAATTATTGAAGGCAGAAGCAACCCGCCTAAGGAATACATCATGACCGCTAAGATACTCACCATCTTTCTGAAGCAGATAGACAGAGATGGGAAGCAATCTCTTCAAGATATACAAGAGCCTATTACCCAACTGATGAAGAAAAGGTTCAAATGTATCACGAGCCTTTGCAACAGCTATTACACATGCTGTCCTAGAGTAGTTAGTTCCATCATGGATGTCTTCAACTCCACATGCATTTACAATTTCTTCGCGAGTAATTTGAGGGCATTTGATTCCCCCCACAACAAATCGAAATTCAGCCATGGCTCGATGATACTGTGCACCCCCATAAAGACGCATCCCTGCATTAGGAATTAGCTTTTGCGGAAACTGAAGACCATCAGTACCAACAAATGCCCCTCCATTAGTTCTCTCNNTGTAGCGTTTCACCAAATTTATCTGGGGGAGCAACAACCGTTCCTTTCAACAGCAATGACAACTTGGTTAAGAATAGGTCATGGAATTCTCTTCCTTTCTCTTTCAGTTTGACTTCATCCAAAGTACTGAGTTCTTTAATGATTTCATTCAGCTTTCTTGCTGCAATGCGGGATTCCTTTTCAAGAAGCGGAATAATCAAAGGAACACTGTCCATATACCTCTTCAGTAGCAATTCCTCTAAGAATGACCTTAGTTTGCNNNNNNNNNNNNNNNNNNNNNNNNNNNNNNNNNNNNNNNNNNTCCAAAGCTGCAACATCTTCCATCTCCCTTAAGGATATGGCCTGTTTAAACTCATCATTGGATCTGTAAACCGAATCATGACCAGAACCAACTCTTCCAGAAGGCACGGATGTGAAAAAGGGAGAGTCACCCAGTATGAAGCCATCCAGGGTATGTGTAGGAGGTGAAAGGAAAACTTCAACATCTGAAGAGCGAGCAAACTGGGGTATTTTAGTGTCAAGTTTGGTTGAGACAATAACAGTCCTTGAAAGTTCAGGATCAATTTGCATTACTACTCTTCGAGTAGTTGCATTACTCCAGTCACTACAATCTTCAAGACATAATATAATGAACTCTTTATGCTGCATTTTTGCTCGCACAAGAGACTCAACAGCTCGAGCTTGG

>Unigene6280_All

AAATTCCTCCCTGATTCTGATCTCATCAAGGAGTTGAAAATAGAAGGAATATCTTTCCCAATCCCCTTTCACAATGCACCCAGGCTCACCAAGATGTAAGCAATCACTGAATGTACATTTAGCAGGTCCAGTGGCACTAAGCATTTTCCTGATCTCGGGGAATGCCTGTGCAAGAGATTGCTTTGTTACTTTAAGCAAACTAGGCTGGTTAAAACCAGGAGTATCAGCGAGATACCCCCCTCCAGATAGTGGAAGCAAAGAAACATTACGAGTGGTATGCTTCCCTCTGCCACTTCTTGTTGAAACTTCCCCAACACGCTGTTCCTCAAACCACTTACTTCCTAATATAGGGTCAAACCAATTATCCACTTCTGCAGCATCACAGGCACGGGGGTTGTTTCTCAGAACATTTATCAGGCTAGATTTCCCAACTCCACTAGGACCCACANNNNNNNNNNNNNNNNNNNNNNNGAGAATCAAGTTCACGCTTGGATTCAACACTACAAAACACTGGTTCATAACCCCAGCTGCGCAGTCTACGTTTCCAGGCCAGCAACGTCCCTTCGTCAACAAGCTCCGATTTATTCAAAGCAAGCGTCAAGGGAATCCCAGTGGACTCAGCCTCAACCAGAAATCTCGTAAGGGTGAATGGCTCAAGCTGCGGCTGCTCCATGGAGAAAAGCACCAACAAGTGATCCACATTAGCTATGGGAGGGTCCAAAATTTCTGAGCTCCGTTGAAACACATTCTCGATCATGCCCCTTCGGTC

>Unigene6286_All

GTCTAACTCAAAATGGGGTGATAGGGAAGGGGTGGGATTGGAATAACACAGATCTACTAATGCTGGCTGACATATTATGAACTTCCTCAGTTGAAGTCCTTCAATCTTTTCTTCAGTACATCATCCATTGATTTCTTTCGTGCACTTTGCTTTGTATTTCTGCTTGAAAGGCTACCAAAGATTCCATCAGGAGAATCCTTGAATTTCTCACAGACGCTGGCTACTTTCTTATAATCCAGAGCATTGTAGTTTTTCTGTCTTATCACCGGATTCATGAAGTTCTCTGGTTGGCTGCTAGACAAAAGATTAATCAATTGTCTTGCTTCTATCAAGAAACCTCTAAAACTACCATCTTTATATATCTCACTCAGGCCAGTGTTGCGAAATCTCTCATCAGCGAAATTCTCTAATATCTTTAGGTCATCATTAAGGGCCAGAACTGCATTAGCATTGAACCTCTTAACGCTATCACTGAGAAATGCAGCAACAATGGAATTGGAAATATGCTCAAAAGCTCCACTTCCAACCTTGTATAGAGCATCCACAGGTAGAATTTGCTGTGCTGTTGAAAGAATAGTGTCAAGGTATATTACCACCTCATTTATATACTCATTCCCATTTNNNNNNNNNNNNNNNNNNNNNNNNNNNNNNNNNNNNNNNAGTGCCATAAACTCATCCAATTTAGTGTTCACCAAATTCAGCAGAGCCAGATATGCCGCATCCTTAGAAGTTCTCAGAACCACCTTGGCAGTCAAACTGGCATGAGGTCTTTCAAC

>Unigene6351_All

GCAAAACCAGGTGAATTATCTGATATCTTATGTATTTCAACCTTCTGATAACTTATCACTGGTTGCTTCAAGGTTTGGAGTAGACACACAGTCGATTGTAGATGTTAATGGCAACAGTCCGCAGCCTTTTAATACCATCTTCATTCCAGTCAATCGACTTCCAATATTGTCACAGCCTACAGTTGCTCCTTCTGTGGCATCTGGGAGGAGAGAGAGGAAAGGTTTAATCACAGGATTGGCAGTCGGGCTTGGAGTTACCGGGGTTTTGTTGATTTTGATAAGTGGGATTTGGGTTTATAGAGAGGGTAAGTTGAAGAGAGAGAGAGAAGGGGATGAAGAGAAGCGGAGGATGAACTACTATGGAAAAGAGAAAGGTTTGAAGGACATGGAAACAAGTTTGCTGGCAGATGTTTCAGATTGCTTGGACAAGTACAGGGTCTTTAAAATTGATGAATTGAAGCCACTGATGGGTTCAGTGAAAATTTCTTGATTCAAGGGTCTGTGTATAAAGGGTCCATTAACGGGGAGGACTACGCCATTAAGAAGATGAATTGGAATGCTTACGAGGAGCTTAAGATCTTACAGAAGGTAAACCATGGCAATTTGGTGAAGCTAGAGGGCTTTTGTATAGACACTGAGGATGCAGGTTGCTACCTAATCTACGAGTACATAGAAAATGGCTCTCTTCATTCATGGTTACACGGCAACAGGAATGAAAAGTTGAACTGGAAGACAAGATTACGCATTGCTGTTGACGTTGCCAATGGTCTCCAATACATCCATGAGCACACTAGGCCAAGGGTTGTGCACAAAGACATCAAAAGTAGCAACATTCTTTTAGATACAAACATGAGAGCCAAAATTGCCAACTTTGGACTAGCAAAATCAGGCTGCAATGCCATAACAATGCACATTGTTGGTACTCAAGGCTACATTGCACCTGAATATCTAACCGATGGTGTGGTTTCAACAAAGATGGATGTTTTCTCCTTCGGTGTGGTTCTGCTTGAGCTCATATCCGGCAAGGAAGCTATTGATGAAGAGGGTAAGCTTTTGTGGGTAAAAGCTAATACGGTTTGGGATGGAAGTGTGGAGAAGAAGGAGAAAAGGTCGAAGGAGTGGATGGATGATGTTCTTTCAGAAGAATCATGCTCAATGGAGAGTGTAATGAATGTAATGAGTGTTGCAGCTGCTTGTCTCCATAGAGATCCTTCAAAGAGGCCTAGCATGGTGGACATTGTTTATGCTTTGTGTAAGAANNNNNNNNNNNNNNNNNNNNNNNNNNNNNNNNNNNNNNNNNNNNNGAACCTCAGGTGACGGCAAGATAAGTTGACTTTTGGTAGTTCAAATATATAGTTTCGTG

>Unigene6380_All

CAAAGCTCTGGACATGATCCTATAATCTCCCTTTTTCTTCAAGGCTCTCCTTATTCTGAGGTATCCCTACTGTCTTCATGGCACCCTTAGATATAACGACGCTTCTTTGTTTTCGTGAATCGGATTGGCCCATGCTAATGACTTATTGGATATTCTTCTTCACAATTCCCTCAAGCATTCCATCATTCCCAAAACGGCCTGAAAACTGTTGGTCATCAACTGGACCAGGTAGCTGAAATTTAATGGAGAAGTGCCCTGGTTGGGGCAGATTTTGTGTTTGCATCCTAAAGACTTGGGAGTTCTTGCACACTATTTTTTCACCAGTGGTTGTCACGCCCTTTATTATTATTGTTCCATTAGGTTCAATGTCACAACTAAATTCCTTTTCATCTTTTGCTACCCCAGGCAGGGAGACACGAAACATGTAGGCGTCATCGCATTCCCCAATGTCCATTAATCCCACAATTGGTCCCACCTGTCCCATAGCTGCAGTCCCAGTCAGCACAACTGCACTTTTCGCAGAGACCACTATGTTATCCCATTCCTTTTTAGTTGTGTTAGATGGGAGAAAGACCATACATGGTCCAACTTTTTCAACAGGTTGGGCATCACTTTCAGTTTGCAATGAGTCATAATCACCAACTGGTGCAGCTGAACTAATGTAAGGCACACTGTTGAGAGGTTGAACCTCGAGTGCTTGTTGGTGGGGATTCTGGAATTCATTATTTCCTCCATGCATGCCAAAAGACCTGATCGGGGGAGGCATGGTGGTGTTTCCGACGAACGTCGGGCGATGAATTGAAGTGTCGGCGACCTATTTCGGCTCNNNNNNNNNNNNNNNTGCGTCTCTTTCACAGGCAACTGAGACAAGGTCTTGGGTGTAAATGATCTGCTGTTTTGTGCG

>Unigene6394_All

GTTCCTTGGTGTGGAGAGATCTCCGCCATAAAACTAAAACAGAGAGCTCGTAGGCCTTATTAGATCAAAGAGCGACGTAATTCCCCATTCATCCGAAGTAGTCTTTTAGTATAGAACAGAGGCATTCTGAGCCGACTACTATGACTACATGCGCATCTAGTGCAGTGGCTTGGAAGCAAGCTACCTTGACCATCTTCCGAAGTTCTAAATAATCTACTGATCAAAGGCTGTAGGGGCGGACTGCTCTACATTCAGCCACGCCATAGTGACCCCCGAAGCGATCTTCTTCTAGTTGCGGGACGAAATCCGGCAGCCAATTGCTGGCTCTGAATAACCAGCCCAGCAATAAGTTCAATTCTTCCATAACATAACGGGGCGGGGTTGCGCGCGAGCCGAGTGACGTAGGTGCACAAGAGTACTTCGCGCCACAACCATCTCTTTTTTATAGGTTCTACGGACCGATGCCTGCTGCTTCATCTGGGAGAAAAGAATCATAGATATAAAAAGAAACACCCGCCATAAAAAGATTCCTCGTGTATCATCTGTAGCAAAACTATGAACGGAAGCTAGCAATCCGGACCGTATTGAAAAGGTTCCTAAGACNNNNNNNNNNNNNNNNNNNNNNNNNNNNNNNNNNNNNNNNNNNNNNNTGAAGAAGGGGTAGAATTACTGAATGAATACAAGCTGTGGCTAATACCCAAGGCATAAAAGAAGCATTTTCTACGGGATCCCAAAACCAC

>Unigene6416_All

GCGATCTTTTGTCAAAGAACAATGAATCATGACAGCTTGTCCAGTCCAATTTTGACCCACTCATCGTCTTCCCCCATGATTCCTGGTTGGAATCGCTACGAGGATAAGCGCTTCGAGGAAGCACTGATTCTGTTTCCGGAGAATTCTCCTGATCGGTGGCAAAAAATCGCTGATCATATAGGAAAATCGGTGCAAGAAGTGACAGAGCATTACGATGTTCTGGTTCATGATATTGCCGAAATTGAATCGGGGCGCGTAAAGTTACCAGATTACAGCGATGATTTTATTGACTCGAGTTGGGACTCGTACATGCCAGCGTCGATNNNNNNNNNNNNNNNNNAACAAAGCTGGGCGAGGCCGAAAGAAAGAAAGGAACACCGTGGACGGAAGAAGAACACAGGAAATT

>Unigene6448_All

TGAGCATCTTCATTTTCTTATAATCTTTCAGTTATGAAAACTGTAAGATTATAAGAAAATGAAGATGCTCATAATTAAAGATGAGAGGACCTTTTTGCTCAAGTCACCATTTAAACATTCTCTAACAAGTGAAGGAAAAAAAAAAAGGATAACAGATTCCATCCAGCCACCAAAATCCACCTTCACCCCATTGAGAAATAAATAATTAGATAAAGACCCACAGAACTCCCAAGTTATGATCCTAACCTAAAATGTCATCAAGGTGCTGATCAACAACCAATGTCCTGGCATAAAAGGATTCAAAAACCAATGTCCTGCATAAAAGGATTCAAAAACTTGACATCTGCAGGAGTCTGTAGCTGATCAACTGGCAGATTGCAATGAATCAGATCTGCAAACATCCCCACGACAAAGTGGACATACCCTGTGGATCTCCTTGAGCCATTTGTCTATACATGTTCTATGAAATTCATGATGGCAAGGCAGTATCCGCATGCTGTCCCCTTCCTCATNNNNNNNNNNNNNNNNCATTGAGCTGCCTCCTCATGCTGTTGCTTTTGCAACTTCGTGTATAATTTGACTGGCAAAGATTCCACTACTTCAGTAGGTGCAGGTACAGATCCAAGTGACGACATTGATGGCCGAGAGGATAGCACCACAGATTGCTGGTGAATTTCATCCAGAACCTCAAACAAAGCTTCAGCTAACATGACAATTCTTGATATGCTAGCTCTAGCACTTGTGTCATCATTTAAATTCGGATCCCTATTAGTTAAACGACATGCACAACGACCCGTTCTATGATGGCCGGATAATATACAAGATCTCTCATGTCCAGACAGATTCTCGAAACGGCTTCCCAACCGCTGAAGAGCACGAACCTGCGATCGTATTCTCCTCCTTCGCTCAAGGAAATTTGACCTATGTTCCAGTAGGTCATGATACCTGGCATCTCTTGATCTTGTAGGTTCCAGATCATGATCGTGAACACTGAACAAGGAGTTAGAGGTGCCAGAAGGAGGATAACCAGATGAAGTTGGAGAGCTTAAAGTATCACCTTCAGATTCTGATACCCTCATTTCACCACCTGAAGCTTGTCTCCCACTAACCTGAATGGCATCTATCACTTGTCTCTCTTGTTGCAAAGGGCAAAATGTAACATCAGATAGTGATGATCGACGAAGAACTCTGTCACGAAGTCTTCCCACACTAAGGGTGCGACTAAAACGAACATTACGTTCAACGGGTTCTTGTGCTCCAATCCGCCTATCAGAAATTCTAGTCTCAAAACTATCCGAATCACTAAAAATTCTCGGAGAATGCGAGGTTCCATCAATCCCCACTCTGGTGCCACCACAATCTCTGCTTTGATCATGAACAGAAGAATTTGTCTGATTGCCTTGATTATTGATCAAACCAGATGCTCGATAGTGAGATCCCAAATTTGAAGACGAGTCTTCACACTGTTGTGTATGAGTTCTATTGATCAAAGAGGTAGGCAGCAAGTTGCTACTTTGACAAGTTTCATTTCCCTCCATGATGCCATTGGCAGATCTTGGCCGAAGGTGAATCTCTTCTTCATCATTGAGCATTTGGAGGCTTGTAGAAGGGATATGGTAAGGCCTGGATGATCCTACACTGGTTGCTCTGCTTAGTCTAAAACTGACATTACCAGGAACAAAACTAAAGCGAGAAAGAAAGCTACTTTGAGATGTCAAGGATGGAGTAGAAGCAGCTCGGGCAGAGTTGTTACCCATCCTGGAGGTAGTATTAGTGATACTTGCTTGATTCCACTCATCAAGCTCAACATTAGATGATACACAAGTTTCATCAGAT

>Unigene6454_All

CATGAACACTCTACTATTATATTCCCTAGCAATCACCAAACATATACTTTACATTTCTTCACTCCCCATAAATGAGGTGTAAGAAGCACCCCTGTGACCCCAGTAGCACCGTCGGTGTCTGTGCCTCCTGTCTCCGGGAACGCCTTCTCGTCCTCATCGCCGCACAGGCTCGAGTTCAATACACACAGGACCATGCTCAATTGGCCCGCGCCTACTCACGCGCCACCGTAGATGATAGTCGGAAATCAGACGCTCGGCGACCTCCTATTATATTCCCTCGCTCCGTTTCTCCTTACGTTGCTCGACGGAAATCTGACGACACCTCCTGGTCCCACAACCTCCGCTTCTACAGTACTCCCCAGGTGGGACCCACTTACAGCTCCTCCTCCATCGCAACCGGCTTCGCCACTTCAGCAACTTGTAAAACTAAGCGCGCCAGATTCTCCCTTTTGTCGAATCTATTCAGGTCTAGATCCGAGAAATTTAGTCCAGATCCTTCTGGTTCCGGTCCTGGGGTTCCTTCTCGGAATTCGAGCGAGGCATCATATTCTTCGCCGTCCTGGCTATCAGCGATATTTTCCGGTCGCAGGAAAAAGCAATCTGCGTTATTCTCTGAGCAAAACTCCGGCGTTGTAGGCCATAAGCCTCGTCGGAGGATGGATCGAGGAATGTCGCCAGCAAGAGGGGCGGATTCCGACGACGATTGTGAGGATTGCAATCCGTCTCAGTCTGGCAGTGGGTCCTCTTCTGAGTCATCGCAGTGGTGGAAAAGGACACCAGTAGCGCCAGCGGCGATGAGGCGCGGGAAGCCGGGACACTGCAAAAACGTGTCGGGATTAGCGTTTTGTTTGAGCCCTCTTGTGAGGGCCAGCCCTAACCGGCACTGGAACCAGAAAGGCGGGTTGCCACCCGACATGGGGTTCTCCGGCGAGGTGAGGGTTCCCGTGAAGCCCCATCTGTCTGCGGCGGCGTCATTTTGCGCGAACCGGTCGAGGAAGCTATGCGATTTTGGAAGGGCCAATCACAACCGTTGAGAACGGGTCGGATCGTTGACATTTGATCGCGCTGGTGGTGATATTTTTAAAGTACGGTTTTGCCCATCCTCACTTTCGGTTTTTTTTAGTCATTAAATTTTTTTTAACAAATTAAACCAAATATTGAGAGAAAAGGAAAACAATGAATATGGAGAGGGNNNNNNNNNNNNNNNNNNNNNNNNNNNNNNNNNNNNNCGAAATGTGGTGTTGGTTTTCTGCGTACATTGACATTAGCGATGTTTGTTATTGGCAGAAACAAAGGG

>Unigene6461_All

ATGAACCCTCTTCCACTATGCCTTGATTTTATGGCCTGCGCAGCTAAAACCCTAAACCCTAACGCCATAGGAGCCTTTCTTCCAGATCTTTACCCTTTCTCTCTGCATCTCCCATCATGGCCTCATCTTGCAACAGATTCATCTCCAGATCATCCCTATCGTCCATTAAATCCTTCATTAGATCCAACGCTCCAAAATCTCCTGTCACAAGATCTACCACTTATTCCTCTCCCTCTCCTCATTTCCCTTCCTCTTCCATTCCTCGCCGCTTCTCTTTCTCTTGTAGGCCTCCGTCTAAGCTGGGATGCCTGCAGTCGCTGCTGCCGCTGCCGCTGCACAGCGCGGTAGCGACGGCGAGGATGACGTCATGCTTGAGCACCACATCGAGGAGTTGCCGAAACACGTGTGCTGTTTTTTGGAGAGCTTGGTCTGTCAGTTCCAAGGTGACCTTTTTGCAGAAAATGGGAGATTAACTTCTTCATTATTGAAGAAGCAAGGCCTAGTAGCTCTACAATTTGTGAAGATTTTTTTTTTTTTTTNNNNNNNNNNNNNNNNNNNNNNNNNNNNNNNNNNNNNNNNNNNNNNNNNNNNNNNNATAGGAGGACCCATCTACTGAGATGTAACAATTAAATGCAATTCATATATCATATCGCGCG

>Unigene6465_All

CAGCTACAGGGAGCAAGACTCATCTTTGTCCTATCGCTCACTTGCGTTGCCGCTCATTGACCGTTAAGTGTCGTGCCACGGTCGCCGGCGAGTCAGATATGGCAGGCCTAACCATCCCCAGCGCTGGAGCCCACCGGTCAATCAACGACCTCGCTGCCAATTTCGCTACCTGCTCTCGCCTTACCAAAGAAGCAGCAGCTGCTGGGGCAAAATTGCTGTGTTTGCCAGAAAGTTTCTCTTTTATTGGGGCTAAGGATGGGGACAGTGTTAAGGTTGCAGAACCTTTGGATGGTCCAATCATGCAACAGTATTGCTCCTTGGCAAGAGAGTCTGGCATTTGGTTATCGCTTGGAGGCTTCCAAGAAAGAGGTTGTGATGATGCACACCTGCGCAACACGCATGTTATCATTGATGACAGCGGGAACATTAGAGACACTTATCGGAAAATATTCTTGTTTGATGTAGATGTTCCTGGCGGAAGGGTGTACAAGGAAAGTAGCTTTACAGAAGCTGGAAAGGATATAGTTACAGTTGATAGCCCTGTTGGATGCTTGGGTCTATCAGTTTGCTATGATTTAAGATTCCCAGAGCTTTACCAACAGCTCAGGTTCCAANGGTTCCAAAAAGAAGCACAGGTTCTATTGGTACCTGCAGCTTTTACAAAAATTACCGGTCAAGCACACTGGGAGATTCTGNNNNNNNNNNNNNNNNNNNNNNNNNNNNNNNNNNNNNNNNNNNNNNNAAGCTGGAAAACATAATGATAAAAGAGAAAGCCATGGTGAGGCATTAATAATTGATCCCTGGGG

>Unigene6492_All

TTGTAGAGACACCACAAAAGCTTACTTGCTGAACTTGGGTATAATCCACACGGAATCACCAACCCCCCACAAGTCTCAAAGTGATGTCTGAAGGTCAGCTGGATGTAATAATTTCTATATTTGGGAAGGTACAATTCCTGATATTTTGCTGACCTAAAATTTTTAACTCCTTTATGAGCATGGTCGATTCTTTGAAGACACTTGTGGAGCTTTATTTCAAACTGAAAGTAATTTTATGTGTATTATAGCCTGCCTTTGTATTCCATACAGAGCTACAGATACAGAATTTGTTCTTAGAGCTGGTAATATTTGCCACAATCATTATGTTCTTTCAGCTTTGTACCGATGATCTTCCTTGTATNNNNNNNNNNNNNNNNNNNNNNNNNNNNNNNNNNNNNNNNNNNNNGGGTGTTGGCTTCATCCGAGGTTGAGGTGGAAGTTGGAGTTATTTGTTTTCTCCAATGACTTCAACCTCAGTTCCCCAGAAGTCACATTAAATGAAGGTATGTAGTCAATTTTCGTGAAAGCAATAACTAAAGCAGGCTCGTTGTGGAACCTGGGACGAACTCACTGATTGCAGTCACTTTTTGTTTATGTAACTATGAAAGCAGTCGTAGAATCATTTGAGTTGTTAAACTAACGTGCA

>Unigene6509_All

CATCAACACTTGATAGATACTGGATTATTCATGACTGAGATTTACAAGATGCATTGATTATATGGTAATGAACTATTTGGACTATCCTGTAAGAAAGTAAAGGAAAAACACCAGTTGGATTGGTTTATTGTTTCAGACATTCTCACTCTATATGCGTTTAATACATTATCTCGTGTACAAGCGAAGAGCCGTGAAGGCCTGAACAAACACACGTGCACTGAGTGAGGCAAAAGTTTGTGTGAAGCAGCCATTAGCCGTCTGGTCAGATCAGAGCAAGATGTAAACCCACCTTGCATTTCTGATGCATGATGGATTAATTTGGCTGCAGTGCTTCCTTAAACTTCCCATTCTAACCTAACTTGTTTTTTTATGAAAGTCCATCCAGCATAGGGAGCTAGTTATTGAGCAAACACCAGAATTTTCAGGTCCAGAANNNNNNNNNNNNNNNNNNNNNNNNNNAAACTGGCTACCTGAATGAAAACTGCACCATTGATTGGGTGATGCCAATGAGCCATCTGTGTATTCATCACTGTTGAGGAGTTCATGCCCTTCATCCCCAGAGTGCTCAATGTCCCTGCTCTTATTGTTTTGTGAACACATGAATTCTGTGTTGTTTAGTCCCTGTTGTGCTTCAGGATCATTATTGATGTTTCCATTGTTTGTAGCAATGAGAGTGTTGCTCCCTTCTAAACCCTTGCAAGTCATATTCCCATCACAAGGTTGATCCAATAGTTCATTTAGCTTCTGCAACTGTATGAGCAAAGACTGTCTTTCATTCTTCAAGGACTCAAAACAGGATGCTAATTTGTCATAATTAGCTCTTAATGTCTTG

>Unigene6543_All

GGAGAAGTGTGAAATGACTAGAAAGGGGAGGGACAAGCACTTGAAATGCAGAGAATAGAACCCAAGGAAGCATATTTAAGAAACGTCTCGATTGCATGTACAGAAGGCAAAGACAAGTGTCCTGCAAAATGACTAAATATTGTTATGTGTATGCACACACGCTAATTCTCTTGATTTCGTCTCTCAATTGATTCTAAGTTCCAAAAGCTCCCTTCCTCTTCACCACCATCCTTATAGAAGAATATTGACAGAAGCTAAATAATTAAAAAAAAAAAAGGTTTCTCATCCTCTTTGTATCTGTTTCACCACGATGACACCATCCTCTATCCTTCTCTCTCTCTCTCTCATTCATTCTCTTGTTTTTGTTATCAAGTGTTGGTTGCTGGTGCCTCCACAGGCACCACATATCTTCCATCTTCTGCACCACGTGTTCTAACGGCTGTATAAAGCCCATAACCATTTTTGCATTATAATTCACCATTGTTGCTCAGCAATCTCATATTTTTCCTTGCTGGGTTGTTCTTTTTCTTTCTCTTCTTGTTAGAGTTTANNNNNNNNNNNNNNNGGGTTGTTCTTGTTTCTGTTTTTTAAACTGAATCCCAAGTTAAAATTTCCTTAAAATTTACCACCAAGACTCTCTTTATCTATTTGTTTCTCATTTCCTGAATTTGGTTGTTCTTGGTGGTGAATTTGAAAAATTTTTCATCCCCTATGGCTTTAATTCTTCGATTTCGAAAGCTATGCTTTGTGGAGCCTGTGAGGTTCCAGTCGTATGAATCCCAACAATGCGAGAAGAAAGAAGAAATTGTTGCCCCACCTACCAAAAATGGCTTAGAAATCAACAAGAAAAAGACTAAAAGAGGGCCCAGAGAATTGAGTTGCATAGACTACTGTTGTTGGATGATTGGGTATATGTGCACTATTTGGTGGCTTCTGTTTTTCTTGTATCACTGTTTGCCGGCCACATTGCTTGGCTTTCAGGTCCCTGAATCACCTGGAACAAGACTTAAACGTGAAGGCTTGATTGCTCAGCATCCAGTGGTTTTGGTACCTGGCTTTGTTACTGGAGGCCTGGAGCTCTGGGAGGGCAAGCCTTGTGCAGAAGGTCTTTTTCGAAAGCGAGTTTGGGGTGGTACCTTCTCCGAGATGTTCAAAAGGTTGGTGCAGCAAGCACATAAAAGCAATCATGAACATTGGTCCAACATTTCTTGGTTCTCCAAAGATGGTTACTAGTATACTCACTACCGAGGGAAAAGACGTTGCGAGGTTCAGAGCTATGTTATCTGGTGTTTTGGATTCTGAACTTCTCGGGATTCAGACCGTAGAGCATGTCCTGCGGGTGAGTCGAACATGGGATTCAACTGTGTCATTGCTGCCTAAAGGAGGAGAGACTATCTGGGGCAACTTGGATTGGTTTCCTGAAGAAGGGCAAGTTTGTGTTTGTTCAAAGAAGAGATCCCTGCTAACCTCGACAAATGATAACAGCACGGATGTGAAAGGAGGTTTACAAGTGAAAGAGTCAAATAGGTATGGAAGGATAGTTTCTTTTGGCAAGTCAGCATCACAATTACCATCCTCGGAGCTTCCCTCTTTTGATACAAAGGAATTTTTTGGTTTGCATATGGACAAGAATTCAGGGTCCTCATGTGGACAGGTTTGGACTGAATATGATGTGATAAACAGGGAAAGTATACAAAAATTTGCAGAAAACAAAGCTTTTACAGCTGCAACTGTTTTTGATTTACTTCGTTTTGTGGCTCCAAAAATGATGCAACGTGCTGAGTCTCAATTCTCTCATGGGATAGCTGACAACCTTGATGATCCTAAGTATGAACATTACATATACTGGTCTAACCCACTAGAAACCAGGTTACCTGATGCAACGGATATGGAGATATACTGCTTATACGGGGTTGGCGTTCCTACTGAAAGGTCATATGTATTGAAGATATGCCCAGCTGATAGGTGCAAGAGTATTCCATTCCGGATTGACACCTCAGTTGATGGAGAAGAGGGTAGTTGCTTGAAAAATGGAGTATATTTAGTTGATGGCGATGAAAGTGTTCCCGCACTGAGTGCTGGCTTTATGTGTGCCAAAGGTTGGAAAGGAAGAACCCGGTTCAACCCATTCGGCAGTCGCACATACATAAGGGAGTATCAACACAAACCATCATCAAGCCTGCTTGAGGGAAGAACTACAGAGAGTGGATCACATGTTGACGTCTTGGGAAATTATGCTTTTATTGAAGACGTACTAAGAGTTGCAGCTGGAGCCACAGGTGCTGAGATTGGAGGTGATAGGATTCATTCTCATATCTTGAAAATGTCAGAGAAAATAAATCTTCAGTTGTGATTATATTAGAGGTATAGACACATTAACCACGATAACAGGAGTTTACCATCTTCATTGCACAGAAAGAGTACTACATATGTAGAGTTGCTGATTGAGGTTTGTTCATAACTGCATTTACTATTGAAACTACCGTGGAAGAGTTAAAAAGGTGCAAGAGTTTATGGGGTTCCTAGCTTGATGACAGAACTACGTCTGCGTGAGTGTGAAGGATATGAATTCATAGGCAAGCTGGAAGCATATGAGCTCTAGCTATACAGGGAGTGTCCTACAGGAAATCACTAGTGCCCTGGTAGACTGGTACTGTTGTGAGTGAGATTTGACAAGCTGGGTGCCCATTTTACCTGAGTTTATCATCAAATAAAGGATAAATCTTGTTTTTCTTGCTAAAAATCGAGCGATCAAAGCTTTGTAATCTTCTTTTTGG

>Unigene6616_All

TTAGTGTCATTGAGTTCTCCCGAGTATTGAGTCTCTGTGAAGTTAGTGAGTACAAGACTTGGATCCAAGTTGGGAACACAAGCAGGCATTACATATCCATCCTTTGTGTTGGAGACATTCAAATGACATTTGGCTAAAAGACTTTCATAATTGGGGATCTGAGGCTCAGTGCCTGGCACTGTATTGGAGCCTGTGCTGTTCGTACAAGGCTGACTCTTAAATTGATCAGCTACTTCCTCAGCAAAGCATTCTGCATTGTCATTCTTTGTAAGGACAGTCAGATTCAATGATTTCCTGTAGCTGTTAATGCCTTCAAGAAGTGTATCCTCCTCATCACATTTGANNNNNNNNNNNNNNNNNNNNNNNNNNNNNNNNNNNNNNNNNNNNGTCTTGAAGAAGCCATTGTTGTCATAATTGTTCTGCTGTTGCCGCAGAATCCAGATGCGAG

>Unigene6682_All

AAAACTTTATAATTAACTTTAAATAATAAAAAAAATTGTGTACTGGGTCGGGAATTATTTTTACTGGGCTTTATAACCAAGCGGGACTGGTCCGCGCGCAATTAAGTTTCCAACTAATTAAACAATTACAATTTAAGGTCCTGAAAATTAAATCTCAAAATTTCAGCATTGAATGCTCAATCACCTTTGCATTTACGTCACAAAGGATTAAAAATTGTACGCAACATACATTTTAGAGAAGCTTAGCCAACCCTACAACTCTCTTAATTCTTTAACTGACACAAAGAAATACGAAATACACTCAGCTTCCAAATAAAACCAACTTACTAGATAGTAGTTGTTGTAATTAAGCATCAATCTTTGCCACCAATTCTTCGAATTTTGGATAACATGTAGATTACTTCACCGCATCTTATAATATTGAGTTCTTGAGACCACACAGCGCCGCCCATTCAATGCATATTTGATCATTATATCAATGTCACGAGGATTTTTTTTGTTTGGTGCTACAGTCATGTTCCCAGTTAGGACTTCCCCTTCACATATAGTTAGCACGTCTTCCAGATACAGAACTGTTTGCTTCCAGTGTGTAGCCCGTGATCTTGGTCCTGTAGAGAAGCCCATTAACTTGTGACAATTGGTAAATGACACATCAAAGTAGGCTACCAACGCATGAATGTAATCATCACGATCTGCCACAAGCTTAAAAGGAACTGTGAAGGAAGCATCCCCAGAGACCATTTTAGAGATATCCATAGTCTTGAGTAGCTGGCAGTTTGTGACAATTTGATTCTGATCAACTGTATCAACAAGAGGTTCCATAATGGCTTGCTTCTTGATACAACTCATGTNNNNNNNNNNNNNNNNNNNNNNNNNNNNNNNNNNNNNNNNNNNNNNNNNNNNNNNAATAAAGGGAAGCTTTGTCTGGTAGAAGAATTCCATCACTAACAAGCCATTTATCTCGTGCATAGAGGACAGTGTTTAACATATTTTCATACAACAGAAAATATCNNNNNNNNNNNNCATCCATTCTGAAATTATGATATCTACATTAGCGACTGGTAGCTCAATCTCTTCAACCTTT

>Unigene6762_All

CGTAGTTCAGATTCAAGTTGGTTCAGTGAGGGCGACCGCGAAAGTCACCGAATGGGTCAGTCTTTTCCTTTGTCCTATATATATTTAAAATCAAAAAAAGCGTGGGAGGACGTTGCAGATGTCCGGGACGGACACGACTTCTTCTAACGCTAGAAGACCACAGGAATATACCTGGGACCAGAGCATGTCGTGAAAGAAGCACACTGGGCGGGCGTGCTTCGACCGTGTCTCCGGGGCACAGTTGAATGTAGATATCATAAACGCGAGGTGCAGGATACCAGGCCGAGAAACCCGGGCAACCACCCCCTATGTGCCGATCGCTAGCGCATCCAGGGCCCCGGCGCCCAGCTCAAATGGAGAGGCCTAGCCTGATCTGAGAAGTGCTAATTCGGTTCGGATAGACTTCATTCAAGAACGCCGCCGCCCCAATAAGAAAACAAGTGCTAAGTTTCAGATCAGTGAATAAACCGAAACGAAAGAAGAGACGTTTCTCGCTCGGCGCCTAAAGCGCACTATGCTCCGCTTCAACAAATGAGAGTTTTCGGTGGAACCGGTGAACCACGCGAGCTGGTTAGATGCGTGGGACAGAGGGCTCATAGTACCTGCTAACGCGGCTATGCAGTGGAAGGGAAGGAGCCTAAATCGACCCCCTTCTTTCTTTTTTTTTTTCAAAGAAAAAAAGCAGTACAAAGAGATTAGACTCTCGGATGAGACTAAGCAGCCACCGACCGTTCCGNNNNNNNNNNNNNNNNNNNNNNNNNNNCTTGATTAAGACCGAAAACCTATCTAAAATGGAGCCTAGTTTAAGCTGTCAAACAAATGATAGAATGGAAAATGAAATGTTGAATAACCCACTAGTAGGGAAGAGATATGGAT

>Unigene6795_All

CAAAGCAAAGGATTCTGAAATGGTAAGATTATTCTCCATCATCCTTCTCTTATGCCGAAGCCAAGCAGCCTGTATGAAACAGGCTGCCCACGTCCTCCAATGATGGGAATGATACCTGAATGTATGCTGCAGCCTTTTGCTATGGAGACGTCTGAATTGGTTGGCAACAAACTTTAGATCTTCAGCTCTCAATGCGAAAGCTTCAACTTCATTTAGAGCTCTGACTGTCCTAGTTGAGGAAGGCAAGTTAAGAGACGATTTCGGATGCAATGCCCAAGCCAGTAGCTCCTCTCCGCAAAAATCCCCAGGTATCAAAGTAATTGAGTTGAAGAAACCAGTTCGGCCTCCATTGGTAGTAGAACTCTCCAGTCTGCCTCTGATAACAAATAGCATTTCTGTTACAGGGTCACCCTCACGTACAATATAGGTGCCTGCAGTGCTAAGAGAAGAAACCAGACGCTCACATATTGCATCAAGCAACTGATCATCCATCTGTGAGAAGAAAGGGACACGTAGAACAAGGTCCAAGCATAAGTGGCGCTGGATATCACGCCGAAGATCAGCAGGTAAACCACCCAGGATAGATTCTTCATGTACTCCTCGAGTTGCAAGCCACTTATATTGAACAAAACGTCTAACCTGCCTTTTCAGATCTTCAGGGAGTTGGCGATGCCTCATCCACTCCTCAGTATCTCTTCGCTTAAGCCTCCACTCCTCAAGTCTCACGGTAAGGGACTGAAGGTATGTCTGCATATTGCCAATCAAGTGGGCAAAGAGAACAAGACCCAAGATGGAAATGAGAATGGCAAATGATGTCTCACCAATATATGTGCTAGTCGACAAATTCTGCCCATATGAACTTAGTTGCTGTAAGCCCCACCACAAGCAGTAGAAATACTTTTCAATGAAGTTAGAGGAGACAACCTTTTTTGACACTGCATCTTCAAATATACCATAATCGAAATTATTATTGCTTGGATCACAGTTCTTGAAAACACTTGTGCTATTTGCCCATGTCTTGCGATCACCATGGTTAAAGGTATCACAATCTAAATAACTTAGGAAACATTTGATAGGATCATTTTCCTCTCTACAATTTGATTTCCGCAAGATTTATACCTATCAACAGATAGCAAATACCATGCTGCTCCTAAAACATGGCTTGCCAACATGTACAGTAGCAAATTATATACAGCCCCTGCCCAAGCTGTTCTTGTGACTACTCCAGCTGCTTTAATGATTTCAGAACTCAAAGGAAAAATTAGATATAATCTGGGAATATATTGTAGCAGAACAATTAATGCAAGTGCATTGTTCTTATGATCAGTTGTTCGACTTCTTGCTGCTGGTATGATAAACCAGATTACAATCTGAGGTAGAGGCAGTGTGGCAATAAGATCAATGAAGAAGTCAGATCTAATATATCTCCAAGCAATCTTCTTTGGANNNNNNNAAGTTCGCCCCTCCCAAAGACACGAGTGTTTGGAGCAACATAAGCAGTCCTGAACTTTATAATCATGTGCAACAAATAAAAAAGATCTGCAACAGTCCGAAAAAATGTTACAACTATTCGCAATTTTGAGTCACCATTGACACATAAAGATTCTTCATCCCCTCCAACAATAGGCAAGTAAAAGTACAGAGGATCAACAAAAAGTGCCAGCAAGCATGAGACAAGGAAAATCCTGTTCCATCGCAGCACAATATCGCTTCCTGGATCAAGCATCCTATGATGCCATGATGCATGGTCTGCTGGAAAAACCTTGGACCTCCCAACTTTGCTACTCTCTGTTAATCTATTTCTACCACCCATCATCCCATTGTCTGCTTTTAGCAATGACGACGAAACTTTGTCTGCAGAAGACATCTCAGGGTGTACAAGATCATTTTTTCCCCATGATGTTTCTCTATATTGTTTTCCATCAGAATAAAACCTTACAAGTTTACCCTTCTTCNNNNNNNNNNNNNNNNNNNNNNNNNNNNNTTGAAGCTATAAATCTGATCTTTCCAAGACCATAACAGAACCAAAAAAAGCTGTTAAAAATGTGAGTTGAATTACAGTGTAAGATTCAGAGTTGAGAGTGAGGAATCAGG

>Unigene6828_All

TATAACAATTTGATGGCCAAGTAATTTGGTTTTTGGCTTGATTCTTCTACTCTCACACCCAAATTCTGGTTTCTATATTCACAAGGCACCCTTTTATATTCCACATCGACAATCCCAAGTCCTAGAATGTCTTTGCCCATTCCCTCGTTGGCCATGGCCATGAAAGCTCTGCTACTCAGAACAAATTCTGTCTGGTTATTGTGATTGAGATCAGTCATGATCACTGTAGTTCCTTTACTGCTACAAAGGGTATTGTCTTTGCATCTTATCTGAAAACAGGCACCGCAACCAGCCCCATCTTTGTAAAGAGTAGGAATACCAGCTGCAAGGTGTCCACTGTTGAAACCTGGTGCCAAGGAACCGTATCCACAAGCCCCAGAATTAAGTGCAGAAGCTTTGGAGAAATATGCAGCCTTAGTTTGGTGCACACAGCGATCACAAGCAGTTACAGATGAGATAAGAAAANNNNNNNNNNNNNNNNNNNNNNNNNNNNNNNNNNNNNNTGAAAATGTCAAATGAGAAAAATCAATGTGGTATAGAGAGAATGGTGAAGAAGAAGCGGAT

>Unigene6845_All

CTCGGAACAGAACCATCAATATTGCACCAGAAACACAGACAAGGGTGCCCCCAACCTTCACTTGCCCTTCAAGTCTTAACAAATTCACTCTTTCTGTACCCATCATCACAGCCAAGACAAAAGTAAAAACAGGAATTGAAGGCTGTATTGCAGCAGCGTAAGTTGGATTTGTGTAGCTAAGGCCAAGAAGAAATAACAGCTGGTTGCCAAATATCCCTGTTAACCCAAGGAAGAAGAATGACAAGAGTAGGCGTTTAGTCATAGGAGGTCGAATCCTTTTTTCACGGATATAGGCCACGGGGGCGAGGATTAAGAGAGCTAGCAGGTCACGAAAGACACAGAAAACGAGCTGATTGACCCCATCATTAAGAGCCACTTTGGTAATAACGTGATATCCTCCATTGAATAGCTGCACCATGGCGACATGAGCCTTCCCAGCCTCTTTATCCAGAGCTGCTCCTCCACCACCACCTCCAGCAGATGCTGCCATCCCTTTCTTTCTCTCCAAAGACTGTATTACTAGATATTGGGAGAGTAAAGAAAGAAAATTTATATACNNNNNNNNNNNNNNNNNNNNNNNNNNNNNNNNNNNNNNNNGAAGAACCAACCAACCAAAGCCCAAATAGAAGGTGAGTTTGGTGTTGGGATCGCATCAAAAACAGTAACAAATGGAAATATCCATCTGGGTATCCTTAAT

>Unigene6851_All

GATAGCCAGTACCAGAAGTTAGGGTTTTGCGAGTGTATAGCGGTAGCCATGGCGCAAGATAGTAACAAAAGGAAGAAGCCAAATATCCTGATAACAGGAACTCCCGGAACAGGCAAAACGACGACATCGTCTGCTCTGGCGGAGGCTTCGCAGCTCCGCCACATAAATATCGGAGATTTGGTCAAAGAGAAGAACTTGCACGACGGCTGGGACGAGCAATTCGAATGTCATATCATCAATGAAGACCTGGTATGTGATGAGCTTGAGGATATAATGGAAGAAGGGGGAAATATAGTTGACTACCATGGCTGTGATTTCTTTCCACAACGGTGGTTTGATCGTGTAGTGGTGCTTCAAACAGACAATTCTGTCCTGTATGATCGCCTCAATAAGAGGGGGTATTCACAGAACAAGATTTCCAACAACATTGAGTGTGAAATTTTCCAAGTTCTGTTAGAGGAGGCAAAAGAAAGTTATGCAGAAGATATTGTAGTGGCATTAAGGAGTGATTCTATTGACGACATAATTAGAAATGCGTCTAATTTGACAGACTGGGTGAGGGGTTGGCAACCTGTATCATAATTGGATTGATACTAATGTGATACTGTTCTTTCATGCTCCTTTCGCAGGGATGGAGCAGTTTATGTTATTAATTCTTGCTTGAGAAGGTTTTTGATATATGTATTTTGCTTTTCTAGTGCTGTTGCGAAGTAATTTGAGTCTAGTTCTGGNNNNNNNNNNNNNNNNNNNNAATGTACGTCAAGTCTTTGAATATCTACTAGCTCGTTTAGCTGAATTATAATTTGCTTTGGCCATTTGCTTTGTTATGGAATGGATTATGTGGTTGAG

>Unigene6884_All

GAGAGCATTTTCTCTTGAGTTGCGGAAAATGTTTAGGAGGATCTGAGGGAAAAGGAAACCATCCAGTACCAAGCCAGCATAAGACCTTATGTTCGGCCAAAGGGAATGCTGGTTTCTGTAAAAGCTTGGAGAATCCATCTCCTTGCCTAGTTTGCAATTCCAACAATTCATGAACAAGGCAATTAGGCCCCCAGCTATATATAATGGCAAAGATACATAGAGTGCCTTCTTCTCAGCAACCCATGAGGCCTTTTGGTTTCCATCAGCCAATCGTGCAGACCATACGAGTTGCAGAAACCGGAATTGCAGCATAAAAGCTACCATTGTTACCACCTTTACGATCACCTCATTCACTTCAACCCATCCACCACCTCTGAGCAGGAAAGATTGAGTACTTTGCTTTGGCAAGAACAGAGCTTCAAAGTTGAGCACTAGAGGGATCATGCGCCCTACTGTAAGTACTACAAGCATGAGAAGTGAGATAAAAGGGAATACATTAGGGTGTTTCTTCACATAGAAGATTTGATATCCCACAAAGACACATATAAGGGTATTAGAAATCAAAGCCATGATAATCTCTAAATCCATCCTTCGTATCNCGCGACTGTAATAAGGAACTGCAGAAAAGGAAAGAGGTTCAAAGTATAGAGGAGATGAACTTTCTCGTGTGCTTTCAATGCGTCCCTTGATATATTGGTTTGAATCAAATGGAGGCAACTGGACGTTGACAAGAATCTCACAGTCCATCGAATCATTTGCTAATATCTGGTTGTTTGAGTCAAGATATTTGCAGCCCACCATAGATAGAACTCCCGTTTCACCATCGTAAATCCCTTCAGCAGAAATTTGGACATACGTATGCTCCTTTGAAGAAGCATTGAATGATGTCAAGCTCATTTGGTAACTGATGTTCAGTGGCTGGAGTGGCTTTGCTTTATCCTTTGCCTTTTCTGTTCCTGGACTTGAATATGGCCTGAAATATTGGTTCCTTGGAGCAATTTTATCACCAATAGAGATAGGATTCGCATGGCCCCATCCAAATCTTTTTCCTCTGGAATTCTTGATTGACATTTCAAATTGCATGTCATCTGAATTCCCGTCTGAATACTGCCTTCCTTCACTTCTACCGGGCTCCTCCTTGTATGACTTTCTTGCTTTGTCCACCAGTGAATACTCATACTTCAACNNNNNNNNNNNNNNNNNNNNNNNNNNNNNNNNNNNNNNNGTAACCTGAAACCCCCACAGCCTTATTGCTTGAAATATGTCCTGCAATGCTACTAGCATTTCTGATTGACCAAACAGAAGGGAATCTCAAACTCAACCTAATTGAACAATCCTCAAC

>Unigene6910_All

CCTGTTTCTTTGGCTTCCTACCACGCTTCTTCACCAACATACTACCTCCGTCCTTACCAGAATCAAAATTTTCTACTGCTTTCTCACTACGTTCTTTCCCTTTCTGATCAGTTTCCACTTCCCCAATCTTAGTACGTAATTTATTATCTATAAACCCACCTTCCATTTCGTCCACATAACCACAAATTTCCGTCCCTCTCATTTCAATTCCTTCCAGTTTCTCGCCGTTTCGGCCTCCAAATTCGGCTTCTTGGTTTTCCAAAACATTCTCCTTTTCCTGTGGCAGAACATCGCCGCCTCCATCTTGCTCCTTGGACCCACAAGCTTCCGGGTGATTGCCTTCGATTTCAGGCAATTCCGTCGATACATCGAGCGACTTTATACCATGTGTCATGGCCAAAGGACCGAGAACGAGAGATCGATCAGTGCAGATAGCACAGAGAACGTACTTAATTAGCAATAGGGACAGAATTTGGGGAAGAAAAATTAAAAATATGGAACAAGAAAGACGAGATGGTTTTATAGGGCAAAGTGAGGCAAGAATGAAGAGGGTTTTGGATTTGGGAAAACAAAAACAGTGGGTGGGAATTTCTCGTGTGGTTTTGAAATCTGGCGCCAAATTGTGGTTTTAGGGTTTCTTCTTCNNNNNNNNNNNNNNNNNNNNTTTGTCATATTTTTGAAGTCAGATTGCTTAGGAACACCTATTAGCCAAACTTCTTGTCACG

>Unigene6923_All

GCAAGGATATGACAAATGAAAGAAGCTGCAATGAATGCTAATAAAGATGCTACAAAGAATGTTGATGCCATGGGCCACAATGGCTCTTGTAACTTTAATTTCATGTTTTTCAACAAATAAAGCAACTGCTAGTCTAGCGATTGCAAAACCTAACTGCCCCAATATATGTGGGAATGTGAGTGTTCCTTACCCTTTCGGGATTGGTGATCCAAATTGTGCATTTAGCTCCGACTTTTTACTCCACTGCAACAATACTAGTGGTTTTGCATATCTATATCTGGGTAATATACCCATTCTCAAATTCTTTCAAGATGAAGCTGCAATGATCATTAGTGTAGAATCAGGATATGACTGTTATAGCTATGGCGAGCTGGTTTCTTCCCATGGTAACATTATCACATTAGGGTCACACAACCTCTTCACGTTTTCATACACACGAAACAAGCTTGTGGGTCTTGGTTGTGATACTTTAGCTCTTATTTCAGAGTTAAATAGTGATCGTGCTACTAGAATCAGTTGTACCTCATTTTGTGATGGAGCAATGAACACAGCGAAGGATGTTAATTCTTGCTCTGGAGCTGGATGTTGCCAAACCTCAATTCCAGATGGACTTCAAAGTTTTGAAATCGGAATTGGGAGTATGTCAAATTATAGTAAGAAGTTGAGCAGCATTTGCAACTATGCCTTGTTGATTGATCACCTAGATCCAAGTTTGGGTAGCATTTTAAAAACCAATCTCAATCATCACCCAGCCAACACTACCATCAAAACGCATGTAGTGGTTGACTGGGTCATAGACAACAAGACATGTGTAGCTGCTAGTACAGAGTCATCTCTTTATGCTTGTGGCCTTAACACTTTATGCATTGACTATAACTCTGGATATCGTTGCGTATGCAAAGAAGGGTTCCAAGGGAACCCTTATCTTCAAGGGTGTGAAGATATTAATGAGTGTCTTTCTCCGCAAATTTATCCATGTGATGGGTCTTGTACCAACACAGAGGGAAATTACGCTTGTCGTTGCCCACCATTCATGCACGGAGATGGTAAACTGAGTTGCAAAGGAATTCGCATAGCCACCCTGGCTGCTGGTATAAGCTCAGGCTTTGGATTGATATTGTTATTTGTTGCATTCTACGTTCTGCACAAATATATGAGGAAAAAGCGTCAAATTAGATGTAGAAAGATAATTTTTAAGAAAAATGGTGNNNNNNNNNNNNNNNNNNNNNNNNNNNNNNNNNNNNNNNNNNNNNNNNGACAGGGCAAGGATTTTCACTGCAGAAGAATTGGAGAAAGCCACCGACTCATACAATGAGAACCGAATTCTAGGGCGAGGAGGACAGGGCG

>Unigene6943_All

GTTGTGTGCCCATCTTGCTCATCGGCTTTCTTTCTGGAAGAAGAAACAGAATGAGTGTTGCAGTGGAATTGTTAGCTACTCGAAACCTTAGCTATTTGCAGTTAAAGAGAGCTTCTTTATCTTCTTCAAAGTCCTTGTGTCCTTCTTCGTCTCTAAGGCTCGAACGTTTGCTTAAACCTAGGGGTTACAGAGTTGCGATTGAAGTTCCTGCTGTTTTTACTCCTAAAGGCCTAGGGATTGGGATTGGTTTTCCTTCTCTCCAGCGAAGGAATTCGTTGAACCGATCGATTGTGTCATTTGCTGCCTCCCACGAAGAATCGAAGCATTCAGAAATTGAAGTGGACAAGGAAAACTATGGTGATGAGTTGGGAGCCGAAGAATCACAAGAAGTTTGGAAAGAGACGCTAGCTTCTTTCAAAGAACAAGCCTTGAAGATGCAAAGTGTGTCTCAAGAAGCATACGACGTATACTCCAAGAAAGCAATGGTTATTTTGAAAGAAACATCGGAGCAGTTGAAAATTAAAGCAGACAAGGCTAAAAGTGATCTGGCCGTGCTAGCTAAGGAGGTGACCGAAGAGAGTAAAGAATTCCTATCAATAGCTGCTCAGAATTCCCCTGAACCAGTGAAGGAGGTTGTGGAAACNNNNNNNNNNNNNNNNNNNNNNNNTTTTCTGGAATTCATGACTTCCATCTTGGAATACCTTATGGTTTGCTTCTTTCTGTTGGTGGCTTTCTTTCCTTTATGTTGATGGGCAGCATTTCTGCTGTTAGGTTTGGTGTCATTCTTGGTGGTGCTCTTTTGACTTTAAGCATATTGAGCTTGAGATCATACAAAAAGGGACAATCATGTACTTCAGTTGTGAAAGGACAGTCAGGAATAGCTGCCCTAATCTTTCTTAGGGAGATAAGATTGCTGTTTCAGAAAGCTTCTATTTTGACCTTTTTCTCAACCGTCATCAGTGGCGCAATGGTTGCATTCTTTCTTTATAGAATTAATGTGAATGGTAAGCAGAACGAAGGATCAGACATGGGACAAGGAGCTGAAAATTGATGAATTTGTTGCAGGTTGGTTGGATTGACATGTACCCGTAGAGATATGAACACAAAATATGAAGGCTTCTAGATTATGTTAGCTGTGAAGAACAGAGTGAAGTTTCAGAGGTGATGGTTTGTTTGTGGAGCATTAGAGTACAGAAATGGGTTTTCAAGTACGATGATATGGAA

>Unigene6954_All

CAAAACTAGTTCAGAGAGCTTCCAGGTTAATCAGTTATCATCTGTTGGATGGCAGTGGGAAATTTCACTGCTTCAAGCTGTTGATGCCATCTTTCCTTCACAATCTCTAATTGCTGGTCAAGCATTAACCTGTTTCTTCATGCTTAAGAGTCTTAGGAAATCATCAAGGACTGAAGAAAAAATTTCCTCTCTTTCCCGTTCCCCTGGAAGTGATGTAAGGTTGGCTCCTGAGGGCAACAAAGATGCACTTTTTGATATATGTTGCTCACCTTTGGCTGATTTCCATGATTGTGAAAGATTGCAGCAGCTTGAATTGTCAAATCAGGAAGACACAAATACAGTGGACTTCATTTTGATCTCTAGGCCACTGAAAAGTGATATCAACCCTGGGACGTCTGATGCACCTCGTCTCTTTTCTCATCATGCATGTCATTGCAGTACTGCCAGCGCAAGTCCAATATCATGGGTTGTGGCTGGGCCACGGACCAAATGTCATGACTTCTCTGTTTCATTTTGTGAAATAAACTTGAGAATGATTATTTACAACGCGTCAGATGCTGTTGCATCTGTGGGCATTAATACCCATGATTCCCCTGGTGGCAATGTTCAATTAAGTGATGGAAACACAGTTGCTTCAGGAAACCAAGCAGGGTGGCACAGTCTATCTCCAGTGGACGATATTAAAATCAATTCTGATGTTCTTGGAAGCAATGTTGGAAAGTCACAATCACCAGAAAGCGTCTCTCCATTCATTTGGTCTGGCACAAGCTCTACCAGAGTCCAACTTGAGCCGATGTCCTCGACTGAAATTCCTCTTCAGATTTGCGTATTCTCCCCTGGTACATATGATTTGTCGAACTATGTTTTGAATTGGAATCTTCTACGTGTGAACAACGAAGGAAATGTAGGAAAAACAAAACAGACCTCTGGAACAAGCCCAGGGTATCCTTATTATCTTACTGTCCTCCCATCAGATTGAGATCTCGGATTGAATTTTCGGCTTCCTTTCTTCTGTGACAGATAGAGGATCTTCAATGTACTAATTTTTGTAACATTGGCTTGTAAATTATTATTCCAATGTATTTGATTTCTTTTTCTTTCACAAAAANNNNNNNNNNNNNNNNNNNNNNNNNTAAAACAGGAAACAAACTAAATTCTTTTTATTCCCACTATTATTCTGAGGGACAAAACAATGTAGTTT

>Unigene7021_All

CGCAAACATGCGACCAAAGAATGAACCAATCAACCAACCAACCATAATATCCAAATGATCCATCATCATACCCTCCTTCCCTTTCCTTCCTCTCTTTCTCTGACTATCCCTGCACTTTCCCCCAATCCAAACAAGGTACAACACTAAACCCCCTTGCCGATTTCATTCAACTACTCAGTCACCGACGCACCCCACCTGACCTTCTCATGGAAGAGAAGCGAATCGGCGAGGAACTCAGTTACCCGATCCTACTCGCCGAGCGAGTCCGCTCCGCTGTTGAGGAGGCTGATTCCTTCAAGTTAGAGTGTAGTACCATCGGCAATTACACCGATATGCTCGCTGAGAAGCTGCGGTTACTTGTCCGCTTCACCTGTTCTGCTCAATCGTTCTATGAGCGGCCTGTTCGTCGGATTATCGCCGAGGTTTTCAAGAATCTCGACCGTGCCCTAACCCTAGTACGCAAGTGTAAGCGCCGGAATGTGCTGCGCCGGGTTGTTACGATCATCAGCGCCACTGATTTCAGGAAAGTGCAAAATCTCCTTGAAGCTTCTGTTGGTGACGTGAAATGGCTCGAGAGTATCTTAGGCTTCGGAAATGGCGCTGCGGGTGAGGATGTTGGTATTGATCTTACTCTTCCGCCAATTGCAAGCAACGATCCAATTCTCGCTTGGGTTTGGTCCTCTATCGCTTCTATTNNNNNNNNNNNNNNNNNNNNNNNNNNTGAAAAAATTGAAGCTGCTAATCAACTAGCCCAGCTTGCCCAAGATAATGATCGAAACAA

>Unigene7042_All

TCAATTAAAATTTTGAGCTACCTTATCATTTAAAAAATAAAAATAAAACAAAATAAAATAAAAAAAAACAAGCTGTTCGCTAGAGTGATAAAGATGAACTATATACAACATTTACAAAATGTCATCCACCAGTTTCCTCACGCACGACTCACCCAAACTTTGCCAGCTGAAAATCCCATCCCAGAAAGGATCACAATGAAAACAGACACGGAAGGACAGAAAAAACCATTAGAGAAAATGAGGACATAGGGAAATACTGGCTTTTTATCTGCTACTTGATTTAGAAAAGAACAAAAAGCTGTACAGTTCAAGCAGCAGGTCATCGTATACAGATGTGATTGTTGCATGAAGGACATCGCTTATTTAGTGAAACATGATTGCAAATGGGGCTTCCAATGCAATAGGAACTGTACAACCATTGCACACCAGACAAAACCATGAGACCAGGCCTTTCTAACTCATGGAGAGAAGACTCCAAAAACGCCCGCAAAAATTCCATCATGTAAAGATATTAGGCTAAGACTCTTTCTTTTTCCTACCCAACCAAATGCAGGGAGGTGACACCGTATAGTTCAATTAGGAGTGCCCTACAGTGAAGCCAGCAGTGATGGAGCCAAAACATAGTTCAGGAGTCCAAAGTCCAATGCCCCACACCCAACTAAAAATGAAGAACACCTTCCAAACTAGACTGAGAATATAGATCCAAAGGTAGTTTGTCCAAGGACTTCAGAACTTCGATCAAGGCCGTGAGAAGCAACCATGTATATTAGAGCAGCATAACGCTCCTCATTCAGATACAGTGGCTTTCCTCGTTGCATTTCGATATCCTCTTCACCAAATGCATCCAAGTAAGGAGATGGCCATAGAGCCAAACGTGCACATCTTTGTAGCAAAATTGTAGTTCTCTTGATCAACAAAAATACACCCCAGCCCCGAAAGCCACTGCATGAGGTTCGCATCCGCTTTCCCTGCAGCACGGCTTCCAGATCGGGGAGCATAATCTACCACATAACAGGCATAGAGCAGGTTCATCAATCACACTCTTGCAGTCAGCACAACGTTGTTTTATATACCTCTCAACTAGATCTTGGTATACATGAGGGAGCTGCATCAATTCGCAGGTGTGGAATGCAAAACGGACTGAAAAACAAAGACTTCGTATTCCTTGTGAAAGTGATGTAACCATTTCATAACTAAAGANNNNNNNNNNNNNNNNTTCCTTGGTCCTTGACAACAACATCCAGTGGTGGAATCTTGAAAATCTTTTCTAGTTTCTGAACTTCATTGAGCTCAACTATACCACCATTCATGTAGTCCATTGTGTCATCATCAATGGCATGTAGCACATCATCCCTATTACAGAAGGGTTCTGAGAAAGAAGTGCTTAATAATTCCCAGAGCAACACACACCTCCTCAAGTAAGGGAAGCTCAAT

>Unigene7076_All

TGAATGTGCCAAGAAGACATTCATCTCCAATTGTCTCAAGGGGAAGACTAACAGAGACCCCTGGAAAGGGTCGTGTGCATTCAAATGGGCATGTTACTGATATGTCTGAACCTCGAAAAGTGTTACATGTGTCAGATTTAGCAATGCGGAAATCTGTTAAGTCATCAACAACTGCCGCAGAGAGTACTGGCTTTGGGAGGACAATATCTAAGAAGTCTTTGGATATGGCGATCAGGCATATGGACATAAAAAATGGCACAGGCAGCACTCGTGCACTTTCAGGTACCACTCTTTTCCCCCAGAGCATTCGATCTGCTTCCAGAACCCAATCTGTCCGTTCTATGAGTGCTCCAGAATCCATTAACAATGGAGATATTTTTGAGAATGGAAACCATATGAGCAGGCCNNNNNNNNNNNNNNNNNNNNNNNNNNNNCAAAGTTGAGTGAAGTTGACAACTACGAAAGCTCCCGATATGATACAATATTACTTGAGGA

>Unigene7097_All

CATCAACACTCTCTCATCTTTTCACTTTCTCTCTCTACTAGCAAAATCACTCTCTGGAGATGCAAGGACTGCGACGTTGCTCCAACGACGCCGTCCACCTCGACCTCACCCCTCCTTCTCCTCCTCCTCCTCCTCCTCCTCCCCCAACCTCTTCCACTTCTTTATCCTTTGACGCTGTCGAATCTGCTGAGACTCGAATCCAACGCCTTATATCAGAGCATCCGGTCATCATATTCTCTCGATCTTCGTGCTGCATGTGCCACGTCATGAAGAAGTTGCTCGCCACCATCGGTGTCCACCCTACTGTCATCGAGTTAGATGACCATGAGATCTCTGCTCTCCCTCTTCCACCTCCCTCTCACGATAACGATGATGCCCCTCGCAATCTTGCTCCCGCCCTCTTCATTGGTGGAACTTGCGTTGGCGGCCTCGAGTCCCTCGTCGCTCTCCACCTCAGCGGCCACCTCGTCCCAAAGCTCGTCGAAGTTGGAGCTCTGTGGGTATGATGAATATTAATTACAAAGCNNNNNNNNNNNNNNNNNCATGTTAGATTTGGGTTTCTCTATGACTGTTTACTGTTAGTGGACATAAAATTAGCTAGGG

>Unigene7109_All

CTTGCAGTAGGAGCACATTAATTTAACCTTCTCGTGTGCATCATCAGGTAGTGTCTCTTTAAATTTCCACATATTAAAAGCCTCCATTATATCCTCTCTGTTTTGCTTGTCCATTGCAAGAATGATATCGAAATCCCTAAAATCAGAGGGTCGTATTGGCCTCGATATGGATGTTATCTGAATCCCGCGCCTTTTAGAGGCTGCCCTCATTCTTGGGTCTGCTGGATTACCCTCGTGATAACCAATGGTGCCAGCAGAGTCAATCTTGAACTTGGAGTCAAGGCCTCTCTTCTTGACAATTTCTGTGAAAACACCCTCAGCAGCTGGGCTTCTACAAATGTTACCTAAGCACACAAACAGAACAGAGAAGGGTTTGGTCTCTGAGTCGGTGGAGGAAGCCATTGATGAAGCTTTGATCACCCAGGACCTCGAGGAAGTGACTCTGATCGTGGTTAATTTTTTGTGATGATGGGAATTAAGAAAGCAGTTTTGGACAAGTGGGTAGCTAATAAATGGGATTTTCAGAGAGAGGAGACCGAGTCTGGTGGTGAAGTGGCAAAGTTGTAAATTTGGAGATGGGGTTGGCAACCTTGTATAAGCACCATGCAAGGATGATGATGATGCTGCTCTCATCATCCCTTCTGTCAGGGTGGAGGTTTGGGGTTTTGGTTTGTGTTGTCGGATTTGTCTAAAACTGGAAGTACAGCATTCTTTCTGGGGGAACTTAACTGGTAACAGAATGGATAGGGTGGCATGTGAGTCGGGTGNNNNNNNNNNNNNNNCCGTTTTGTATCATCAATTTTCACATCATAAAATTAAGAGTTTAACCAATGAAAGCTTAGAAGAGCTCTATGATACATGTAAAGTCTGTTGTGTTTAAAAA

>Unigene7217_All

CTGAAATATATAGAAGATAGAATTGGCAGGAGAAAGCAAAATAAGAGACTAATATGTCAAATCATGTACATATATATTCATGTAAAAATAAAAACCTGAGAAATCAACCAACAAATTTGCATTTAGGTAAATCTATCGGAACAACCTTGGTACAGATCTCTTTGGCACTTTGCCTTCAAGCTTTAGTCTTCTATATGCTTCTCTTATGTGGCAAGGTCTGATTGGCCCAGATTCCTTCCTCTCTGTCATTACCATTCTGGCTGTTTCAACAAGTTCACCAACGAACATTTTTGCTATTCCACAAACTACAATTGTCATTGGTAAAGAAATTTTCTGGGATCCAGTGATGCTTACCAACAACCTTCTCATGTTGGCCTTCTGAAGAGCAGATCTGCGAAATGATTCATACCTGCTCATCTGTTCGTCAGTGAATTGCGATAAAATAGCCTGCATCTTGGCCANNNNNNNNNNNNNNNNNNNNNNNNNNNNNNNNNNNNNNNNNNNNCTCAACCTCCATATTATCCTCCTCTTCATCATCCTCGTCGTCTTTGTTTTTGGGCTTG

>Unigene7218_All

AAGTAATCCTGCCAATCGAGCAATCCATCCCCCATCGTGCACGGTAGAAGCCTGTGGATTGTGATGCTCAACGACAAGCTGATTATGCTCAGAAGTTTGAAGACCTTCACTTTGTGCAGAGCATAGCATTTCTTCGTTAGGATGAAGCACTGGAGCACTCCCTGCACTACTGGATCTAGCGTGTACTTGCTTTCTATTTCTAAGGCTACTAGCTTGCACAAGCTCAACATCATTACTCTTCCCAGTTGGAATGTTAAGCTTAGCTTCATCTTCCACAGACACTTTCAAGCCAGAATCAGAACCCAACTTAGACGCGAGGACAGTGGCAGCAGCTGCCTTTGCTGCTGGATCAGGATCATATCTCTGAATGAGTTGCTGCGTTGTGTAGTAATTTGTCTTCTCTTTAAGCTCATCAATTTTTGCTTGCCTCTCAGCTCGAAGCCTTTCTAGAGTTTTCTGATCCTTGCGATCACAAATCCTTGTGAAGCTCACAAATGCTGAATAAGCAAGGGACGATAAACCAGGCAAAAGAAACATTGGCAAAACTCGAAATGCCCTCATCTTCCAATTCAAATCCATTGATCTAGTCGTCATTATAGCATAACCCACTGCAATTACCTCAAAAATGATGGAGAAAATAATGAGANNNNNNNNNNNNNNNNNNNNNNNNNNNNNNNNNNNNNNNNNCTAGATAAAACAGCAGCTTCTTCCTTGGAAATGTTTTGCAGTCTCTTTTCAAAATCGTCCCCATGGAATCTAAAAATGCCATTCCAGATACGAGACATAATACCCTTGCGCTTCTTTTTATCAATTAATCTTTGTTCATTCTTTTCACCTTCTGCTATGCCTTTGTCCTCTGCCATTTCTTTCACTCGTTTCTTGTTCTTGTACTACTGCTGCTACCCGAAAGAAACGGATGGACAGTCGATCTATCAAAACGTTCGGGTAGTAGCTGGCTGAAGCTTCTTTAGTCCTCTTTTC

>Unigene7220_All

TGCTCTTTTTGATTTAAAAAAGAGCAGAACTTAAGTTTGACTGACGAAATTGATAAACGAGTTAAATAATGTTGATGAAACCCAGCTATGATAAAATCTTTCCAACAAGTTATATGTTCTGTATGCAATTTTCAAGGTGTCTCAGAAAAATACAGAGAGAACCCTTTATCCTGAGTTTGTATTGTCATATCTTGTAAACGGGTACAATTCCAAGAAGGAAAAAGCACTTCCTCATGACCACGGCTGTTGCTTCACACAGCAAGAGTCTACTCGTTTCCTCCGCTGACCCAACAACCTGACAATTGGAATAAAATTTGGTGAAGTATTCAGATAAATTATAGAGATATTCACACAACACGTTTGGCAGGAGATTGGTGCAGGCCTCCTCAACAGTCTCAGCAAACTGTAGTAAATGAAGCCCCAGTGCACGCTCATCAGGATGAGCCAAAACCAAAACTCCAATTTTCTTCAATTCCTCTATGTCTTTACCAGATTTCCTGATGATTGAACAGATCCGAGCATGTGCATATAGCAAATAAACAGCAGTATTTCCCTTATCATTAAGCATCTGATCATAGTTGAATGTATAATTGGTCAATCTGTTGTTTTTCAAGTCAGCATACTTGATAGCACCATAACCAATTGCCTCAGCTGTTTGCTCAAGCTCCTCTTCAGTCCACTCTTCAGCCTTGCCACGCTCAACAAGTGCTGCTTTGCTACGAGTCTTTGCTTCATCTAGCAAATCAACTAACCGAACCACTTCAGTAGCTCGAGTTCTAAAACGCTTGCCATCATCTCCAAGAACAAGGCCAAATCCAACATGGCTAGCTTTAGGAAACATACTATCACCAGCTGGGAGCCAACCCGCACGTTTGGCAGCTTTGAATACCATATCAAAGTGCTGCTNNNNNNNNNNNNNNNTAACATATATAATCCACTCAGCTTTATCTTCATTAAGGCGATACCAAAGGGCAGCTAGATCAGTAGAAGC

>Unigene7230_All

GCCAGTTACGAACTCGCGTTCATCAAACTTCAAAAACTGAATTTTAACTATGAAATAAGAATTAAAAAAAATAAAAGAAATTAACCATCAATTAAATTACACTTTGTGCTCGAGGCTTCCCTTATTTTTATAGAAATGCTCCAATACTACACAACGAGCATGTTGCAAAGATAAAGGCTAGTCAAGTATCTGGCTCAGAATGTTTTCTCAACACTTGGTTNTGAACCTAAGCAGGGTTCTTGGTTGGTCACTTTGTGGAGTATGTGGTGGGCCTTCCAACTTATCAATTCCATCTTGCACTATGACATCAGCTGTTGGCTCACGCATGGTTTGATATAAATTGTCTGCCAGCTCCTTAAGACCACTCAAACTTGCTTCAGCATATGTGATAGAACGAGTTCTCCCAGAAGGTTTAAGTTGATCCTCCAGAGCTTTAGGCATTTTCATTTTGTTACCGCCAACAAAATTGCACAGGTACAACACACCCATATTTGCAAGATCCTGCTTCTCCTCCAAAGAACCTATCTNNNNNNNNNNNNNNNNNAAACCTGAAACTAGACACTGCAATTGCCACAATTCTGAGCCAATTTGTGATATATTTTCAGAAGCAGCATTAACATCATTTTGAATTGCCTTTGAGATCTCTTTTTGGCTCTCCATTTTATCATCCAAAAGCTGTATTCGCTGAGTCAGATGTACTTTTGCAGCAGATAGAGCCTCGGTTACTTGCTC

>Unigene7231_All

ATGCATTTACAGATTTTGTTTTCCAGGCAGCAGTTCCAAAGTTTCTTCAGCTGCACTTAGATCCAGCTAGCAGCAATACGCTACCTGCAAGTGGTAATGGTTCGGTTATGCAAAGCTTGAGAGTAACTAACAGCCAACATGGAAAGAAATCACTTGTGATGCGCATAAGGATAGCTTACAAGCTGAACAACAAAGATATGTTGGAAGAAGGACAAATCAACAATTTTCCTCGGGATTTGTGAAGTTAATTGGTCCAGTTAGTGGTAATGGTGCCTTATTGCCATGCATGAGCTCCAATTTTTTTTTGTCCTCTGAGAAGCACCTTACTCTTTGTAGTAATAGTAAAATATATTGTTTGTATTCTTCTACTCGAGGGCAGTATTTTAGCAGTTGATATTTTTTTTTTTTTTTTTTTTTGGGTTTTTTTCACAAGATTTTCCCCTTAAGTTTTTTGTGTACATTTTTAAAAANNNNNNNNNNNNNNNNNNNTGTTGAATTTTGCCTTTTTGAGGCTCATAGAGAAAGGTCATCGTCCCATAATGGAATCAGC

>Unigene7329_All

GGTTGAACCACGCTTCCTTCGAGAACTAAACCACCTAATCTGAATTTGCTCTTTTGTGGGTTCGCTTTGTATTTTTCCATGGCATCTGTTGGGGTCACGTAAAACACTAATTGATCTTGAAAGTTGTTTAAAACGATAACGATAAAACCCGCGATGCAACTGAACGTCAAAGCATAGGTCCAGAGGCGGCGTGTTTGGAGTTGACGGGCTCGGGCCCCTATGTCCACTGTCTTCGAGCGGGCAGGGTGGCGTCGGGGGGTGGAGAAGAATCGGAGACCGGTGAGCGAGGGAAATTCCATAGAGATTGTACGGAGATCTGAGAGAGGGGAGAGAATCGAGCATTCAGATTTCATGGTAGAAAGGAATGNNNNNNNNNNNNNNNNNNNNNNNNNNNNNNATAGTCGTGGTGTTGGTGCGGAGGCGGTGGAATCTGAGGCGAAAGAGAGAGGAGCGAAAAGCC

>Unigene7386_All

CTTAATGGTGTTAATGGTGTTCATTGGTCGCTAATCATCCATTAGACCTTCTCTGCTACTGCTAAACACACAACACAAACAATAATTAATTAAGTACTGCAGTAATTAATTAAGCAACCCTAAACAATAATTCTAATCTATATAGTCACCAGTCACCGCCAAAGAACAGAGCAGAACAGATTCTTCTCCTTCTCCTTGTTGATTCTTTCTCATTCTTGATCAGAAATTCAGAATTCATCAATTAGCAAGGATTCTCATTAGAAGCGATGAAAAACTGGGAATCAAGCTTGGCCCTGCAAACCGGACACACAGAATGCTTTGAGAGCCAAGTGTCAGCGCATTCCAAATGGAACCCATGATTGCAACCGGGAACCAGTCTCACGGGTTGCTCGCTCTNNNNNNNNNNNNNNNNNNNNNNNNNNNNNNNCCGCCAACCAAAGCATGCTCCTCTCTGTATTTTAGGCAACATCTCAAGCTCAGATGCTGACAATCCCTTCTCCGCCGGCNNNNNNNNNNNNNNNNNNNNNNNNNNNNNNNNNNNNNNNNNNNNGCGTACCAGAGGAGACAAATATAAACAAGAAAAACAGCGCTCATGCCGACGCAGGGGAGAAAAAGGGCCAAAAAGACAGAGAGGAGCATGCTTTGGTTGGCGGAGGCGAAGTGATTGTTGACGGGGGTTGGAGGCGGAGAGGGAGAGAGGAAAAGGGGTGCGTAGCGCACGCCATCGCGCATTATAGGGATGGAGAAGGAAAGTGAGAAAATAAATGGGCGAAGTCGAGAAGATGGAGCGTGGTGGGTTGGCTTGGCGTTGGCGTTGGCGTTGGCTTTTCAGGTATATAGTGTAAGCGGGTAGATG

>Unigene7397_All

TGAACAATCATTCTGAGTTATTTTAAATATAACCATGTAACACGAGAATAATTATCTACAAAAGTAACAAAATATCTAAATCCAAATTTAGAAGTAATAGAACAAGACGAAGCCCGCTCATTGACTCGATGATGCTTAGCAAACTGACTCACCCTCTAGTATTGACAAAGACTGAAATTGAGGACATAAGTAGATAAATAAGTAGATAAATAAGGATAGCCCAATCGAGGATGAACTTCTTGGGAGTATTCATAGGACCCGCATAGGAAGAGCCTGAAGTTCCCTTGTACAGAACCGCTNNNNNNNNNNNNNNNNNNNNNNNNNNNNNNNGTCGATCCTTGCGCATCCTTCCACCGCCTGAGTTGAATCCGAGAGATCTTCGGGTAGAC

>Unigene7466_All

GGGGGCTTCACCTTCTGTATCTTGTAATTGCCACTGGCCAAAATGGTTTCTTAATGTAAATTTTGCTAAAAAATGTAATGGAACTTGAGGTTCTATAAAACGAACTTGAGGTTCTATAAAACATAATTGTTCTGATTTTGTATTGTATCTTGGCTGCTCTATACAAGGCTACCAAAAAGATGGAAATCCCTCGTGCATTTCTCACATCATTCTTCCGATGCTCATTTCCATATAATGTCCCCCAAAAATCTAAGAGCTGGAACACCTCTGATCTCTACATCAACCAGCGGTGCTTTTATCAATGTCAAACTGGAGAGTTCTGGATCACTTTGTATCATATCAAGAGCACGCATCTGATCCTTTCTTTTCATTGCACAAAACTTGCAGTCAGAGGCAGATGGAGGAAGAATTTGATTAACTATAAGTCTCTTAACAGGAACATTCTCCTTTTGCAAGGAGGCACGCAACCTAGACGACTCACTGACTGCCATTACCGTGGGAATTGTAACTATGACAAACTCTGTGGAGTCAGTATCACGAAAAAGCTCACGCACTTTTATCATCCTCTCCCTCAATCGTTCCAATTTGTCAGCAGCATCCTGTCGGTTTTCTTCCTGTCCAAAAACAGATTTGATGGCAGATGTGGCAGAAGCTATTTTTTGTTTAAGCTTCAATATCTTGCCTATGGATGCATCCAGGAAGNNNNNNNNNNNNNNNNNNNNNNNNNTAATGTATGGCCCGTGGGTGCAGTATCAAAAACTATTCGAGTAAACATGCTATATTCTTGAGATTCTAGGAATTGCATCACTTTTGAAATTGCTATAGCTTCATCCAAACCAGGAGGAGGTGTGTCCAGTAGCTCTCCCAATTTCAACTCTCCCAGCTGTTCAACAAGCATTCCAAGGCCCATGCCTTCCATAAAATCTTTGACCCCAGTTCCTCCATCTTTCCGAGTTGCTGTACGAAATTCTTCCCTAGCCTTTTCAGGGTTTATCTCAAGTGCATACAGTGGGTAATCAGGTCCTTCAACAGGTACTAACGTCCCTCCAGTTAAATTCTGAGCAAAAGAATCACTCAAAGAATGTGCTGGGTCAGTTGAAACCACTAAAGTGGGATGCCCATTGTTTGCAAATTTTACTGCAAGTGAAGCAGCACAACTGGTCTTTCCTACTCCTCCTTTTCCGC

>Unigene7519_All

ATTCATGAATTGATAGATCTACTTTGTGAAAAGGAAAGACTATTCACTTTCGTCGCAATTTGAAATTGTACAAGCACAATGTATCCACAAAATATGCAACTCAACTGACCTTCAAAATCAAATCTTAAGGTGTAATAGGAGCTAGGCTTGGACAGTGGTCAACCAGATCCTTAATCATTTCCTCCTTAAAATAAACCTGAAACCAGCGCATAGCCAGATGACAGCAACTGAGTGCTACCTGAGCTTCTACGCTATGCTCCTTACATATTGGTCAAGAGAGGTATTGAACAACTCACACCTGATAGGAGTTCCAGGTGTATAGAGAGGATTTTTGACAACATATTCAACATATAAGTNNNNNNNNNNNNNNNNNNNNNNNNNNNNNNNNNCAGTCCTGGGATGAGTAACCAAGATAATCTTTATCCCAGAAGGACTTTCCATGAAGCTAAGCTTGTATGTATTTGTTCGAAAGCTGTGGAATGAACAACCTTGGCCAGGTANGAGGCATCCCAAGGTTTCCTTTCTCCGCACTCGTGGGATCCATCTTTGCAGTTAGCGACTTGAGCGAGAAAAGCAGACCAAACATGAGCTTATGGTCCTGCTGCGCATTGAGGGTGTGAAGTGGGCGATTCCACTCCCTGTAGAGCAAACATACCCCGTTTCTATTGAACACATACATCATGTGCGCATTGTTGCCCGATGCTGTTGGTGCCGGTGGCGATGGACTGATGTCTGATCCTCCAAAAAATTGCATACTTTTCCACCAATCAAATGGACTTCAAAAATGTATACCACAAGCCCTGAGCACCCGGTTAGCTAGGCAAGCGAGGAAG

>Unigene7524_All

AGAACCCACAAATGGCCCAGCAGCGTTATCGACATCTGAAGCAGATACATTCACATTCGCAGACTTGTGAGGGGATAATAGAACCTGTTGAATTTGTTTATTTAGCGAAATCTGCTTGAGAAGGGCAGATCTCAAATCCTCTATCTTGAATCTCAAGCCTTGTTCCTCTAAGTTAGTGGACACAAAAGTGGCATTTGAATTAACACTAGAGTTGGAATTGGAATTGGAATTCAAAAGCGTATTGTTGTTATTATTGTGAAAGGGAAATGATTGGTTCAAGAGAGAGATTAGAGAAAATTGCTGCTGACCCAGCAAGTAAAGCGCTTGTAACTCAGCCTCTCTCATCCGGTCTGAAACGGAATCAAGCCTGAGAGCAGATAGATTGGCGGAGAACATATTCCTTATATCGGCGGAGAAAATGACGACGACGATTAAGAGAGGCAGAAAAATAGCCAACAAATAGTAGTAGTACCTCTTATTGAAGTTACTAAAGAAACGACGGCGTATTGAACCACCAAGAGACTCGAGATCCTCAATGTGGAAGGGGGAGGAGGACCGCCGCTGAGGAGAGGAAGTGGGGACTGGTTGGTGGTGGTGGTGGTGATTTGGCTTCCTATCGTTTTGCTCAATTAGAGTCTGGCGATCGTCTTCCTCGTCAGATGAATCTCTATCCATTTTCGGGAAATGGGTATTTGATTAGGCAATGCAAATGAGTATGAATTTGAGTATGAGAGTGAGAGTGAGAAAAGATCTGACAGTGGATGAGAAGAGGAGTGGAAGGTTAGAAAAGAAGGAATATCGTCGAACAGTCGGTGGATGGANNNNNNNNNNNNNNNNNNNTATCGGGTCGGGCCTTCGTGTCATTTAAGTTTGATTGGTGGCTAATGGCCAGGTTGGACTTTGGGTCCGTACTCCAGATCCAGGTTTCAGGGTTTGGCTGCTTTAAACGGTACCGTTTTATTCAAAAAGGCCAAAAATTAGGGGGAGAGAGGCACGTAATGAAAAATTGAAGACACGTAGCCATGGTGAACGCGTTACAGAGCAAGCATTTGTTAGTAGGTACGTGGAGACGGCAACAGTTGCAAGTTCCCATCAAAGGTAATCAGCAAGCAAAACACAGAGTAGCAAGTGAAAATGGA

>Unigene7579_All

AAAAATGAACAGAAGATCAATTGTAGCAATTGTGCTTTAGAGTTAAAAGATAACTTTTACTCACAACGGATCTTTCCAACTCAAGTAACACAATTGTTGACTTCTGTAAATGATTTTTGAAAAAACAAAAATGAAAAGCCAGTGGTTCATACCAACTCTTTGTAATATCCTAAGCTATTTCCCTGCCTTTCAGGTAGCAATTTCATCCACCTCAGTGTTGAAAACGAAACTCGGAAATGTTGTTGTGATATCCCTTAGGTAGGGTAGTGTCATGATCTTTAACAAGGATGGGTTTTTACAGACGAAGTTCTGCCATTCGGATCTGTCTATCTTGCCATCATTGTCAGAGTCAGCTTCCATGAAAGTCTTATCAAGAATTATCTCAATCGTCTCATCAGCCAACTTCATTTCAGATTCACAAAGGAGGGCAATCAACATTTGCTTAACCTCTTGGCGCTCAATAAATCCTGTATTCTCCAAATCATAAAGCCTAAATGAAAAGTCTATCTTTTCTTCTTGCGNAAAGACATTGAGTGCTCTAACAAAGTCACCAAAATCAATGACTCCCCTTTGCTTAACATCGAAGAGGTCAAAGATTNNNNNNNNNNNNNNNNNNNNNNNNNNNNNNAAAGAGCCAGCTGAAACTCCTCCTTATTTATTAACCCATCGTCAACAACAGAACTACTAATGCTCTTGAATAGTTCAAAAAGTGCTTCGACTTC

>Unigene7608_All

AAATCAACACAATCAACTCTAAACACATTCAATGAGCCTATTTCCCTTTCATTTCGTGTATTTTACAGTAATAAAATCATTGCTGCTATTTTAATTTAAAAACAAATCATCACCGGGTTGTCAAATCCCAAAAAAATTGAAGTTCTAATTTTTCATCGATTGTTTGAGAATAAGCTGAAATCCCAACGGCGCAGCAAACCATCCATAATGTGGCTTGCTTGGGTTCCGTTCTACTGTCAATGTGGCTTTTGTGTTACTTGACAAGTCAGTATCTATTGAGCTCTGGATGCTCCCTGAAGTATCACTACGGCCAGAGGTGGTTGTGCTTCTTGATTCCGTCATAGATGGTGAGGTGTCAAATGCTGGTCCCGTGGATGACACAGTAGGATCAGGTGATGGGTGTTCTATTGCTGAATTTTCACCATTGATTGCTGGAGAGGAAGCAGGTCCTATCTTTGAAGGCTCATGTTGCTCTACTGTCTTCAAAGAATCACAATGTTCTGCAATGGAAGATGCATCTATGTAATGTTTCTGTGAAGATTCAACGGAAGTGGGGTTGACATTTGCCTGTTGTTCTTCAGCATTAGGATATCTCATCTCCAAGTCCTTTAATGATGCAATTACTGCTTCCATGAACATCCTTTCTTCCTCCTCTACATCACATGGAAAACCTGCCAAGTCATCCAGTTGATACTCCACATATTGATCATCATCCATCGTTGTTGGAACATGTGGACCGTAAGGATGACCATTGGATAATTCAAAGCTTATCATTTTGGATGATGATGGCAAATGATCGTCATCAATTTCTCCACCCTCAACTTCAGATTGATTGCACTCAGATGGAATATCGGATGGAACCTCTGTTCGACTCATTGGTCTTTTGGGACAAACTTGTTCAAGGGCATGGAAATCTTTTCCATNNNNNNNNNNNNNNNNNNNNNNNNNNNNNAACTGAAAATTCCAGGTTATTGCCTACTTGCACAGTGCTCCCACTACCCTTACCACAGTAATCATGCATTGTGTCAAAATATGTTCCCCCCACCTCATCCTCTGGAGGA

>Unigene7626_All

GAGAAAATTGCTTTCATTCTTGAACAAGTGCGTCTATGCCTAGACCGCCAAGATTACGTTCGTGCGCAAATTCTCTCAAGGAAGATTAGTCCAAGGGTATTTGATGCTGATATTTCCAAAGAAAAAAAGAAACCCAAGGAAGGTGATAATATTGTTGAAGAGGCTCCTGCTGATATACCGTCACTCTTGGAATTGAAGCGAATATACTATGAATTAATGATACGGTATTACTCCCATAACAATGATTACCTTGAAATTTGTCGTTGCTATAAGGCCATATATGAAATCCCTTCTGTAAGGGAAAACCCAGCTCAGTGGATTCCGGTCTTGAGGAAAATCTGTTGGTACTTGGTACTTTNNNNNNNNNNNNNNNNNNNNNNNNNNNNNNNNNNNNNNNNNNNGATGATAAGAATCTTTCTGAAATTCCAAAATTTAAGGTGCTTTTGAAACAATTGGTCACAATGGAGGTCATCCAATGGACATCTCTTTGGAATACATACAATGAGGATGAGTTTGACACTGAGAAGAACTTGCTTGGAGGCTTTTTGGGTGAAAAAGCAGCAGAAGATCTGAAGCAAAGAATAATTGAACACAATATCCTTGTCGTTTCAAAGTACTANNNCAAGGATTACTTTGAAGAGACTTGCAGAATTATTGTGTCTCCACGTCCAGGAAGCTGAGAAGCACCTTTCAGATATGGTTGTGTCTAAAGCACTGGTCGCAAAAATCGACAGGCCAATGGGGATAGTCTGTTTCCA

>Unigene7642_All

GATGGACGGGTGAGTGAATGGGATGAATTTGAGTGGTCCAAGCAGGCTATTCATGTGAGTGTTCGTAAGCAAACCAAATGGTGGTATGCCAAGCGTTTTCTACATCCTGATATTGTTGCACCATATGACTACATATTTATCTGGGATGAGGATCTGGGAGTTGACCATTTTAATGCGGAAGAATATATAAAACTAGTGAAGAAGCATGGCTTGGAAATTTCACAGCCTGGTTTAGAACCTAACAAAGGATTAACCTGGCAGATGACAAAGAGAAGAGGTGATCGTGAGGTTCACAAAATAACAGAAGAGAAACCAGGCTGGTGCACTGACCCACATCTACCACCTTGTGCAGCTTTTGTTGAGATCATGGCTCCAGTCTTTTCACGAAATGCATGGCGCTGTGTGTGGCATATGATTCAGAATGACTTGGTCCATGGATGGGGTCTTGACTTTGCTCTCAGAAAATGTGTAGAGCCTGCCCATGAGAAGATAGGAGTCGTAGATTCTCAATGGATTGTTCATCAAACTATTCCCTCGCTTGGAAACCAGGGTGAGTCACAAAATGGGAAGGCACCATGGCAAACGGTGAGAGAGAGGTGCAGAAAGGAGTGGACNNNNNNNNNNNNNNNNNNNNNNNNNNNGCAGAAGAAGCATATTTTAGATCAAAGGCAATTGAATCTTCCAATTCCACAGTTCATTAGCCAGACA

>Unigene7675_All

CGGAAATTTTTGGACACCAGCATTCATCCATTAGTATGTTCTCTGGTTTTATATCACAGTGGATGATTTGCGTCTCGCACTCTTCATGTAGATAGAGGATGCCCCTTGCTATGTTCCGGGCGATCTCCACTCTTTCTCCAAAACAAGGTCTGCTTTCAGGAGAGAAGATTACATCAGCAAGTGACCCATTTCTCATGTACTCGTACTCGTATACCAAGAGCCTGTTAGGCCCCTCATTGCAATAACCAAGAAGGCGGACTAGGTTCTTGTGATGTGTTTTCCCAATAACCTTCACTTCAGTTTGGAATTCTCGTTCCCCTTGAGATGCAGCTCCTTCTAGTCTCTTCACAGCTGCAACTTTCTGGGTGCTCGATATTAGCCCTTTATAAACAGTCCCAAATGAACCTCTGCCAATTTCTTCCTTGAAACCATCTGTCACTCTCTCTAGTTCTTCAAAGGTAAAGGATCGAGGAGCAACTTCCTCNNNNNNNNNNNNNNNNNNNNNNNNCAGAGATCCTTTTGTATGCCCACACACGATATTTAAGAAACAGAATGCCAGAAATTGCCAACGTAACAAGCCCAAAAGCAACAACTGAAATGCCAACGATTAGGACGCCCCTGCCGAGCTGATCACTTTTCTTTTTGTCTACTGCAGGCTCGTTTCTGTCTGTGTTGGTCCTGATTGATGTAGATTTAACCTTGATCAAAGCTGAGTTTGACTCATCCGGGTTTCTTCTCCCATATCTCAAAGGCAGCTTTTGCTTTTTGCACAGACCGTCTTCGTAAAACGCAGCATCACAGTTACAATCCTGCAGACATGCTTGCTCGCACTCTCCCTTGATAGATAATGAGAGAACGGAGTATGATACATCTTCCCACGCGGTGCTAAGAACTTCTTGAATTCTGATGCTTTTATGATCTTCTTTGCAACTTTCAGCTGTGAAATTCCTCTCACATCCAGCAGTCCGATCTCCCGGATTAACAGATGCAAATCCTGGAAGACATATACATTTAGGTTCCAAATCATTTAAAGCGCAATAGCCATTAAGACCGCACAGTCCCTTGGGTTCGCACTTATTAGTTGTGGATGACCACAAAACAGAAG

>Unigene7684_All

GCGCTCTATTCAAAAGGTGCGCAATCACACATGTTGTGCTTGTTAGCTTCTTCGGTTCTCAACCTTTTTATAACATATTTCTAGCTACAATTTCACACCGATTCCTCTCCGTTCACTCGCTCTTAATATTTTTTTCCCCTTAAAACTACTCGCTCGAAAGATCAAACTCTGCCTCTCCTCGGTCCACTTCTCCTTTCGGCTCACCGCCATTATCCCAAAACTTGCTTGTAAAACCCACTTTCTCTTCTTCTTGCCCATTCTACAACCATAACATCGCCTCGGAAGAAAGCTTAGGTCCTTTTATTATTCTTCTTTCAGGCTAATCCTCCTAAAGAAAGAGCAAGCAGAGGTCTGTTTCTTAGTAGACGCTGAGACGTGCNNNNNNNNNNNNNNNNNNNNNNNNNNNNNNNNNNNNNNNNNNNNNNNNNNNGACTGAATTTGCTCGATTTGCAGAGATTGTAGGGTGAAAACGAGGTCGTTTTATTTATTGATTCGAAGTTTTAACATGACCTGAATTTTGTGTTTGCGAGAAAGAGAGAAGAAGAATTCTGTCAAAGCTCATAAAAAGCGGTCGTTTTTTCTGCTTGGAGCAGTGAAGTCCGTGAAAAAATGCCTCACAGAACGACTTACTTCTTCCCCAGGCAATTTCCGGATCGGTCCGGACTCGATGTATCTTCTAAACAGCTGTTGGATCACGAGAAGAAGAAGCTTGTCAAAGATACTTTTAACATCGAAAATGACCACCGAAATGACTCCTCTACAGTTGGCAAGACTAAATCGCCAACGCCAACTACGACTGCTACTACGCCAACTACTGTATCCGATCTCTTCACGAGCAGCGATGACGAAAAGTATCATCAAAAGAAGAATCAGTTTGGCGAAGACAATAAGTTCCAAAAGAAGAAAAAGCAACTTGCTGCCTTTTATGACTGGCTCGCCG

>Unigene7740_All

CAGAGAACTTCAATAAATTTCATTCTTTCAAAGAATACTTTTTCCCTGTGAATGCTTGAAATTTAGGTTCTTCAACCTTCTGAGGTGCCTCTTGACTGCTCTTATTTGGGGCATTCTTTGGTGTTCCACTTCGGGGTTGGTTTACATTTGAACCAAAGACGAGCTTCCCAGAGTGTGAACAAGATGCAGAGTTTGATGGCTTGGAGCTACTACTATCTTTCCCAACCTCTTGAGGGTTCTGTTTCAGATTGTTAGCTGATGCAGTTGCTAGTGCTGATAGTTTTCCATCCAAACGTCTTGCTACACCAGTGAATGGACTGAACTTTGGCATCTTTTTGGCTGGCTCTTCTTCAACTTCAGGTGGTGTCTTATTTGACCTAGGAGATGGAGCTGGCTTTTCAGGTTCTTTATAGTCAAGAGGCGGAGCAAAATCTACCTCACAGTCTGTTTCAATAATACTGATTGCAGAGCATGGTTTTGTTTCAACTATGTCAATGTAGTACTTCTTGTTGTTATAAGCAACCATTATTGTATCACCAGTTGTTAAACAAGAGTAGTTCCTCAATGAAGTCTCTAAGATTGCTTTGGGGTTGGAGATATCTAAGAAGTCCTTAGTGTGGGGCTGCAGCTTTACATAAGTTCCCTTTGTGAGGCTGGCATTTTTTAATTGCACAATGTCTCCCTCTTGCAGGAGCATGTTTTCCATCATCCAATAGGGCAAGAATATGATTCCCTCATCAGCAATAAATTCTAGAACCCCACAATGTGTAACTCGCTCAACAGATGGATTTCGGAGTTCAAAAAGCATTGGGTAGTCAATGTGCAAAGAAGCAAGGTGATCAAGAGCTGAGGGAGGCATAATAATCTTATCGCCCTTTTCCACATGTGTCTTATCAATGAAAGAGACAGGGTAACAGCGATAACTCCGTTCAAACGTTGAACTGTNNNNNNNNNNNNNNNNNNNNNNNNNTAAACAGCTCCAACCCACAAGATGTCCACCTATGGAATCAGAGATTGACTGATGACGACGGGGAGCCAACACAGTTTTGGGTGAATGGAGGAAGGCGGTCCCCGGATGGGTGGAACTTGCTGTGGGTGAATGACGGTTATTTAAGGAGAGTGCGGG

>Unigene7791_All

AATTTCATAATTTCTTCAAAAACAAAATTTCATAATTAAGAGTAATTTTGTAACTGTGTGTGCAACTATTTCATCTTTTGGGAATAAAAAAAGATTGTATAAACAAACATATGACTACAAACCAAGTGAACTTGCTGGGAACGTAACGACTATTCGTGGATTGGTCGGCAAGTGTTGAAAATCTCGCCTCTCATCACATCAATTGGCATGTGCTCAAGTGTGTAACGTTCAGGACCTAGATACACGCCCGTCTTCTCCAATAGAGAAAGCCCTTTATCTCTTCCATATCTTGAAGTGTAAAACTTCAGGTATTCAATGTCCTGTAAGCCACTAAGAATGCGCTCTAGTCTAAGTGAAGCAACTGGTTGACTGGAAGATGAAAACACCTCACCAGGATAGAACAAAACCCCATCACCAGGAGGTAGACCGCGCCTAAATCTTATCTCTGCACTAGGAACTGTAGCCTTCTCGTAGCAGTTGGCACCCCAATATAGAAACCCAGTTCCACCTTCTTTCCATACACGCCACATTACAGCACGATGCTGAGTACCACGCATCCCTAGATGCCAATTAGGATGAGGATCAGAGGGGCCCATACACACATATGTCCACCATTCCTCACCGTTCTCTGGCTGCAATTCAGAGATAATATCCTTGACCAGATCCTCCCGATTACCAAGCACCCACTCACTTGTACAGTAAATTTGAGTGTGAGGGCGTAGGAACTTTGGAACTTTTACAAACGCCTCAAAAGGTGTAGGTGCAAGTGGTGCATCACTAGGTCCACAATAATAAGTAGTTAAAACACGGGCATCAGGAGCATAAGCATGGATCTCACTGGCCATGTTGCGGACTGAGTCATATTGCTCCAAATTCAATGGCTCATCCCACAAGTAAAAATAGGCTTTCTTCCAATGAGACTTTGTCCTCAAGATCTCAATTTCTTTCTGCAAGTAATCCTTCGCTGCATCATTACCAGACACTGCTTGATTGTATGGTACAGCATATGCTGCCAATCTTGGGTCAGACAAATATTCATCTGATTTCCAGTGATCAGCTGGCCATGGGCAGGTGTAAGTCAAAACACGCATACTGTCTCCCCATCTGCAAAAGTATGGGCTTATTCTATACTGAAGAAGCCACTTGAAATGCTGTTCCAACGCTTCATACCATTCATCACTTCCATGCTCAACACCAAAACGATCCTCAATTACAGTATCTGATATACCAAAAACAGCAGGAAGTGAAGGAGTTGCTGGAAGTATGAAATCCCATACAGTTAAACTTAACTTCACACGAACTGAGAGATTTGAAATGGCATCTTCATCCATCATATCAACTGGCCCATTATCTGAAAAGAATTCAGAAAATGATGCAGACAGAAGAATTTTTCTTAAAGATGTAGTTGCAGATTTCACTCTTTCTACCACTTCATCCAATGGTTTTCCTTCAATAGGCTCCACAGTGTCAAGACAGGCCCTAAGTCCTGTGTATAGTCGATGCTTCTCAGCTTTGCTCAAGCACTGTGATTGAGATTCTGACTCGGCTTTTATGGCAGTAACGATGAACTCCCCTTCATATTGACCTGGTGGCTGTACACTTGGAACATCTATGGAAACCCAAACAGCAGTTGTTTCTCCAGGAACTAGGCCTATCTGACAAACTGGATGATCTAAAGGCACAAGAGCATCTGGGACACCCAATATGGAAACCACTTTCCGAAACGTTATTGATTGTCCAACAACCAACCGATCACCAGATGTGGAGGATAAATCAGTACATTGAATCTGCACAATACCTGCATTACCAGAACTGCTCCAAGAAACTTTTGGGCGAATAGCAATTTGAACACTTTCTCTCTCATTCCTAGCTGCCAGAAGATTTACAGGCTCCAAATGTCTTGGCACCTCTTGTGGTCCAACATTCGCTGTGCTTGGCATGCACCACACATGCACCAAGTCTGCAGTTGGAACCTCTGAGGGATCAATTGATCCCTTGAGTGGATTTGAACCATGTAATCCTCCATCATTCCATCCATATGCTGTCCCTCCACCGGCAACACCTTCAACTGGTGGCACAATAACATCTTGAGNNNNNNNNNNNNNNNNNNNNNNNNNNNNTGCTTGAAATTACAAAGATCTCGATGTTCCCTCCGCTGTCGTCTGGTTTTAGGCCGAGGTAAGAGAGCAAAGAGTTTCCTGACAGAGAAGATCGCTTTTGGAGAGTGGAG

>Unigene7809_All

CTAGACAAGACCAAGGCTTCAATAAAGAGGATGCAGTCTCTAATTATTGTCACATCCCAGGCTGTTTCCACCACTTCAACTGCTATCATTGGTCTTAGGGACACTGATCTTGTCCCTCAGCTGGTTGAACTGTGTCATGGATTCATGTACATGTGGAAATCAATGCATCAGTACCATGAAGTTCAGAACAACATCATACAACAAGTCCGTGGCCTTGTGAACCGATCAGCCAAGGGTGACTCAACATCTGAACTGCACAAACAGGCAACTCGTGATCTGGAGTCGGCGGTATTCGCCTGGCATTCCAGTTTCTGCCGCCTTATAAAATTCCAGCGGGACTTTATCCAATCTGTCCATGGCTGGTTCAAGCTCACCCTTCTTCCTGTGAGCAATGACAATGTCAATGGCAATGTGGAACACTCTGATTCATATGCCTTCTGTGATGAGTGGAAGCTTGCTCTTGACCGTGTACCAGATACGGTTGCTTCTGAAGCCATCAAGAGCTTCATCAATGTTGTTCATGTGATATCTGTAAAACAGACTGAGGAGCTGAAAATTAAAAAGCGAACAGAAATTGCATCAAAAGAGCTAGAGAAGAAGGCTTCTTCTCTTCGGAACATTGAAAGGAAGTTCTATCACTCTTACTCCATGGTTGGTATTGGGCCCCCTGATACTGGACCTGATAACGGGCAGGTATTGGATGCTCGGGATCCACTGGCCNNNNNNNNNNNNNNNNNNNNNNNNNNNNNNNNNATGAGATGCTGAGACATGCCAAGGCAGTGGAAGTGACAAGGGCCATGACACTGAATAATCTTCAGACAGGCCTGCCAGGCGTTTTCCAGGCATTGACTAGCTTTTCTTCCTTATTTATGGAGGCACTTGAACTGGTATGCAACCGTTCCTATGCCATCAAATAGTTATACATGATTTTTCTTGTTGGATTTTTGAGGTAAGTTGGAGTTCATAAGGGCTGGGATTATTTGGCTGTGGGCTCTTAAGATATTACTTTTTGGGGGATATTTTTGATATGTAAATATGTGGAGCATTTTTGCGGTTTTGTCATCCTTCTTTAGACCAAGGGGAGGGAAATGTCTCTGTATAGGAATTTGGTTAGA

>Unigene7831_All

AAAAATTGAAGGCATTAGAAAGAGCGCGAGAGAGAGGCAAAGGAATTAAACCGCCAACACAAACAAATCAATGGTACAGATATATACTGAATGGGCATTGTTGGTTTCTTCTCCACGAACAAGCGGCATGAACATTCTCCAAAGAAAATTCAACATAAATGACCAAACATAATCGAGACCAAATTTGCCAAGCTGTCTAGACTGGAGCAAGCAAAACTTTCCTTTACCTAGACCTCAGCCAAATGGAGAAGGAACAGGCCCTGGCCCTGCAAGCGCATCTGATCTTTTAATACTGCCCATACCACAGATAATACATCCAGGTGTTGAGAGGGCAGAAAATTTGGCCCCACAAAGATCACAAACATCATATCCGATGGTCGACAAACGGCTGAGTGTCGCACCACAGAACTGAGAAGGGTCCTCCAAAGGATCAATTGACTTGTTGGTTGAACCCCTCTGAACACACATGTCAATCAGGCTTCTCAACTCATCCTGCTTGCTAGGTGGAGCTTTGGACAGGAGGAGCTCAAGCATCTGCTTGGAATAAGCAAAATTCTGCACCTCCATGTTTCGTTTAATGGCTGTTCGGATGCAATTTATTCGGTGCTTTGCCAGAAGAGGCAAAGAACCTAGATGTCGTGATAGTCTCGCCATCTCATCTTTTGCGCTCAGTGCACTTGGACCTTGTACTTTCTGTAGCCTTGCAATTTCCTGAAGCAGAGTAACTGCTATCTTGTACTGAGCACAGATGGTTGCTTGGGCTTTAATATCAGCTCCACGAGAGCTATCCTTTGCCAATGCCAGGAATGCTTCATCAAAACATGATAATGCATCAGGAAGTTGATTCTGCTCAAGGTGAGCAAGTCCAATTTTGAAACATACAGAAGCAGCCGCTCCTCTGGGAACCTGTCCAGGTCTCACAGATGATGGTGGAGCAGCAGCTTGCACAGGAGGTTTTCCTGAATCTGCAGAATTTGGAACTCCTAGGACACTGAGATCAAGAGGTTGAGATGAAAGAGGGGCCCGAAGGGCCTGAGGGGCCTGCACATGTGGCTGAGATGAGAGAGCAGCCTGACTGGAGGCTTGTGGTGGGACACCACCATCAGGAAGTCCAATGGATTCAAGTGAGACAGCTTGCTGAGTGGCTTGTGGGGAAACACCACCATCTGAAAGACCAATGTCAGCCACAGAAGGACCAACTGGATTAGGCATTACCTTGTCACTTTCAANNNNNNNNGAGAAGTTTGATCCAGTTTTGCAAGATATGTTCCAGGAGGAGGCAATGATGCTGCAACTTGGAGGGAAGGAATAGTATTTTGGAAAAAGTCCTCTGGAATAGGGCCAGCTGTAACACCCATACCCNNNNNNNCTGGTTGTGATACTGGTGCTGATTGTGTTAATGTGTCAGTACCAAATAAATCAGCAGCAGGTGAAGGAACTGTGGAAGCAGCAGGGGCACTTGCACTGGTAGGAGGGGGTTGGGATAAAATCTGACCAAGGTCCTGGGAACCAGTTAATGACTTTGTCCGCATGGGTGGACCTAAACCTTCACCAAGTTTAAATATCTTTGTTGCTTCTTTGATCTTATTGACATCTACTGTAGCAGAAGTAAGCGGTTTGTCTCGTATTCTAATTTGTAGTTTTCTGGCTTTCGAAGGTTCTTCCTCATCACTGCTGCTACCATCAGTAGCTGCACCATACATANNNNNNNNNNNNNNNNNNNNGCCTGTTCATCAGCTGCAGATGAACTTGCATTTTGTTTAATAAGTGTTTCAAGACCCATCAAGGAATCAGCCTTTACGTTACCATCAGGCAATCCCTTTGACTCATTAGACATGGATTTAGATAACGAGTTTGGAAGTCCATTTGGCTGATTGTCACCCACAGACTTAAAAGCTTTGACCAAACTATCCTCCCTTACTTCAACAACGTTGCCTCTTCCTTTAATTGATCCCAGGTACACACCAATATGATCTGTAATAATGGCAGGGATGGGGGCATCGTCAGGCTTCATCATTACTTCTGCAGCCAGTTCCCACTGAGGTATACTTTTCAAACTAGTAGGCGTTTTAATTTCCCAGTTCCCACCACCCCATTCATTTCCTTTTGGAACCATGCTCTCAGCTGCAAAGTTGGCAAAAATGCCTTGCGTCCACCCAGTAGAACGAACCCTTAAAATCCTTTCACAATATCTCCTTAACTCTGGATCTGCACCTTCTTCTTCCAATCTCTGAGCAAGGCGTCTCATGGCACTAGGGTTAAGGTGGCATATGAACAAATCAAGCATGCTTTCATGATCTGCTATAACTTCAAAAGTTTCCTTTGCACTATCAAACTGGCCATACTTGATACAGGCATATCCCAACTGTCGAAACCGGTGGAACAACTGAGATGTTGGAGGACATTTTGGATAATCTCGAGATCGCAAGAATTCATCCTTCAATACAGATAAAGCAGTAGAAAAACGAAGAGCTTTGATAGCATAGGTGCCTCGCAACACCTGGGTGAACTGTGGACCTGCTTGGGACAATGAAACTGCAAGATCTCCACAAACAGGAGGACCTCTAGCAAGAATATCAAGAGATCTTGGAGTGATGCGCAAACTATCAAACCTTGATGTTATTTGATAAAGTGTTTCTGAGAGGTCAAGCTTCTGCTCAAATGTTTGTTGCATTGTGGCAAATCCAATGAGAAGAGGTTCAAGGAGTCCAACAAGGCAACTCCTAATCTCCAACCCCTTTTTCTGTCTCGGATTTATATCCGTTGGNNNNNNNNNNGATCATTCAAAGCACCAATTAAAACTGAATAGGGCATGCTAATAGAAAGAATGGTCCTCACTTTTCCATCCCAGCCAAGCACACTGACTGAAGTGGCAGTAGAAAAAAGAAGAGCTGGTCCAAGCCATAAAAGGGATCTAAATGAAGGAAGTCCTTTATCAAATTTCATAGAACTGCTTGCCAGTATATCAAGATCAGCTGAAACTATAAGAACCCTTTGAGTTGTTAATACTCCTGCTACATAGCCCCTAGGGGTTTCCTGCCAGTGCACCTGGAGTACAATCTCATTCATTTTTAACTTGATTGACT

>Unigene7834_All

TAATTTTGTTTAGTGGTGGGTCTCATAACCTCCATATGCTGCACACCAAATTATATAGCGTTAACACAAAGTTAGTGGACATTAGCTCAATAACTTAGAATCTCCTTTCTGAGACCCCCTTTTAGGAATTCTAATCTCCTCCCTAATAGTCCACAAACAAAAATCAGAAGTACAACAAATCAAACAATTATGGCTATCATCTTCTTCACCCAACGCACAAATTAAACCTGCTCTCTAATTTCTCAATTTTCTTCATTCCATGTACTGTCCGATTCCCTTTCTACTTCCCATTCTTATTTCAAGGATACACGAATGGTCTGGTCGATCTAAACAGAGCTGAAGGTTAAACTTGTCTGAGAAAACTTTTAAAAAAGAGTTGGGTCCTCCCTACCTCCCAATGTAGCCCAGCCAGTGAAGGATCATGGCTAAGAGGAGCATGCACATGGCCATAATCATACCAGGCCTCCCTAGTAACCAGTTTTTTGCAGTCCTTGCACCTTCAGCTCCTCTCTGGATCCTATCTGCCCATCGCATCATTCTATCCAGGTCCTCTGGTGATAAAGACGACATAGCTTTTTGAGCTTTTGCTGCATCTTCCGGAGAAAGTTCCAATCCAAATTGTTCACTCATGTTGGCCATCATCTCTGGGCTCATATTCTTCATCATTGATGTGAACATCTGTCGCATAGCTGGATCTTTCATTTGGTTTCTCACTTGTTCTTGCACATCAGCAGTTGAGCTAGGAAAGCTTGGCTGAGAAGTATTTTTTGAGCTTGAACATAAACCATTAGATGAACTTGTTTCACTGCTGACATTCCTATGAACTGCAAAACTTCCTTGACGTCCTGTCAGATTTGACCCAGTGTCCGAATTTAGTCCATTAGTACTTAAAGCTGATGTTGTAGGAATTGAGTCATTTCCTCTCAAAGAGGATGCCATTTCAAACATTTTCTGAAGCTCTTCTGGAGGCATCCTACTCATCATATCAGTCGCTGTTTTAAGCATGTCTGGTGACACATTTGGTGGAACTGGGCTGGGTTTGAAGCTGTTCAAACTAGCATCTGAAGAACCTGCCATTTCAAACATTTTCTGAAGCTCTTGTGGAGGCATCTTACTAATCATATCAGTTGCTGTTTTAAGCATGTCTGGCGACACGTTTGGAGGAGCTGAACTGAGTCTGAAGCTGTTAAGACTAGCATCTGAAGAACCACCAGCAGCATATGGGTTTCCCCCCTGAAATGAGGAGGCCATTTGAATCATTTTCTGGAGCTCTTCCGGTGACATCTTGCTGATCATATTTGAGGCAGTCCTGAACATATCAGGAGACACCTCTCCAGCTTTTGCACCACTTAAAGCAGCCAAAGTTTCNNNNNNNNNNNNNNNNNNNNNNGTTTTGGAATGATCTTATAGATTCTGGGTCATCTTTTAAAGCATGCAGAGACTCTGAATTGGTTGAACCCCTGCCATTAGCCCTACTTTCACCATTAGGAGCATCAACAATTTGCTTGGTTGAACCCCAGCCATTAGCCCTACTTTCACTGTTAGGAGCATCATCAAATTGCTGTGGTTGTGAGACTGAATATTCTGCAGATAAACCTAGGTTTTGAGAAGATTTAGTCTCAACTTCTTCAGTTATTTCTTCAATTATCAATCCTGTTTAGCAAAAACAGGGAGCGACC

>Unigene7837_All

TTTTTCCATAAAAGGCATGTGAGGAAGTCGGCTGGTTCCATCACTGCTGAATGTATGTACACGTGCCACCGTTGCCTTGATGGGAAATATGTGAAAATTGACTTAAAAACGGGCAAGAGTTATGGAAAAAGAGGGAAAAATAACAAGAAAAATATGAAAGTTCAGCATCAAAAGTCAAAAAATGCCTCTGTGGCTTGCAGGTCAGCGCGACTTAAAAACGGTAAAAAAGCTGTGAAAGGCCCGTGGTCGCTACGATCACGAAAAAAAAAGAAAGTTGCTGTTGTTGTACCTCTTCGTCGTTCTCCTAGGAAAGCTAAATACAACAACTCTTTGCATAATAAGAAAGTTGGAGGATGCAAGAAAGATAAACAGGTCAAATCCAAAAAAGCAACATATAAGAAACCAACAAGTGTTGCTTCATGGCGCAAGAAGAGGACACAGACTTGTCATAGTTACTGGATTAATGGTCTTCTGTTGTCTAGAAAGCCAGATGATGAACGTGCGGTGCATTTTAAGAGTAAAAATTTTATTGCCCCTTCCAACAACGACATACTTCATCAACCCAAATGCCATCTTTGCTGTGAAGTTGTATACACATCTACCTTGAATTATATTTCTTGCGAGAGGTGTGGTGAGTGGTTTCACGGAGATGCTTTTGGACTTGATGTGGAGAACAGCCATAAGCTTATTGGATTTAGGTGCCACATGTGCCGAAATAGTACCCCTCCTGTTTGCCCGCTATTACCAACCACAAGAAGACATGATATTCAGAATGAACTCTCTGAGGAAGCAATCAATGCTGTCTCACAAAGTAAGGGAGTGCAAAATGATGCTCGGAATGAACTTTCAGAGGGAGCAACAAATAATGGCCCGCATCAAAGTGAGATAGTGCAAAATGATGTTCAGATTGAAGTTTCTGAGGAAGCAACCACTGTCCTGCATCTAAGTGAGATAAATTCATTGCGAGAGTCGCTTGCAAATGACGATCAATGTTCACTTCTTGTTGATGAGTCTGTTCAAAAGGAGGAGCAGTTAGTTACTACACTGGATTCAGATCAGAGTTTTGCGCCTGGATCTAGGTTTGAAGTGGGCAAAGGGCAGTTACTCGATTATGTGAAGGAGGATACTGGTGCATCTCAAATTTCTAACAAGAAATTGAATCCAGAACTTATATCATGTAATGGAAATCATATGCTTAAAGAGAGCACAATTGATTCGGGACAAGATGCTGTTGTAACTTCATCTGATCAAATGCAAAAGCTCTTCCCACAATGTGATGTGGATGTGATAAACTGAACTGGCGCCATCAGGGTGTGAATGTGTAAGGGATAGCTGATCAACCCCCATCTTGAAGTCATCAATTGATGGAGCCCTTGTTGATTCCATTGAGTTGAATCCAATTTATTTATGAACTGAAGTATAAGGGGAGGGAGGAAATCAAGTGTTGATTTGATCTGTAGGCCTTCATTTAATTGGTGAAACAAGAAACTGTAGAGGCTAGAGTTATTTGTAAATCTTCTAATATTGTTGAAATTCATGGTAGAAATTTAAAAGTGACACTGGGTTGATTCATTTGAAACTGAGAAATAAAGAGTTTCATTGGGGCTGTCGTTTCTTTATTGACTTTCCATTTCATGAAGAATGCTCCGCTTCATTGCTTGGTTTTCAAGATGTTCAGGTACACAAATGGCTGATAAACCCATCCATGGAAGCATGTCCAGAGAGGCTTTGGATAGTCTTTCGACAAGGTCTGGTGACATTTTTTGTCTTCCATGTTCGAAGAGGTTGATGTACTGCTTNNNNNNNNNNNNNNNNNNNNNNNTGGTTTGTTGAGAGAGCTTCAAGGCATGGAATAGCAGCTGATTGATAAACGTTTGCGAGGGCATGACGGGAACACAATATTTCATAACCAAGTGAGTAGTCTTAGTGTTTAACACTTACTTTAACATTGATAGTTCAGATTTACTTTCAATTGAAATCTAGTATTTGTTAGCTGCAAAAACTCATGTAGATATCAAT

>Unigene7838_All

AACAATAAGCATGGAGGAAGATGGAACAAGATCACCATTTTTGGAAGAAAAGGTGGATCATAAGAATTGGGAGATGAATGAGCATTCTTCTTCTTCTTCTTCTTCTTCTTCTTCCTCTTCTTCCTCGTCCATCACATTTACGCTTGTCTTCTCCGCATTAACCATTGTTTGTGGTTTTTACATCTATGGAAATGCTATGGGGTATTCATCACCTGCTGAATCTGGAATCTTGGGTGAGCTCCGCCTTTCTTTGGCAGAATATTCGCTTTTTGGTTCAATATTAACAATAGGGGGACTAATGGGTGCATTATTGTGTGGGAAGATGGCAGACCTCATTGGTCGGAAAGGTGCAATTTGGATTTCAGATGCATTGTGTCTCATAGGGTGGCTTGCAATTACATTTTCCAAGGTTGCTTGGTCACTTGACCTTGGAAGACTGTTGGTGGGAATTGGAATTGGGATTCTTTCTTATGTGATACCTATTTATGTTGCAGAAATTACACCGAAGAATTTCAGGGGAGCATTTGCATTACTTATTGCACTTATGATGGGTTCTGGTATATCAGTTACGTTTATCATTGGTTCTGTCTGTAACTGGCGCATCTTGGCTCTACTAGGTACTATTCCATGTCTAATTCAGCTCCTTGGTGCATTTTTCATTCCAGAGTCTCCCAGATGGCTTGCAAAAGTTGGCAGAGAAAAAGAGCTGAAAGTTGCTCTCCAGAGCCTTAGGGGAAAGAATGCTGATATTTCTCAAGAGGCTGCTGAAATCATTGATTATACACAGAACTTTAATCAGACATCAGAAGATGGCATTAAAGAGTTGTTTCAAAGGAAATATGCTCTTGCAATTATTGTTGGAGTTGGGCTGATGGCAGTTCTACAATTTGGAGGGCTCAATGGCTATTCTTACTATTTAAGCTCTATTTTAGAATCAGCTGGTTTCCCTAGTAGTGTTGGGTCTGTAGTAGCAAGTATTGTTCAGATAGTGATGAATATTTGCAGCTTGTTCTTAATTGATAAATTTGGAAGAAGACCGCTTCTACTGGTTTCTACAAGTGGATGCTGCTTAGGTTCACTTGTAACAGGATTGTCCTTTCTATTGCAGGGCTATCACCTAGGGNNNNNNNNNNNNNNNNNNNNNNNNNCAGGAATATTGGTATTTATAGGTTCTGTGTCAATAGGCCTGGGAGGAATACCATGGATTATGGTGGCAGAGATATTTCCTGTAAACGTAAAAGGTTCAGCCGGAAGTTTAGTAAATTTGTTCAGCTGGTCTGGGAGTTGGATTGTTGCATATACTTTCAATTATTTGTTTGAGTGGAGCTCAGCAGGGGTATTTCTGATTTATGCAATCATTGCTGGCTTGGGAGTTATATTTGTGGCAAAATTGGTGCCGGAGACAAAGGGGCGAGCACTTGAAGAGATACAAGCATCATTGATTGCCTAGACTTAGTCTACCTGTGATCCAAAATACAAATATGTGAAACTCATATATTTAATAAGTGGATATCATATATCTTGTATCTTAGATACAAGATAGGATCGAGTTCAGGCGAAAAAAAAAAAAAATCCATGCGAGGATAATAGGGTTGAGATTTTATGAGATGGGTTCAAACAATATCAAGCATAATAACCCAAGAAATTTAATTTAAGCAATGAAAAATCAC

>Unigene7857_All

GTCATTTTCCTGCTTTTACTTTTGAATTTTGATTACTGCTATTTTAAAAACGCCATGAAAAGCAAAAACTAAACTGCATATGAAAACCAGAAGCTTCTTTATTCTTTTGCTATGTCTGTACTCTACCAAAACCAACACATGAATCCTCTATCACGGTCAAAAATATCCAAGGAAAGATAGATATATACAGCAGCAACTATATAAGATACCCAGTAAATTTTACTTGACGTGCCAATAGCTCCCTTGTGAGTTCTTGATTCCAATATAAAGTAGAACCCATCATCTTAAAGCTAAAGCTAAAATAAACCCATTTGCCTCAAGGGTTATCCTCTNNNNNNNNNNNNNNNNNNNNGAGGATTCAGAACCAGAACGCCTTCATTTTCCTCCATTTTCCTGCTGTTTCTCACTCTATCATGTGTTTCCAATGCACAACAGCAAAATCCCATCAAGACCATTGTGGTTTTGGTGATGGAAAACAGATCTTTCGATCACATGCTTGGGTGGATGAAGAAAACGGTGAATCCAGCAATCAATGGGGTGACAGGAAGAGAATGTAACCCTGTATCGACCCAGAACCCAAAGCAACAGTCTATTTGCTTCACAGATGATGCGGAGTTCGTGGATCCAGATCCTGGCCACTCCTTTGAAGCTGTGGAGCAACAGGTATTTGGCAATGGCCCAATTCCCTCCATGACAGGCTTTGTTGAACAAGCGCTCACGATGTCTCAAAACCTCTCTGAAACTGTCATGAAAGGTTTTAGGTCTGAATCTGTGCCTGTTTATGCCACTCTGGTAAAAGAATTTGCAGTGTTTGATAGGTGGTTCTCTTCAATCCCTGGTCCAACGCAACCCAATAGGCTATTTGTATACTCTGCTACCTCTCACGGCTCAACCAGCCATGTTAAGAAGCAATTGGCTCAAGGGTACCCTCAAAAGACTATATTTGATTCACTTCATGAGAATGGTAAGAACTTTGGGATTTACTTCCAGAACATACCCACAACTCTGTTTTACAGGAATATGAGGAAATTGAAGTATGTGTTTAAGTTTCACCAATACGATTTAAAGTTTAAGAAAGATGCTAGAGATGGAAAGTTACCTAGCTTAACTGTAATTGAACCAAGGTATTTTGATCTTAAGGGATTGCCCGCGAATGATGACCACCCATCTCATGATGTTGCCAATGGCCAAAAGCTTGTCAAGGAGGTTTATGAGGCACTAAGGTCTAGTCCTCAATGGAATGAGACCCTTTTGGTCATTACTTATGATGAGCATGGTGGGTTCTATGATCATGTCAAGACTCCTTATGGACATGTCCCTAACCCGGATGGGAACACCGGCCCTGCACCTTATTTCTTCAAGTTCGATCGCCTTGGCGTTCGTGTGCCCACAATTATGGTCTCCCCTTGGATCAAGAAAGGCACCGTGATAAGTGGCCCTTCTCCAAACTCAGAGTTCGAGCACTCATCGATCCCTGCCACCATAAAGAAAATTTTCAACCTCTCCTCCAATTTCTTGACCCACAGGGATGCTTGGGCTGGCACATTTGAAGGAGTTGTTGGGGAGTTAACCTCTCCCAGGACTGACTGCCCAGTGACCTTGCCGAATGTGGCGCCTATGAGAAGCACCGAAGCAAAGGAAGATAGTAGCCTATCTGAATTTCAGAGCGAGATAGTTCAACTAGCGGCGGTTCTTAATGG

>Unigene7901_All

GTCATCCTCTCTTTCTTCTCCTAAAACCCTAGCCGAACATCTCTCTCCCTCTCGTCGCCACCCAAAACATGACCGTGACCGTAGAATTGGATGACGGTTACGCGGCGGAGAAGCTCTTTAACCAGGGCTTCTCATACACCTACGACGACGTCATCTTTCTTCCTCACTACATCGACTTCCCAGCTGATGCTGTTTCTCTCTCTACTTATCTTTCACGGAATGTTCCTCTCTCTATTCCCTGTGTTTCTTCTCCTATGGACACCGTCACTGAATCTTACATGGCGGCTGCTATGGCCGCCTTGGGAGGTATTGGTATTATTCATTCTAATATGCCCCCTTCTCACCAAGCTGATATGGTCAGATCTGCTAAGTCTCGCCGTGTTCCTATTTTGTCTAATCCTGCCTTCAAGTCCCCTGATTCCAGGATTCTTGATCAATTCGATGATGATTCTCTGCCTTGTGTTTTAGTTACTGAGTCAGGTACTGCTAAGTCCAAGCTATTAGGCTATGTACTCAAGTCTGATTGGATGTCTTTAAGTGATAAAGATGCTAAGTTGGTTGATTATATGCGCACTGCTGATTCAAAAATCTGTGTGCCATGGAGTTATGACTTGGCACAGATTGATGCCTATTTTAGAAAAGAAGAGCGTGATTTTGTGTTGCTGGAGGAGGGTGGAGAGGTACTGGATGTGATCACCAGGCAAGAGGTGGAGAGGGTGAAGGGCTATCCCAAGTTAGGGAAGGGGACGGTGGGGTCTGATGGGAAATGGATGGTTGGGGCGGCCATTGGGACCAGGGAATCTGACAAAGAGAGACTGGAGCATTTGGTGAAAGCAGGGGTGAATGCAGTGGTGTTGGATAGCTCACAAGGGAACTCCATTTATCAGATTGAAATGATAAATTATATAAAGCGAAAATATCCAGAGTTGGATGTGATTGGTGGGAATGTCGTGACTATGAATCAGGCGCAAAATCTGATTAAGGCAGGTGCGGATGGATTGAGAGTTGGGATGGGATCTGGGTCAATATGCACCACCCAGGAAGTGTGTGCAGTTGGACGAGGACAGGCAACTGCTGTTTATAAGGTTTCATCTATAGCTGCACAAAGTGATGTTCCTGTAATTGCAGATGGTGGCATTTCAAACTCTGGGCATATTGTTAAGGCTTTAACACTTGGGGCATCTACTGTAATGATGGGAGGGTTTTTAGCTGGAAGTACTGAAGCTCCTGGGACCTATGAGTATAAGGATGGTTATCGAATTAAAAAATATCGTGGCATGGGTTCTCTCGAAGCAATGACTAAAGGAAGTGATCAAAGATACTTGGGAGATACAGCTAAGCTAAAAATTGCACAAGGAGTTGTGGGAGCAGTTGCTGATAAAGGCTCTGTTTTGAGGTTATTGCCTTATACCATGCAAGCAGTCAAACAAGGGTTCCAAGATCTTGGTGCTTCCTCTCTCCAGTCTGCTCATGATTTGTTGAGATCAAAGACTCTGAGGCTTGAGGTTCGGACAGGTGCTGCGCAAGTTGAANNNNNNNNNNNNNNNNNNNTTACGAGAAGAAAGCCTTTTGAATTTAAAATCCTCTAGCATTTATTTCTACTGCTAATGATTAGTTTCCTCCTGCACTTGAGATCTAGAGCATGAGCAACATTTGAGTCGACTGCACTGATTCTGCTATGGCAATTTGCTAGCAGTTTTTTCATAAAGTTGAGGTGGTTTTTGGCTGAAATAACCAGAAGCAGAAATCCCAATTGTTGTTTGTTCTTTGCTGATGGGTATGCAAGCACTCTTCTACTTGTATCTTAGCCAATTTTGGGATTTAATTGAATAA

>Unigene7907_All

TTTTTTTTCTCCTTTCTTTTTGGTTCTAGTGATGGTCTGGTATTTCTTAGTCAGTGTTCATGTCGCAGATCATTTCTTTACAAATGTTAAAGGTATTCTAATATATGTGAAGTCGGTTGTGCTCAAAAAAAAAGGAGAAAAAAAAAGTCCACAGTCCAATTTCAAGCAAATAAACAATTGTCAGACGAGCACAATTCCTACACTAAACTAAAGAACTGTTTCCCCATTTAAAATTCCTCCGGACTATTTCTACTCGCGTTTATTCCACACATTACTGTACACAGGAAAAAAGAAAATGGGAAAGATGGTGGTGTTATGGGCATTGACTGACTTAAAAGATGCCAACAACCTTTTCATCCCTTGTTTCCCTCCGTCATTTGAGTCGCAACACAAAACAAAGCCAAGAATGAATTGCCGCCTAGCAAAATTTTGCCAGGGATGTGATTACCAAATAAGAAGAGGATTCAGCTGTCTCTACTAGGGTGAATTGAATCCTCCATCTTGGCATCCACTTACCATACTGGAGTTCACTGGATAGAAAGTGGCCTCCGTGAGGCCTGGTTTGAGGAAATGCTCCCTCGTTGTTGACCTGAACAGTCTCCCTCCCCCTTTTCACCTGAGTGTGAATGATGTGTTGAGGTCTGCAAGCCATTGATGTTGTGGTGAAAAGTGCCTCCATTTACATGGTTTTTAGCTTCAGAAGTTCCATGCACTTGTGGTGCTCCTCTTCCCTCTTGTTGACCACGGGGGAAAATCCACACCTCAGCAGAATCTGAAGGCAATGGCTCTGAACCTGATGAACTGCCAGACTGAGATGAGGGGACTGGACCAGAATCATTGACCTTTCTTTCTGTGGTGTTCTTAGAAGCATCTGCATGAGTTTTCTTTGCAAAACGTCCTCCAGTACCCCTTGCCCTTCTCATAGCATGCTGGTGCCGAGACTCATGAAGATATGGCTTTCTAACTTTTATCAGCTTCTTCTCAACCTCTGCTTTTGCACGTGCCTGTCTTCGCCTCAGAATTCCTTGGTATTGTTTGGCATTTACATAAACAGGTTCCTGGGCGATCTCATGAGGTAAAGGCATTCTTGGATGATGCATTGCTATCACATGAGGATAACTCAAAGGTTGATGCCCATAAGCTGCCATCATTCCACCGTAATATGGATCCTGGTATGGATTTGATGCACATGCAATAGAGTGACCAACAAGTTCAAGCTGAGGAGGATGTGTCAGGGACTCATCATGCAACAAAGTCACACTTGAAGCAACATGGTGCATATTTCTGTGTTCTTGTCCACTATTCACAACTGATCGTGAAGATGCAGTCGCTTGTGATTCTTTGCTCACACCATCATCATCATCCTCATTCAGTCTTTCATTTGACAGTGACTGATCATCATTTGACAGTGAACCATTTGGGCCATTAGATGNAAATTGGTTGCATTTTCCCCCAGCACAGCTGGAGGCACGGTATTACAACTGACACTACGCCACCATGGTTCAGAGTGAACTGCATATGGTTGGATGACACGTGGATCTGGTTCCAGTTGATTTGCACTTACAGGTTTNNNNNNNNNNNNNNNNNNNNNNNNNNNNNNNNNNNNNNNNNNNNNNNNNCATGGTAACCAATTGGCTAATGATAGTGGATTGGAACAAAACCCTAGATTTTGGGGTTATT

>Unigene7915_All

TTACAACAAAATAATAACAACCTCTCTCTAATTTCCTCTCTTATGTGGGTTGTGAGGAAATCATACACCAGGCGATGCATCAACCCCTTTTGTTTGTGATTTTATTACTCAAAGTCTTCCATTTCTCTATTGCTTTCATGGTTCCATATTTACTATTATTTTTAGCTGTGCAAGTTCATTGACATGTACTGAAATGTGAATCTTGTCCAAAAGCTCCAAGCATTCTACAAGCTACTTCCATTAGCTTCCCTCTCCTGGATTTGGTGACAACCTTGACGGCGGCTGTCGGTTTTTTTACGGTCTCTTTCTCCTCCTCCGGAAACAAAACCCCATCAATTCCCTGCACTGAAATCCTGCCGTCGGTATAAATGTCTGGATCGAACAAATACGCCGAAGTATCACCCGATCCAAACTTCACCGACCCATCAGCCTCCTGAGCCACTACCTTGTGAGGCAAACGCAGCGTATCGTATTTTATTTTCCCAAATCTCCTTACTGCATTGTACATGCTCTCTTCCGTCTGGTACTCCGGTATAATATGGTAGTAAATGATCTGCTCCGGCGCACCCGGTTCGCTTAGCTGGTCCGTCGTTAGCTTGGCCATGGCTTCGTCGTTTGGTGCCAAGACGGTGAGAACGTACCCTTCTGAAACCAGCCGCCCCATTTCGGTGGCCAATGAGGTTAAGTTCACTAAGATATCCGCCAGTTCATTGTACCCACCGTAATGTAATAGTGTCTGGATGAAGTCCTTGACCTGGCTTTCACCGTCAAAGTGACGGTGAGGTCCGCCGGGGCCTGGAGCCGGCGCCGGGGCCAGAGAAGGACCTGGAGCCATGGCATCGTAAATGGGTAGCACTGGCGGTGCCCCAGCCGGCACAGGTGCTGCTGGTTTCTTCAAACGGTGGGTTCTGGGATCTACCTCCGGCGCACCCTCTGGTAGCACTGCGGATATGGATCTGAGATTCCTCCTCCGGTTGAAGTCTTCTTGTACAGATTGCGGGATAAGAAGGCGCTCGATCCCGTGGATAACACCGTCGGGGCGGATAACGTCGTCGGCTCGGATAATCTCGGCGGAGTCAACGAGTTTCTTACCAGAAGTCTTGCTGATTAAGTGAAGATGATCATTGCACAAAGTGGTATGCCTAGAAGGCTTAGATTTTTCAGTGGGCCAATCATTGGATCCGACCCGTTTAGGAATTACATGGAACATCAAGAGGGTCTGGAGAGACTTGATATTACCAGGTTCGAGCAAGAAACGCTTAAACTCAGGGTCAAGTTNNNNNNNNNNNNNNNNNCTGGGCGCAAAAATGGTGATATTGTGGTTGCCGACAGCTTCCTCAAGGGTCTGAAGAAGAAGAGCCTTTTCAACGAGTTCAGCCAATTCAGTATAATGCGAGTCAAGAAGCGCCACAAGAACAGAATTAGAGTTAATACCAGTATTATTATTATTGTTATTGCTAGAAGAAGAAGAAGAAGAAGAAGGGAGCTTAGAAGATGGGTATTGAGGCAATGCAGAGAAAAGGAGGAAAAAGCAGAGAGTAAAGGAGACACCATAGATGTGAGGATCCATGGTGCTAAAAGAAAGAAAAATGTGAAGCAGCAGAAGGAAGAAAGATGAGAG

>Unigene7916_All

CTTTCCTTCCAATGCGACCAAAAACCAGATAAATCCTCCTGTCGTGCCTTCCAATCCTTCACTCCCTTTCTTTTGCTTGTCTAAAAATTCCGTTTGGCAGAAGTTGCCGACATCTCTGTTTGGTGTTCATTGAGGCCCTCTTCTCAGTTTTTCTCTTTGCATCACTATCTTCACCAGGTTGGTTAGATCTACACTTCTTTTTATAGCCCATNNNNNNNNNNNNNNNNNNNNGTTTCGTTCTGTATTGTTCAGCTCTTGATTTTCTGAATTCTAGCTGAAATTTAAATTGGATCTTTGCTTATACACTCGTTTCGTGATCTGGGTCTTCCAGATCTGGCACTATTCGCCACAGAACTCCAAAATGGACCATTTTTCTAATTCTAAGATCTGGGTCTTGACCCTTAGCTTGATTTTCCAATCTGTATATGGGTTTTACCTTCCGGGTAGCTACCCCCACAAATACAACGTCGGTGAAGCTTTATCAGTGAAAGTGAATTCCATTACTTCTATTGATACCGAGATGCCATTTAGCTATTATAGTCTACCCTTTTGCCAGCCTGCAGAGGGTATCAAGGATAGTGCTGAAAATCTCGGTGAGCTCCTTATGGGAGATAGGATTGAGAATTCCCCATATAGGTTTAAGATGCATGTGAATGAGAGTGAGATCTTTTTGTGTCGAGCTGACCCCTTATCTACTGAGAACTTTAAGCTTTTGAAGAAGAGGATTGATGAGATGTATCAGGTTAATTTGATTCTTGATAATTTGCCGGCAATAAGGTATACTAAGAAGGAGGCATACTTGTTGAGGTGGACAGGATATCCTGTTGGAATTAAGGTTCAGGATGCCTATTATGTTTTTAACCATTTGAAGTTTAAAGTTCTTGTTCATAAGTATGAGGAGGCCAATATGGCACGTGTGATGGGGACTGGAGATGGTGCTGAGGTCATTCCAACAATTGGGAATGGAGGATCTGATATTCCTGGGTACATGGTTGTGGGATTTGAAGTAGTTCCTTGCAACTTCATGCATAATGTTCAGTCCGTGAAGAACTTGAAAATGTATGGCAAATACCCATCTCCAATTAAGTGCGATCCAACCACTGTGGCTATGCCAATTAGGGAGAATGAGCCAATTGTTTTTACCTATGAGGTCACCTTTGAGGAGAGTGATATAAAGTGGCCATCGAGATGGGATGCCTATTTGAGGATGGAGGGGTCAAAGGTCCATTGGTTCTCGATCATGAATTCTCTGATGGTTATCACCTT

>Unigene7936_All
[truncated: 4,311,658 more chars]
